# Supplementary figures and images for: S-phase PARylation of microprotein RSMC enhances the function of Sororin in sister chromatid cohesion (part 1 of 3)
Source: EMBO J. 2025 Nov 19;45(1):278–309. doi: 10.1038/s44318-025-00641-8 (PMC12759081; doi:10.1038/s44318-025-00641-8)

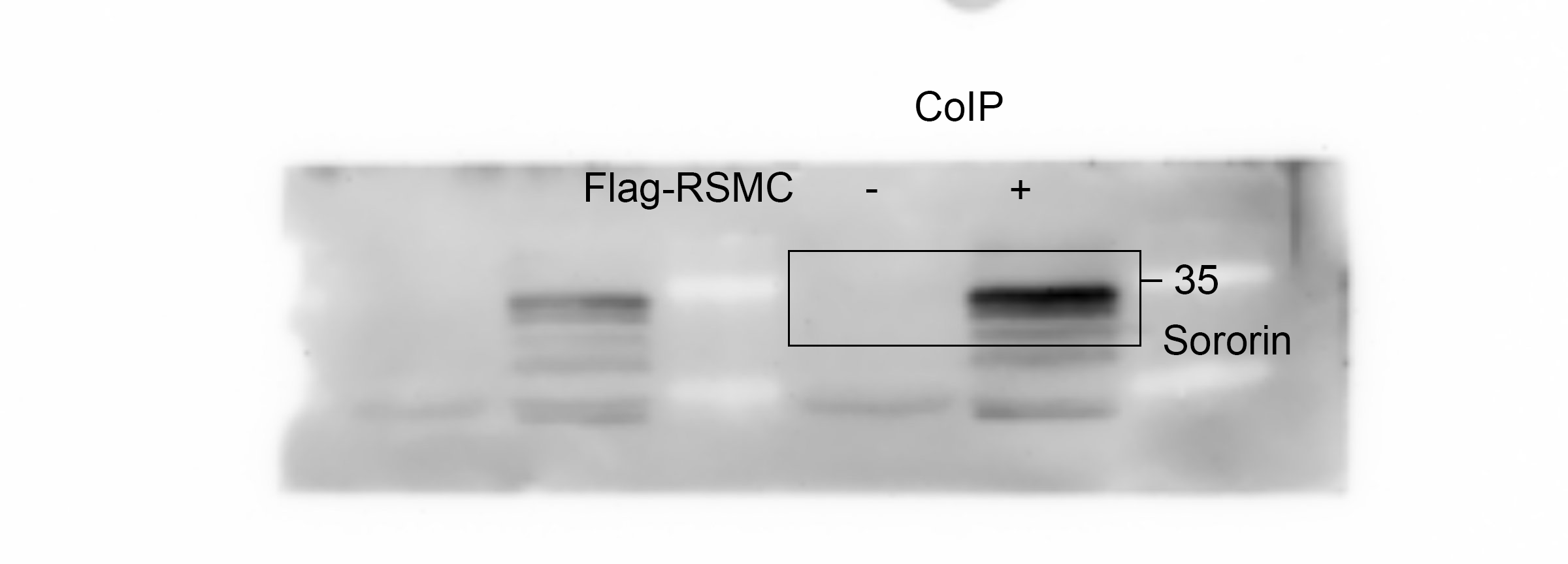

Supplement: Supplementary file 2 — Source data Fig. 1 [file 44318_2025_641_MOESM2_ESM.zip › EMBOJ-2025-120713R_SourceDataForFigure1/EMBOJ-2025-120713R_SourceDataForFigure1B/1. CoIP Sororin SourceData.tif]

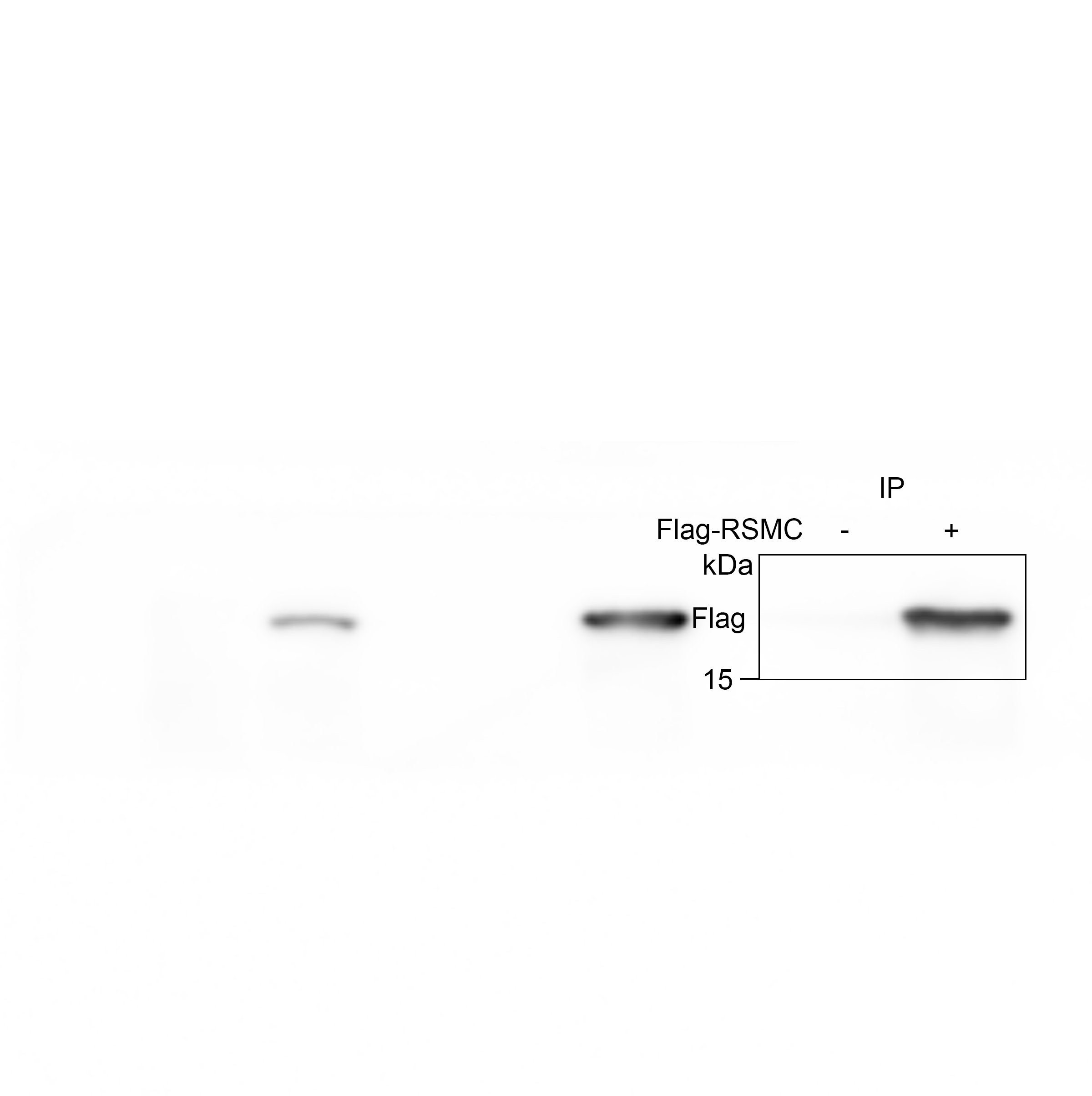

Supplement: Supplementary file 2 — Source data Fig. 1 [file 44318_2025_641_MOESM2_ESM.zip › EMBOJ-2025-120713R_SourceDataForFigure1/EMBOJ-2025-120713R_SourceDataForFigure1B/2. IP Flag-RSMC SourceData.tif]

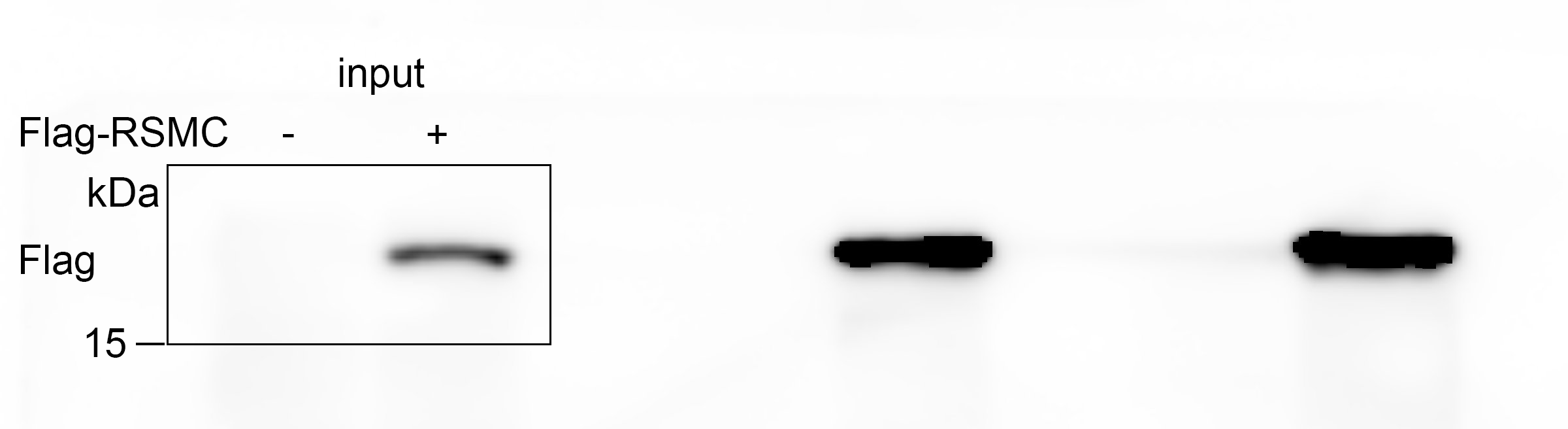

Supplement: Supplementary file 2 — Source data Fig. 1 [file 44318_2025_641_MOESM2_ESM.zip › EMBOJ-2025-120713R_SourceDataForFigure1/EMBOJ-2025-120713R_SourceDataForFigure1B/3. Input Flag-RSMC SourceData.tif]

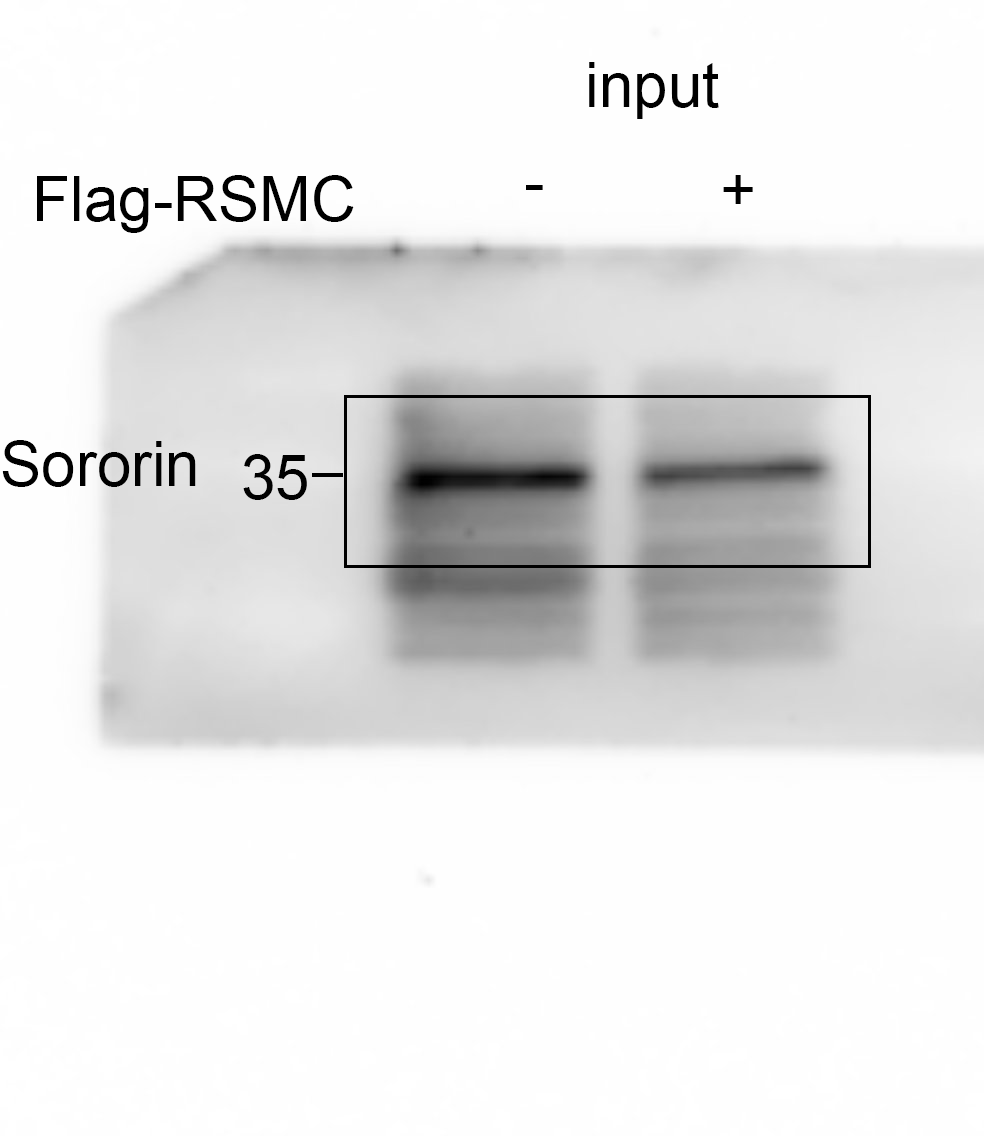

Supplement: Supplementary file 2 — Source data Fig. 1 [file 44318_2025_641_MOESM2_ESM.zip › EMBOJ-2025-120713R_SourceDataForFigure1/EMBOJ-2025-120713R_SourceDataForFigure1B/4. Input SororinsourceData.tif]

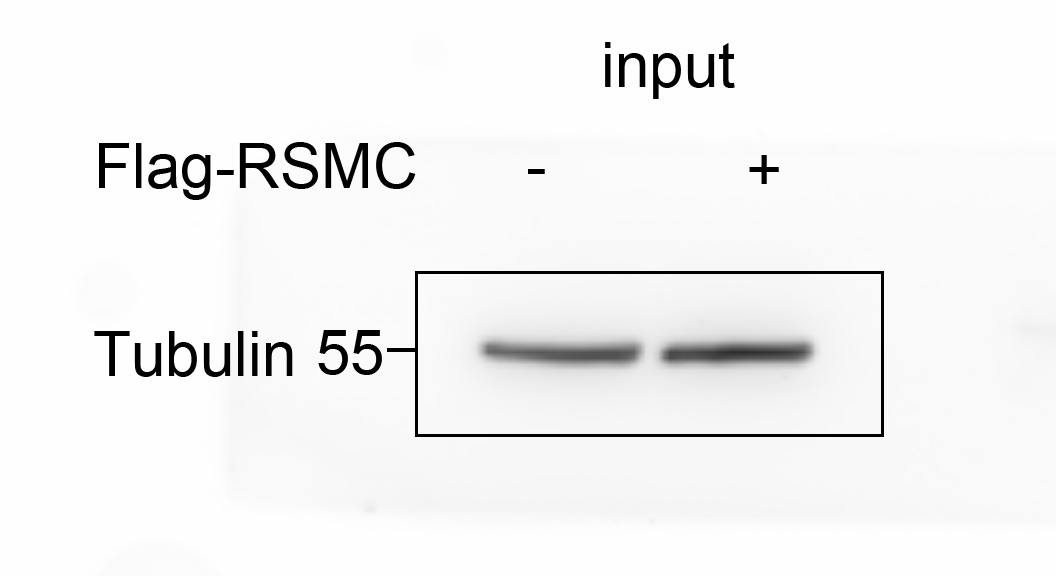

Supplement: Supplementary file 2 — Source data Fig. 1 [file 44318_2025_641_MOESM2_ESM.zip › EMBOJ-2025-120713R_SourceDataForFigure1/EMBOJ-2025-120713R_SourceDataForFigure1B/5. Input Tubulin SourceData.tif]

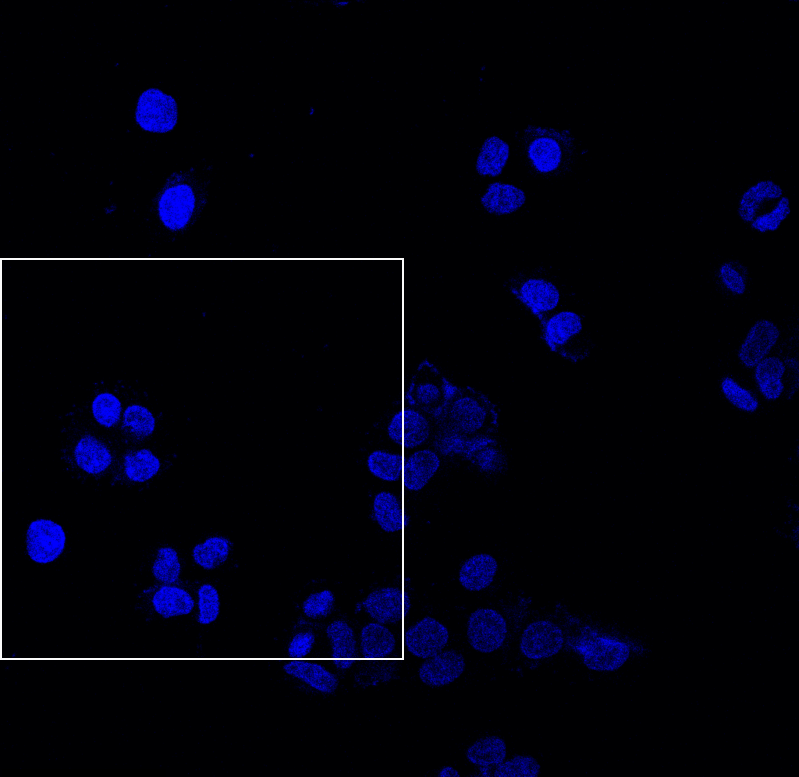

Supplement: Supplementary file 2 — Source data Fig. 1 [file 44318_2025_641_MOESM2_ESM.zip › EMBOJ-2025-120713R_SourceDataForFigure1/EMBOJ-2025-120713R_SourceDataForFigure1C/1-RSMC-3Flag/RSMC-3Flag-DAPI-sourceData.tif]

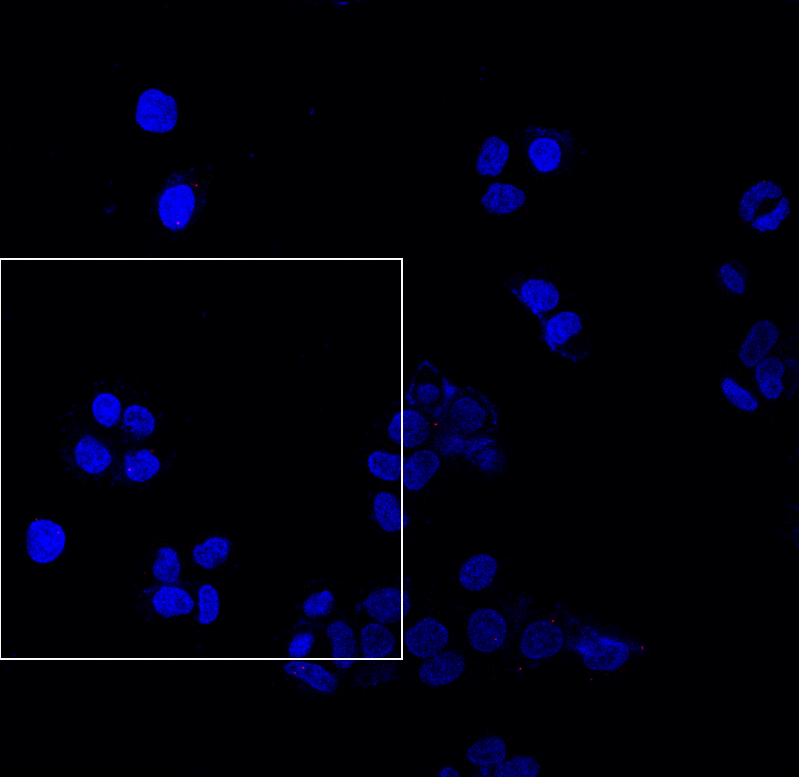

Supplement: Supplementary file 2 — Source data Fig. 1 [file 44318_2025_641_MOESM2_ESM.zip › EMBOJ-2025-120713R_SourceDataForFigure1/EMBOJ-2025-120713R_SourceDataForFigure1C/1-RSMC-3Flag/RSMC-3Flag-merge-sourceData.tif]

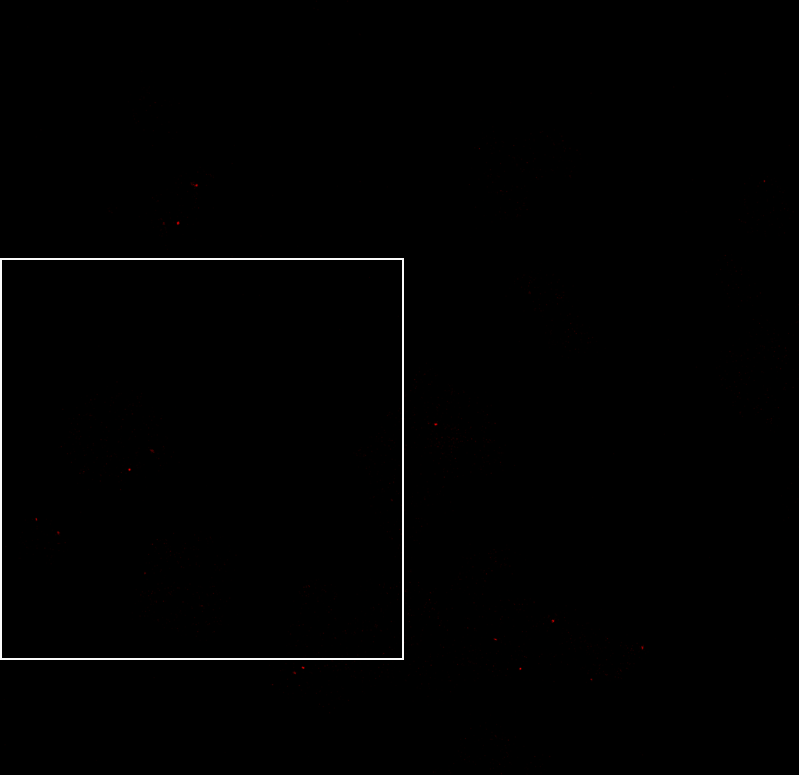

Supplement: Supplementary file 2 — Source data Fig. 1 [file 44318_2025_641_MOESM2_ESM.zip › EMBOJ-2025-120713R_SourceDataForFigure1/EMBOJ-2025-120713R_SourceDataForFigure1C/1-RSMC-3Flag/RSMC-3Flag-PLA-sourceData.tif]

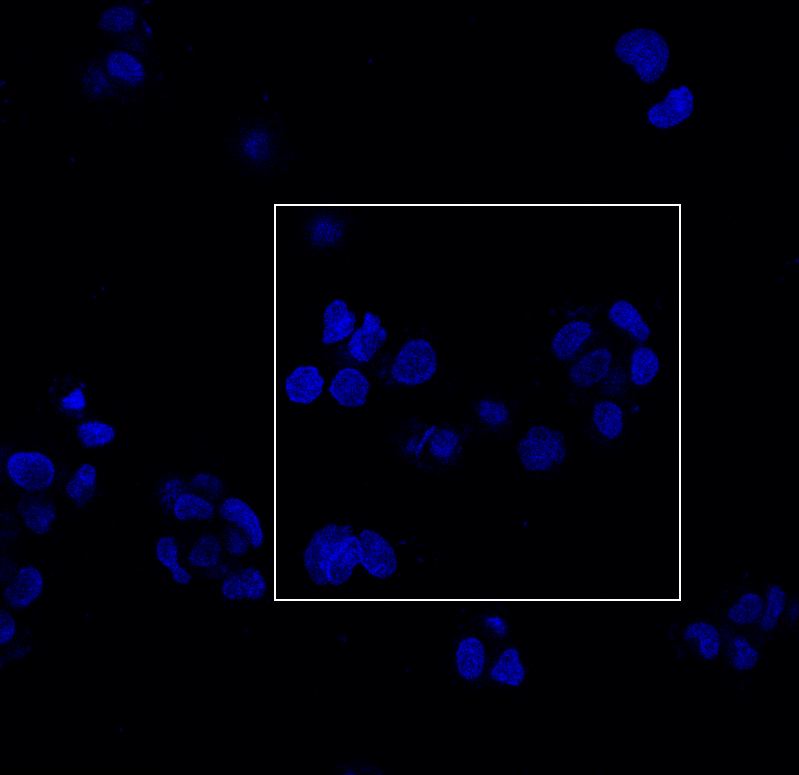

Supplement: Supplementary file 2 — Source data Fig. 1 [file 44318_2025_641_MOESM2_ESM.zip › EMBOJ-2025-120713R_SourceDataForFigure1/EMBOJ-2025-120713R_SourceDataForFigure1C/2-Sororin/Sororin-DAPI-sourceData.tif]

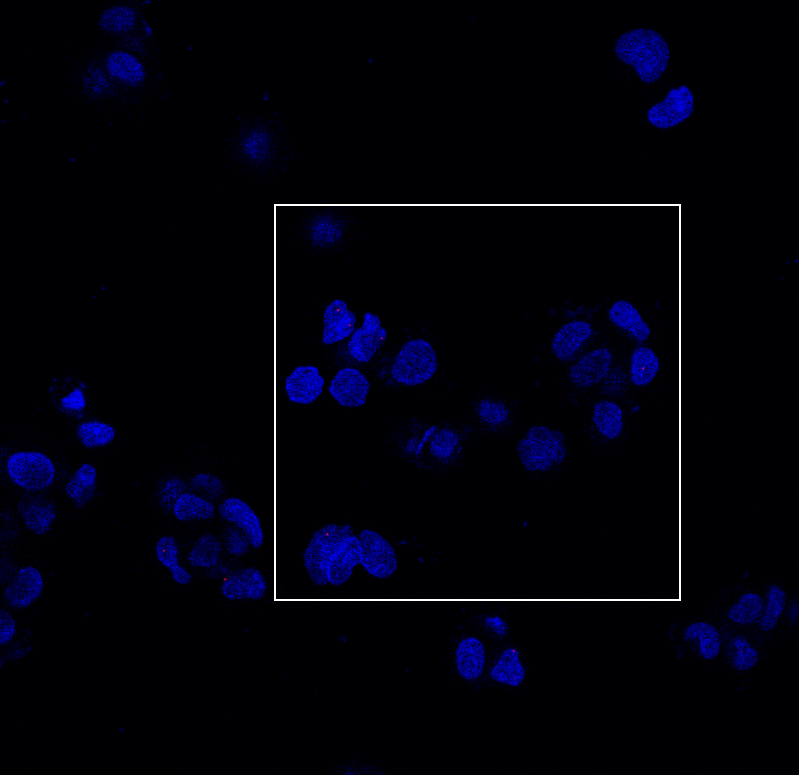

Supplement: Supplementary file 2 — Source data Fig. 1 [file 44318_2025_641_MOESM2_ESM.zip › EMBOJ-2025-120713R_SourceDataForFigure1/EMBOJ-2025-120713R_SourceDataForFigure1C/2-Sororin/Sororin-merge-sourceData.tif]

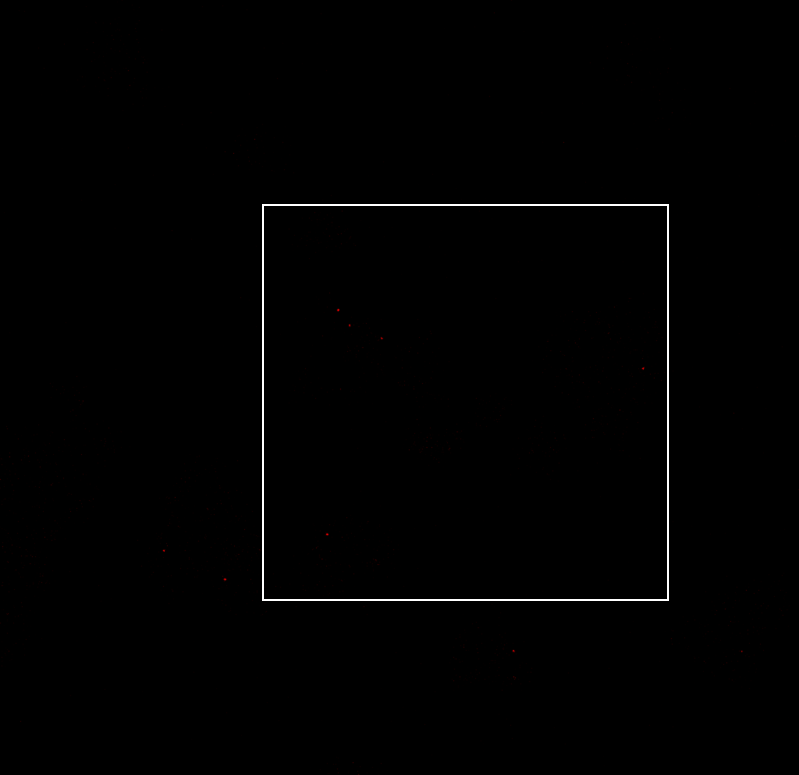

Supplement: Supplementary file 2 — Source data Fig. 1 [file 44318_2025_641_MOESM2_ESM.zip › EMBOJ-2025-120713R_SourceDataForFigure1/EMBOJ-2025-120713R_SourceDataForFigure1C/2-Sororin/Sorroin-PLA-sourceData.tif]

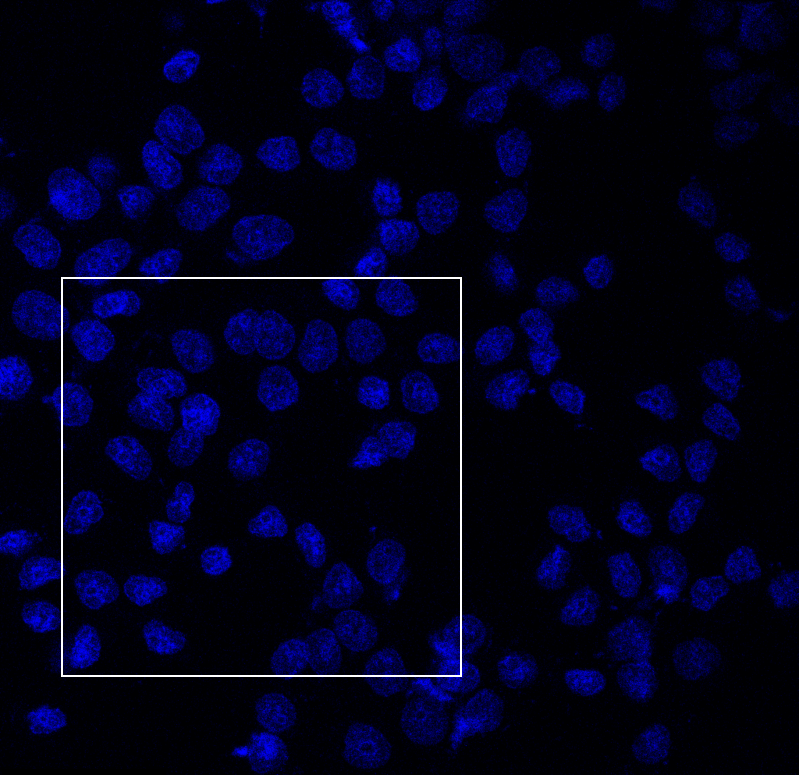

Supplement: Supplementary file 2 — Source data Fig. 1 [file 44318_2025_641_MOESM2_ESM.zip › EMBOJ-2025-120713R_SourceDataForFigure1/EMBOJ-2025-120713R_SourceDataForFigure1C/3-SMC3/SMC3-DAPI-sourceData.tif]

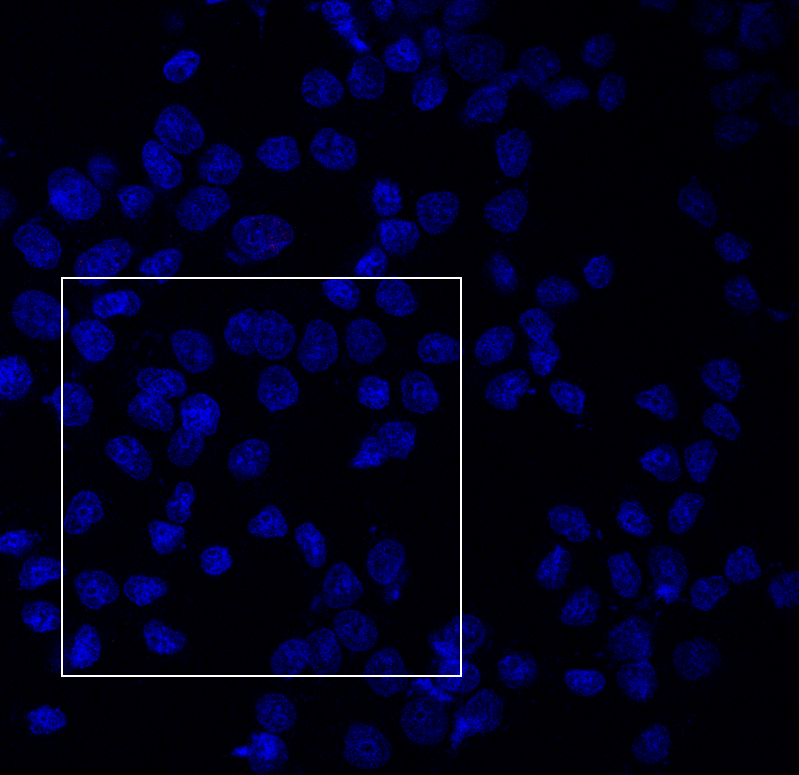

Supplement: Supplementary file 2 — Source data Fig. 1 [file 44318_2025_641_MOESM2_ESM.zip › EMBOJ-2025-120713R_SourceDataForFigure1/EMBOJ-2025-120713R_SourceDataForFigure1C/3-SMC3/SMC3-merge-sourceData.tif]

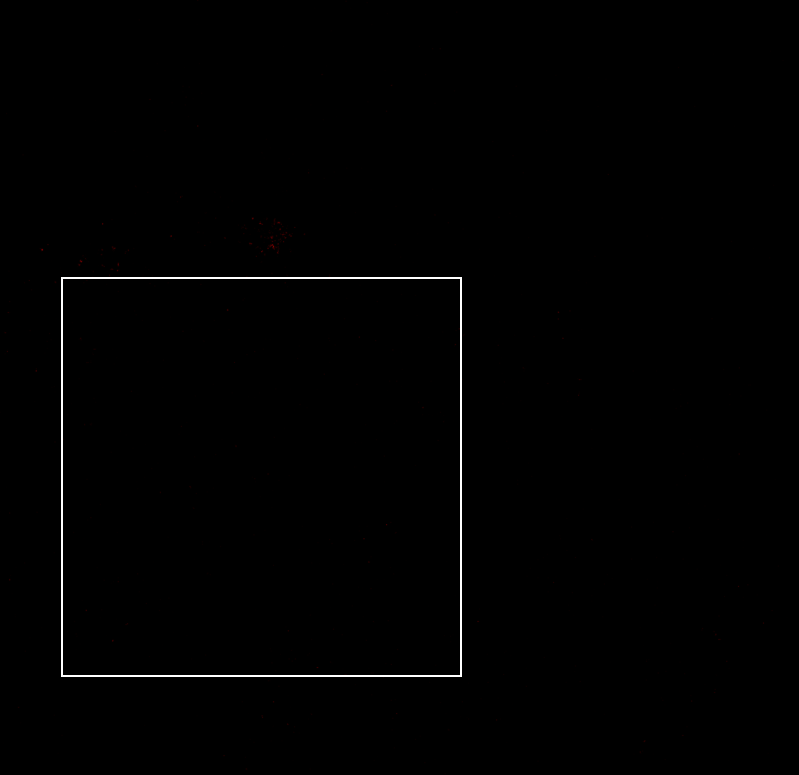

Supplement: Supplementary file 2 — Source data Fig. 1 [file 44318_2025_641_MOESM2_ESM.zip › EMBOJ-2025-120713R_SourceDataForFigure1/EMBOJ-2025-120713R_SourceDataForFigure1C/3-SMC3/SMC3-PLA-sourceData.tif]

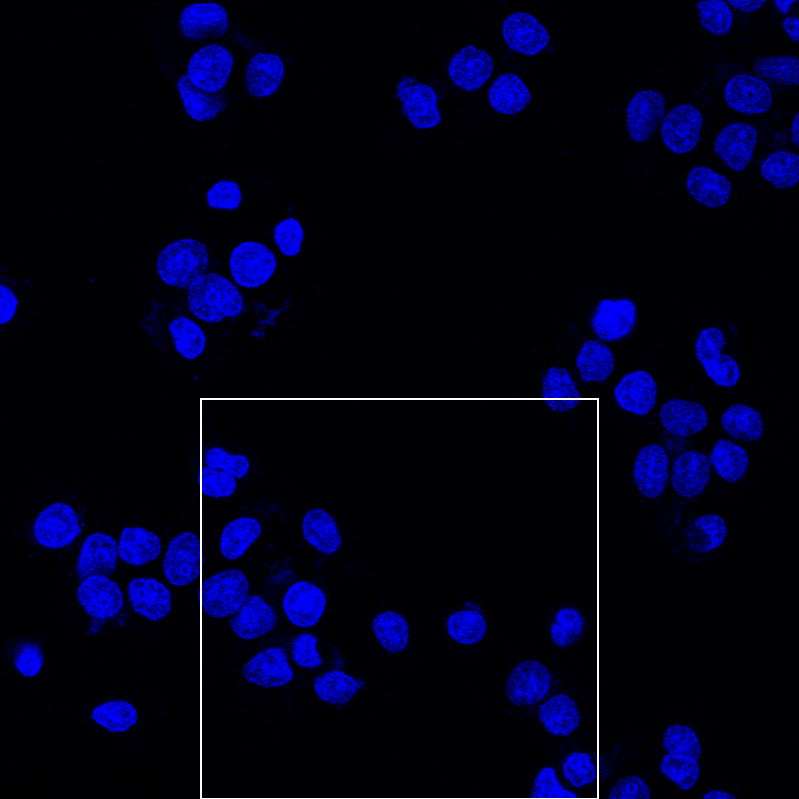

Supplement: Supplementary file 2 — Source data Fig. 1 [file 44318_2025_641_MOESM2_ESM.zip › EMBOJ-2025-120713R_SourceDataForFigure1/EMBOJ-2025-120713R_SourceDataForFigure1C/4-Sororin+RSMC-3Flag/RSMC3Flag-Sororin-DAPI-sourceData.tif]

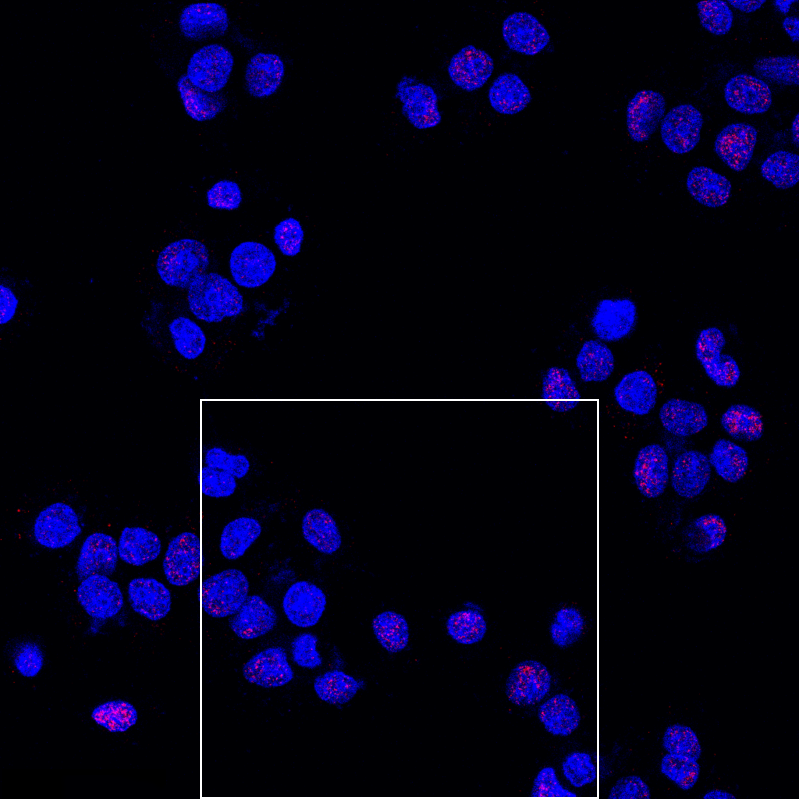

Supplement: Supplementary file 2 — Source data Fig. 1 [file 44318_2025_641_MOESM2_ESM.zip › EMBOJ-2025-120713R_SourceDataForFigure1/EMBOJ-2025-120713R_SourceDataForFigure1C/4-Sororin+RSMC-3Flag/RSMC3Flag-Sororin-merge-sourceData.tif]

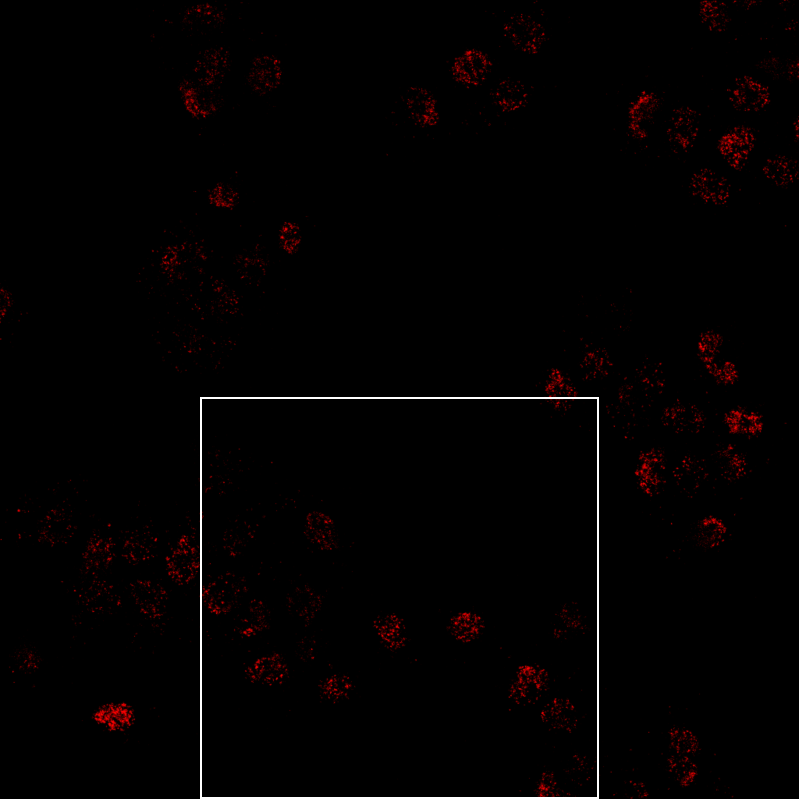

Supplement: Supplementary file 2 — Source data Fig. 1 [file 44318_2025_641_MOESM2_ESM.zip › EMBOJ-2025-120713R_SourceDataForFigure1/EMBOJ-2025-120713R_SourceDataForFigure1C/4-Sororin+RSMC-3Flag/RSMC3Flag-Sororin-PLA-sourceData.tif]

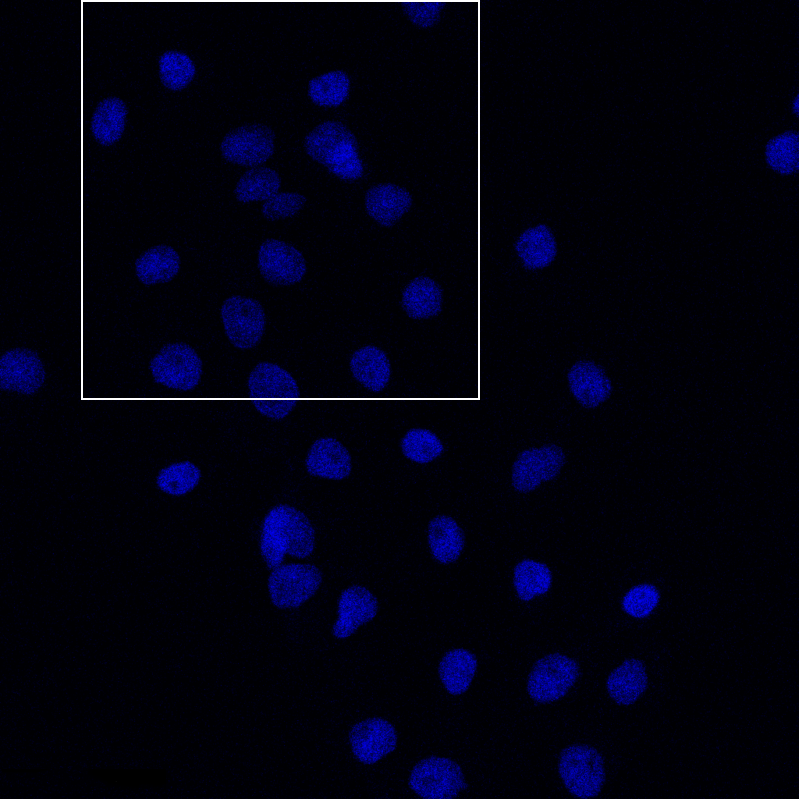

Supplement: Supplementary file 2 — Source data Fig. 1 [file 44318_2025_641_MOESM2_ESM.zip › EMBOJ-2025-120713R_SourceDataForFigure1/EMBOJ-2025-120713R_SourceDataForFigure1C/5-SMC3+RSMC-3Flag/SMC3+RSMC-3Flag-DAPI-sourceData.tif]

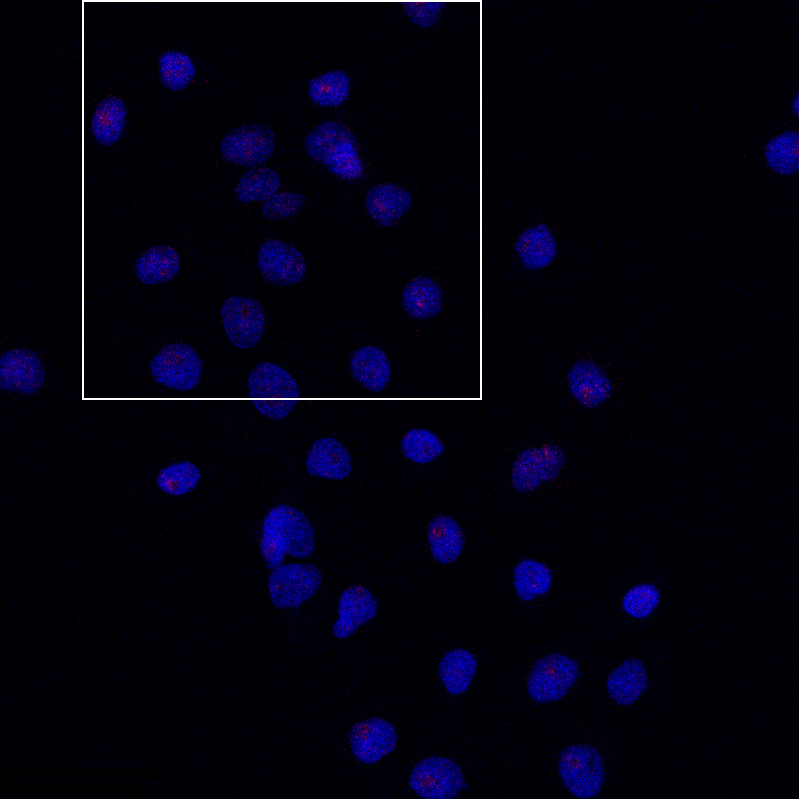

Supplement: Supplementary file 2 — Source data Fig. 1 [file 44318_2025_641_MOESM2_ESM.zip › EMBOJ-2025-120713R_SourceDataForFigure1/EMBOJ-2025-120713R_SourceDataForFigure1C/5-SMC3+RSMC-3Flag/SMC3+RSMC-3Flag-merge-sourceData.tif]

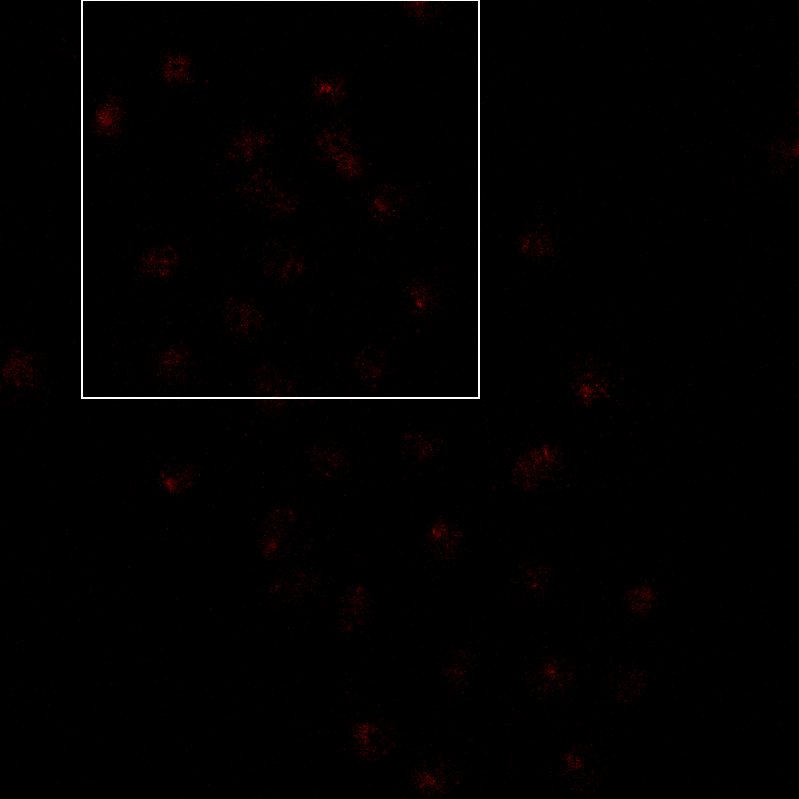

Supplement: Supplementary file 2 — Source data Fig. 1 [file 44318_2025_641_MOESM2_ESM.zip › EMBOJ-2025-120713R_SourceDataForFigure1/EMBOJ-2025-120713R_SourceDataForFigure1C/5-SMC3+RSMC-3Flag/SMC3+RSMC-3Flag-PLA-sourceData.tif]

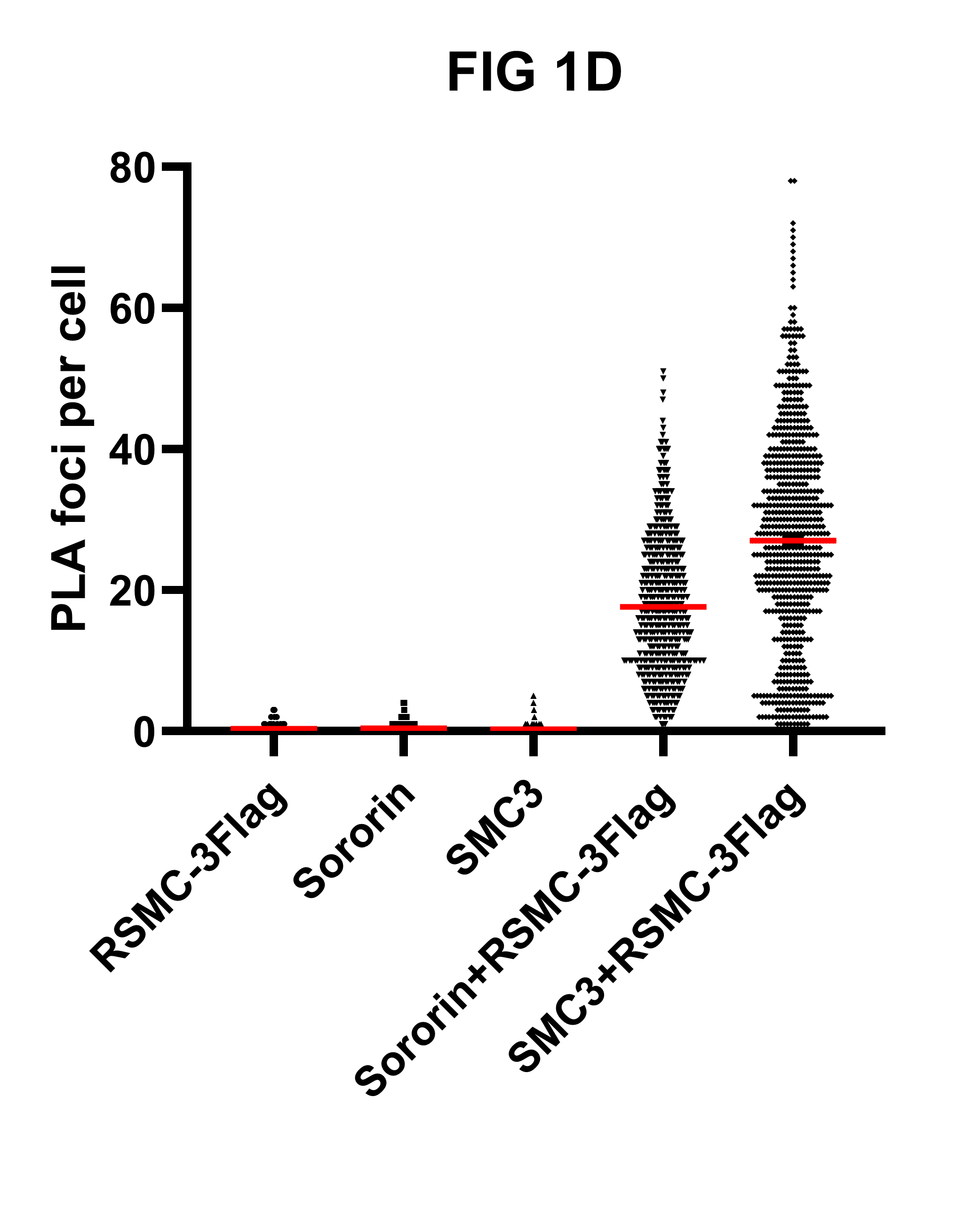

Supplement: Supplementary file 2 — Source data Fig. 1 [file 44318_2025_641_MOESM2_ESM.zip › EMBOJ-2025-120713R_SourceDataForFigure1/EMBOJ-2025-120713R_SourceDataForFigure1D/FIG 1D before PS.tif]

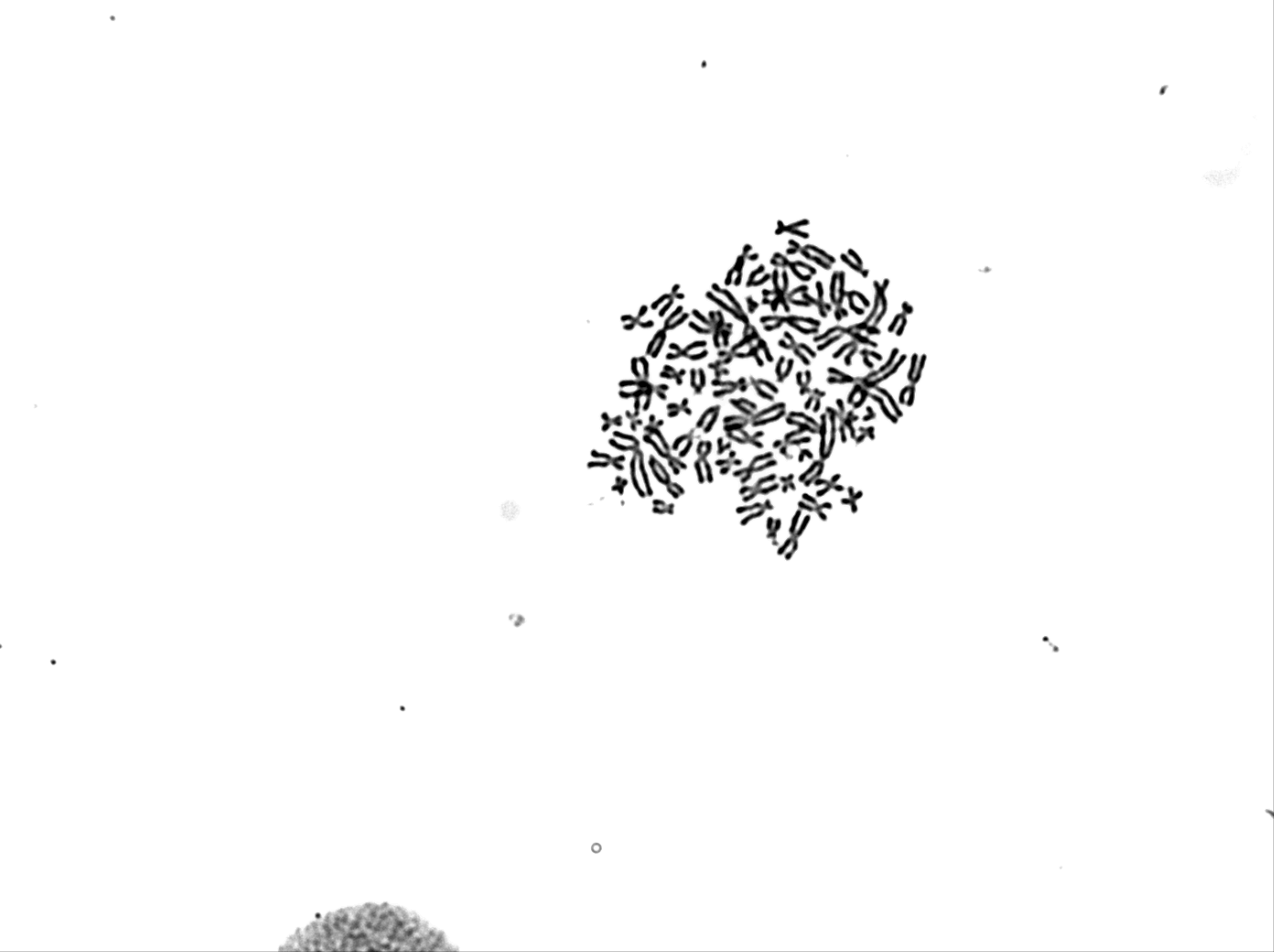

Supplement: Supplementary file 2 — Source data Fig. 1 [file 44318_2025_641_MOESM2_ESM.zip › EMBOJ-2025-120713R_SourceDataForFigure1/EMBOJ-2025-120713R_SourceDataForFigure1E/arm open.tif]

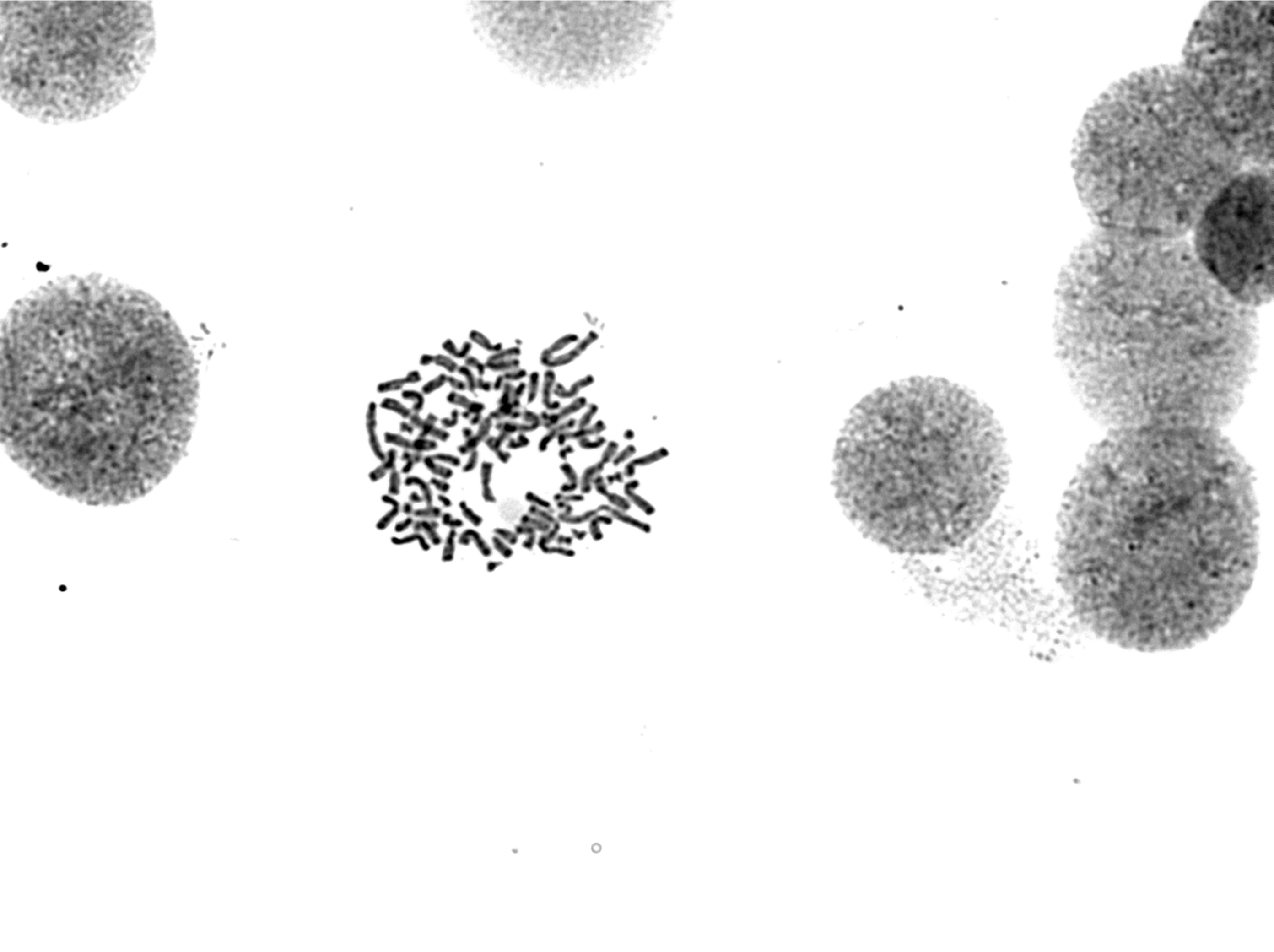

Supplement: Supplementary file 2 — Source data Fig. 1 [file 44318_2025_641_MOESM2_ESM.zip › EMBOJ-2025-120713R_SourceDataForFigure1/EMBOJ-2025-120713R_SourceDataForFigure1E/closed.tif]

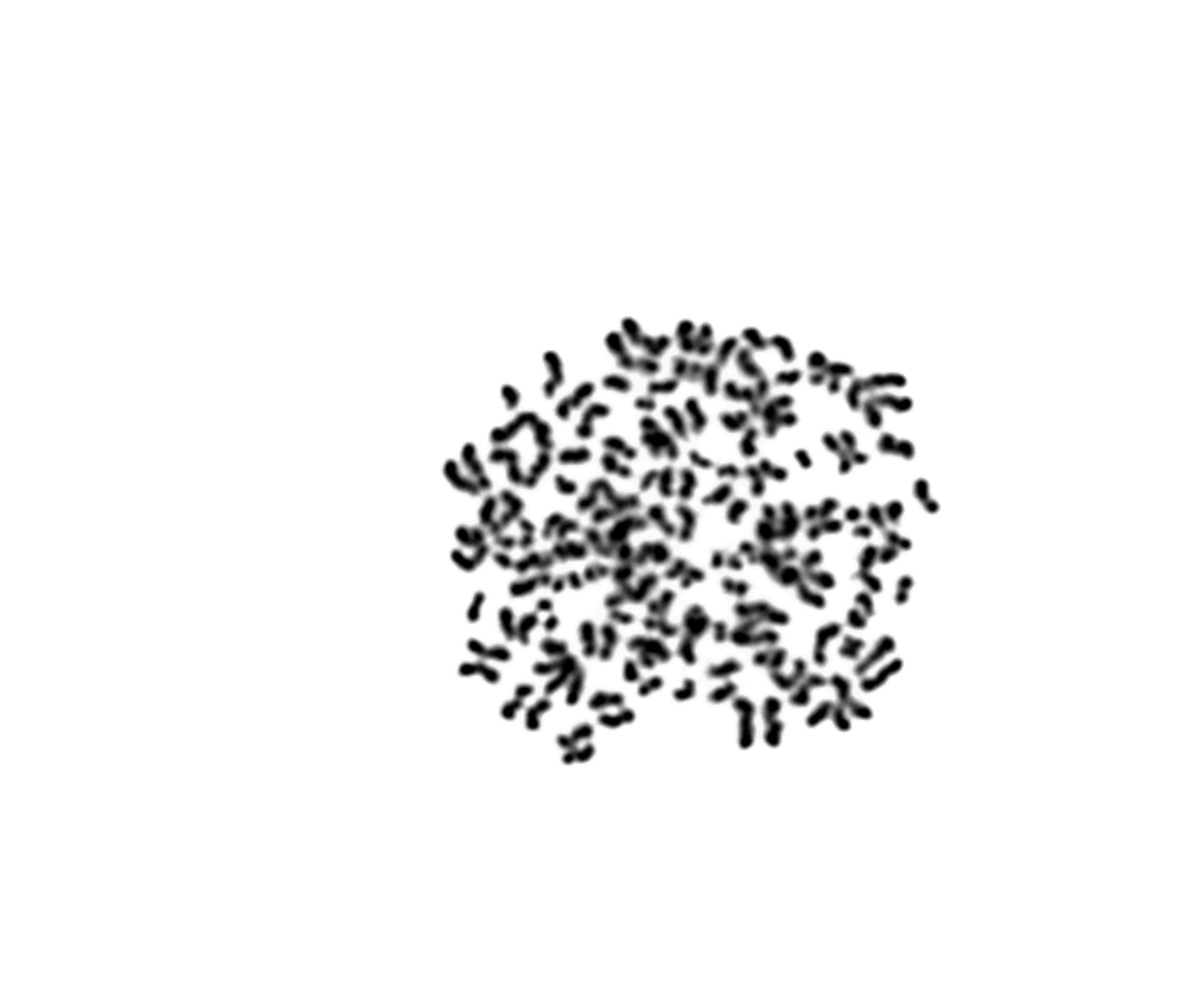

Supplement: Supplementary file 2 — Source data Fig. 1 [file 44318_2025_641_MOESM2_ESM.zip › EMBOJ-2025-120713R_SourceDataForFigure1/EMBOJ-2025-120713R_SourceDataForFigure1E/railroad.tif]

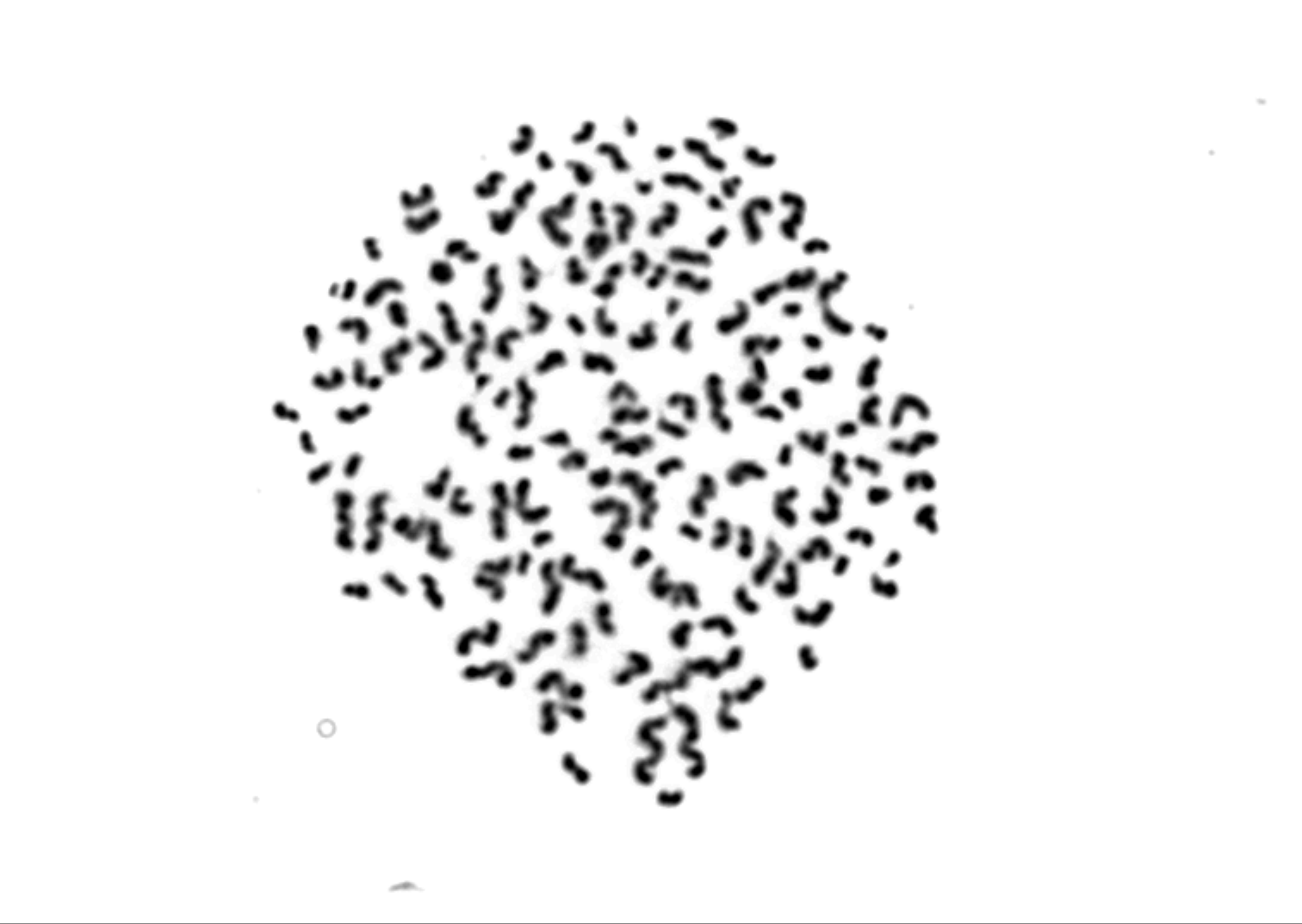

Supplement: Supplementary file 2 — Source data Fig. 1 [file 44318_2025_641_MOESM2_ESM.zip › EMBOJ-2025-120713R_SourceDataForFigure1/EMBOJ-2025-120713R_SourceDataForFigure1E/separated.tif]

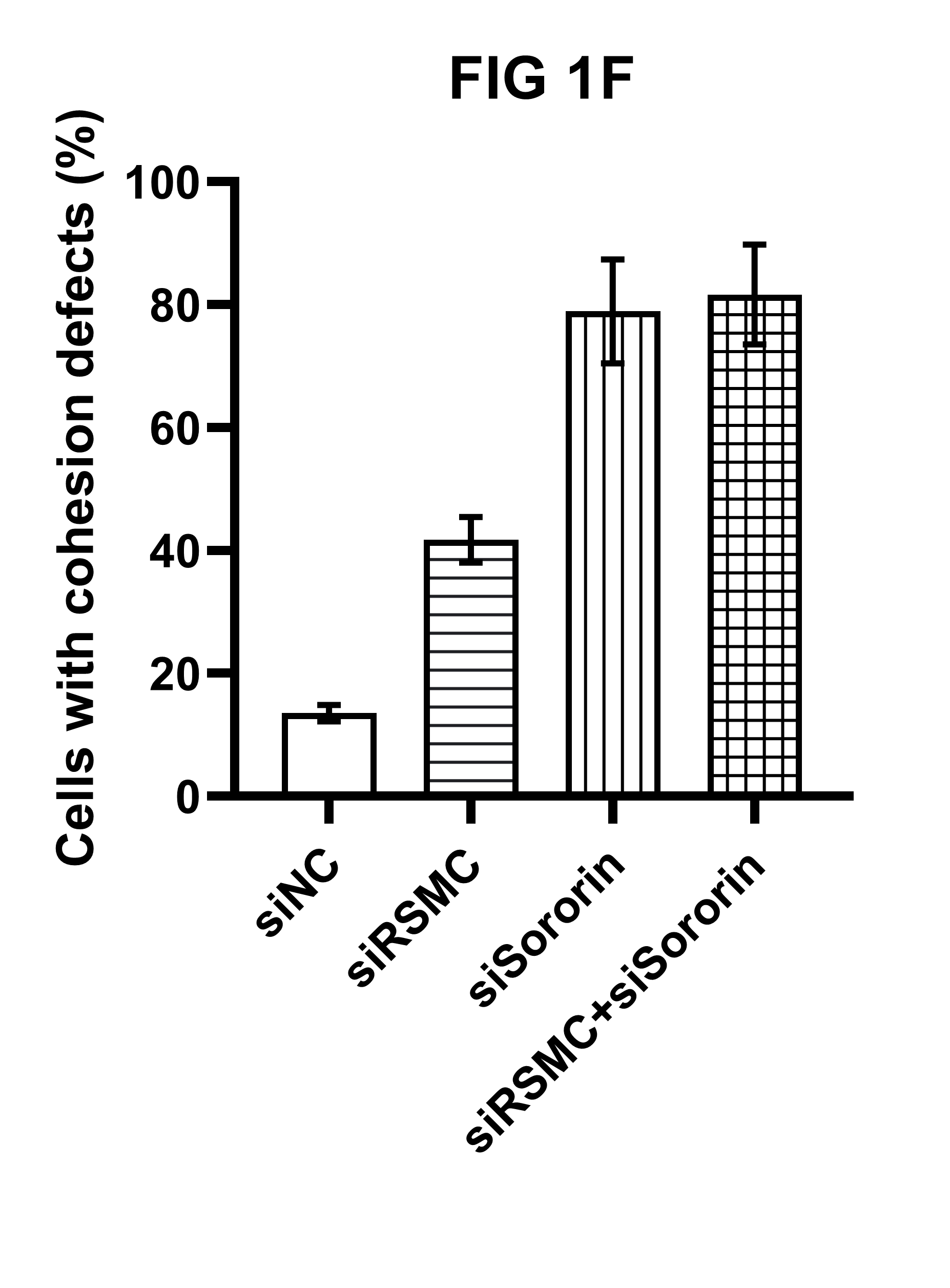

Supplement: Supplementary file 2 — Source data Fig. 1 [file 44318_2025_641_MOESM2_ESM.zip › EMBOJ-2025-120713R_SourceDataForFigure1/EMBOJ-2025-120713R_SourceDataForFigure1F/FIG 1F before PS.tif]

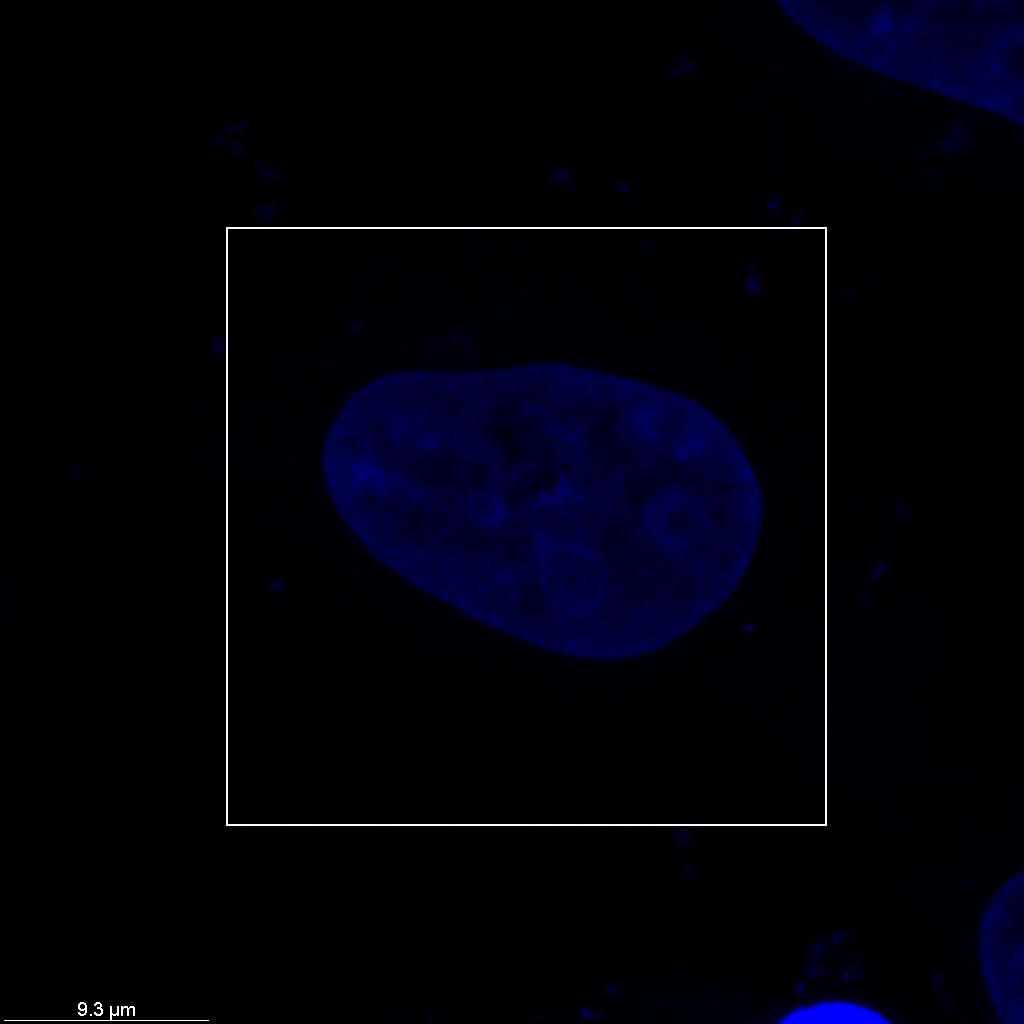

Supplement: Supplementary file 2 — Source data Fig. 1 [file 44318_2025_641_MOESM2_ESM.zip › EMBOJ-2025-120713R_SourceDataForFigure1/EMBOJ-2025-120713R_SourceDataForFigure1G/siNC/DAPI.tif]

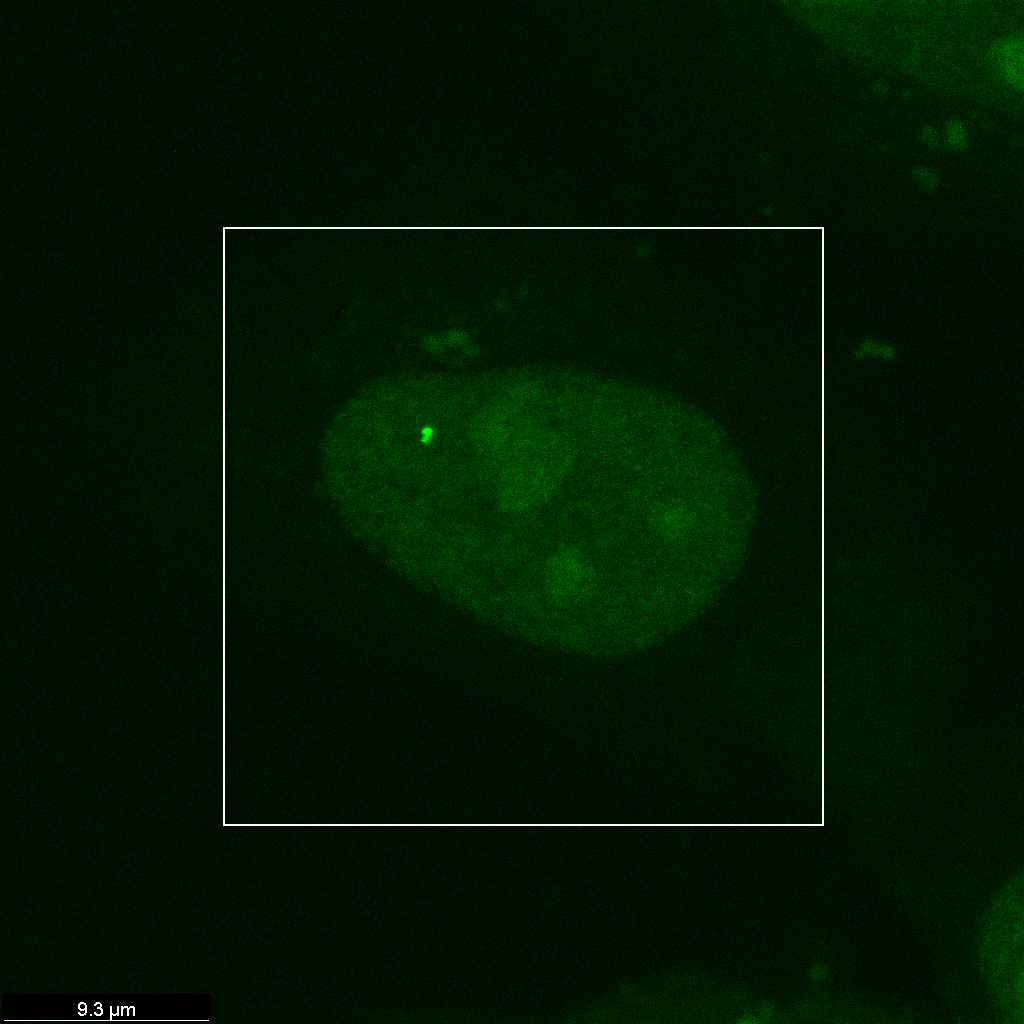

Supplement: Supplementary file 2 — Source data Fig. 1 [file 44318_2025_641_MOESM2_ESM.zip › EMBOJ-2025-120713R_SourceDataForFigure1/EMBOJ-2025-120713R_SourceDataForFigure1G/siNC/GFP.tif]

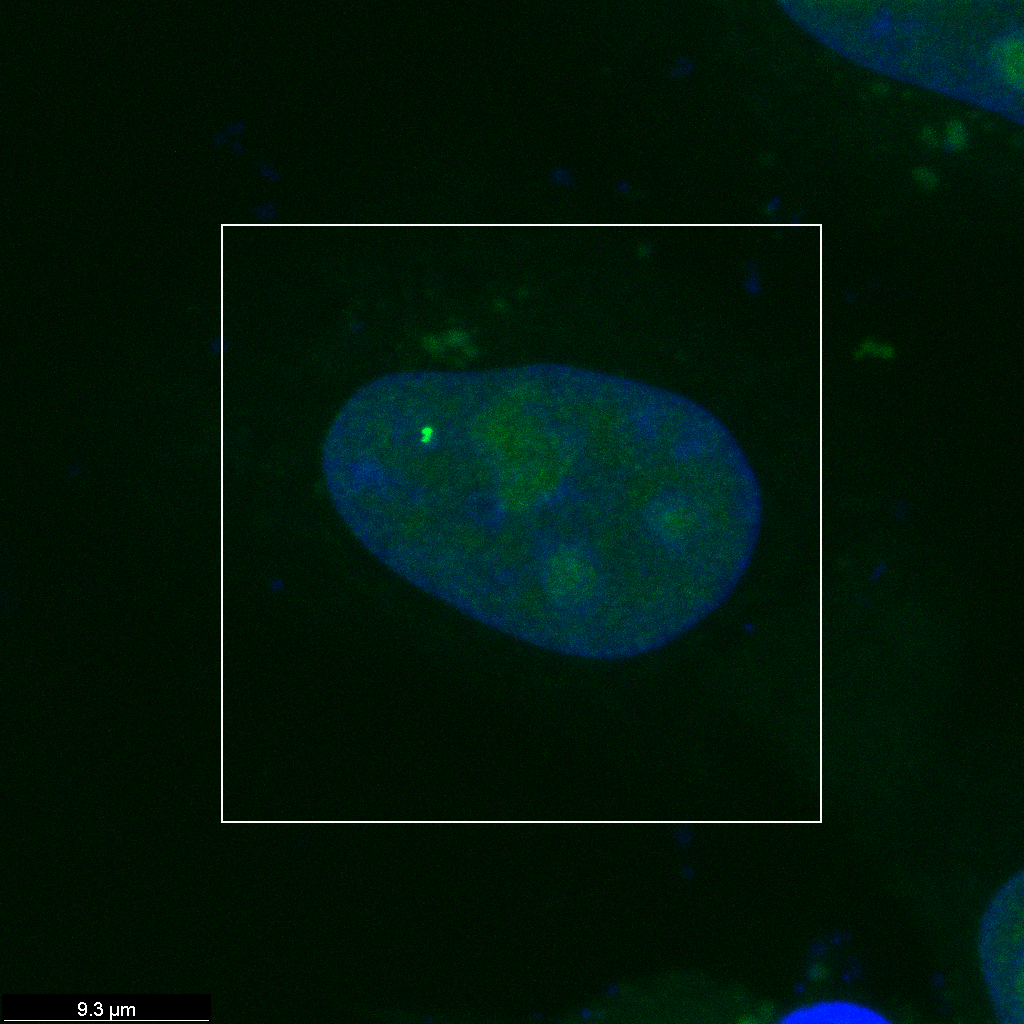

Supplement: Supplementary file 2 — Source data Fig. 1 [file 44318_2025_641_MOESM2_ESM.zip › EMBOJ-2025-120713R_SourceDataForFigure1/EMBOJ-2025-120713R_SourceDataForFigure1G/siNC/merge.tif]

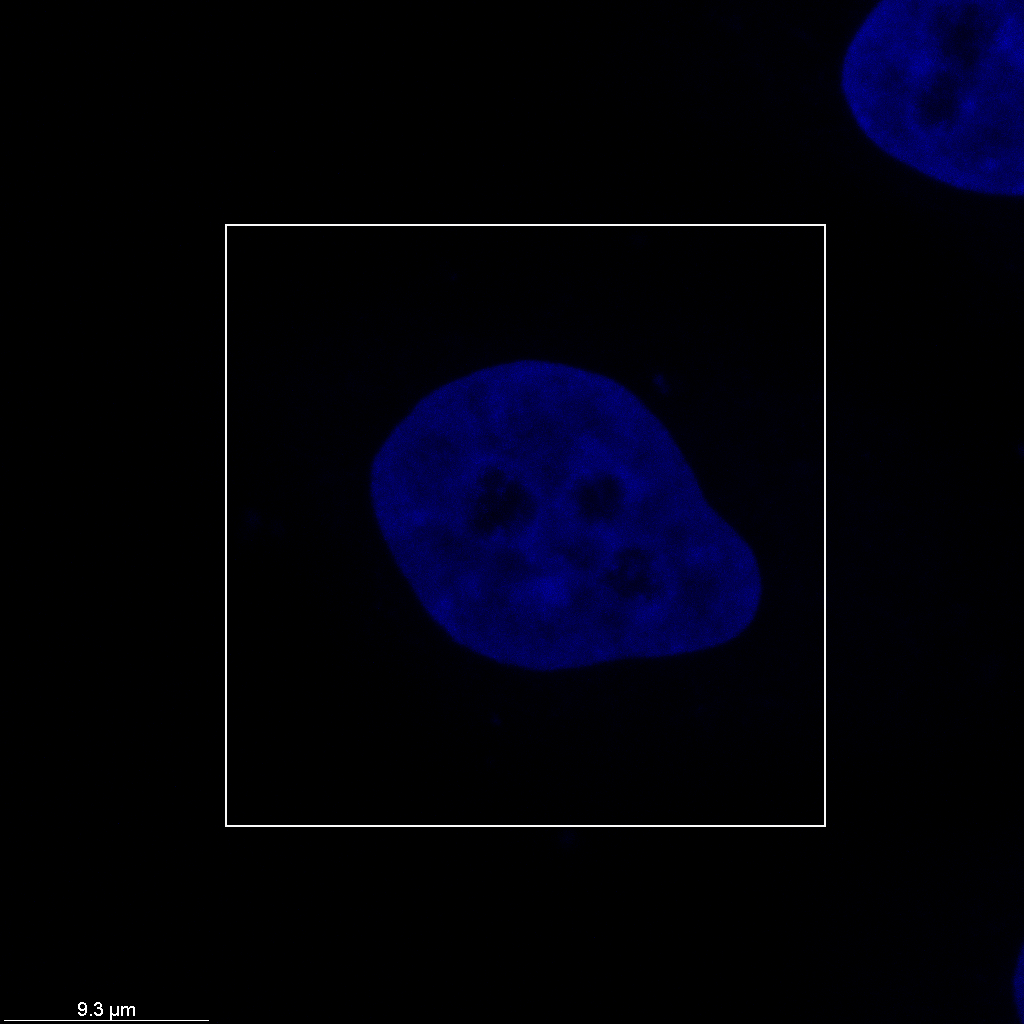

Supplement: Supplementary file 2 — Source data Fig. 1 [file 44318_2025_641_MOESM2_ESM.zip › EMBOJ-2025-120713R_SourceDataForFigure1/EMBOJ-2025-120713R_SourceDataForFigure1G/siRSMC/DAPI.tif]

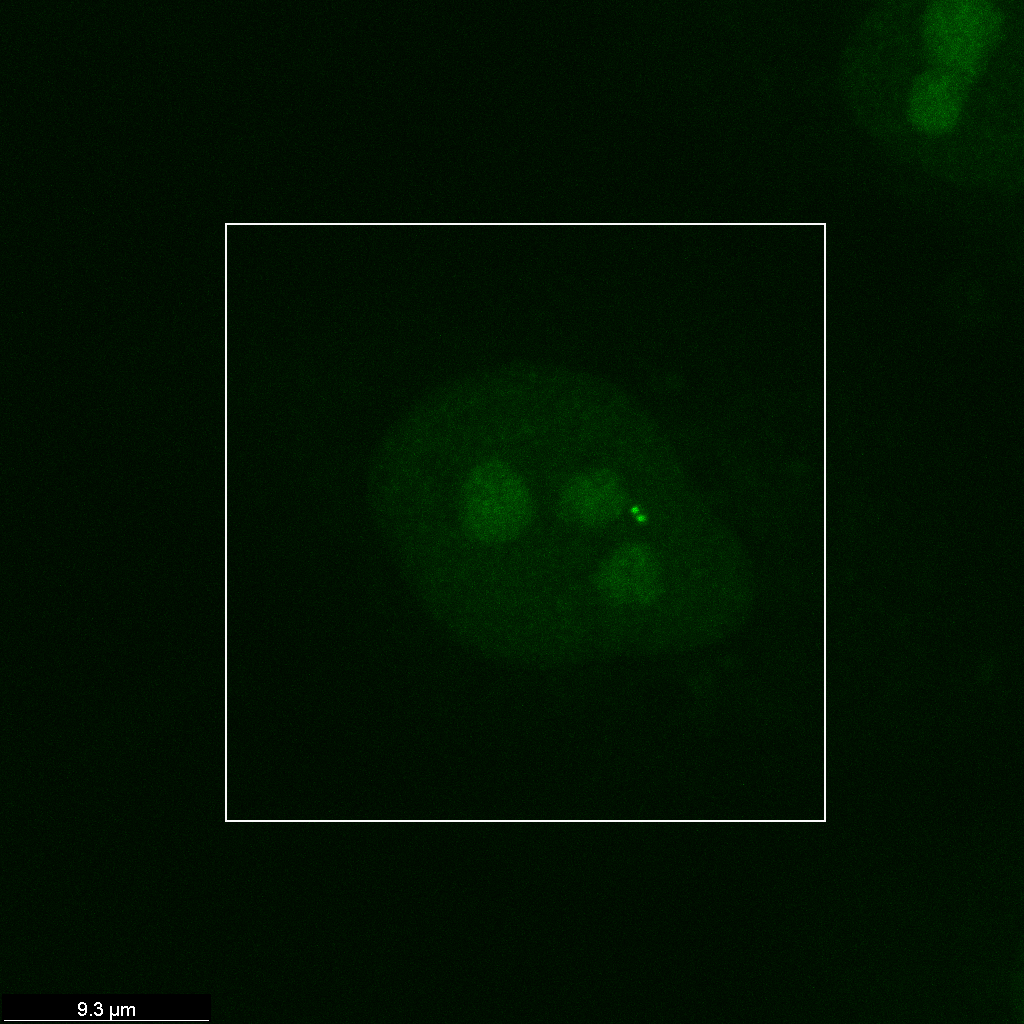

Supplement: Supplementary file 2 — Source data Fig. 1 [file 44318_2025_641_MOESM2_ESM.zip › EMBOJ-2025-120713R_SourceDataForFigure1/EMBOJ-2025-120713R_SourceDataForFigure1G/siRSMC/GFP.tif]

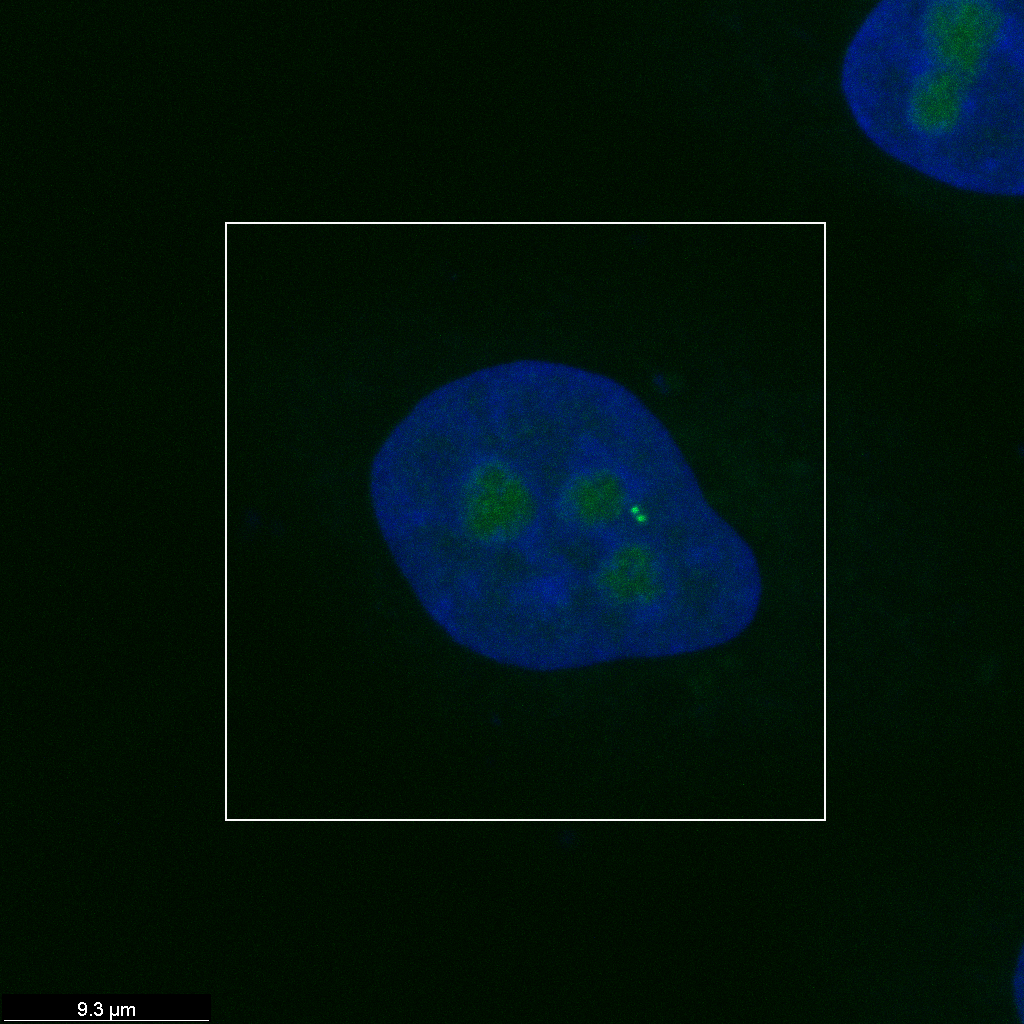

Supplement: Supplementary file 2 — Source data Fig. 1 [file 44318_2025_641_MOESM2_ESM.zip › EMBOJ-2025-120713R_SourceDataForFigure1/EMBOJ-2025-120713R_SourceDataForFigure1G/siRSMC/merge.tif]

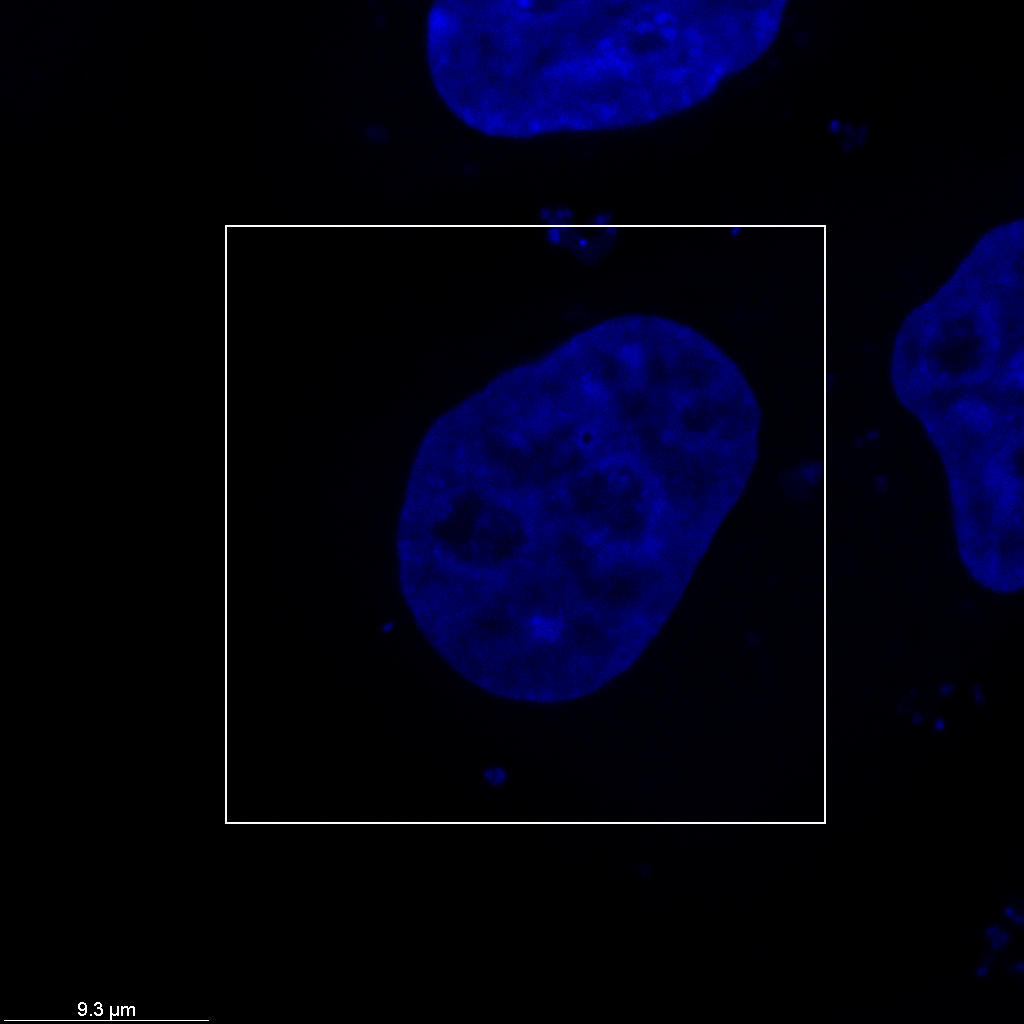

Supplement: Supplementary file 2 — Source data Fig. 1 [file 44318_2025_641_MOESM2_ESM.zip › EMBOJ-2025-120713R_SourceDataForFigure1/EMBOJ-2025-120713R_SourceDataForFigure1G/siSororin/DAPI.tif]

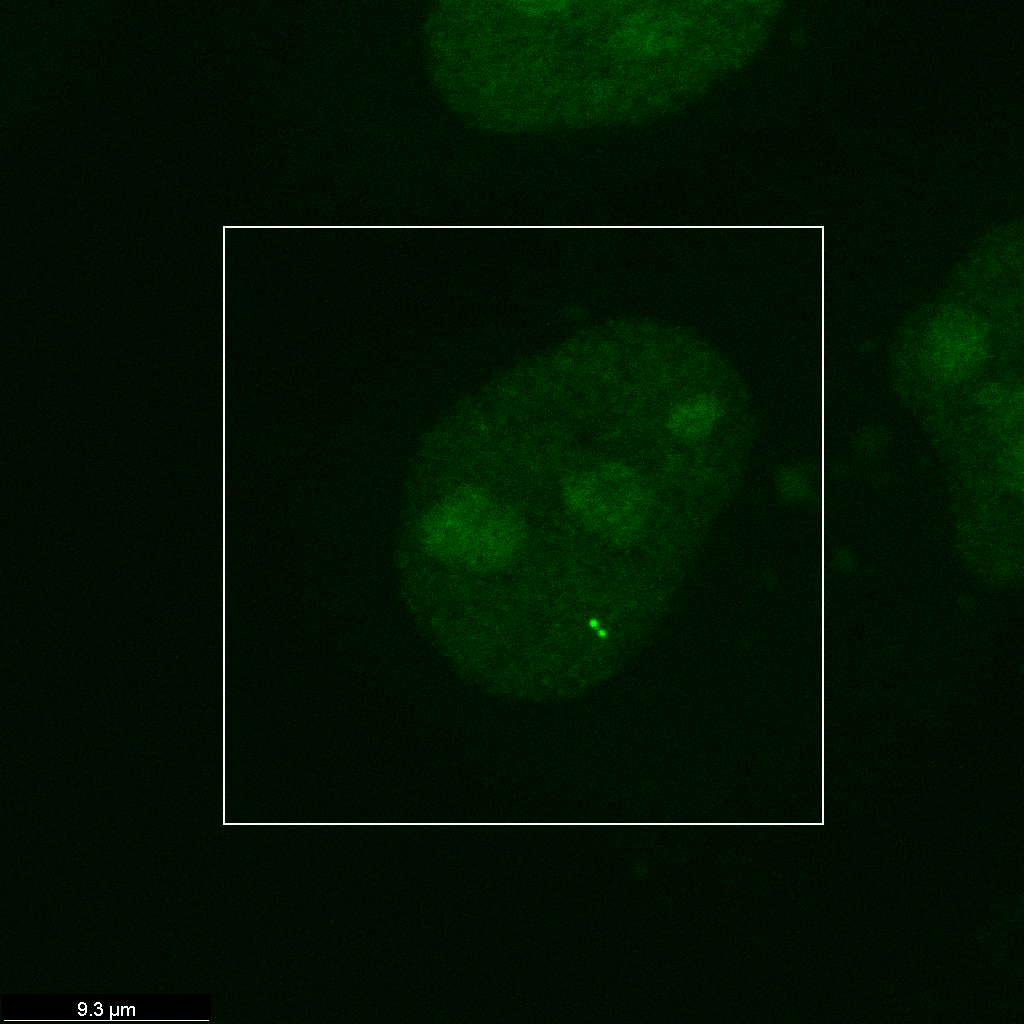

Supplement: Supplementary file 2 — Source data Fig. 1 [file 44318_2025_641_MOESM2_ESM.zip › EMBOJ-2025-120713R_SourceDataForFigure1/EMBOJ-2025-120713R_SourceDataForFigure1G/siSororin/GFP.tif]

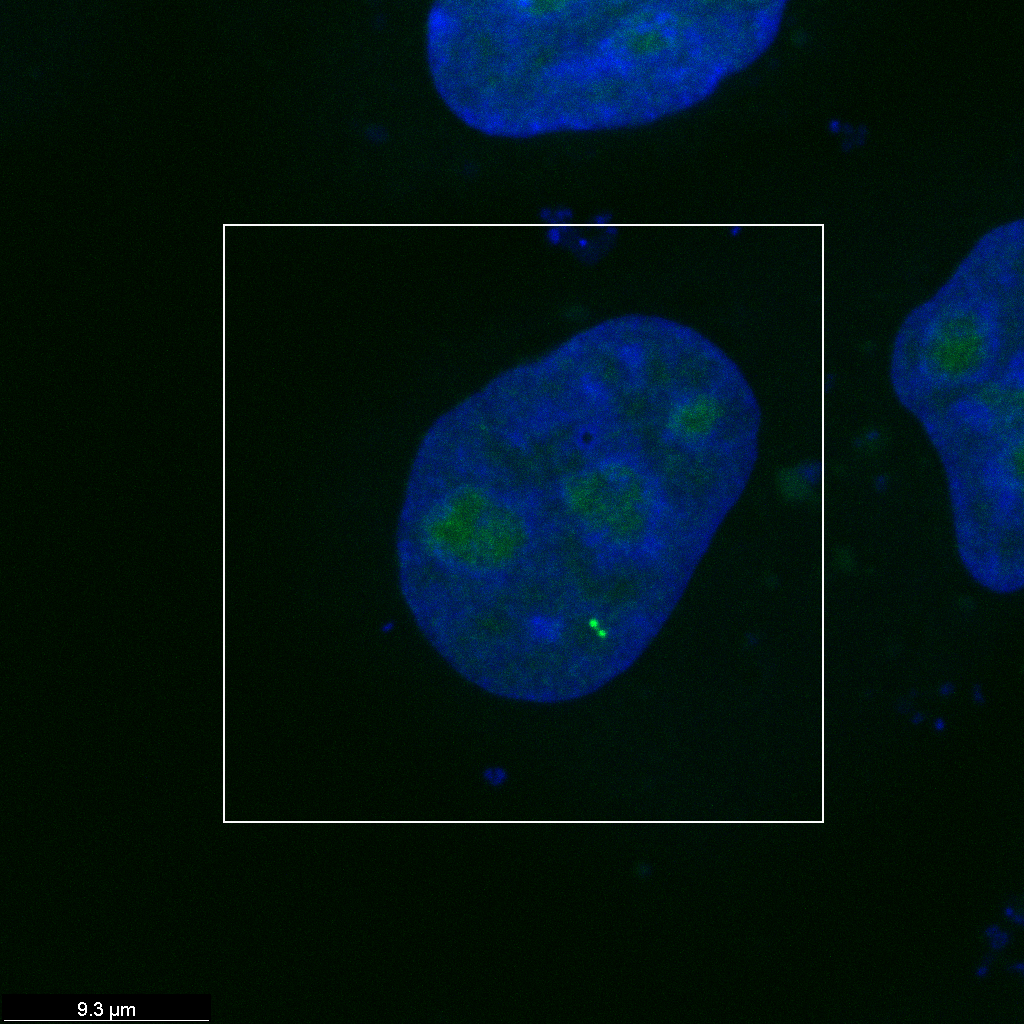

Supplement: Supplementary file 2 — Source data Fig. 1 [file 44318_2025_641_MOESM2_ESM.zip › EMBOJ-2025-120713R_SourceDataForFigure1/EMBOJ-2025-120713R_SourceDataForFigure1G/siSororin/merge.tif]

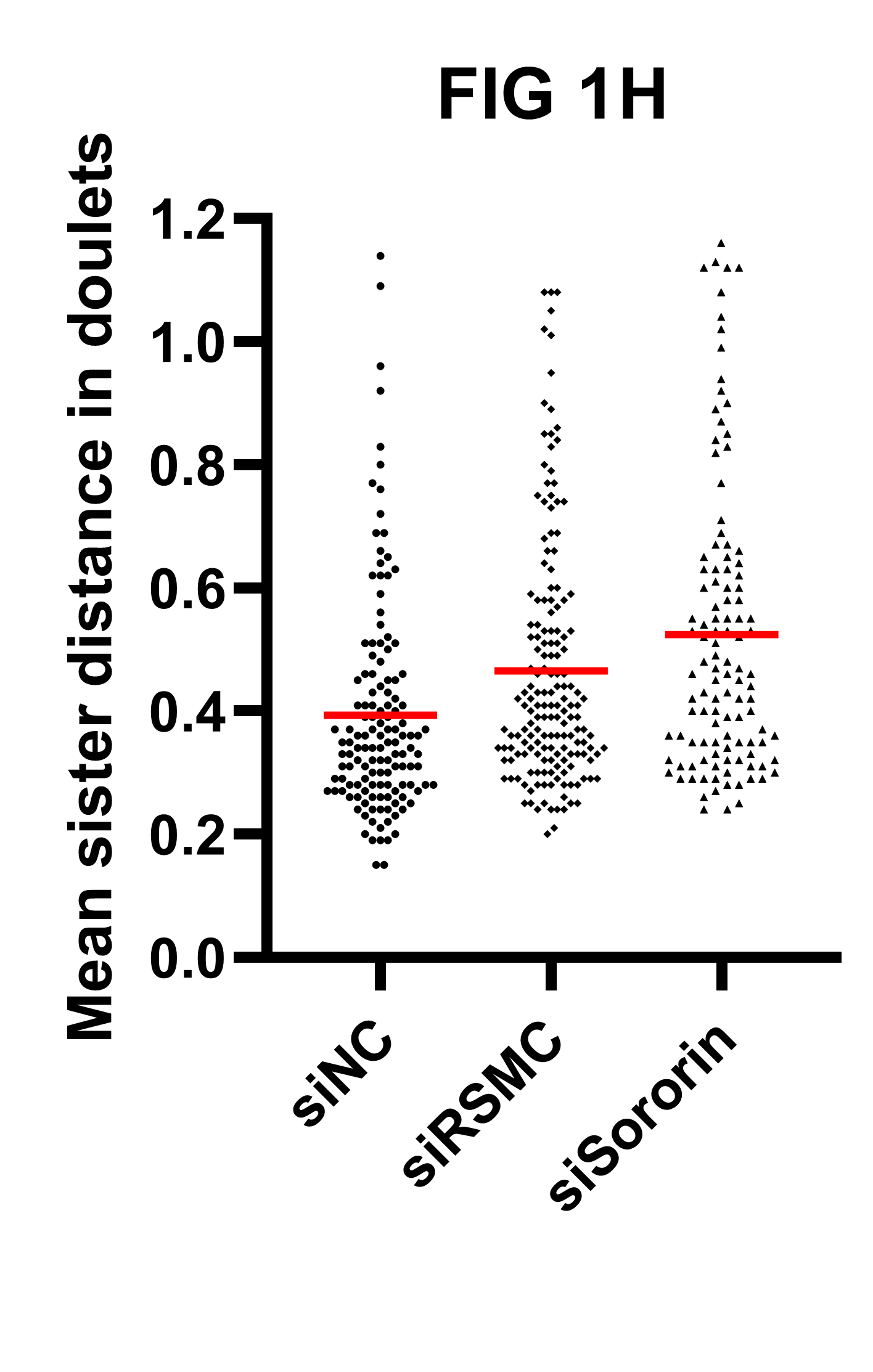

Supplement: Supplementary file 2 — Source data Fig. 1 [file 44318_2025_641_MOESM2_ESM.zip › EMBOJ-2025-120713R_SourceDataForFigure1/EMBOJ-2025-120713R_SourceDataForFigure1H/FIG 1H before PS.tif]

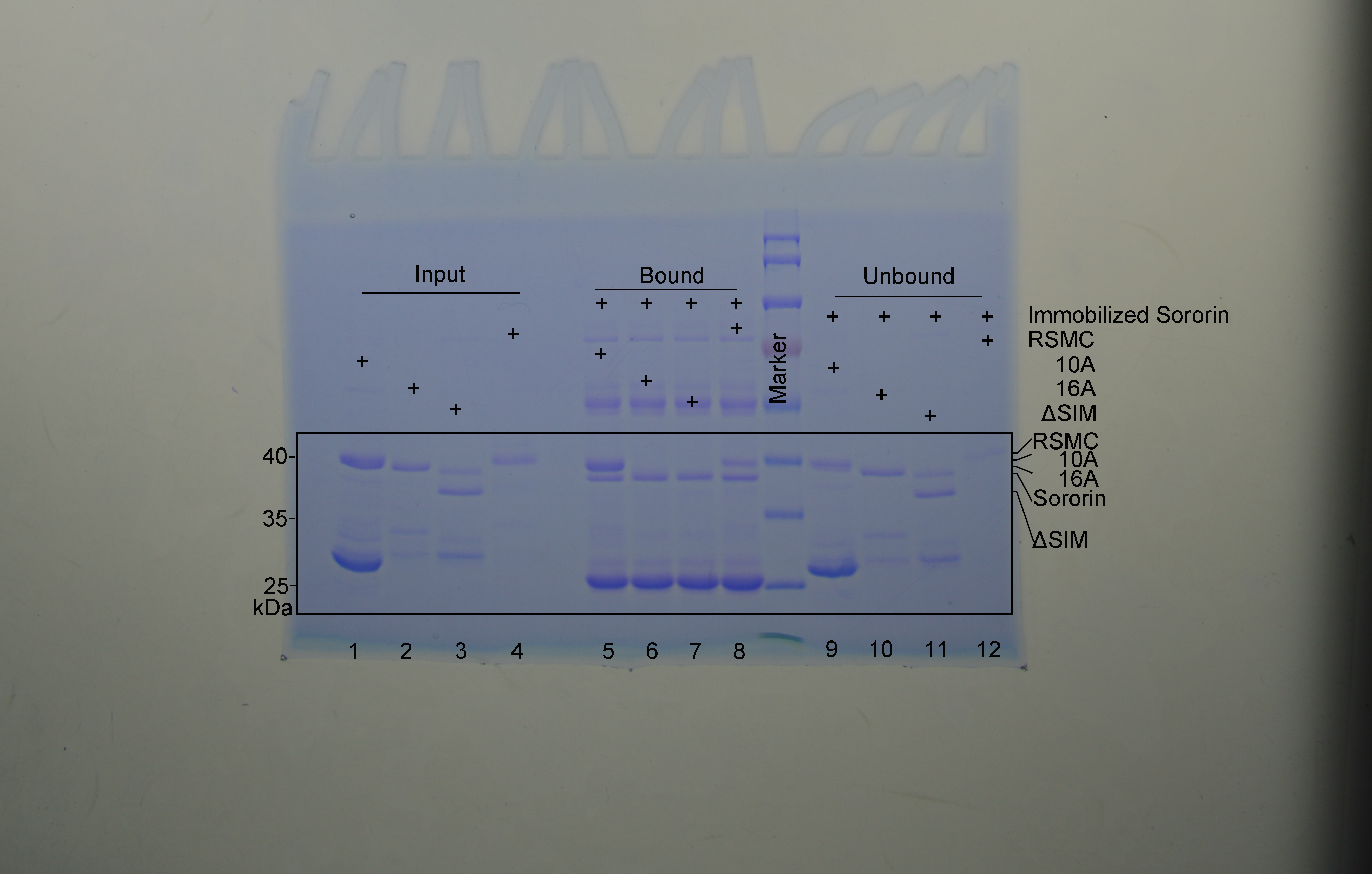

Supplement: Supplementary file 3 — Source data Fig. 2 [file 44318_2025_641_MOESM3_ESM.zip › EMBOJ-2025-120713R_SourceDataForFigure2/FIG 2B/2B-SourceData.tif]

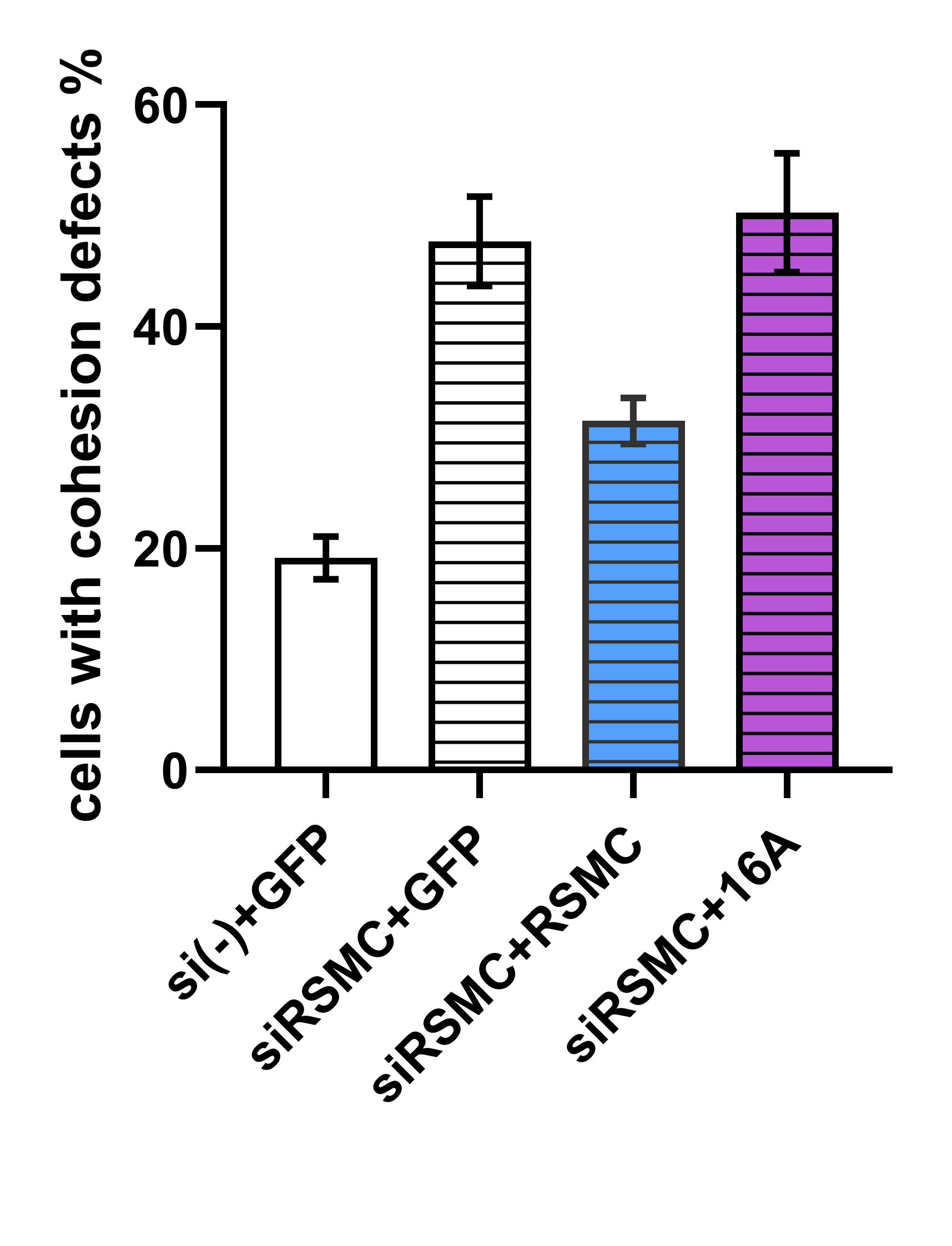

Supplement: Supplementary file 3 — Source data Fig. 2 [file 44318_2025_641_MOESM3_ESM.zip › EMBOJ-2025-120713R_SourceDataForFigure2/FIG 2C/FIG 2C before PS.tif]

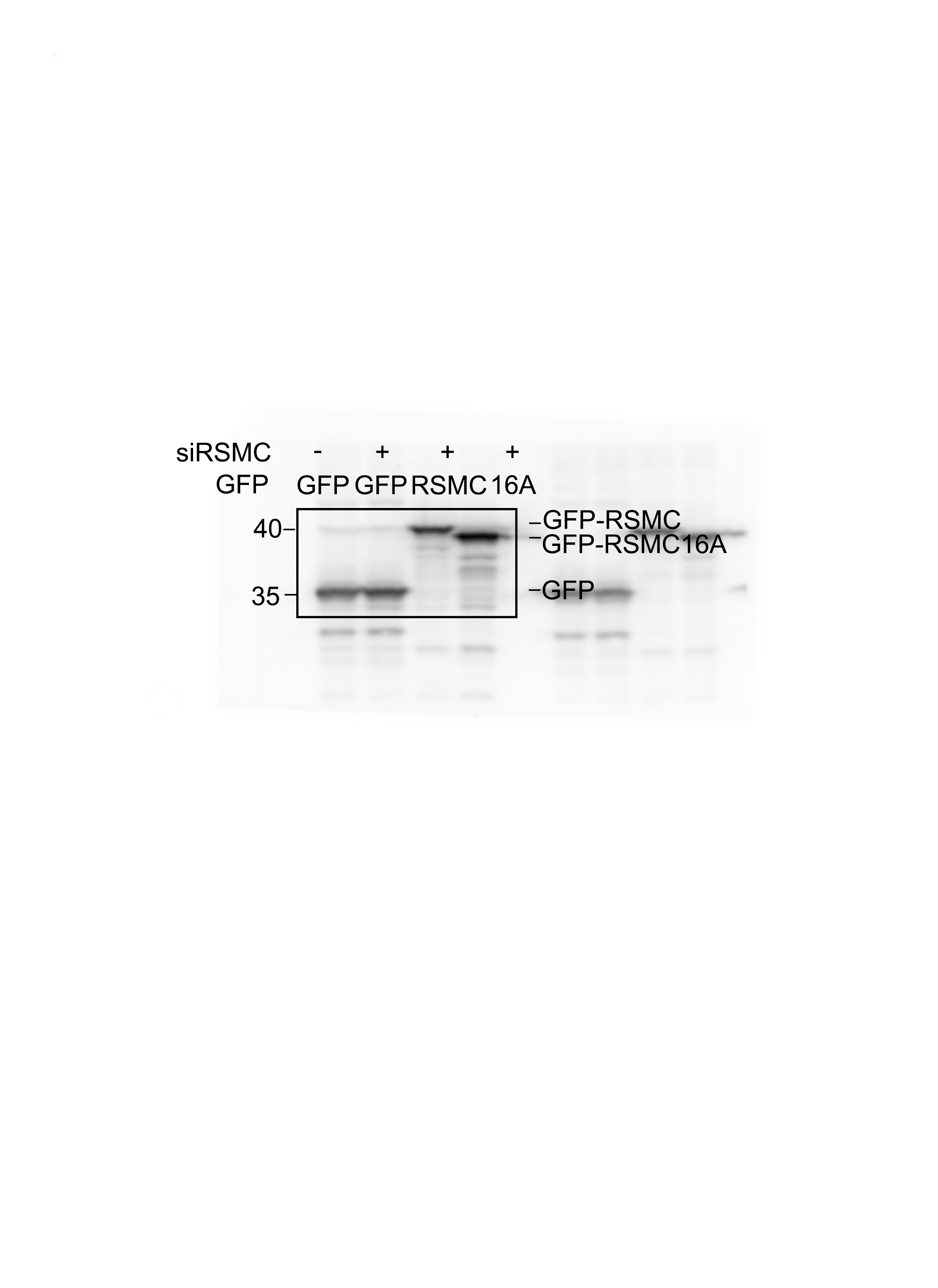

Supplement: Supplementary file 3 — Source data Fig. 2 [file 44318_2025_641_MOESM3_ESM.zip › EMBOJ-2025-120713R_SourceDataForFigure2/FIG 2C/GFP SourceData.tif]

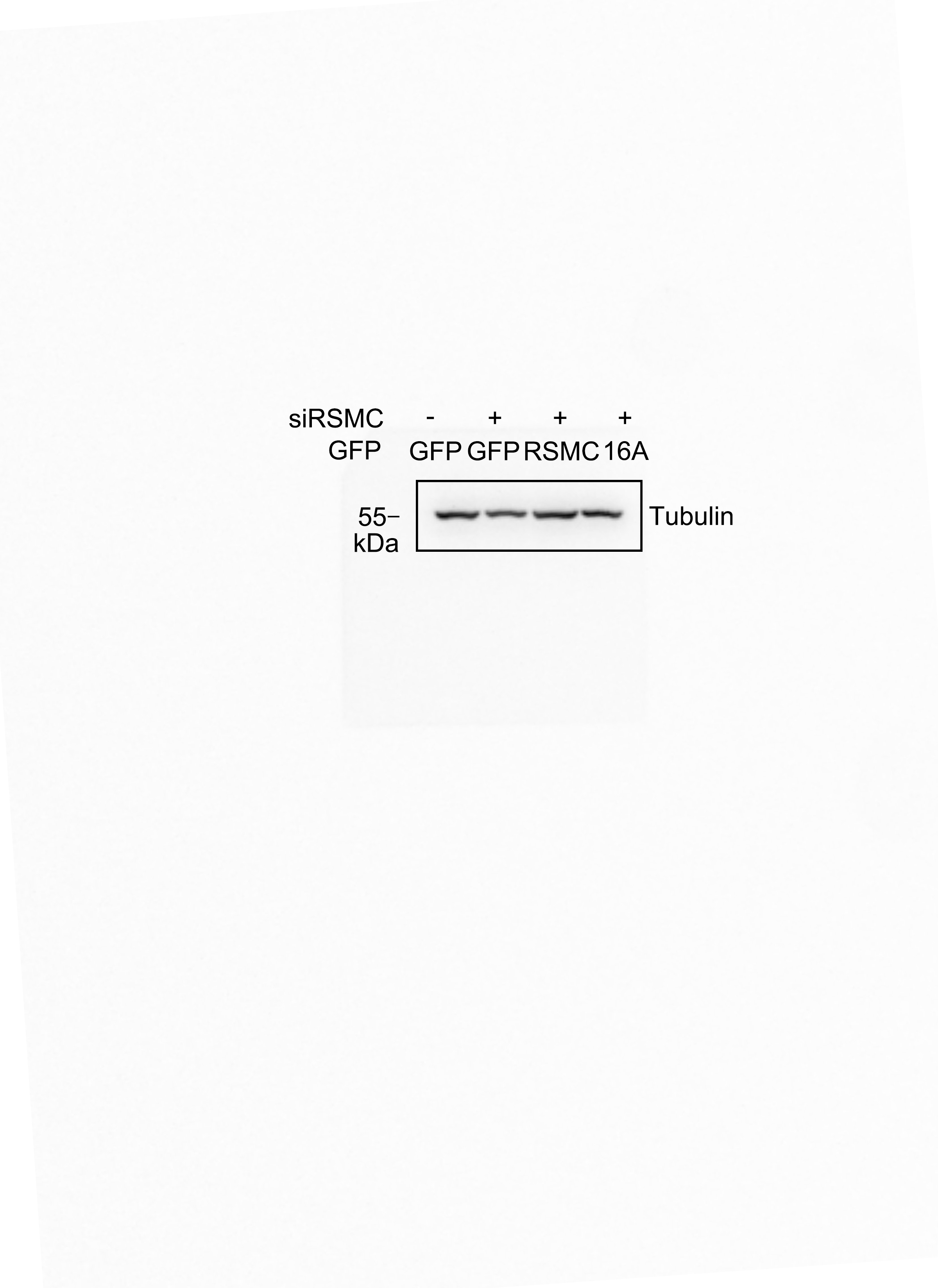

Supplement: Supplementary file 3 — Source data Fig. 2 [file 44318_2025_641_MOESM3_ESM.zip › EMBOJ-2025-120713R_SourceDataForFigure2/FIG 2C/Tubulin SourceData.tif]

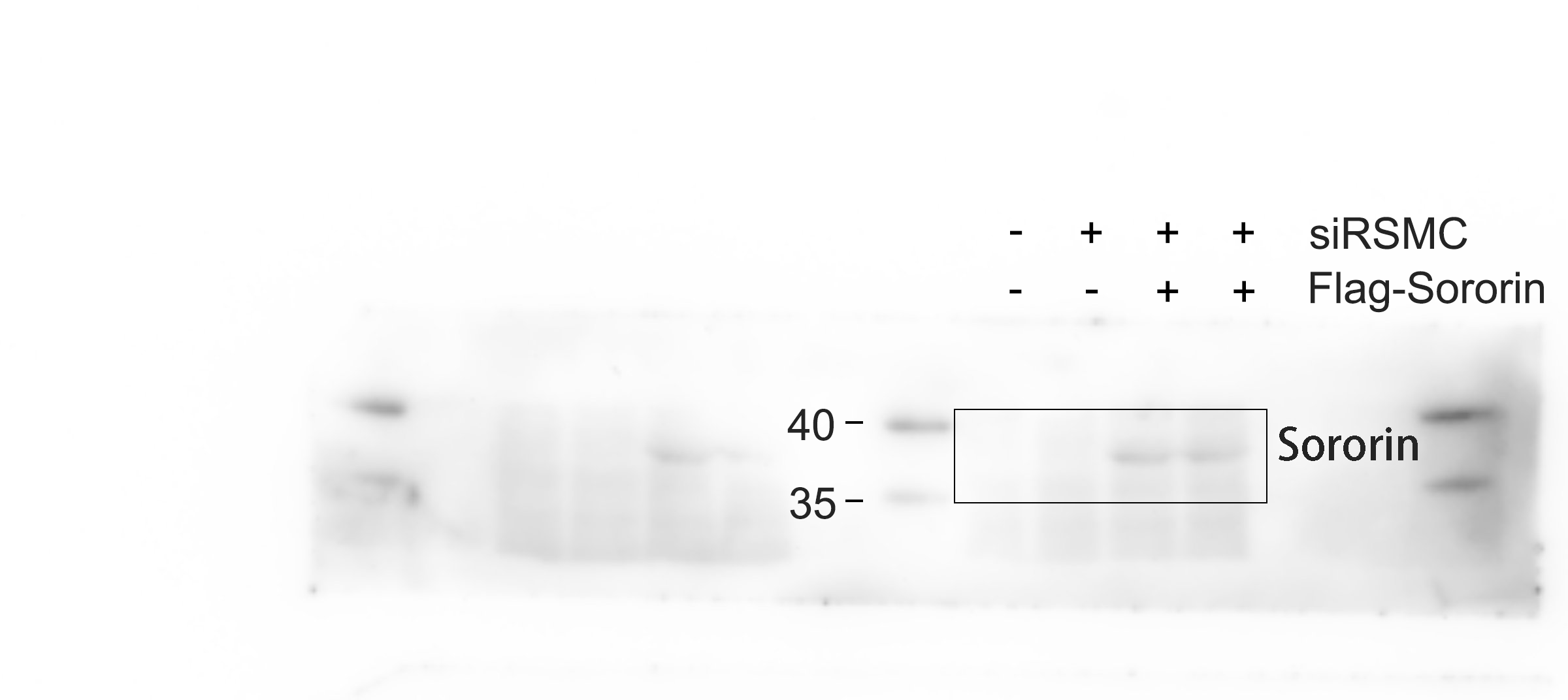

Supplement: Supplementary file 3 — Source data Fig. 2 [file 44318_2025_641_MOESM3_ESM.zip › EMBOJ-2025-120713R_SourceDataForFigure2/FIG 2D/FLAG-SORORIN SourceData.tif]

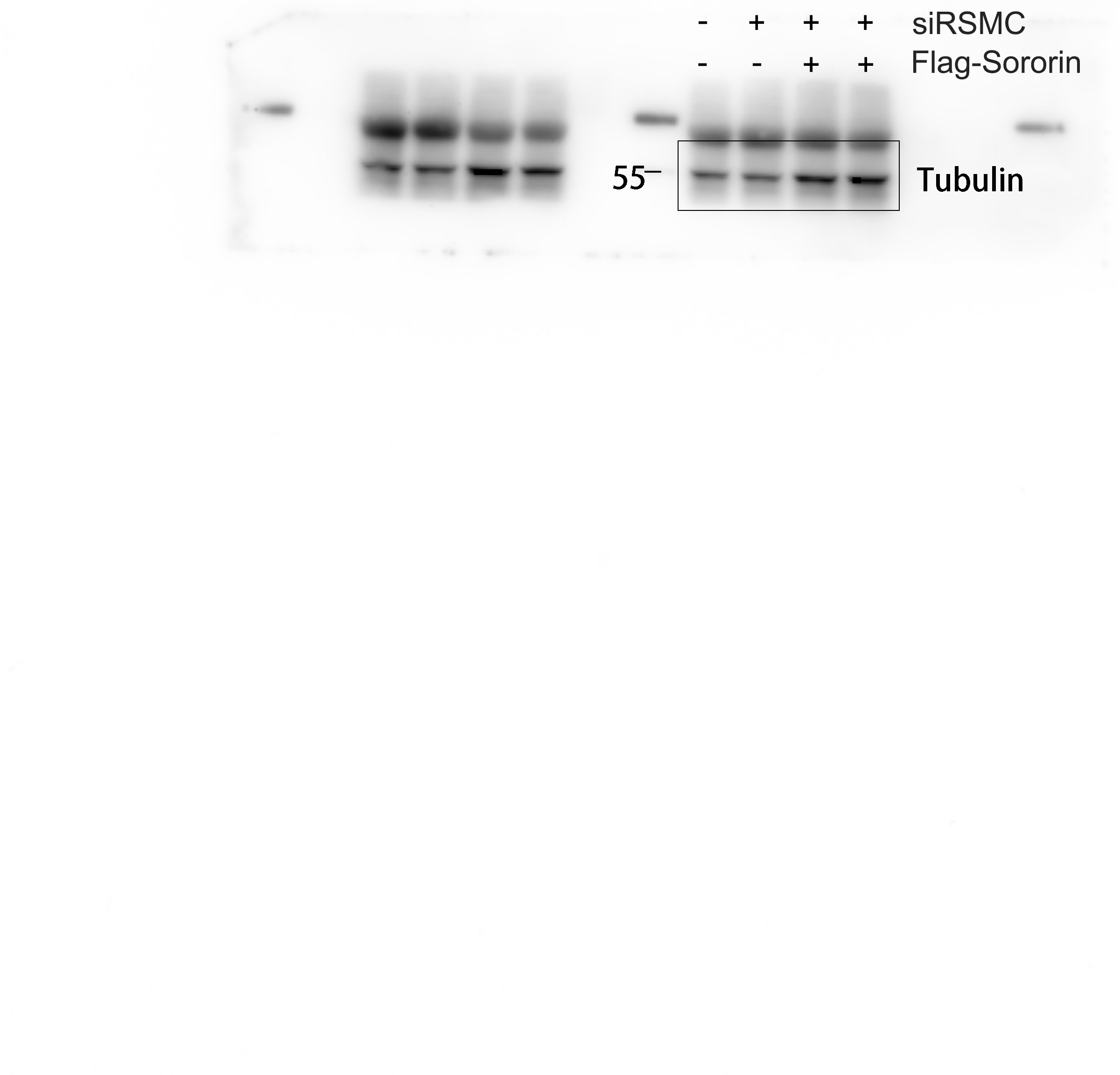

Supplement: Supplementary file 3 — Source data Fig. 2 [file 44318_2025_641_MOESM3_ESM.zip › EMBOJ-2025-120713R_SourceDataForFigure2/FIG 2D/TUBULIN Sourcedata.tif]

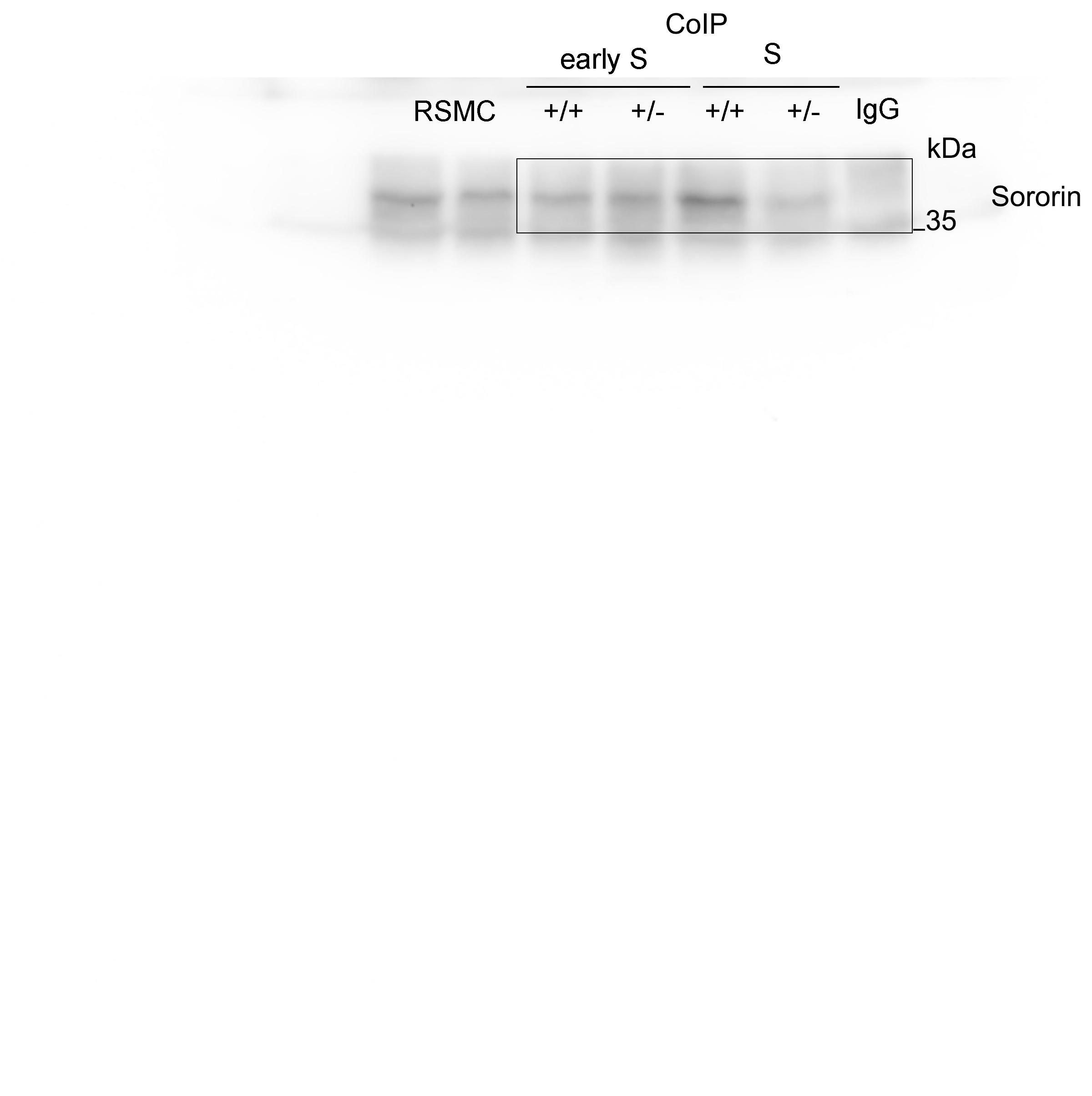

Supplement: Supplementary file 4 — Source data Fig. 3 [file 44318_2025_641_MOESM4_ESM.zip › EMBOJ-2025-120713R_SourceDataForFigure3/FIG 3A/1. CoIP Sororin SourceData.tif]

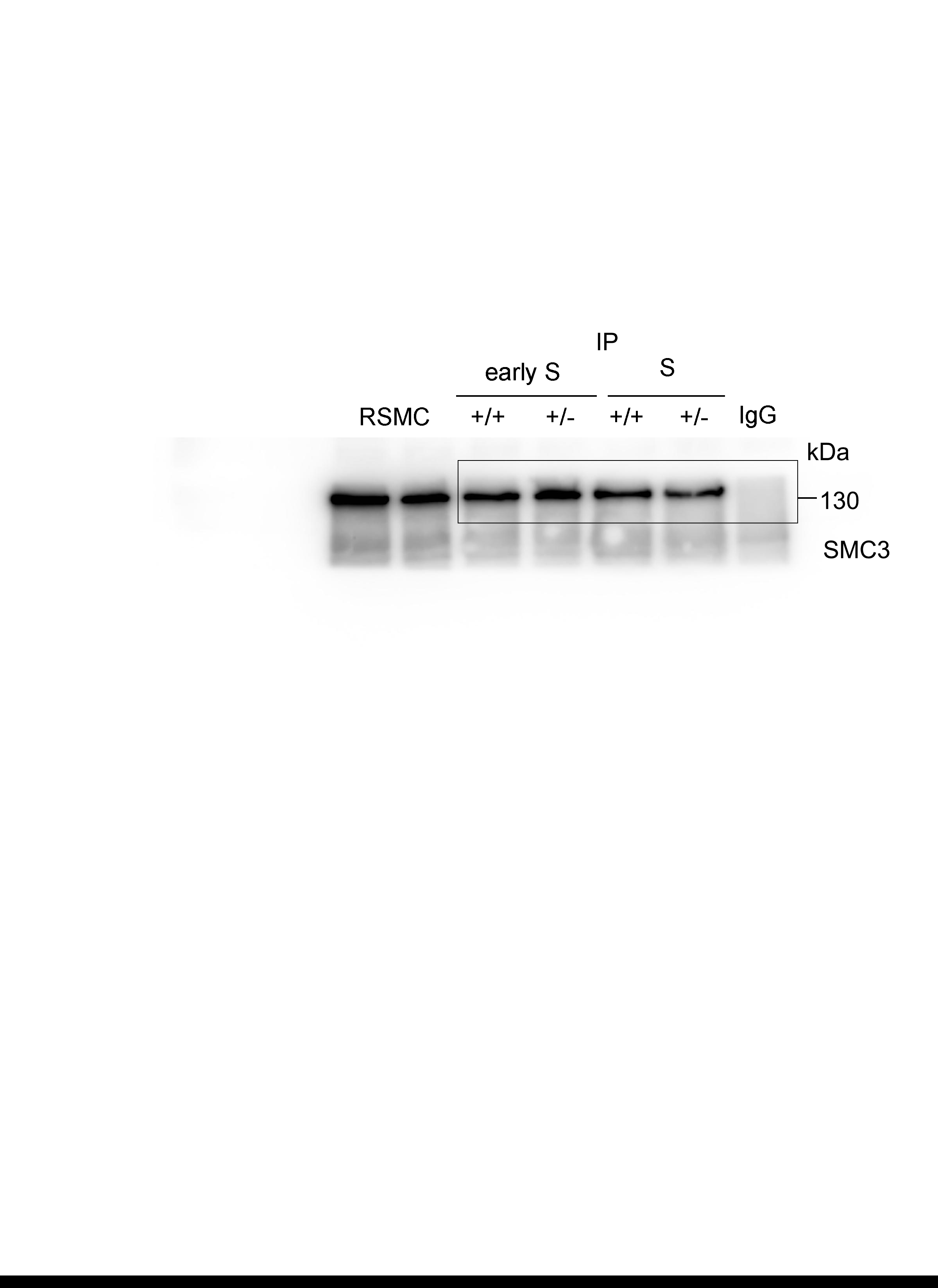

Supplement: Supplementary file 4 — Source data Fig. 3 [file 44318_2025_641_MOESM4_ESM.zip › EMBOJ-2025-120713R_SourceDataForFigure3/FIG 3A/2. IP SMC3 SourceData.tif]

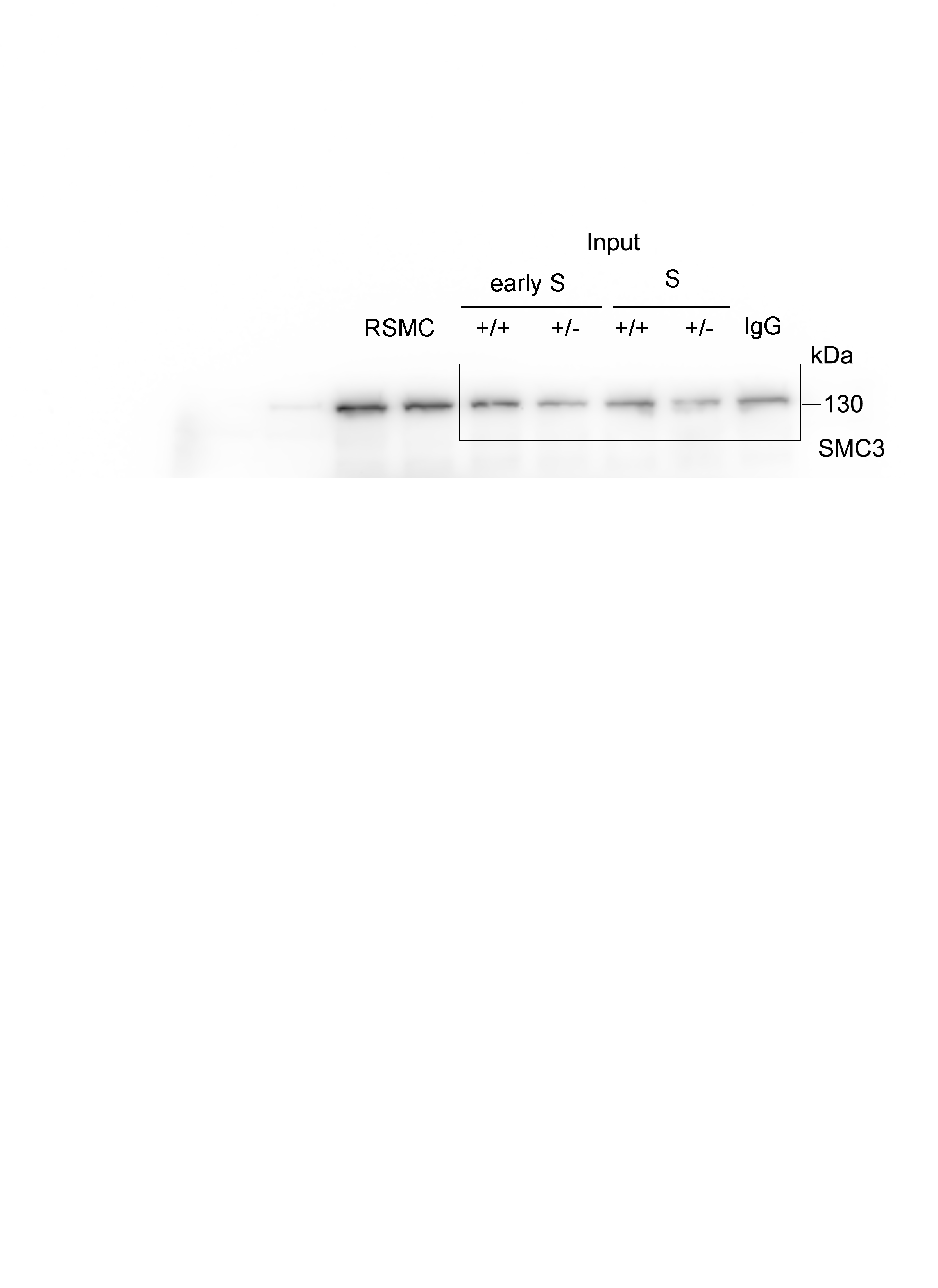

Supplement: Supplementary file 4 — Source data Fig. 3 [file 44318_2025_641_MOESM4_ESM.zip › EMBOJ-2025-120713R_SourceDataForFigure3/FIG 3A/3. Input SMC3 input SourceData.tif]

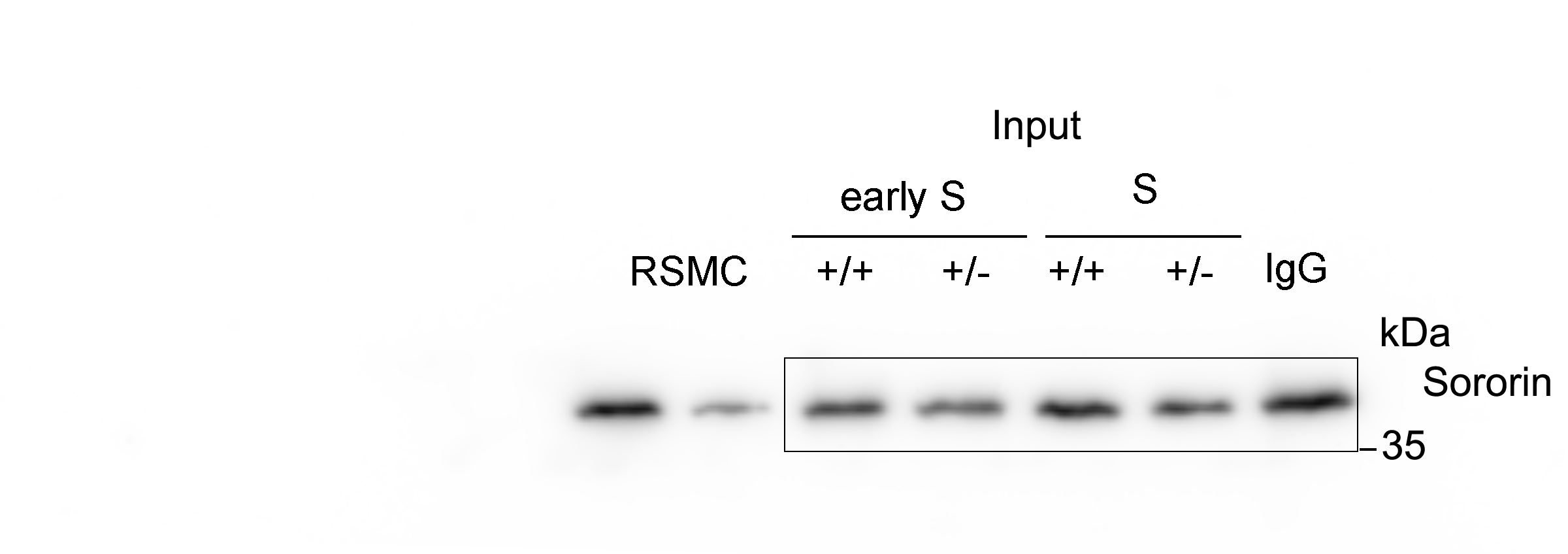

Supplement: Supplementary file 4 — Source data Fig. 3 [file 44318_2025_641_MOESM4_ESM.zip › EMBOJ-2025-120713R_SourceDataForFigure3/FIG 3A/4. Input Sororin SourceData.tif]

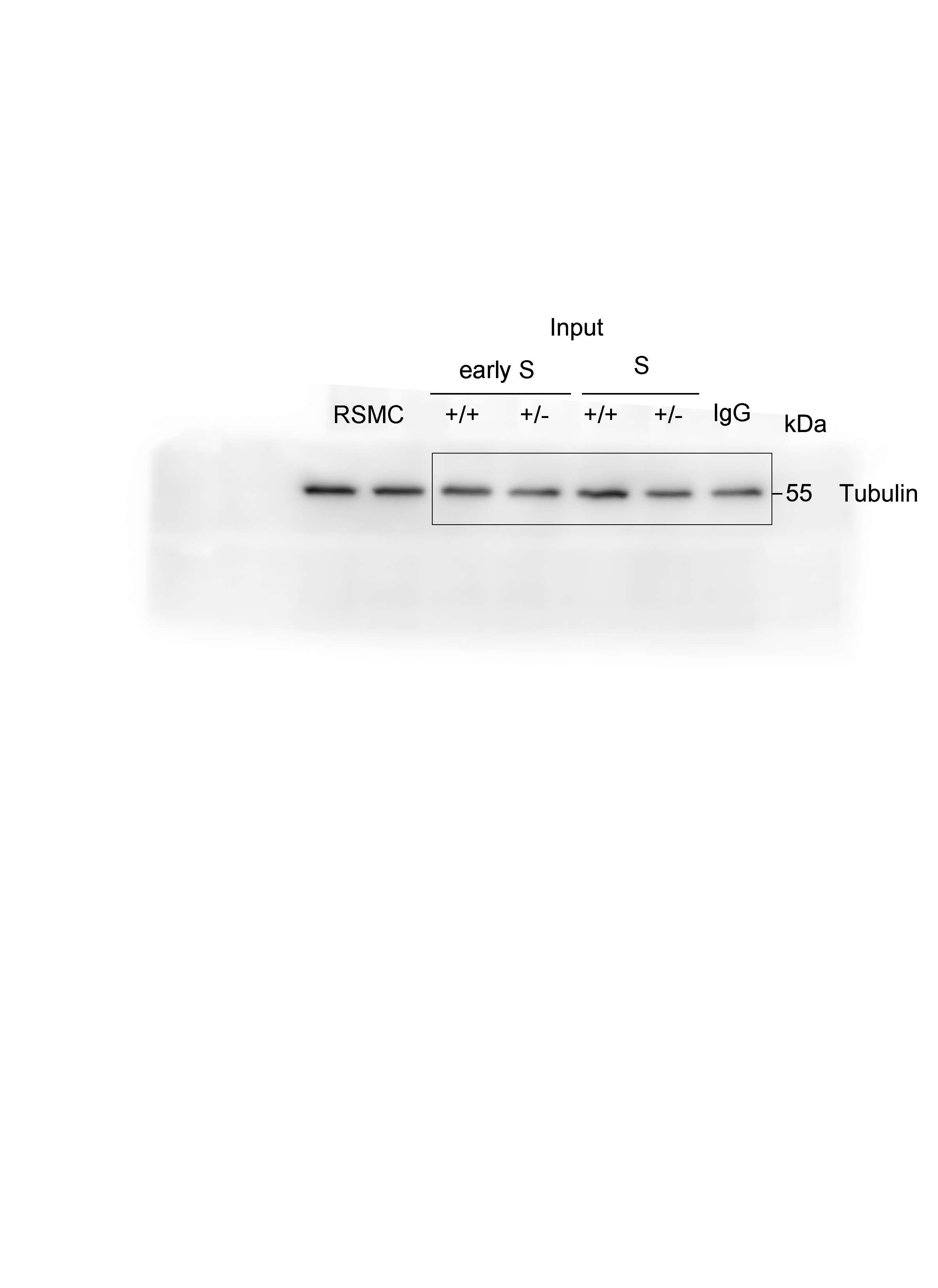

Supplement: Supplementary file 4 — Source data Fig. 3 [file 44318_2025_641_MOESM4_ESM.zip › EMBOJ-2025-120713R_SourceDataForFigure3/FIG 3A/5. Input Tubulin SourceData.tif]

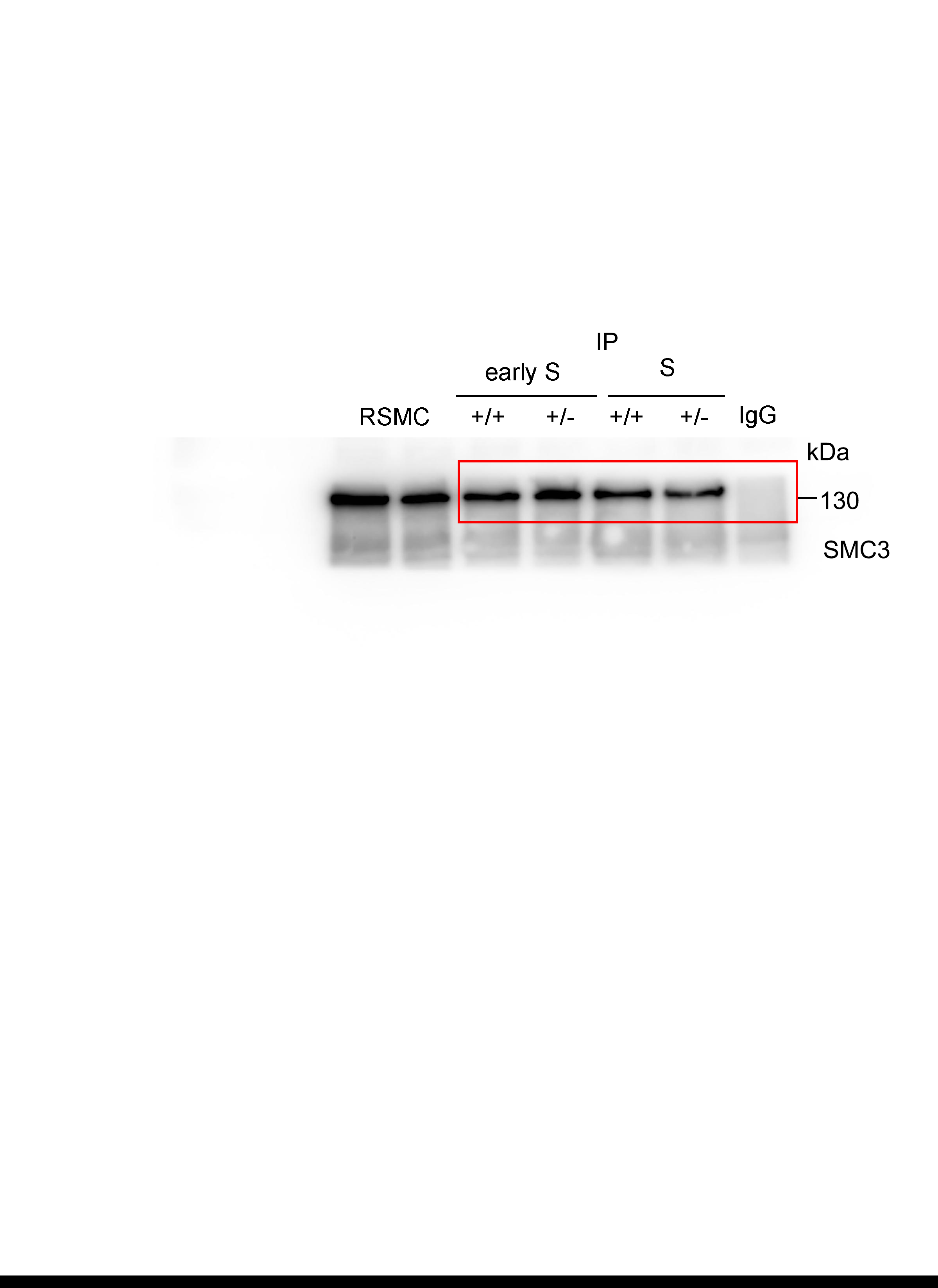

Supplement: Supplementary file 4 — Source data Fig. 3 [file 44318_2025_641_MOESM4_ESM.zip › EMBOJ-2025-120713R_SourceDataForFigure3/FIG 3B/EXP1/SMC3.tif]

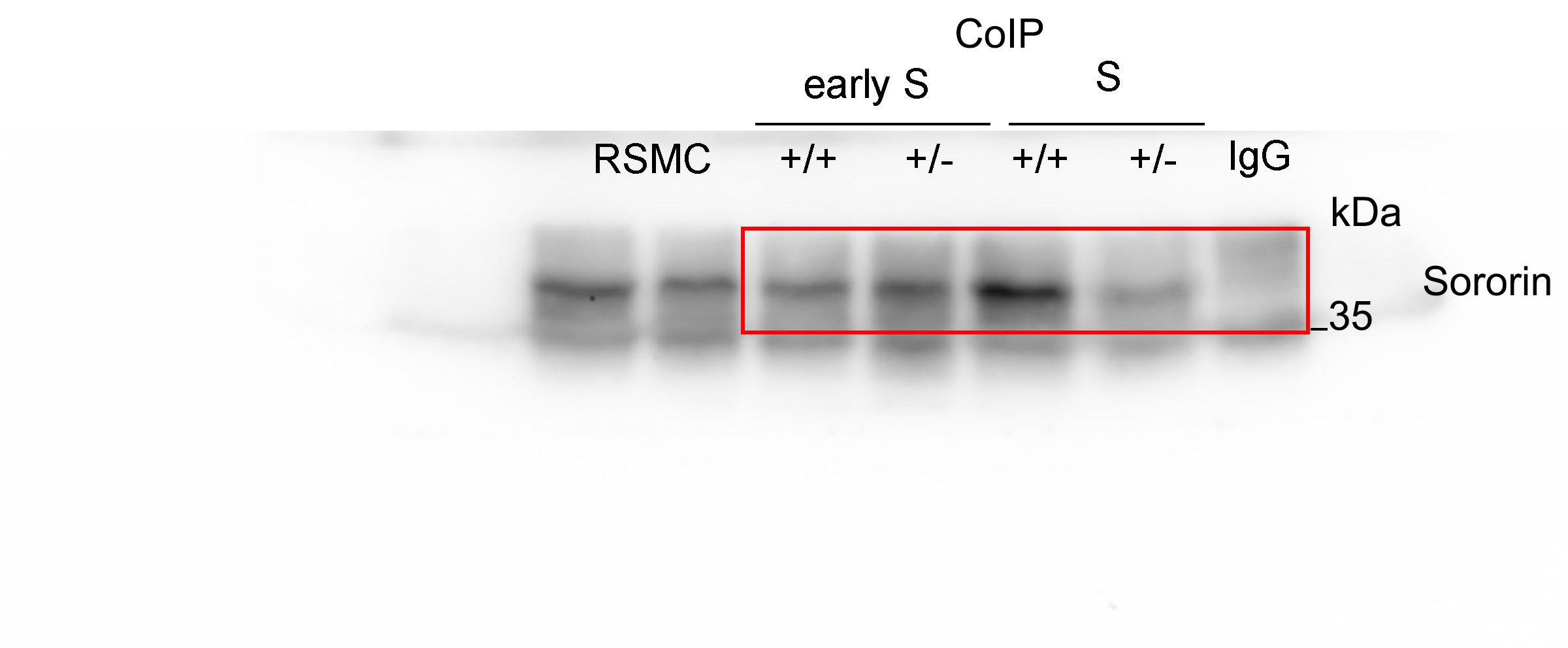

Supplement: Supplementary file 4 — Source data Fig. 3 [file 44318_2025_641_MOESM4_ESM.zip › EMBOJ-2025-120713R_SourceDataForFigure3/FIG 3B/EXP1/Sororin.tif]

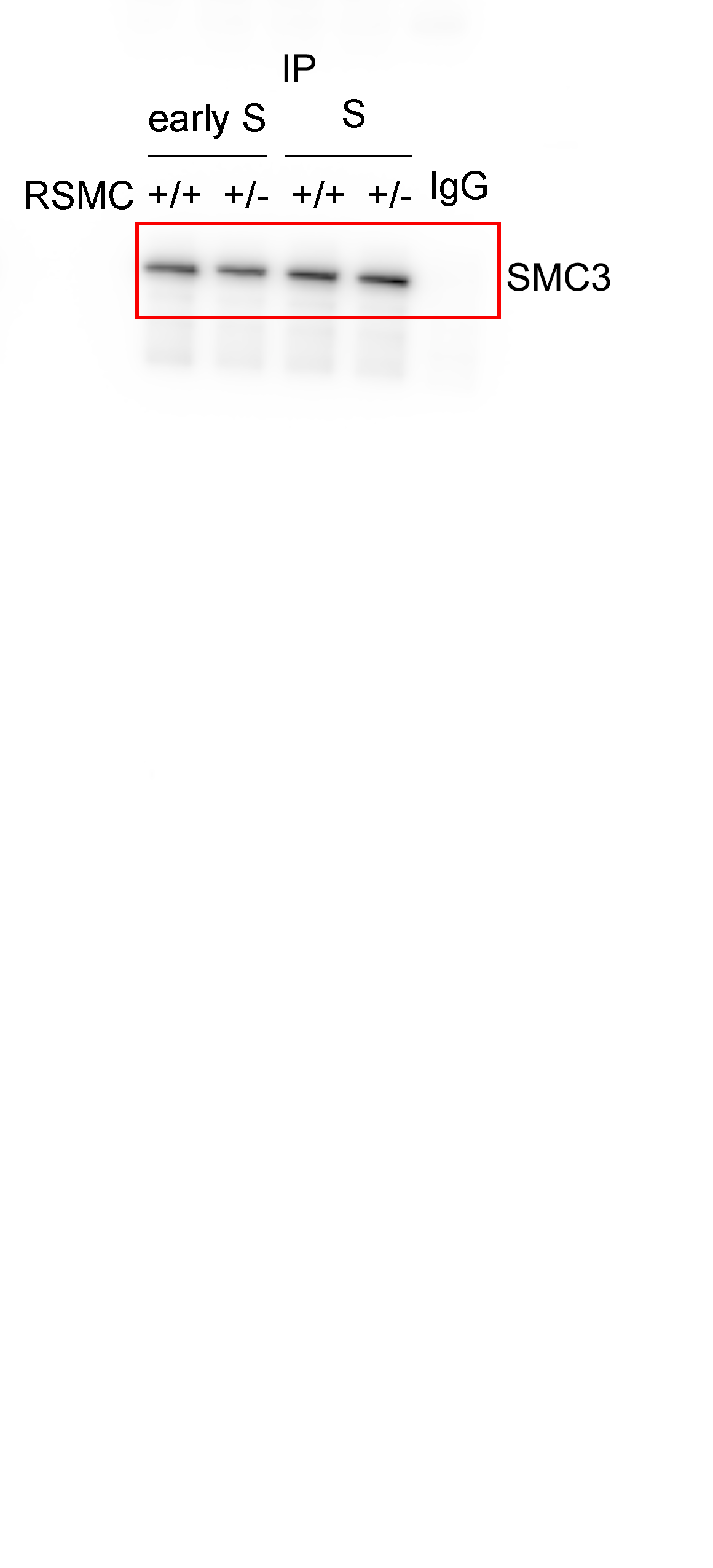

Supplement: Supplementary file 4 — Source data Fig. 3 [file 44318_2025_641_MOESM4_ESM.zip › EMBOJ-2025-120713R_SourceDataForFigure3/FIG 3B/EXP2/SMC3.tif]

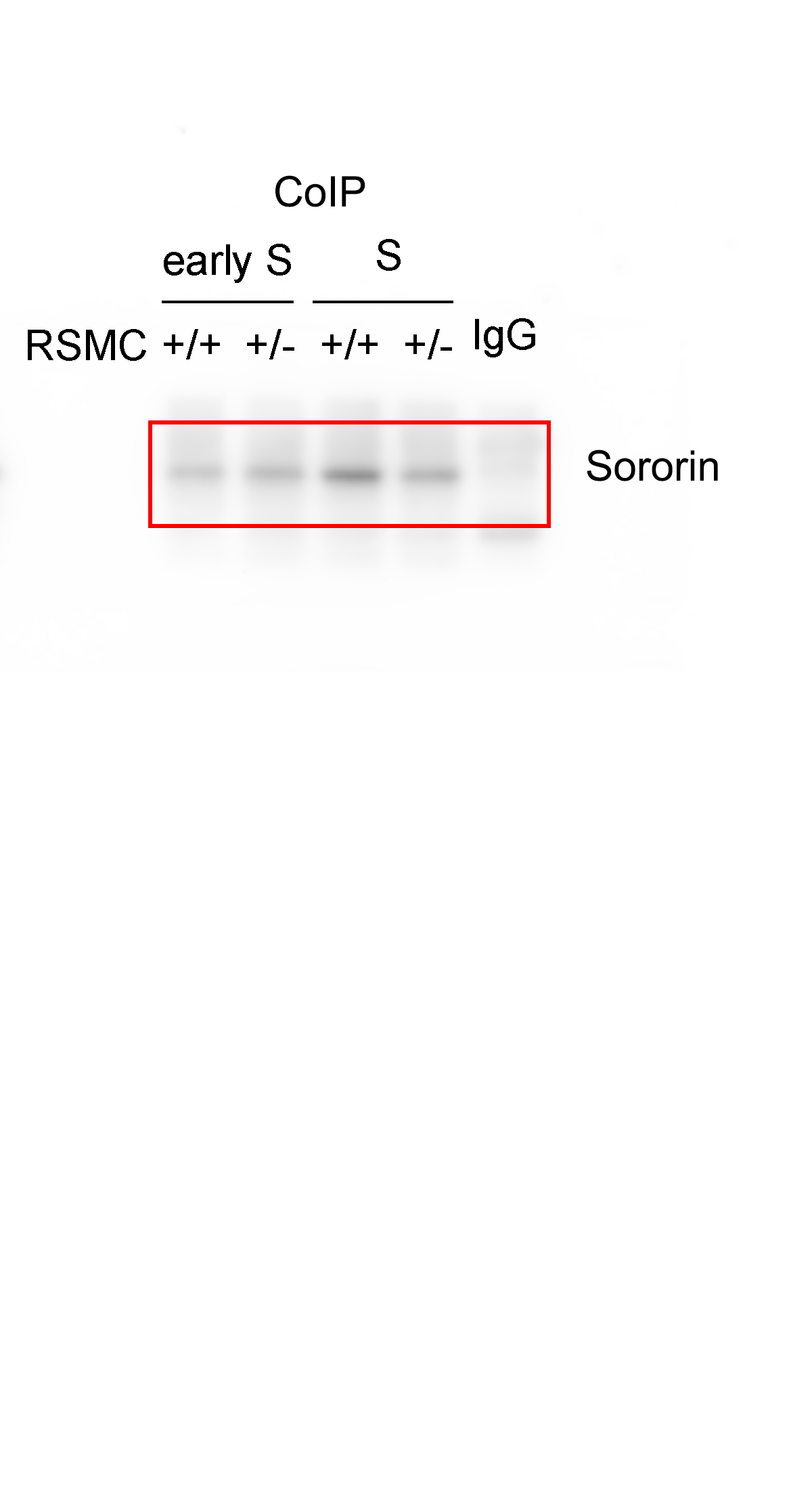

Supplement: Supplementary file 4 — Source data Fig. 3 [file 44318_2025_641_MOESM4_ESM.zip › EMBOJ-2025-120713R_SourceDataForFigure3/FIG 3B/EXP2/Sororin.tif]

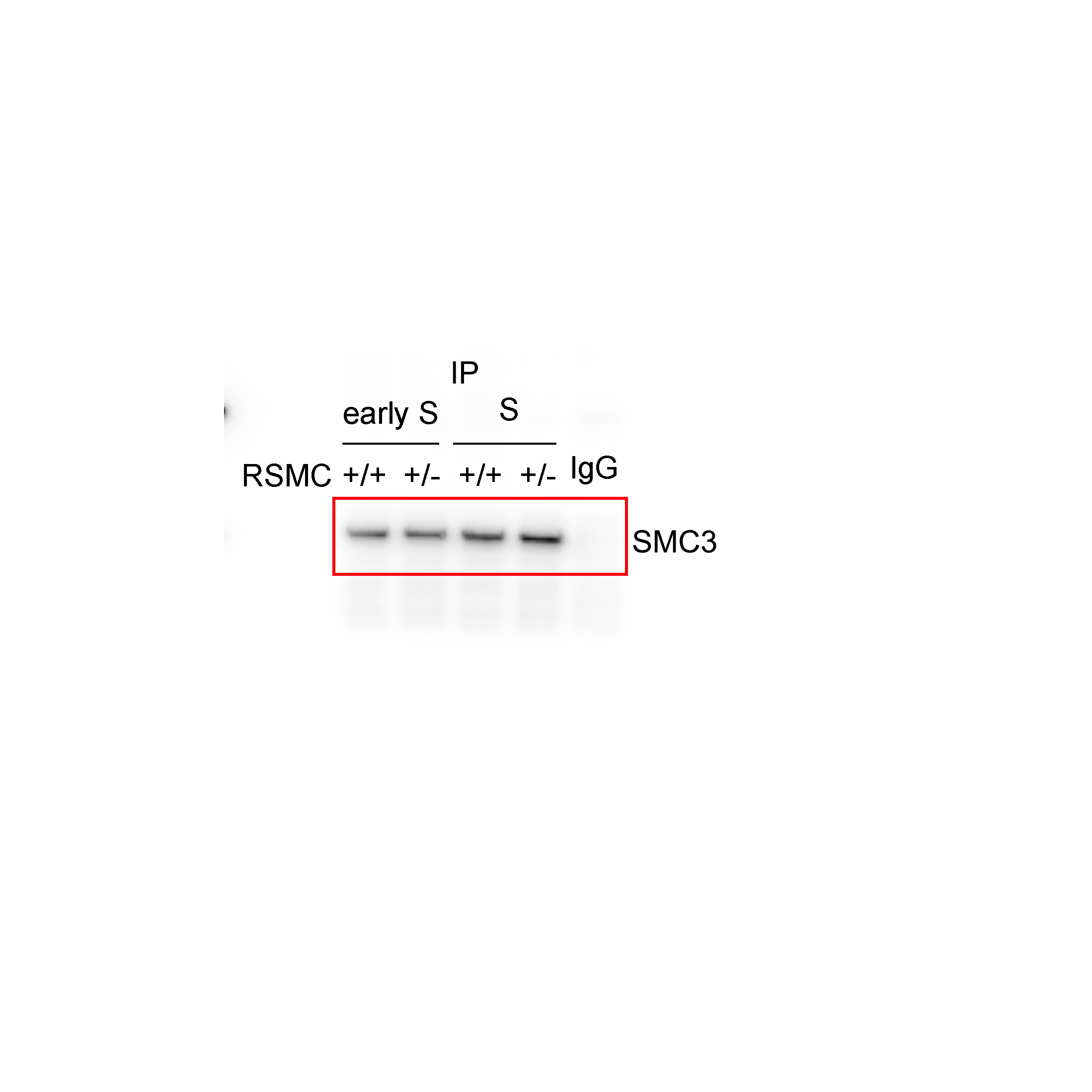

Supplement: Supplementary file 4 — Source data Fig. 3 [file 44318_2025_641_MOESM4_ESM.zip › EMBOJ-2025-120713R_SourceDataForFigure3/FIG 3B/EXP3/SMC3.tif]

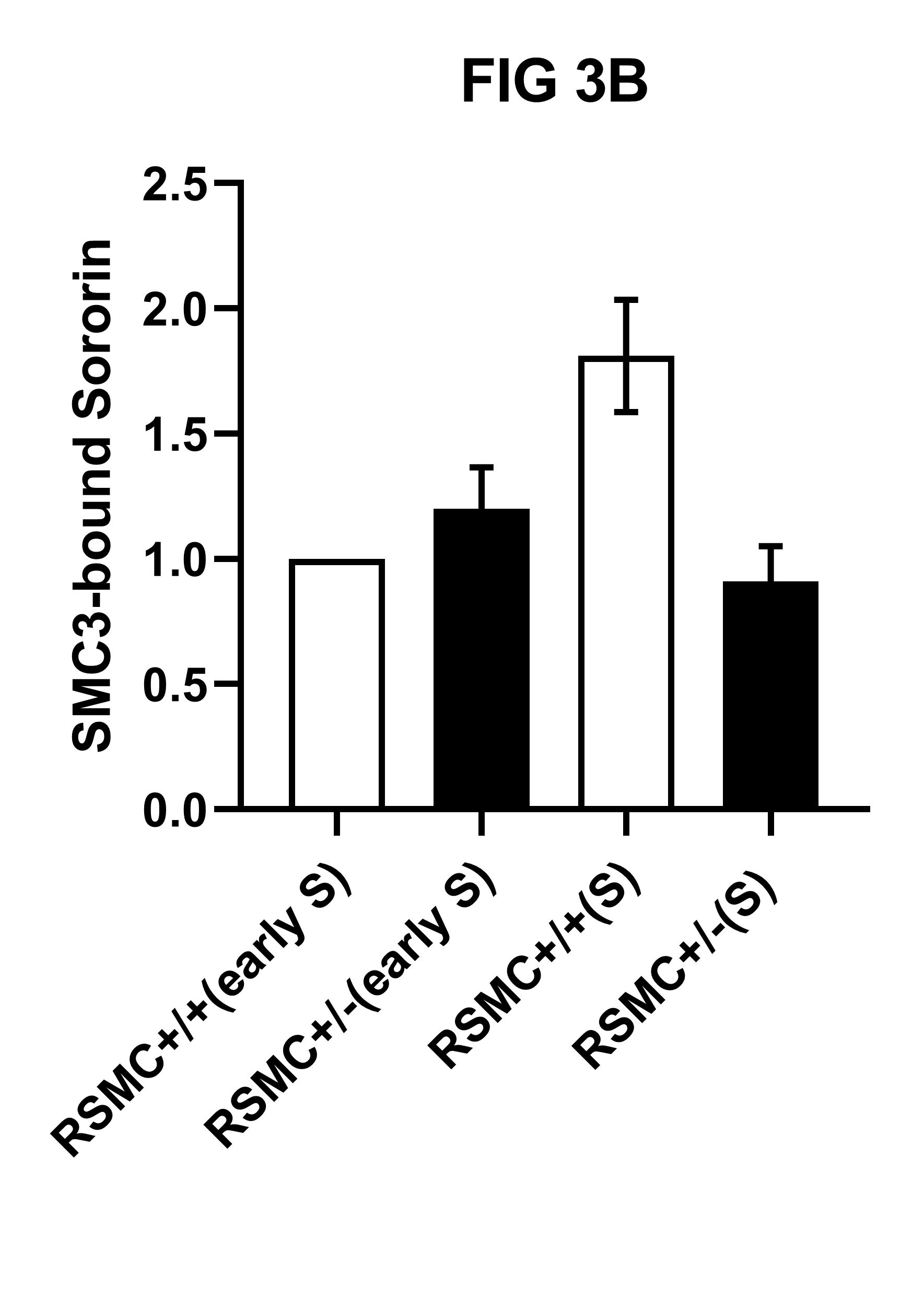

Supplement: Supplementary file 4 — Source data Fig. 3 [file 44318_2025_641_MOESM4_ESM.zip › EMBOJ-2025-120713R_SourceDataForFigure3/FIG 3B/FIG 3B before PS.tif]

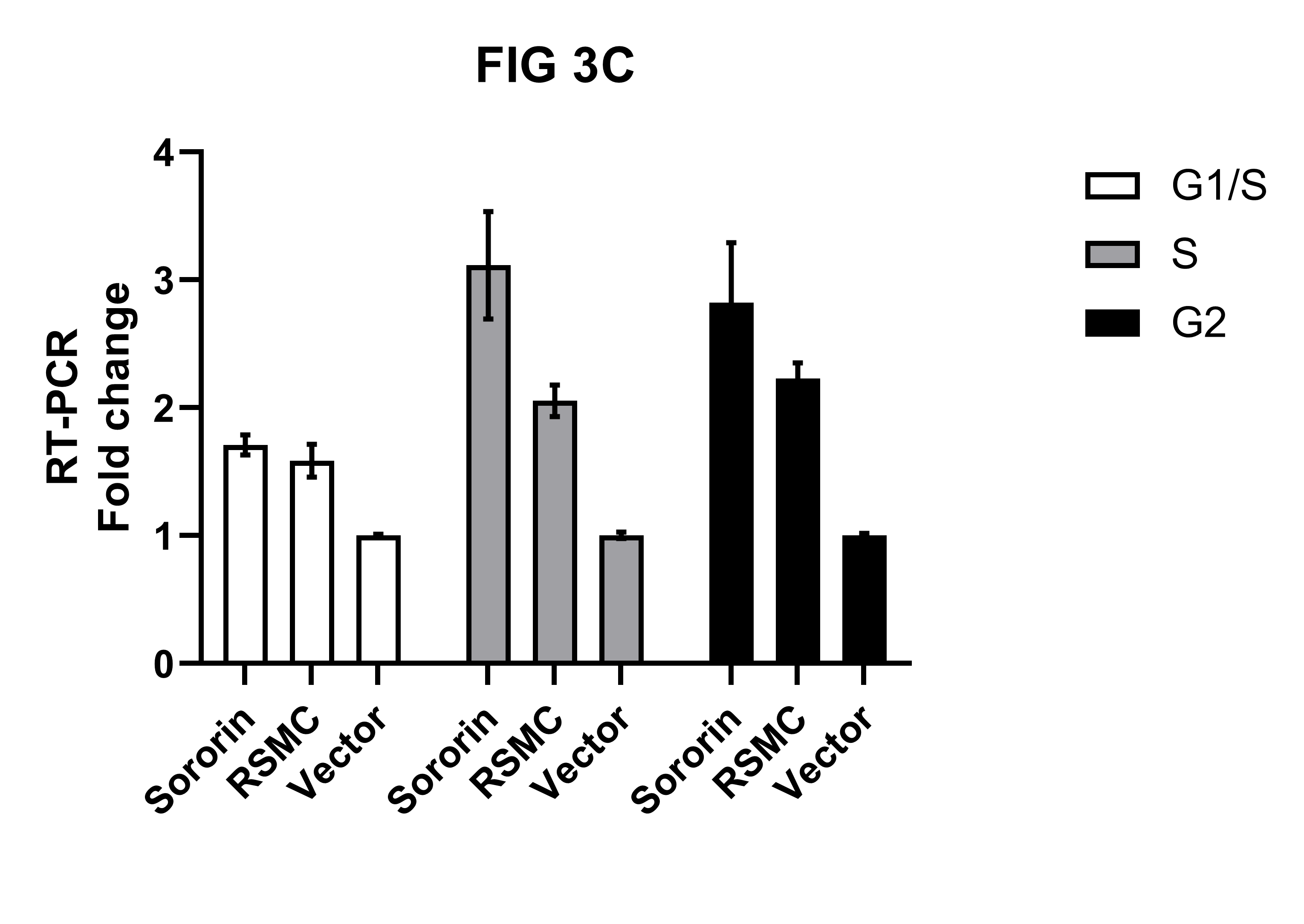

Supplement: Supplementary file 4 — Source data Fig. 3 [file 44318_2025_641_MOESM4_ESM.zip › EMBOJ-2025-120713R_SourceDataForFigure3/FIG 3C/FIG 3C before PS.tif]

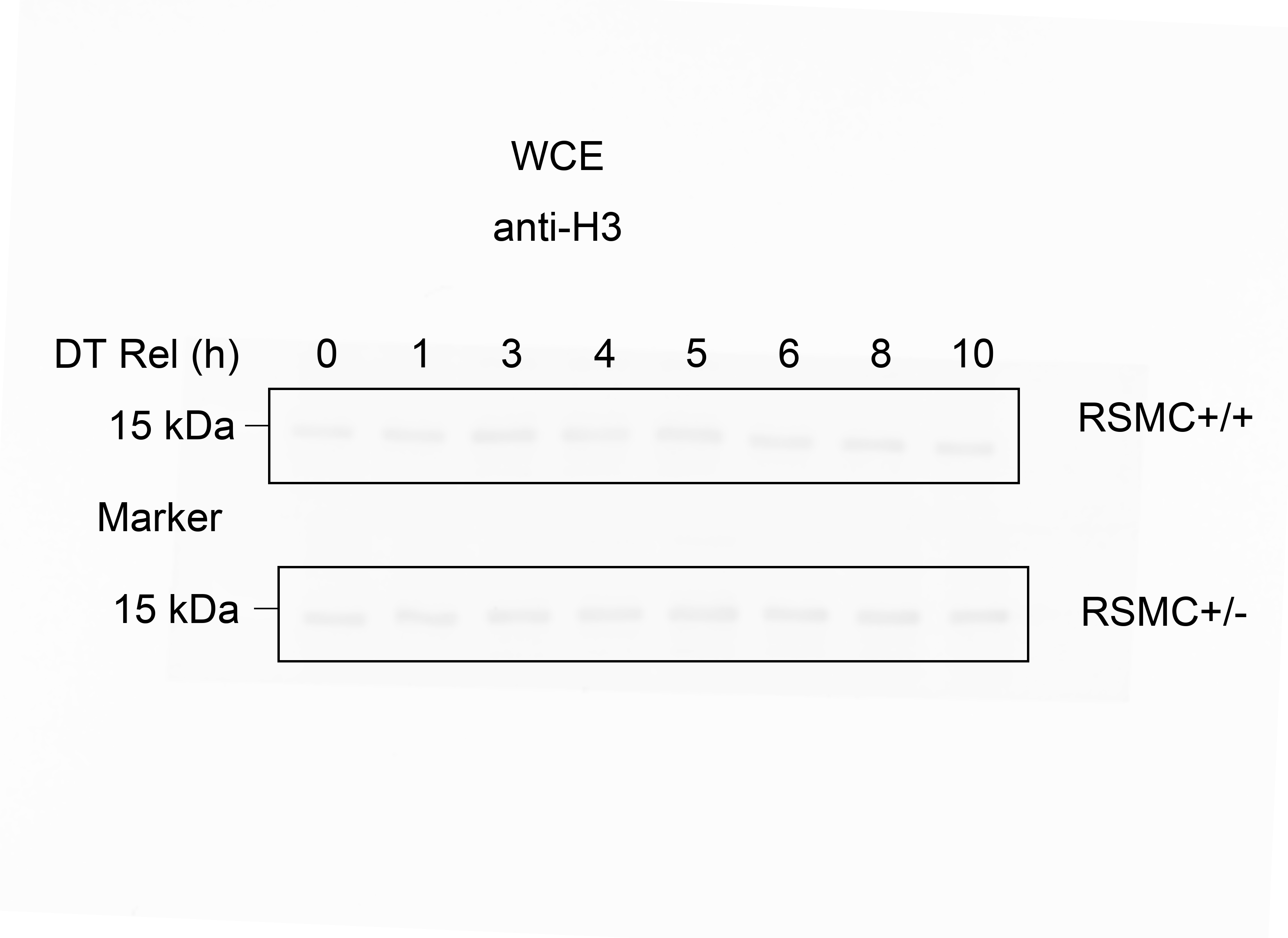

Supplement: Supplementary file 4 — Source data Fig. 3 [file 44318_2025_641_MOESM4_ESM.zip › EMBOJ-2025-120713R_SourceDataForFigure3/FIG 3D/H3 RAW data.tif]

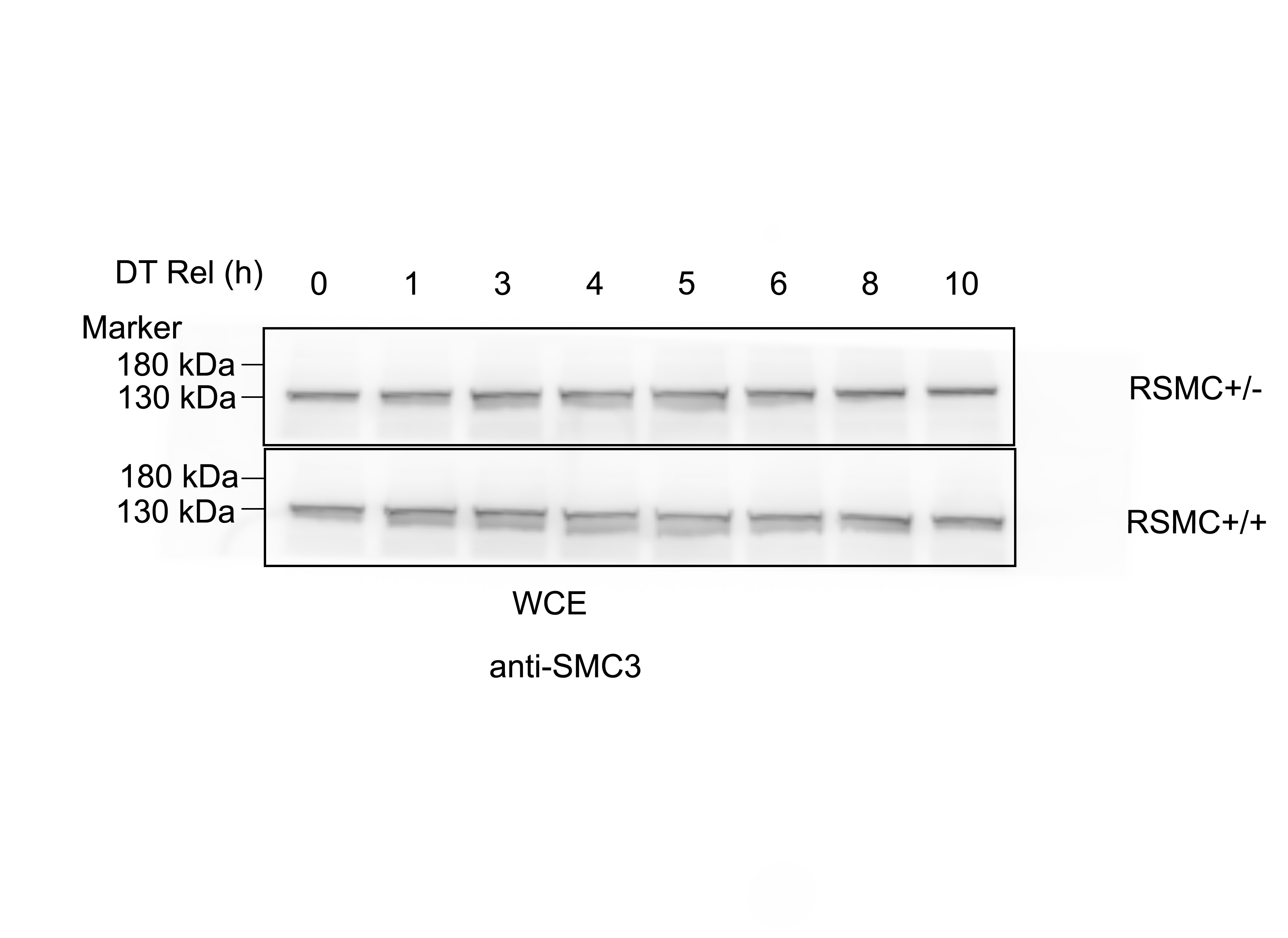

Supplement: Supplementary file 4 — Source data Fig. 3 [file 44318_2025_641_MOESM4_ESM.zip › EMBOJ-2025-120713R_SourceDataForFigure3/FIG 3D/SMC3 RAW data.tif]

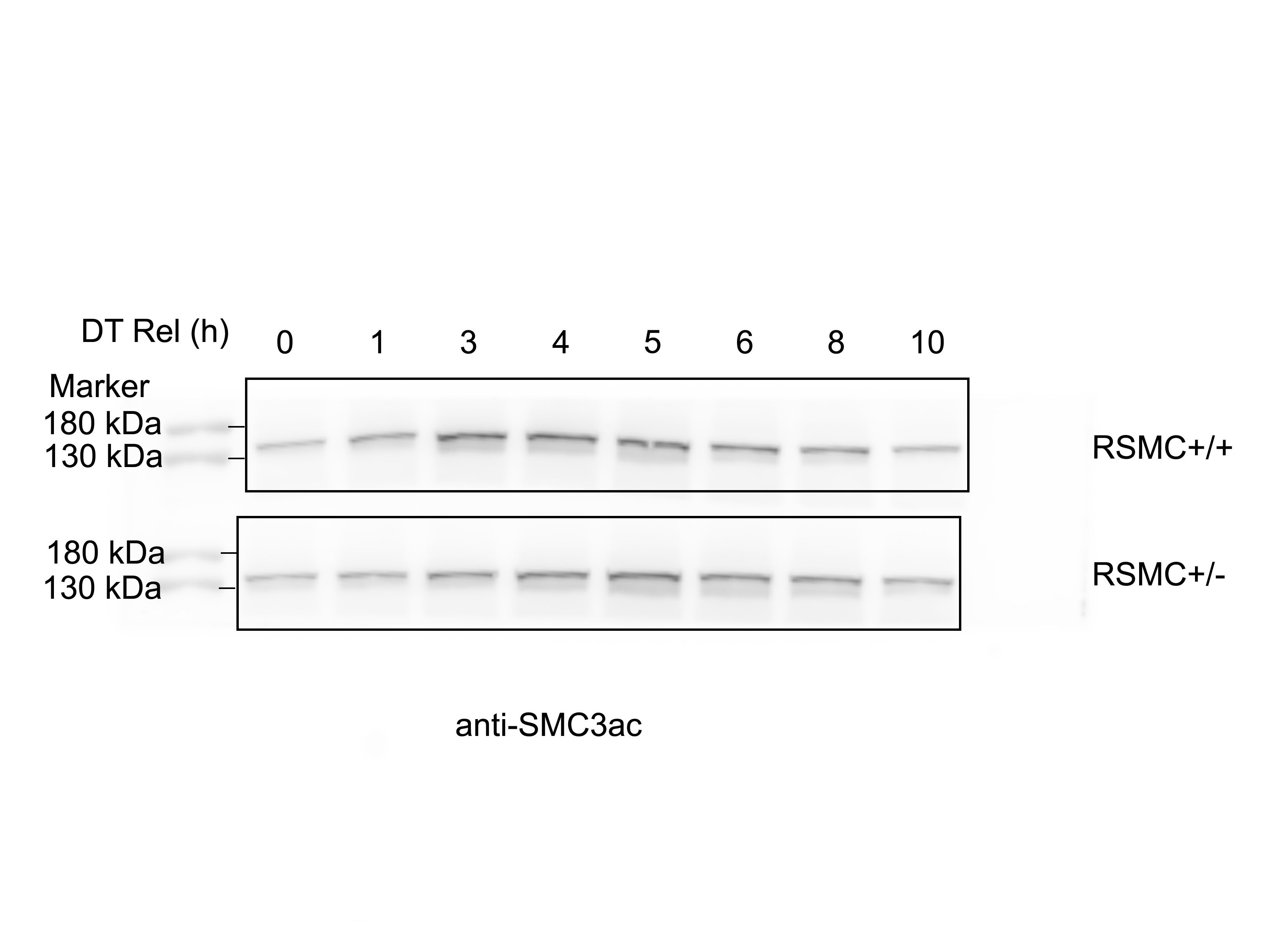

Supplement: Supplementary file 4 — Source data Fig. 3 [file 44318_2025_641_MOESM4_ESM.zip › EMBOJ-2025-120713R_SourceDataForFigure3/FIG 3D/SMC3ac RAW data.tif]

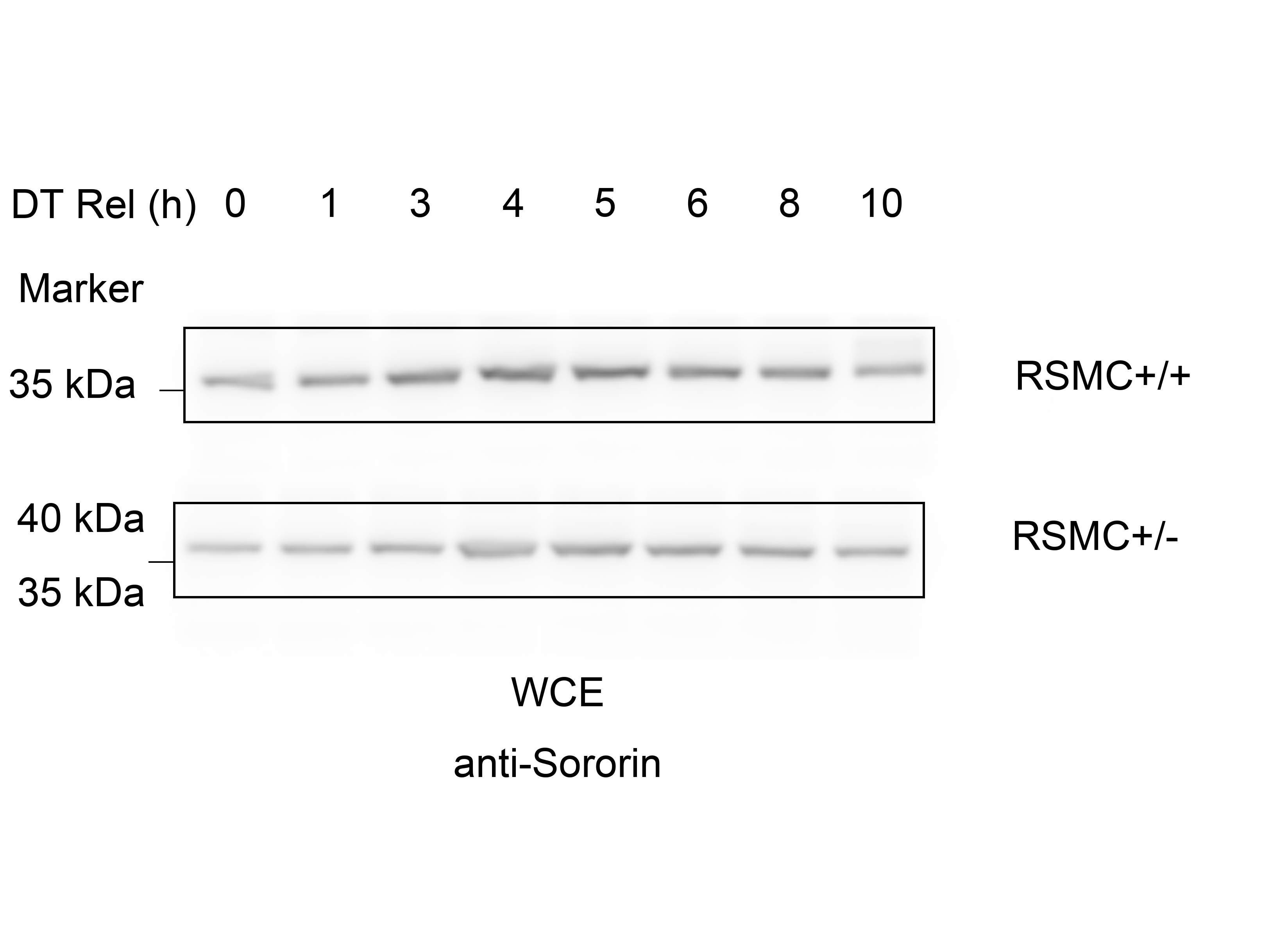

Supplement: Supplementary file 4 — Source data Fig. 3 [file 44318_2025_641_MOESM4_ESM.zip › EMBOJ-2025-120713R_SourceDataForFigure3/FIG 3D/Sororin RAW data.tif]

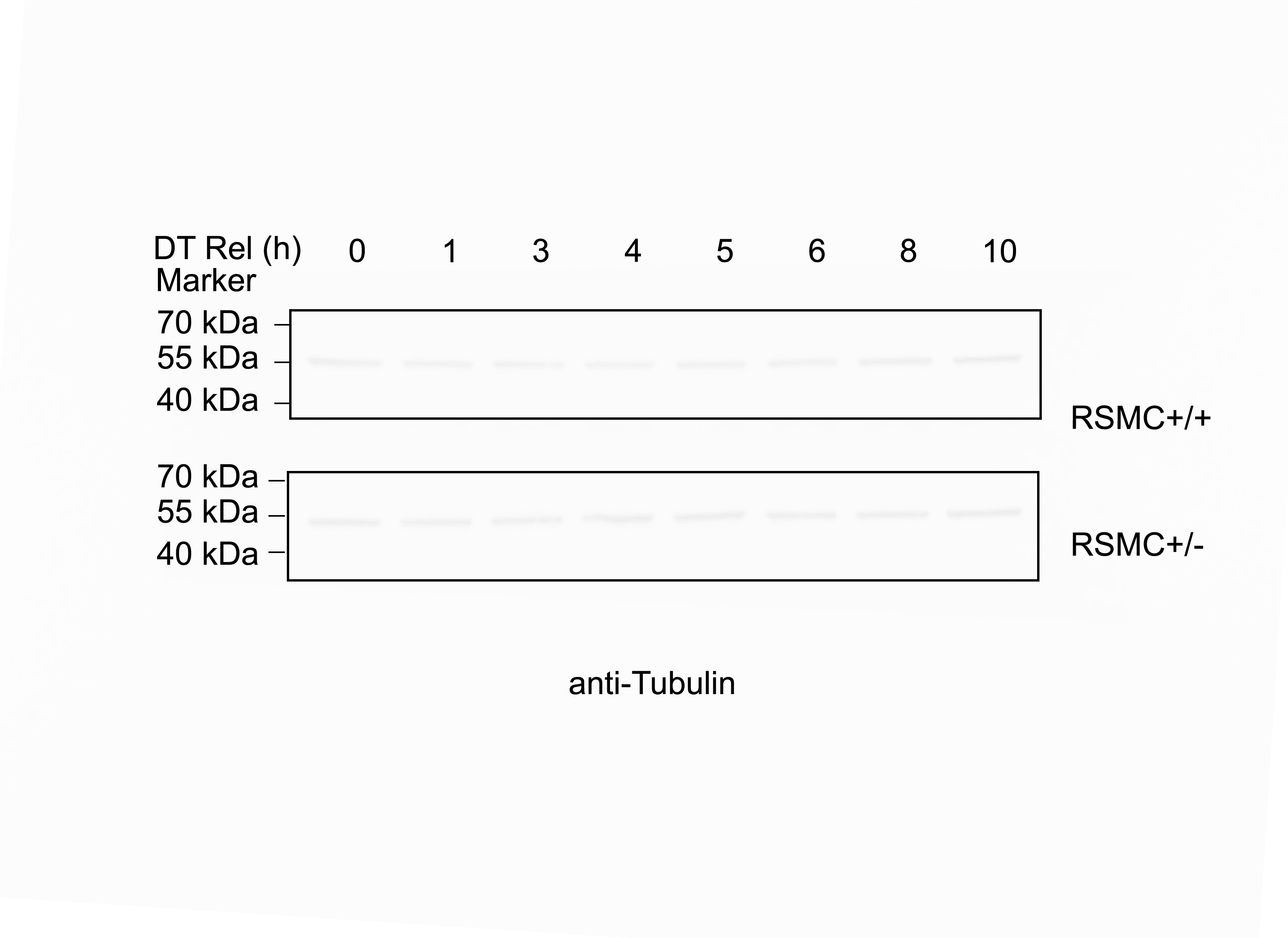

Supplement: Supplementary file 4 — Source data Fig. 3 [file 44318_2025_641_MOESM4_ESM.zip › EMBOJ-2025-120713R_SourceDataForFigure3/FIG 3D/Tubulin RAW data.tif]

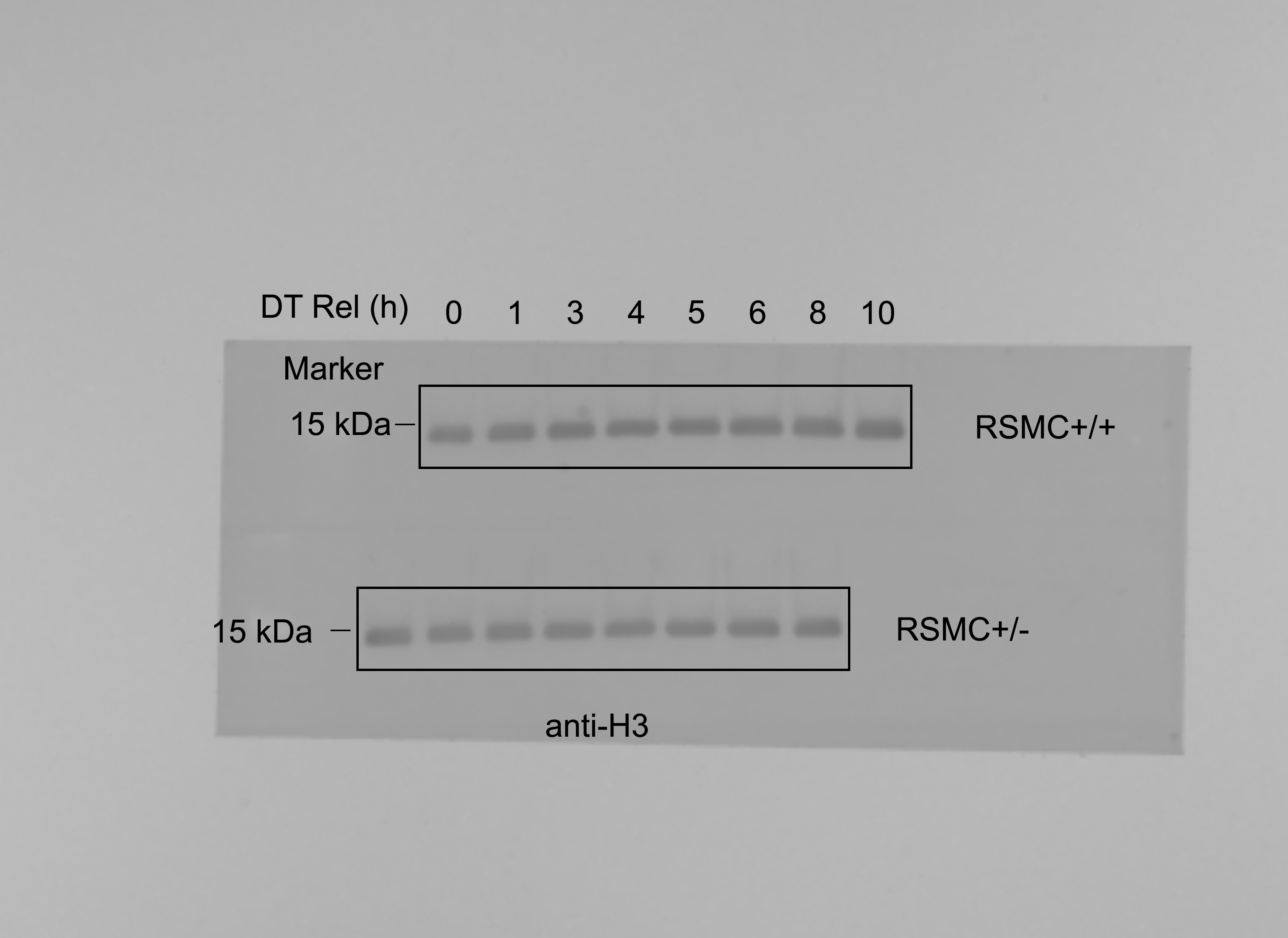

Supplement: Supplementary file 4 — Source data Fig. 3 [file 44318_2025_641_MOESM4_ESM.zip › EMBOJ-2025-120713R_SourceDataForFigure3/FIG 3E/H3 RAW data.tif]

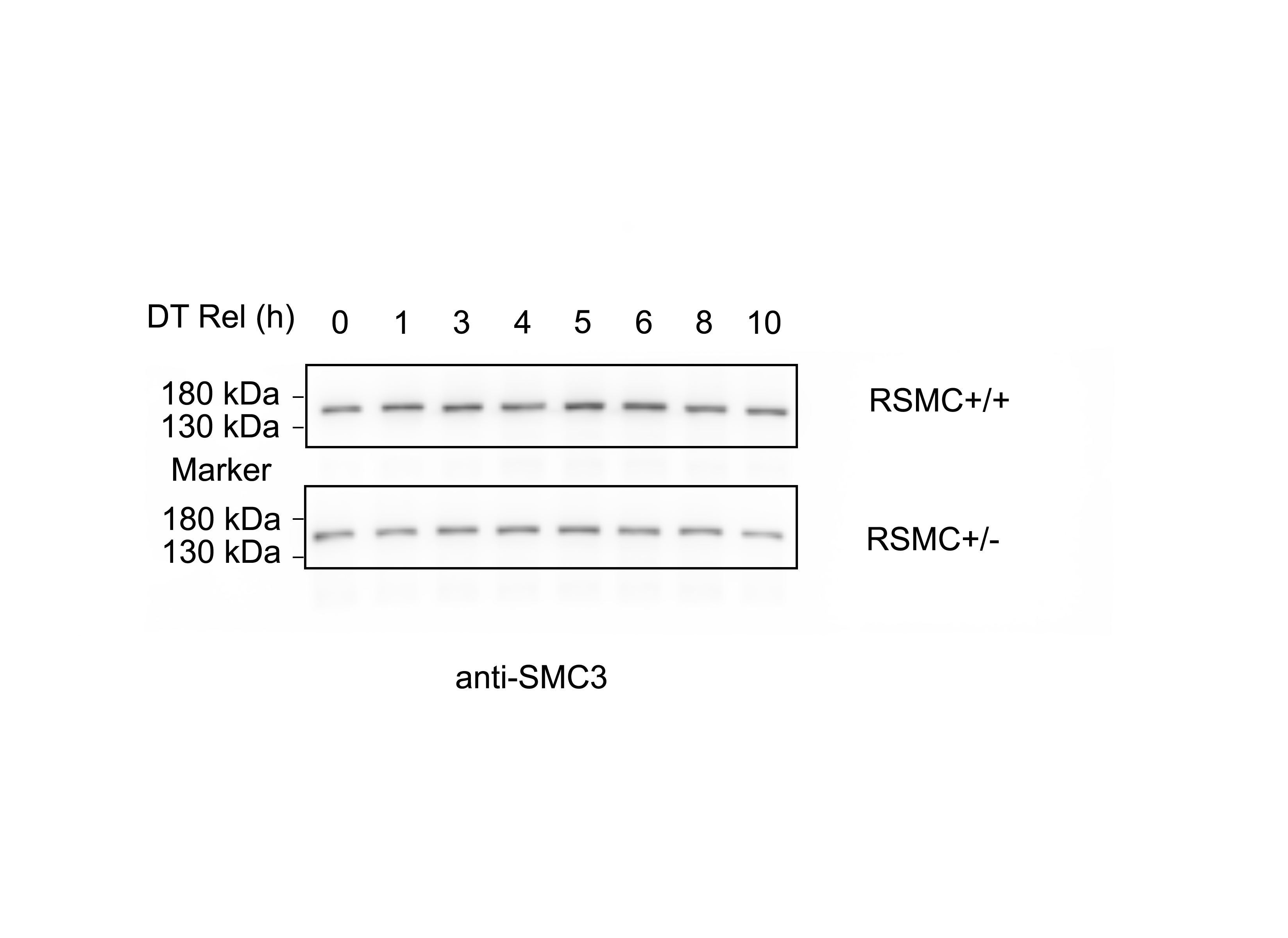

Supplement: Supplementary file 4 — Source data Fig. 3 [file 44318_2025_641_MOESM4_ESM.zip › EMBOJ-2025-120713R_SourceDataForFigure3/FIG 3E/SMC3 RAW data.tif]

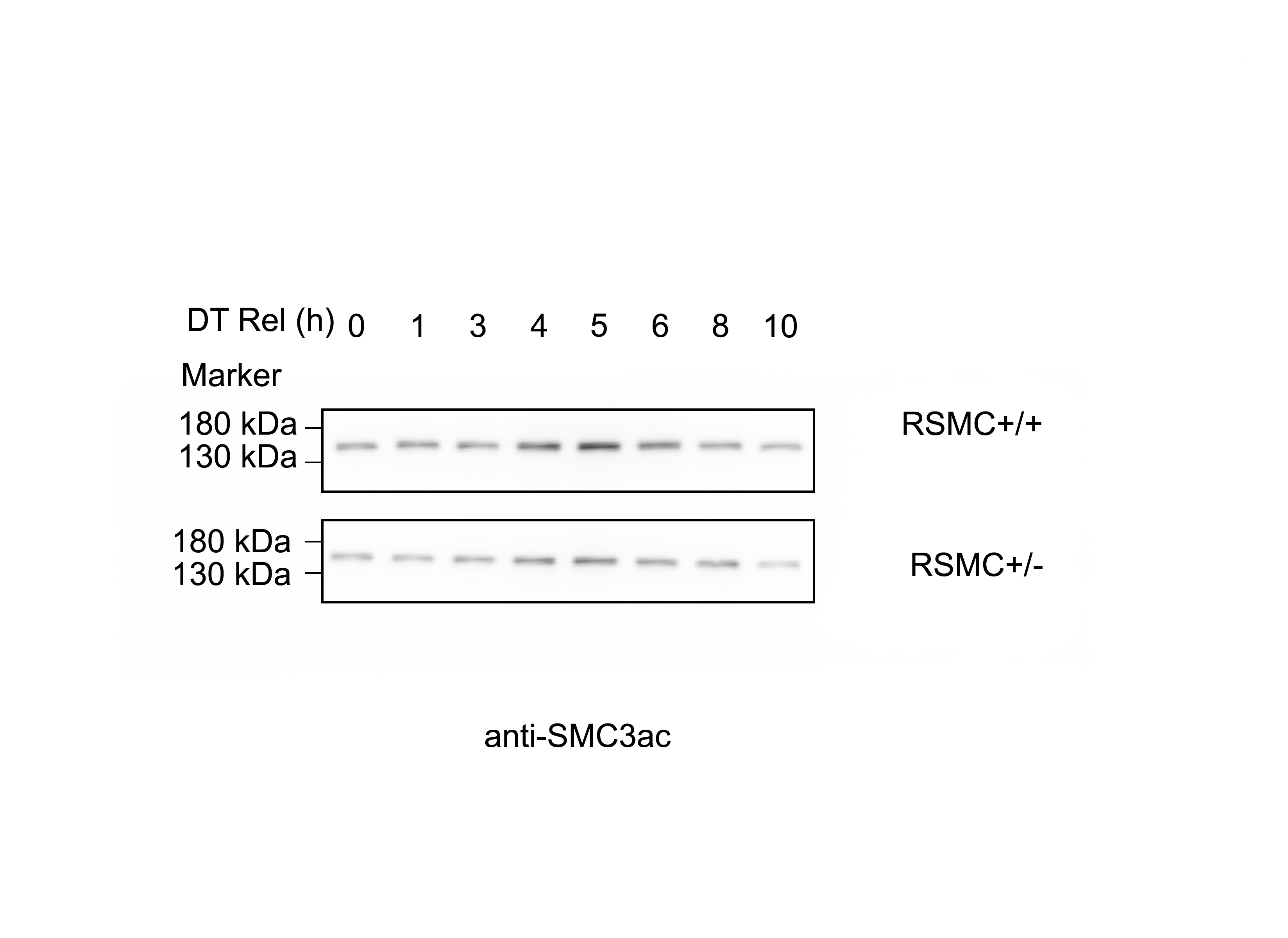

Supplement: Supplementary file 4 — Source data Fig. 3 [file 44318_2025_641_MOESM4_ESM.zip › EMBOJ-2025-120713R_SourceDataForFigure3/FIG 3E/SMC3ac RAW data.tif]

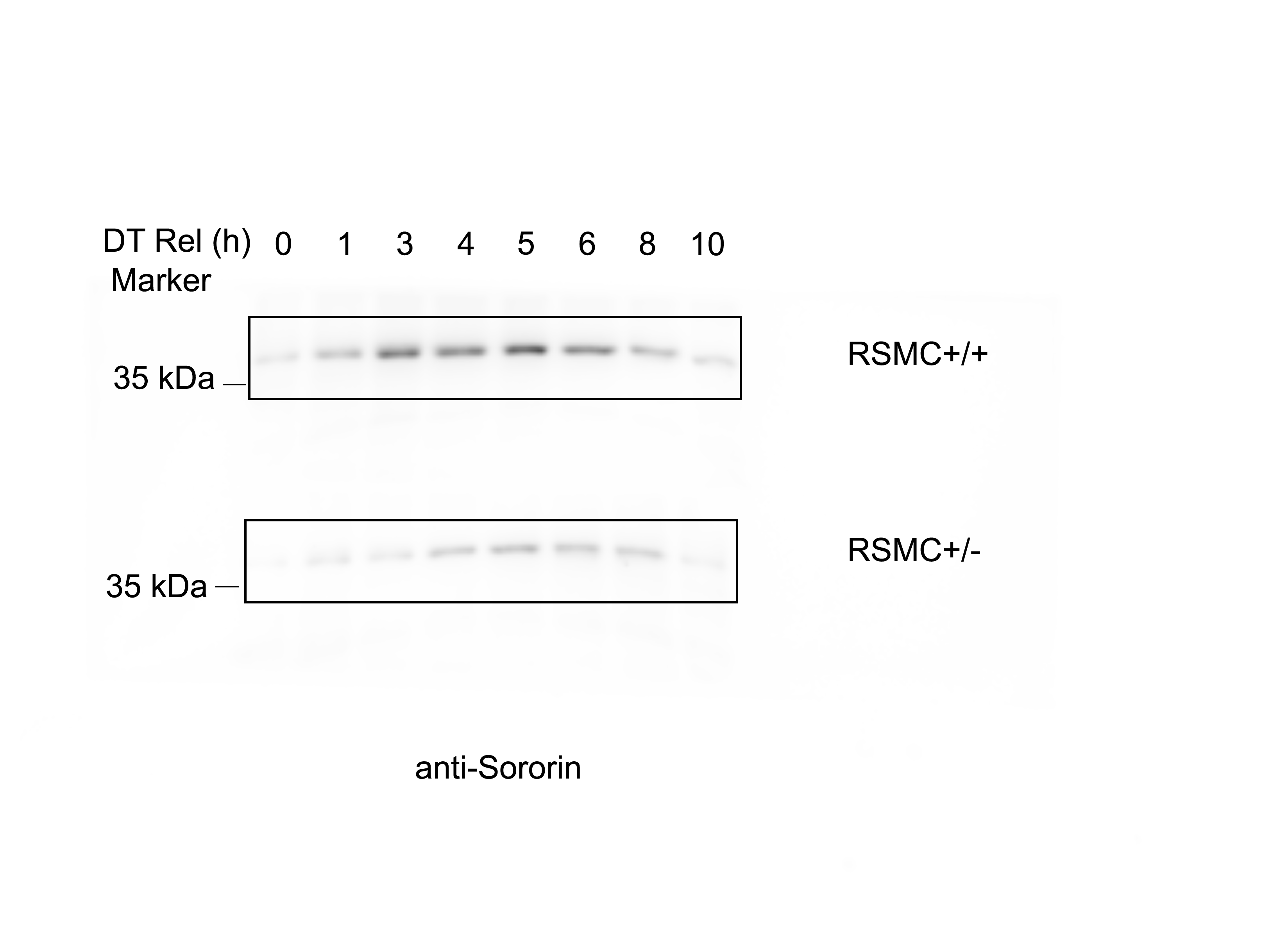

Supplement: Supplementary file 4 — Source data Fig. 3 [file 44318_2025_641_MOESM4_ESM.zip › EMBOJ-2025-120713R_SourceDataForFigure3/FIG 3E/Sororin RAW data.tif]

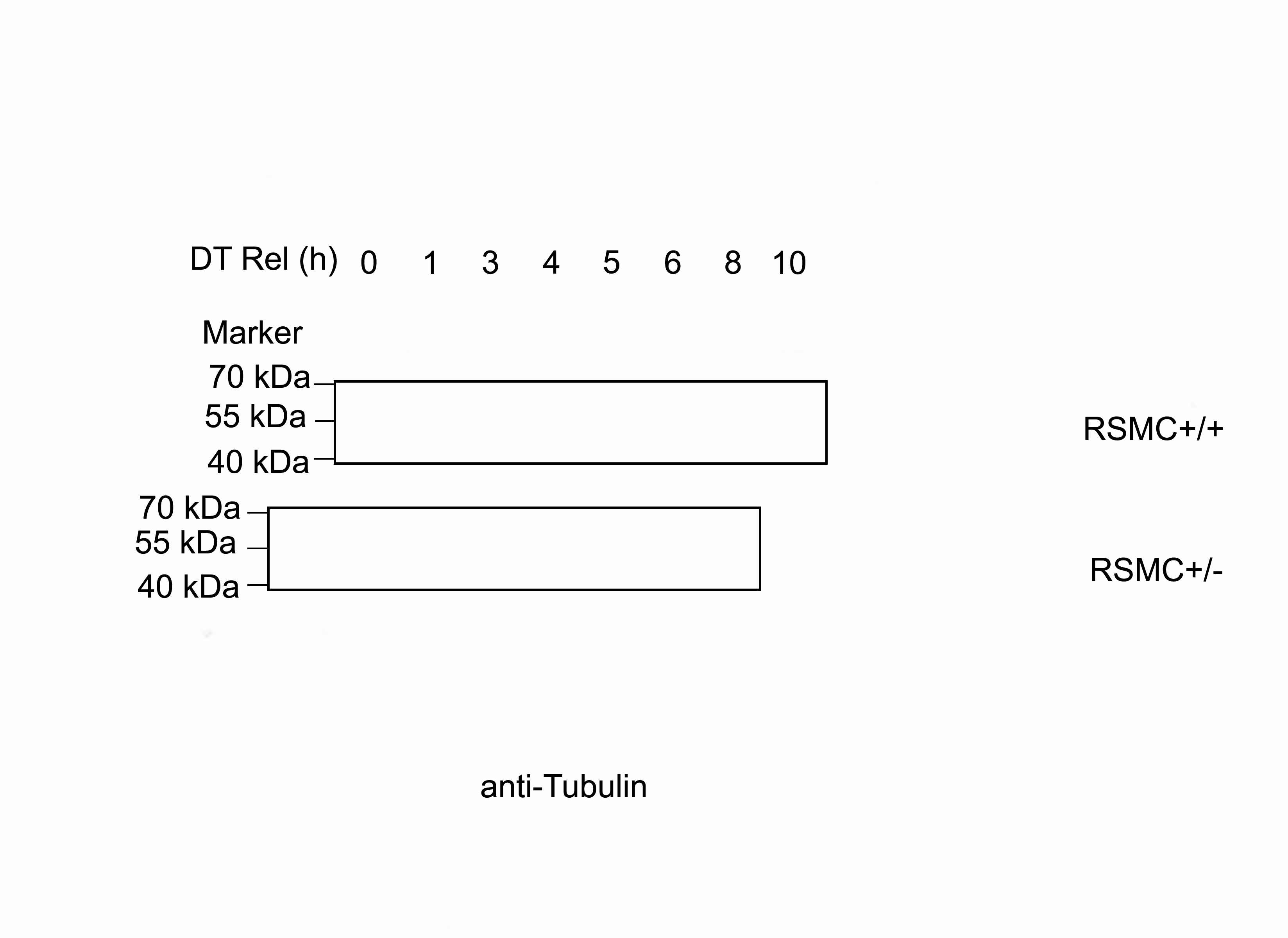

Supplement: Supplementary file 4 — Source data Fig. 3 [file 44318_2025_641_MOESM4_ESM.zip › EMBOJ-2025-120713R_SourceDataForFigure3/FIG 3E/Tubulin RAW data.tif]

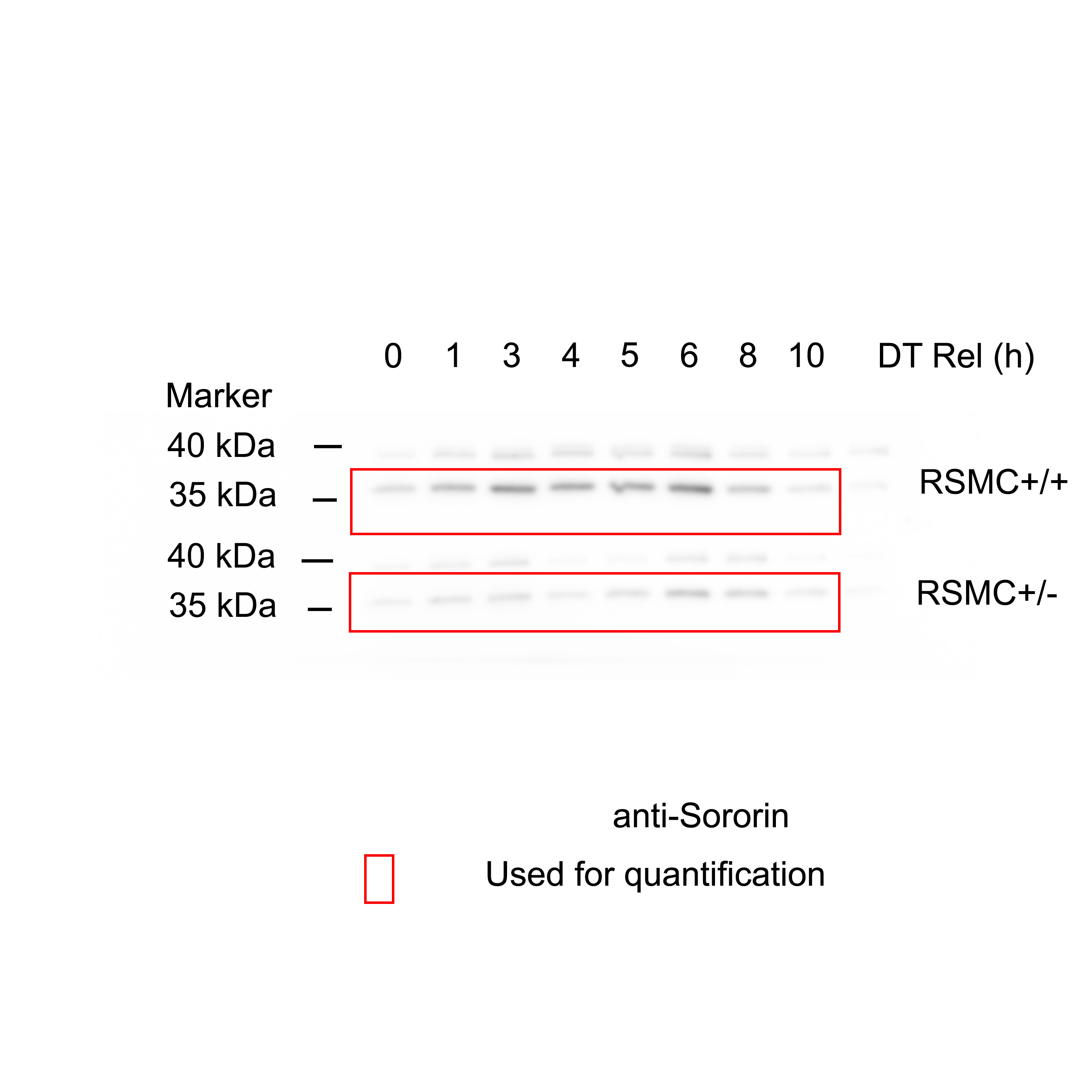

Supplement: Supplementary file 4 — Source data Fig. 3 [file 44318_2025_641_MOESM4_ESM.zip › EMBOJ-2025-120713R_SourceDataForFigure3/FIG 3F/EXP2/Sororin RAW data.tif]

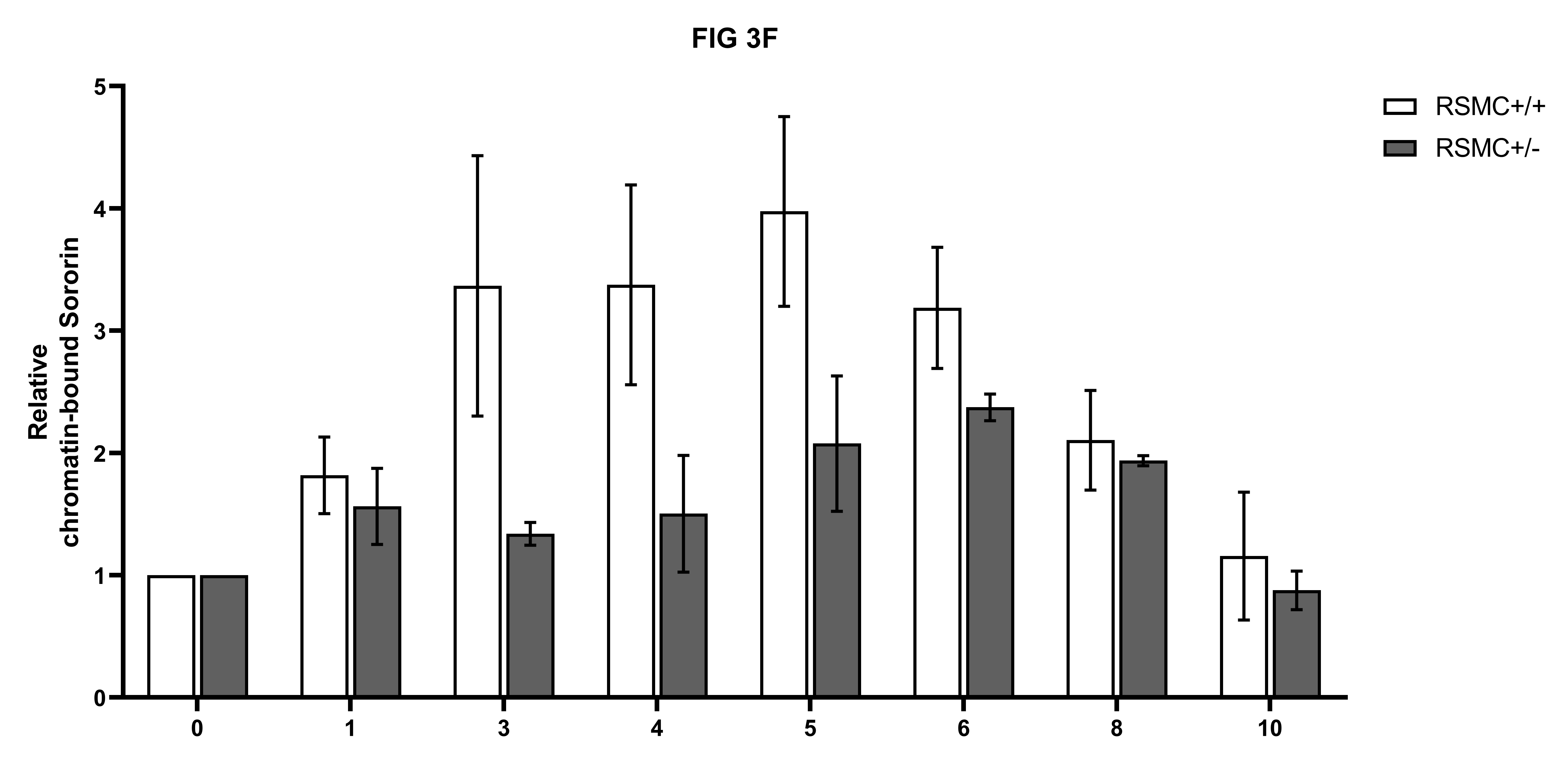

Supplement: Supplementary file 4 — Source data Fig. 3 [file 44318_2025_641_MOESM4_ESM.zip › EMBOJ-2025-120713R_SourceDataForFigure3/FIG 3F/FIG 3F before PS.tif]

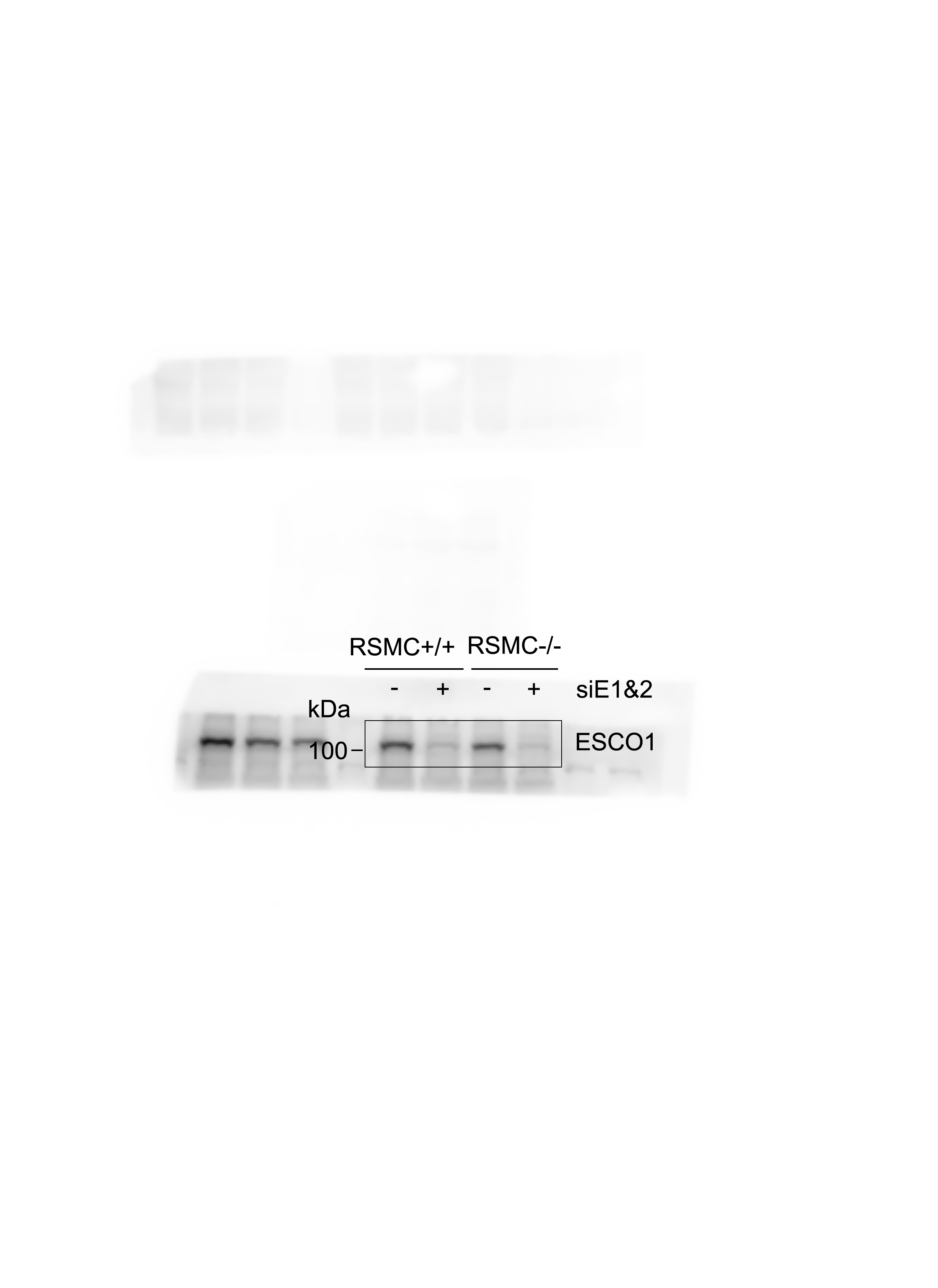

Supplement: Supplementary file 5 — Source data Fig. 4 [file 44318_2025_641_MOESM5_ESM.zip › EMBOJ-2025-120713R_SourceDataForFigure4/FIG 4A/ESCO1-SourceData.tif]

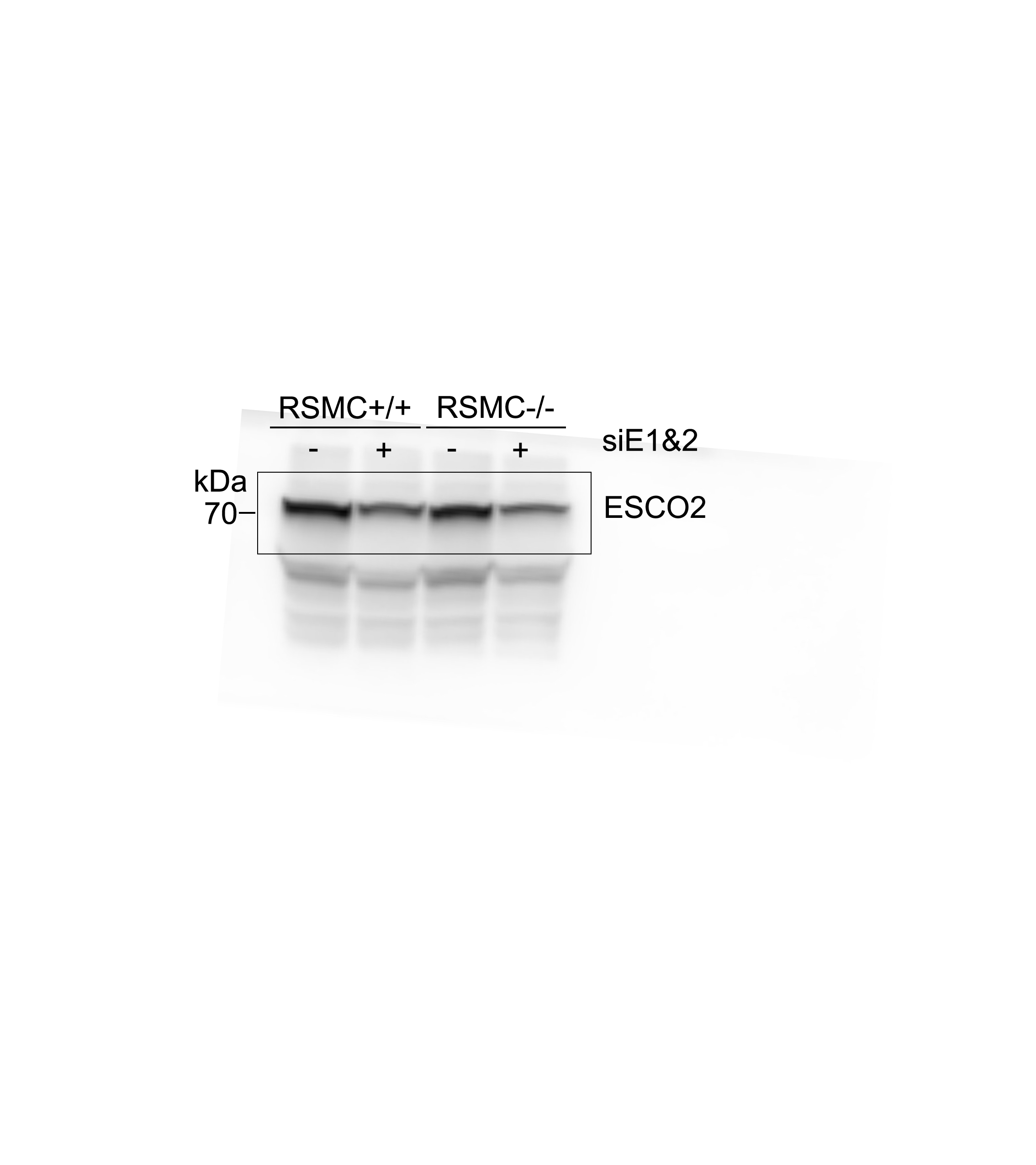

Supplement: Supplementary file 5 — Source data Fig. 4 [file 44318_2025_641_MOESM5_ESM.zip › EMBOJ-2025-120713R_SourceDataForFigure4/FIG 4A/ESCO2-SourceData.tif]

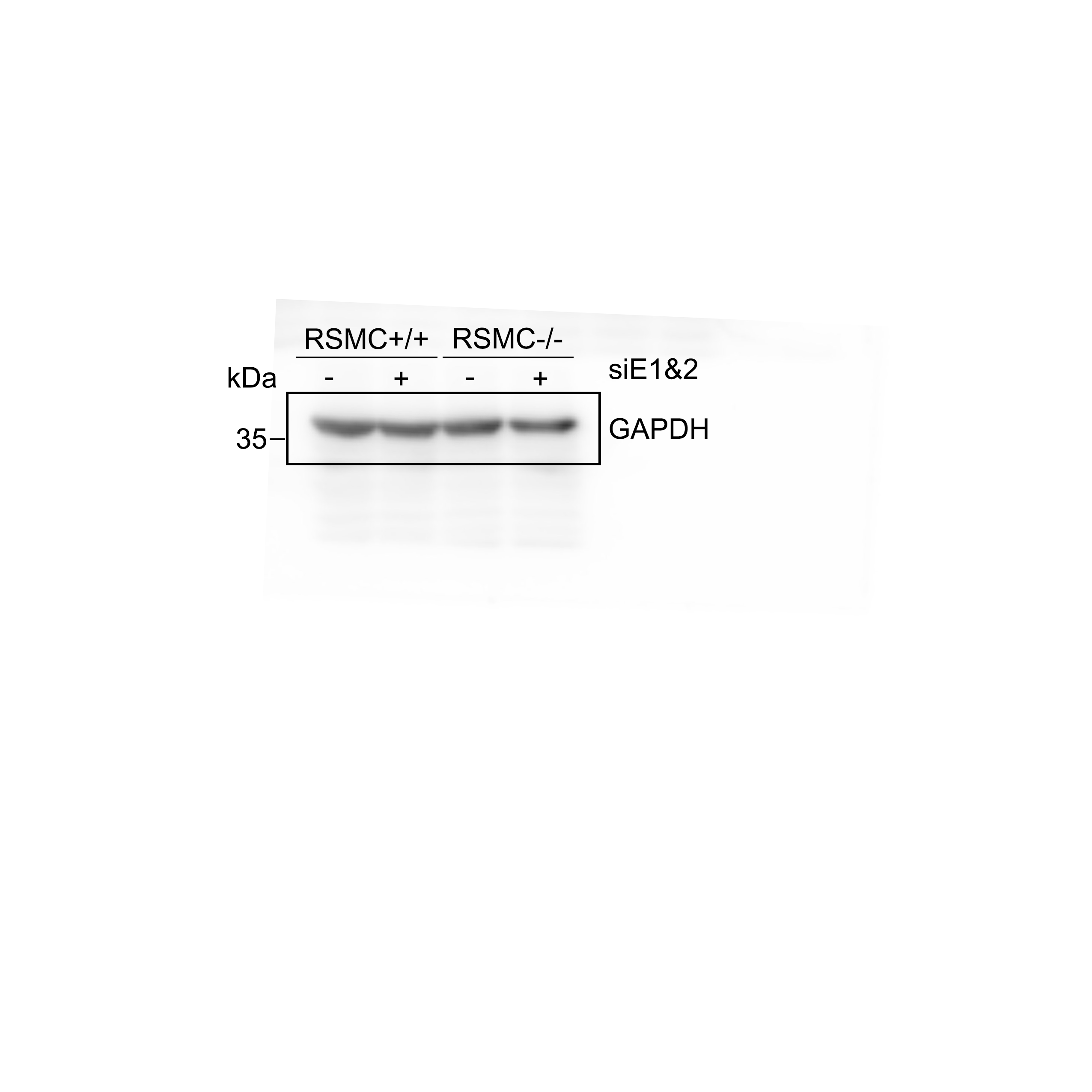

Supplement: Supplementary file 5 — Source data Fig. 4 [file 44318_2025_641_MOESM5_ESM.zip › EMBOJ-2025-120713R_SourceDataForFigure4/FIG 4A/GAPDH-SourceData.tif]

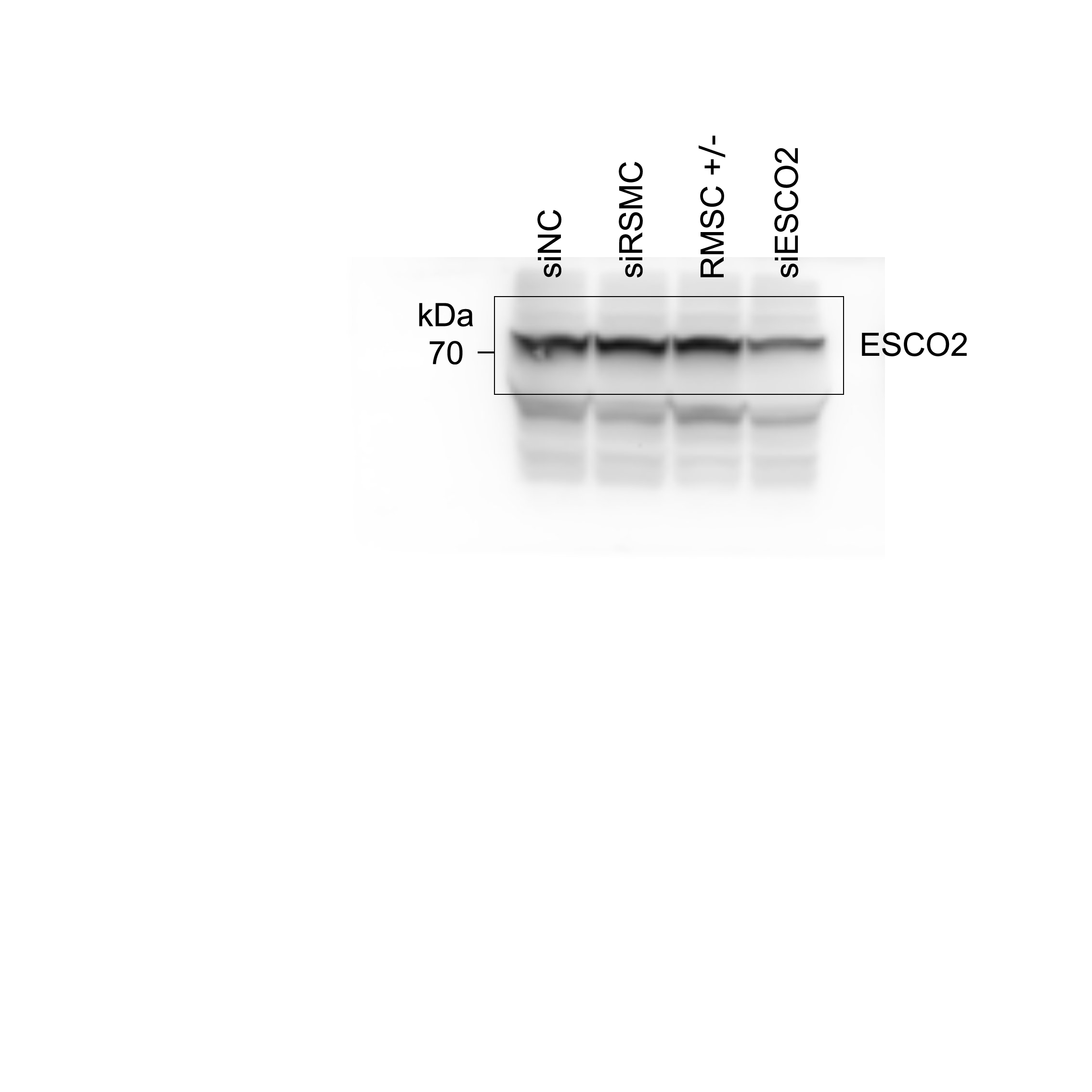

Supplement: Supplementary file 5 — Source data Fig. 4 [file 44318_2025_641_MOESM5_ESM.zip › EMBOJ-2025-120713R_SourceDataForFigure4/FIG 4B/ESCO2-SourceData.tif]

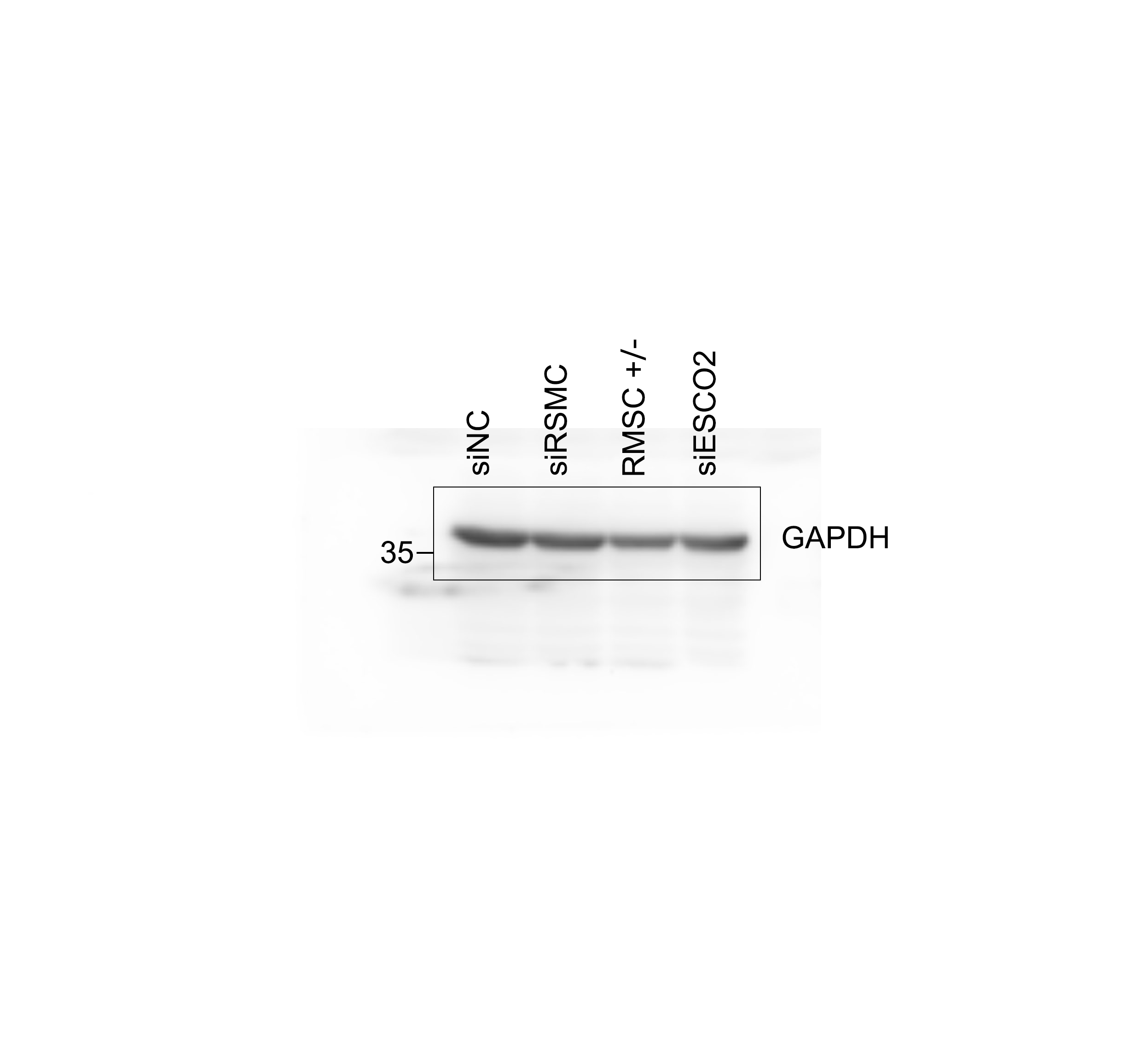

Supplement: Supplementary file 5 — Source data Fig. 4 [file 44318_2025_641_MOESM5_ESM.zip › EMBOJ-2025-120713R_SourceDataForFigure4/FIG 4B/GAPDH-SourceData.tif]

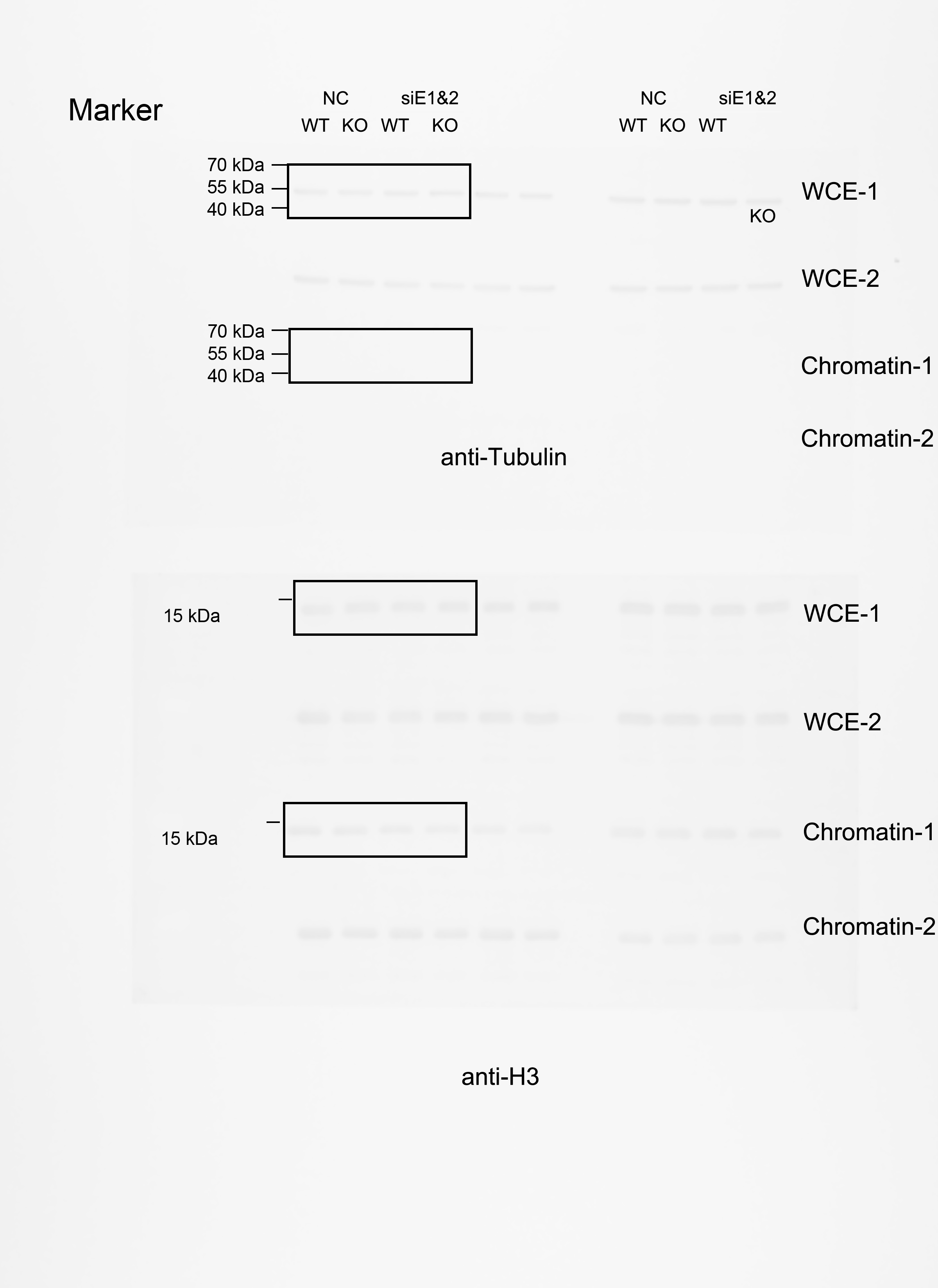

Supplement: Supplementary file 5 — Source data Fig. 4 [file 44318_2025_641_MOESM5_ESM.zip › EMBOJ-2025-120713R_SourceDataForFigure4/FIG 4C/H3 & Tubulin RAW data.tif]

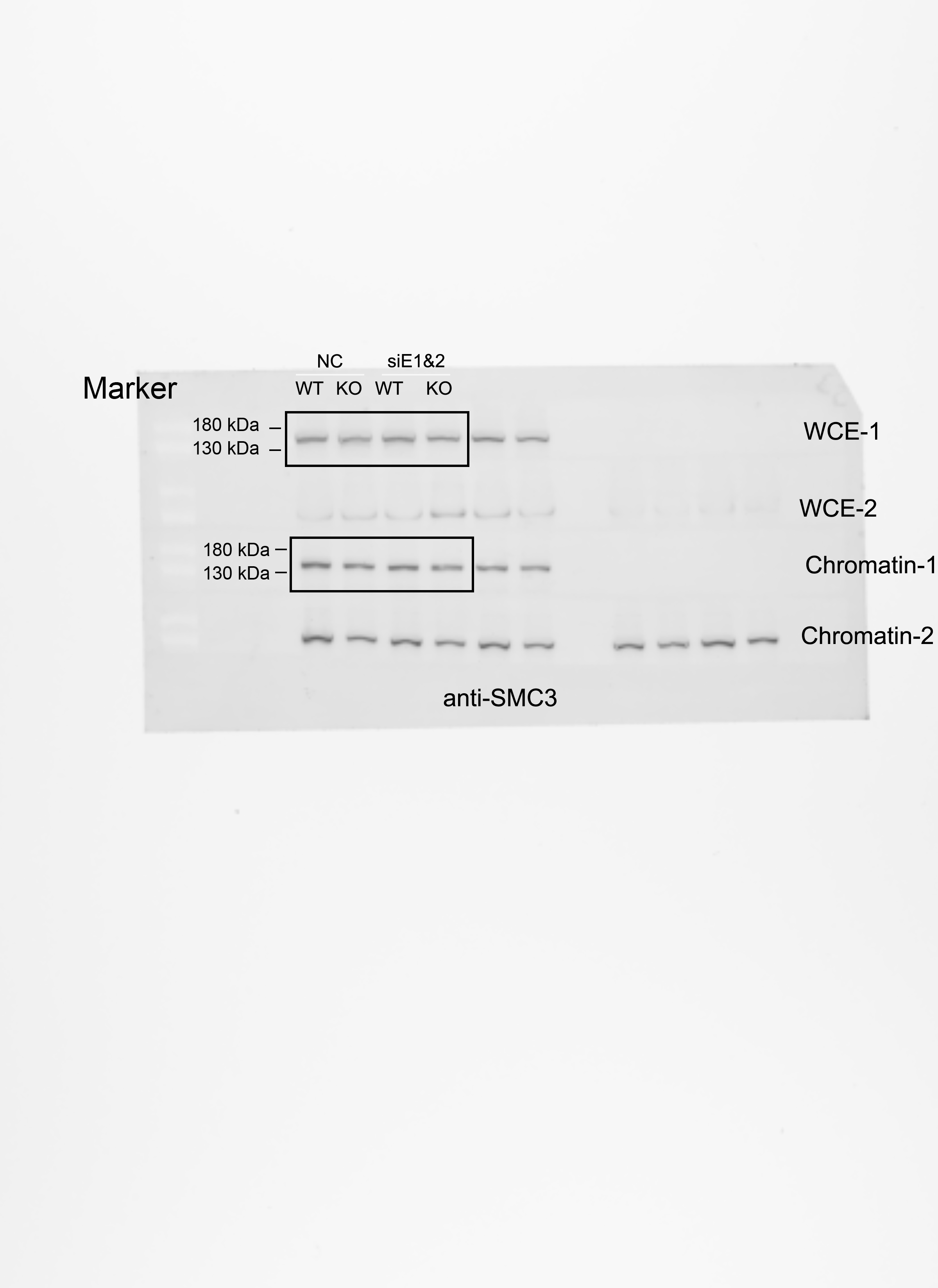

Supplement: Supplementary file 5 — Source data Fig. 4 [file 44318_2025_641_MOESM5_ESM.zip › EMBOJ-2025-120713R_SourceDataForFigure4/FIG 4C/SMC3 RAW data.tif]

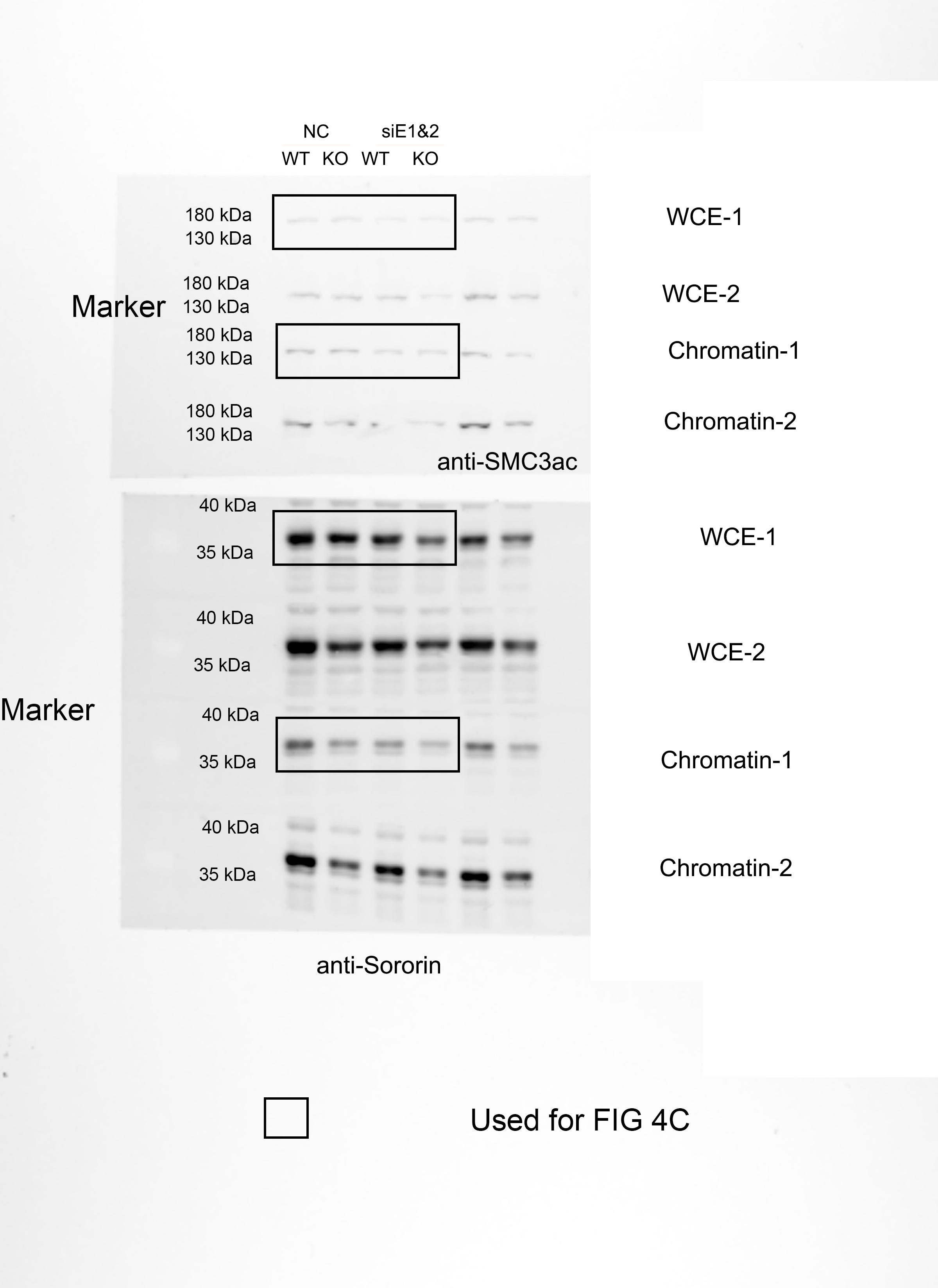

Supplement: Supplementary file 5 — Source data Fig. 4 [file 44318_2025_641_MOESM5_ESM.zip › EMBOJ-2025-120713R_SourceDataForFigure4/FIG 4C/SMC3ac & Sororin RAW data (with Marker).tif]

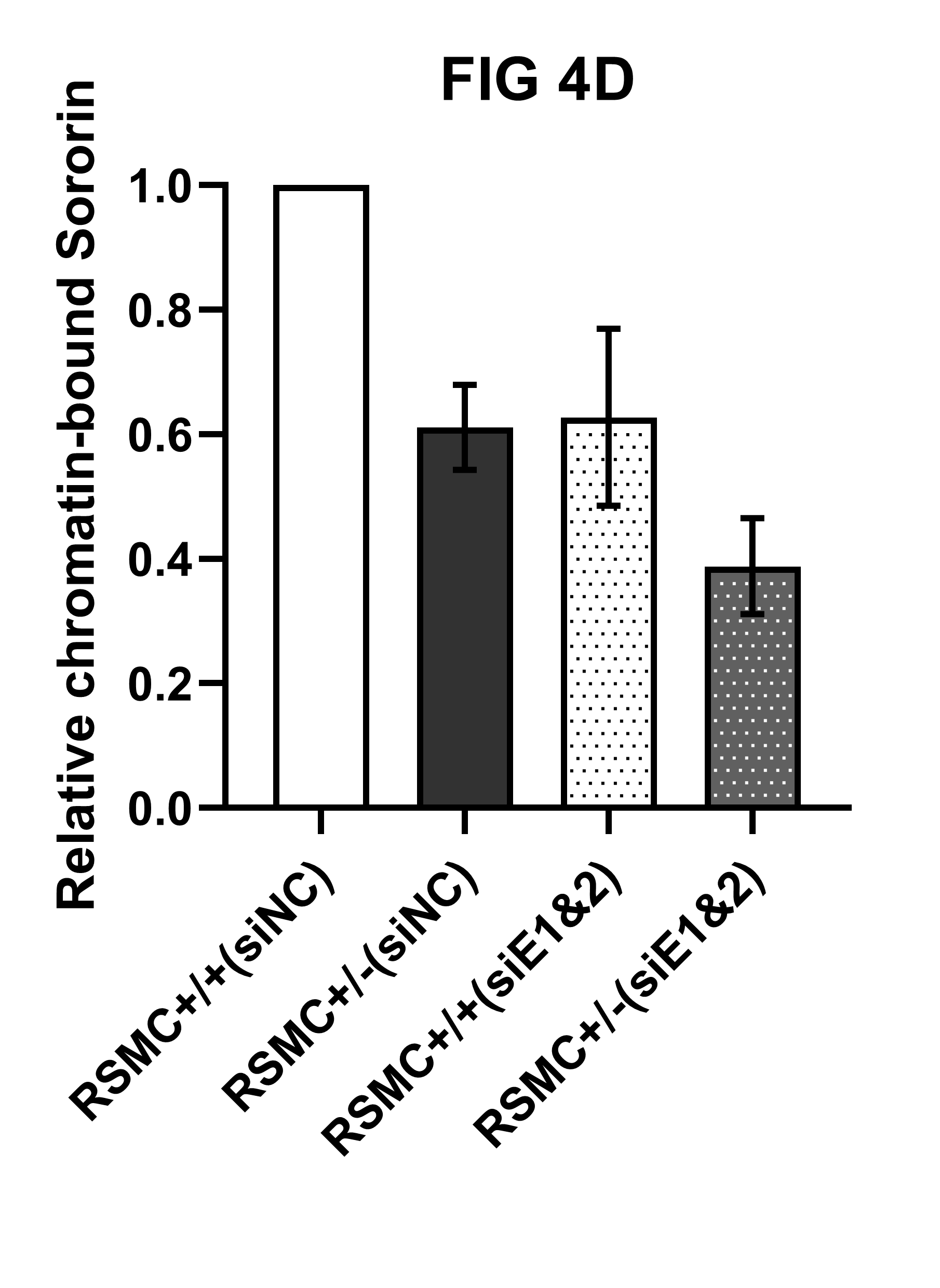

Supplement: Supplementary file 5 — Source data Fig. 4 [file 44318_2025_641_MOESM5_ESM.zip › EMBOJ-2025-120713R_SourceDataForFigure4/FIG 4D/FIG 4D before PS.tif]

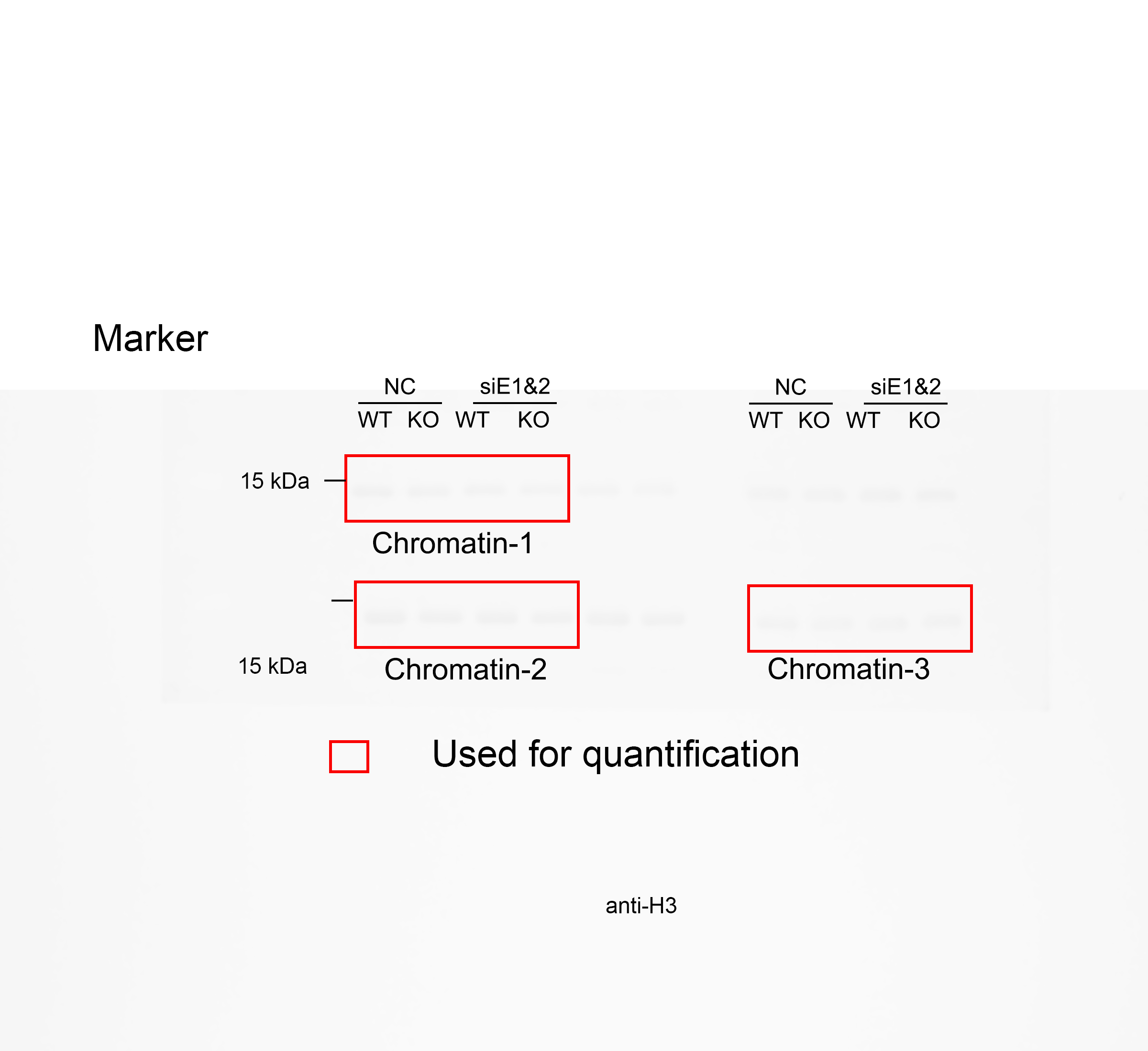

Supplement: Supplementary file 5 — Source data Fig. 4 [file 44318_2025_641_MOESM5_ESM.zip › EMBOJ-2025-120713R_SourceDataForFigure4/FIG 4D/H3 RAW data (EXP1-3).tif]

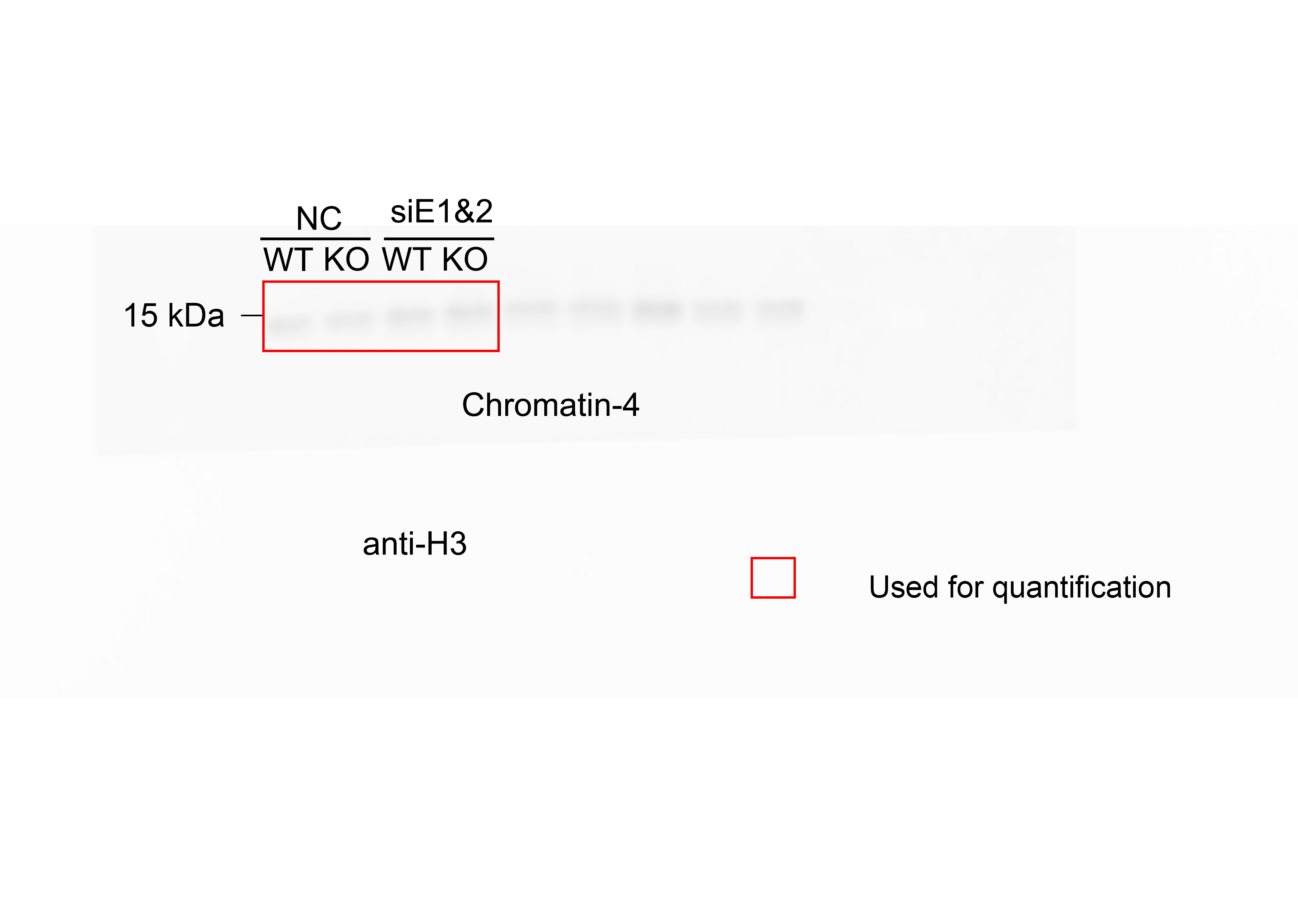

Supplement: Supplementary file 5 — Source data Fig. 4 [file 44318_2025_641_MOESM5_ESM.zip › EMBOJ-2025-120713R_SourceDataForFigure4/FIG 4D/H3 RAW data(EXP4).tif]

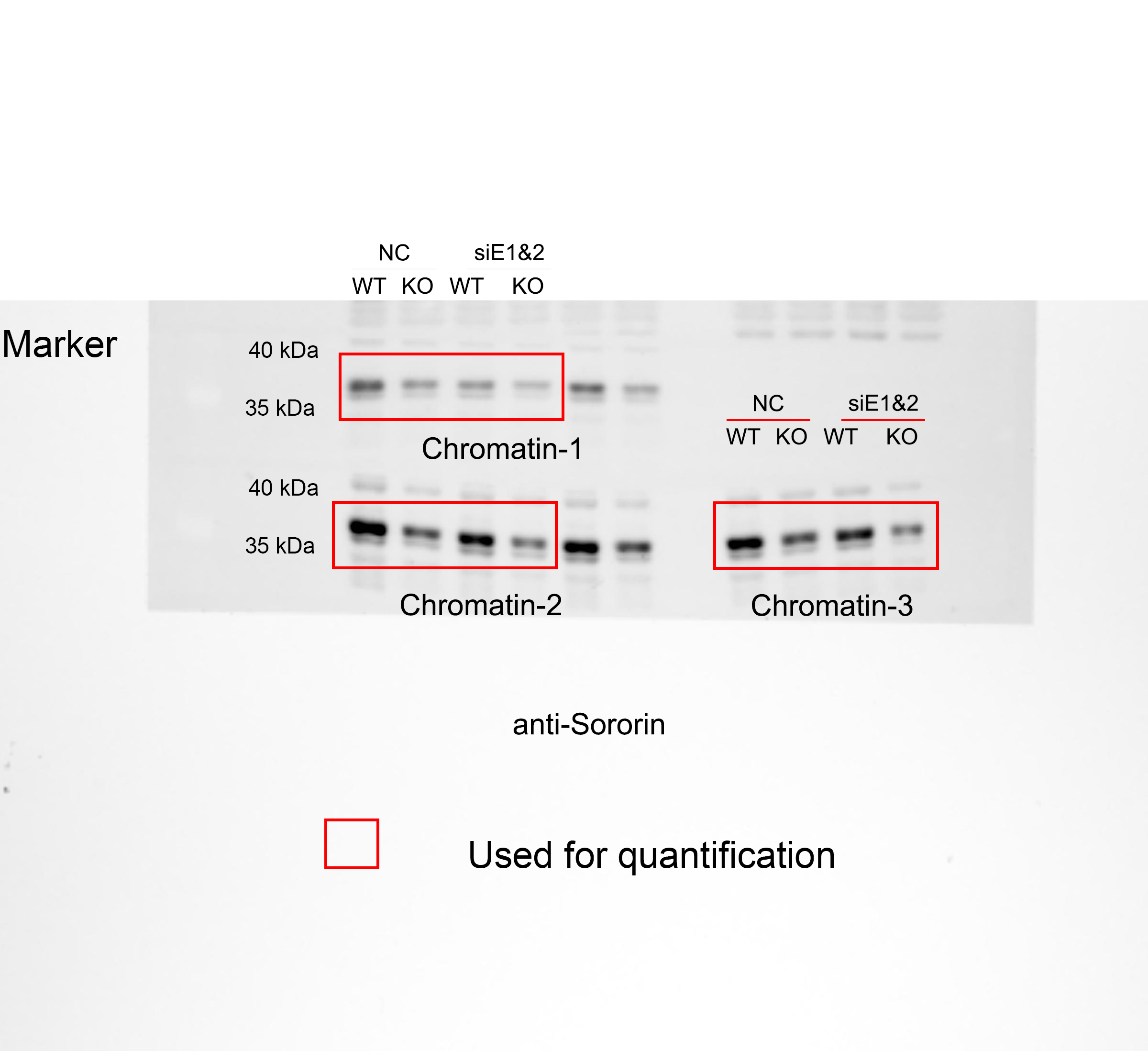

Supplement: Supplementary file 5 — Source data Fig. 4 [file 44318_2025_641_MOESM5_ESM.zip › EMBOJ-2025-120713R_SourceDataForFigure4/FIG 4D/Sororin RAW data (with Marker EXP1-3).tif]

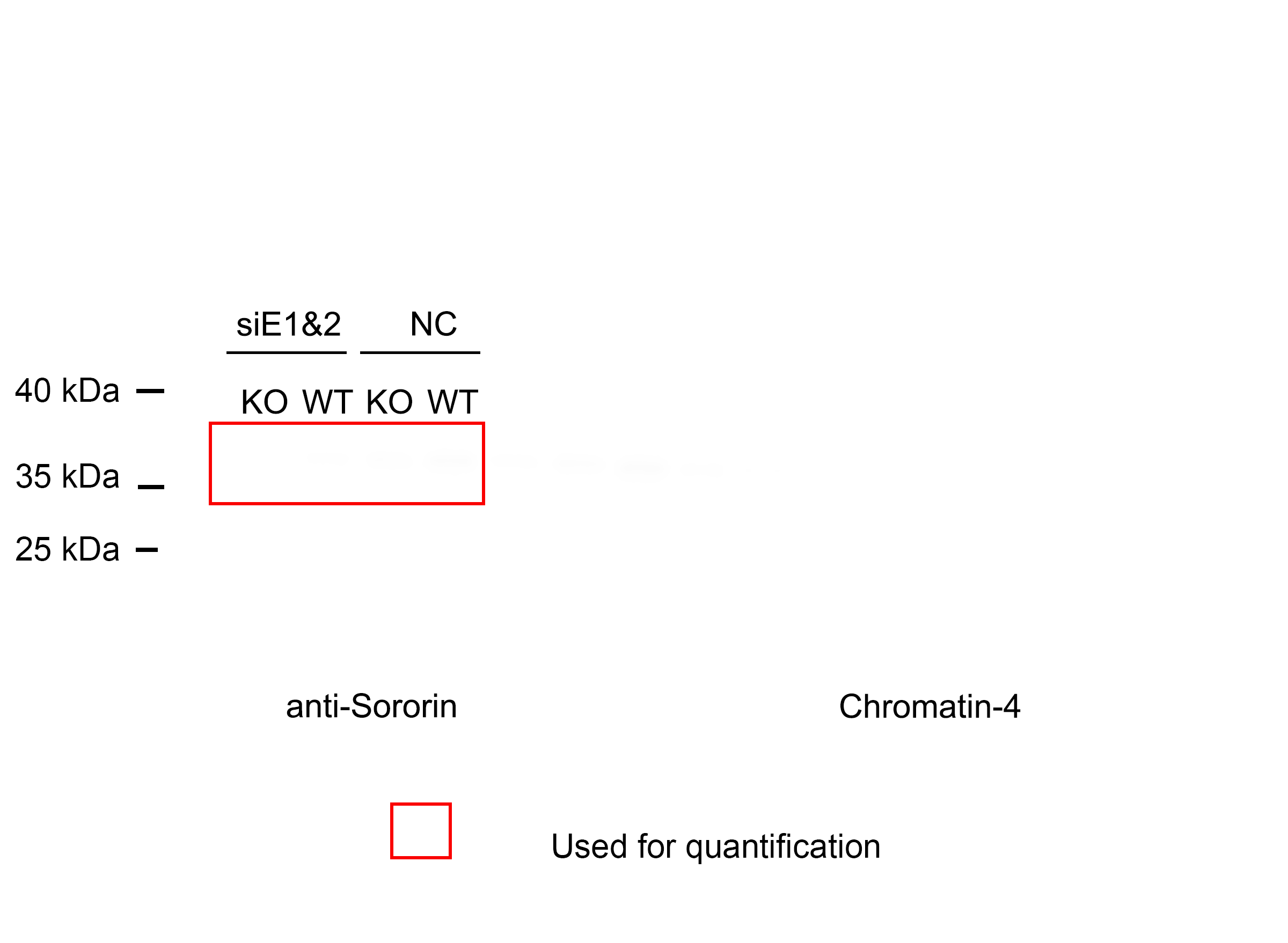

Supplement: Supplementary file 5 — Source data Fig. 4 [file 44318_2025_641_MOESM5_ESM.zip › EMBOJ-2025-120713R_SourceDataForFigure4/FIG 4D/Sororin RAW data(EXP4).tif]

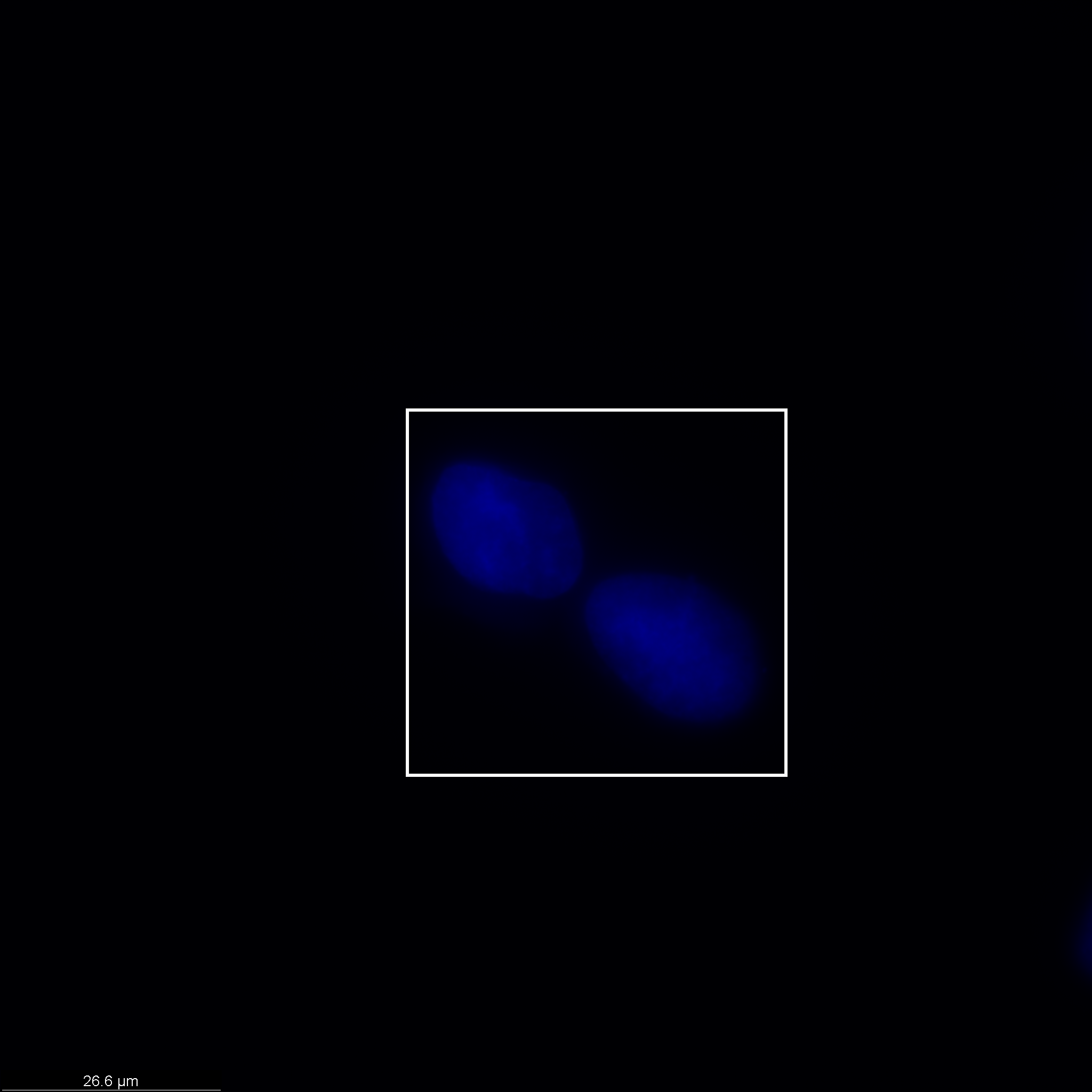

Supplement: Supplementary file 5 — Source data Fig. 4 [file 44318_2025_641_MOESM5_ESM.zip › EMBOJ-2025-120713R_SourceDataForFigure4/FIG 4E/1-RSMC WT+siNC/DAPI.tif]

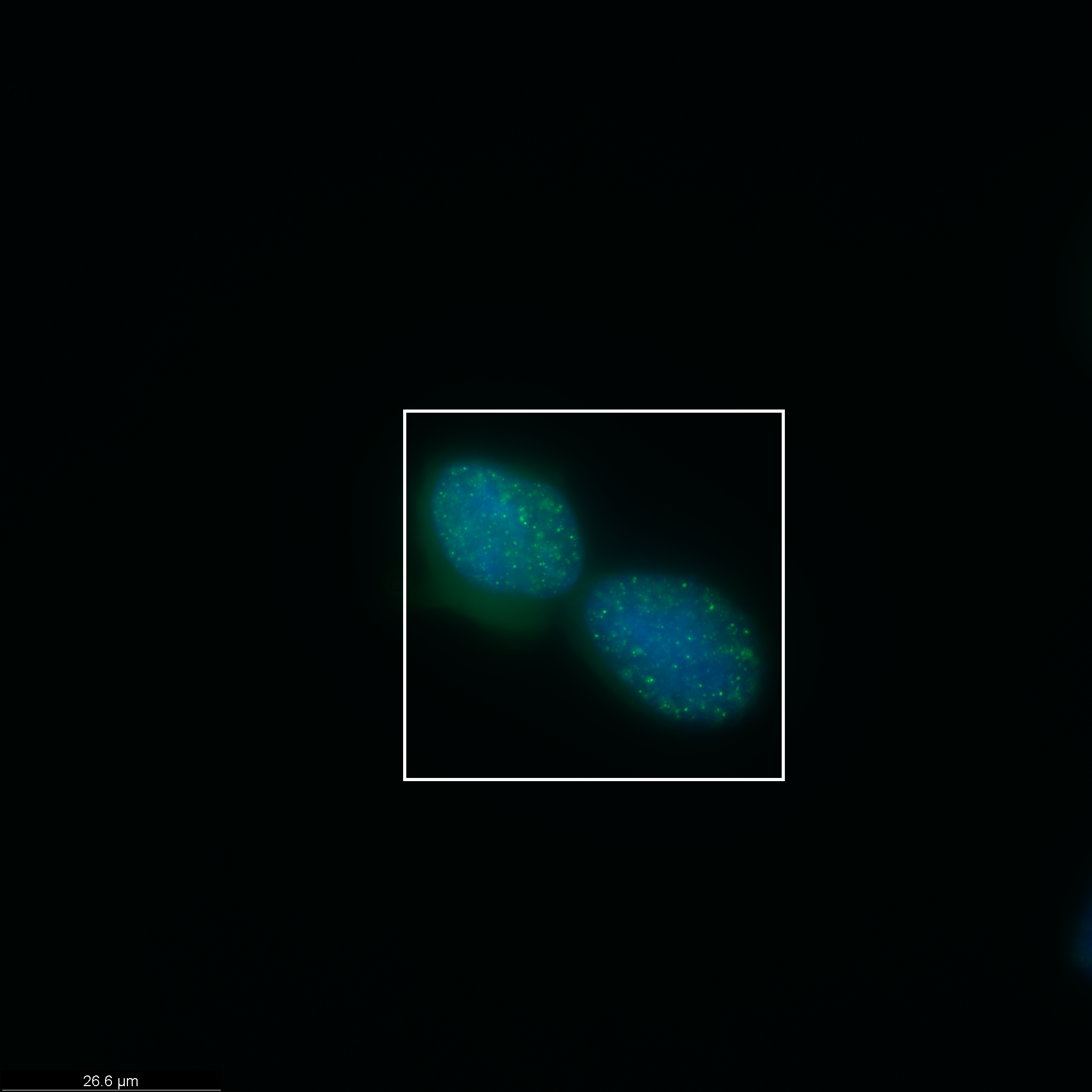

Supplement: Supplementary file 5 — Source data Fig. 4 [file 44318_2025_641_MOESM5_ESM.zip › EMBOJ-2025-120713R_SourceDataForFigure4/FIG 4E/1-RSMC WT+siNC/merge.tif]

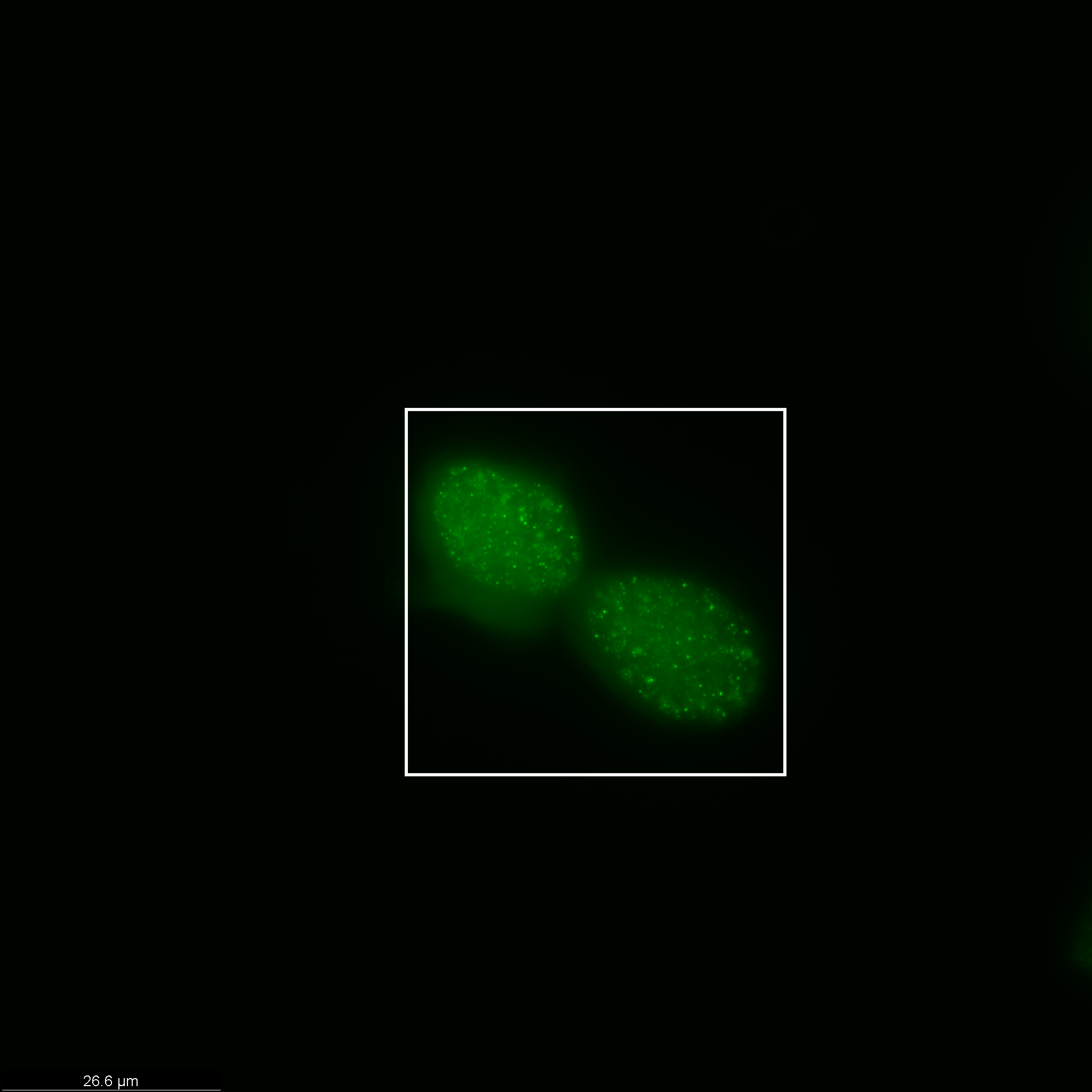

Supplement: Supplementary file 5 — Source data Fig. 4 [file 44318_2025_641_MOESM5_ESM.zip › EMBOJ-2025-120713R_SourceDataForFigure4/FIG 4E/1-RSMC WT+siNC/Sororin.tif]

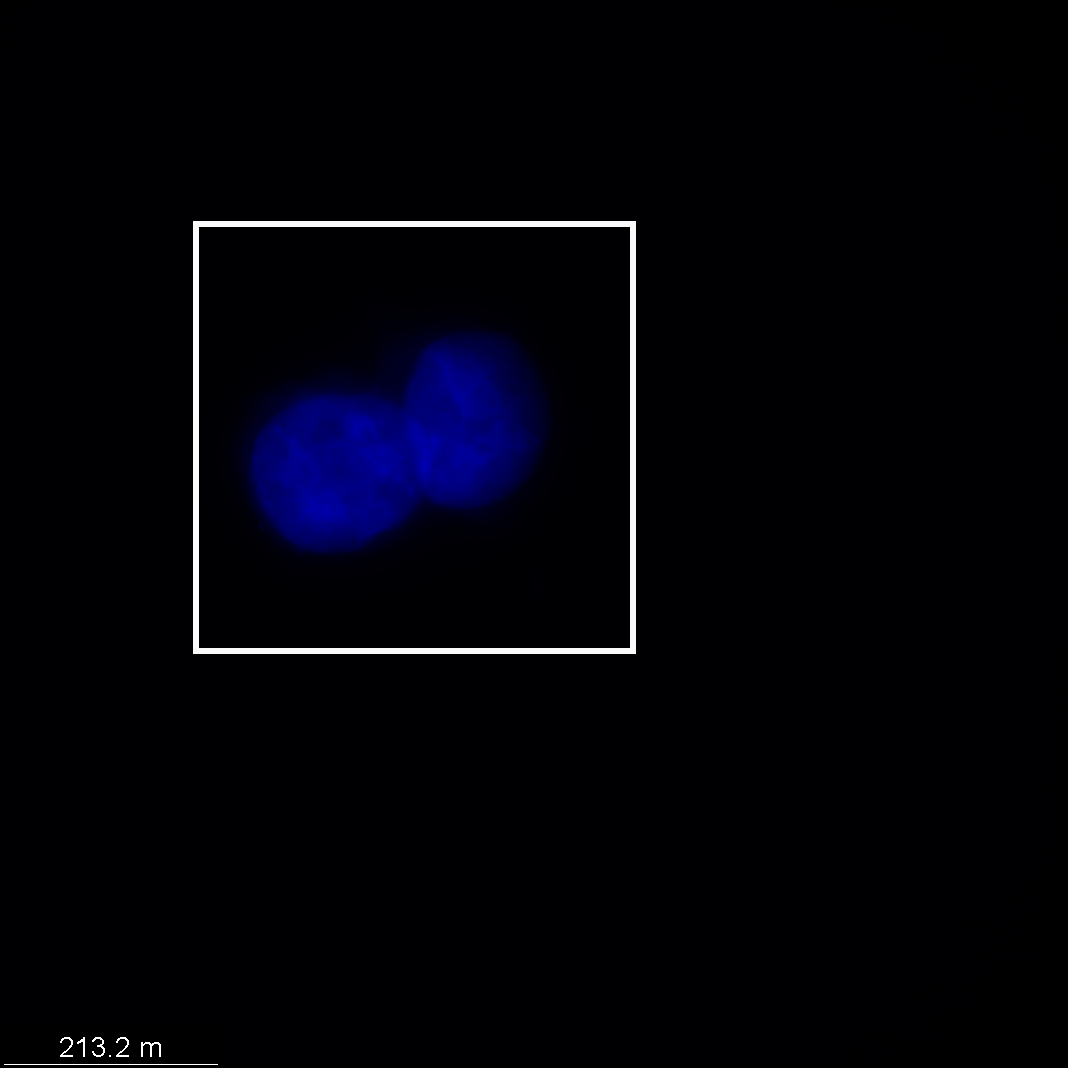

Supplement: Supplementary file 5 — Source data Fig. 4 [file 44318_2025_641_MOESM5_ESM.zip › EMBOJ-2025-120713R_SourceDataForFigure4/FIG 4E/2-RSMC WT+siESCO1&2/DAPI.tif]

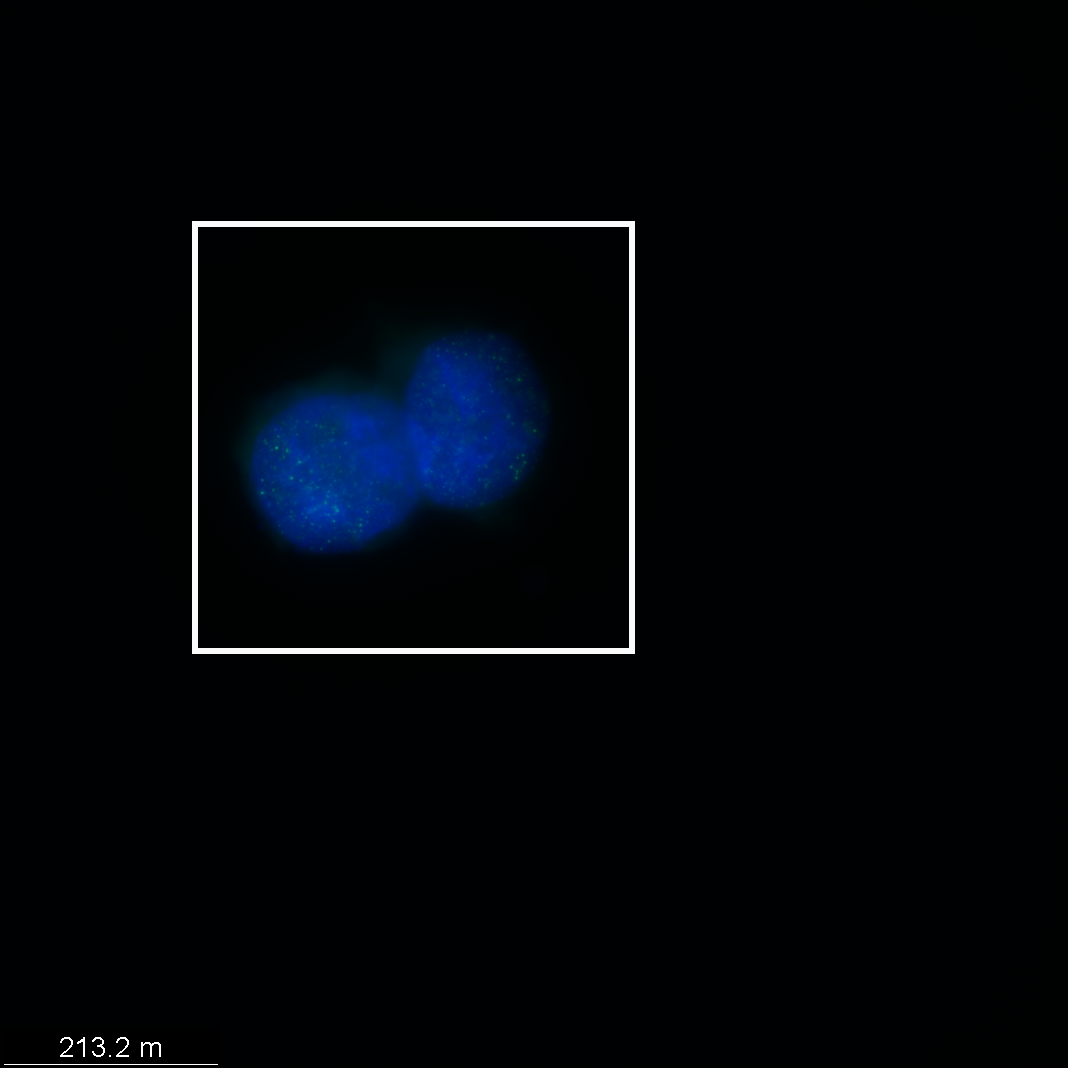

Supplement: Supplementary file 5 — Source data Fig. 4 [file 44318_2025_641_MOESM5_ESM.zip › EMBOJ-2025-120713R_SourceDataForFigure4/FIG 4E/2-RSMC WT+siESCO1&2/merge.tif]

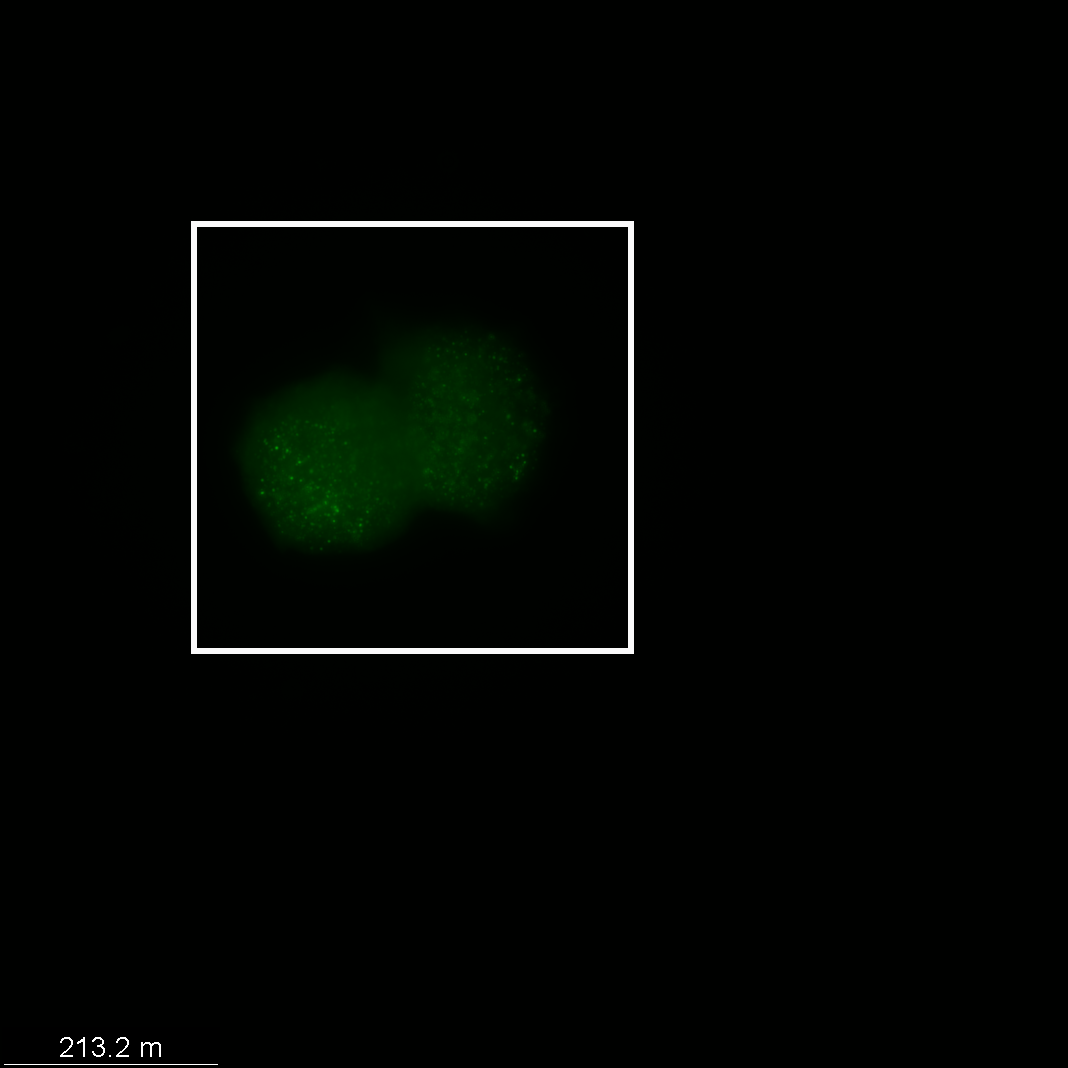

Supplement: Supplementary file 5 — Source data Fig. 4 [file 44318_2025_641_MOESM5_ESM.zip › EMBOJ-2025-120713R_SourceDataForFigure4/FIG 4E/2-RSMC WT+siESCO1&2/Sororin.tif]

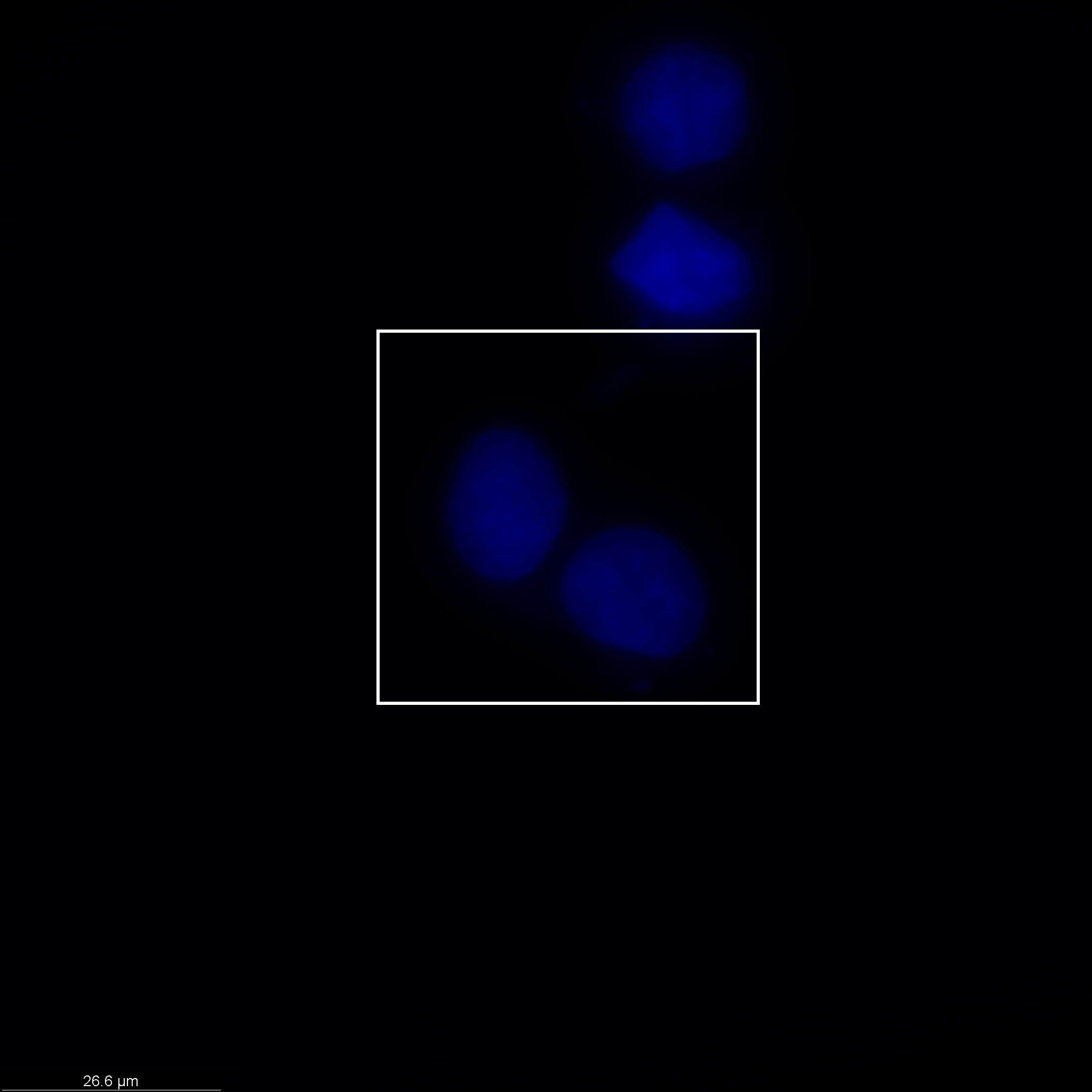

Supplement: Supplementary file 5 — Source data Fig. 4 [file 44318_2025_641_MOESM5_ESM.zip › EMBOJ-2025-120713R_SourceDataForFigure4/FIG 4E/3-RSMC KO+siNC/DAPI.tif]

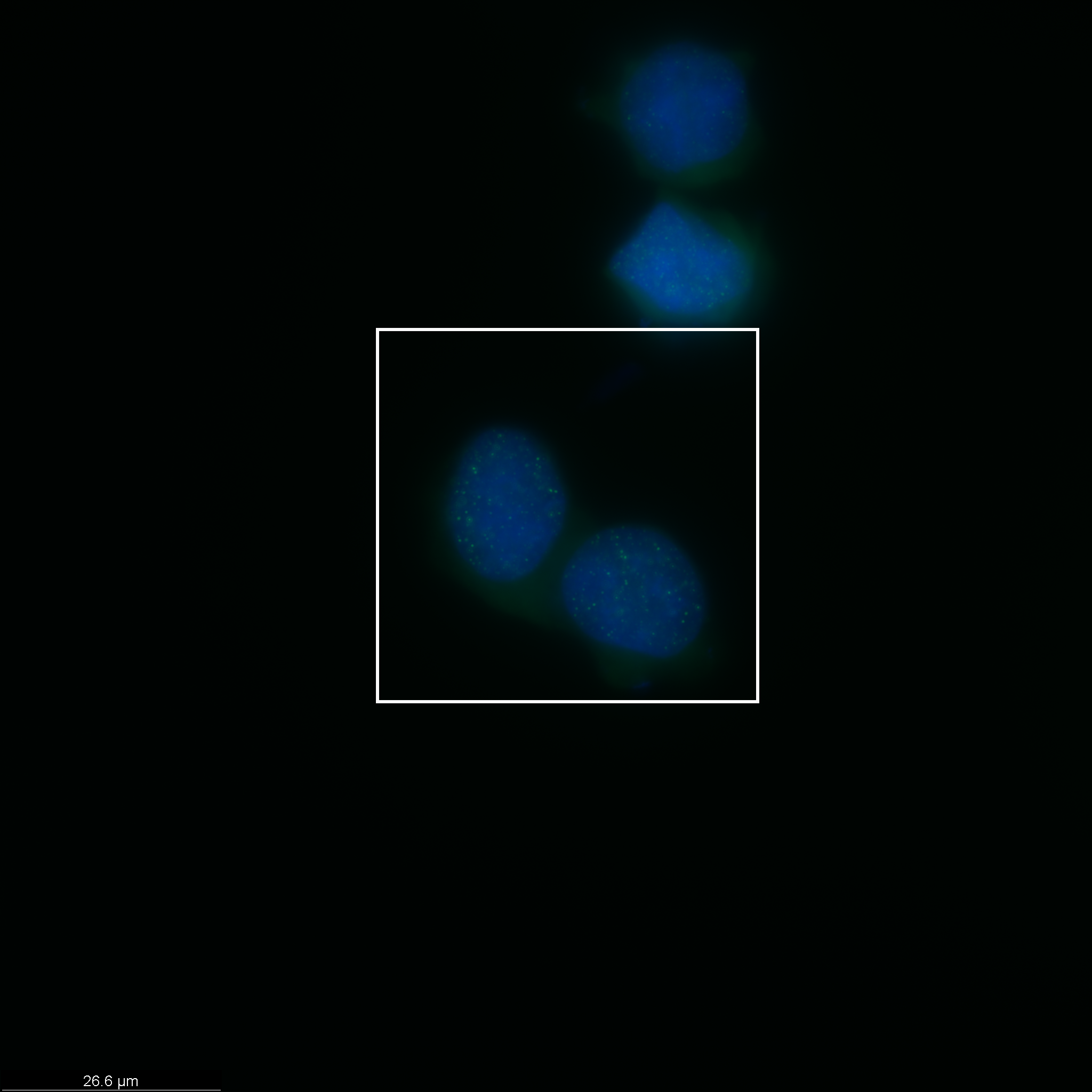

Supplement: Supplementary file 5 — Source data Fig. 4 [file 44318_2025_641_MOESM5_ESM.zip › EMBOJ-2025-120713R_SourceDataForFigure4/FIG 4E/3-RSMC KO+siNC/merge.tif]

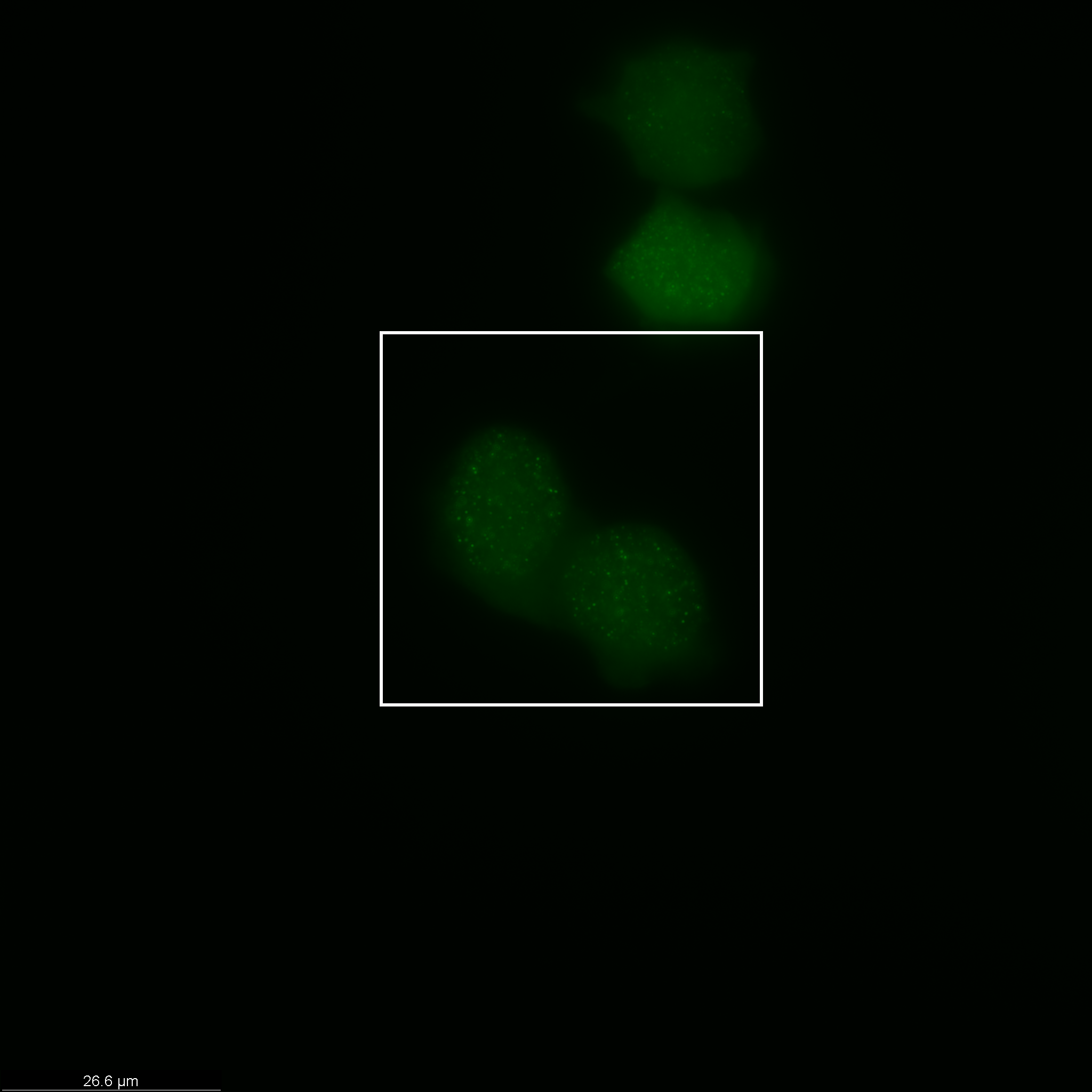

Supplement: Supplementary file 5 — Source data Fig. 4 [file 44318_2025_641_MOESM5_ESM.zip › EMBOJ-2025-120713R_SourceDataForFigure4/FIG 4E/3-RSMC KO+siNC/Sororin.tif]

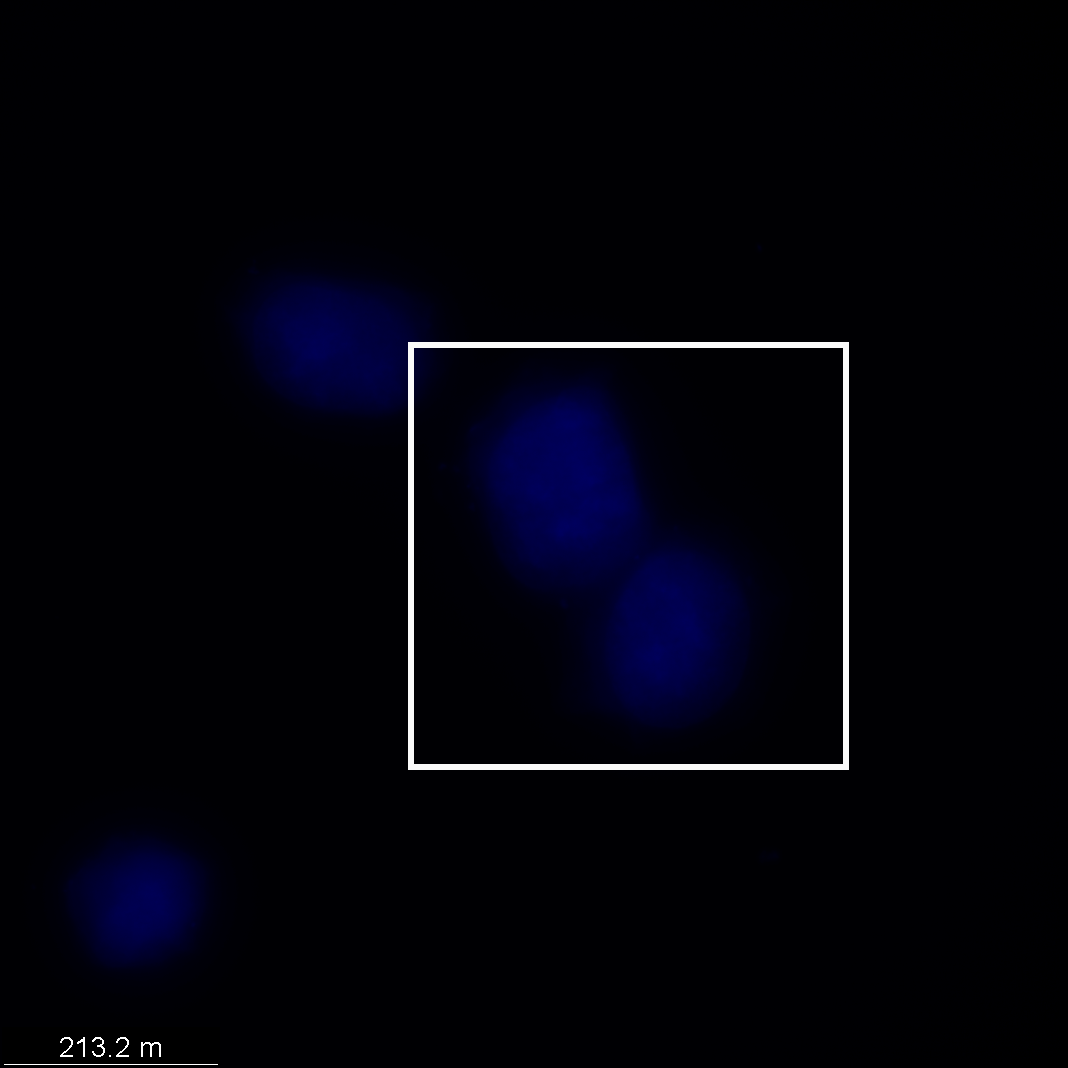

Supplement: Supplementary file 5 — Source data Fig. 4 [file 44318_2025_641_MOESM5_ESM.zip › EMBOJ-2025-120713R_SourceDataForFigure4/FIG 4E/4-RSMC KO+siESCO1&2/DAPI.tif]

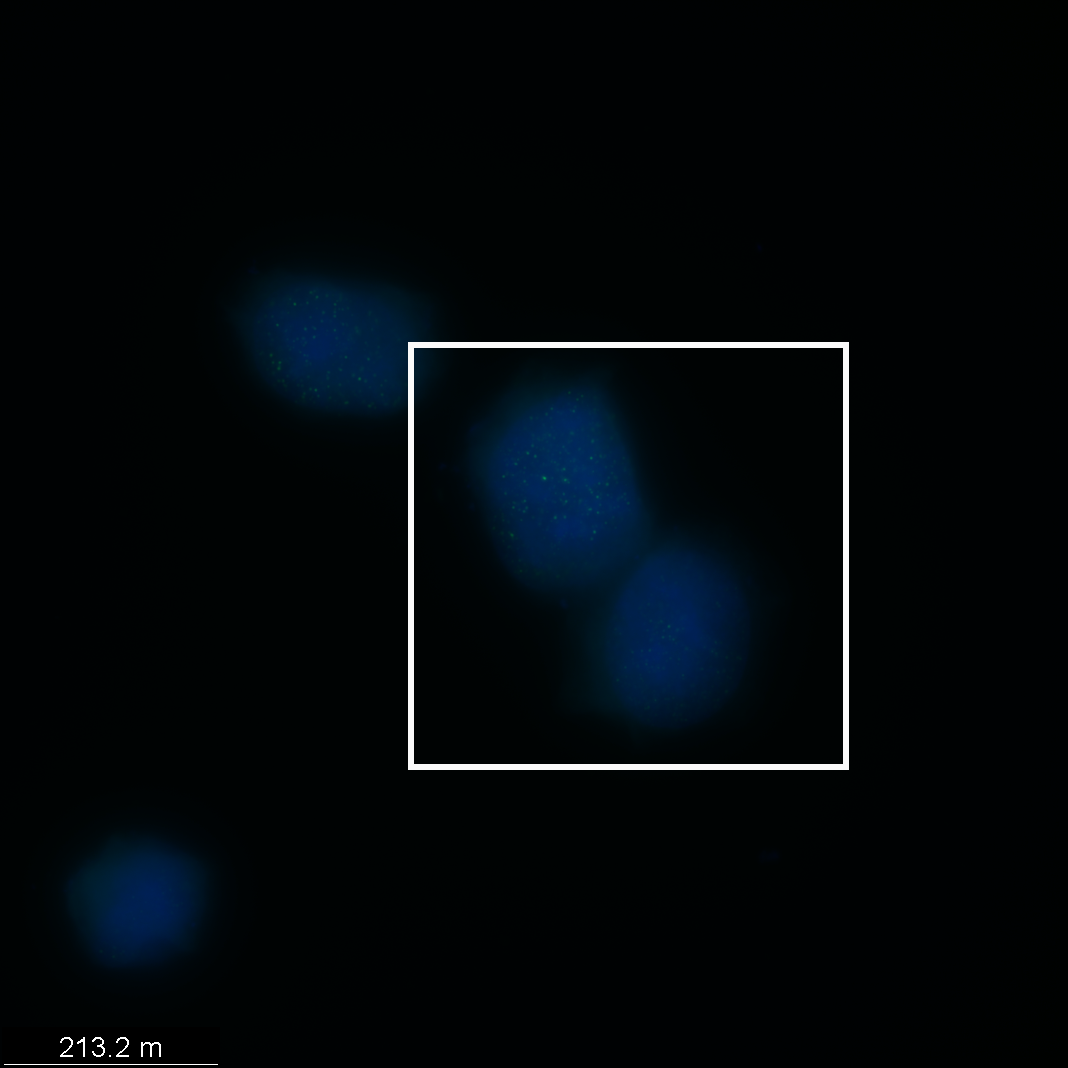

Supplement: Supplementary file 5 — Source data Fig. 4 [file 44318_2025_641_MOESM5_ESM.zip › EMBOJ-2025-120713R_SourceDataForFigure4/FIG 4E/4-RSMC KO+siESCO1&2/merge.tif]

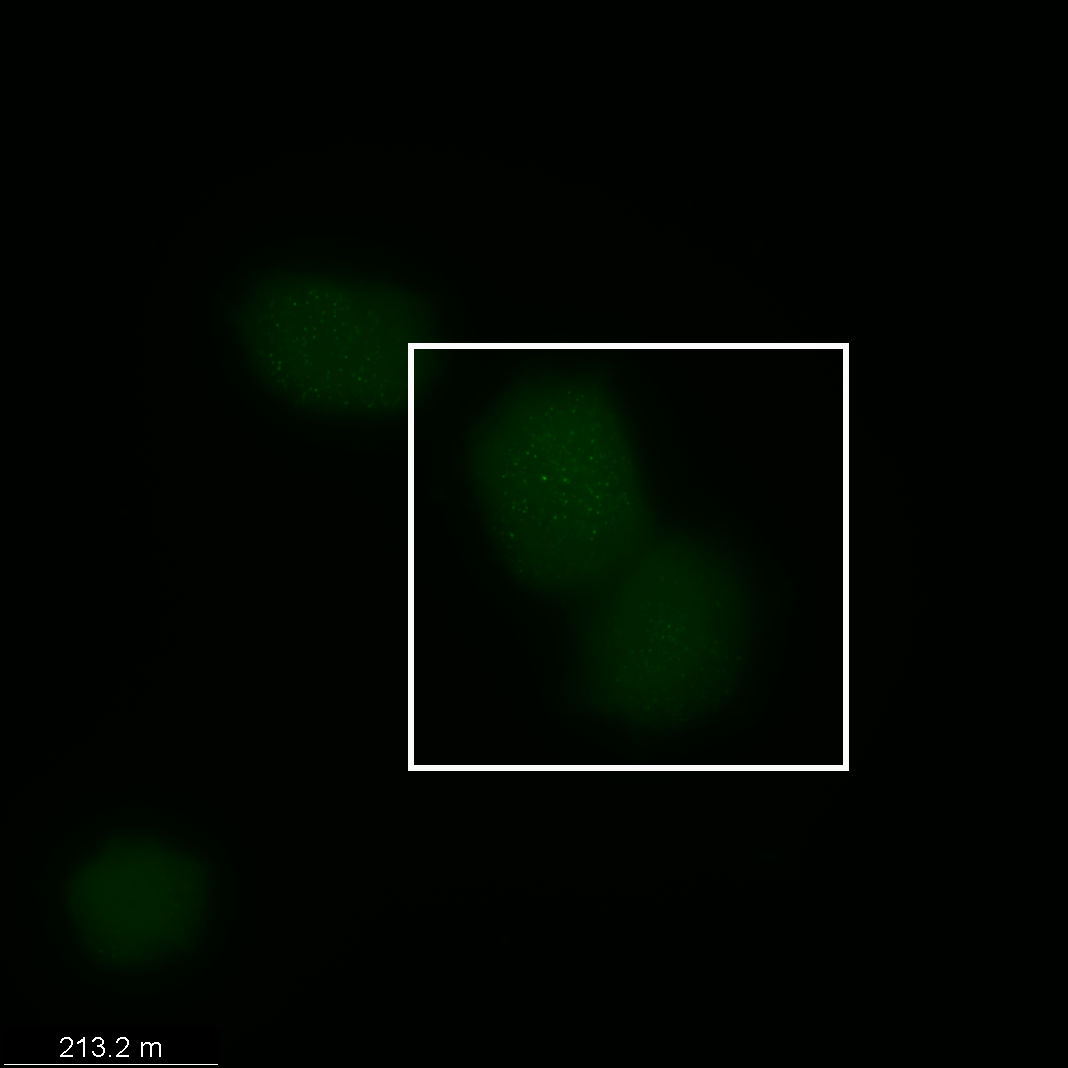

Supplement: Supplementary file 5 — Source data Fig. 4 [file 44318_2025_641_MOESM5_ESM.zip › EMBOJ-2025-120713R_SourceDataForFigure4/FIG 4E/4-RSMC KO+siESCO1&2/Sororin.tif]

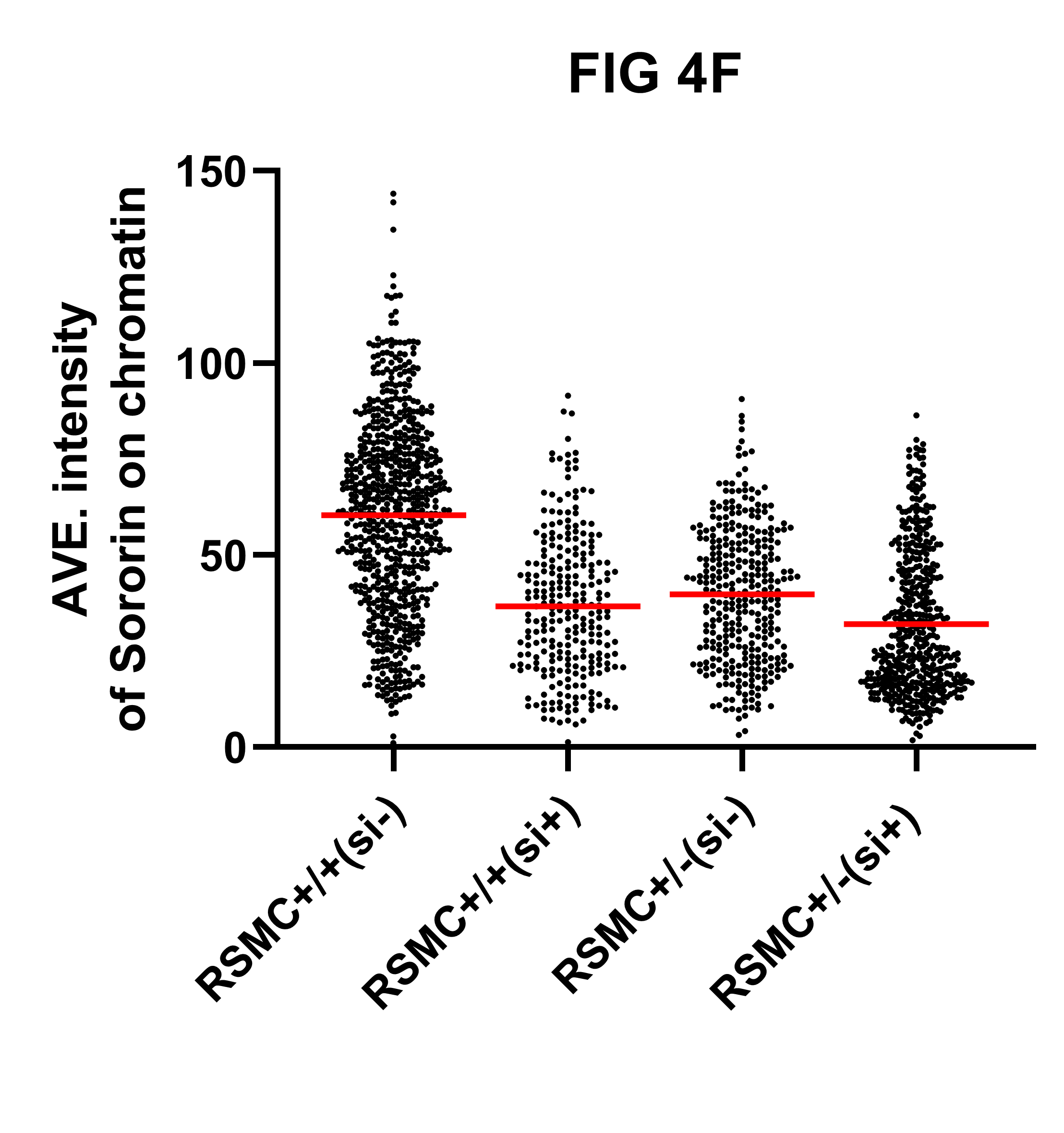

Supplement: Supplementary file 5 — Source data Fig. 4 [file 44318_2025_641_MOESM5_ESM.zip › EMBOJ-2025-120713R_SourceDataForFigure4/FIG 4F/FIG 4F before PS.tif]

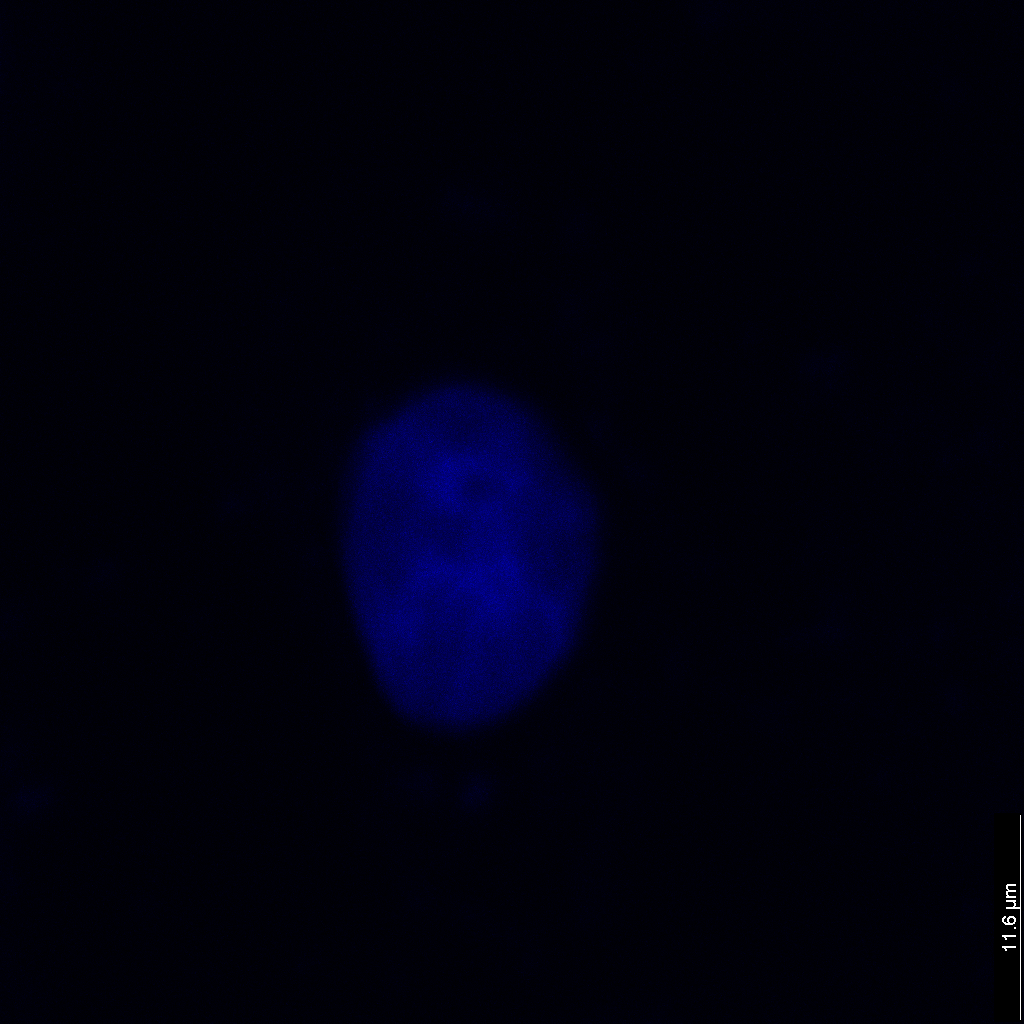

Supplement: Supplementary file 6 — Source data Fig. 5 [file 44318_2025_641_MOESM6_ESM.zip › EMBOJ-2025-120713R_SourceDataForFigure5/FIG 5B/G1-S DMSO/DAPI.tif]

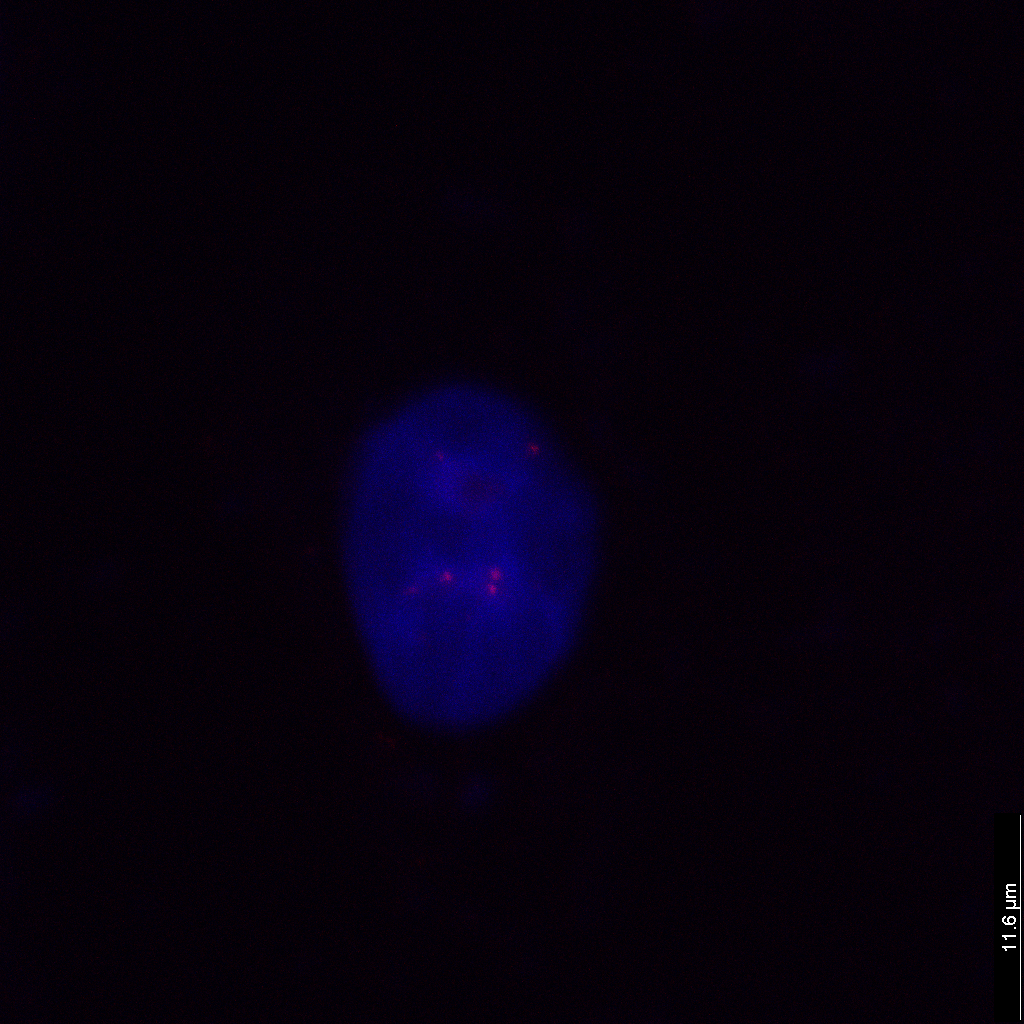

Supplement: Supplementary file 6 — Source data Fig. 5 [file 44318_2025_641_MOESM6_ESM.zip › EMBOJ-2025-120713R_SourceDataForFigure5/FIG 5B/G1-S DMSO/merge.tif]

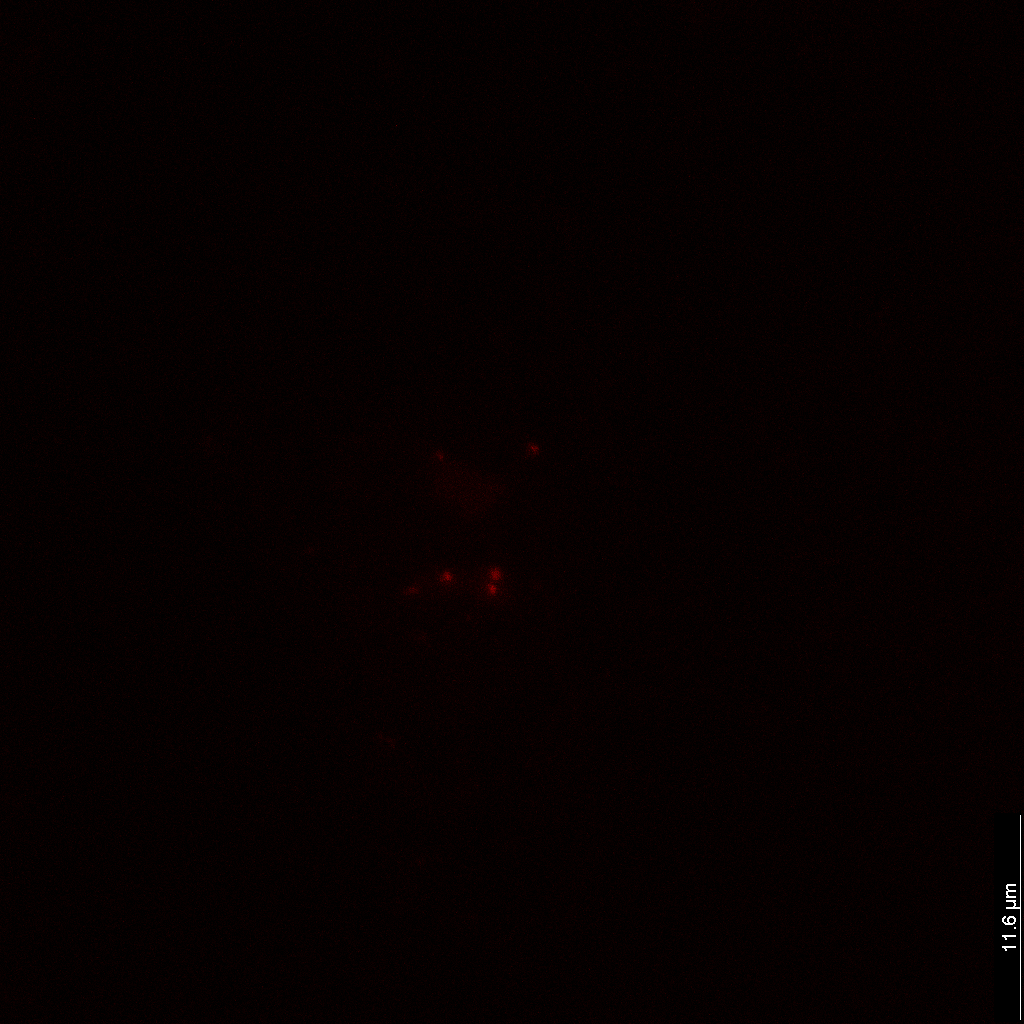

Supplement: Supplementary file 6 — Source data Fig. 5 [file 44318_2025_641_MOESM6_ESM.zip › EMBOJ-2025-120713R_SourceDataForFigure5/FIG 5B/G1-S DMSO/PLA.tif]

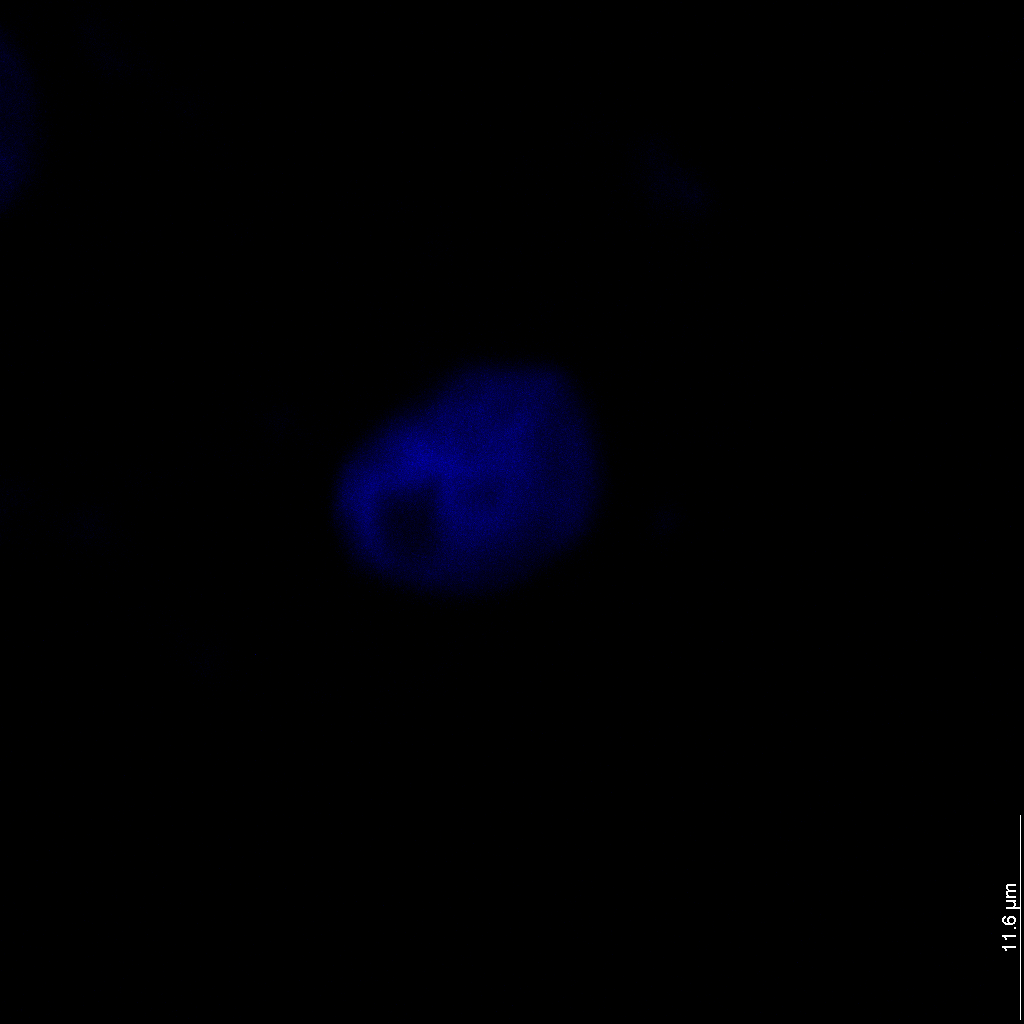

Supplement: Supplementary file 6 — Source data Fig. 5 [file 44318_2025_641_MOESM6_ESM.zip › EMBOJ-2025-120713R_SourceDataForFigure5/FIG 5B/G1-S Olaparib/DAPI.tif]

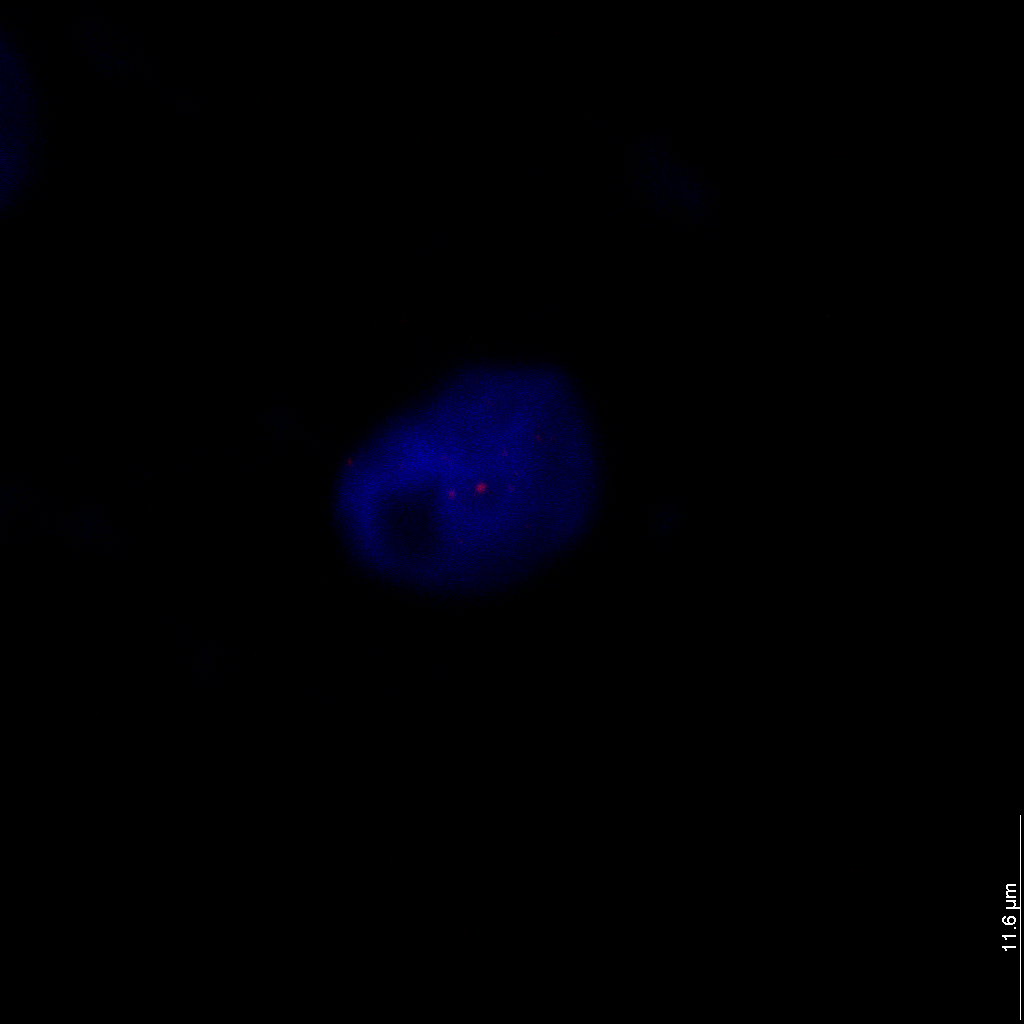

Supplement: Supplementary file 6 — Source data Fig. 5 [file 44318_2025_641_MOESM6_ESM.zip › EMBOJ-2025-120713R_SourceDataForFigure5/FIG 5B/G1-S Olaparib/merge.tif]

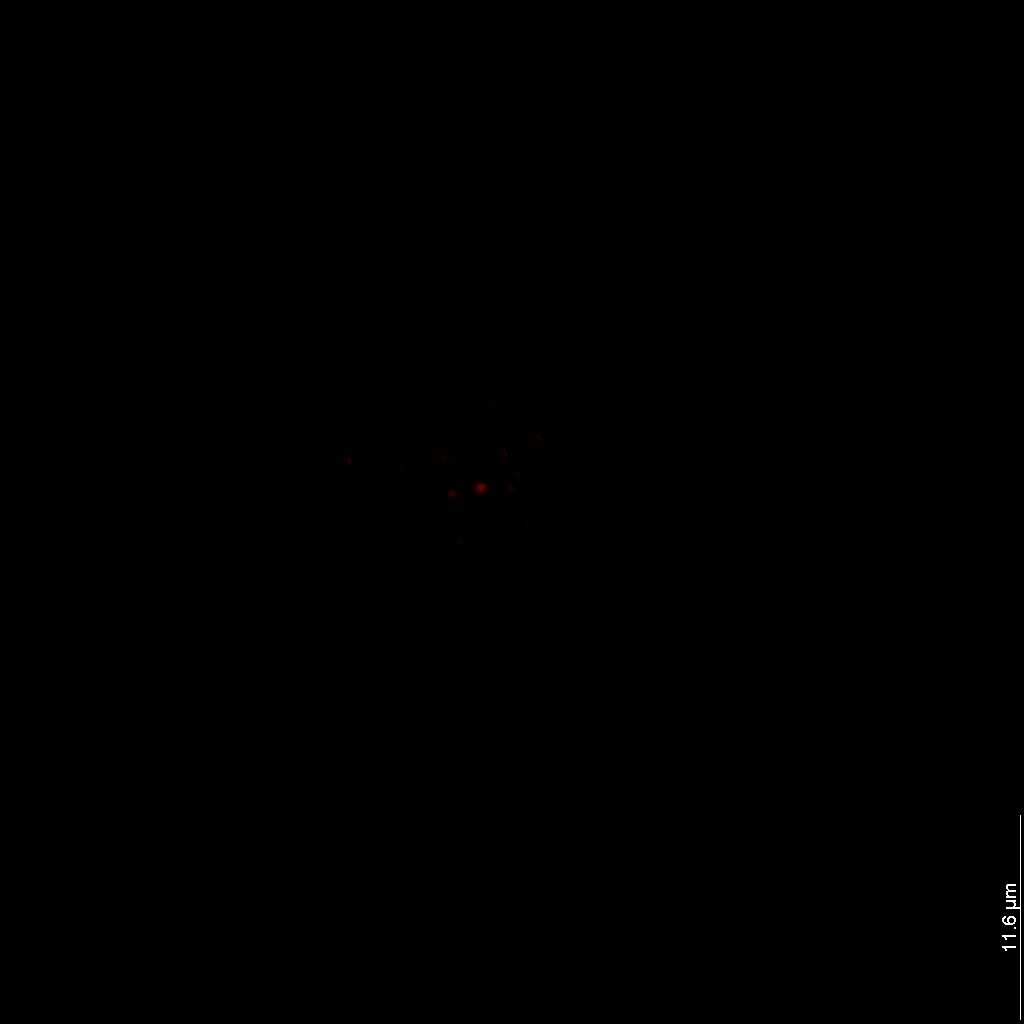

Supplement: Supplementary file 6 — Source data Fig. 5 [file 44318_2025_641_MOESM6_ESM.zip › EMBOJ-2025-120713R_SourceDataForFigure5/FIG 5B/G1-S Olaparib/PLA.tif]

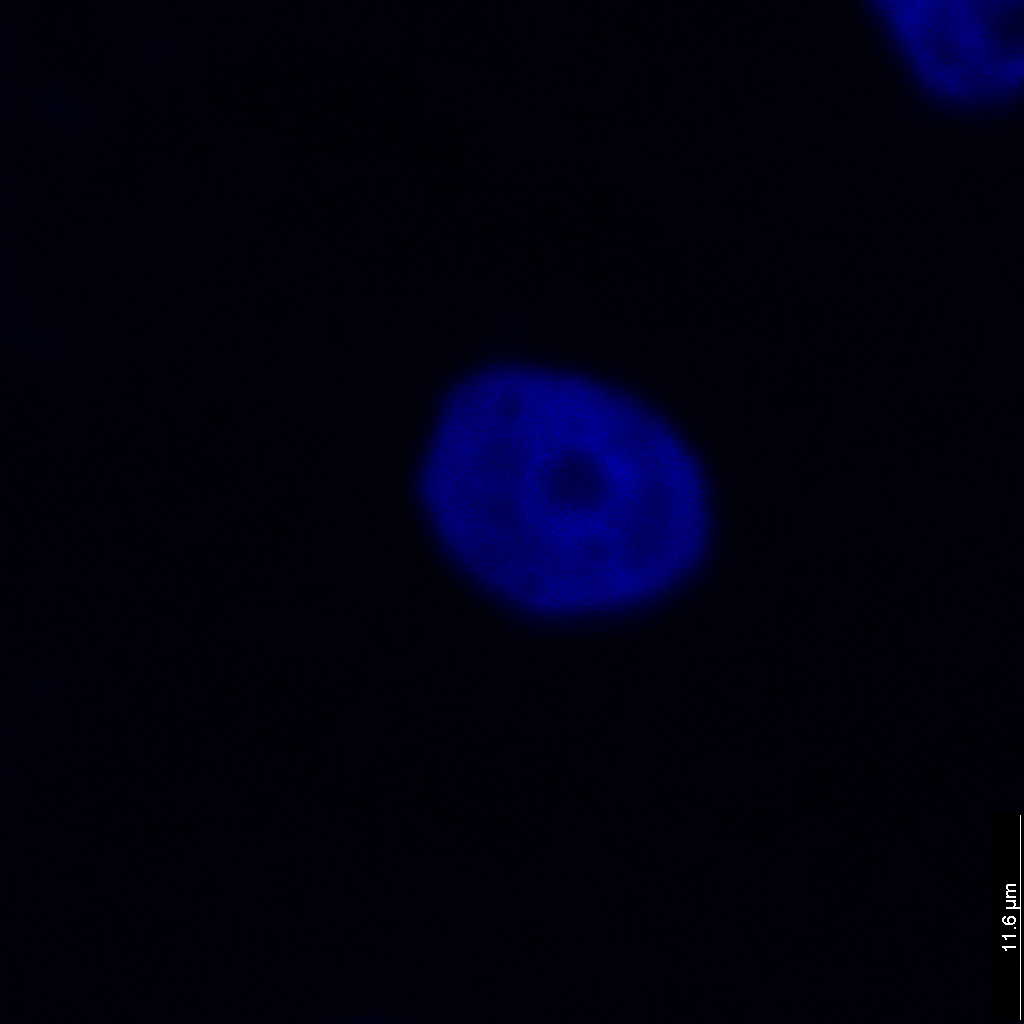

Supplement: Supplementary file 6 — Source data Fig. 5 [file 44318_2025_641_MOESM6_ESM.zip › EMBOJ-2025-120713R_SourceDataForFigure5/FIG 5B/Mid S DMSO/DAPI.tif]

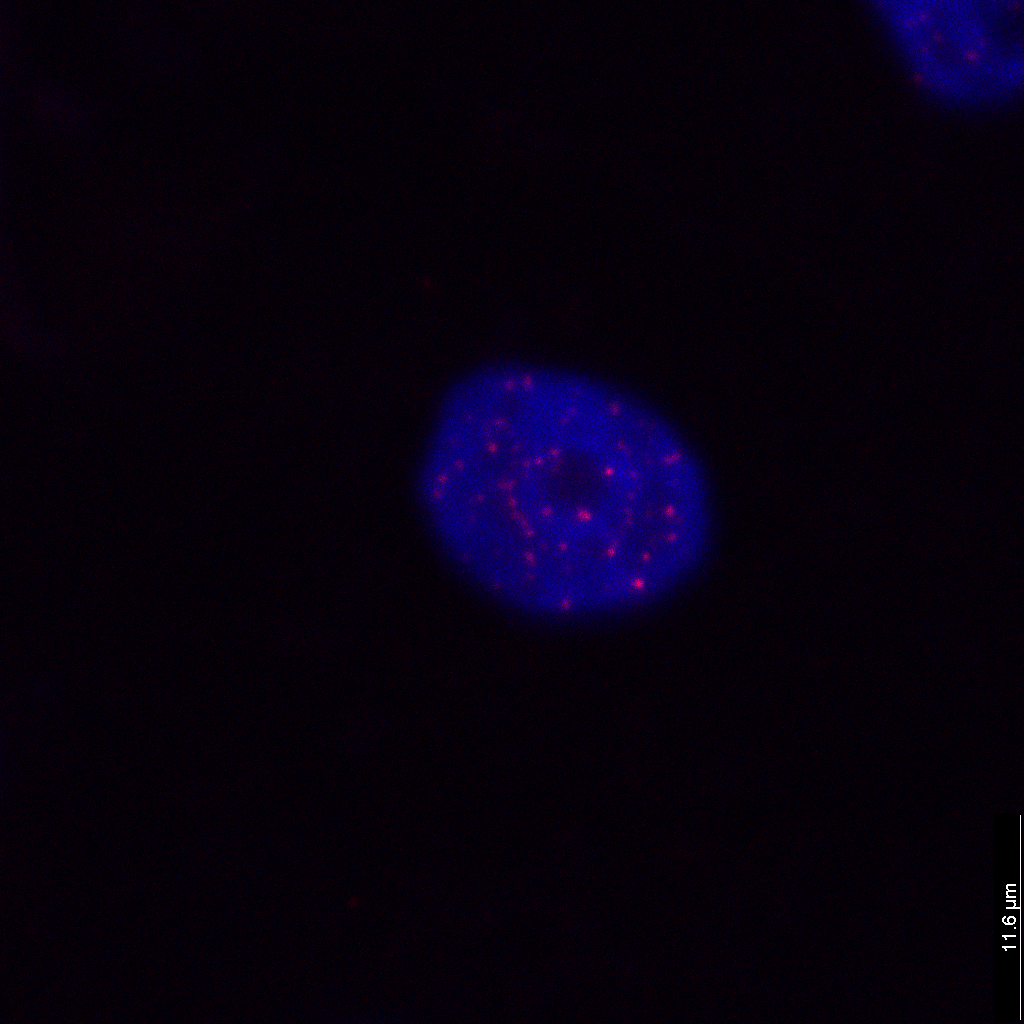

Supplement: Supplementary file 6 — Source data Fig. 5 [file 44318_2025_641_MOESM6_ESM.zip › EMBOJ-2025-120713R_SourceDataForFigure5/FIG 5B/Mid S DMSO/merge.tif]
