# Supplementary figures and images for: S-phase PARylation of microprotein RSMC enhances the function of Sororin in sister chromatid cohesion (part 2 of 3)
Source: EMBO J. 2025 Nov 19;45(1):278–309. doi: 10.1038/s44318-025-00641-8 (PMC12759081; doi:10.1038/s44318-025-00641-8)

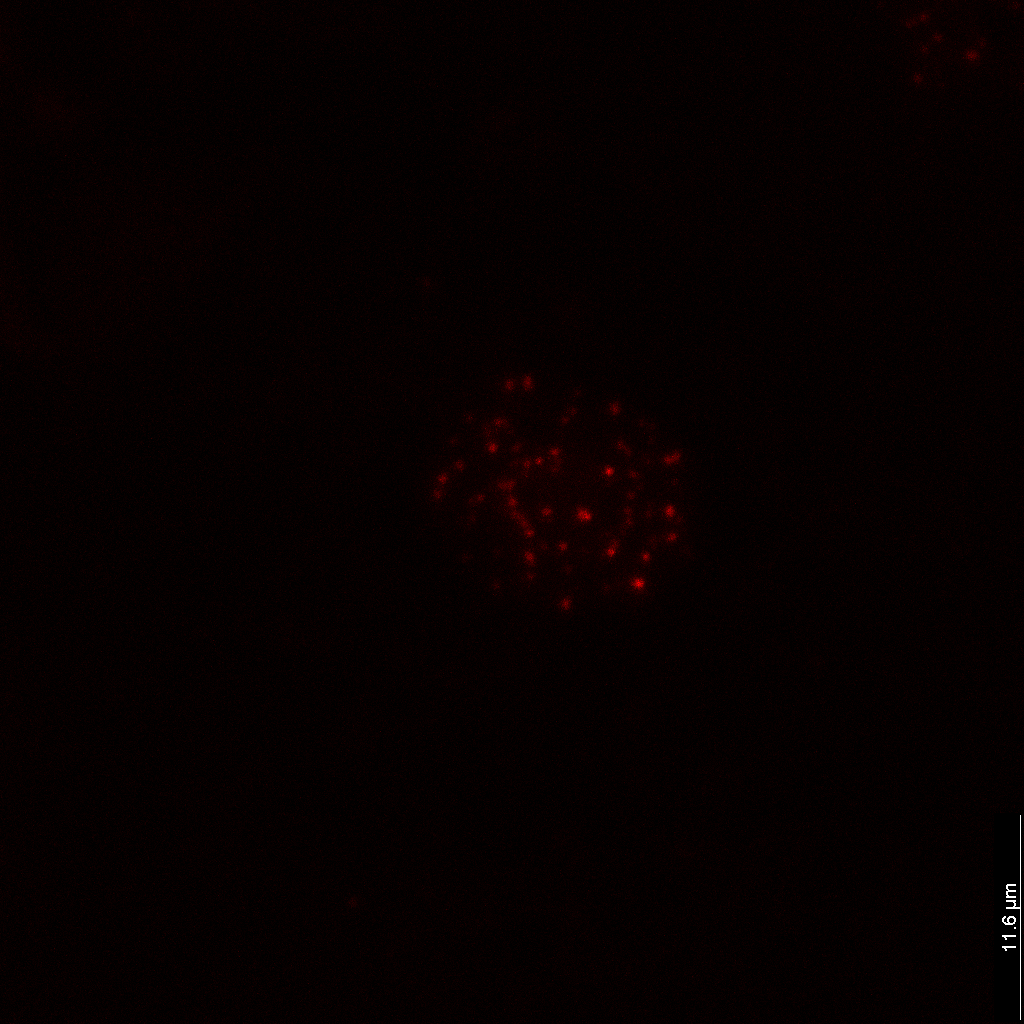

Supplement: Supplementary file 6 — Source data Fig. 5 [file 44318_2025_641_MOESM6_ESM.zip › EMBOJ-2025-120713R_SourceDataForFigure5/FIG 5B/Mid S DMSO/PLA.tif]

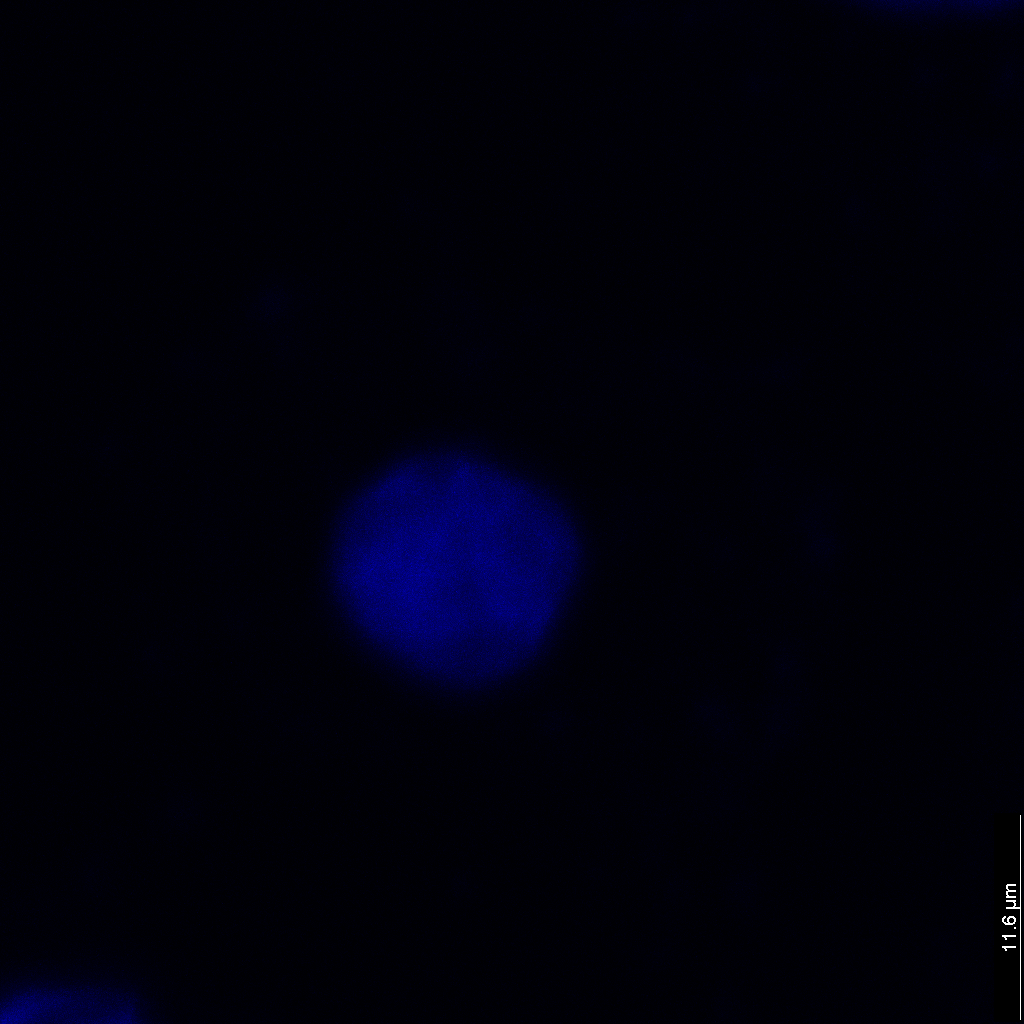

Supplement: Supplementary file 6 — Source data Fig. 5 [file 44318_2025_641_MOESM6_ESM.zip › EMBOJ-2025-120713R_SourceDataForFigure5/FIG 5B/Mid S Olaparib/DAPI.tif]

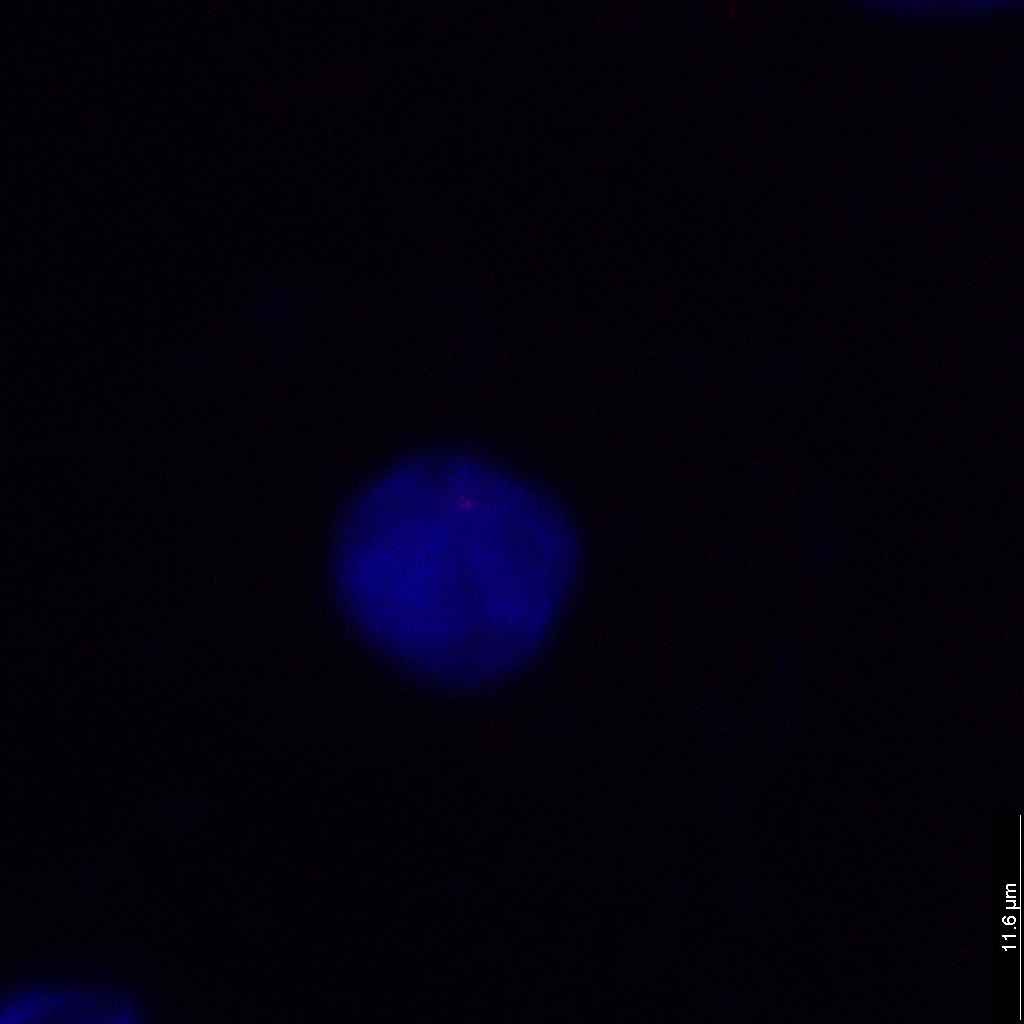

Supplement: Supplementary file 6 — Source data Fig. 5 [file 44318_2025_641_MOESM6_ESM.zip › EMBOJ-2025-120713R_SourceDataForFigure5/FIG 5B/Mid S Olaparib/merge.tif]

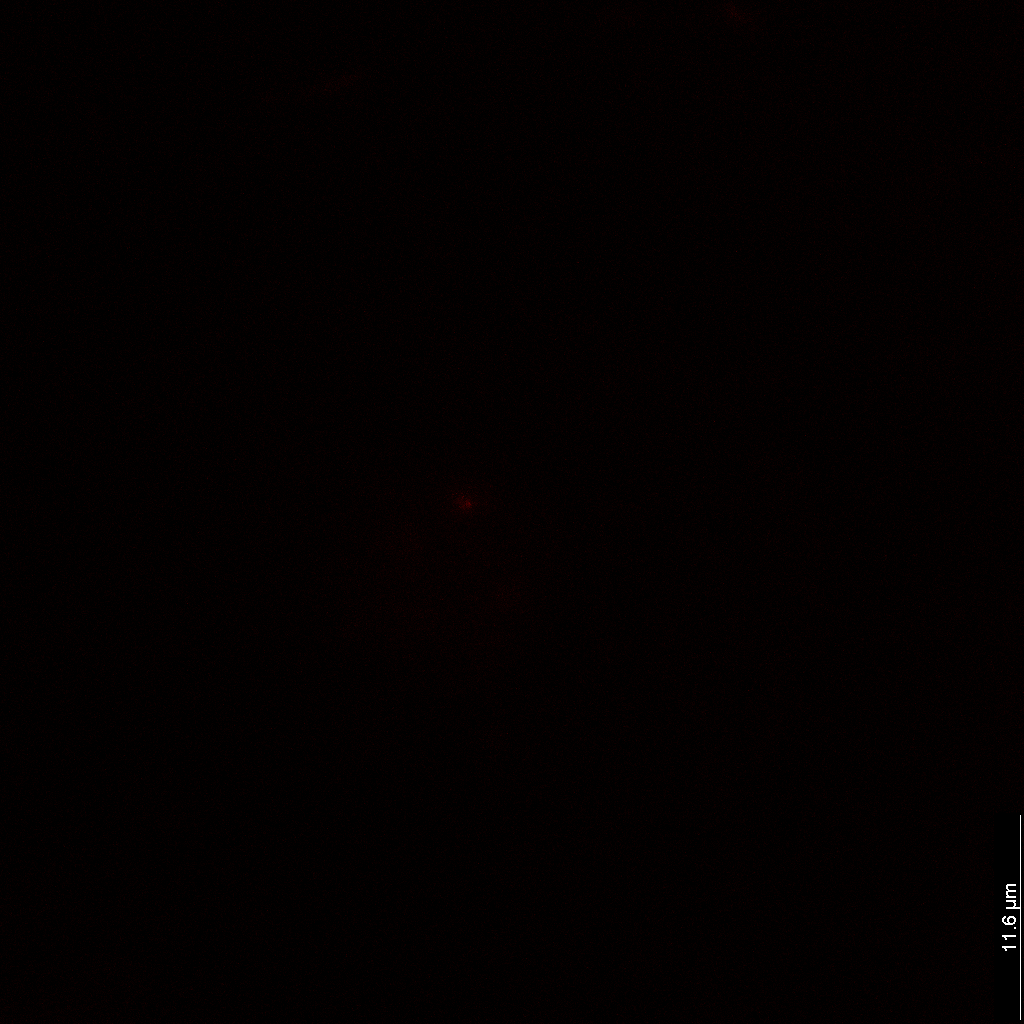

Supplement: Supplementary file 6 — Source data Fig. 5 [file 44318_2025_641_MOESM6_ESM.zip › EMBOJ-2025-120713R_SourceDataForFigure5/FIG 5B/Mid S Olaparib/PLA.tif]

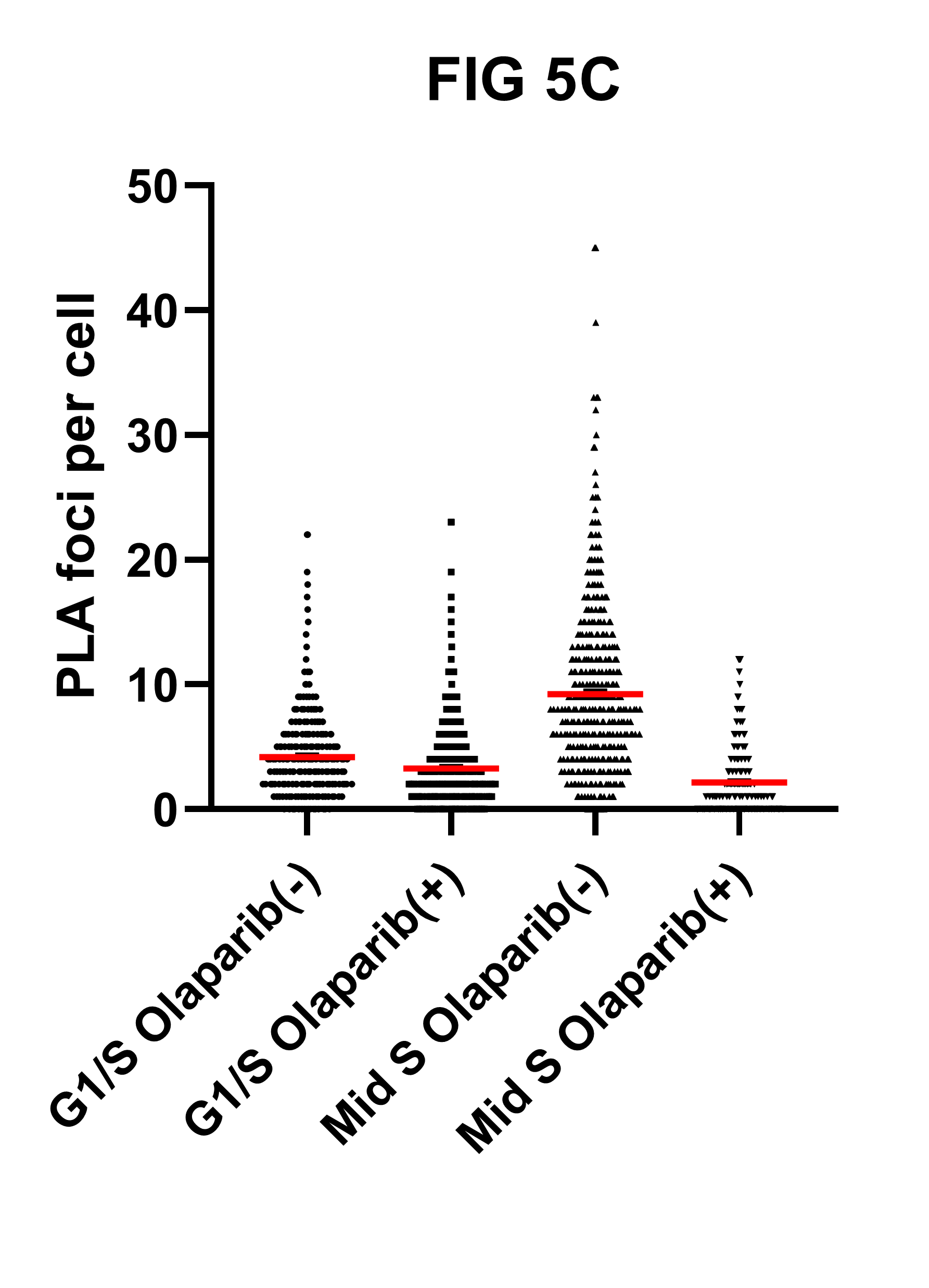

Supplement: Supplementary file 6 — Source data Fig. 5 [file 44318_2025_641_MOESM6_ESM.zip › EMBOJ-2025-120713R_SourceDataForFigure5/FIG 5C/FIG 5C before PS.tif]

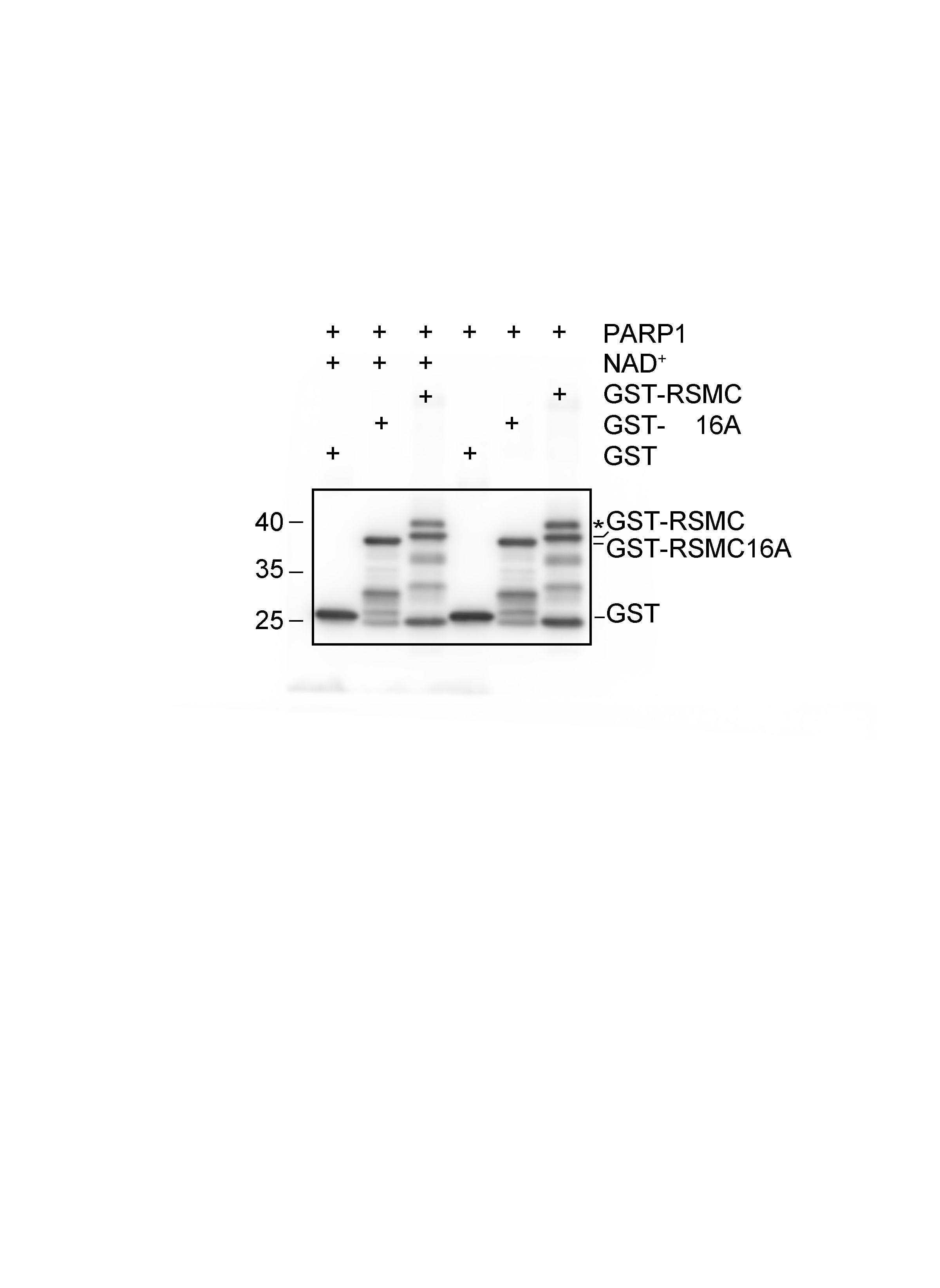

Supplement: Supplementary file 6 — Source data Fig. 5 [file 44318_2025_641_MOESM6_ESM.zip › EMBOJ-2025-120713R_SourceDataForFigure5/FIG 5D/GST-RSMC-rawdata.tif]

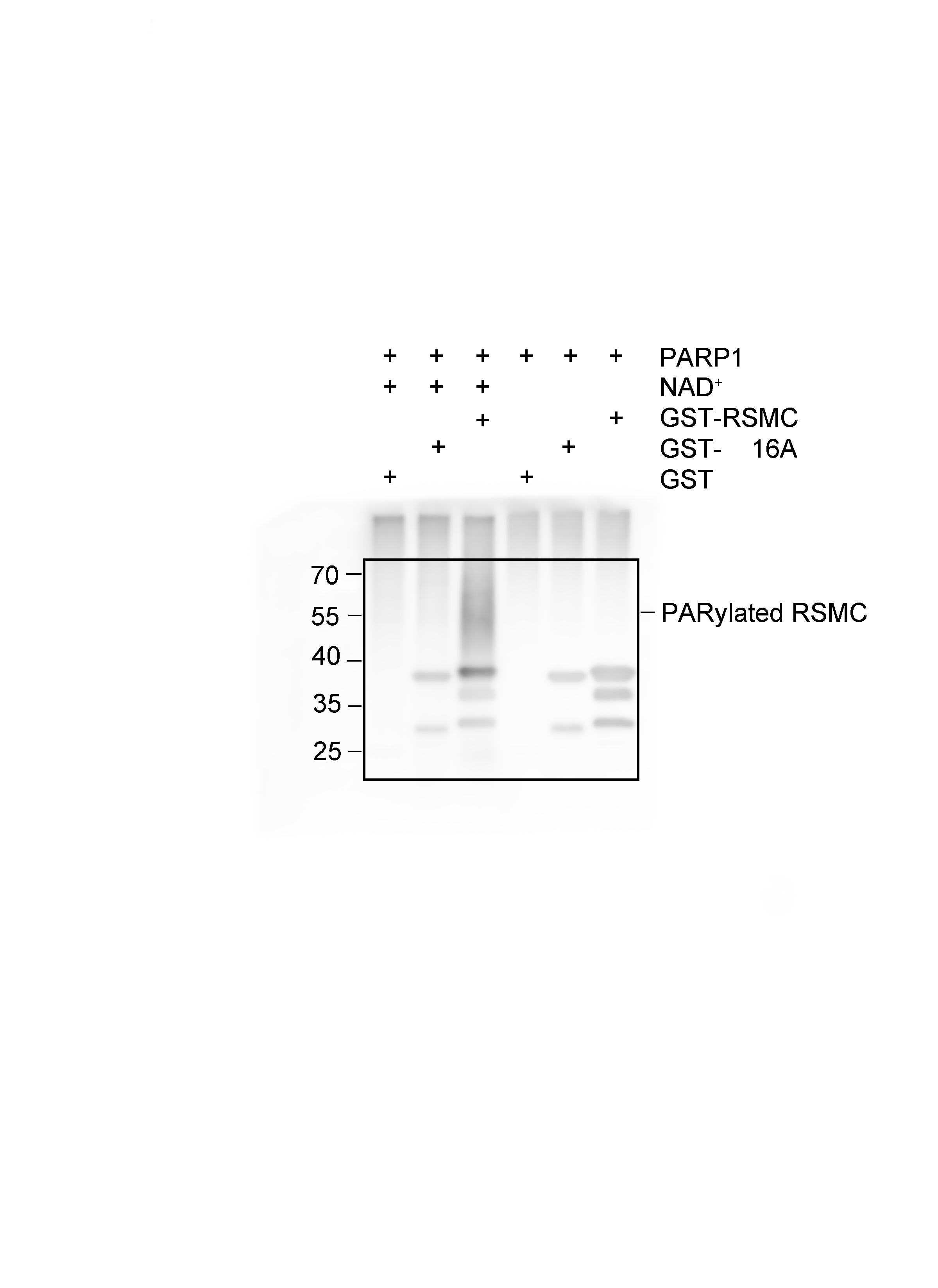

Supplement: Supplementary file 6 — Source data Fig. 5 [file 44318_2025_641_MOESM6_ESM.zip › EMBOJ-2025-120713R_SourceDataForFigure5/FIG 5D/PARylated RSMC-rawdata.tif]

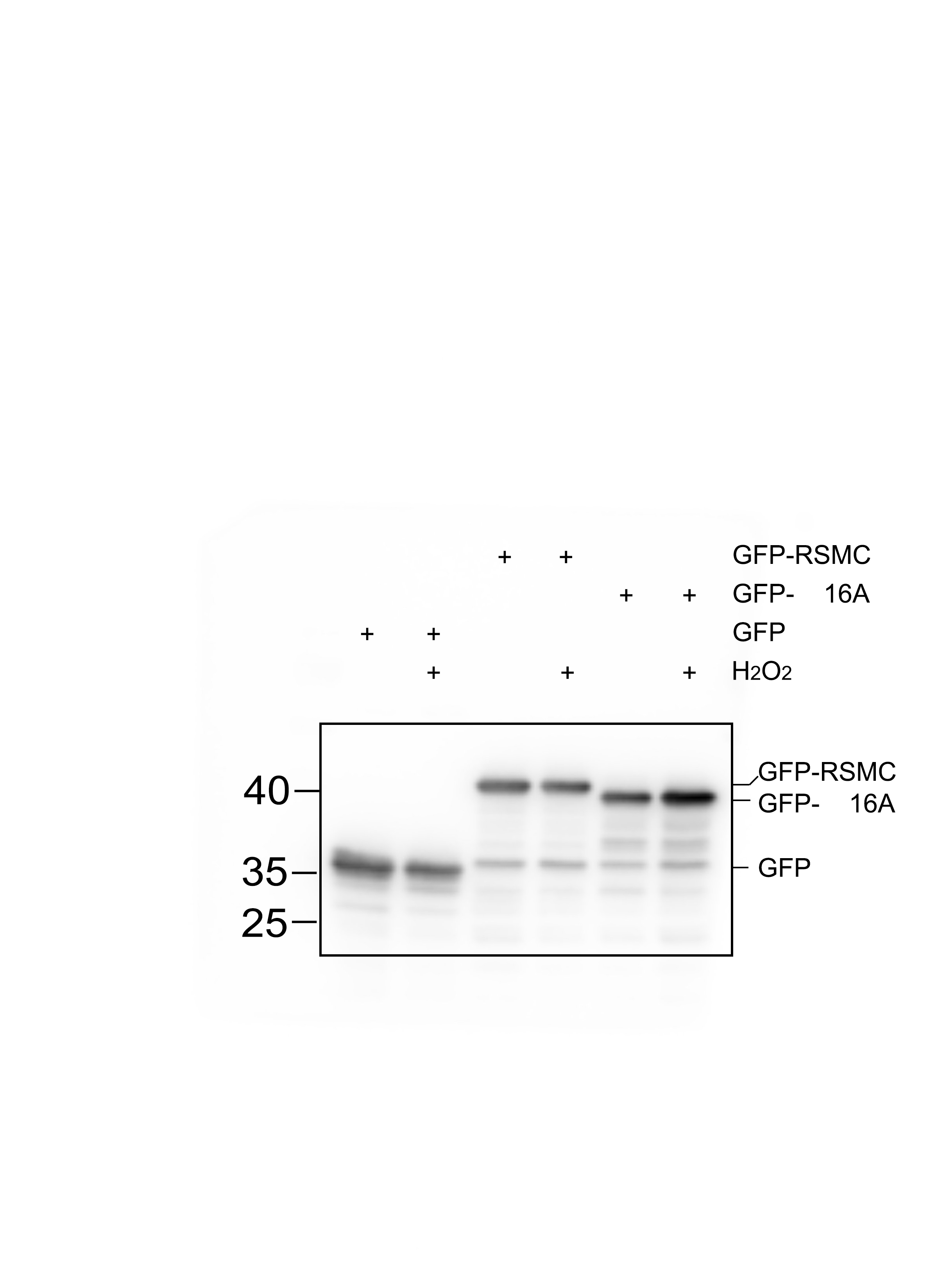

Supplement: Supplementary file 6 — Source data Fig. 5 [file 44318_2025_641_MOESM6_ESM.zip › EMBOJ-2025-120713R_SourceDataForFigure5/FIG 5E/GFP-RSMC.tif]

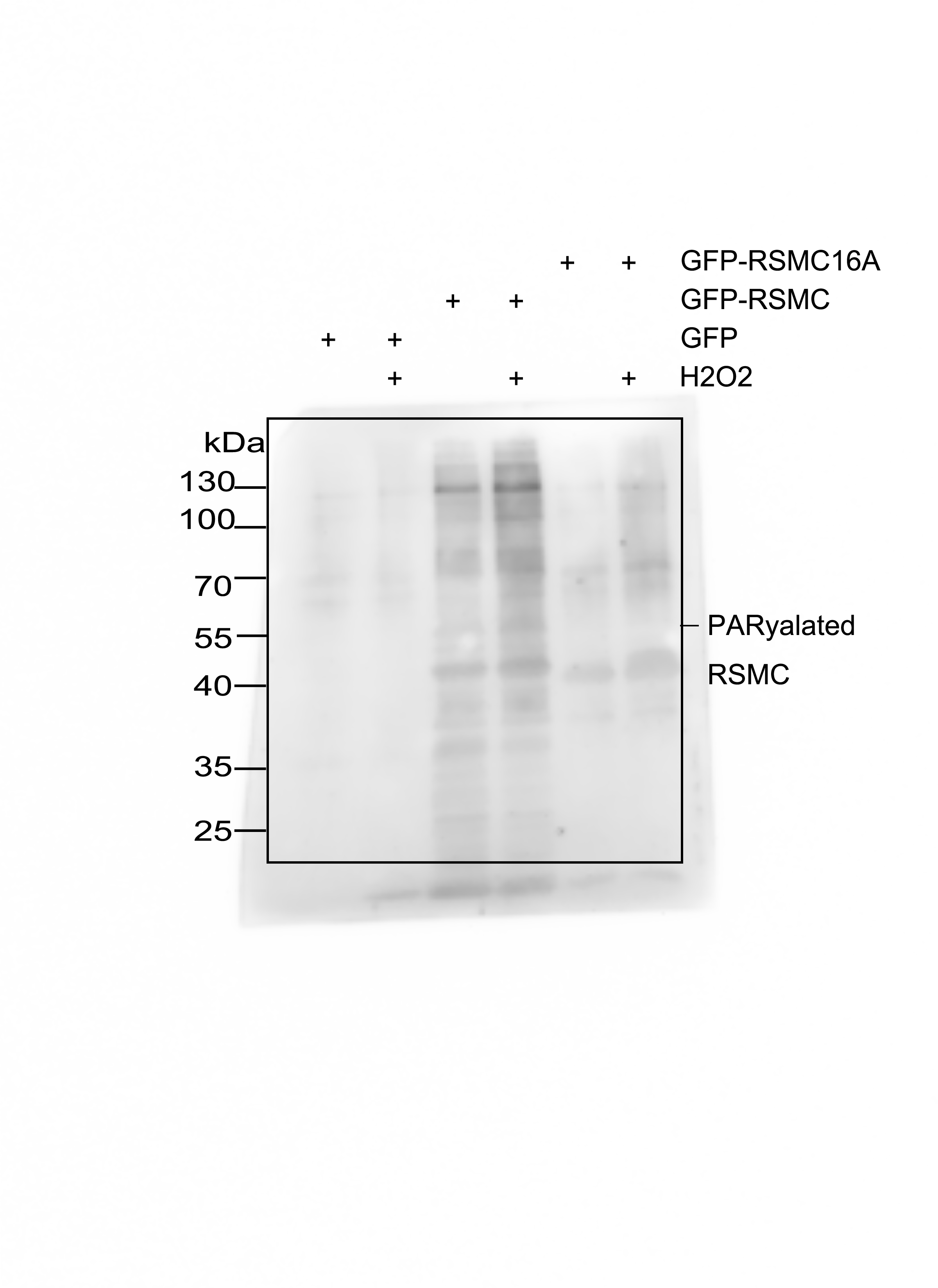

Supplement: Supplementary file 6 — Source data Fig. 5 [file 44318_2025_641_MOESM6_ESM.zip › EMBOJ-2025-120713R_SourceDataForFigure5/FIG 5E/PARylated RSMC.tif]

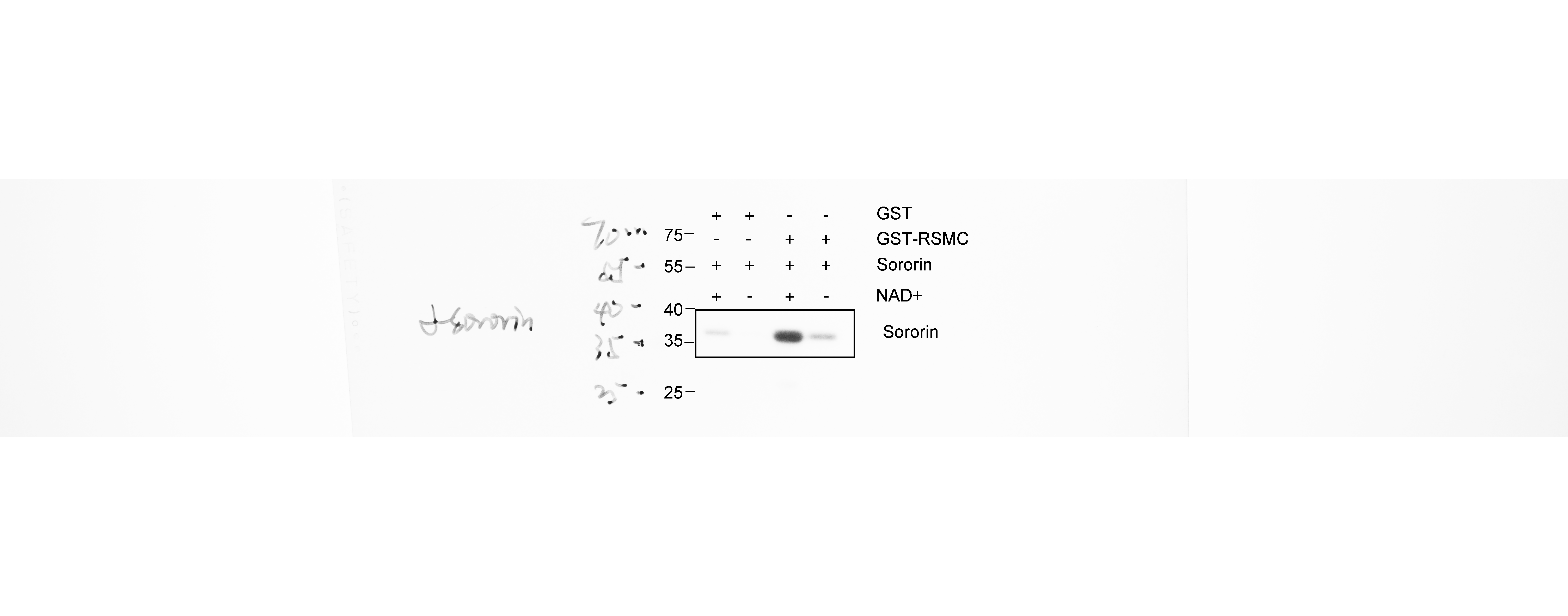

Supplement: Supplementary file 6 — Source data Fig. 5 [file 44318_2025_641_MOESM6_ESM.zip › EMBOJ-2025-120713R_SourceDataForFigure5/FIG 5F/pull down sororin SourceData.tif]

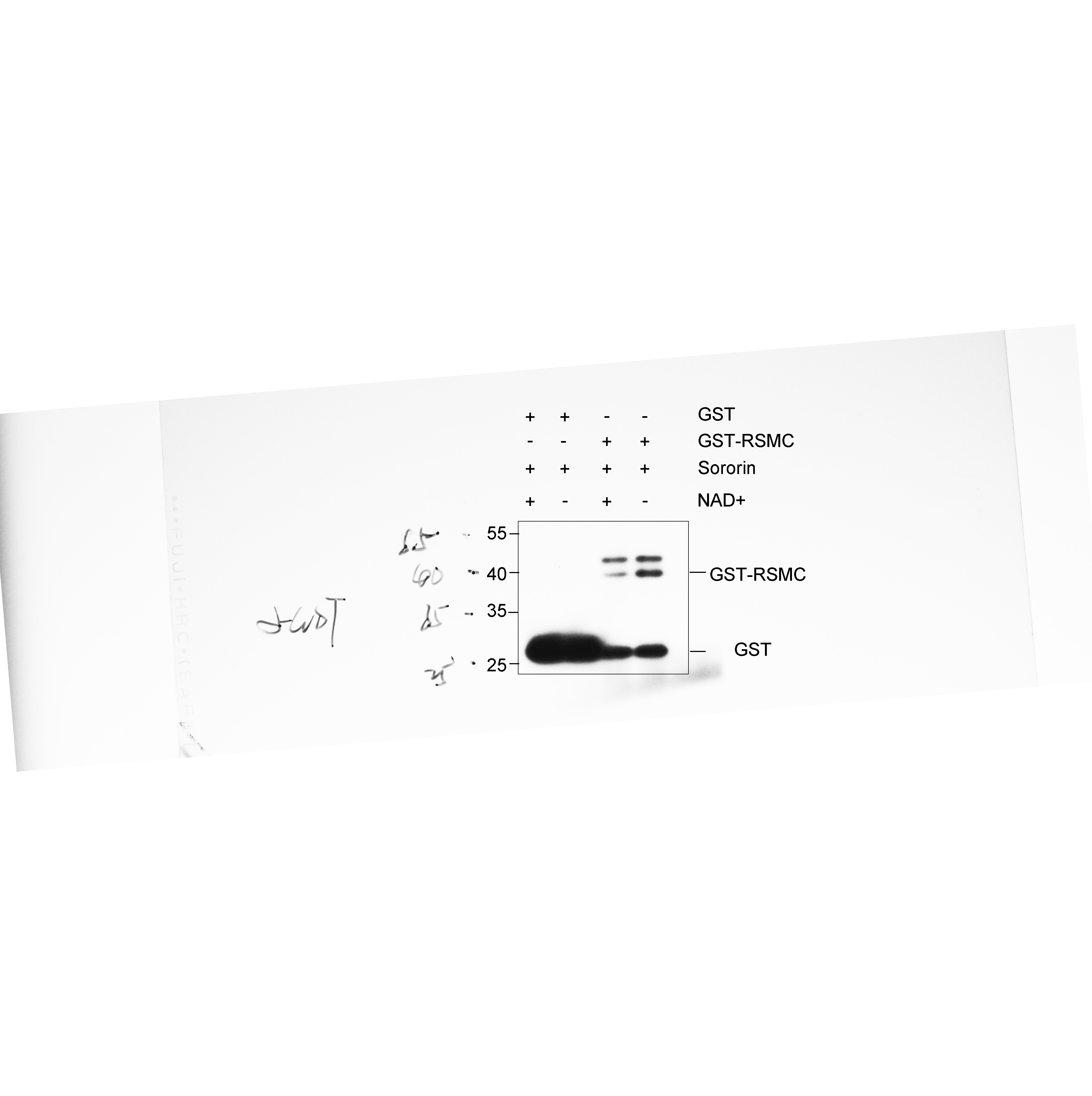

Supplement: Supplementary file 6 — Source data Fig. 5 [file 44318_2025_641_MOESM6_ESM.zip › EMBOJ-2025-120713R_SourceDataForFigure5/FIG 5F/RSMC SourceData.tif]

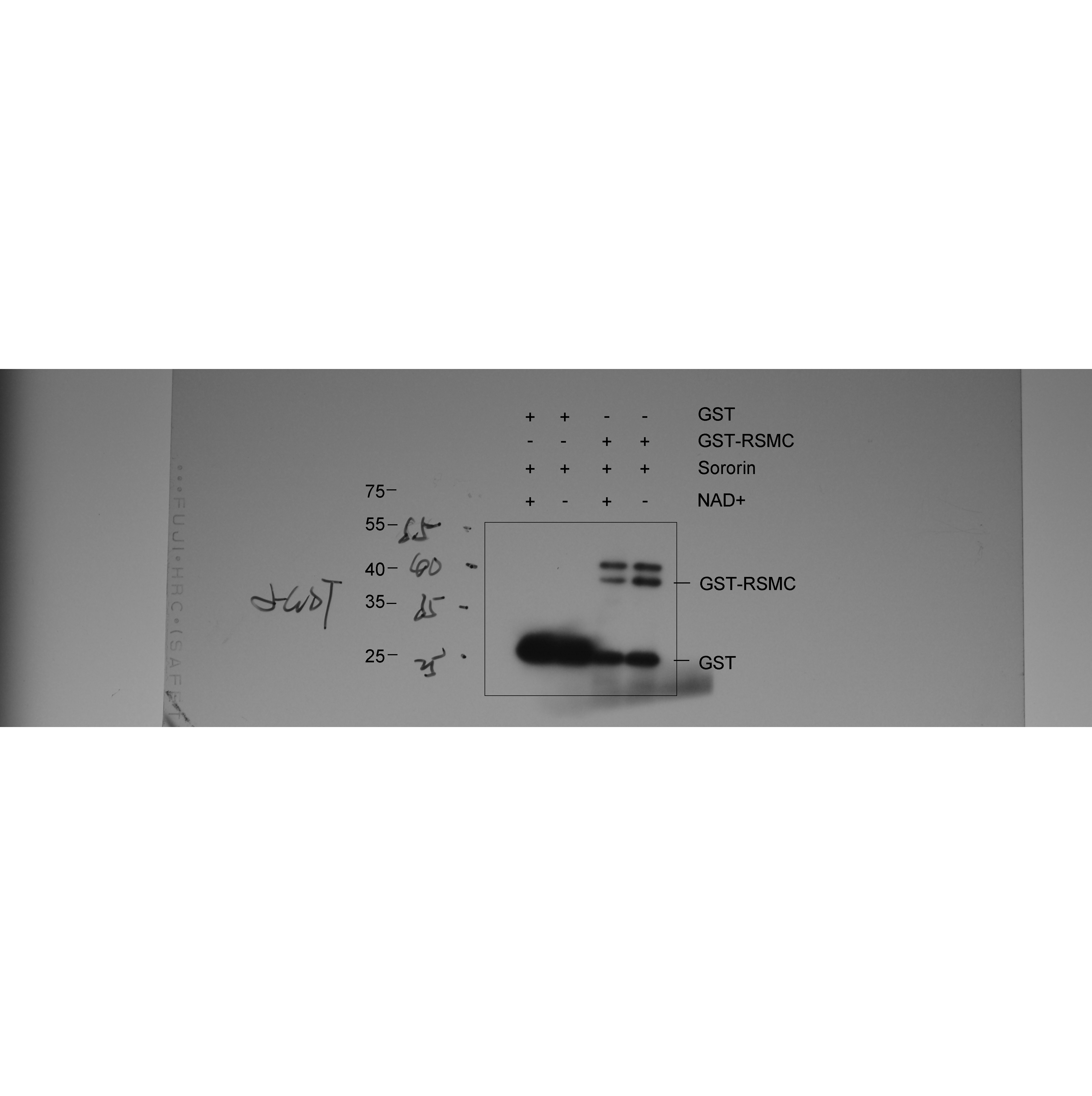

Supplement: Supplementary file 6 — Source data Fig. 5 [file 44318_2025_641_MOESM6_ESM.zip › EMBOJ-2025-120713R_SourceDataForFigure5/FIG 5G/exp2/GST-RSMC SourceData.tif]

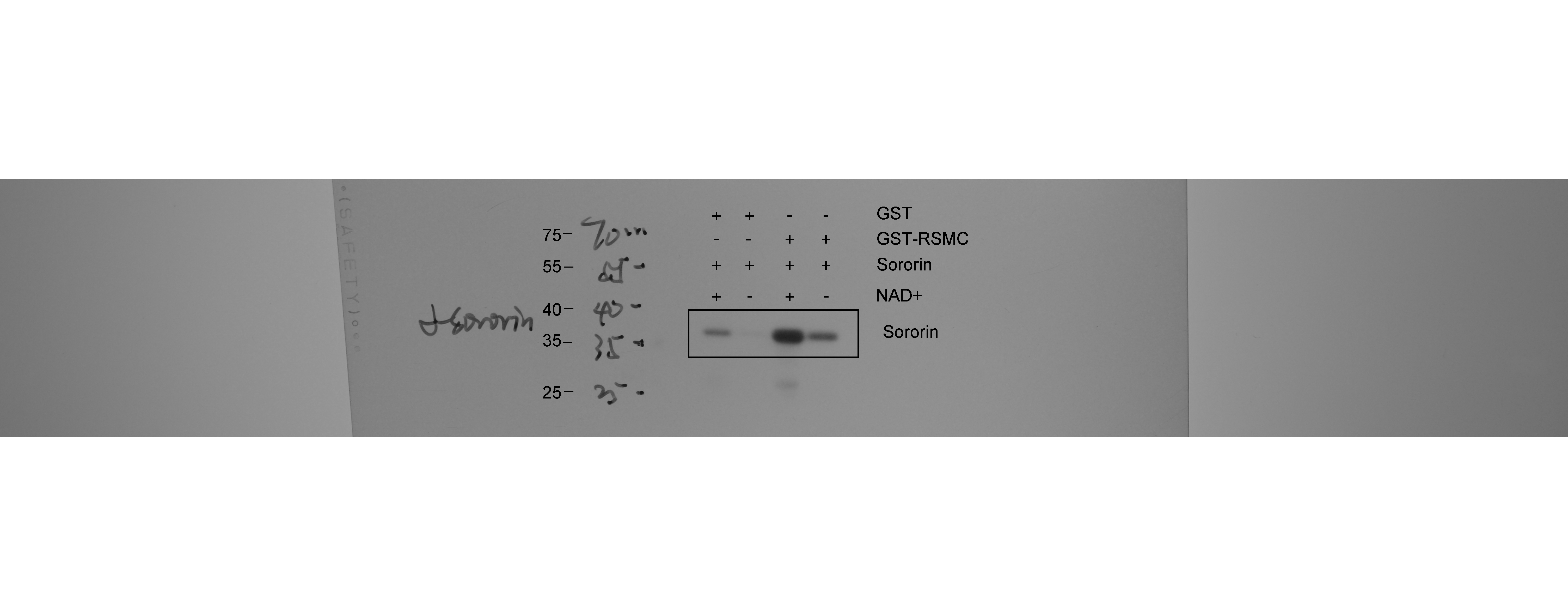

Supplement: Supplementary file 6 — Source data Fig. 5 [file 44318_2025_641_MOESM6_ESM.zip › EMBOJ-2025-120713R_SourceDataForFigure5/FIG 5G/exp2/pull down sororin SourceData.tif]

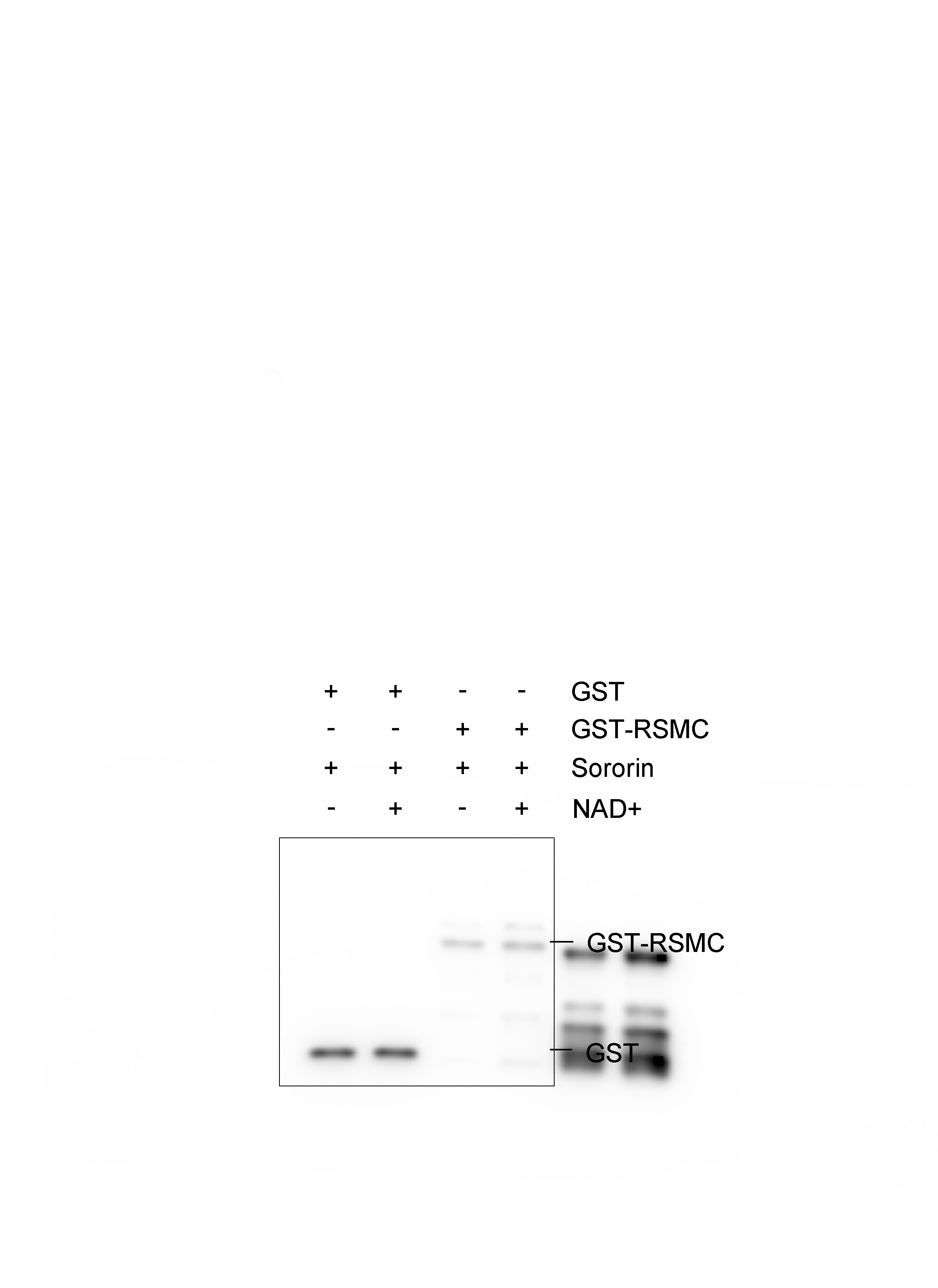

Supplement: Supplementary file 6 — Source data Fig. 5 [file 44318_2025_641_MOESM6_ESM.zip › EMBOJ-2025-120713R_SourceDataForFigure5/FIG 5G/exp3/GST-RSMC SourceData.tif]

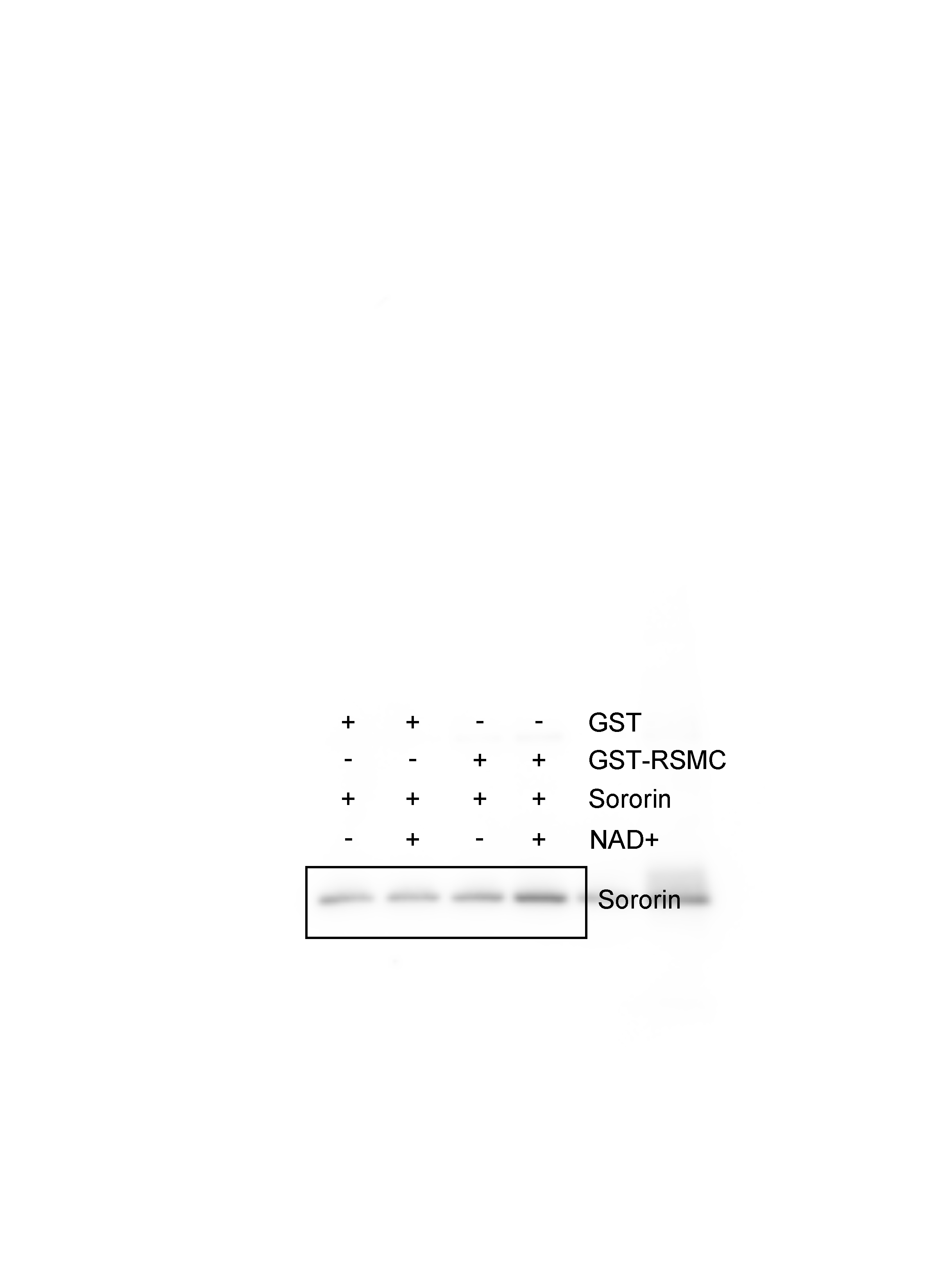

Supplement: Supplementary file 6 — Source data Fig. 5 [file 44318_2025_641_MOESM6_ESM.zip › EMBOJ-2025-120713R_SourceDataForFigure5/FIG 5G/exp3/pull down sororin SourceData.tif]

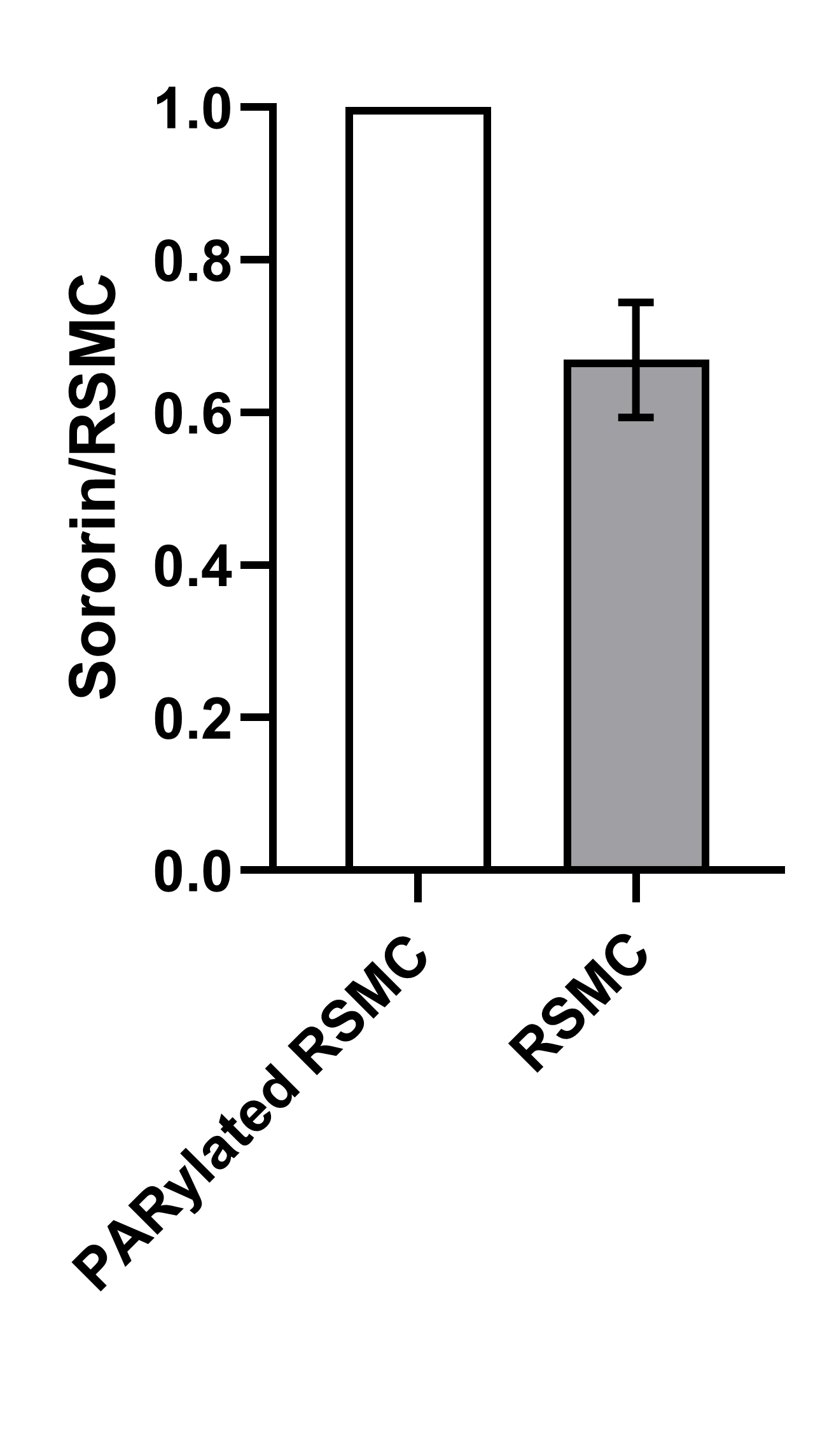

Supplement: Supplementary file 6 — Source data Fig. 5 [file 44318_2025_641_MOESM6_ESM.zip › EMBOJ-2025-120713R_SourceDataForFigure5/FIG 5G/FIG 5G before PS.tif]

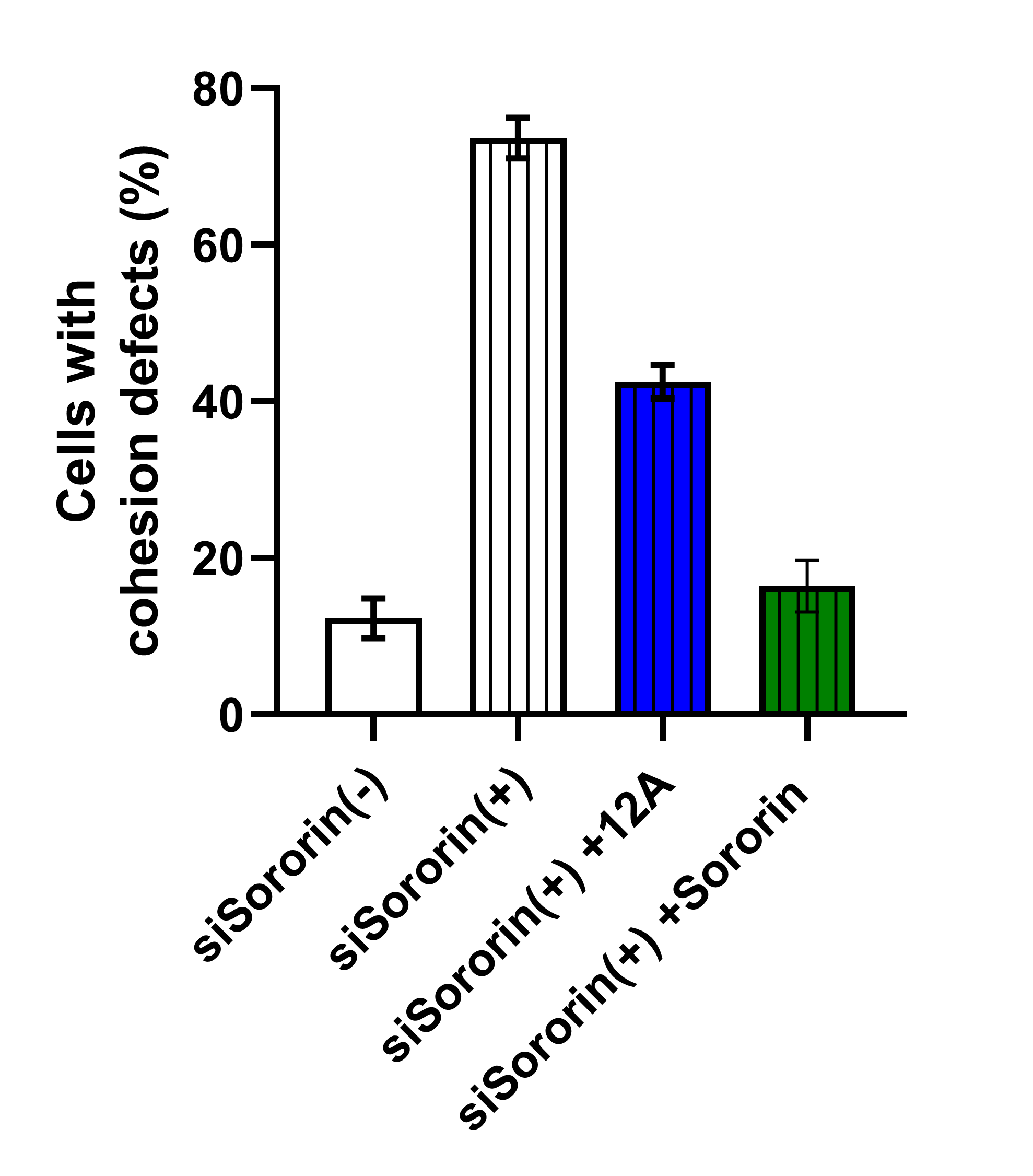

Supplement: Supplementary file 6 — Source data Fig. 5 [file 44318_2025_641_MOESM6_ESM.zip › EMBOJ-2025-120713R_SourceDataForFigure5/FIG 5H/FIG 5H before PS.tif]

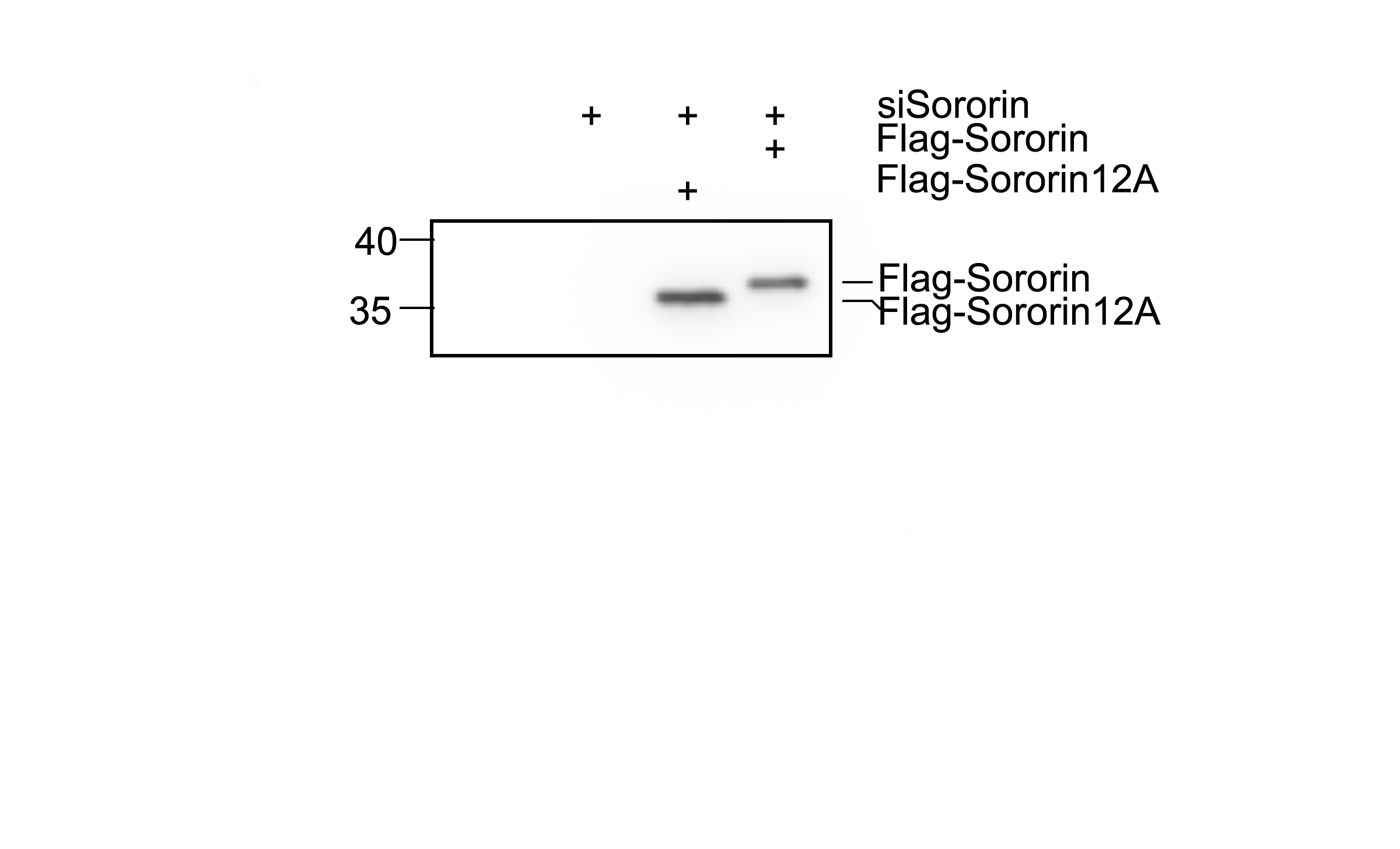

Supplement: Supplementary file 6 — Source data Fig. 5 [file 44318_2025_641_MOESM6_ESM.zip › EMBOJ-2025-120713R_SourceDataForFigure5/FIG 5H/Flag-Sororin SourceData.tif]

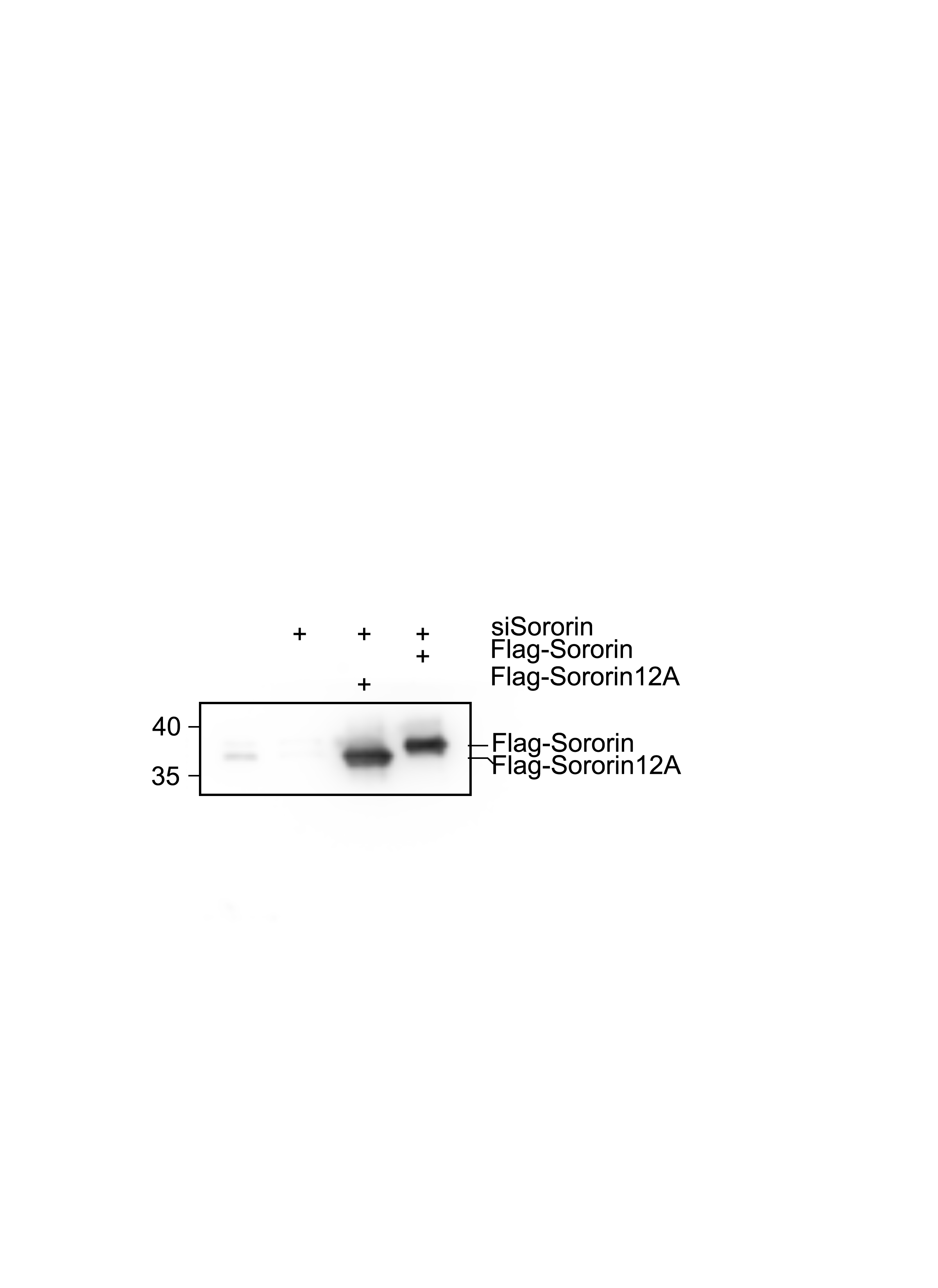

Supplement: Supplementary file 6 — Source data Fig. 5 [file 44318_2025_641_MOESM6_ESM.zip › EMBOJ-2025-120713R_SourceDataForFigure5/FIG 5H/Sororin SourceData.tif]

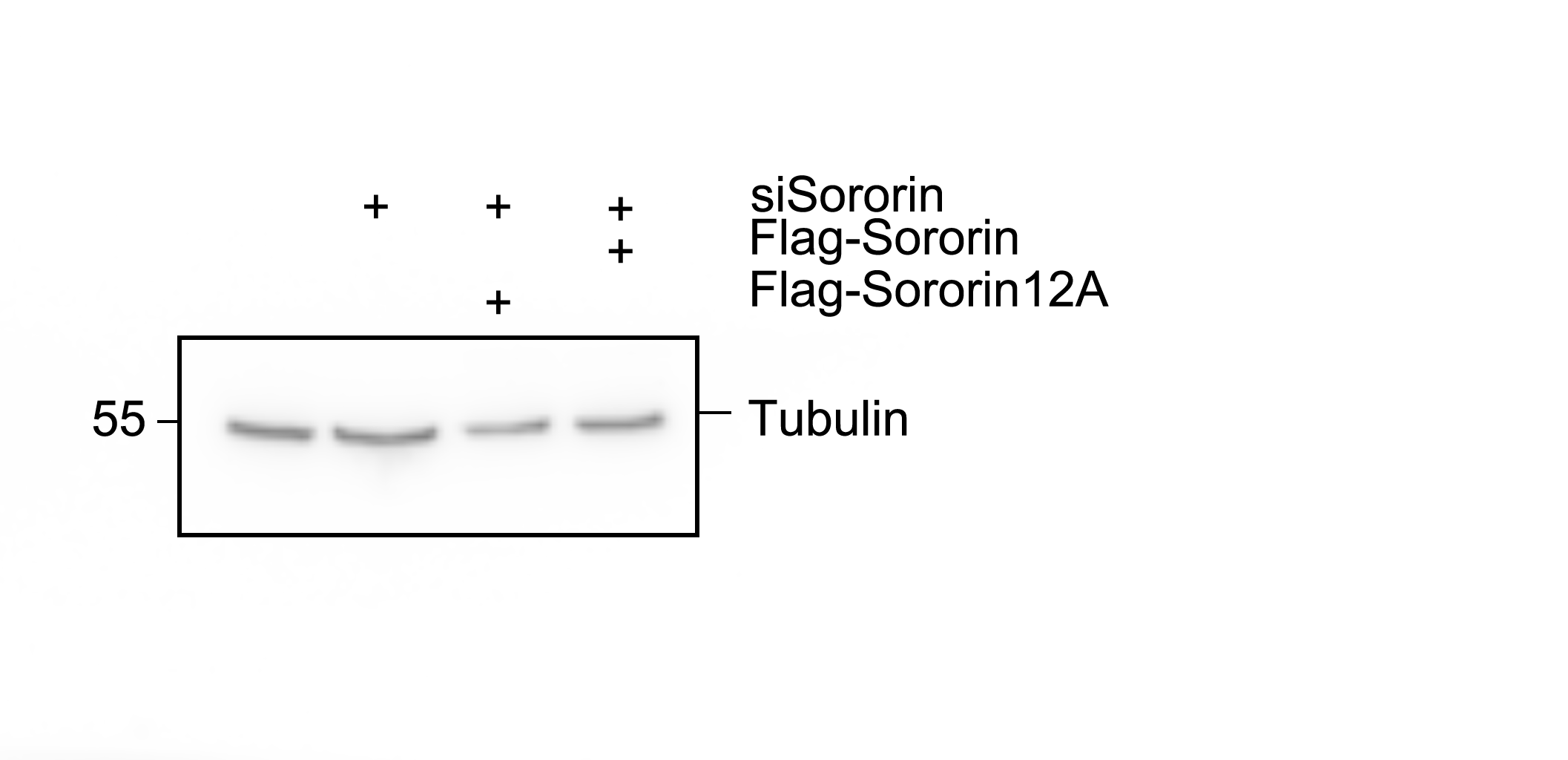

Supplement: Supplementary file 6 — Source data Fig. 5 [file 44318_2025_641_MOESM6_ESM.zip › EMBOJ-2025-120713R_SourceDataForFigure5/FIG 5H/Tubulin SourceData (2).tif]

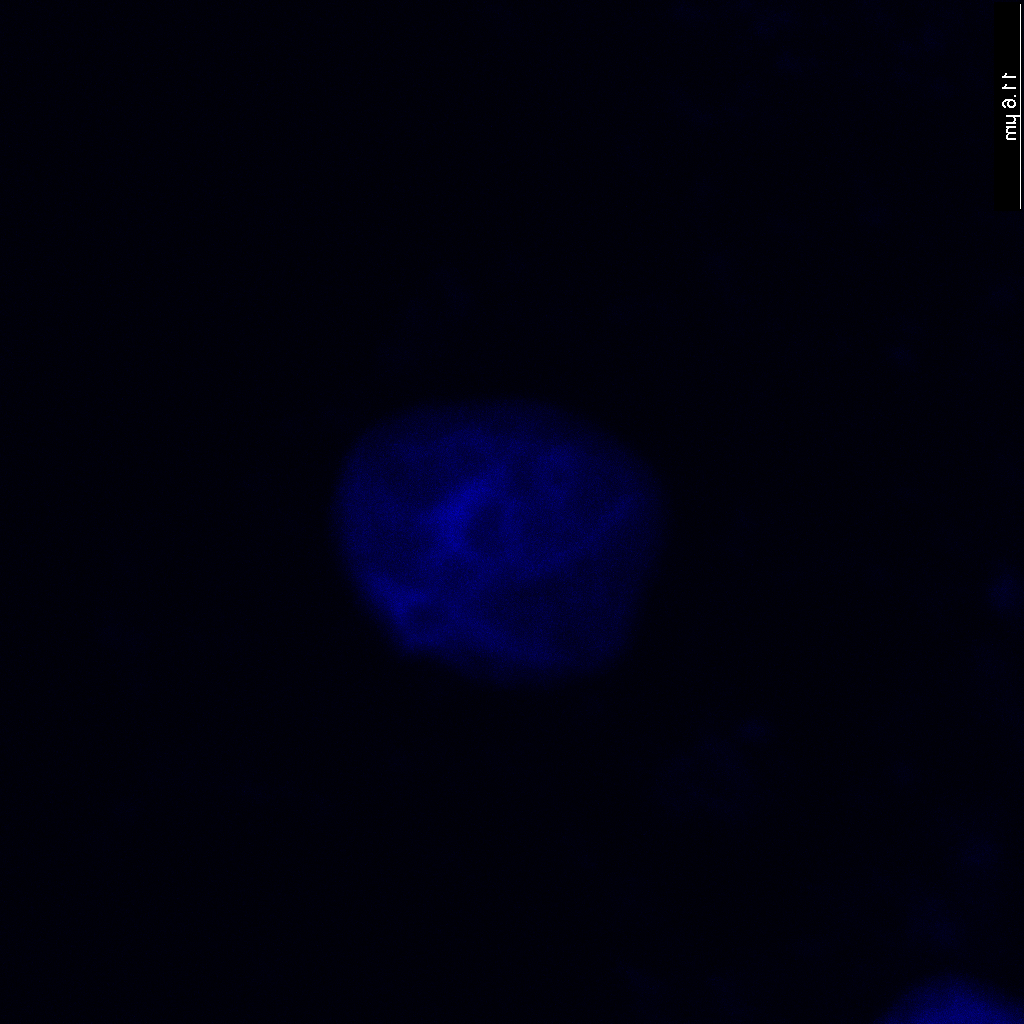

Supplement: Supplementary file 6 — Source data Fig. 5 [file 44318_2025_641_MOESM6_ESM.zip › EMBOJ-2025-120713R_SourceDataForFigure5/FIG 5I/G1-S DMSO/DAPI.tif]

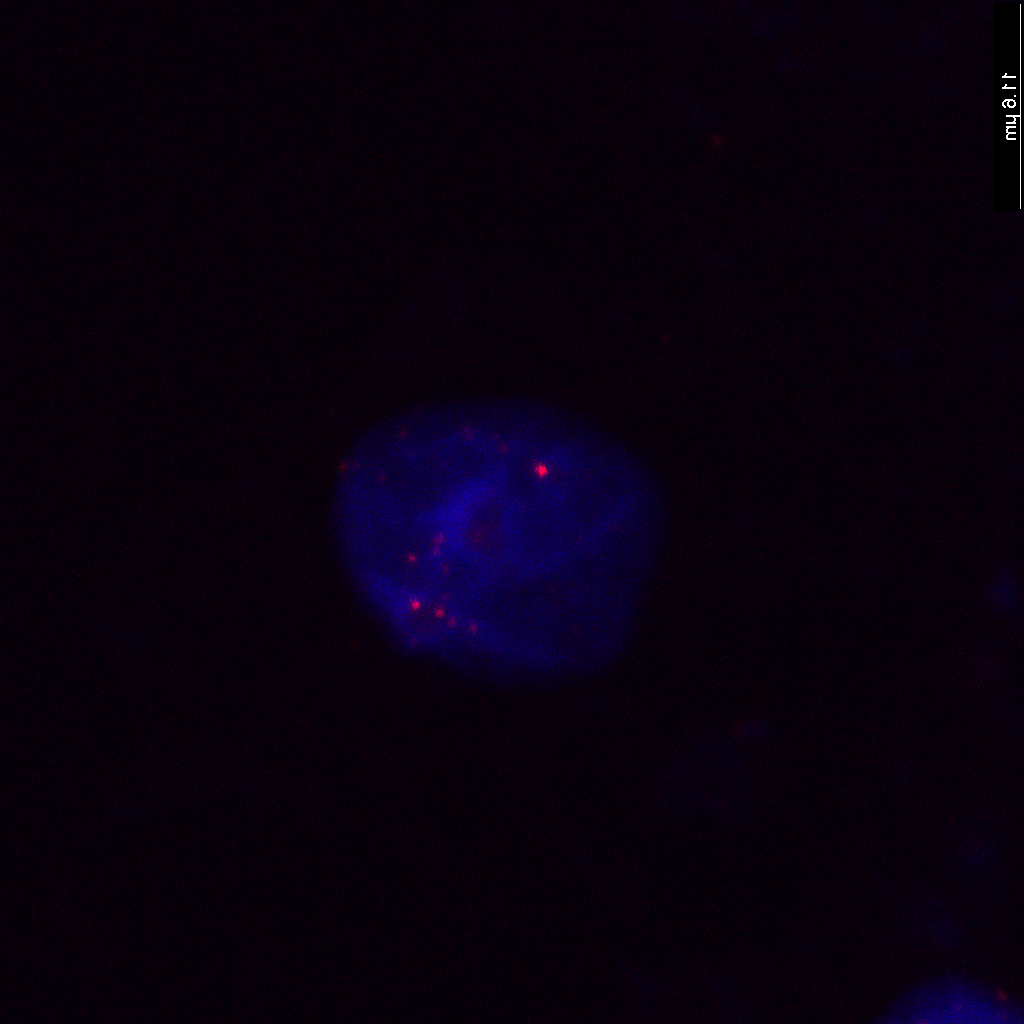

Supplement: Supplementary file 6 — Source data Fig. 5 [file 44318_2025_641_MOESM6_ESM.zip › EMBOJ-2025-120713R_SourceDataForFigure5/FIG 5I/G1-S DMSO/merge.tif]

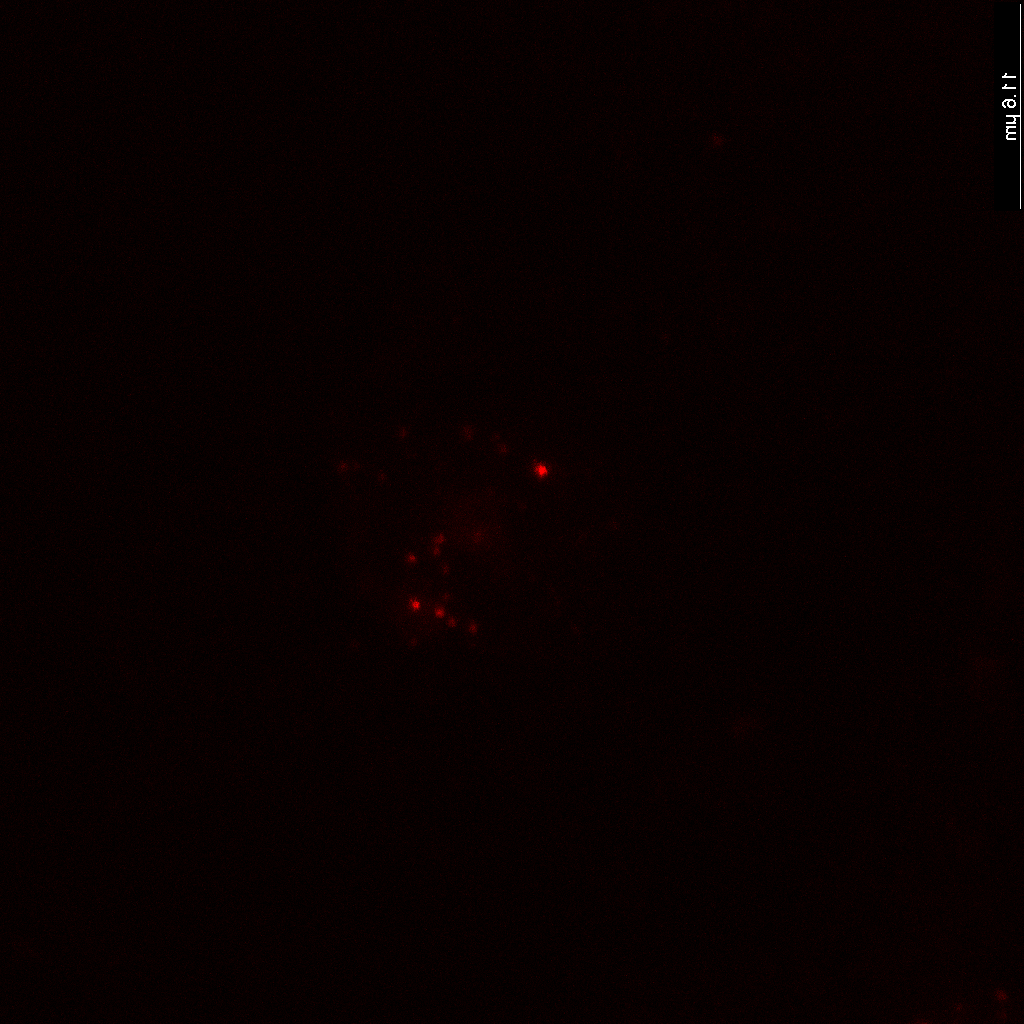

Supplement: Supplementary file 6 — Source data Fig. 5 [file 44318_2025_641_MOESM6_ESM.zip › EMBOJ-2025-120713R_SourceDataForFigure5/FIG 5I/G1-S DMSO/PLA.tif]

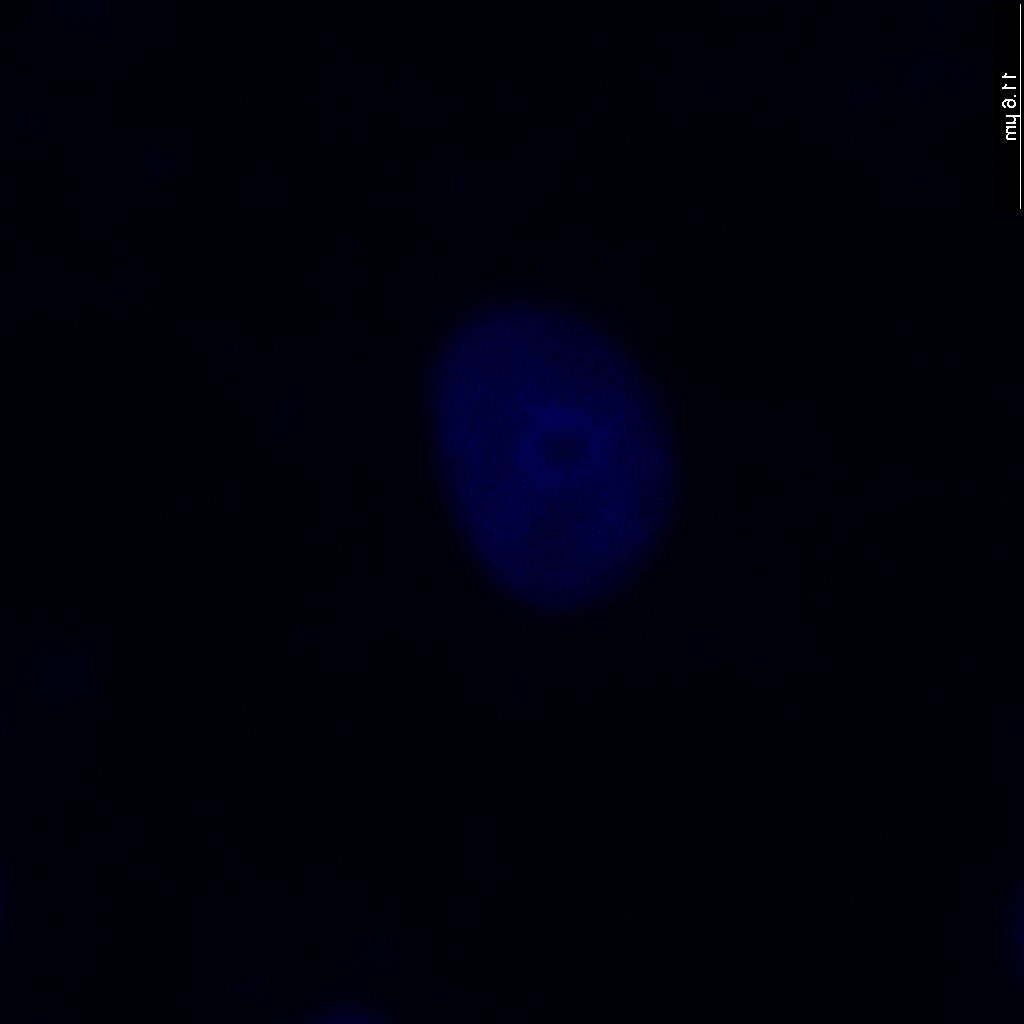

Supplement: Supplementary file 6 — Source data Fig. 5 [file 44318_2025_641_MOESM6_ESM.zip › EMBOJ-2025-120713R_SourceDataForFigure5/FIG 5I/G1-S Olaparib/DAPI.tif]

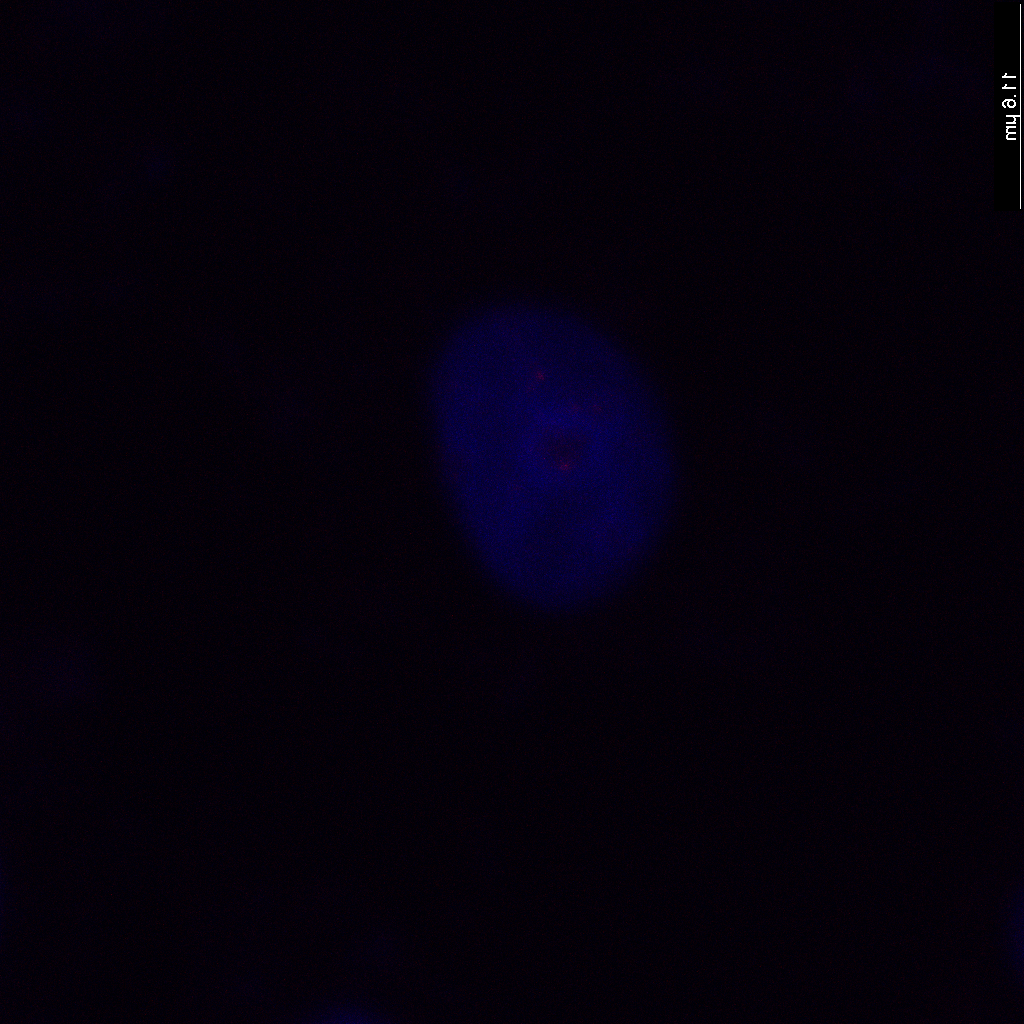

Supplement: Supplementary file 6 — Source data Fig. 5 [file 44318_2025_641_MOESM6_ESM.zip › EMBOJ-2025-120713R_SourceDataForFigure5/FIG 5I/G1-S Olaparib/merge.tif]

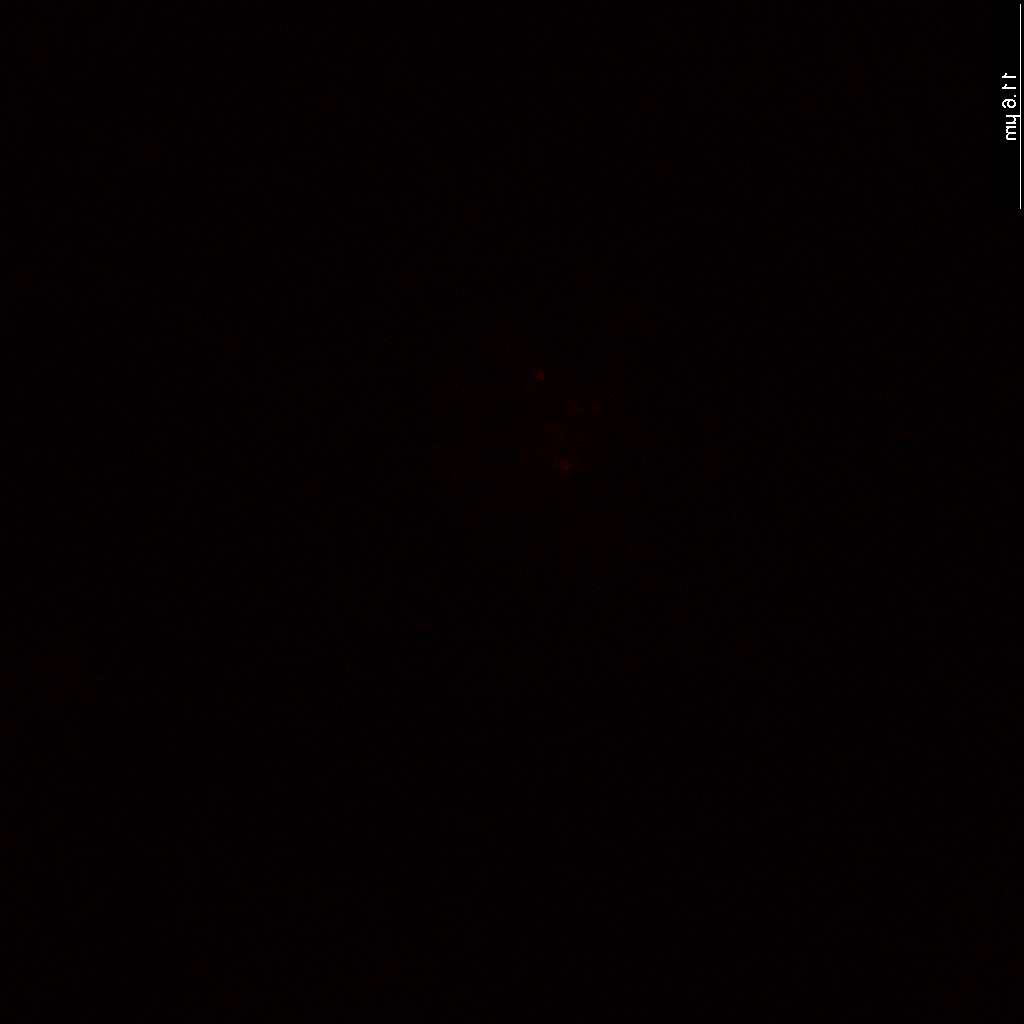

Supplement: Supplementary file 6 — Source data Fig. 5 [file 44318_2025_641_MOESM6_ESM.zip › EMBOJ-2025-120713R_SourceDataForFigure5/FIG 5I/G1-S Olaparib/PLA.tif]

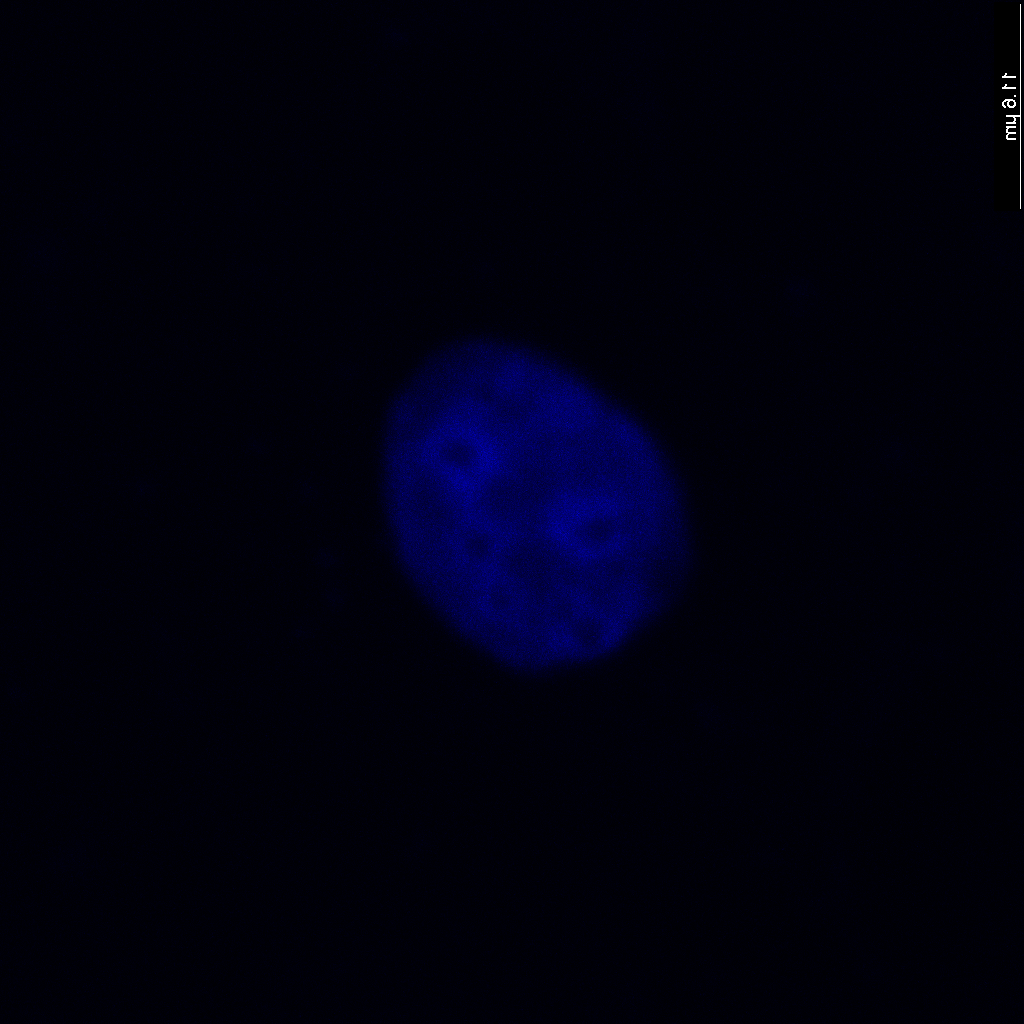

Supplement: Supplementary file 6 — Source data Fig. 5 [file 44318_2025_641_MOESM6_ESM.zip › EMBOJ-2025-120713R_SourceDataForFigure5/FIG 5I/mid S DMSO/DAPI.tif]

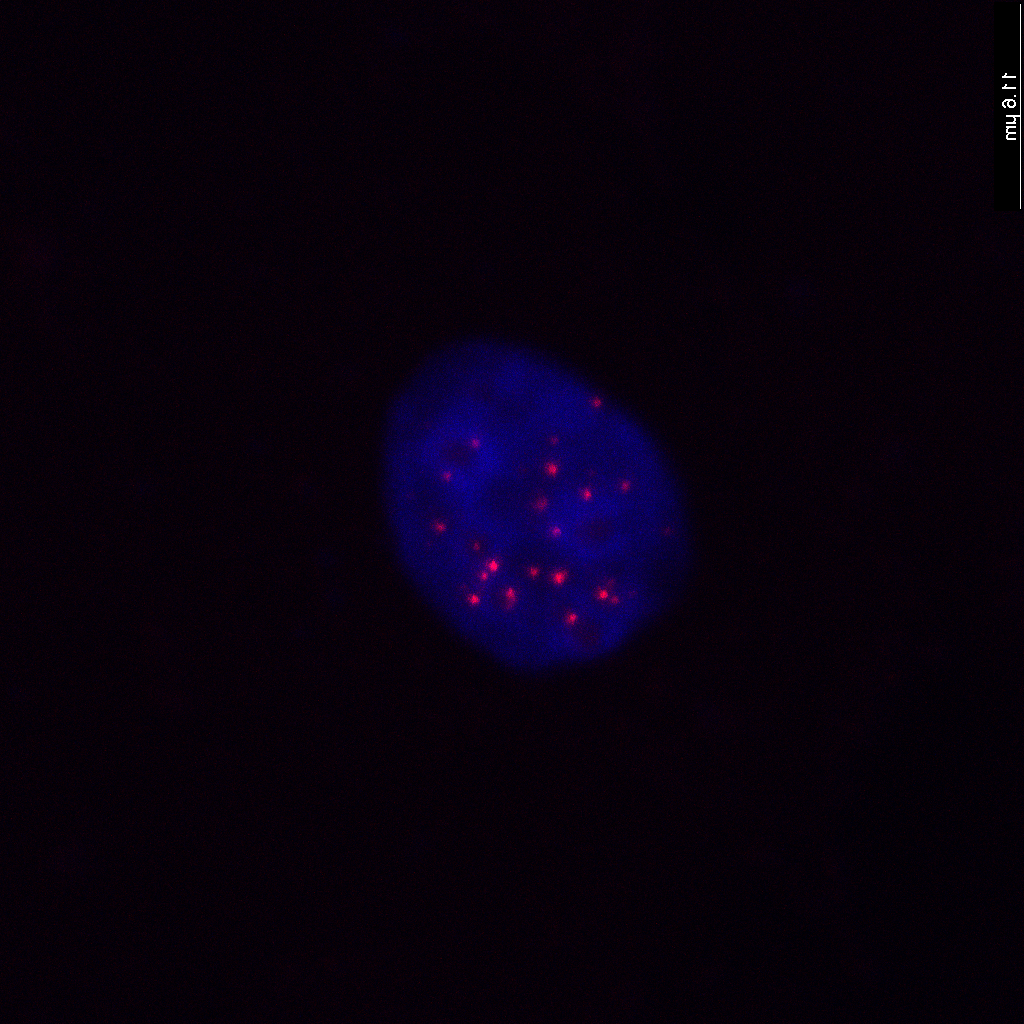

Supplement: Supplementary file 6 — Source data Fig. 5 [file 44318_2025_641_MOESM6_ESM.zip › EMBOJ-2025-120713R_SourceDataForFigure5/FIG 5I/mid S DMSO/merge.tif]

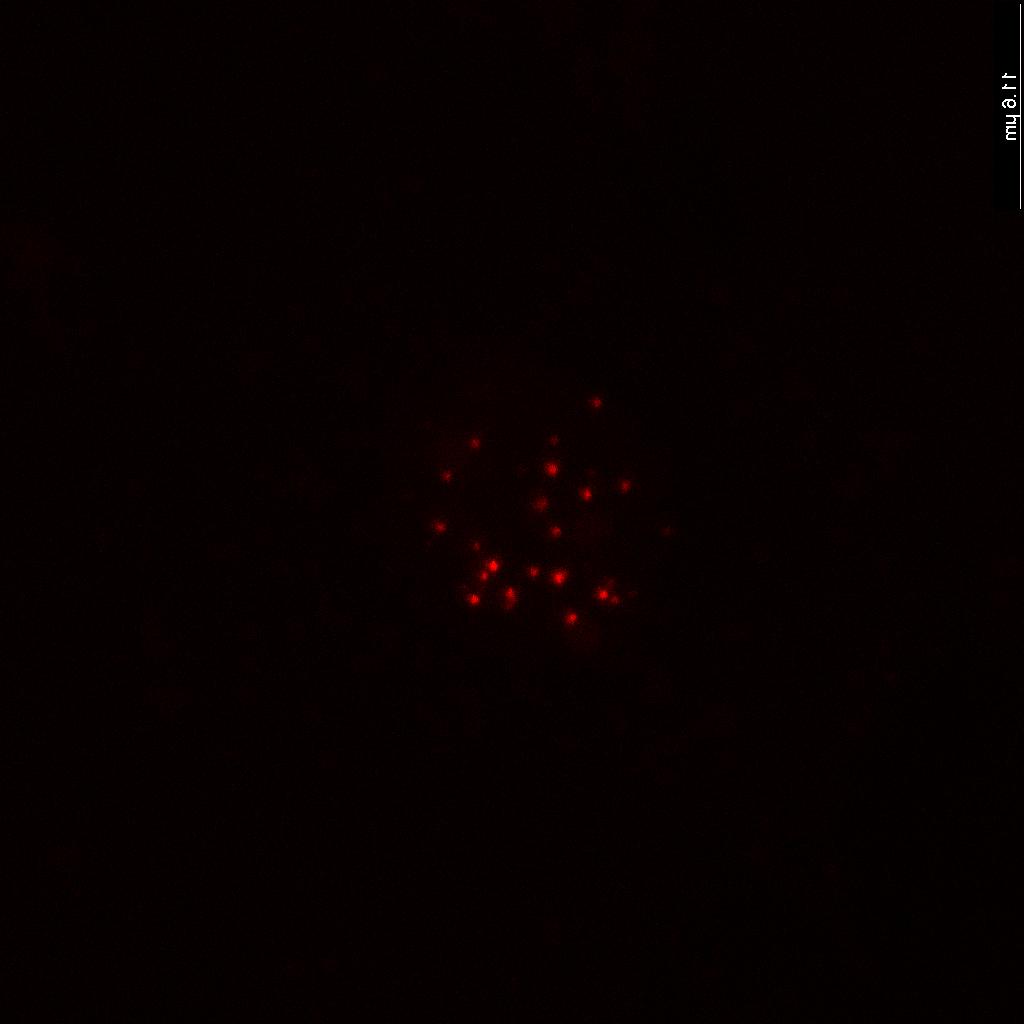

Supplement: Supplementary file 6 — Source data Fig. 5 [file 44318_2025_641_MOESM6_ESM.zip › EMBOJ-2025-120713R_SourceDataForFigure5/FIG 5I/mid S DMSO/PLA.tif]

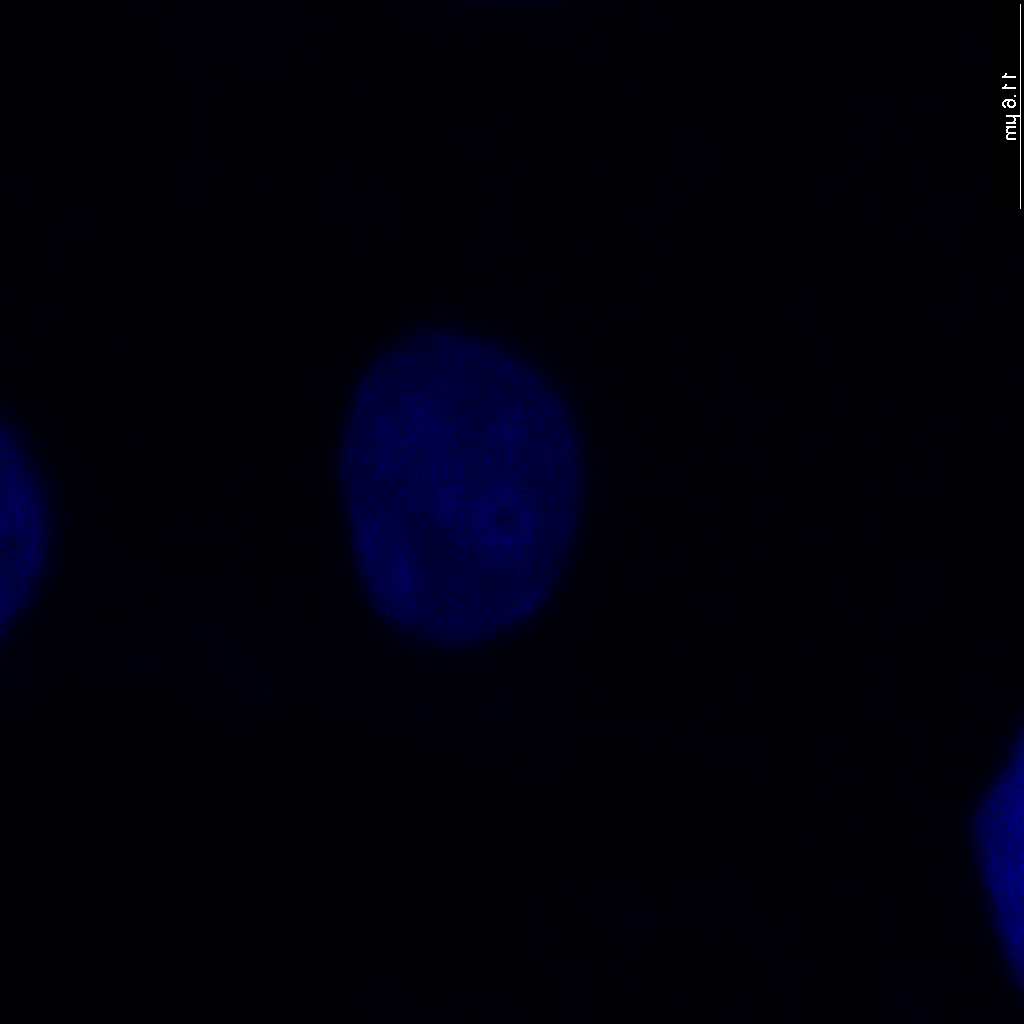

Supplement: Supplementary file 6 — Source data Fig. 5 [file 44318_2025_641_MOESM6_ESM.zip › EMBOJ-2025-120713R_SourceDataForFigure5/FIG 5I/mid S Olaparib/DAPI.tif]

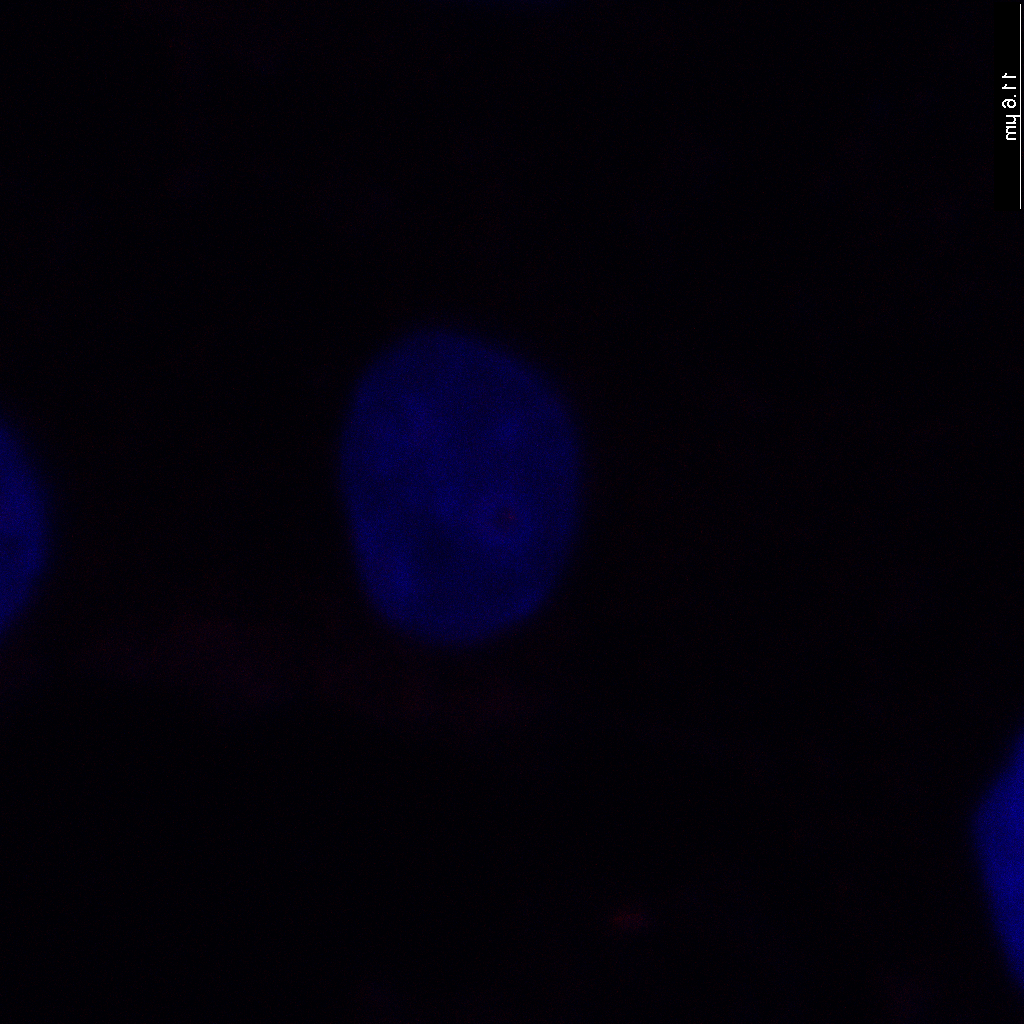

Supplement: Supplementary file 6 — Source data Fig. 5 [file 44318_2025_641_MOESM6_ESM.zip › EMBOJ-2025-120713R_SourceDataForFigure5/FIG 5I/mid S Olaparib/merge.tif]

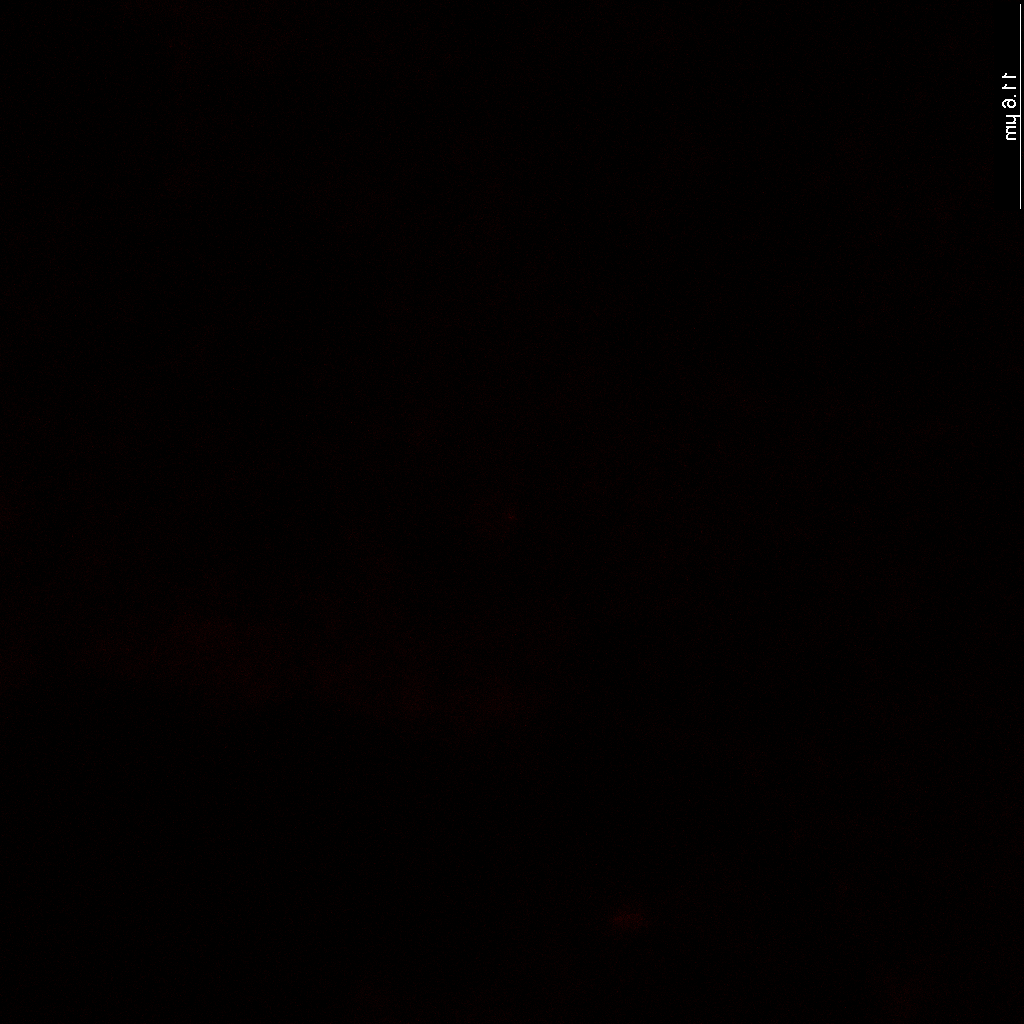

Supplement: Supplementary file 6 — Source data Fig. 5 [file 44318_2025_641_MOESM6_ESM.zip › EMBOJ-2025-120713R_SourceDataForFigure5/FIG 5I/mid S Olaparib/PLA.tif]

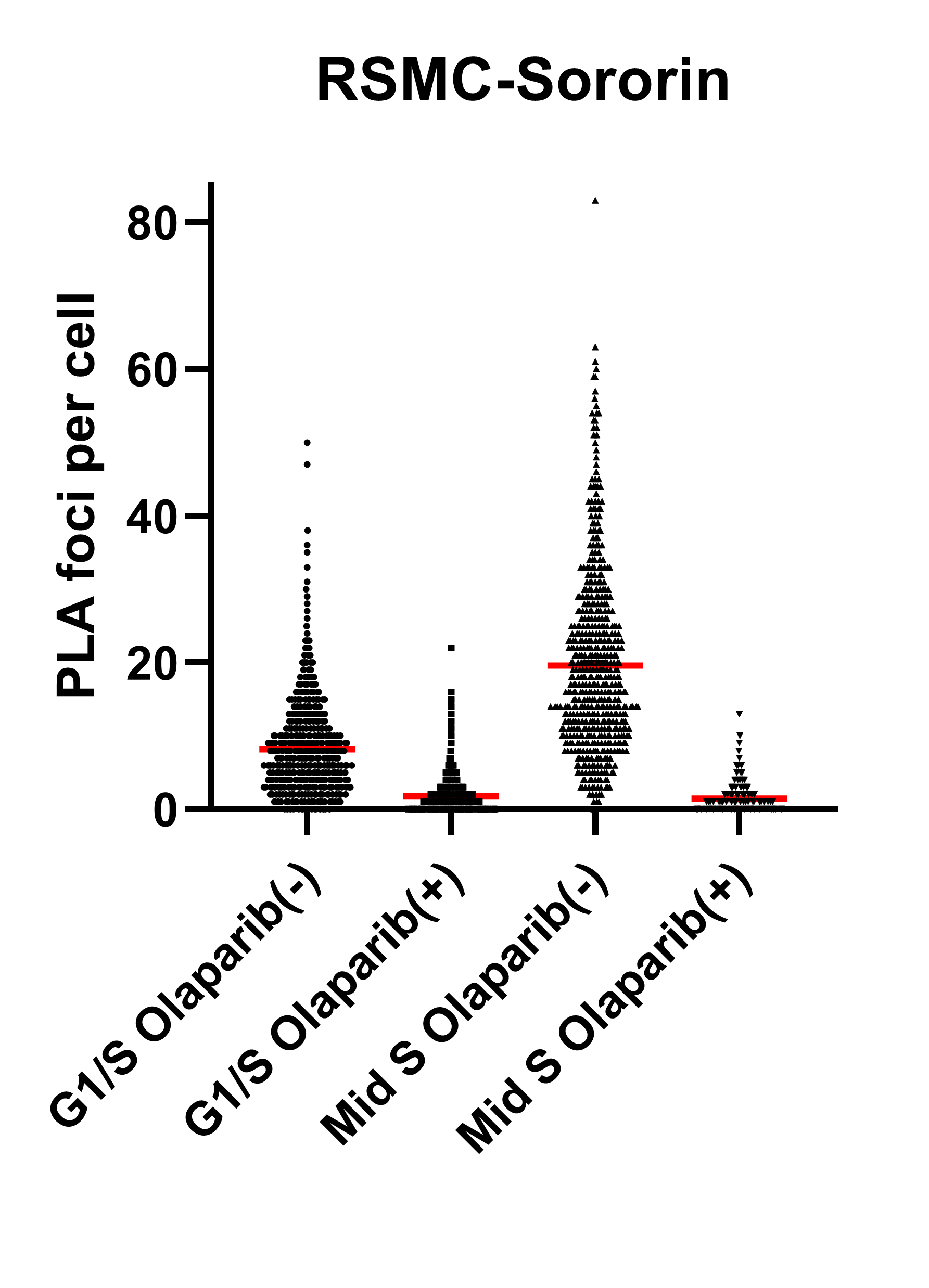

Supplement: Supplementary file 6 — Source data Fig. 5 [file 44318_2025_641_MOESM6_ESM.zip › EMBOJ-2025-120713R_SourceDataForFigure5/FIG 5J/FIG 5J before PS.tif]

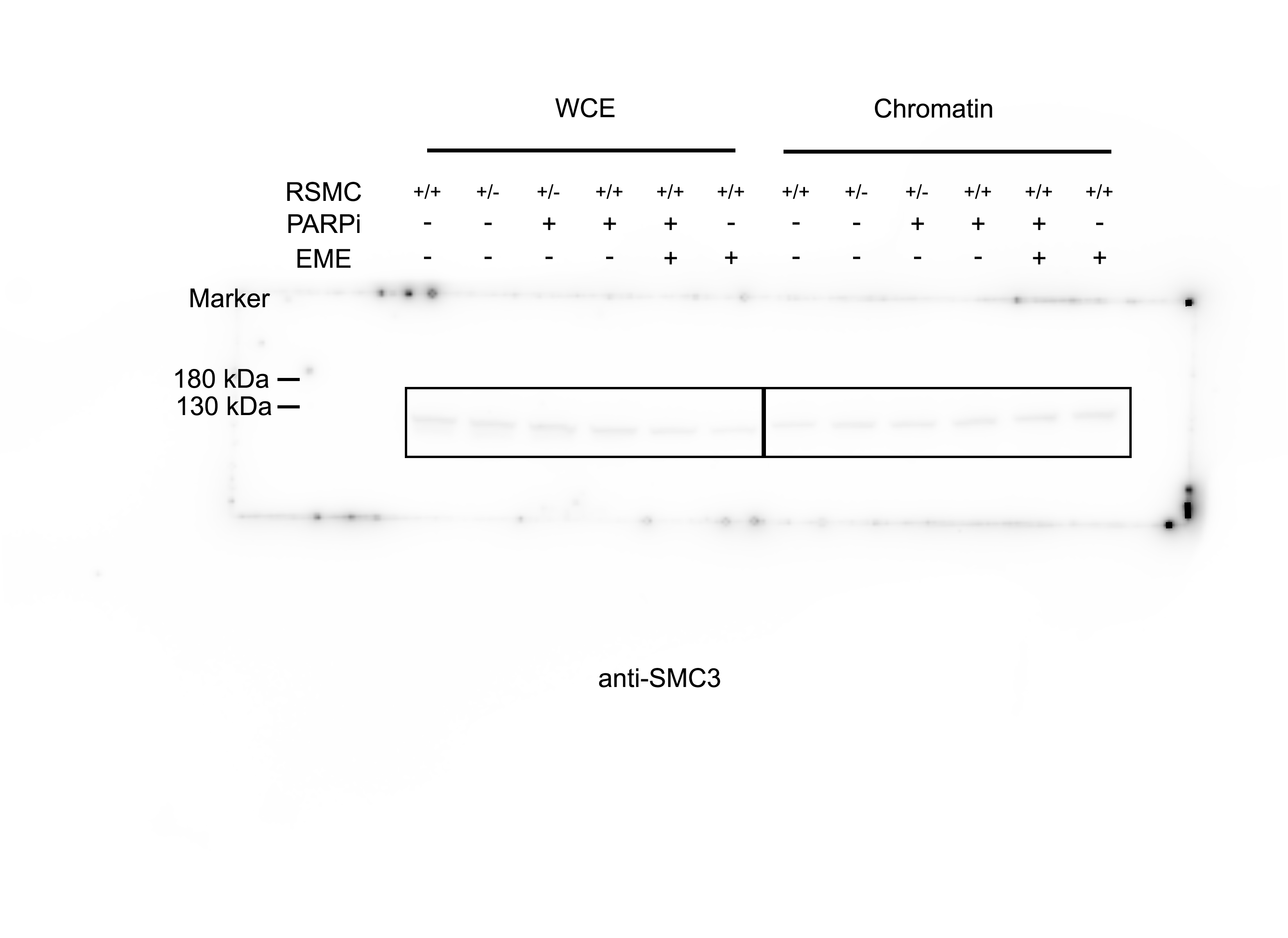

Supplement: Supplementary file 7 — Source data Fig. 6 [file 44318_2025_641_MOESM7_ESM.zip › EMBOJ-2025-120713R_SourceDataForFigure6/FIG 6A/SMC3 RAW data.tif]

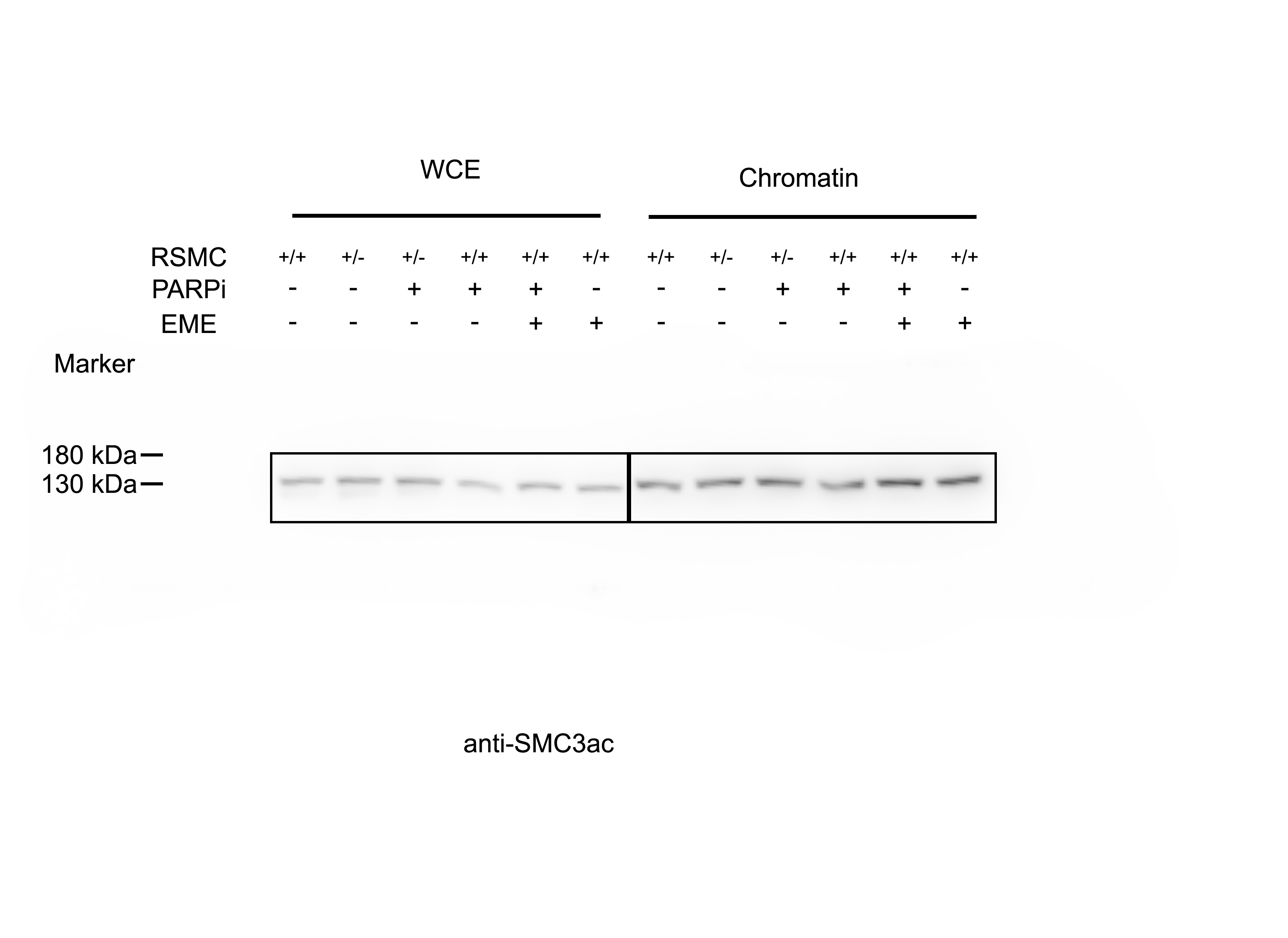

Supplement: Supplementary file 7 — Source data Fig. 6 [file 44318_2025_641_MOESM7_ESM.zip › EMBOJ-2025-120713R_SourceDataForFigure6/FIG 6A/SMC3ac RAW data.tif]

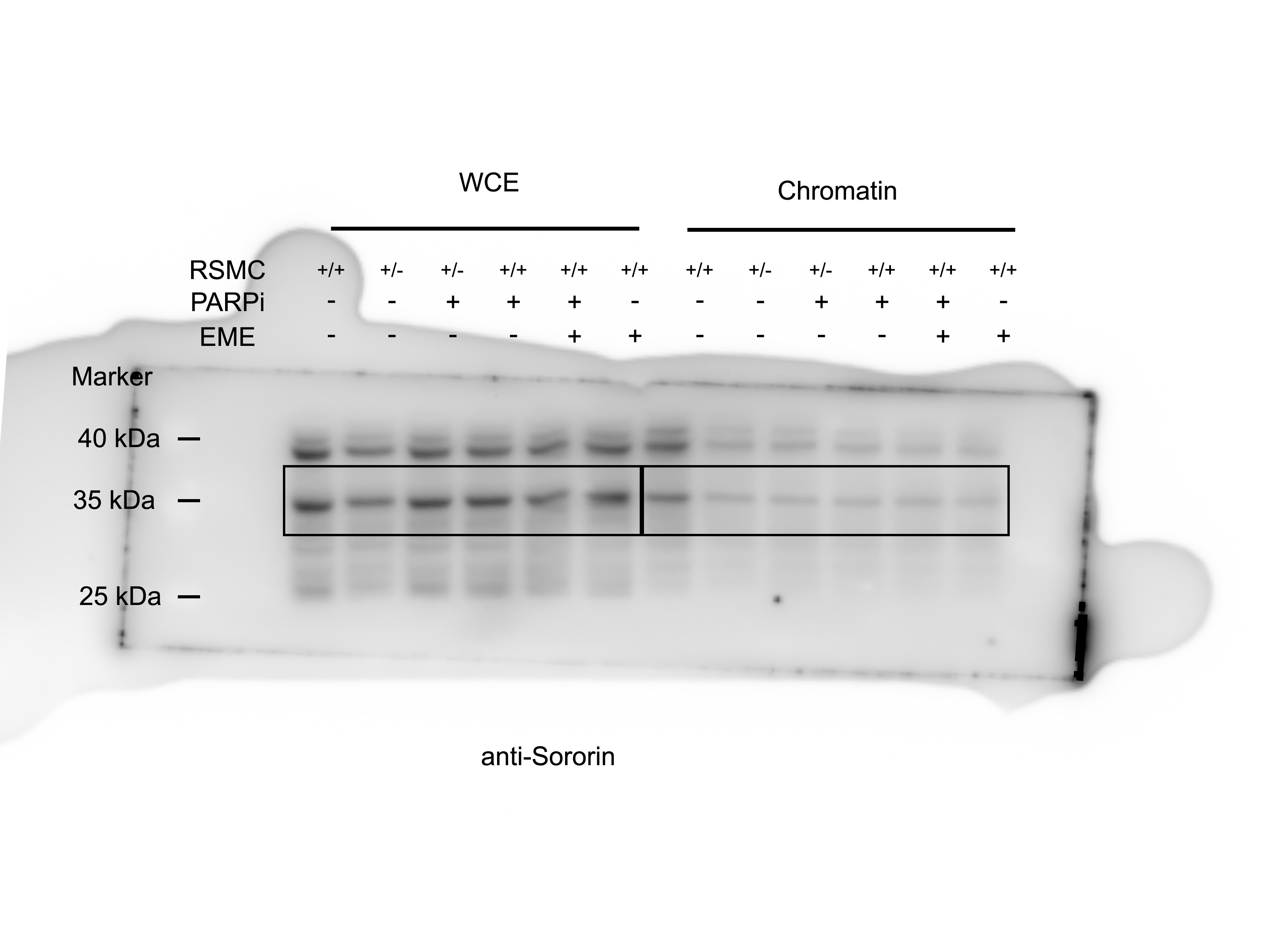

Supplement: Supplementary file 7 — Source data Fig. 6 [file 44318_2025_641_MOESM7_ESM.zip › EMBOJ-2025-120713R_SourceDataForFigure6/FIG 6A/sororin RAW data.tif]

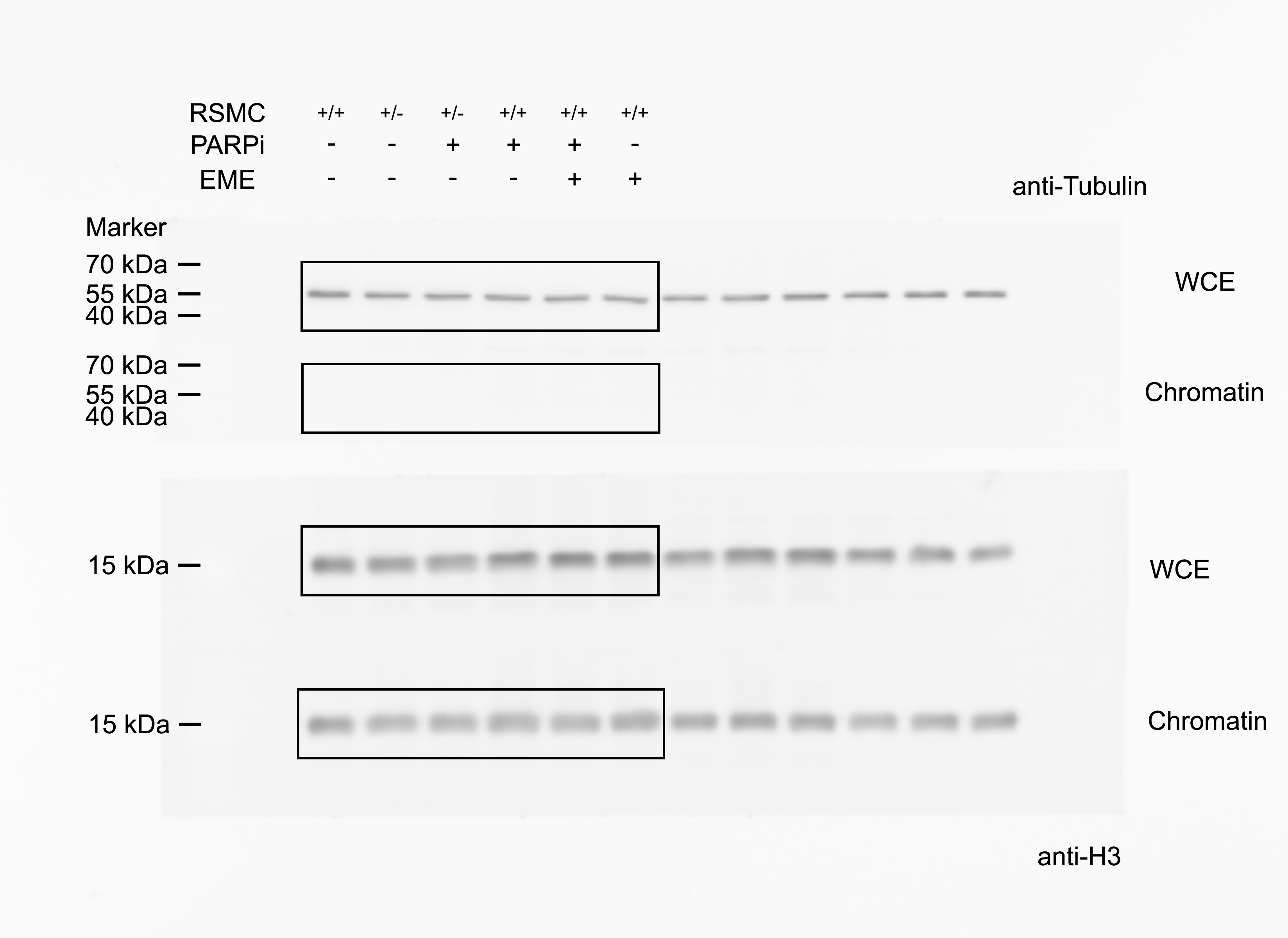

Supplement: Supplementary file 7 — Source data Fig. 6 [file 44318_2025_641_MOESM7_ESM.zip › EMBOJ-2025-120713R_SourceDataForFigure6/FIG 6A/Tubulin&H3 RAW data.tif]

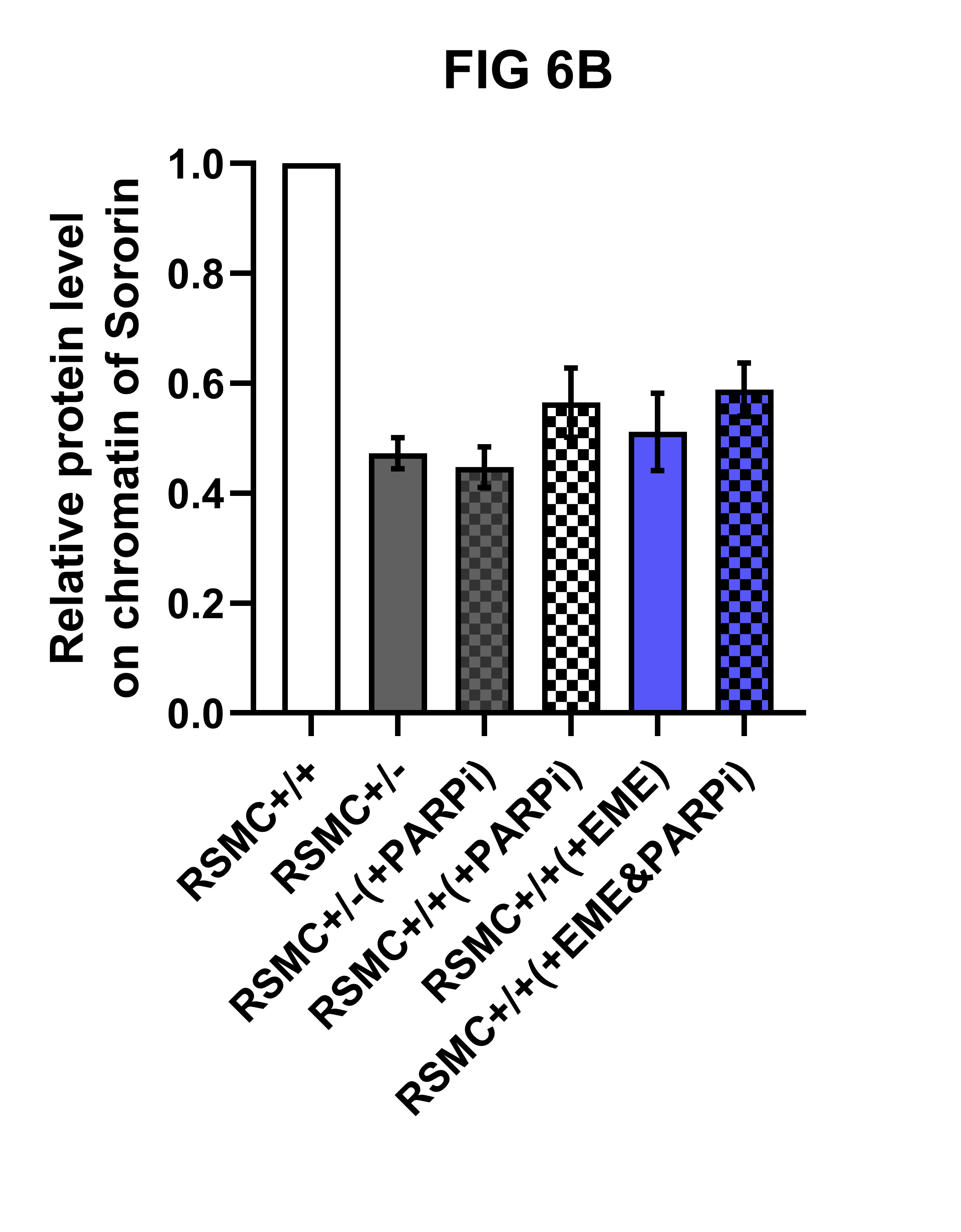

Supplement: Supplementary file 7 — Source data Fig. 6 [file 44318_2025_641_MOESM7_ESM.zip › EMBOJ-2025-120713R_SourceDataForFigure6/FIG 6B/FIG 6B before PS.tif]

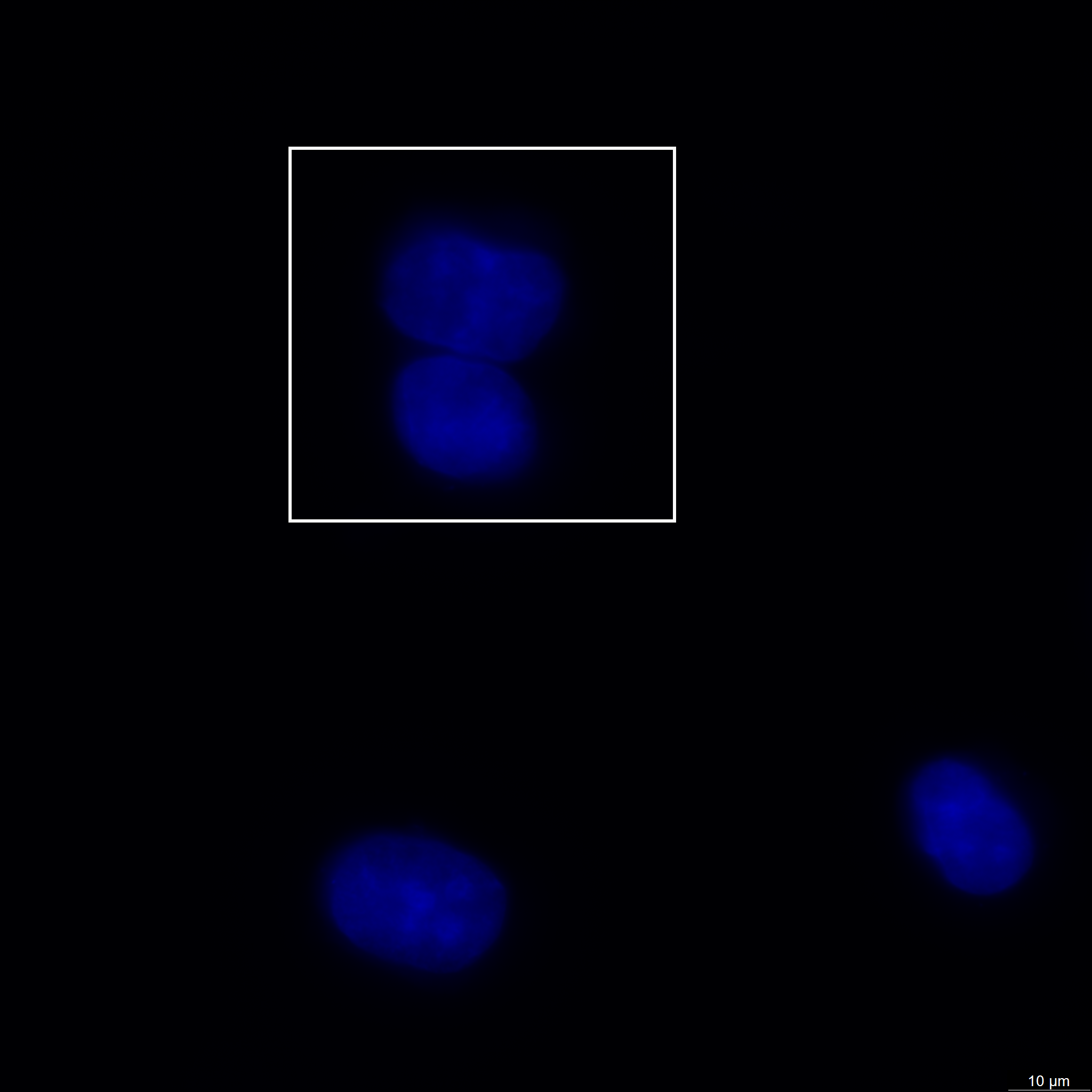

Supplement: Supplementary file 7 — Source data Fig. 6 [file 44318_2025_641_MOESM7_ESM.zip › EMBOJ-2025-120713R_SourceDataForFigure6/FIG 6C/RSMC KO/DAPI.tif]

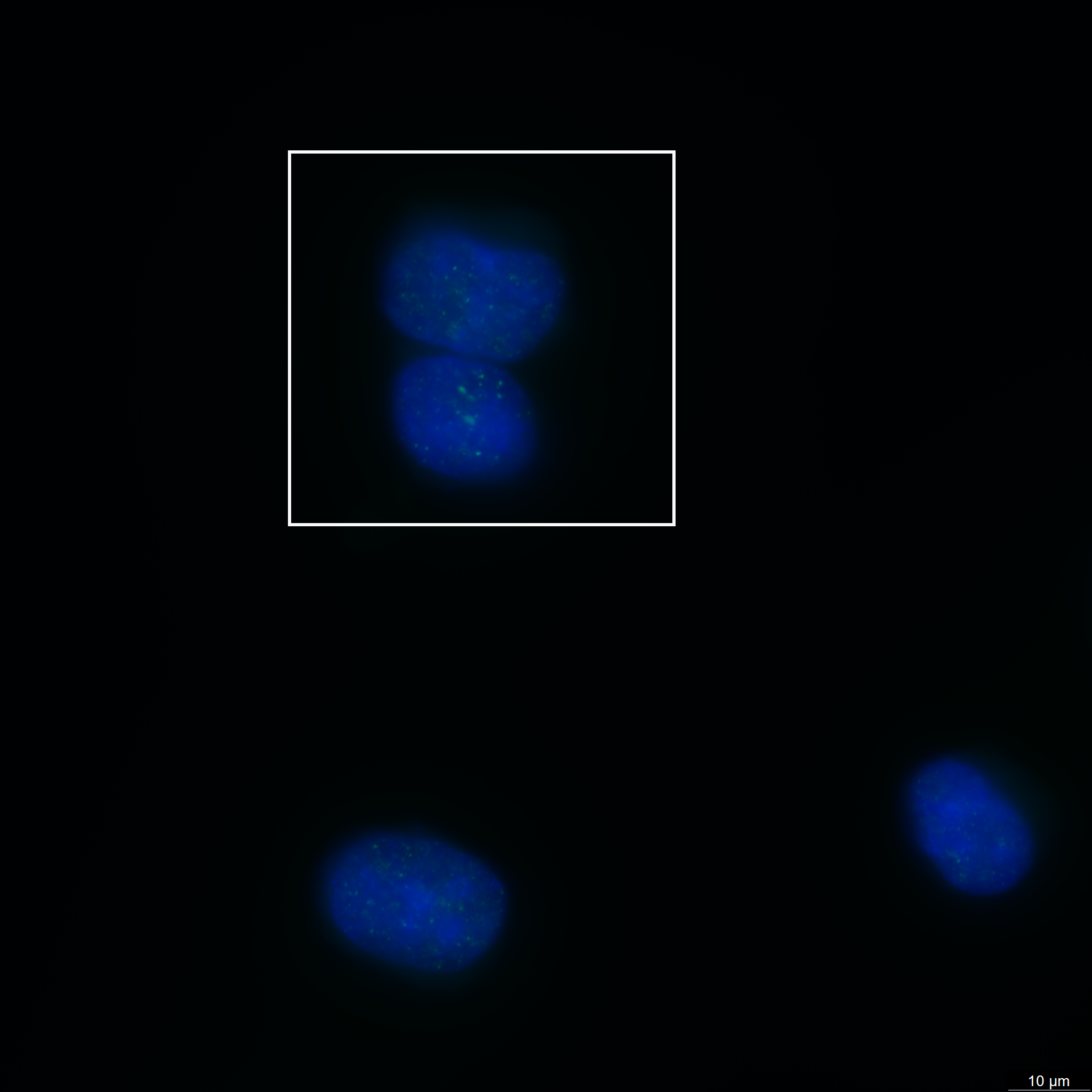

Supplement: Supplementary file 7 — Source data Fig. 6 [file 44318_2025_641_MOESM7_ESM.zip › EMBOJ-2025-120713R_SourceDataForFigure6/FIG 6C/RSMC KO/merge.tif]

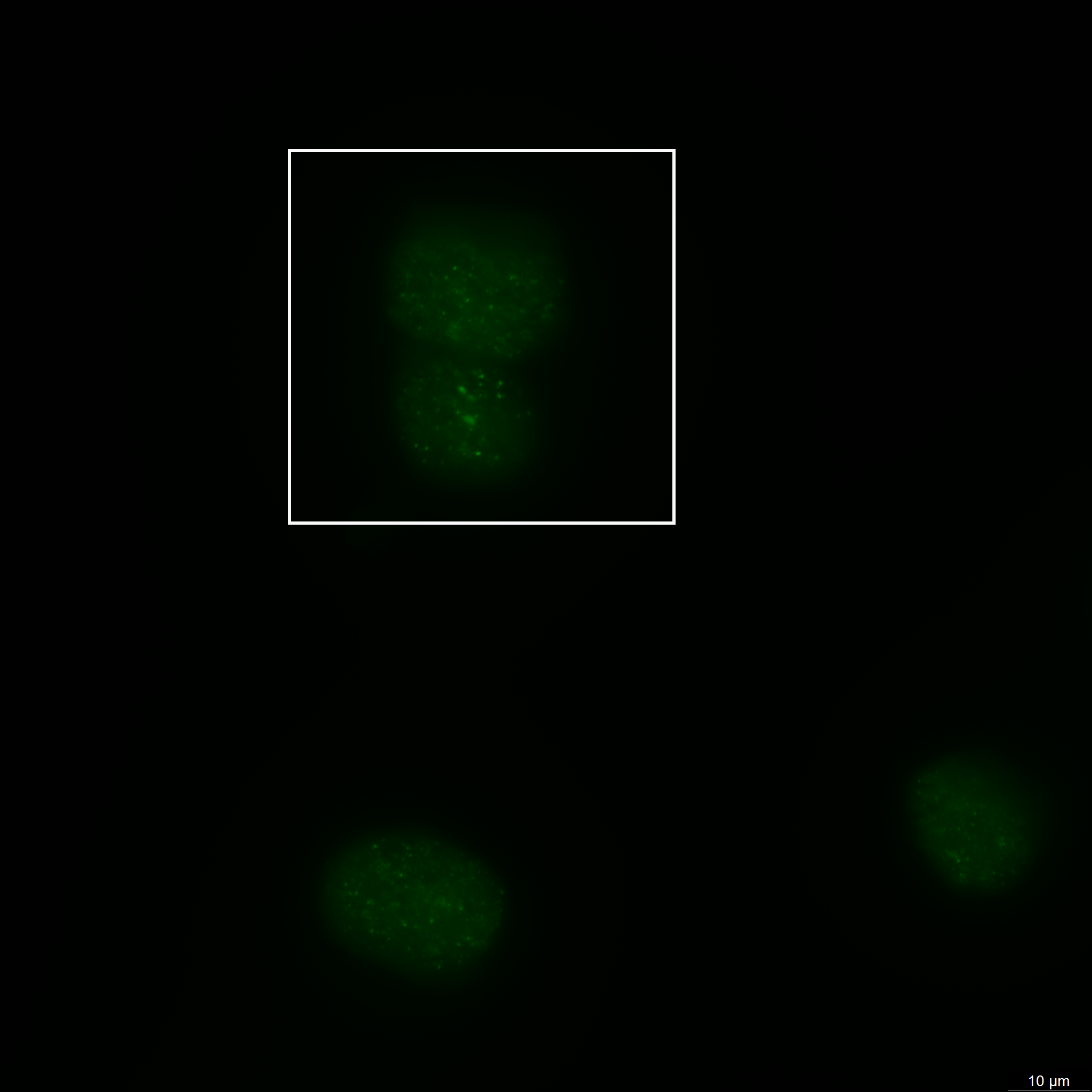

Supplement: Supplementary file 7 — Source data Fig. 6 [file 44318_2025_641_MOESM7_ESM.zip › EMBOJ-2025-120713R_SourceDataForFigure6/FIG 6C/RSMC KO/Sororin.tif]

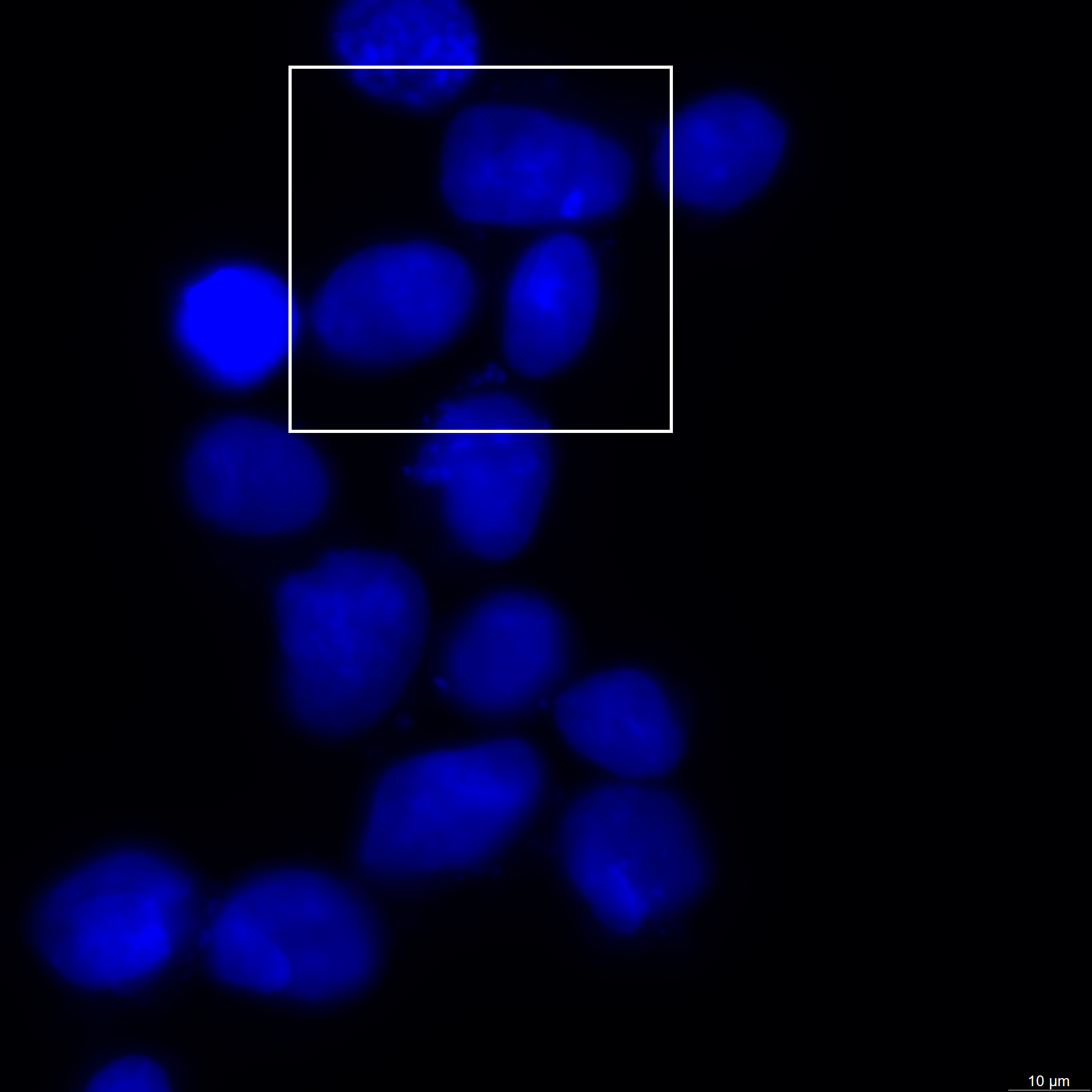

Supplement: Supplementary file 7 — Source data Fig. 6 [file 44318_2025_641_MOESM7_ESM.zip › EMBOJ-2025-120713R_SourceDataForFigure6/FIG 6C/RSMC KO+PARPi/DAPI.tif]

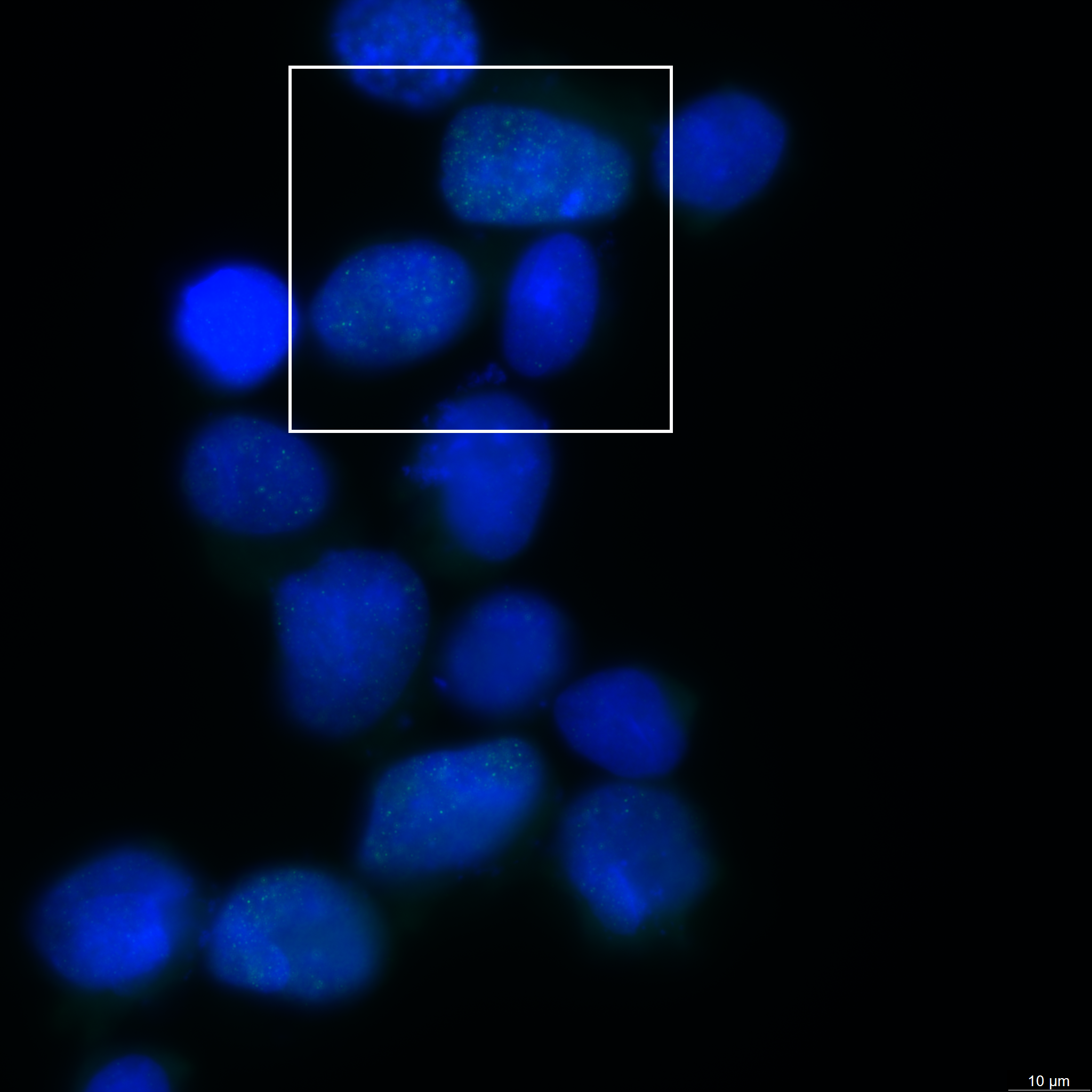

Supplement: Supplementary file 7 — Source data Fig. 6 [file 44318_2025_641_MOESM7_ESM.zip › EMBOJ-2025-120713R_SourceDataForFigure6/FIG 6C/RSMC KO+PARPi/merge.tif]

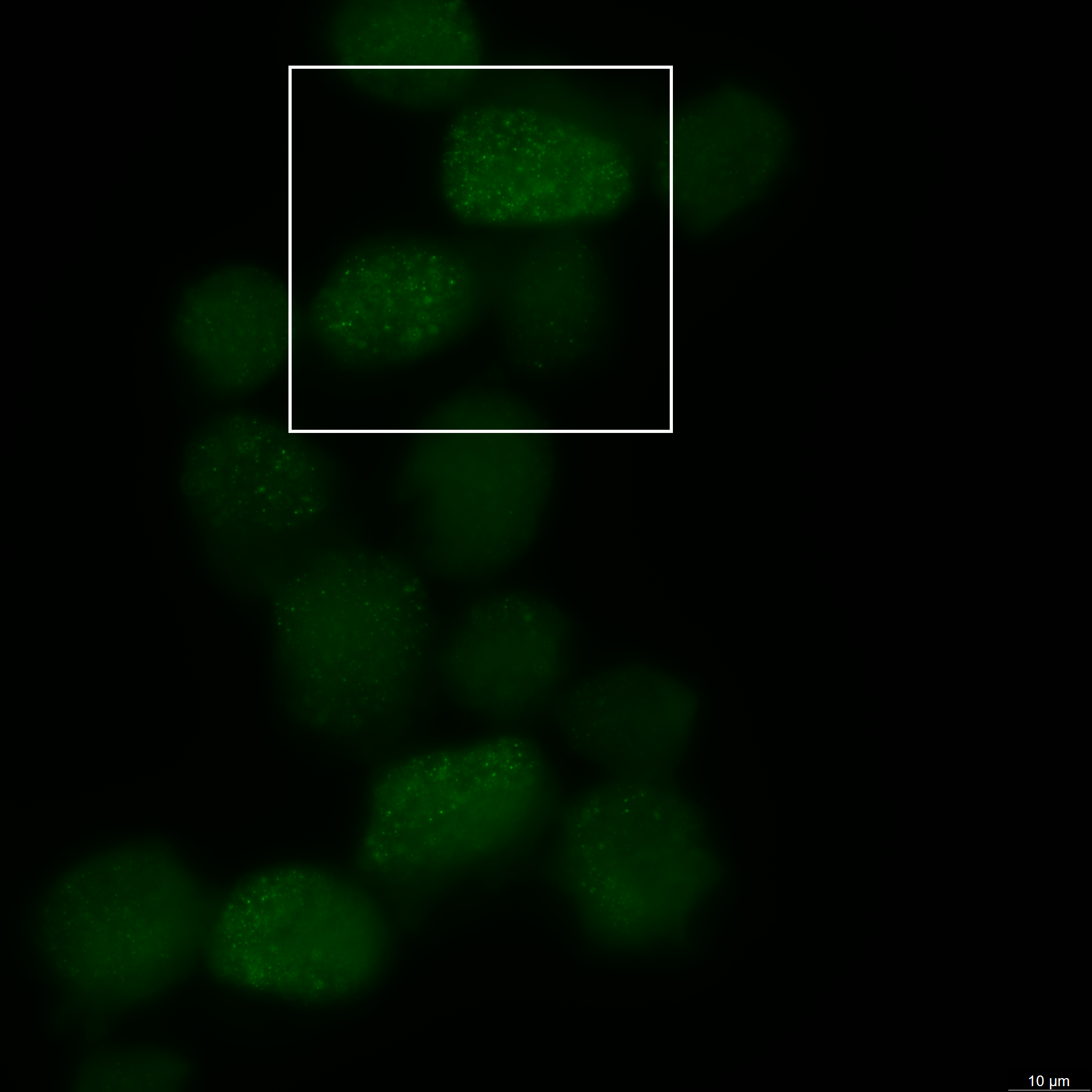

Supplement: Supplementary file 7 — Source data Fig. 6 [file 44318_2025_641_MOESM7_ESM.zip › EMBOJ-2025-120713R_SourceDataForFigure6/FIG 6C/RSMC KO+PARPi/Sororin.tif]

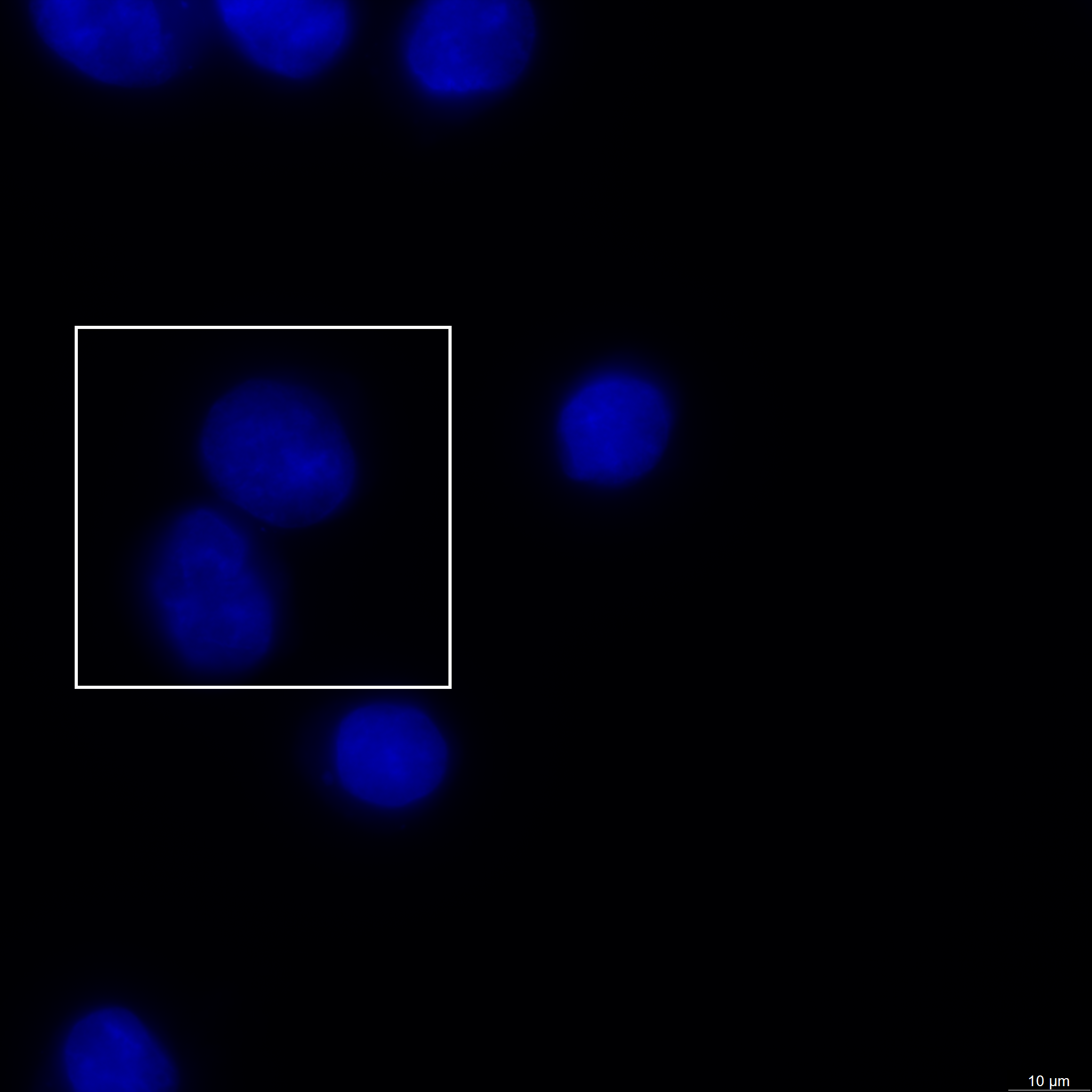

Supplement: Supplementary file 7 — Source data Fig. 6 [file 44318_2025_641_MOESM7_ESM.zip › EMBOJ-2025-120713R_SourceDataForFigure6/FIG 6C/WT/DAPI.tif]

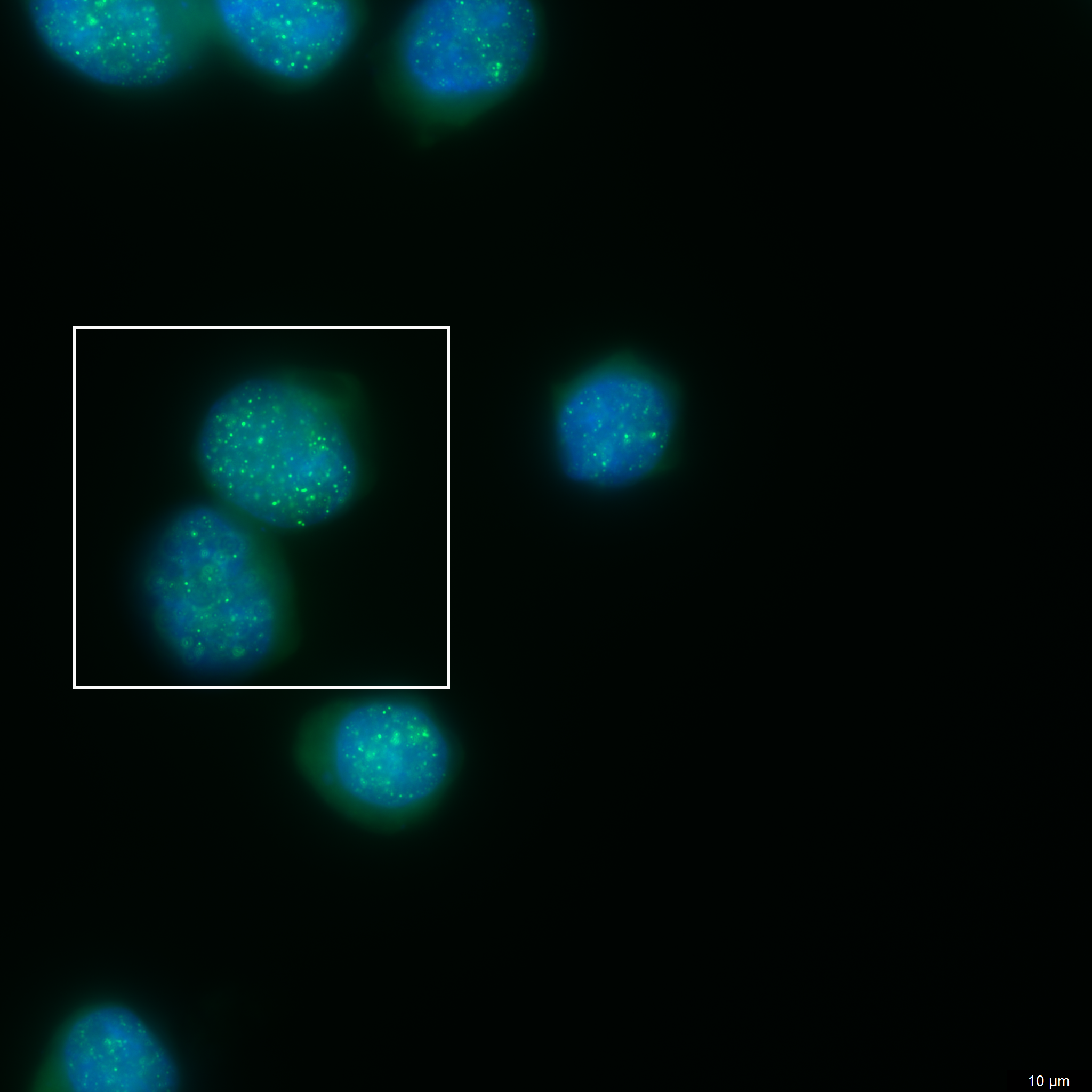

Supplement: Supplementary file 7 — Source data Fig. 6 [file 44318_2025_641_MOESM7_ESM.zip › EMBOJ-2025-120713R_SourceDataForFigure6/FIG 6C/WT/merge.tif]

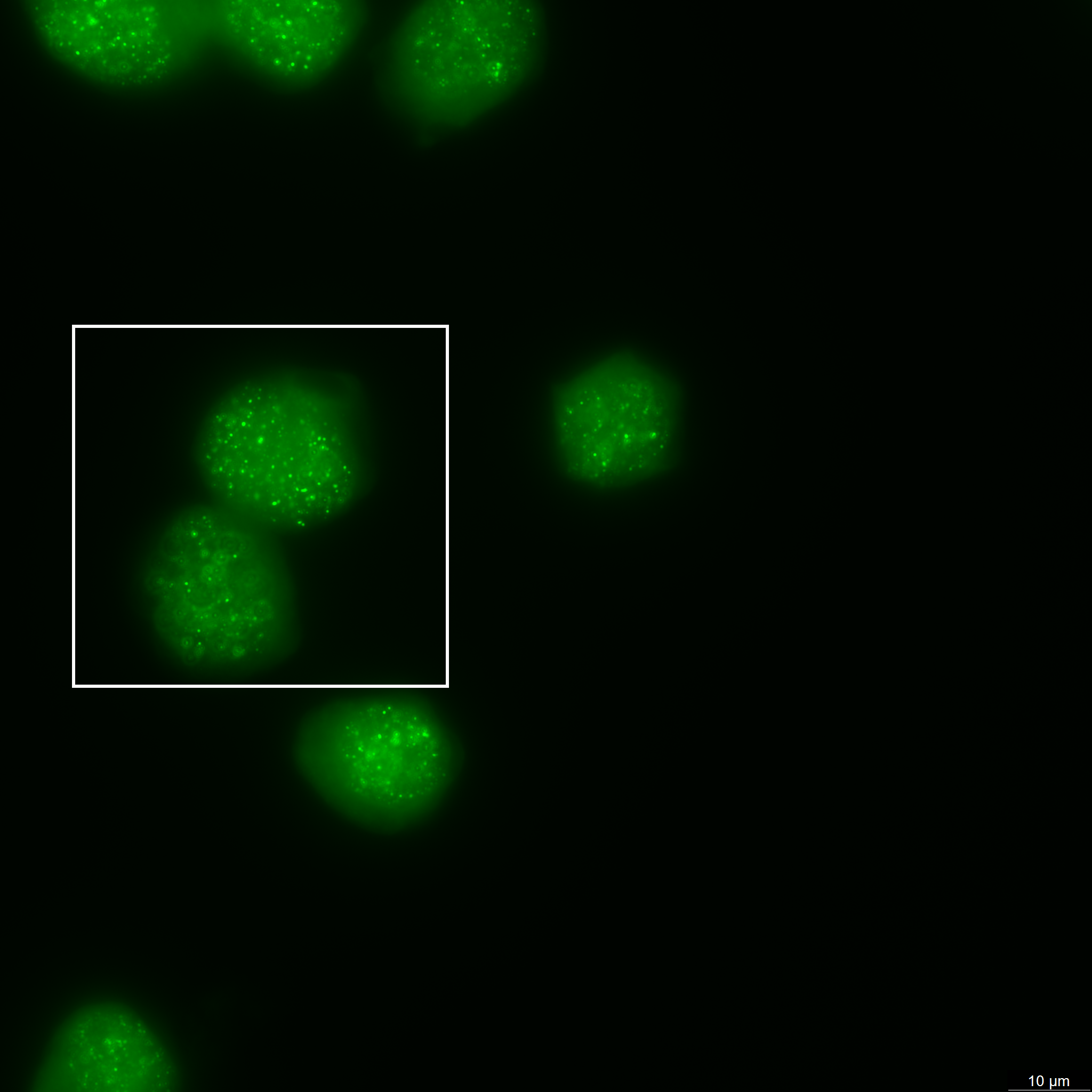

Supplement: Supplementary file 7 — Source data Fig. 6 [file 44318_2025_641_MOESM7_ESM.zip › EMBOJ-2025-120713R_SourceDataForFigure6/FIG 6C/WT/Sororin.tif]

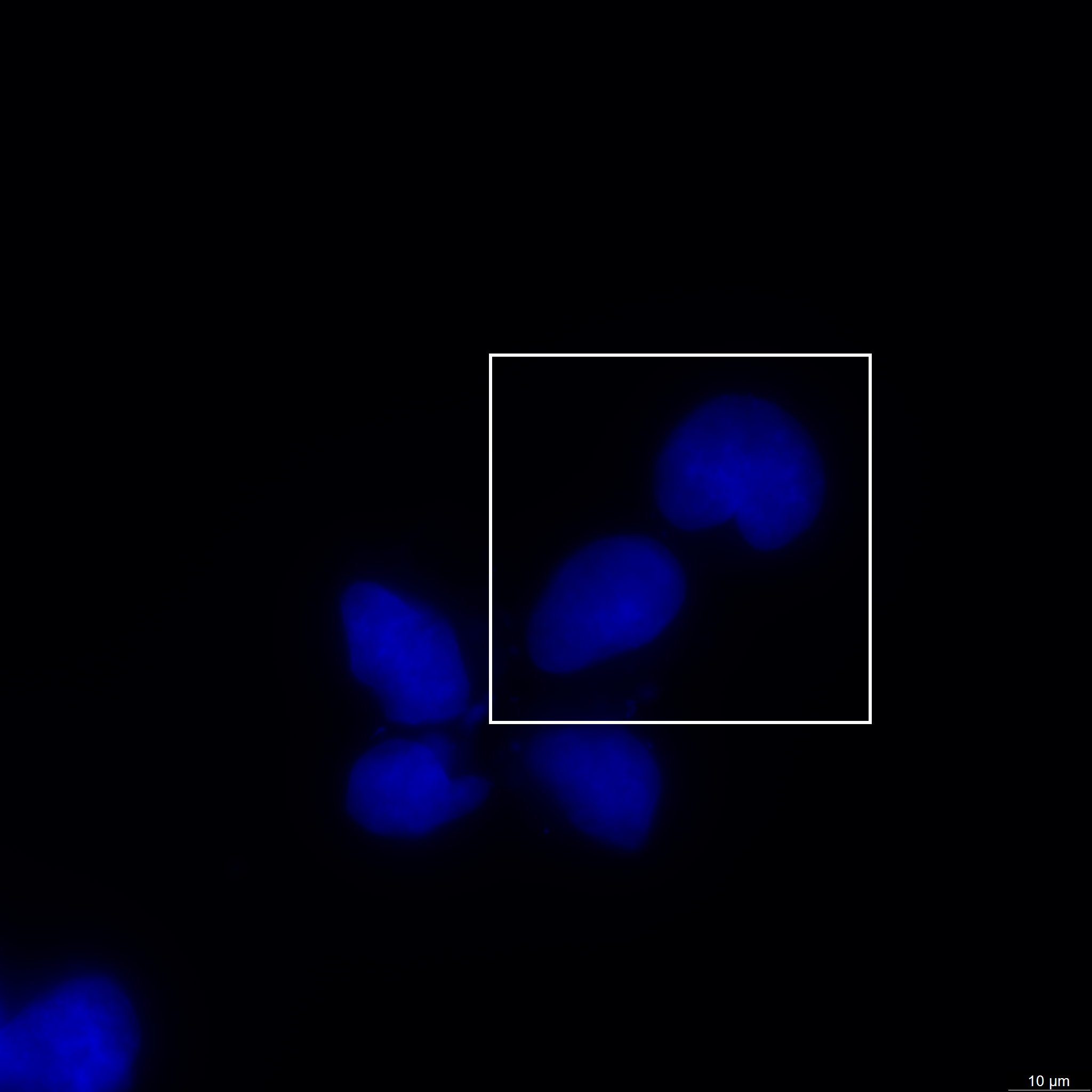

Supplement: Supplementary file 7 — Source data Fig. 6 [file 44318_2025_641_MOESM7_ESM.zip › EMBOJ-2025-120713R_SourceDataForFigure6/FIG 6C/WT+EME/DAPI.tif]

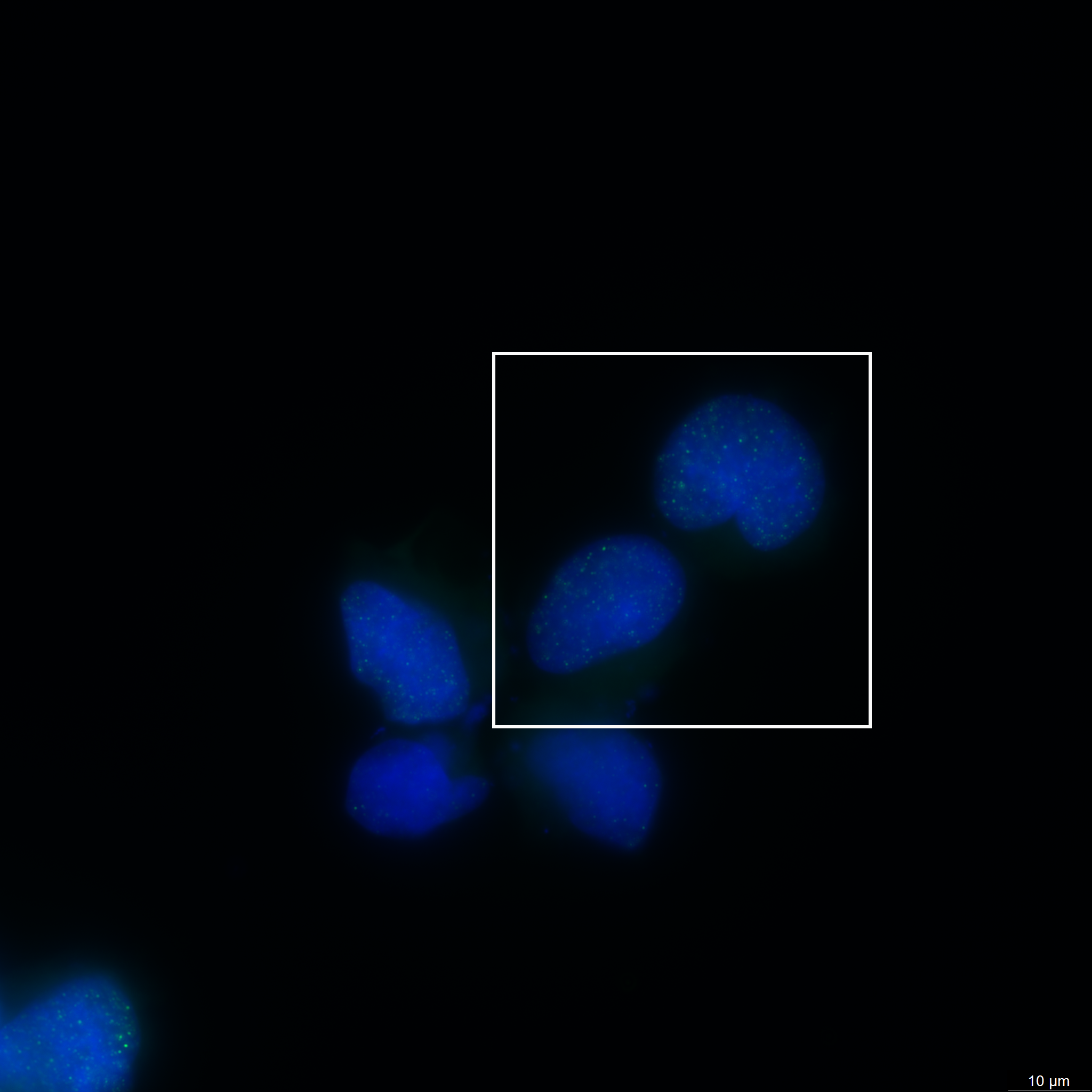

Supplement: Supplementary file 7 — Source data Fig. 6 [file 44318_2025_641_MOESM7_ESM.zip › EMBOJ-2025-120713R_SourceDataForFigure6/FIG 6C/WT+EME/merge.tif]

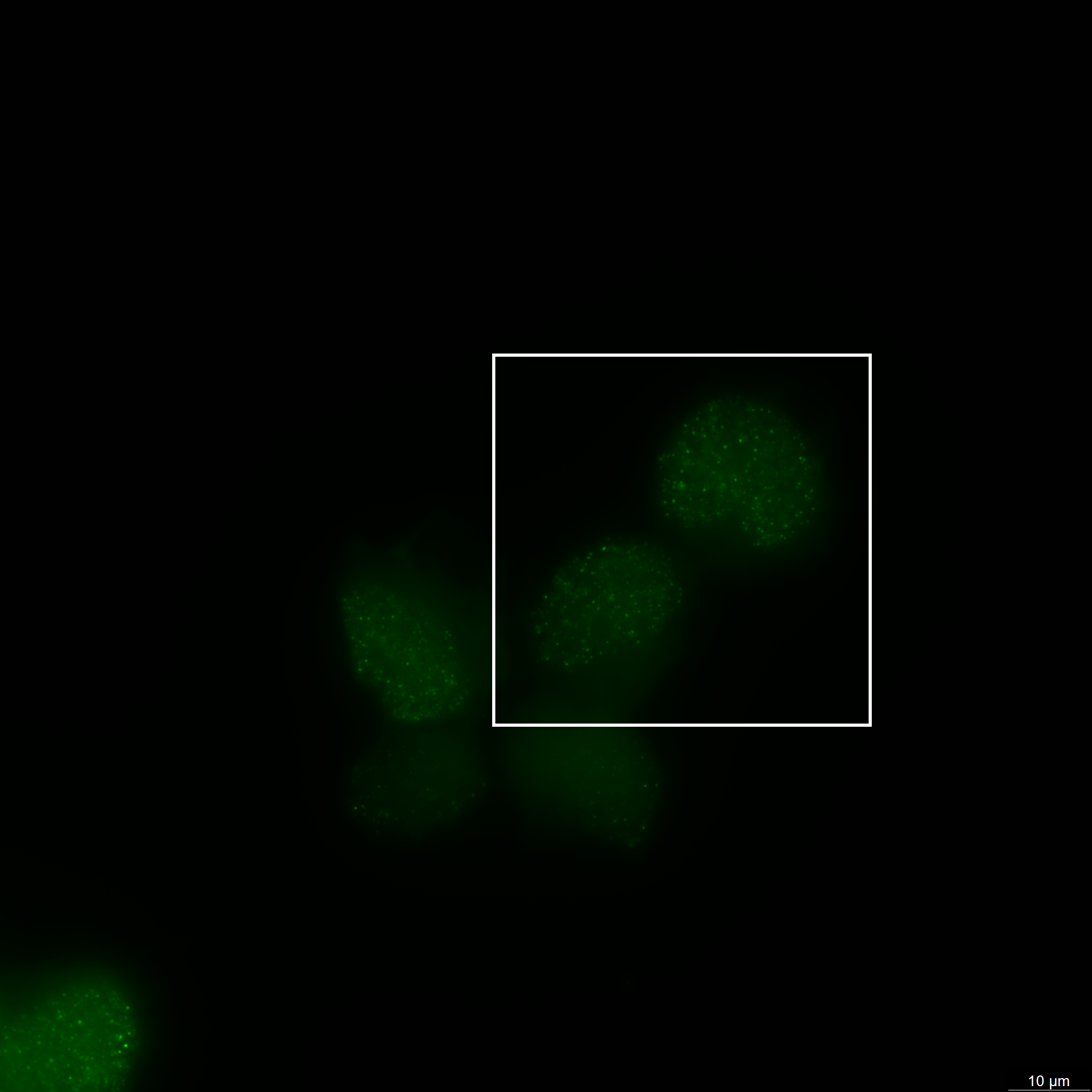

Supplement: Supplementary file 7 — Source data Fig. 6 [file 44318_2025_641_MOESM7_ESM.zip › EMBOJ-2025-120713R_SourceDataForFigure6/FIG 6C/WT+EME/Sororin.tif]

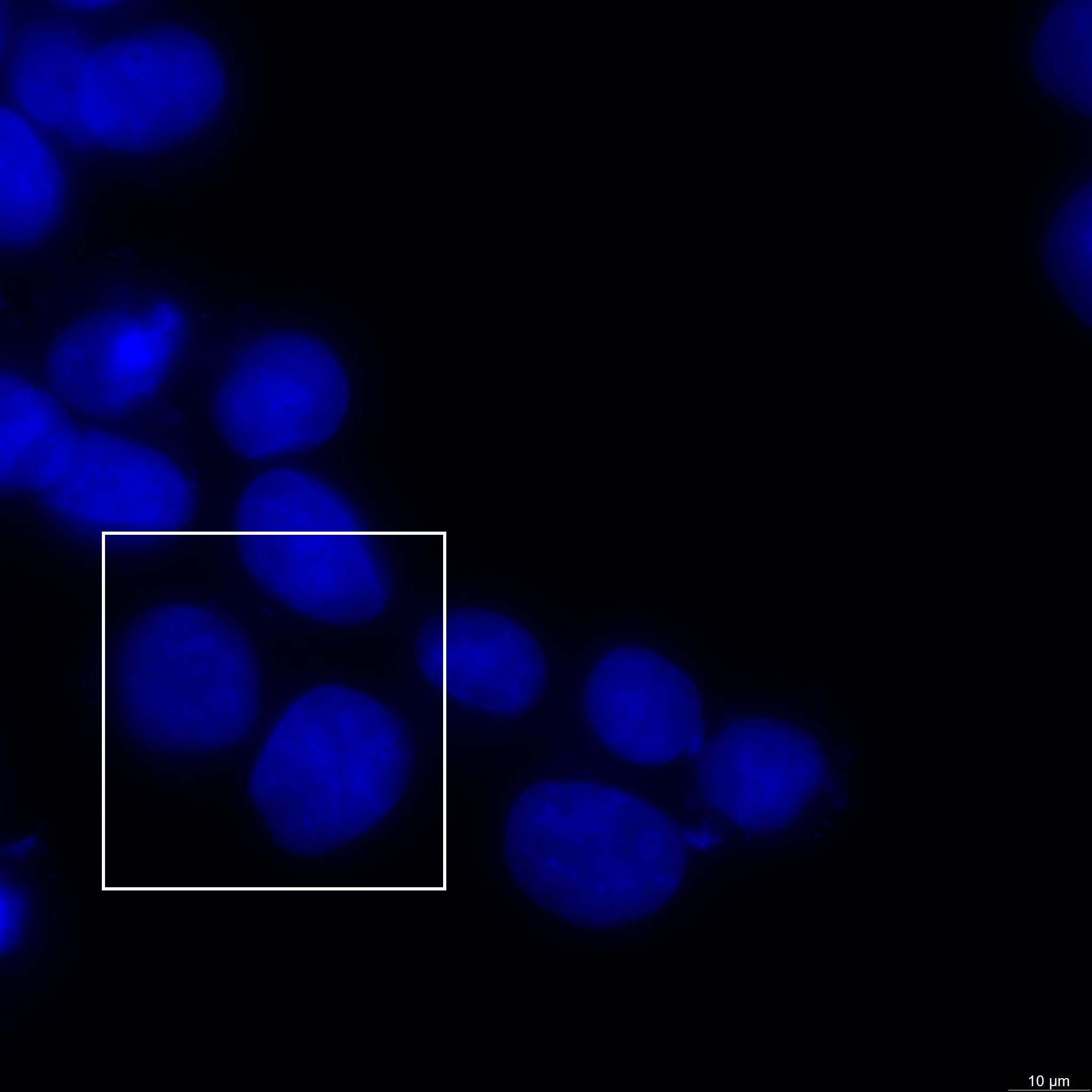

Supplement: Supplementary file 7 — Source data Fig. 6 [file 44318_2025_641_MOESM7_ESM.zip › EMBOJ-2025-120713R_SourceDataForFigure6/FIG 6C/WT+PARPi/DAPI.tif]

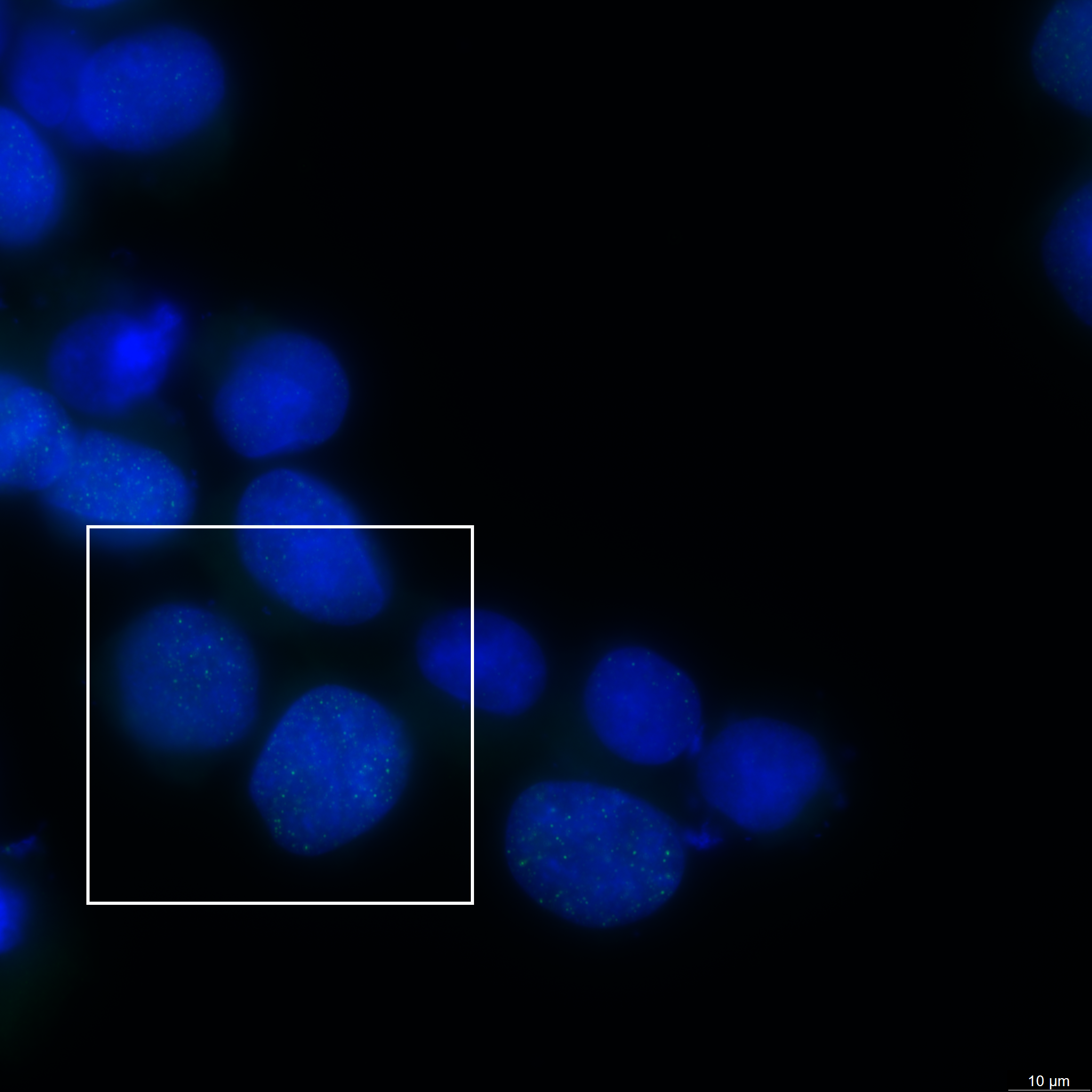

Supplement: Supplementary file 7 — Source data Fig. 6 [file 44318_2025_641_MOESM7_ESM.zip › EMBOJ-2025-120713R_SourceDataForFigure6/FIG 6C/WT+PARPi/merge.tif]

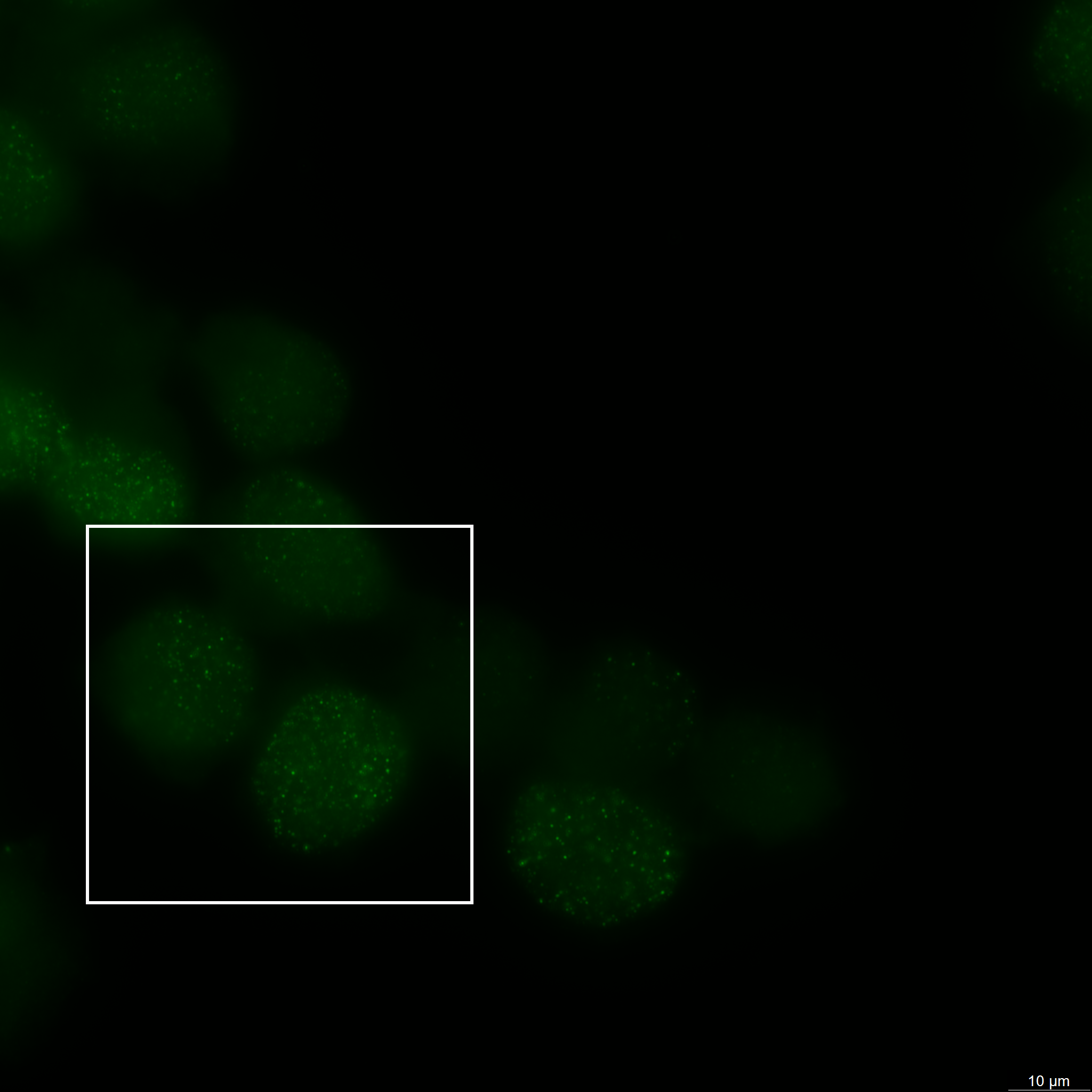

Supplement: Supplementary file 7 — Source data Fig. 6 [file 44318_2025_641_MOESM7_ESM.zip › EMBOJ-2025-120713R_SourceDataForFigure6/FIG 6C/WT+PARPi/Sororin.tif]

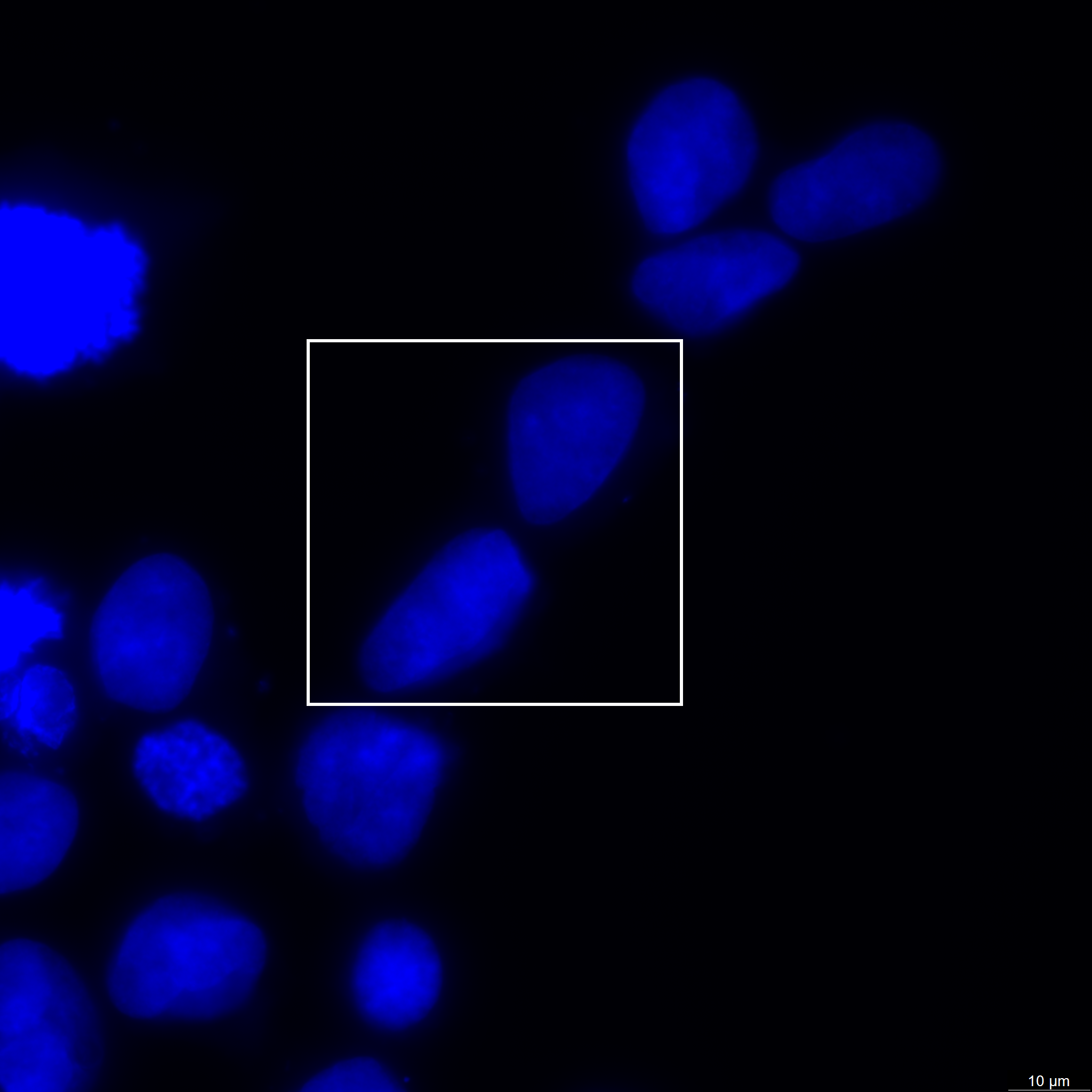

Supplement: Supplementary file 7 — Source data Fig. 6 [file 44318_2025_641_MOESM7_ESM.zip › EMBOJ-2025-120713R_SourceDataForFigure6/FIG 6C/WT+PARPi+EME/DAPI.tif]

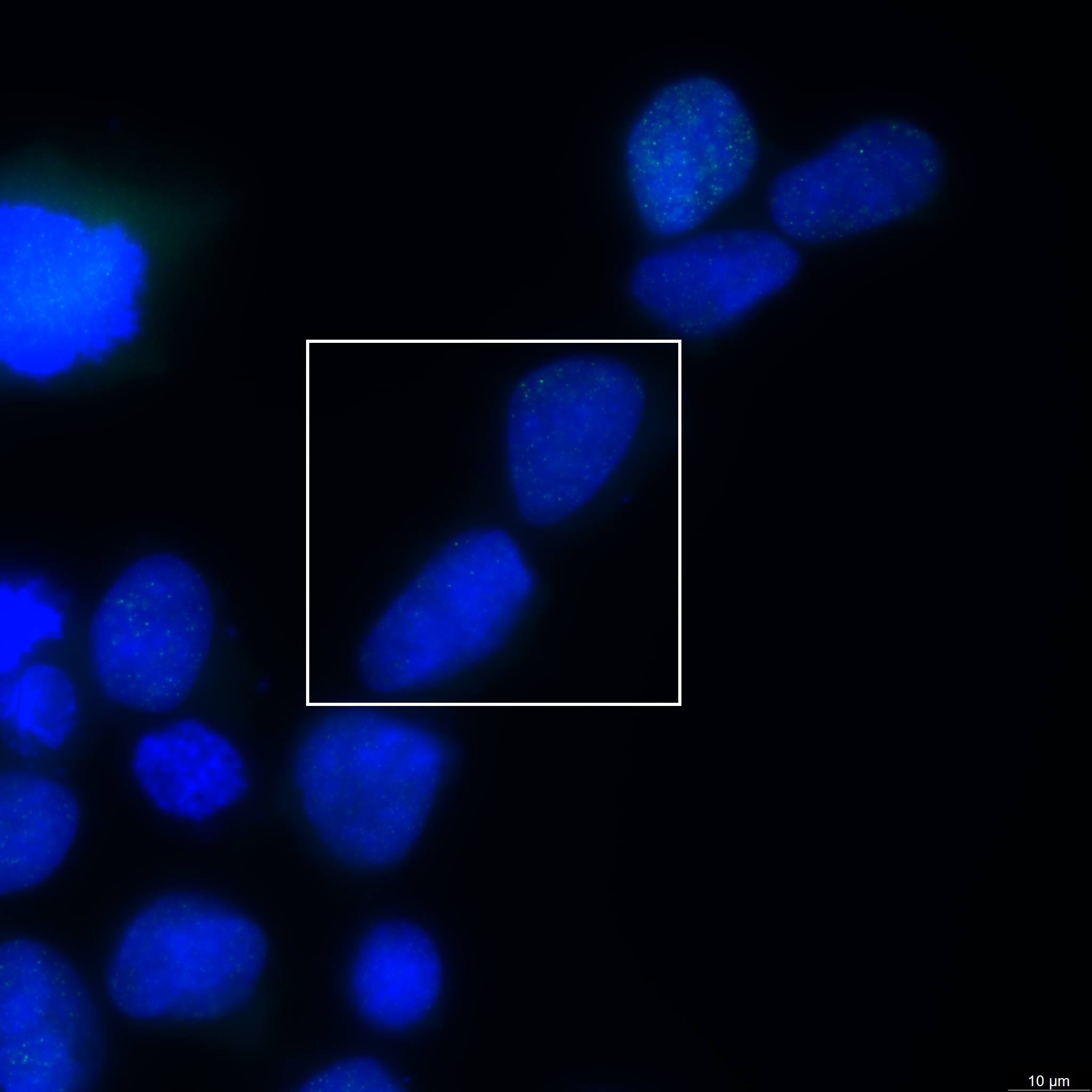

Supplement: Supplementary file 7 — Source data Fig. 6 [file 44318_2025_641_MOESM7_ESM.zip › EMBOJ-2025-120713R_SourceDataForFigure6/FIG 6C/WT+PARPi+EME/Merge.tif]

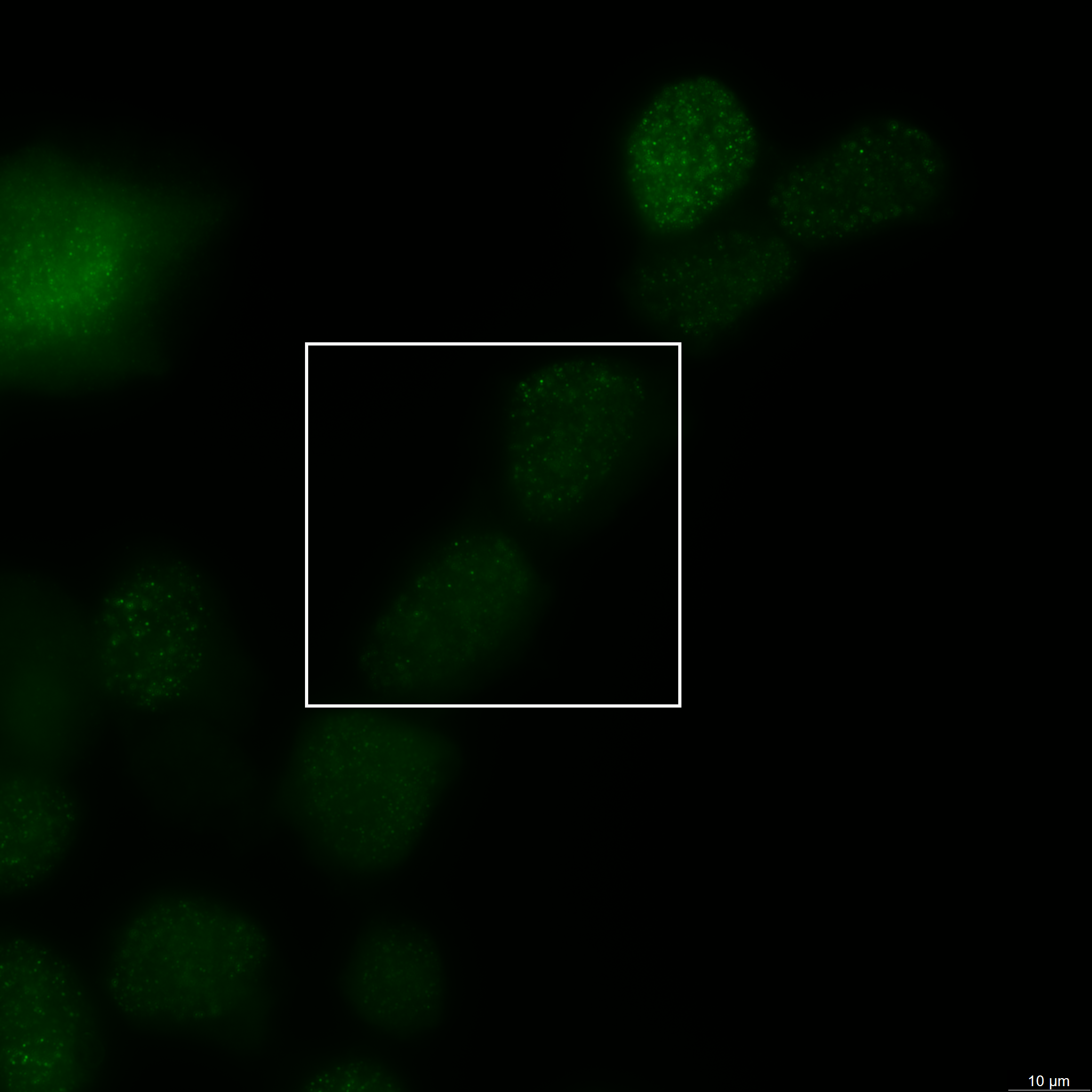

Supplement: Supplementary file 7 — Source data Fig. 6 [file 44318_2025_641_MOESM7_ESM.zip › EMBOJ-2025-120713R_SourceDataForFigure6/FIG 6C/WT+PARPi+EME/Sororin.tif]

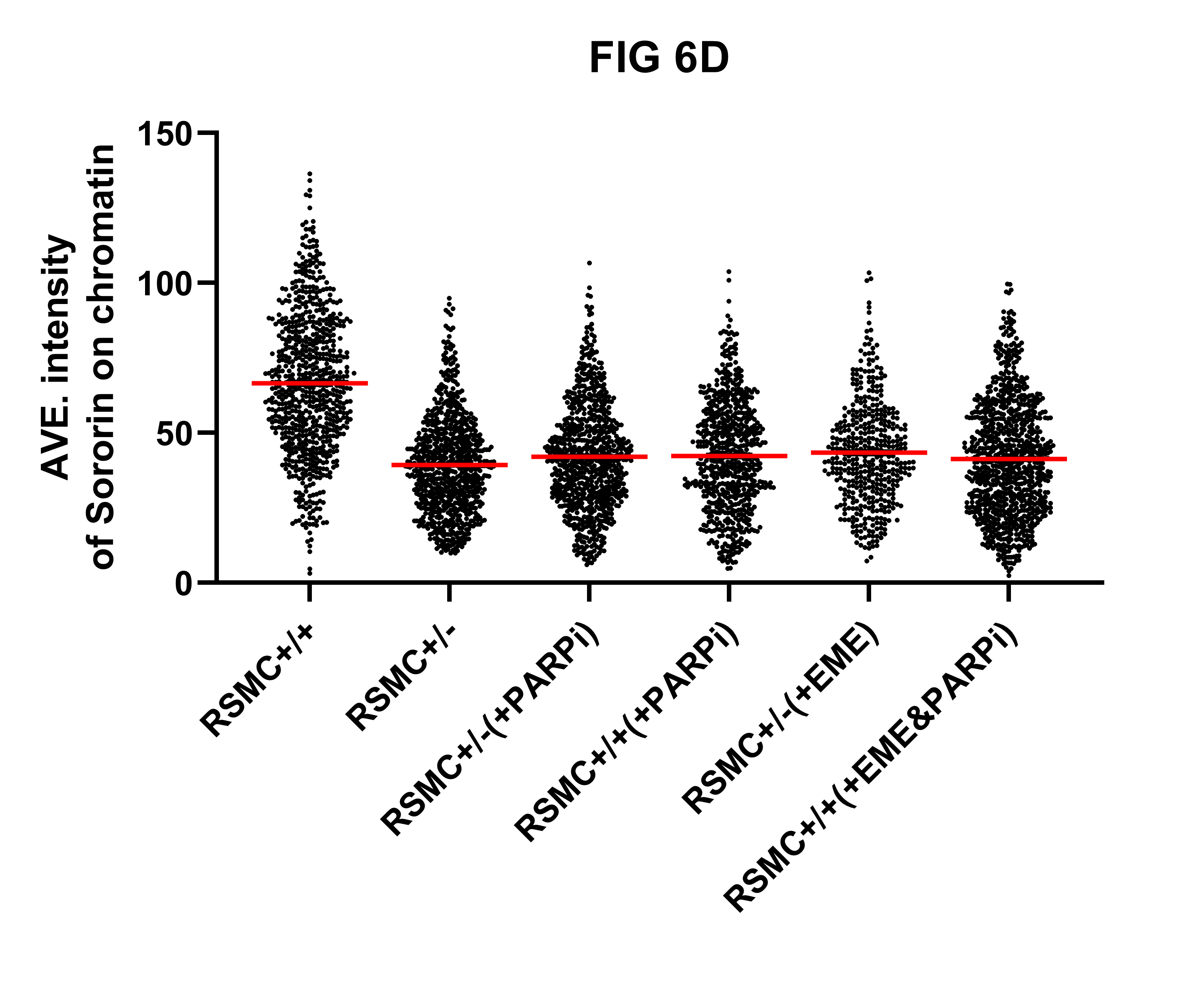

Supplement: Supplementary file 7 — Source data Fig. 6 [file 44318_2025_641_MOESM7_ESM.zip › EMBOJ-2025-120713R_SourceDataForFigure6/FIG 6D/FIG 6D before PS.tif]

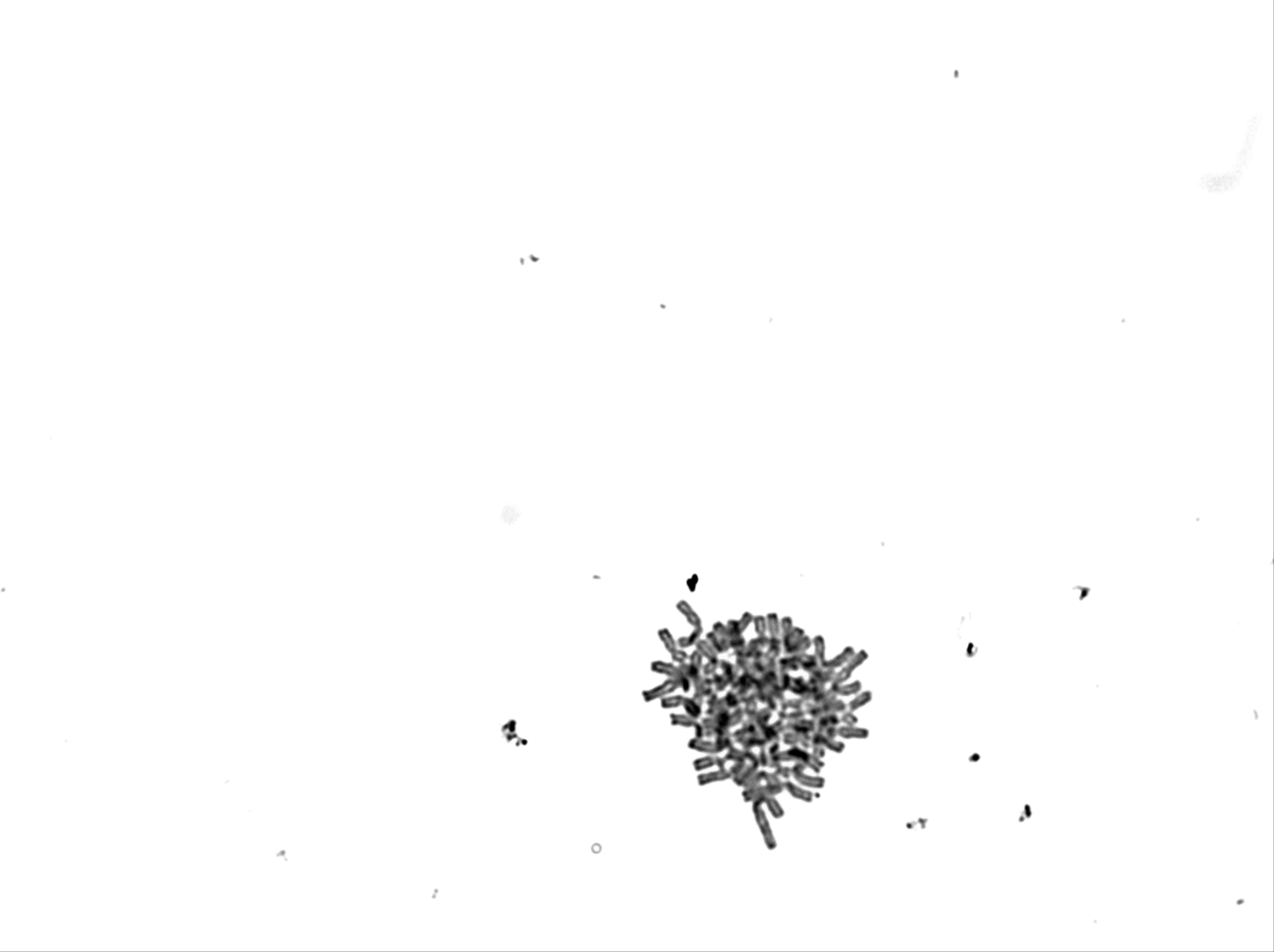

Supplement: Supplementary file 7 — Source data Fig. 6 [file 44318_2025_641_MOESM7_ESM.zip › EMBOJ-2025-120713R_SourceDataForFigure6/FIG 6E/DMSO+RSMC.tif]

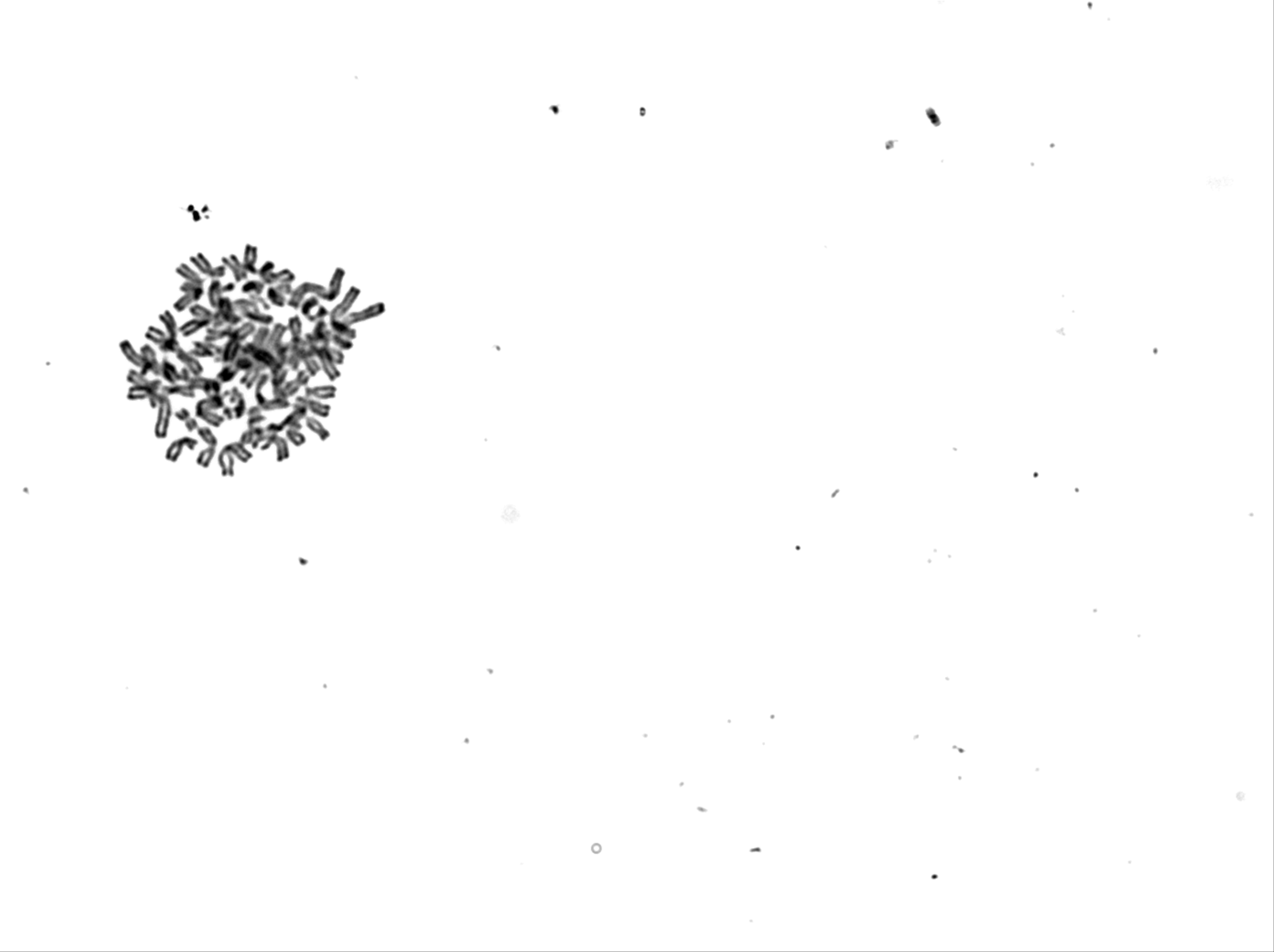

Supplement: Supplementary file 7 — Source data Fig. 6 [file 44318_2025_641_MOESM7_ESM.zip › EMBOJ-2025-120713R_SourceDataForFigure6/FIG 6E/DMSO+Vector.tif]

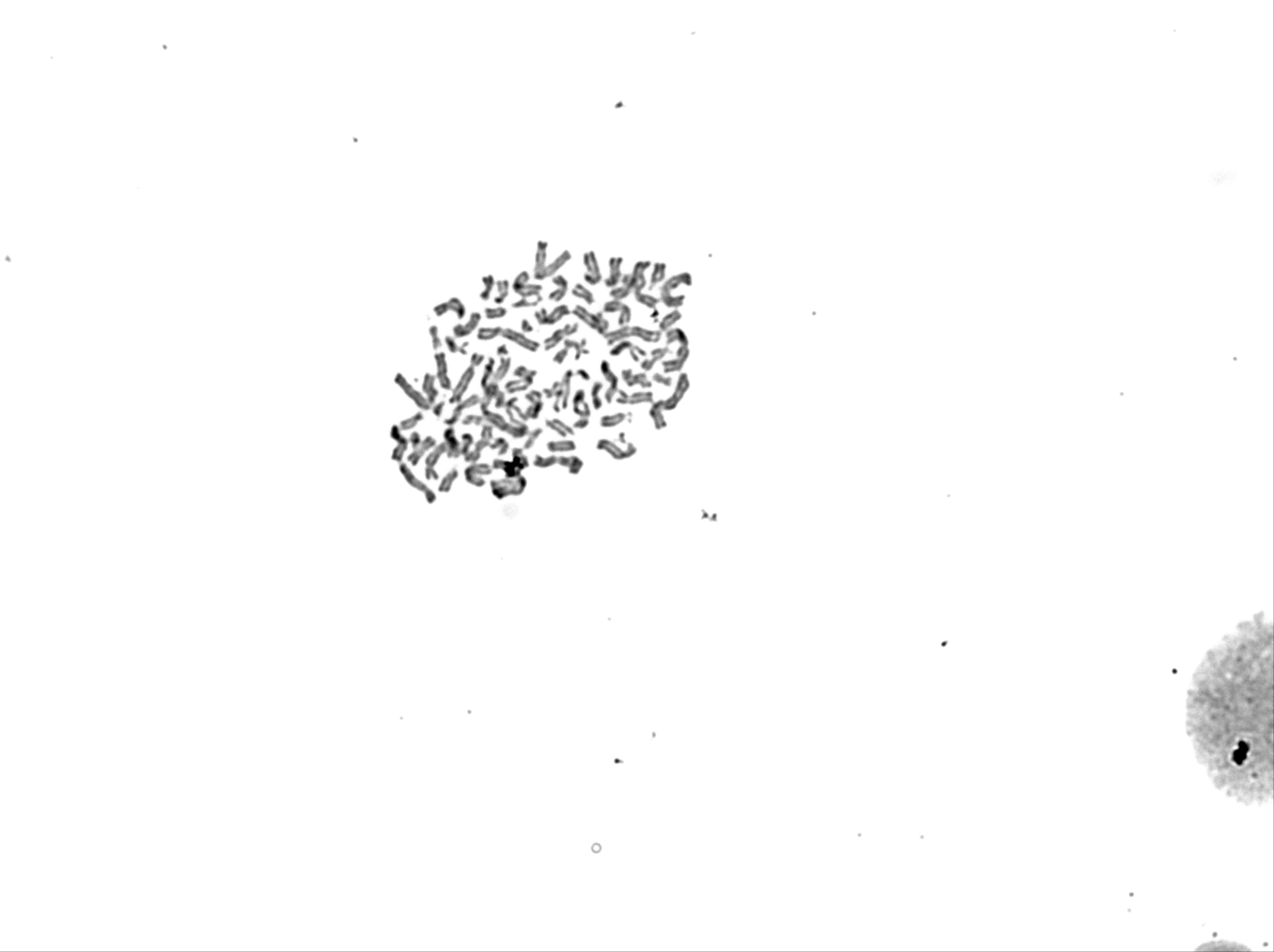

Supplement: Supplementary file 7 — Source data Fig. 6 [file 44318_2025_641_MOESM7_ESM.zip › EMBOJ-2025-120713R_SourceDataForFigure6/FIG 6E/Olaparib+RSMC.tif]

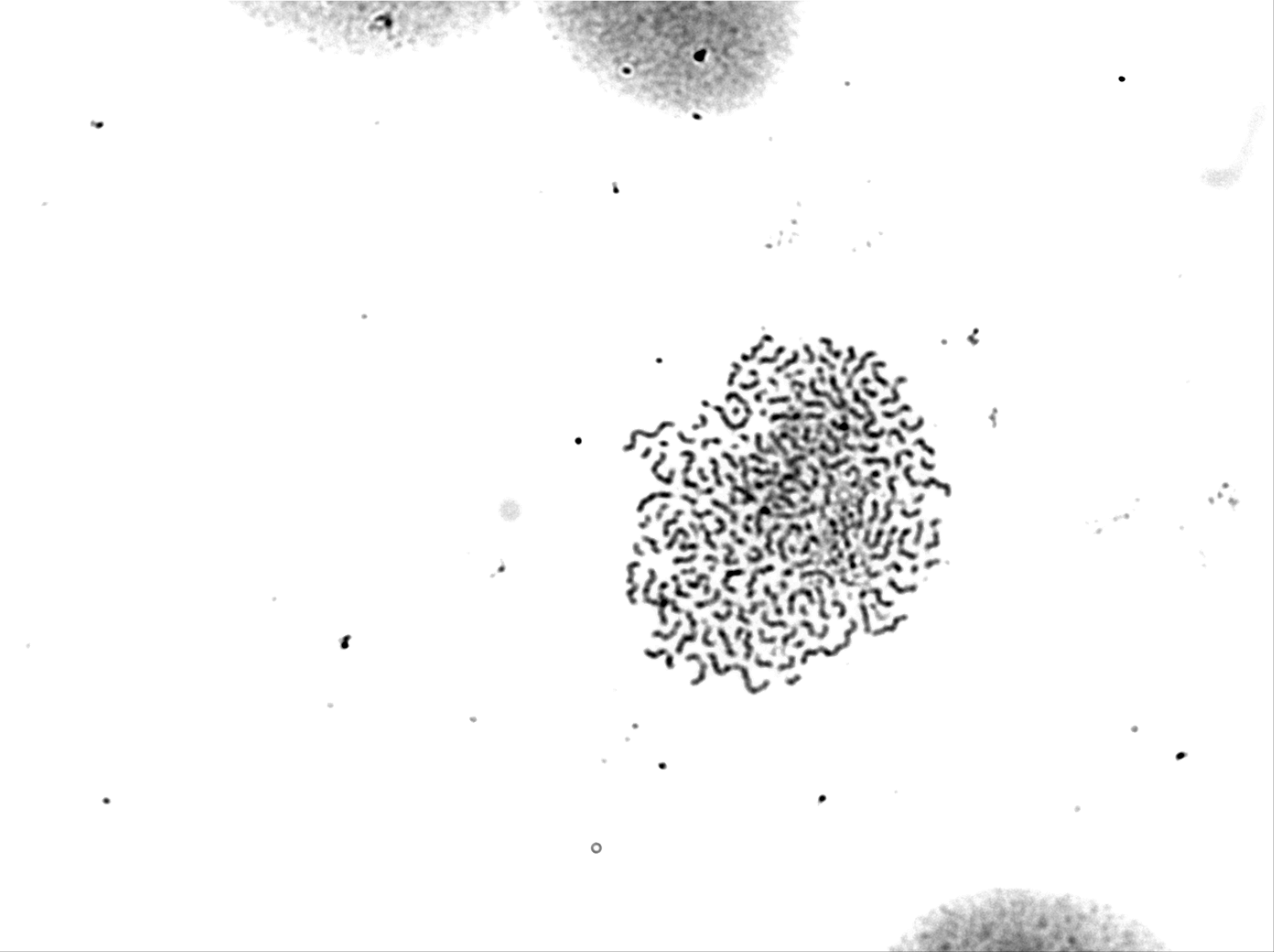

Supplement: Supplementary file 7 — Source data Fig. 6 [file 44318_2025_641_MOESM7_ESM.zip › EMBOJ-2025-120713R_SourceDataForFigure6/FIG 6E/Olaparib-Vector.tif]

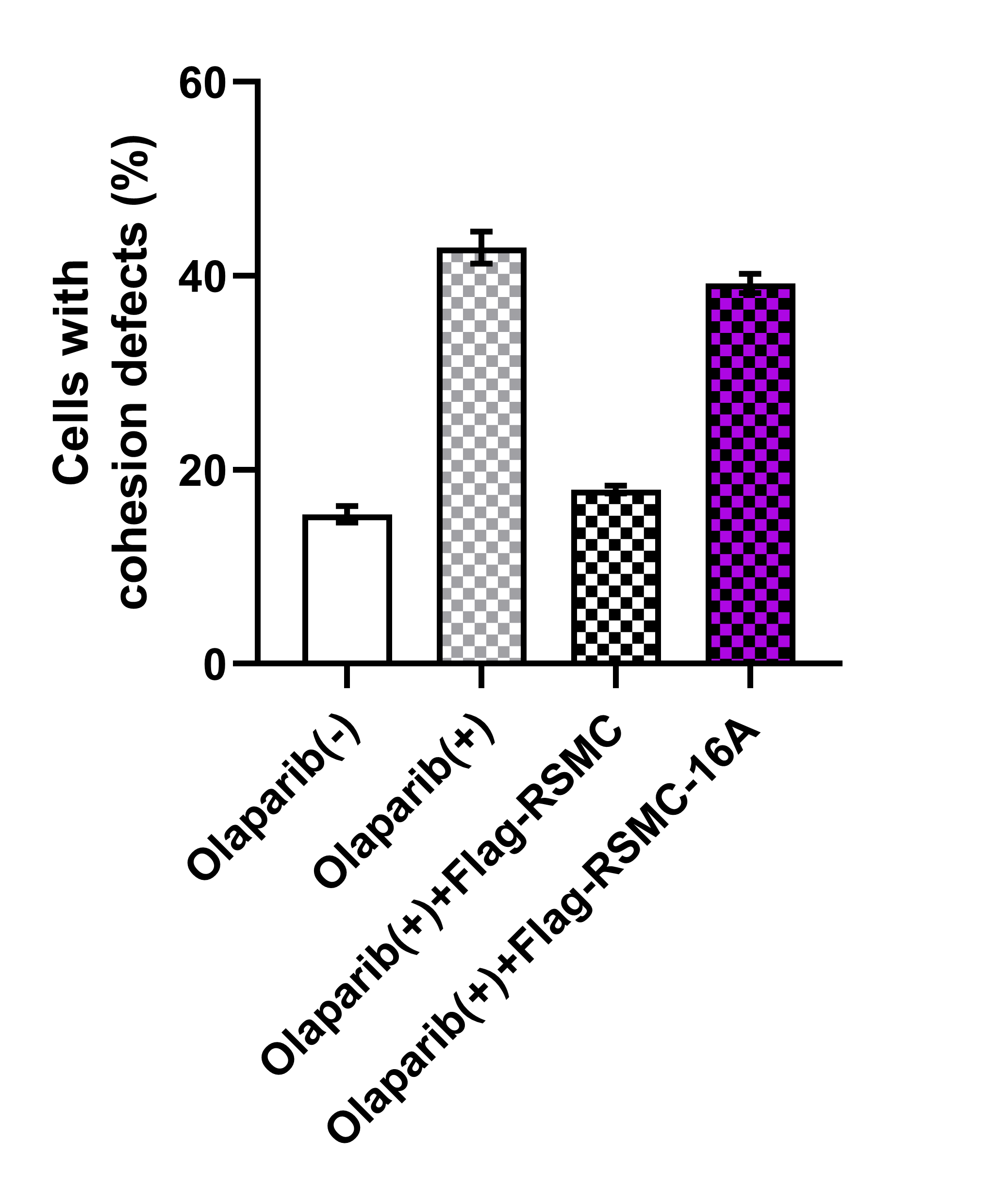

Supplement: Supplementary file 7 — Source data Fig. 6 [file 44318_2025_641_MOESM7_ESM.zip › EMBOJ-2025-120713R_SourceDataForFigure6/FIG 6F/FIG 6F before PS.tif]

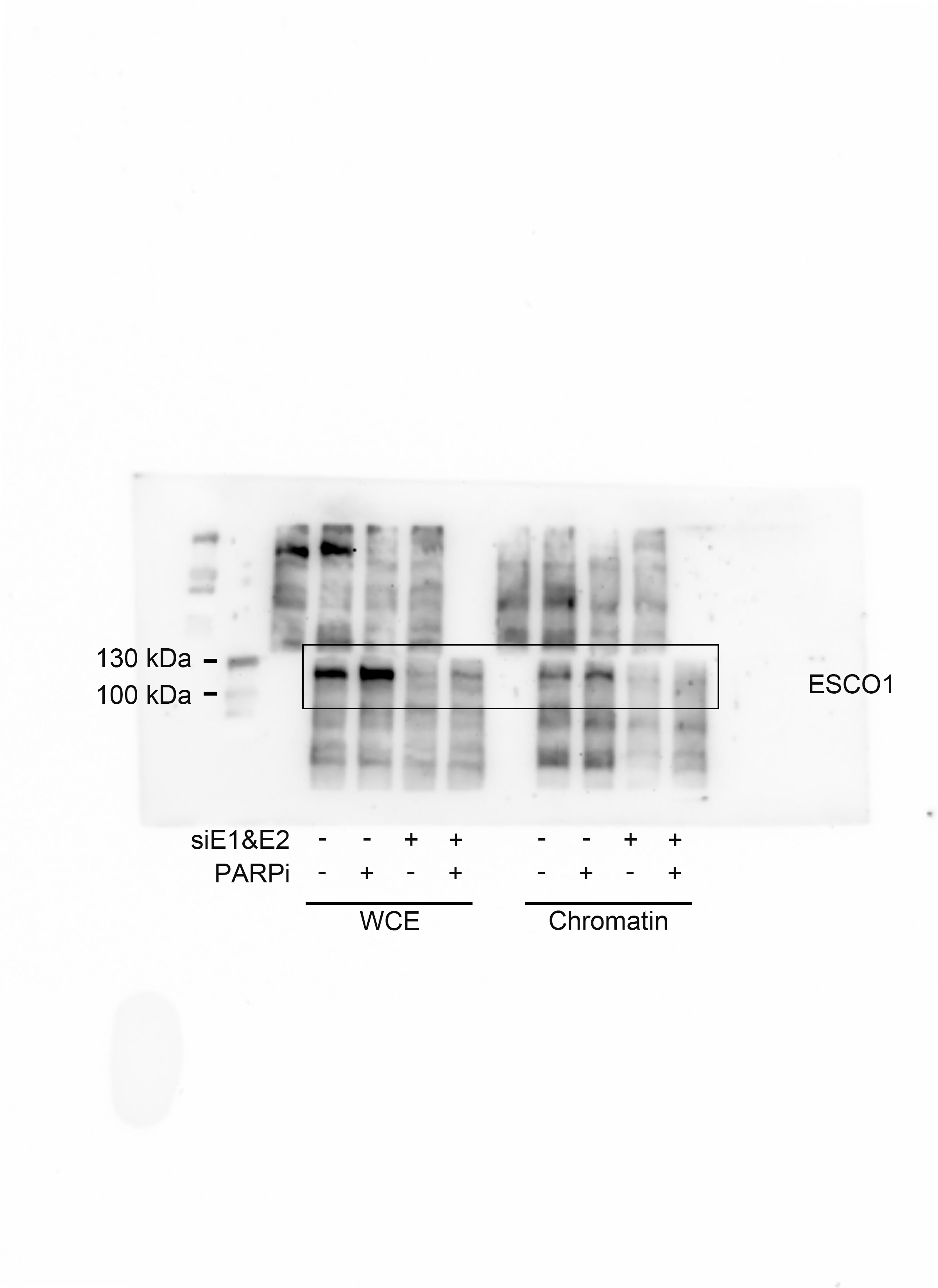

Supplement: Supplementary file 7 — Source data Fig. 6 [file 44318_2025_641_MOESM7_ESM.zip › EMBOJ-2025-120713R_SourceDataForFigure6/FIG 6G/ESCO1 RAW data (with marker).tif]

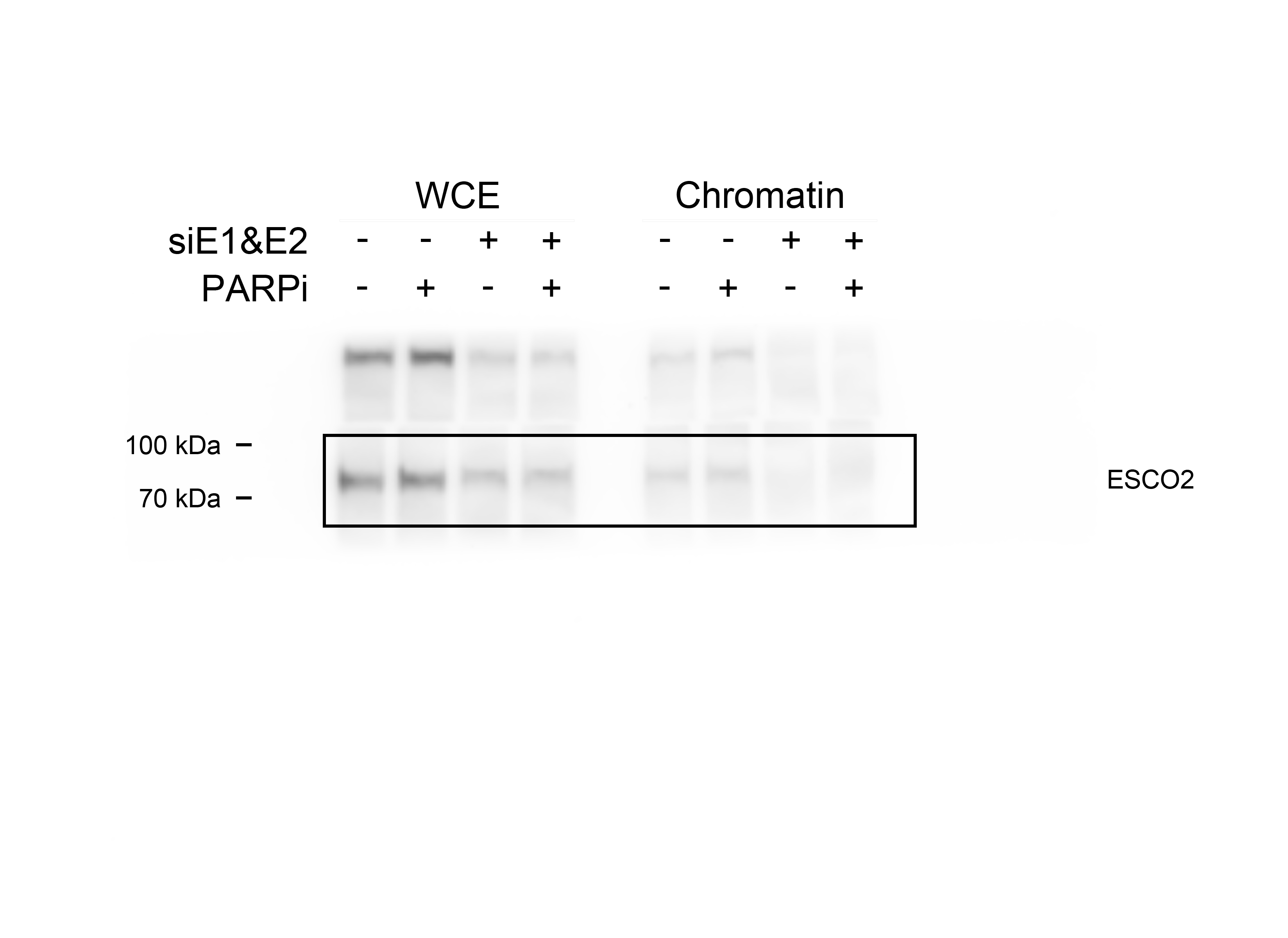

Supplement: Supplementary file 7 — Source data Fig. 6 [file 44318_2025_641_MOESM7_ESM.zip › EMBOJ-2025-120713R_SourceDataForFigure6/FIG 6G/ESCO2 RAW data.tif]

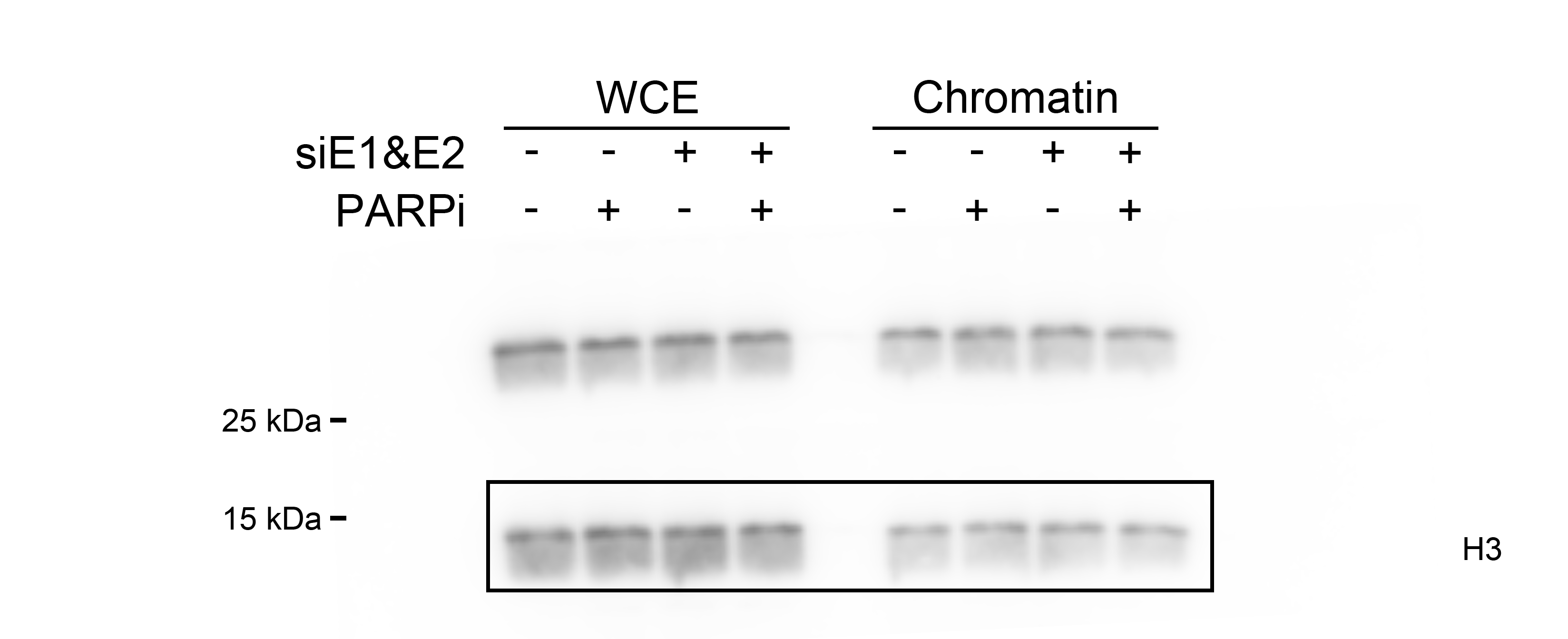

Supplement: Supplementary file 7 — Source data Fig. 6 [file 44318_2025_641_MOESM7_ESM.zip › EMBOJ-2025-120713R_SourceDataForFigure6/FIG 6G/H3 RAW data.tif]

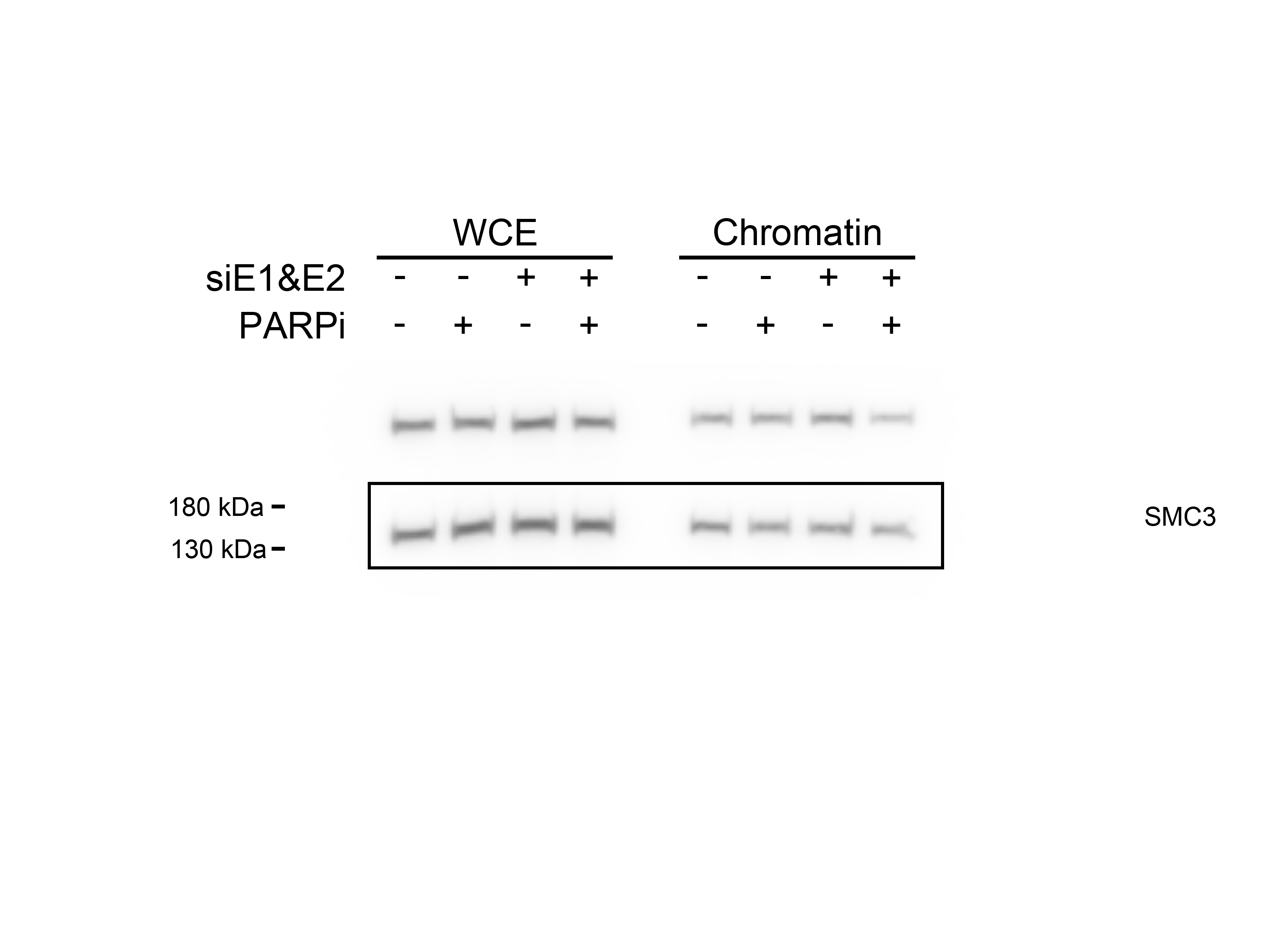

Supplement: Supplementary file 7 — Source data Fig. 6 [file 44318_2025_641_MOESM7_ESM.zip › EMBOJ-2025-120713R_SourceDataForFigure6/FIG 6G/SMC3 RAW data.tif]

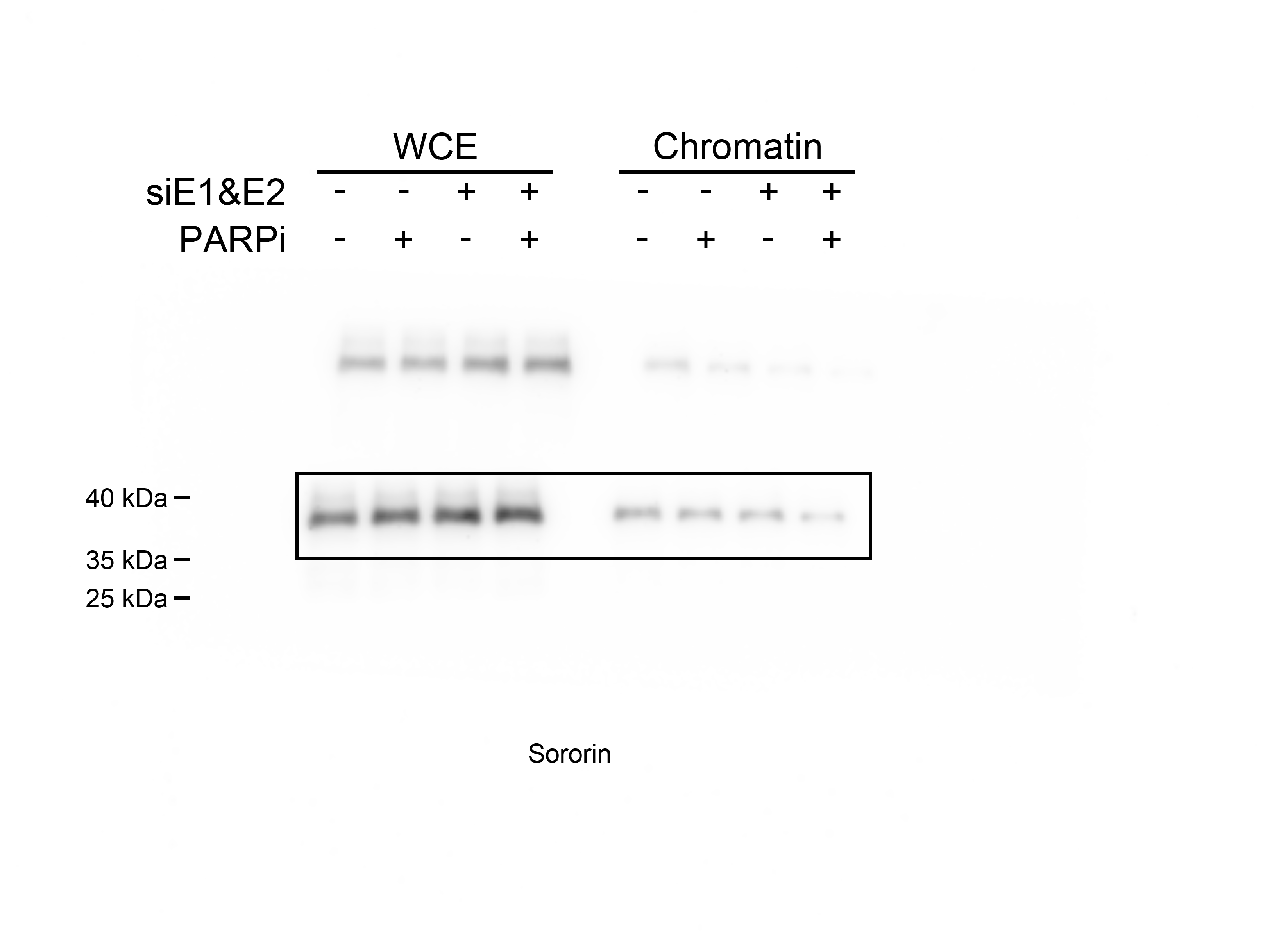

Supplement: Supplementary file 7 — Source data Fig. 6 [file 44318_2025_641_MOESM7_ESM.zip › EMBOJ-2025-120713R_SourceDataForFigure6/FIG 6G/Sororin RAW data.tif]

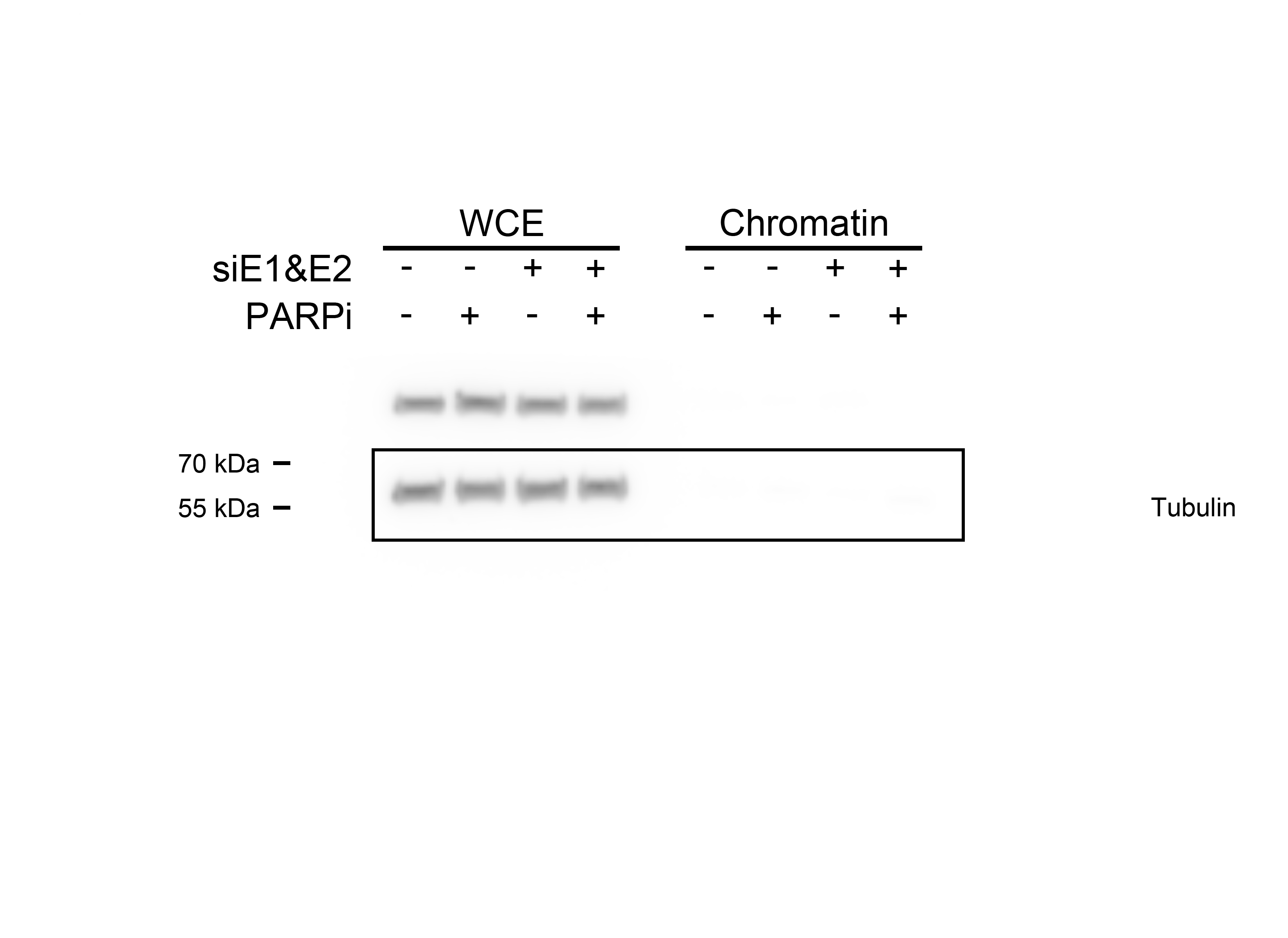

Supplement: Supplementary file 7 — Source data Fig. 6 [file 44318_2025_641_MOESM7_ESM.zip › EMBOJ-2025-120713R_SourceDataForFigure6/FIG 6G/Tubulin RAW data.tif]

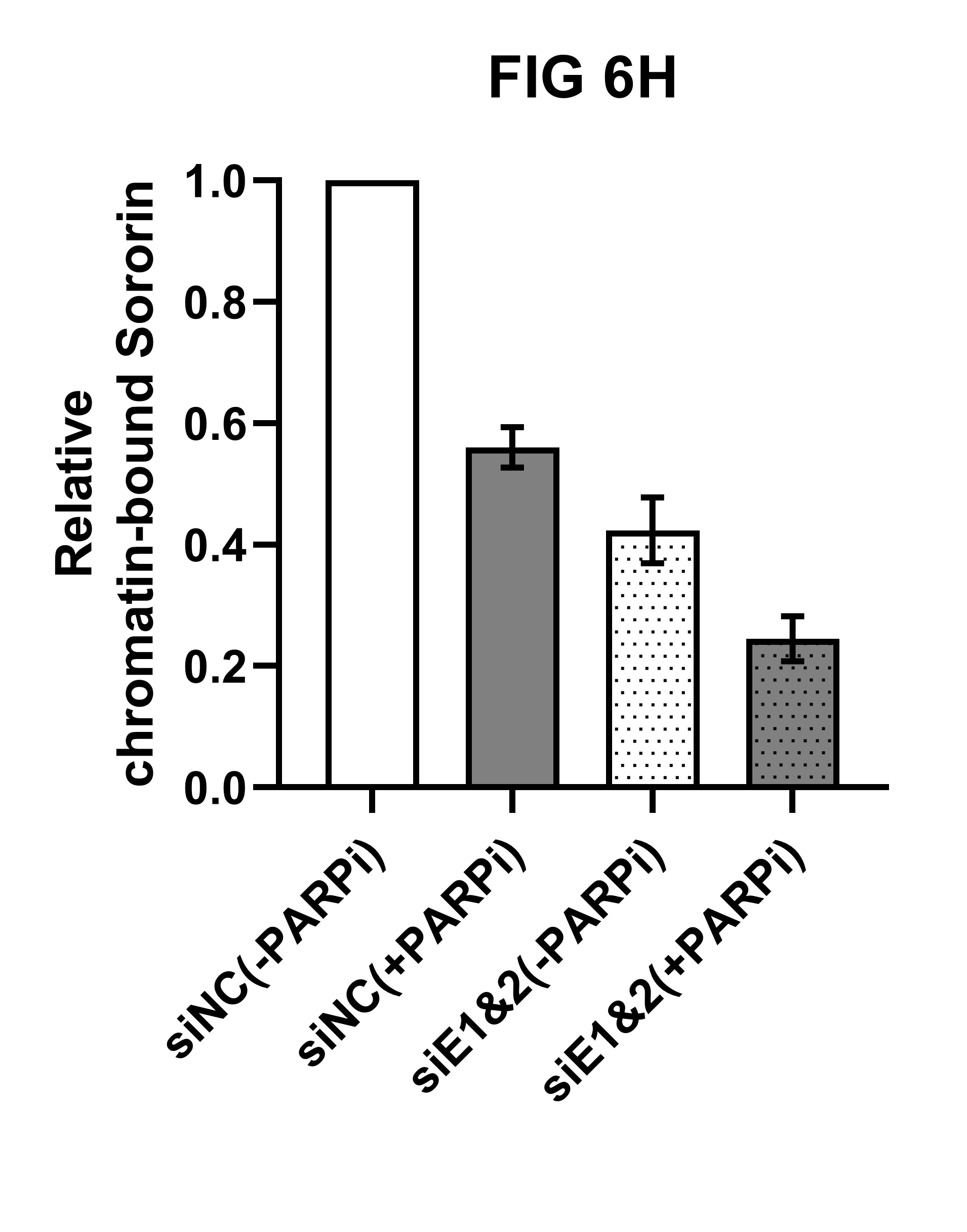

Supplement: Supplementary file 7 — Source data Fig. 6 [file 44318_2025_641_MOESM7_ESM.zip › EMBOJ-2025-120713R_SourceDataForFigure6/FIG 6H/FIG 6H before PS.tif]

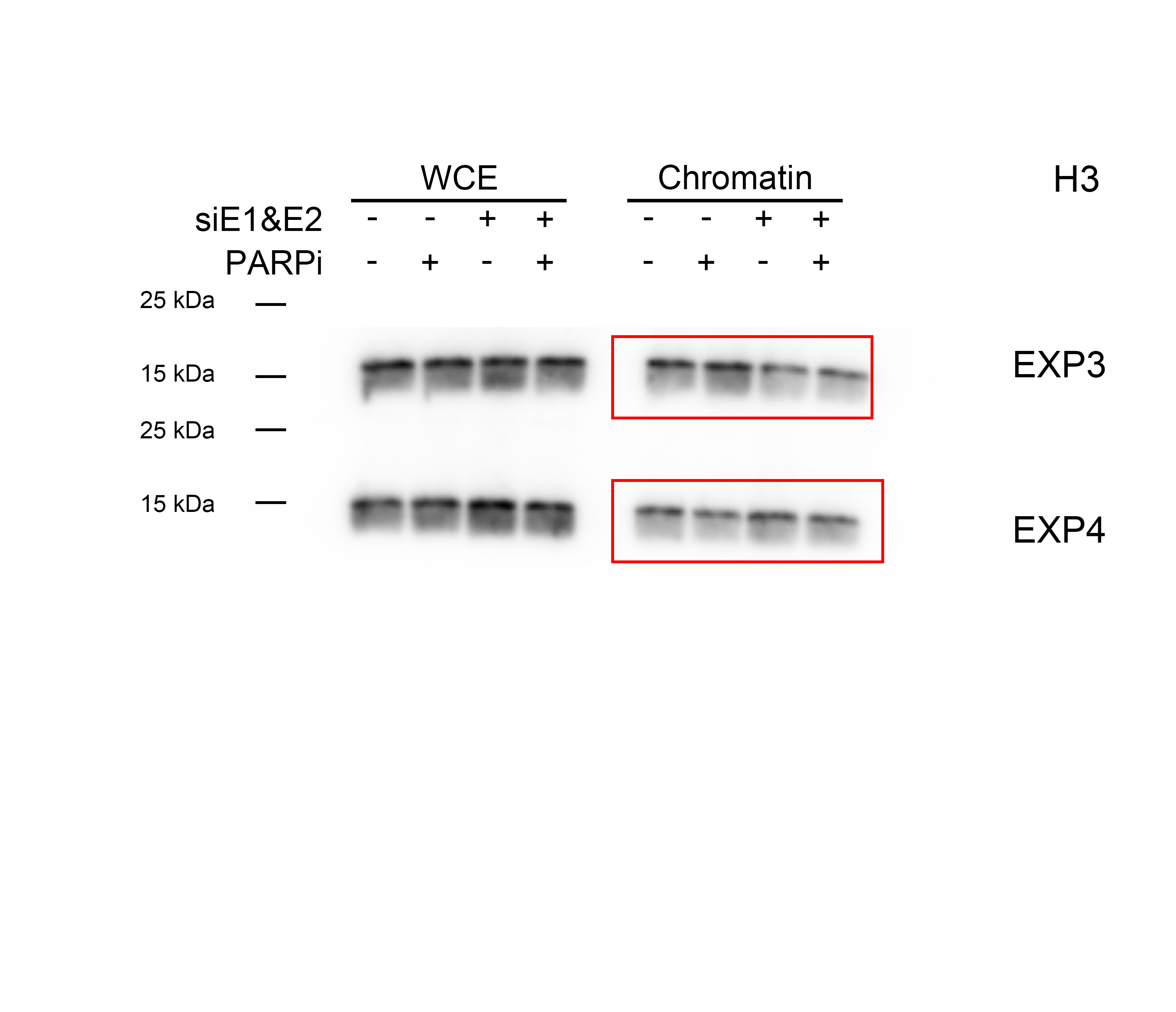

Supplement: Supplementary file 7 — Source data Fig. 6 [file 44318_2025_641_MOESM7_ESM.zip › EMBOJ-2025-120713R_SourceDataForFigure6/FIG 6H/Used for quantification/EXP3 & EXP4/H3 RAW data.tif]

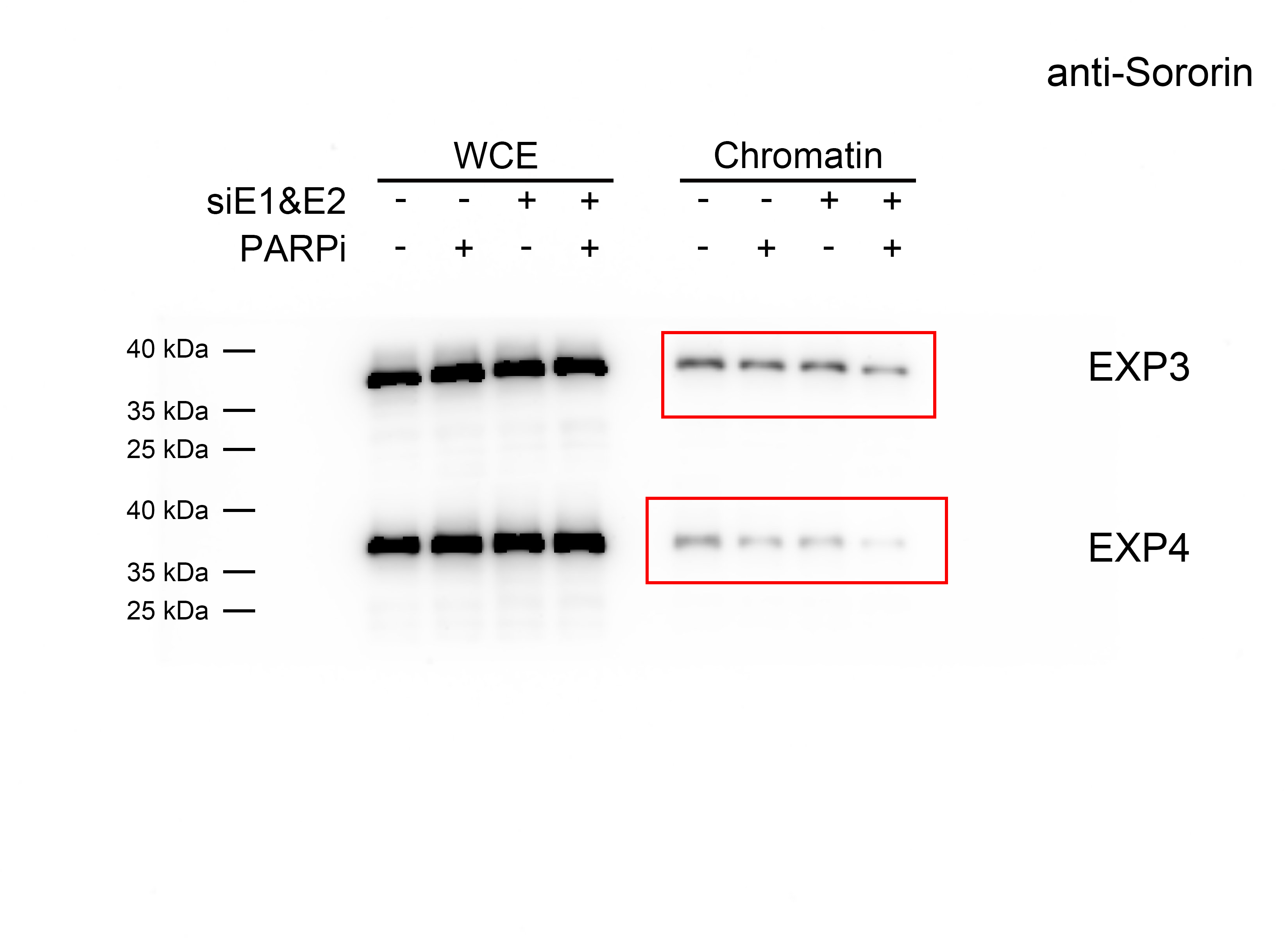

Supplement: Supplementary file 7 — Source data Fig. 6 [file 44318_2025_641_MOESM7_ESM.zip › EMBOJ-2025-120713R_SourceDataForFigure6/FIG 6H/Used for quantification/EXP3 & EXP4/Sororin RAW data.tif]

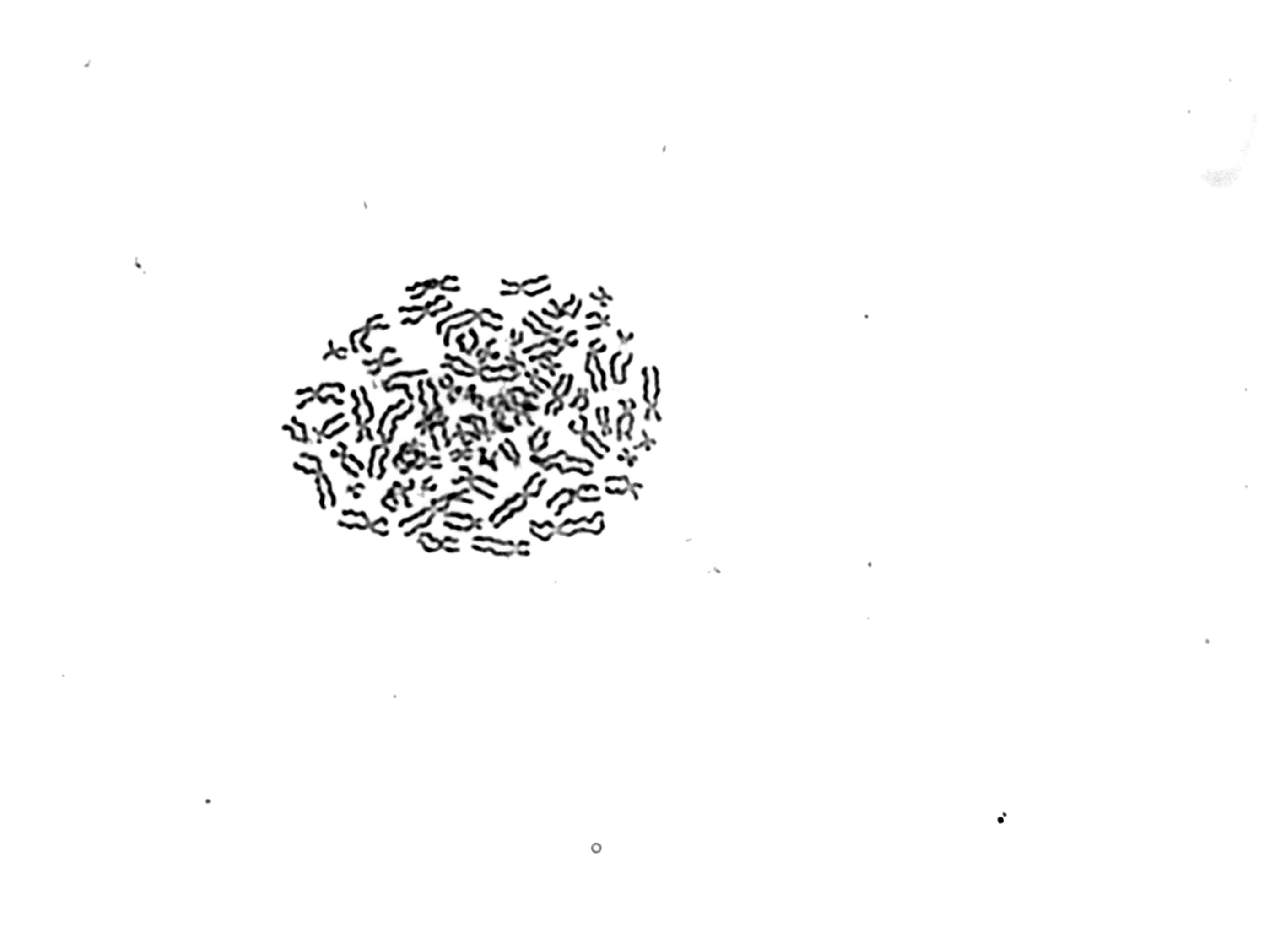

Supplement: Supplementary file 8 — Source data Fig. 7 [file 44318_2025_641_MOESM8_ESM.zip › EMBOJ-2025-120713R_SourceDataForFigure7/FIG 7A/siNC.tif]

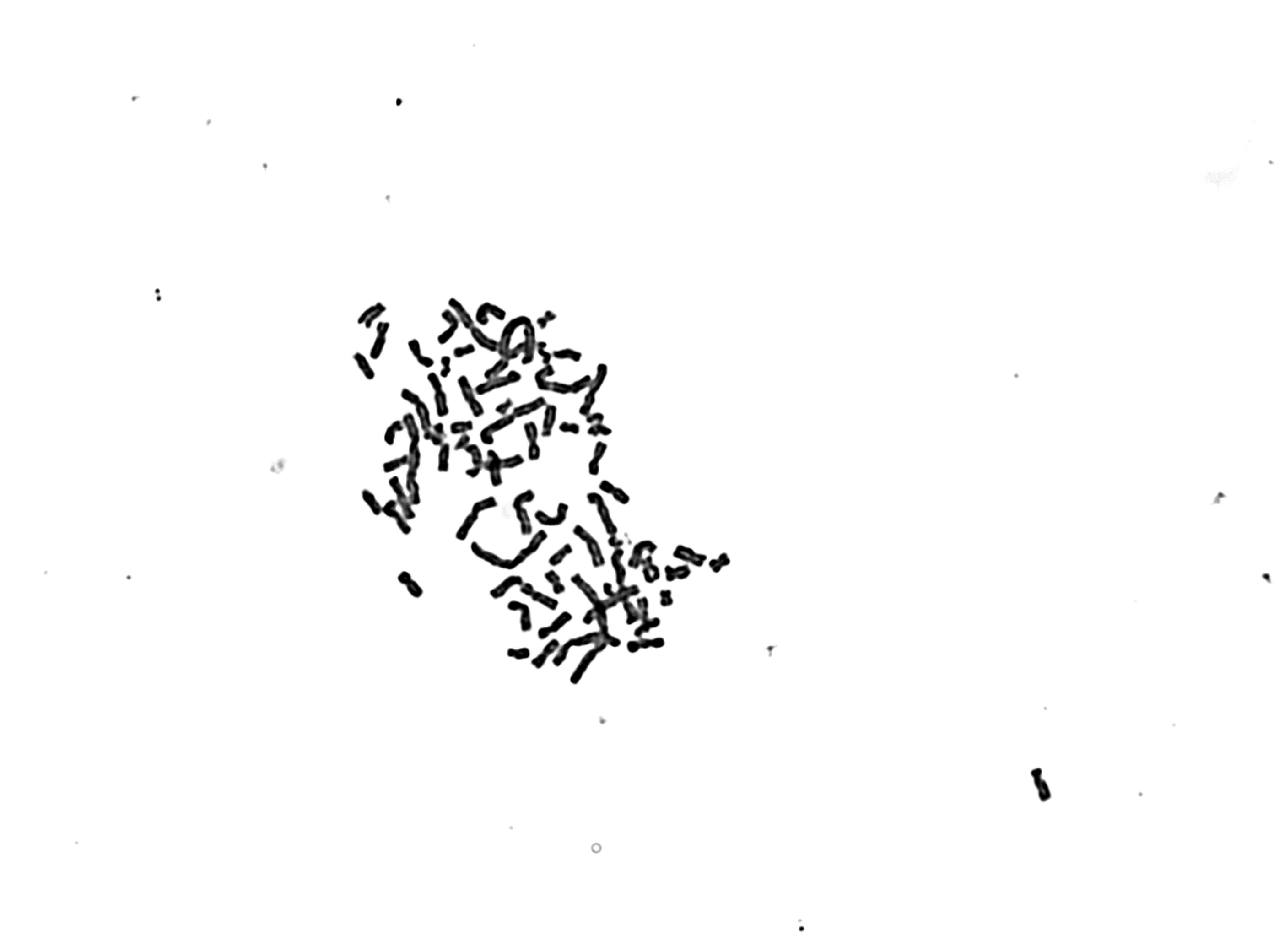

Supplement: Supplementary file 8 — Source data Fig. 7 [file 44318_2025_641_MOESM8_ESM.zip › EMBOJ-2025-120713R_SourceDataForFigure7/FIG 7A/siRSMC&WAPL.tif]

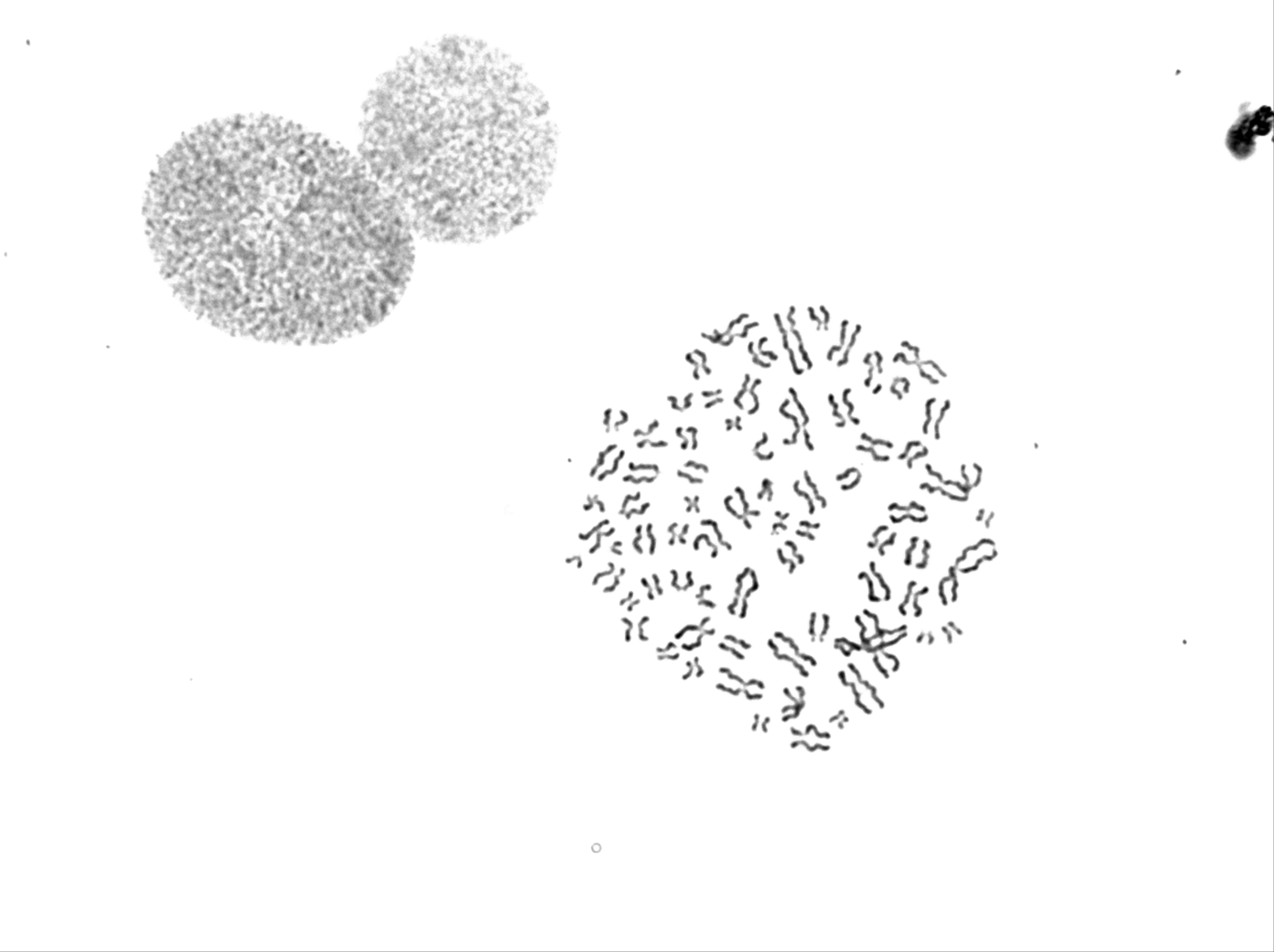

Supplement: Supplementary file 8 — Source data Fig. 7 [file 44318_2025_641_MOESM8_ESM.zip › EMBOJ-2025-120713R_SourceDataForFigure7/FIG 7A/siRSMC.tif]

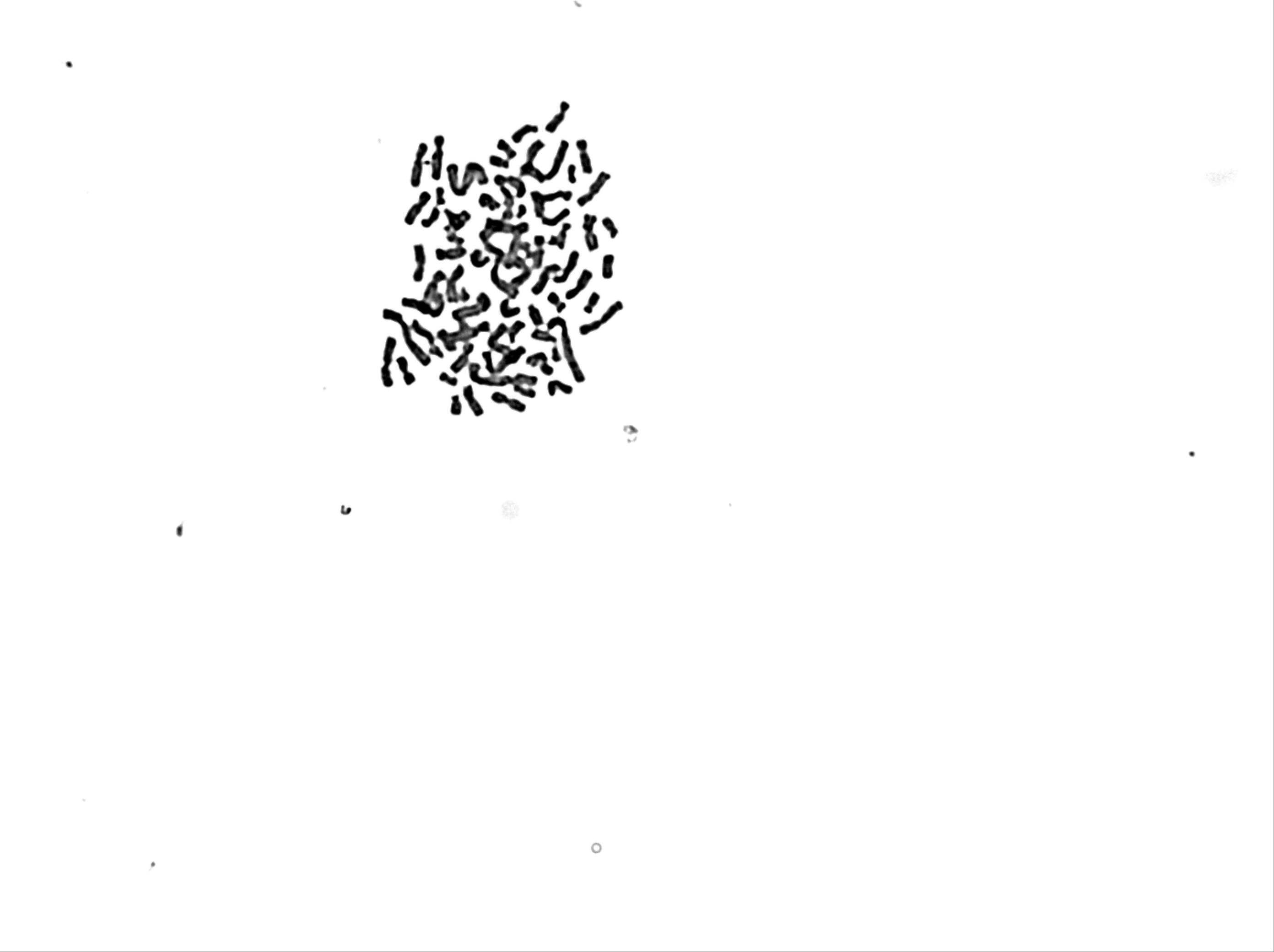

Supplement: Supplementary file 8 — Source data Fig. 7 [file 44318_2025_641_MOESM8_ESM.zip › EMBOJ-2025-120713R_SourceDataForFigure7/FIG 7A/siWAPL.tif]

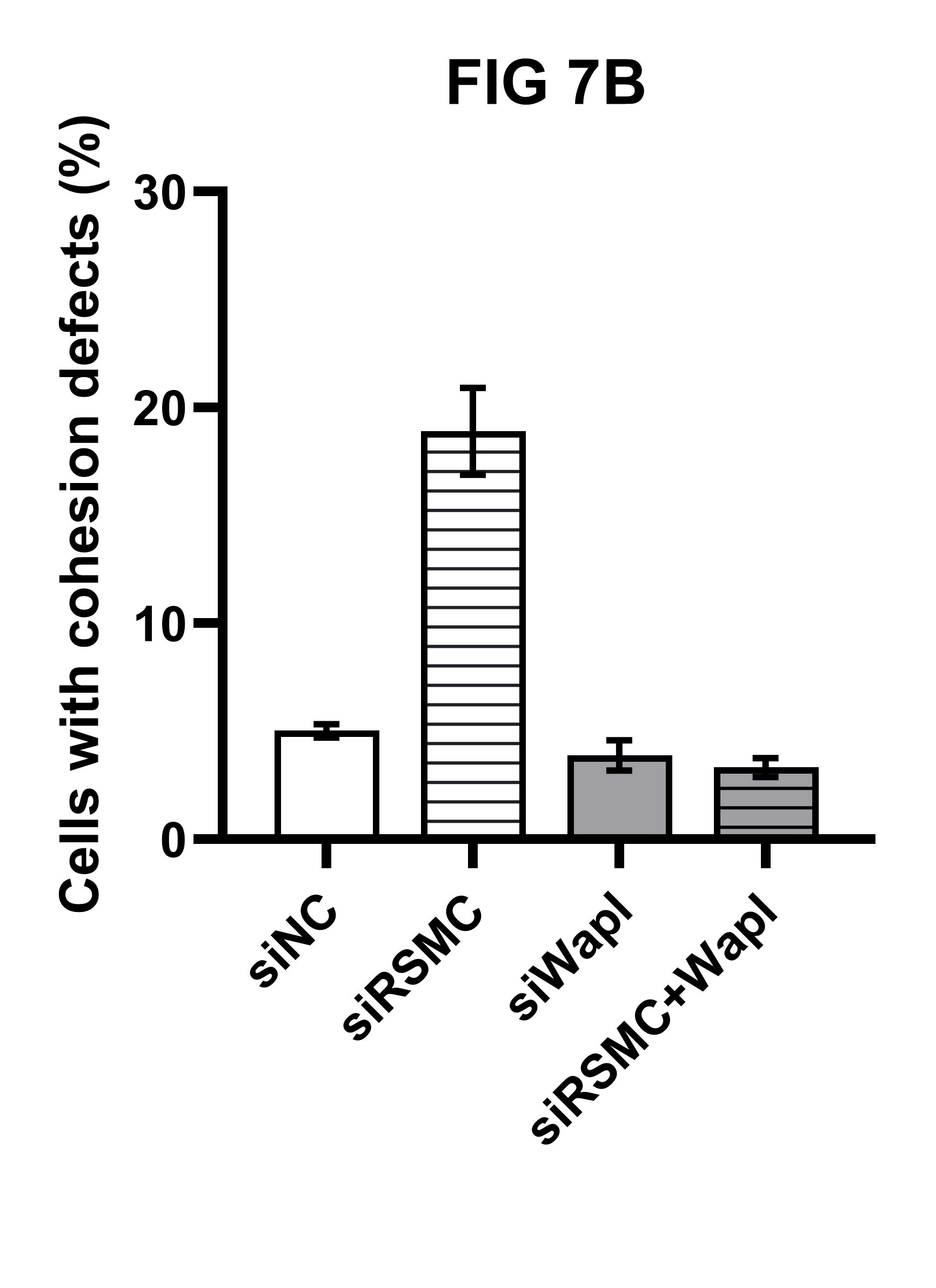

Supplement: Supplementary file 8 — Source data Fig. 7 [file 44318_2025_641_MOESM8_ESM.zip › EMBOJ-2025-120713R_SourceDataForFigure7/FIG 7B/FIG 7B before PS.tif]

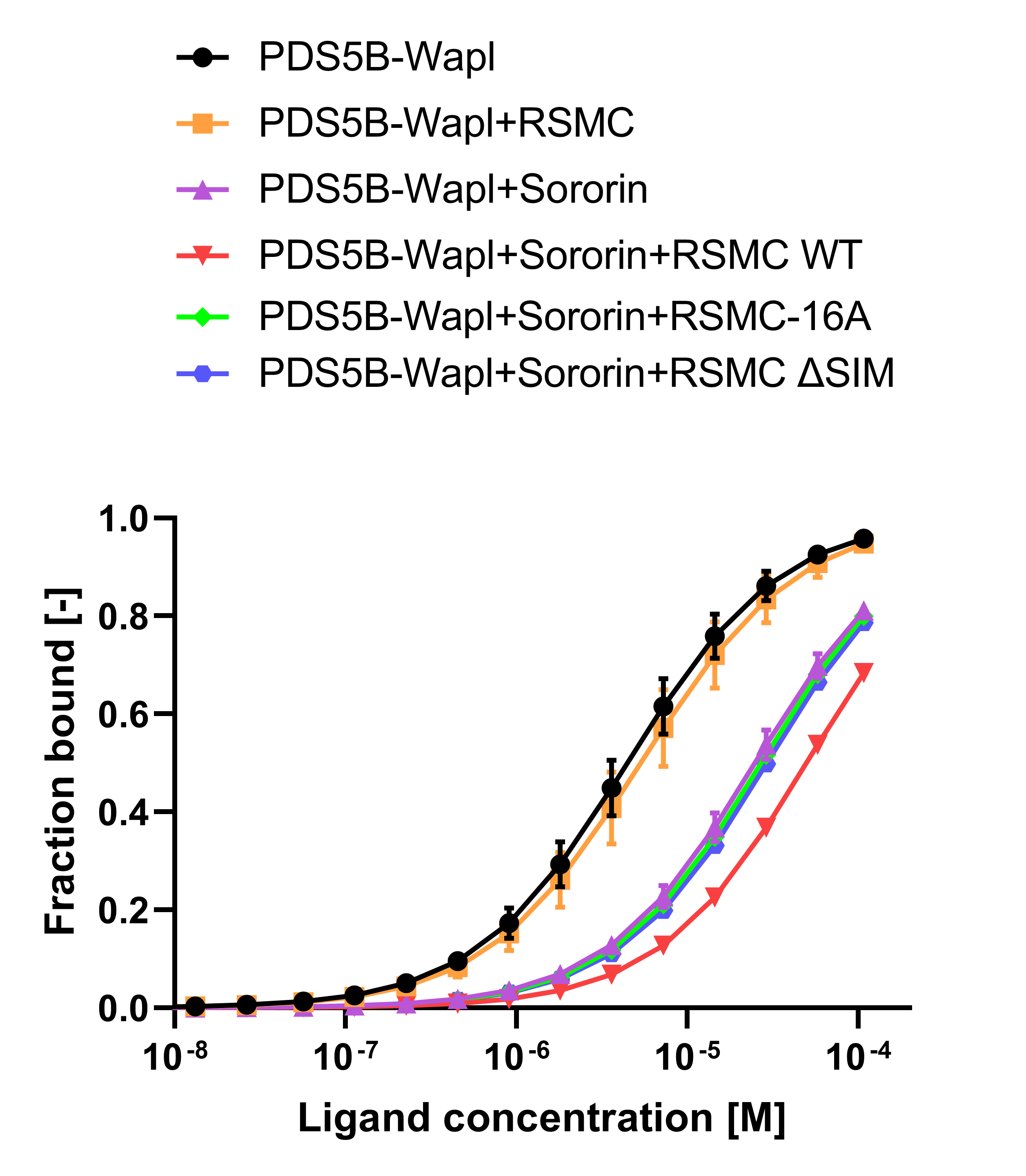

Supplement: Supplementary file 8 — Source data Fig. 7 [file 44318_2025_641_MOESM8_ESM.zip › EMBOJ-2025-120713R_SourceDataForFigure7/FIG 7C/FIG 7C before PS.tif]

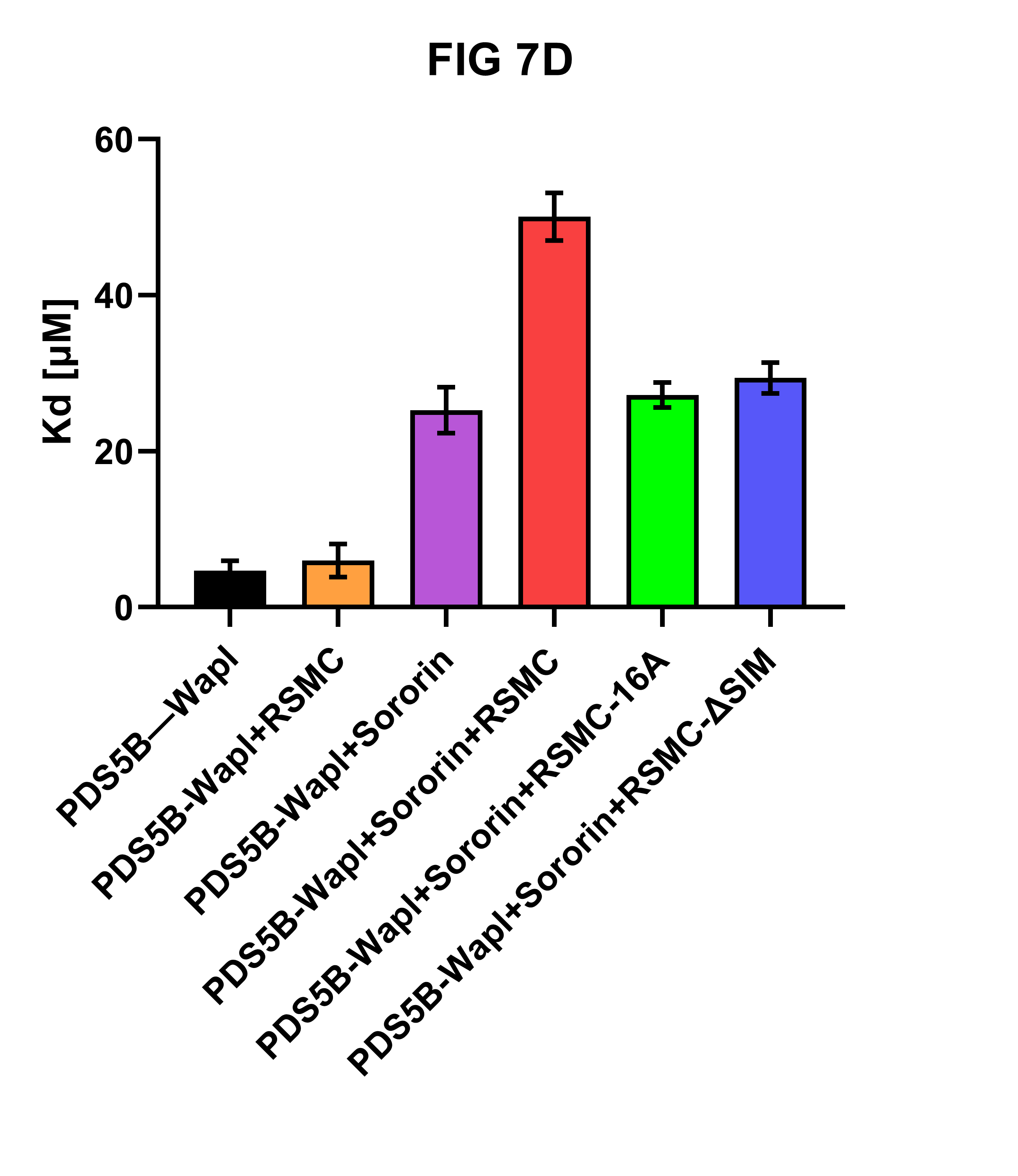

Supplement: Supplementary file 8 — Source data Fig. 7 [file 44318_2025_641_MOESM8_ESM.zip › EMBOJ-2025-120713R_SourceDataForFigure7/FIG 7D/FIG 7D before PS.tif]

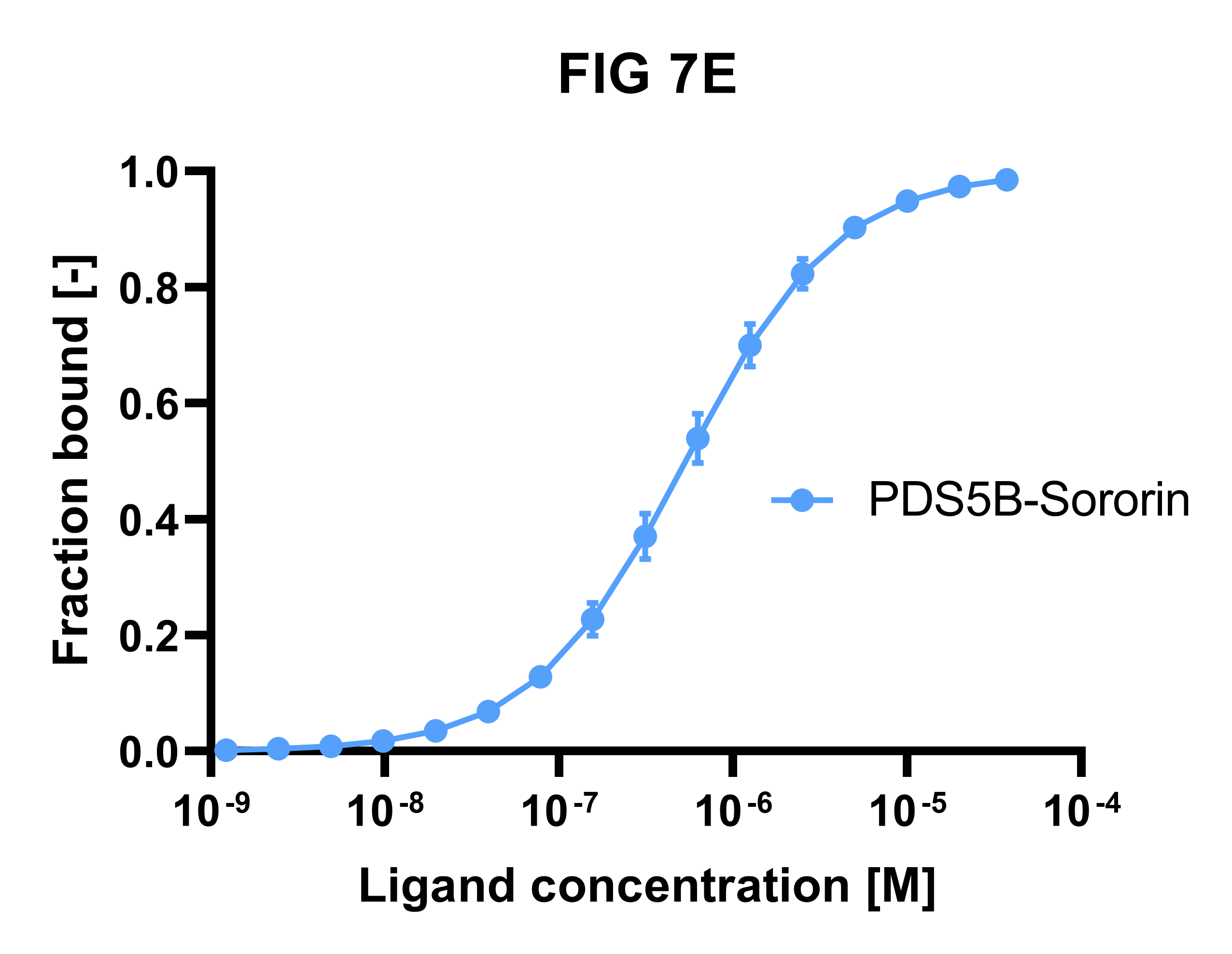

Supplement: Supplementary file 8 — Source data Fig. 7 [file 44318_2025_641_MOESM8_ESM.zip › EMBOJ-2025-120713R_SourceDataForFigure7/FIG 7E/FIG 7E before PS.tif]

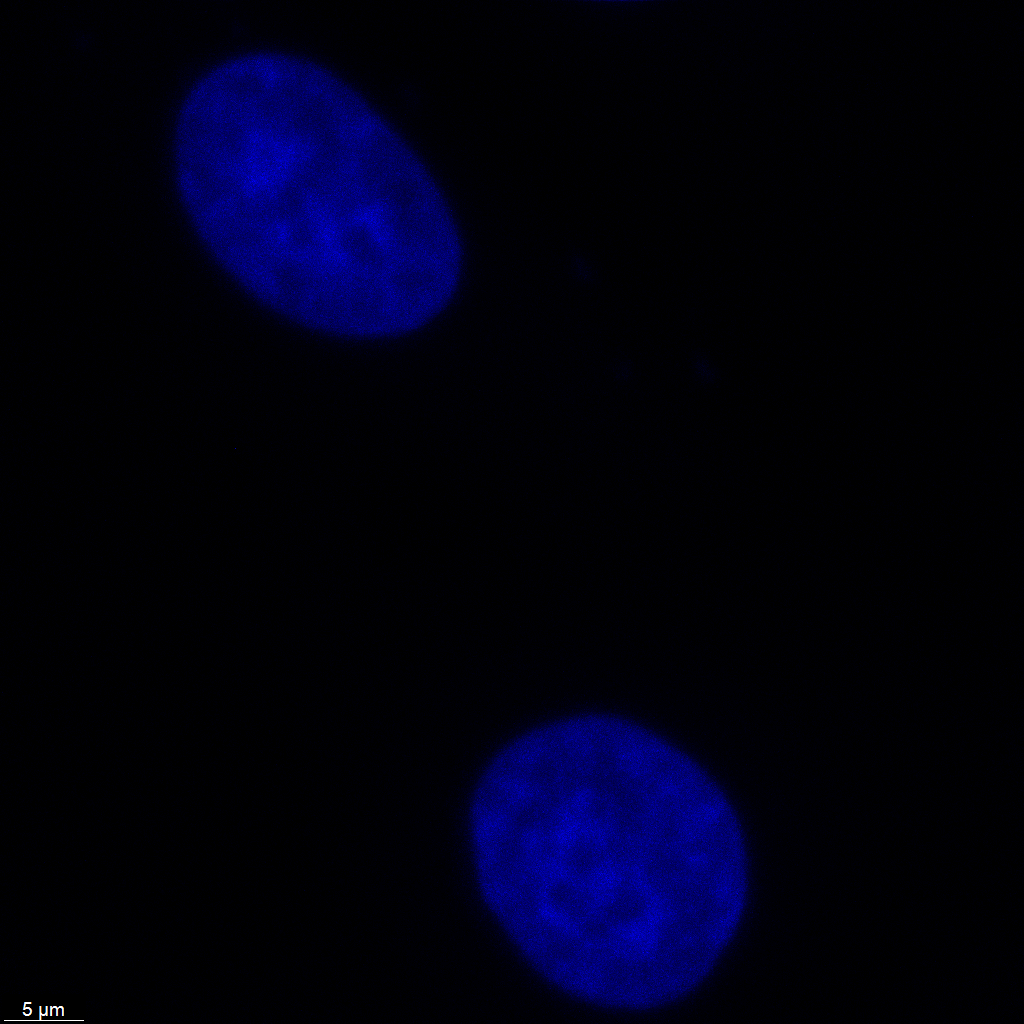

Supplement: Supplementary file 9 — Figure EV1-5 Source Data [file 44318_2025_641_MOESM9_ESM.zip › EMBOJ-2025-120713R_SourceDataForExpandedView/EMBOJ-2025-120713R_SourceDataForFigureEV1/FIG EV1D/DAPI.tif]

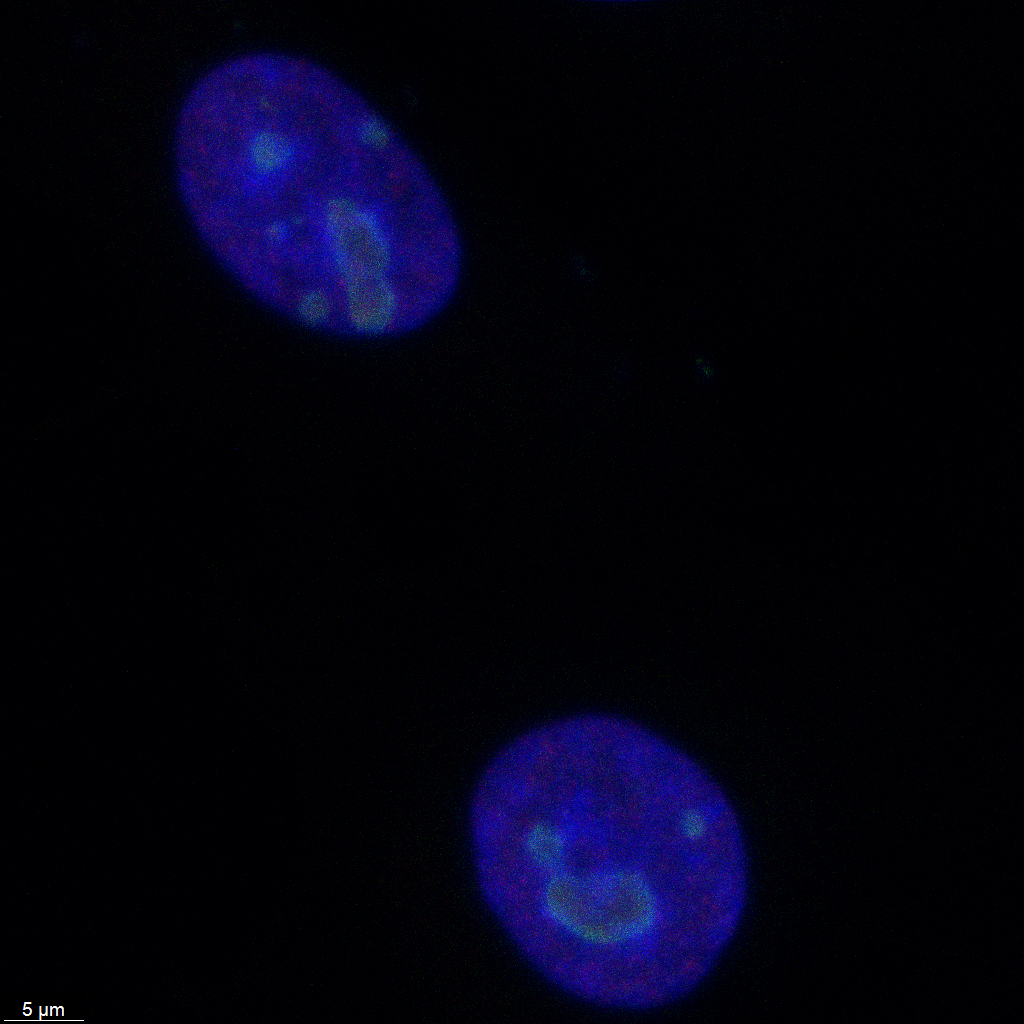

Supplement: Supplementary file 9 — Figure EV1-5 Source Data [file 44318_2025_641_MOESM9_ESM.zip › EMBOJ-2025-120713R_SourceDataForExpandedView/EMBOJ-2025-120713R_SourceDataForFigureEV1/FIG EV1D/Merge.tif]

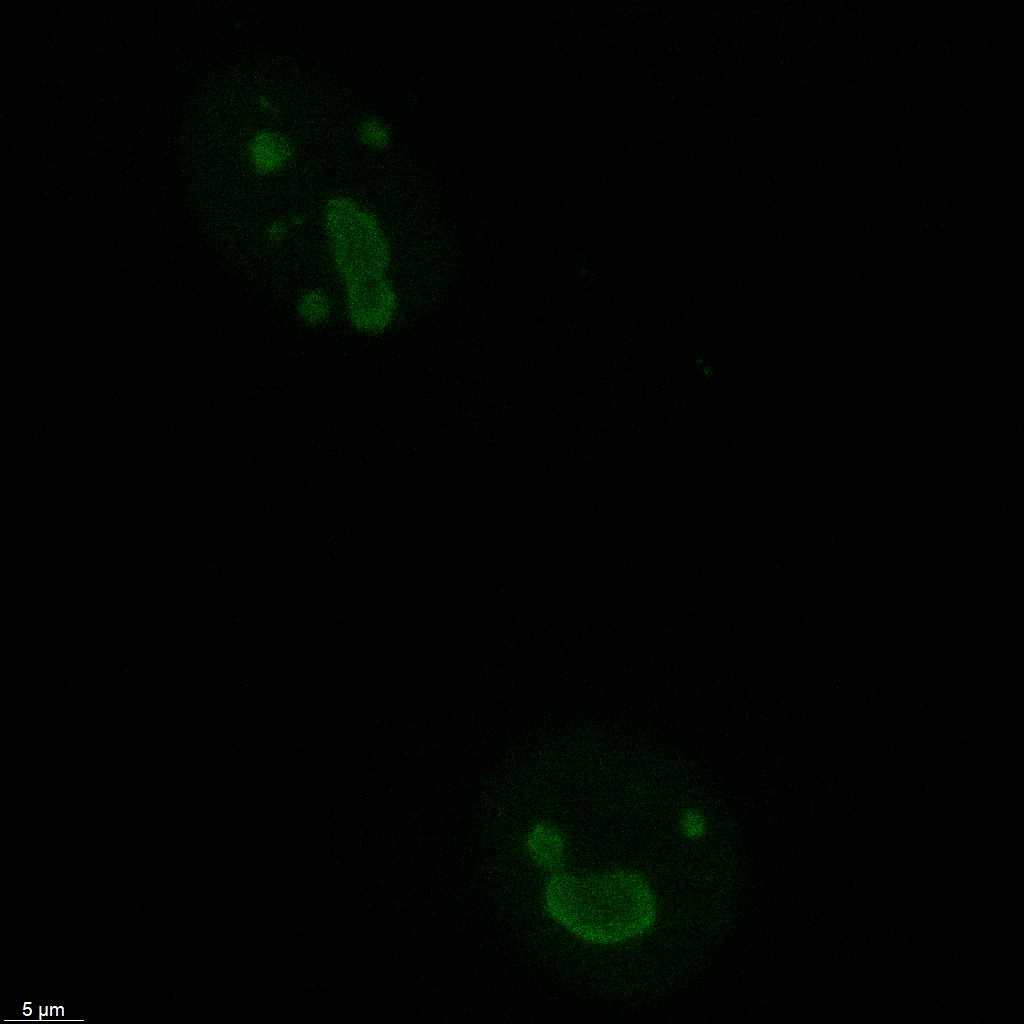

Supplement: Supplementary file 9 — Figure EV1-5 Source Data [file 44318_2025_641_MOESM9_ESM.zip › EMBOJ-2025-120713R_SourceDataForExpandedView/EMBOJ-2025-120713R_SourceDataForFigureEV1/FIG EV1D/RSMC-3FLAG.tif]

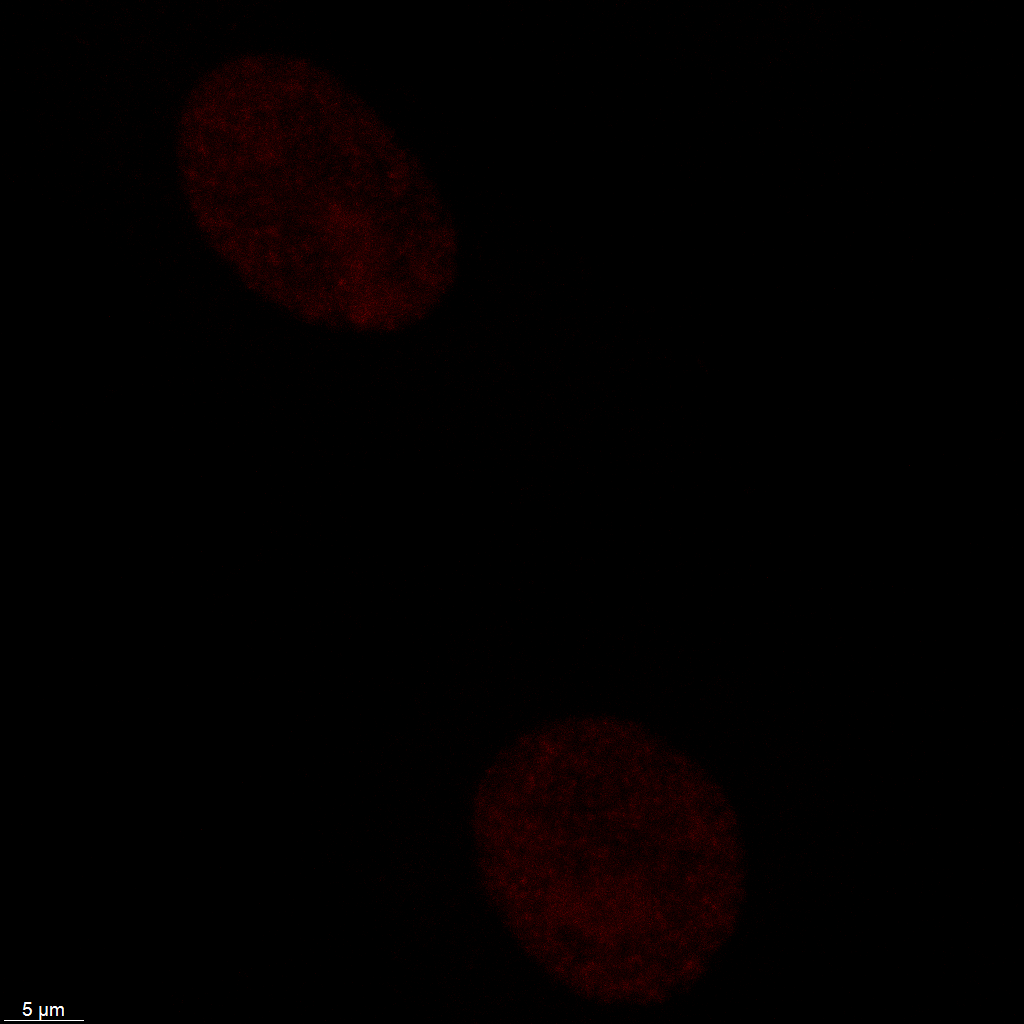

Supplement: Supplementary file 9 — Figure EV1-5 Source Data [file 44318_2025_641_MOESM9_ESM.zip › EMBOJ-2025-120713R_SourceDataForExpandedView/EMBOJ-2025-120713R_SourceDataForFigureEV1/FIG EV1D/Sororin.tif]

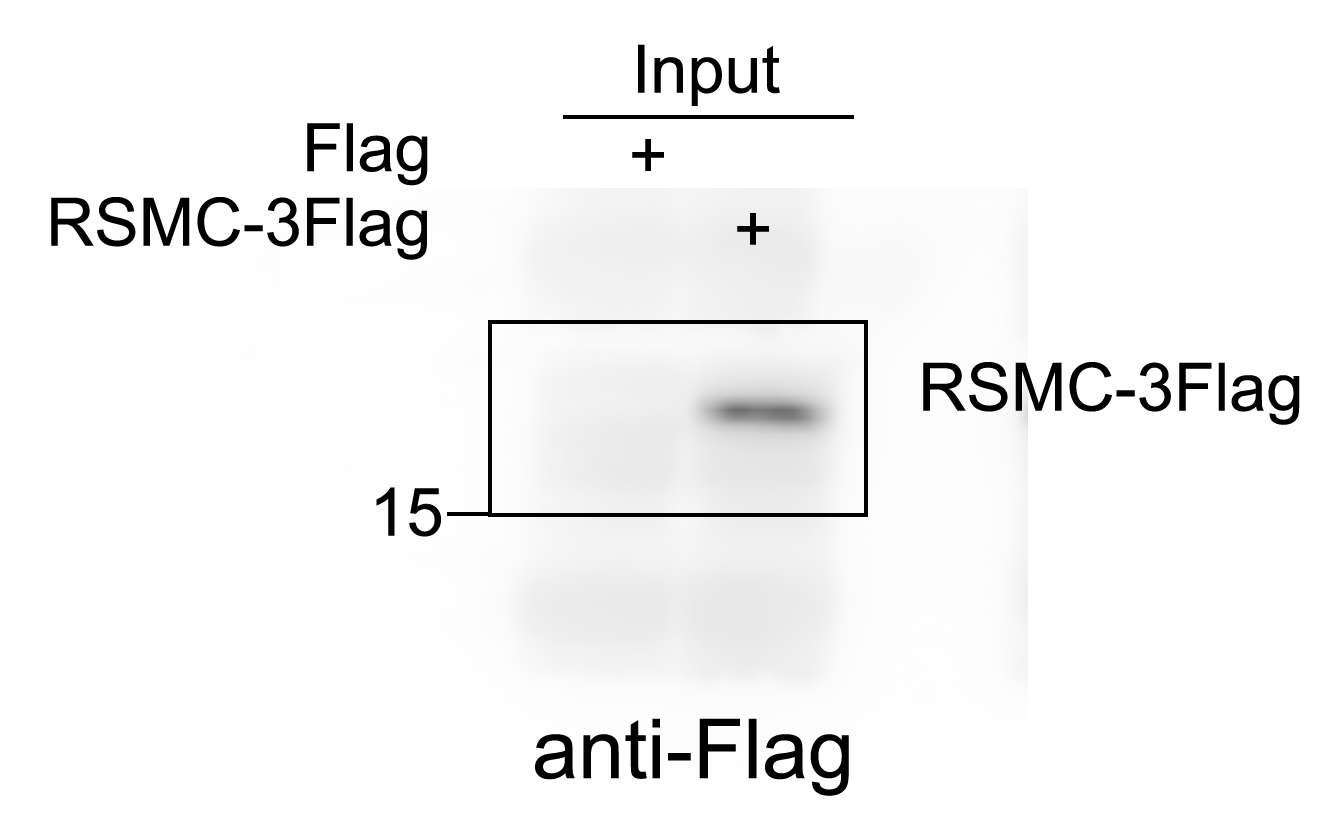

Supplement: Supplementary file 9 — Figure EV1-5 Source Data [file 44318_2025_641_MOESM9_ESM.zip › EMBOJ-2025-120713R_SourceDataForExpandedView/EMBOJ-2025-120713R_SourceDataForFigureEV1/FIG EV1E/anti-Flag input RawData.tif]

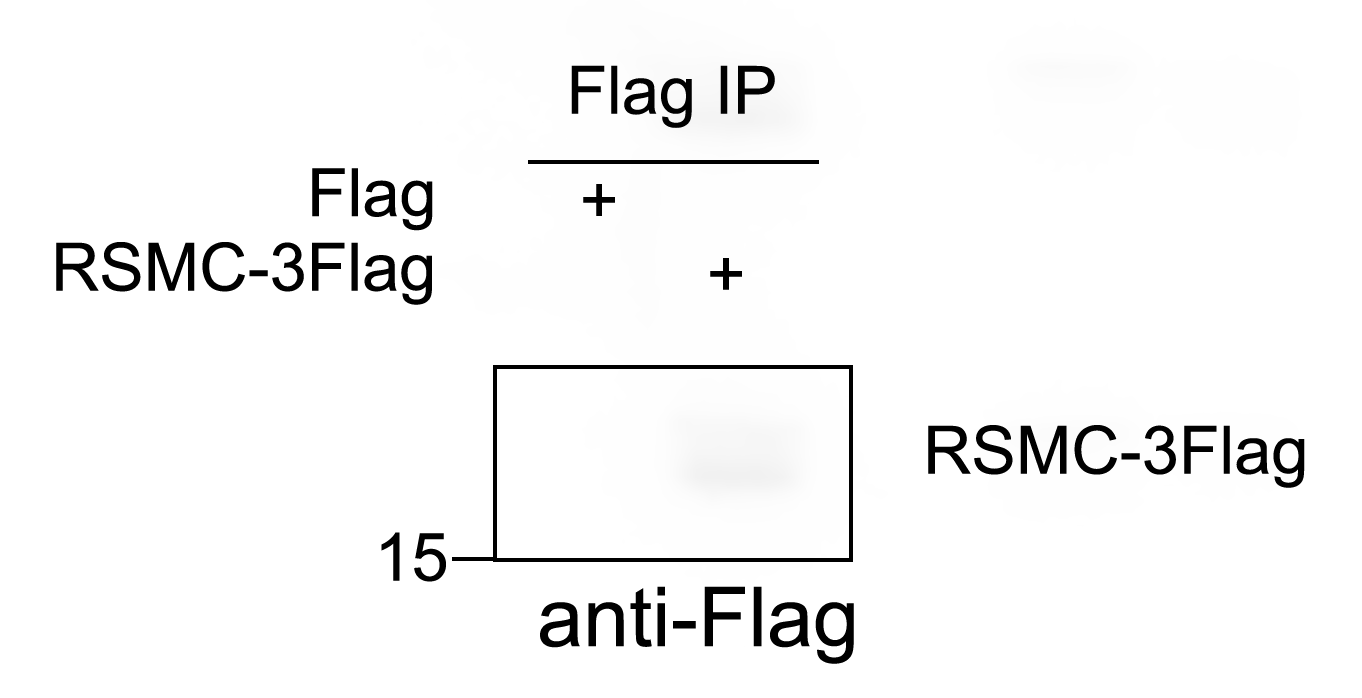

Supplement: Supplementary file 9 — Figure EV1-5 Source Data [file 44318_2025_641_MOESM9_ESM.zip › EMBOJ-2025-120713R_SourceDataForExpandedView/EMBOJ-2025-120713R_SourceDataForFigureEV1/FIG EV1E/anti-Flag IP RawData.tif]

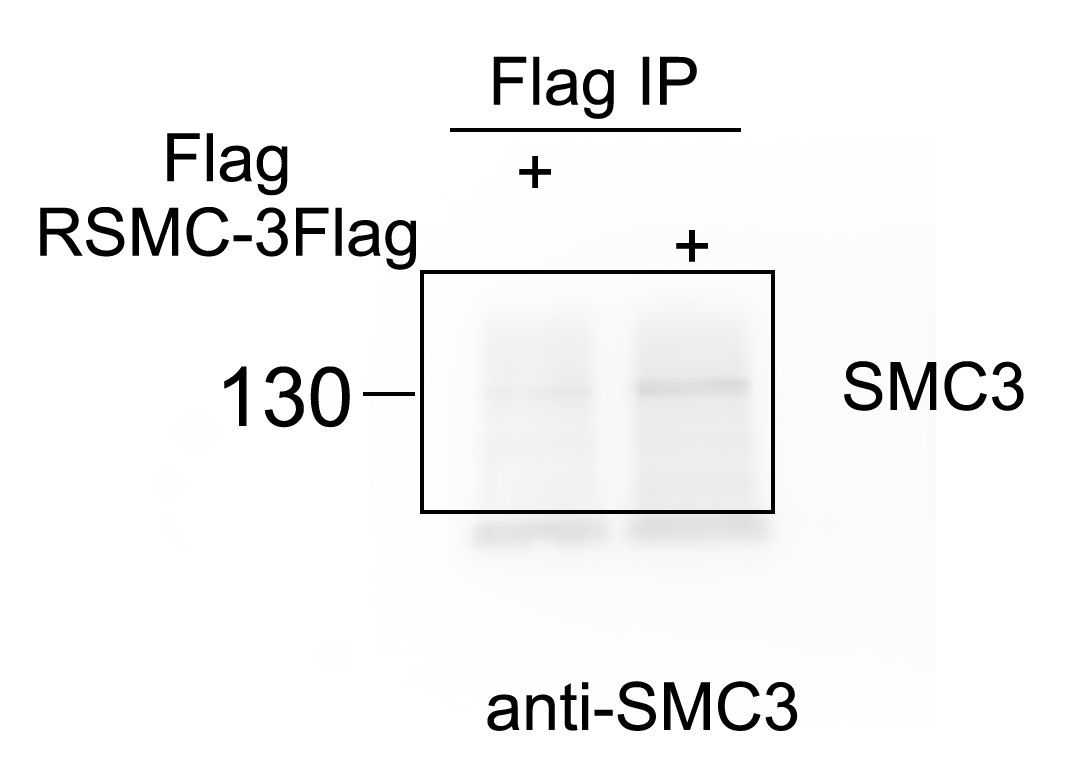

Supplement: Supplementary file 9 — Figure EV1-5 Source Data [file 44318_2025_641_MOESM9_ESM.zip › EMBOJ-2025-120713R_SourceDataForExpandedView/EMBOJ-2025-120713R_SourceDataForFigureEV1/FIG EV1E/anti-SMC3 co-IP RawData.tif]

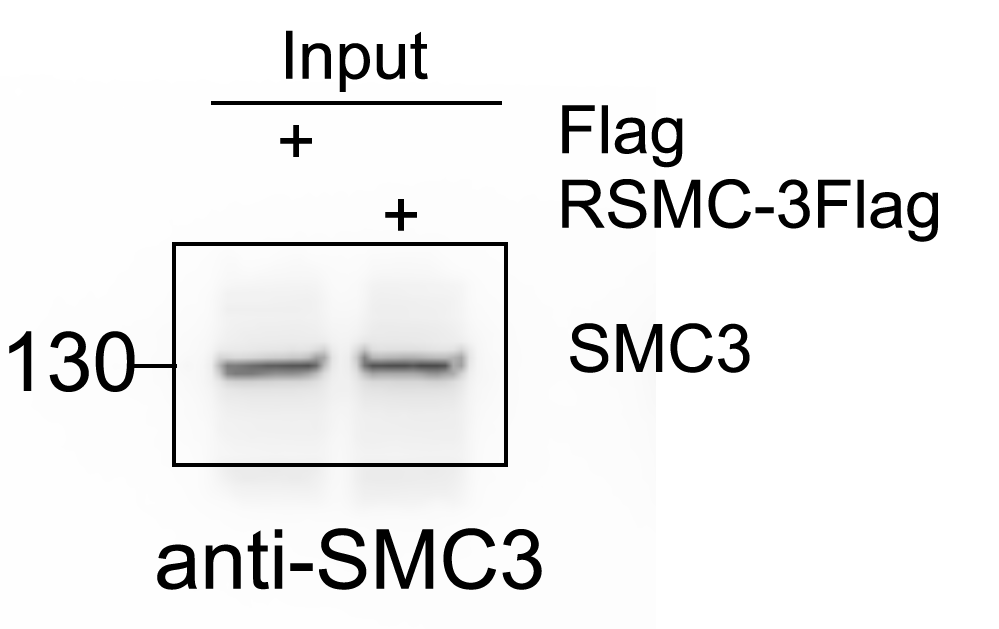

Supplement: Supplementary file 9 — Figure EV1-5 Source Data [file 44318_2025_641_MOESM9_ESM.zip › EMBOJ-2025-120713R_SourceDataForExpandedView/EMBOJ-2025-120713R_SourceDataForFigureEV1/FIG EV1E/anti-SMC3 input RawData.tif]

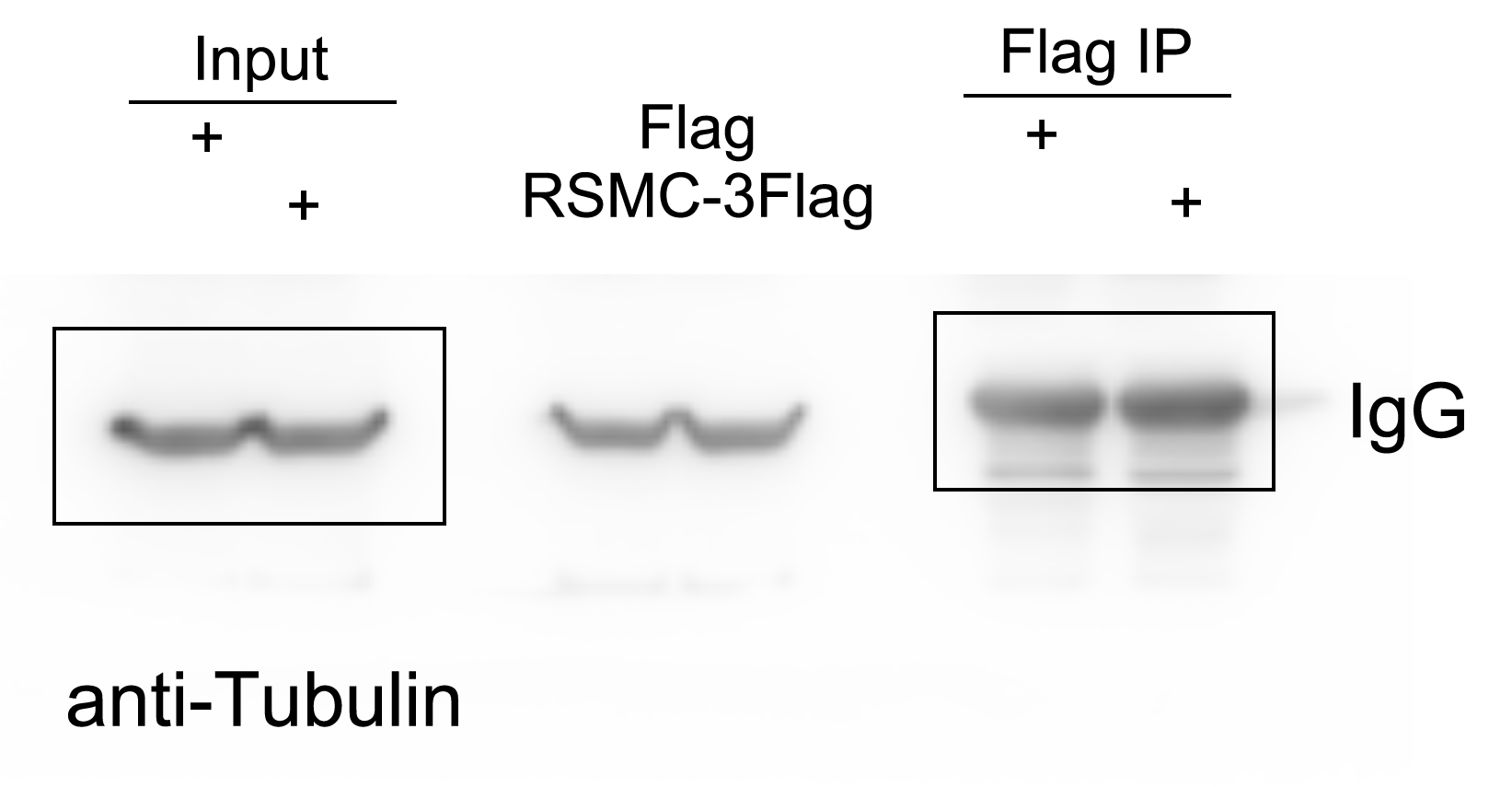

Supplement: Supplementary file 9 — Figure EV1-5 Source Data [file 44318_2025_641_MOESM9_ESM.zip › EMBOJ-2025-120713R_SourceDataForExpandedView/EMBOJ-2025-120713R_SourceDataForFigureEV1/FIG EV1E/anti-tubulin inpput RawData.tif]

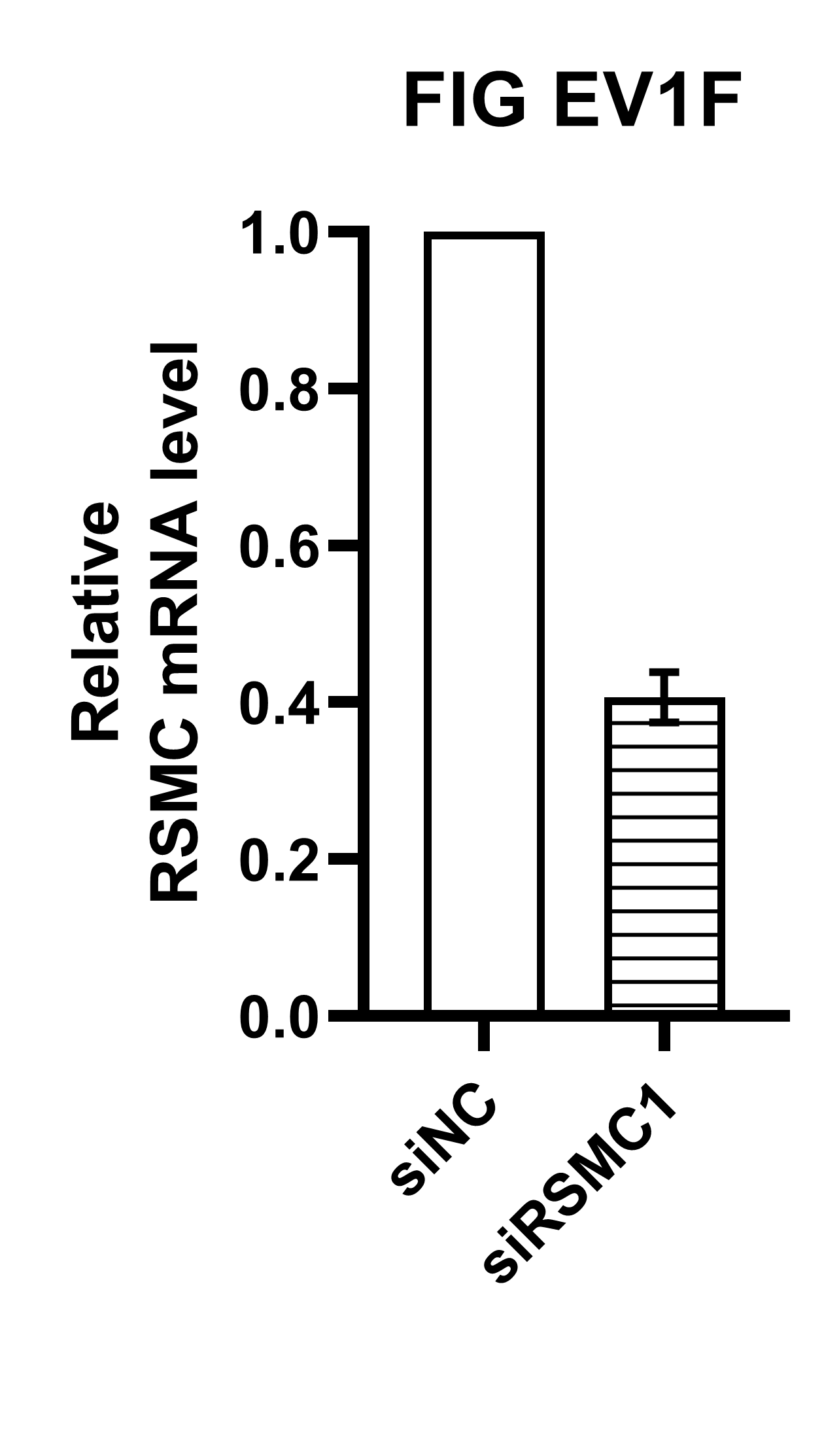

Supplement: Supplementary file 9 — Figure EV1-5 Source Data [file 44318_2025_641_MOESM9_ESM.zip › EMBOJ-2025-120713R_SourceDataForExpandedView/EMBOJ-2025-120713R_SourceDataForFigureEV1/FIG EV1F/FIG EV1F before PS.tif]

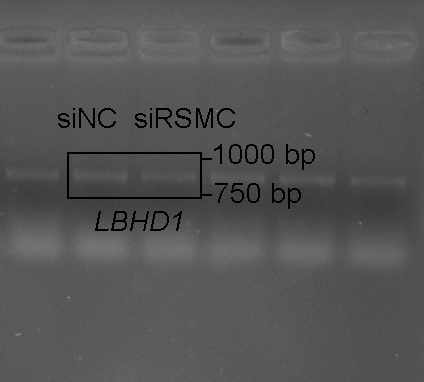

Supplement: Supplementary file 9 — Figure EV1-5 Source Data [file 44318_2025_641_MOESM9_ESM.zip › EMBOJ-2025-120713R_SourceDataForExpandedView/EMBOJ-2025-120713R_SourceDataForFigureEV1/FIG EV1F/Used for figure/LBHD1.tif]

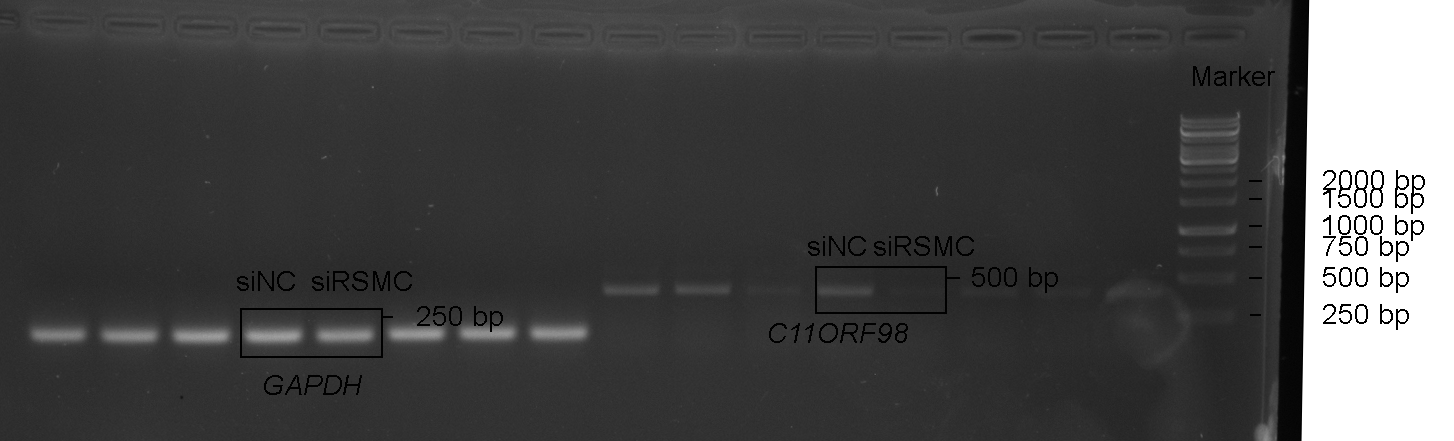

Supplement: Supplementary file 9 — Figure EV1-5 Source Data [file 44318_2025_641_MOESM9_ESM.zip › EMBOJ-2025-120713R_SourceDataForExpandedView/EMBOJ-2025-120713R_SourceDataForFigureEV1/FIG EV1F/Used for figure/RSMC GAPDH.tif]

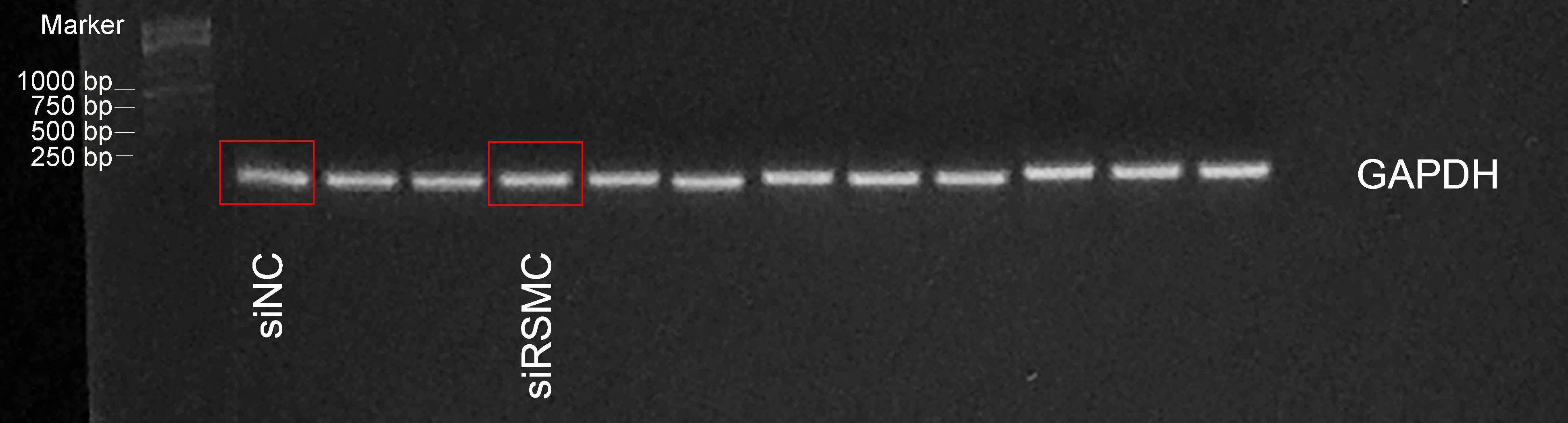

Supplement: Supplementary file 9 — Figure EV1-5 Source Data [file 44318_2025_641_MOESM9_ESM.zip › EMBOJ-2025-120713R_SourceDataForExpandedView/EMBOJ-2025-120713R_SourceDataForFigureEV1/FIG EV1F/Used for quantification/exp1/GAPDH.tif]

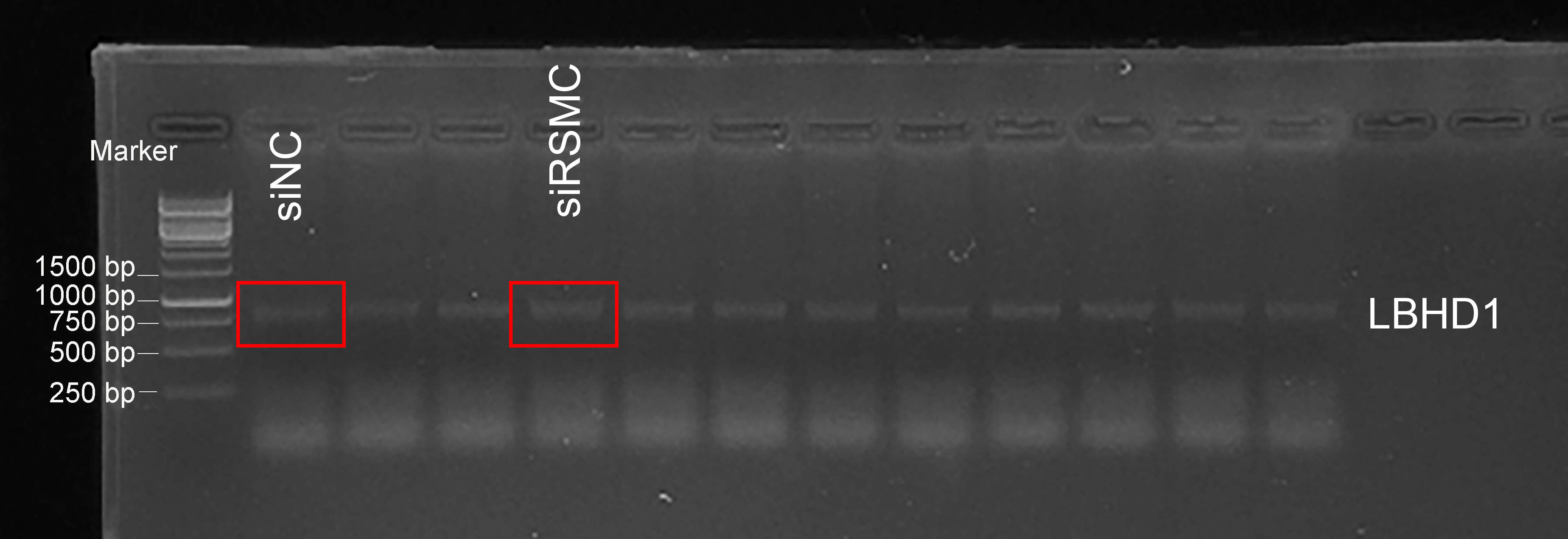

Supplement: Supplementary file 9 — Figure EV1-5 Source Data [file 44318_2025_641_MOESM9_ESM.zip › EMBOJ-2025-120713R_SourceDataForExpandedView/EMBOJ-2025-120713R_SourceDataForFigureEV1/FIG EV1F/Used for quantification/exp1/LBHD1.tif]

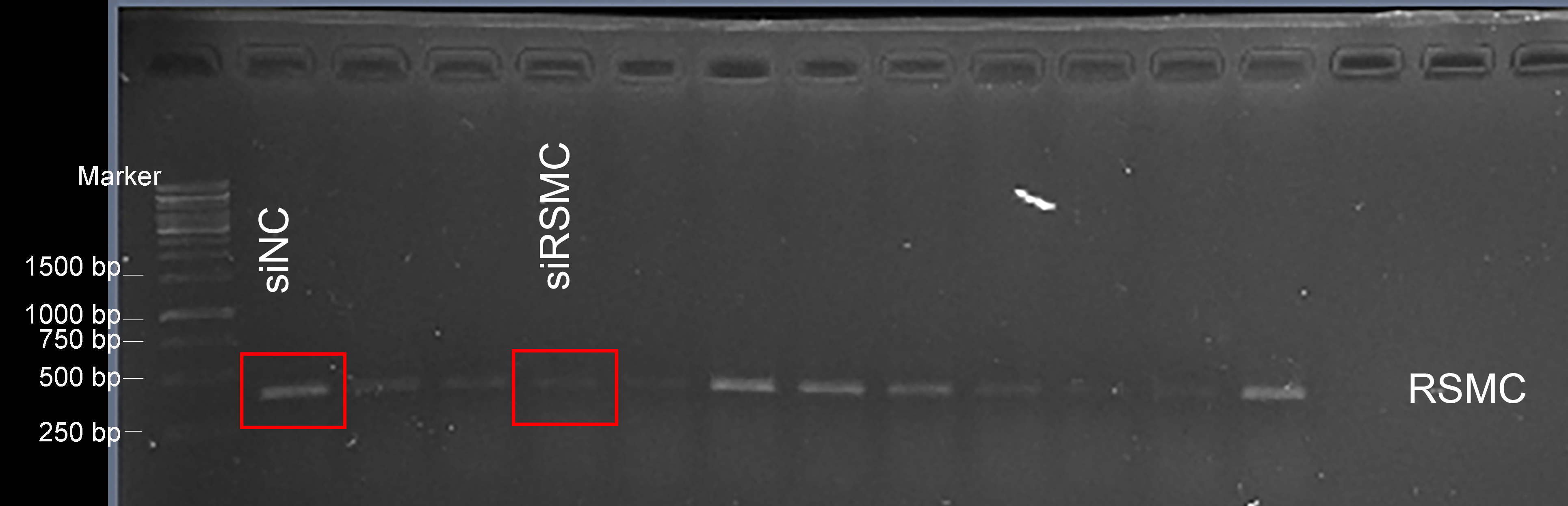

Supplement: Supplementary file 9 — Figure EV1-5 Source Data [file 44318_2025_641_MOESM9_ESM.zip › EMBOJ-2025-120713R_SourceDataForExpandedView/EMBOJ-2025-120713R_SourceDataForFigureEV1/FIG EV1F/Used for quantification/exp1/RSMC.tif]

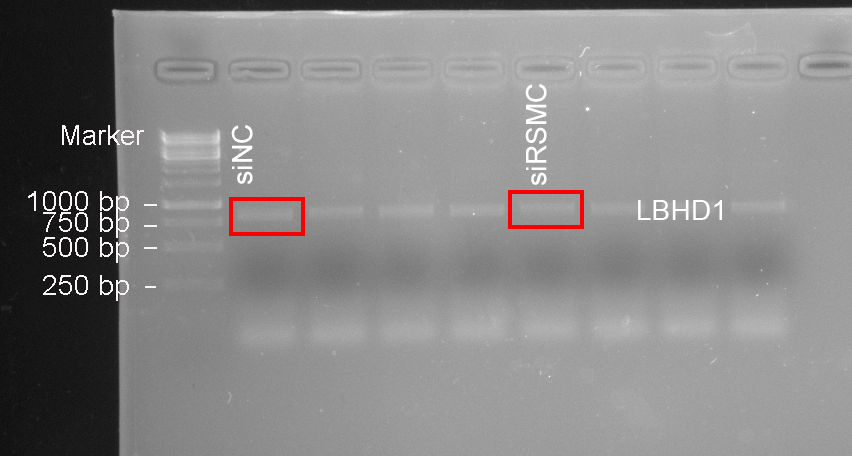

Supplement: Supplementary file 9 — Figure EV1-5 Source Data [file 44318_2025_641_MOESM9_ESM.zip › EMBOJ-2025-120713R_SourceDataForExpandedView/EMBOJ-2025-120713R_SourceDataForFigureEV1/FIG EV1F/Used for quantification/exp2/LBHD1.tif]

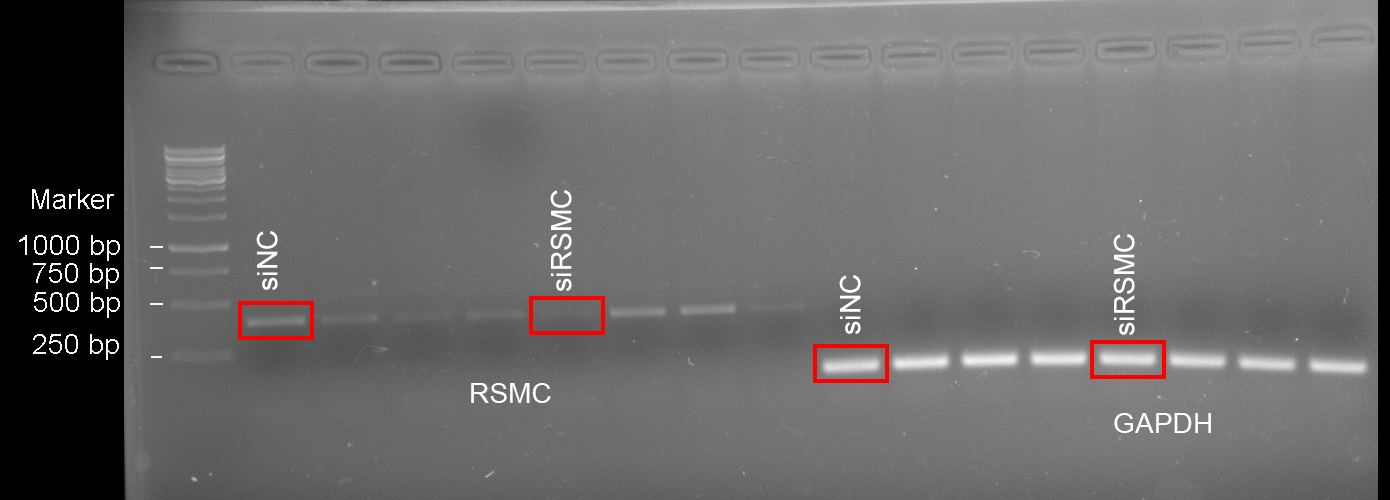

Supplement: Supplementary file 9 — Figure EV1-5 Source Data [file 44318_2025_641_MOESM9_ESM.zip › EMBOJ-2025-120713R_SourceDataForExpandedView/EMBOJ-2025-120713R_SourceDataForFigureEV1/FIG EV1F/Used for quantification/exp2/RSMC-GAPDH.tif]

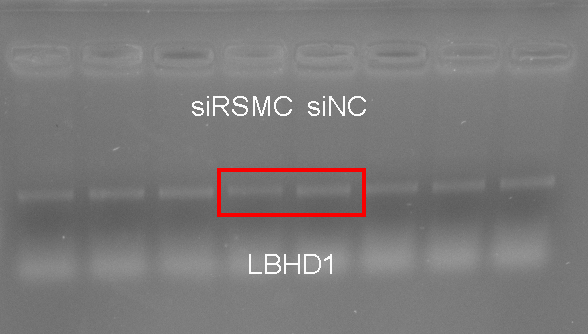

Supplement: Supplementary file 9 — Figure EV1-5 Source Data [file 44318_2025_641_MOESM9_ESM.zip › EMBOJ-2025-120713R_SourceDataForExpandedView/EMBOJ-2025-120713R_SourceDataForFigureEV1/FIG EV1F/Used for quantification/exp3/LBHD1.tif]

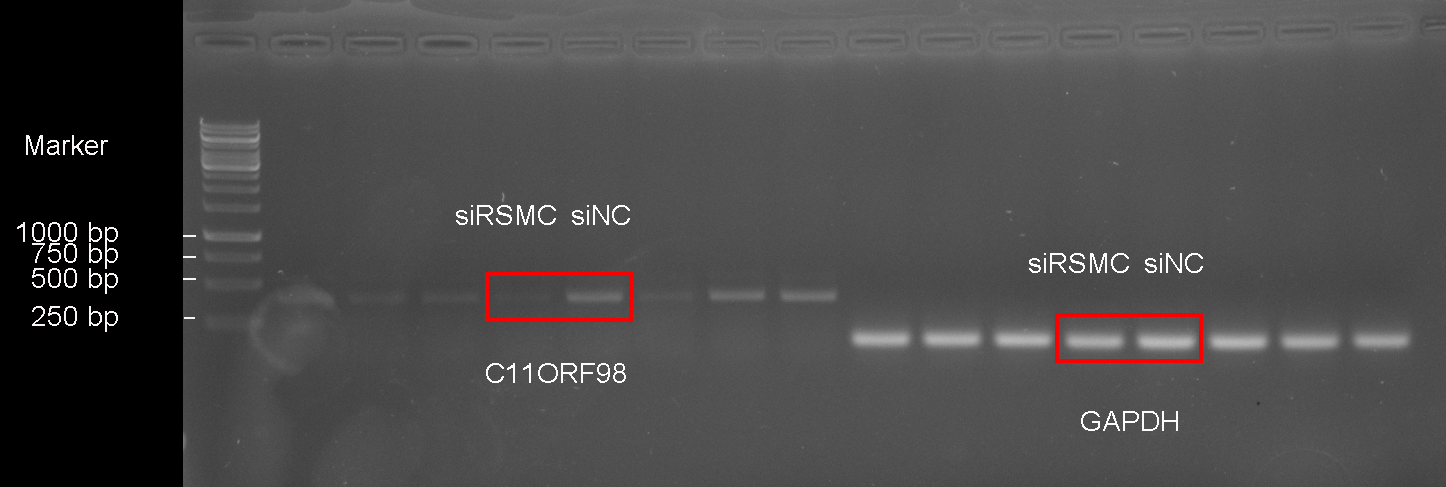

Supplement: Supplementary file 9 — Figure EV1-5 Source Data [file 44318_2025_641_MOESM9_ESM.zip › EMBOJ-2025-120713R_SourceDataForExpandedView/EMBOJ-2025-120713R_SourceDataForFigureEV1/FIG EV1F/Used for quantification/exp3/RSMC GAPDH.tif]

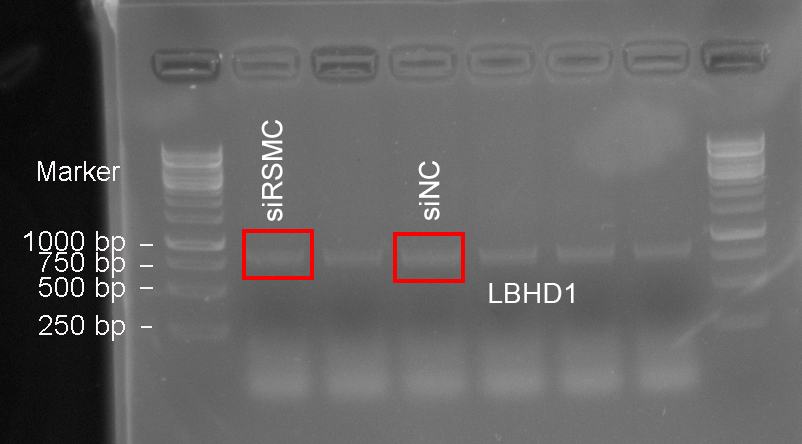

Supplement: Supplementary file 9 — Figure EV1-5 Source Data [file 44318_2025_641_MOESM9_ESM.zip › EMBOJ-2025-120713R_SourceDataForExpandedView/EMBOJ-2025-120713R_SourceDataForFigureEV1/FIG EV1F/Used for quantification/exp4/LBHD1.tif]

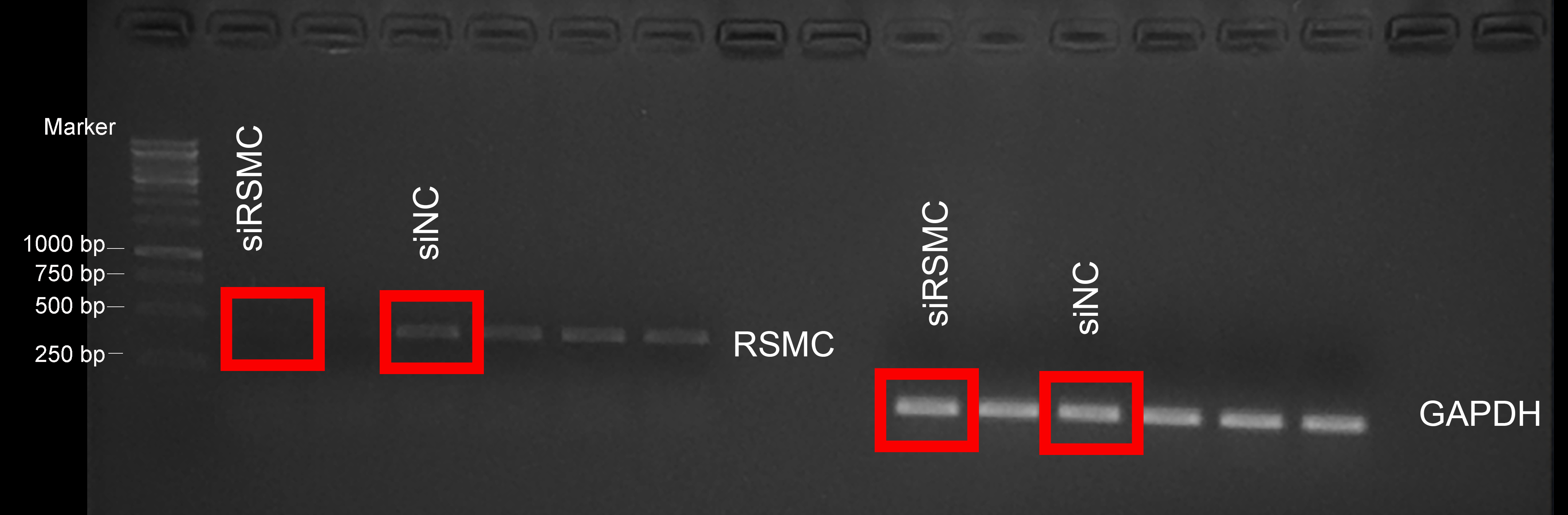

Supplement: Supplementary file 9 — Figure EV1-5 Source Data [file 44318_2025_641_MOESM9_ESM.zip › EMBOJ-2025-120713R_SourceDataForExpandedView/EMBOJ-2025-120713R_SourceDataForFigureEV1/FIG EV1F/Used for quantification/exp4/RSMC GAPDH.tif]
