# Supplementary figures and images for: S-phase PARylation of microprotein RSMC enhances the function of Sororin in sister chromatid cohesion (part 3 of 3)
Source: EMBO J. 2025 Nov 19;45(1):278–309. doi: 10.1038/s44318-025-00641-8 (PMC12759081; doi:10.1038/s44318-025-00641-8)

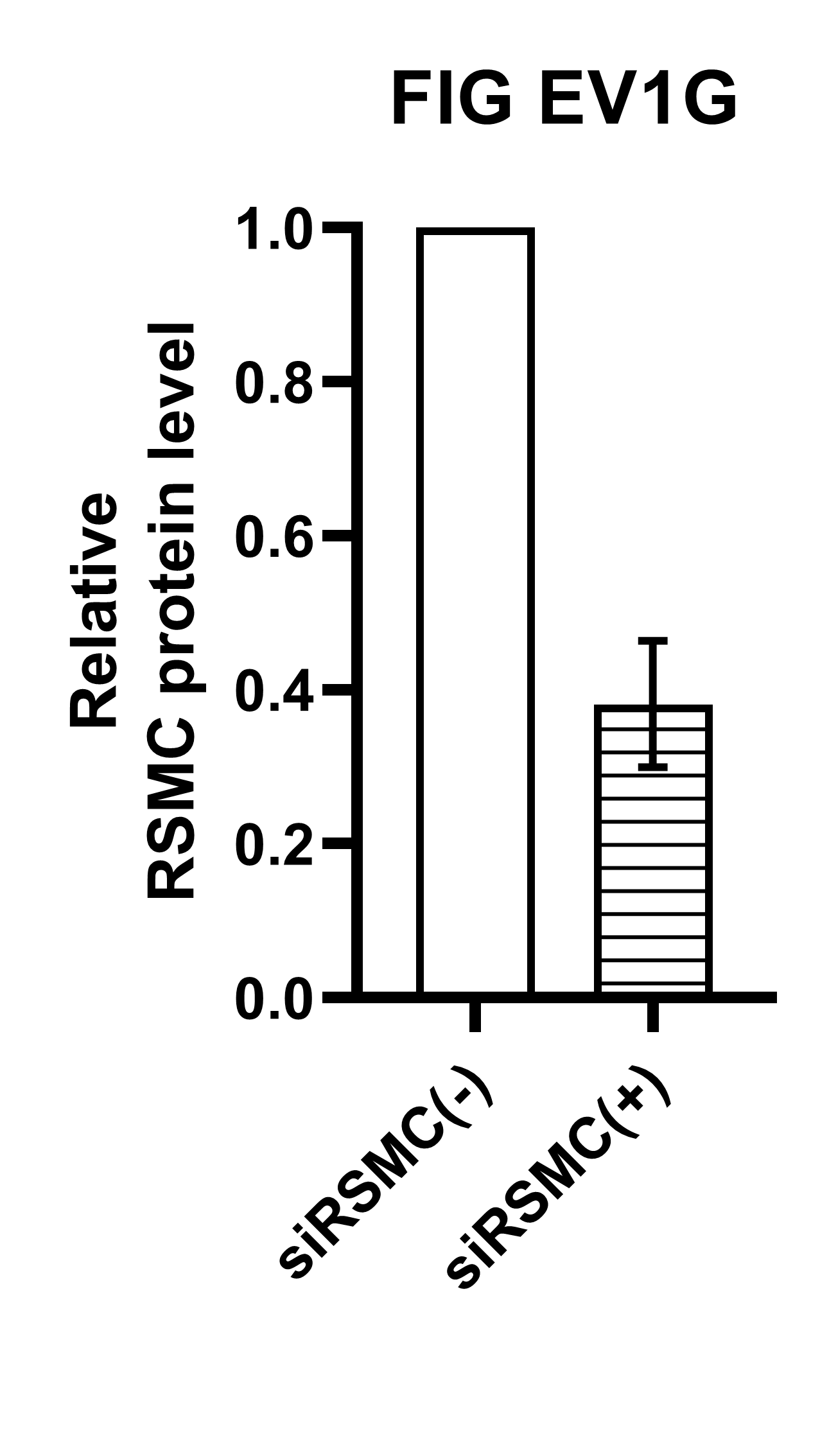

Supplement: Supplementary file 9 — Figure EV1-5 Source Data [file 44318_2025_641_MOESM9_ESM.zip › EMBOJ-2025-120713R_SourceDataForExpandedView/EMBOJ-2025-120713R_SourceDataForFigureEV1/FIG EV1G/FIG EV1G before PS.tif]

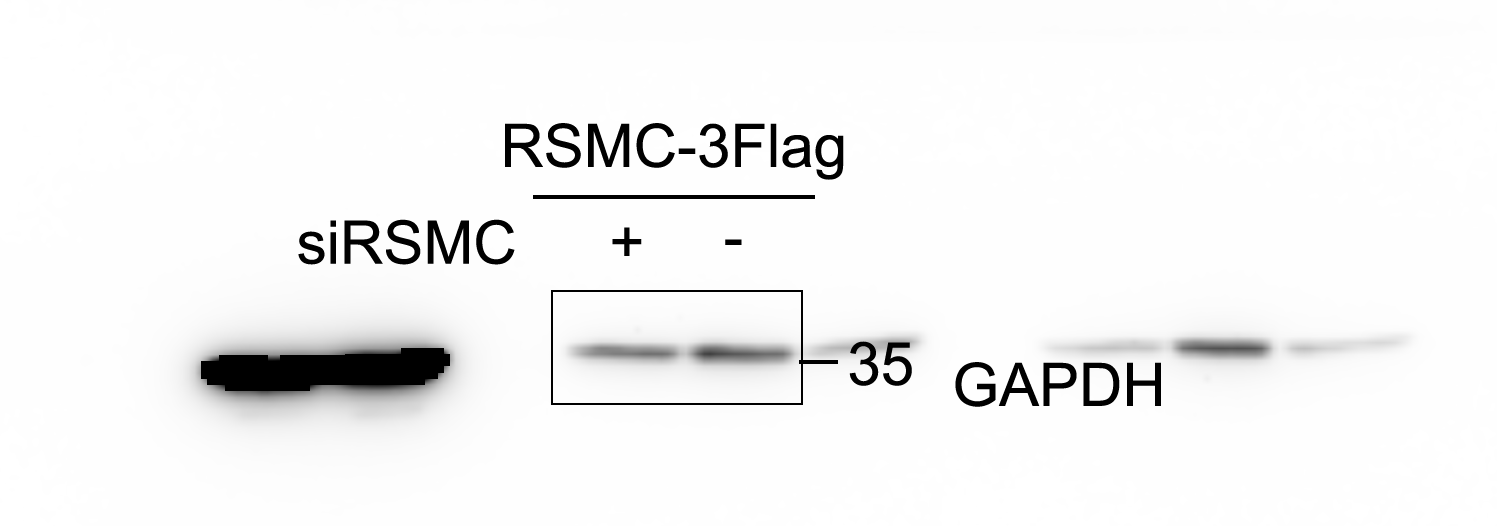

Supplement: Supplementary file 9 — Figure EV1-5 Source Data [file 44318_2025_641_MOESM9_ESM.zip › EMBOJ-2025-120713R_SourceDataForExpandedView/EMBOJ-2025-120713R_SourceDataForFigureEV1/FIG EV1G/Used for figure/FIG EV1G SourceData GAPDH.tif]

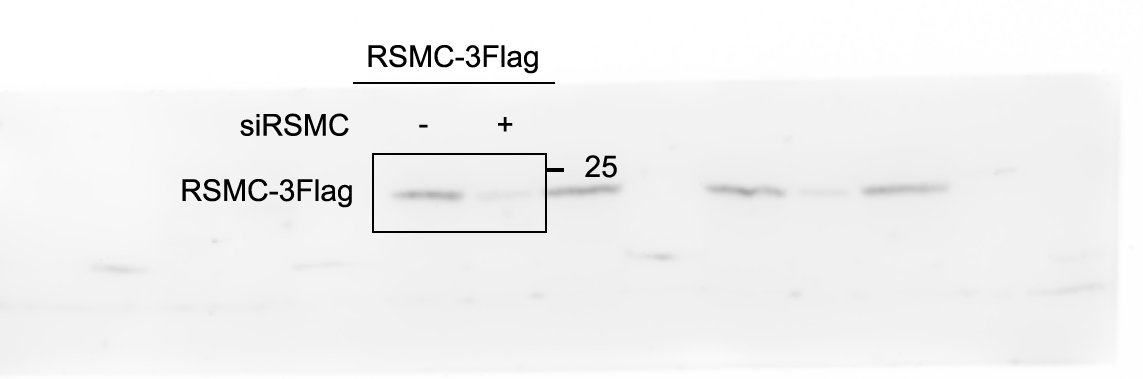

Supplement: Supplementary file 9 — Figure EV1-5 Source Data [file 44318_2025_641_MOESM9_ESM.zip › EMBOJ-2025-120713R_SourceDataForExpandedView/EMBOJ-2025-120713R_SourceDataForFigureEV1/FIG EV1G/Used for figure/FIG EV1G SourceData RSMC-3Flag.tif]

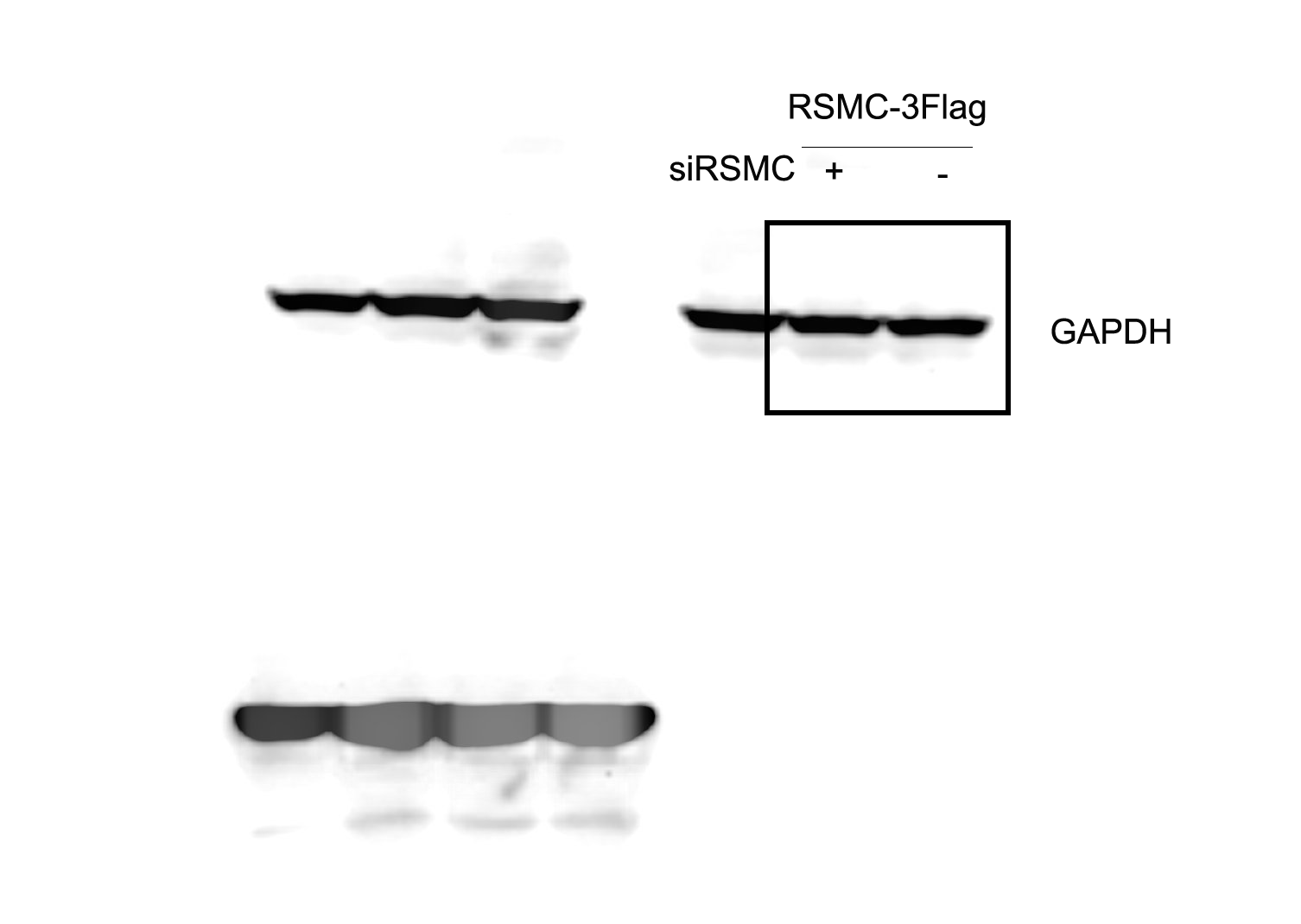

Supplement: Supplementary file 9 — Figure EV1-5 Source Data [file 44318_2025_641_MOESM9_ESM.zip › EMBOJ-2025-120713R_SourceDataForExpandedView/EMBOJ-2025-120713R_SourceDataForFigureEV1/FIG EV1G/Used for quantification/exp1/GAPDH.tif]

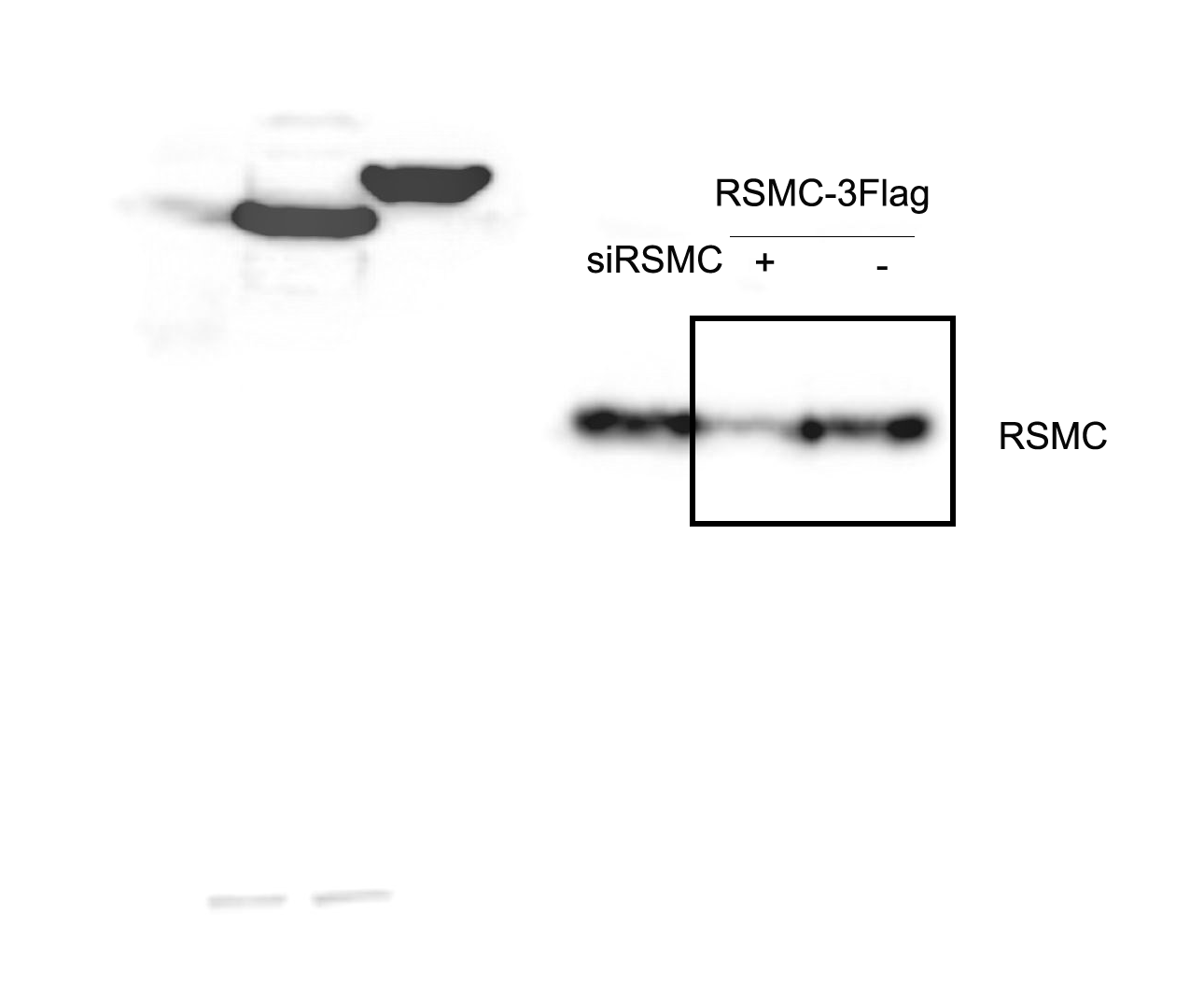

Supplement: Supplementary file 9 — Figure EV1-5 Source Data [file 44318_2025_641_MOESM9_ESM.zip › EMBOJ-2025-120713R_SourceDataForExpandedView/EMBOJ-2025-120713R_SourceDataForFigureEV1/FIG EV1G/Used for quantification/exp1/RSMC.tif]

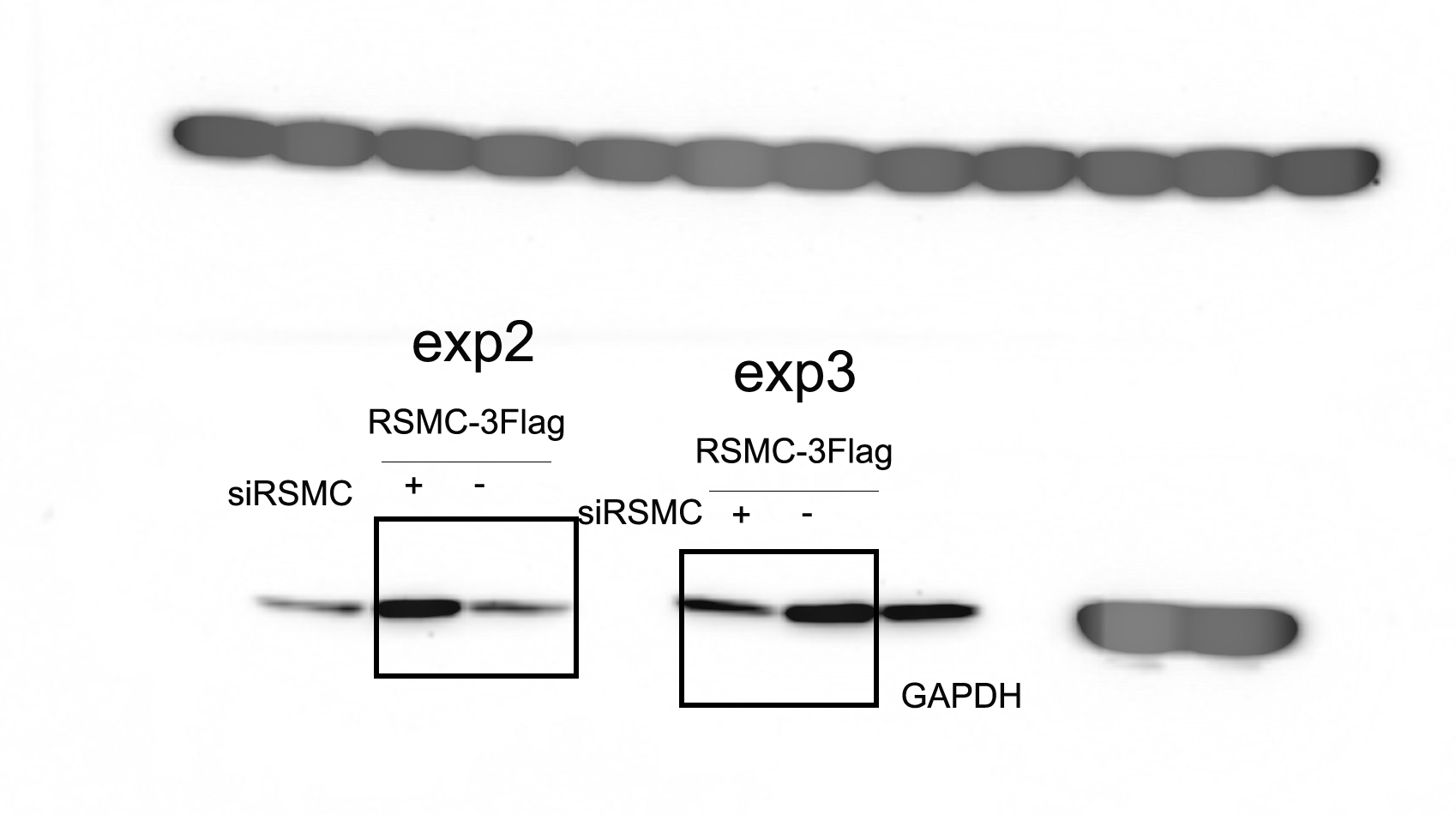

Supplement: Supplementary file 9 — Figure EV1-5 Source Data [file 44318_2025_641_MOESM9_ESM.zip › EMBOJ-2025-120713R_SourceDataForExpandedView/EMBOJ-2025-120713R_SourceDataForFigureEV1/FIG EV1G/Used for quantification/exp2-3/GAPDH.tif]

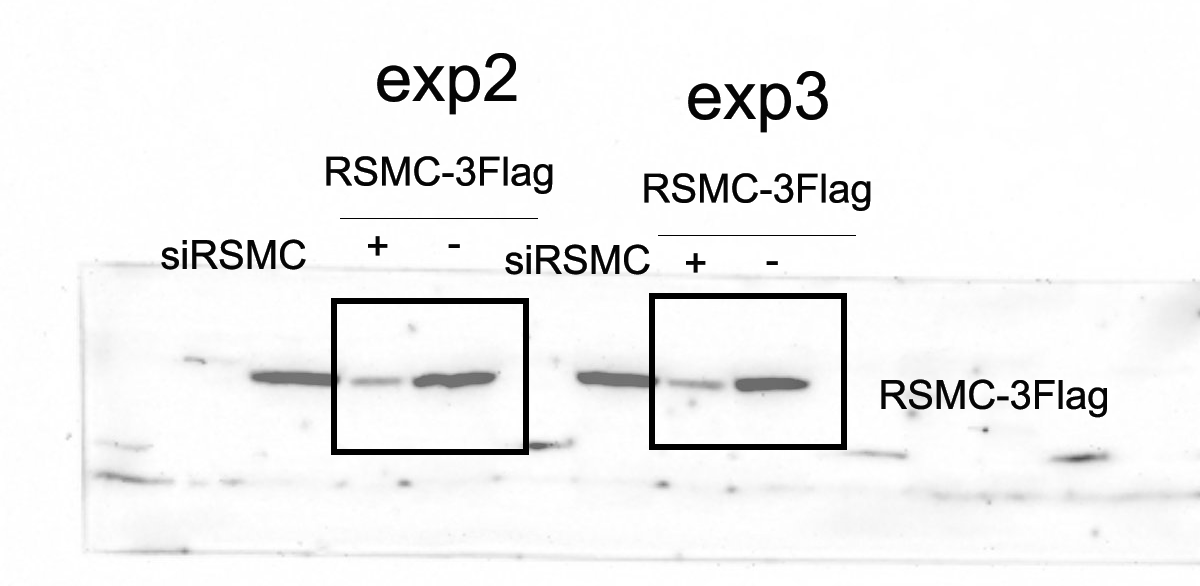

Supplement: Supplementary file 9 — Figure EV1-5 Source Data [file 44318_2025_641_MOESM9_ESM.zip › EMBOJ-2025-120713R_SourceDataForExpandedView/EMBOJ-2025-120713R_SourceDataForFigureEV1/FIG EV1G/Used for quantification/exp2-3/RSMC.tif]

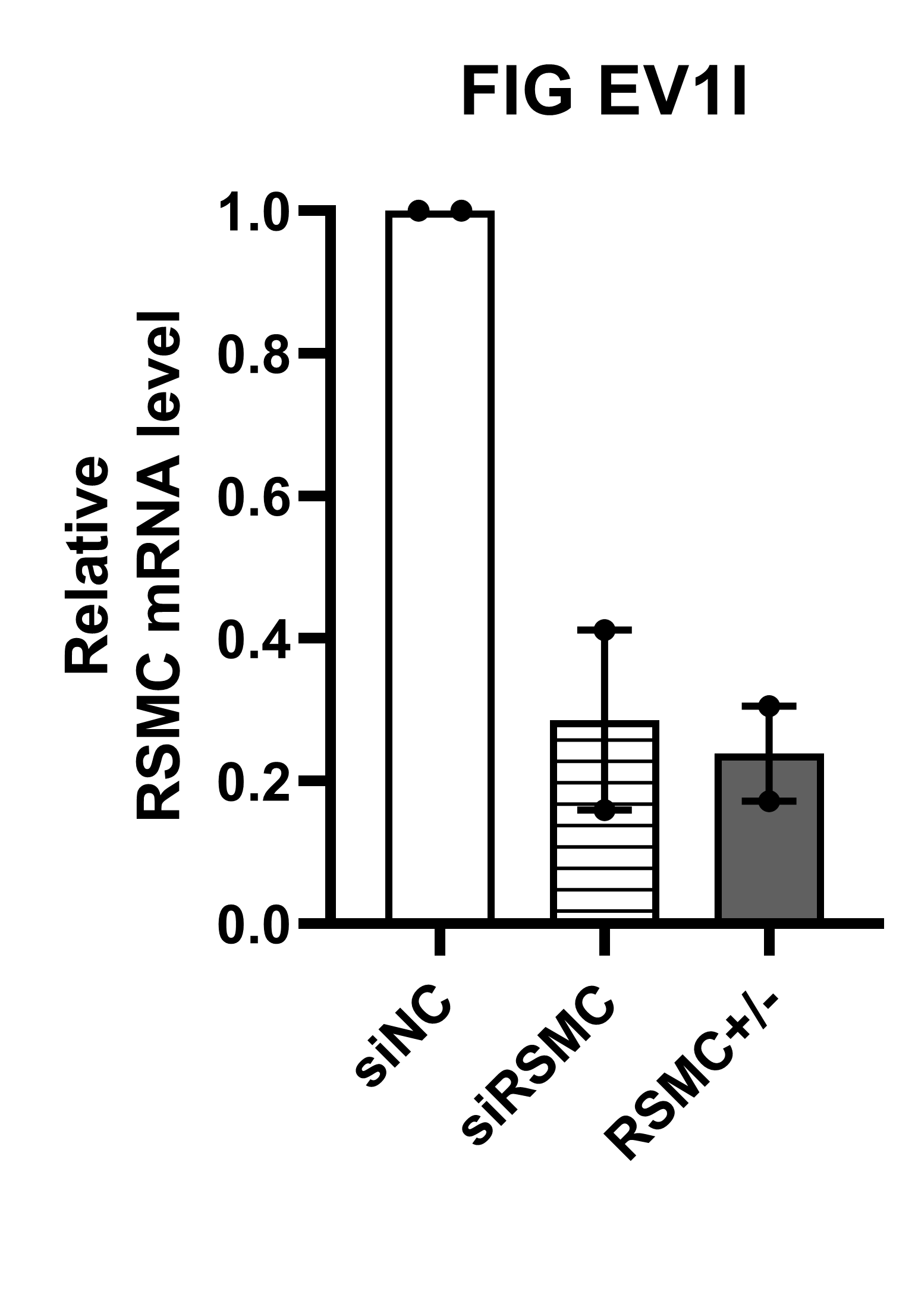

Supplement: Supplementary file 9 — Figure EV1-5 Source Data [file 44318_2025_641_MOESM9_ESM.zip › EMBOJ-2025-120713R_SourceDataForExpandedView/EMBOJ-2025-120713R_SourceDataForFigureEV1/FIG EV1I/FIG EV1I before PS.tif]

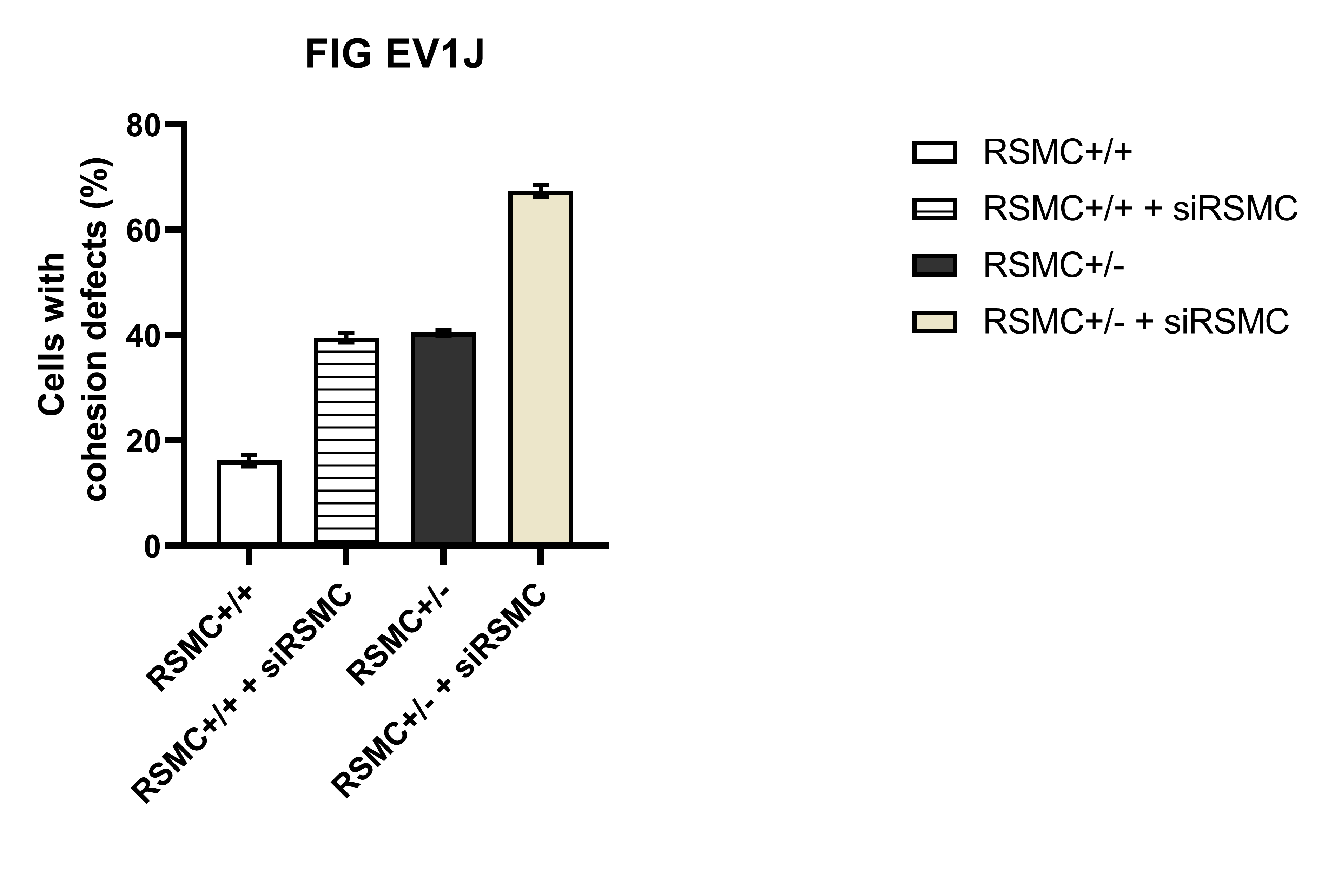

Supplement: Supplementary file 9 — Figure EV1-5 Source Data [file 44318_2025_641_MOESM9_ESM.zip › EMBOJ-2025-120713R_SourceDataForExpandedView/EMBOJ-2025-120713R_SourceDataForFigureEV1/FIG EV1J/FIG EV1J before PS.tif]

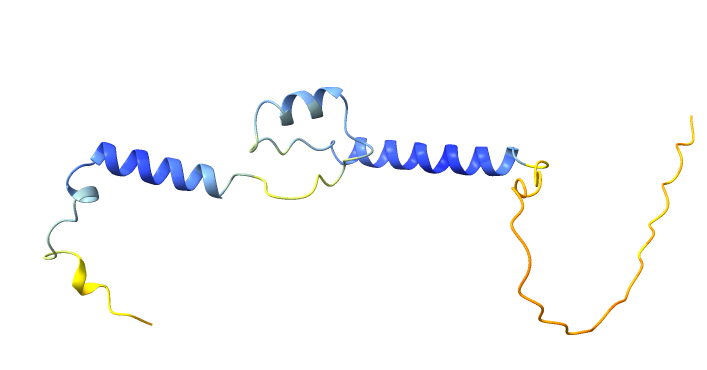

Supplement: Supplementary file 9 — Figure EV1-5 Source Data [file 44318_2025_641_MOESM9_ESM.zip › EMBOJ-2025-120713R_SourceDataForExpandedView/EMBOJ-2025-120713R_SourceDataForFigureEV2/FIG EV2A/RSMC.png]

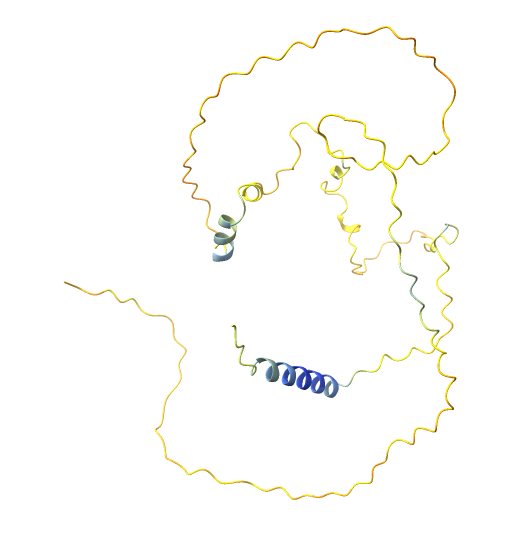

Supplement: Supplementary file 9 — Figure EV1-5 Source Data [file 44318_2025_641_MOESM9_ESM.zip › EMBOJ-2025-120713R_SourceDataForExpandedView/EMBOJ-2025-120713R_SourceDataForFigureEV2/FIG EV2A/Sororin.png]

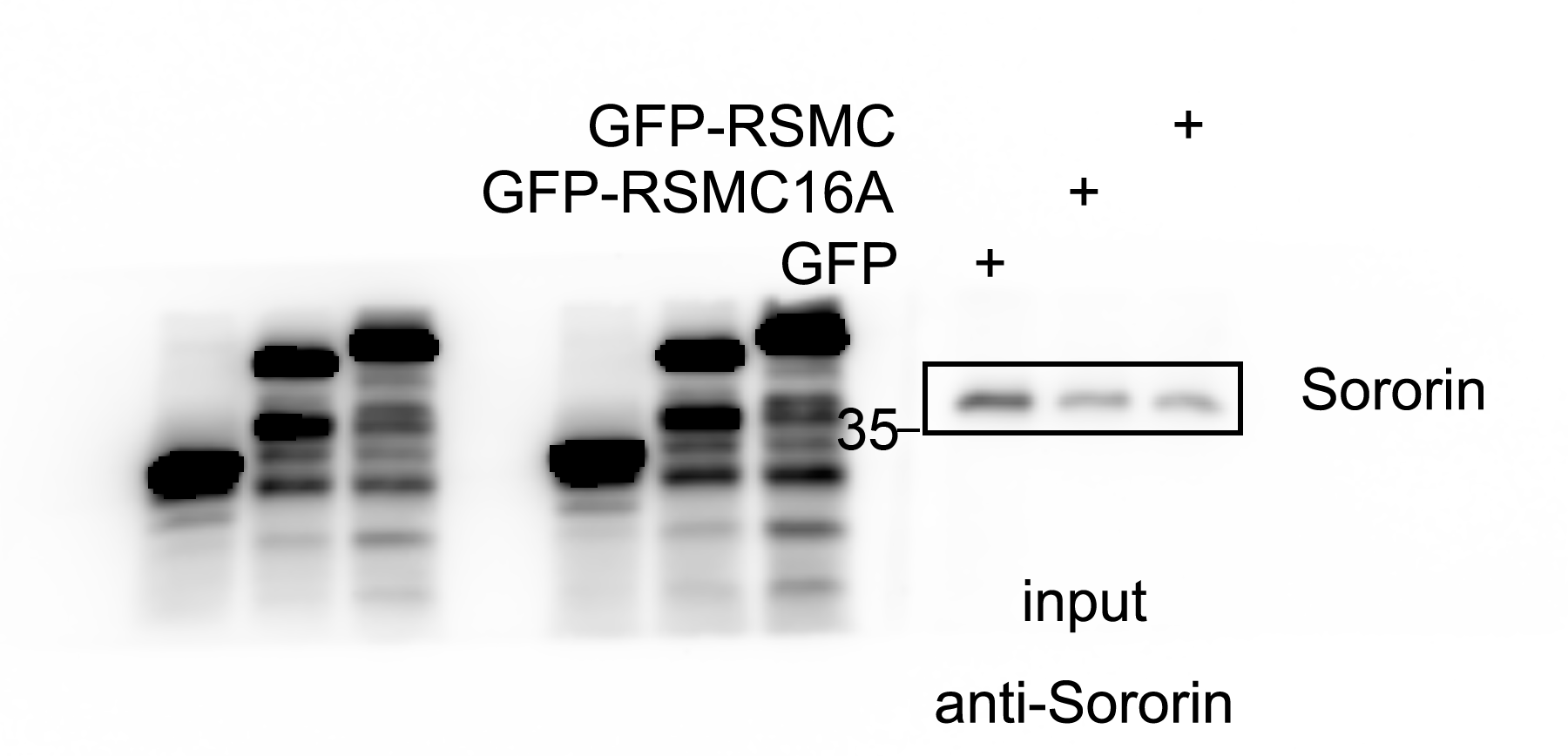

Supplement: Supplementary file 9 — Figure EV1-5 Source Data [file 44318_2025_641_MOESM9_ESM.zip › EMBOJ-2025-120713R_SourceDataForExpandedView/EMBOJ-2025-120713R_SourceDataForFigureEV2/FIG EV2E/gfp ip input sororin.tif]

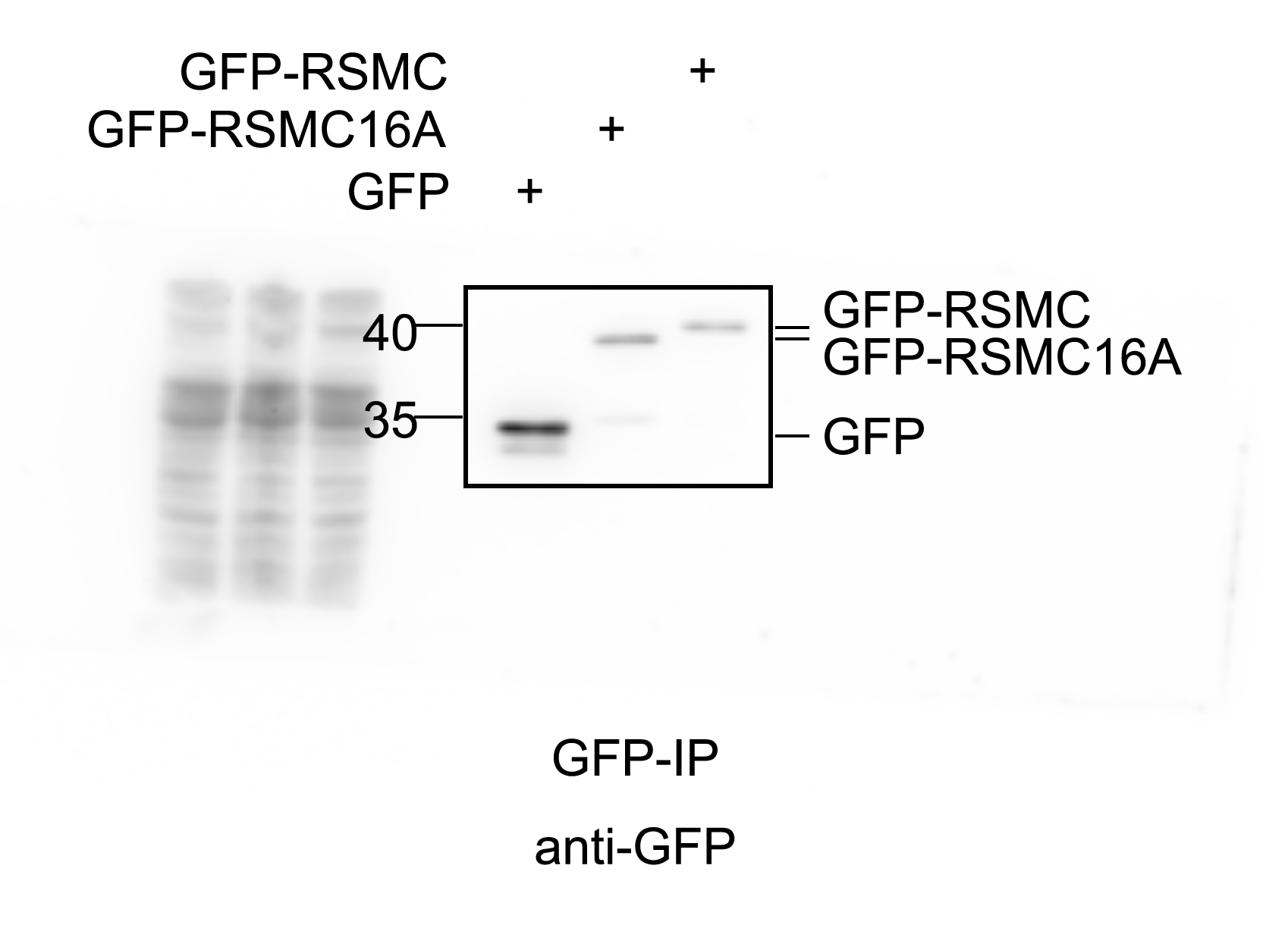

Supplement: Supplementary file 9 — Figure EV1-5 Source Data [file 44318_2025_641_MOESM9_ESM.zip › EMBOJ-2025-120713R_SourceDataForExpandedView/EMBOJ-2025-120713R_SourceDataForFigureEV2/FIG EV2E/Sourcedata- GFP-IP anti-GFP.tif]

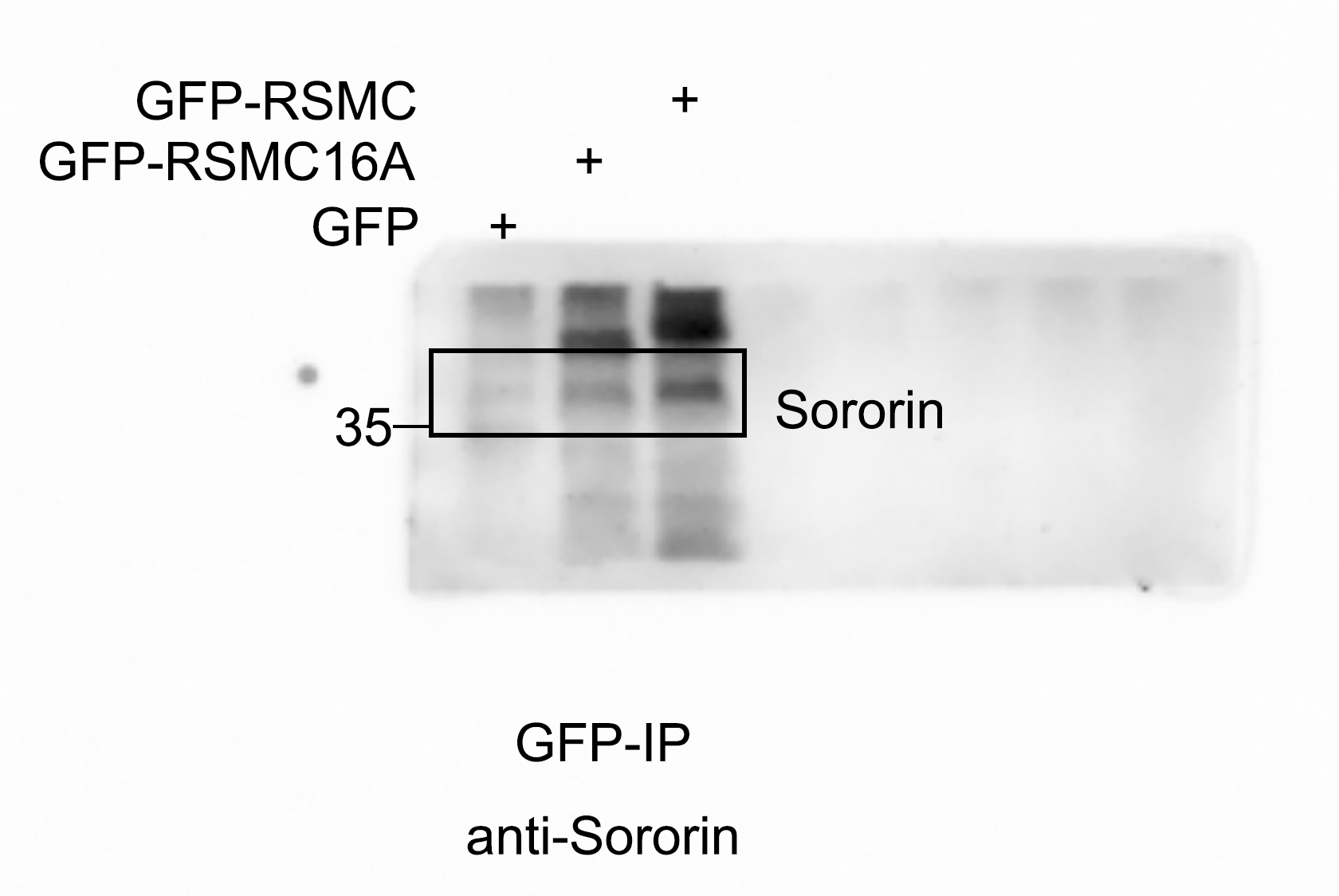

Supplement: Supplementary file 9 — Figure EV1-5 Source Data [file 44318_2025_641_MOESM9_ESM.zip › EMBOJ-2025-120713R_SourceDataForExpandedView/EMBOJ-2025-120713R_SourceDataForFigureEV2/FIG EV2E/Sourcedata- GFP-IP anti-Sororin.tif]

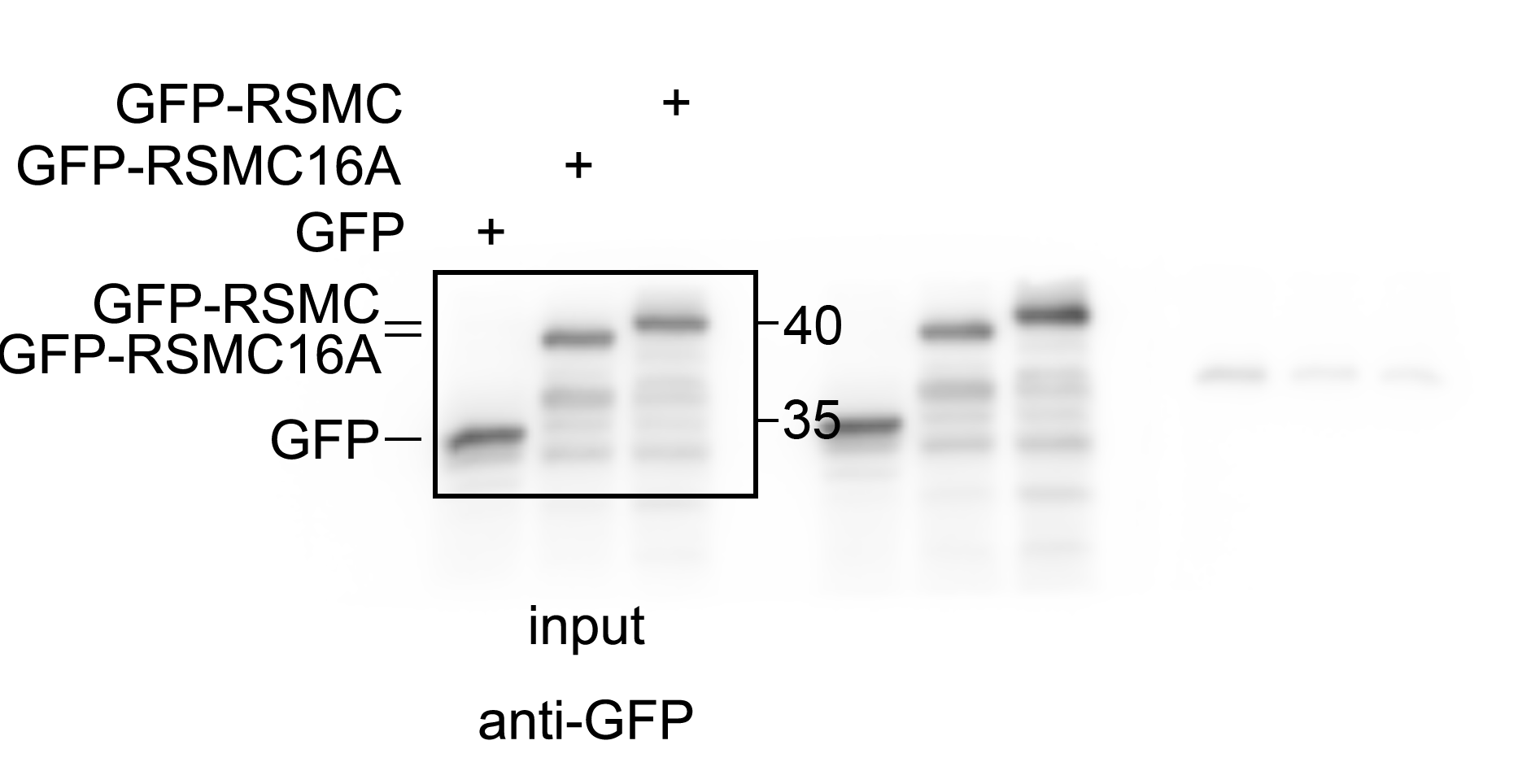

Supplement: Supplementary file 9 — Figure EV1-5 Source Data [file 44318_2025_641_MOESM9_ESM.zip › EMBOJ-2025-120713R_SourceDataForExpandedView/EMBOJ-2025-120713R_SourceDataForFigureEV2/FIG EV2E/Sourcedata- input anti-GFP.tif]

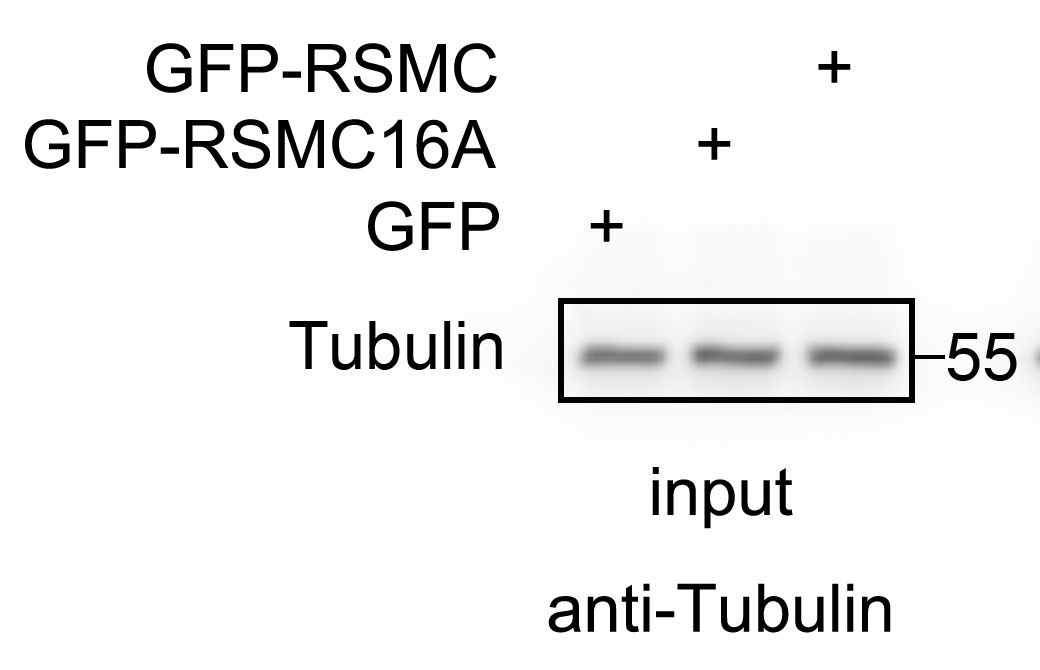

Supplement: Supplementary file 9 — Figure EV1-5 Source Data [file 44318_2025_641_MOESM9_ESM.zip › EMBOJ-2025-120713R_SourceDataForExpandedView/EMBOJ-2025-120713R_SourceDataForFigureEV2/FIG EV2E/Sourcedata-input anti-Tubulin.tif]

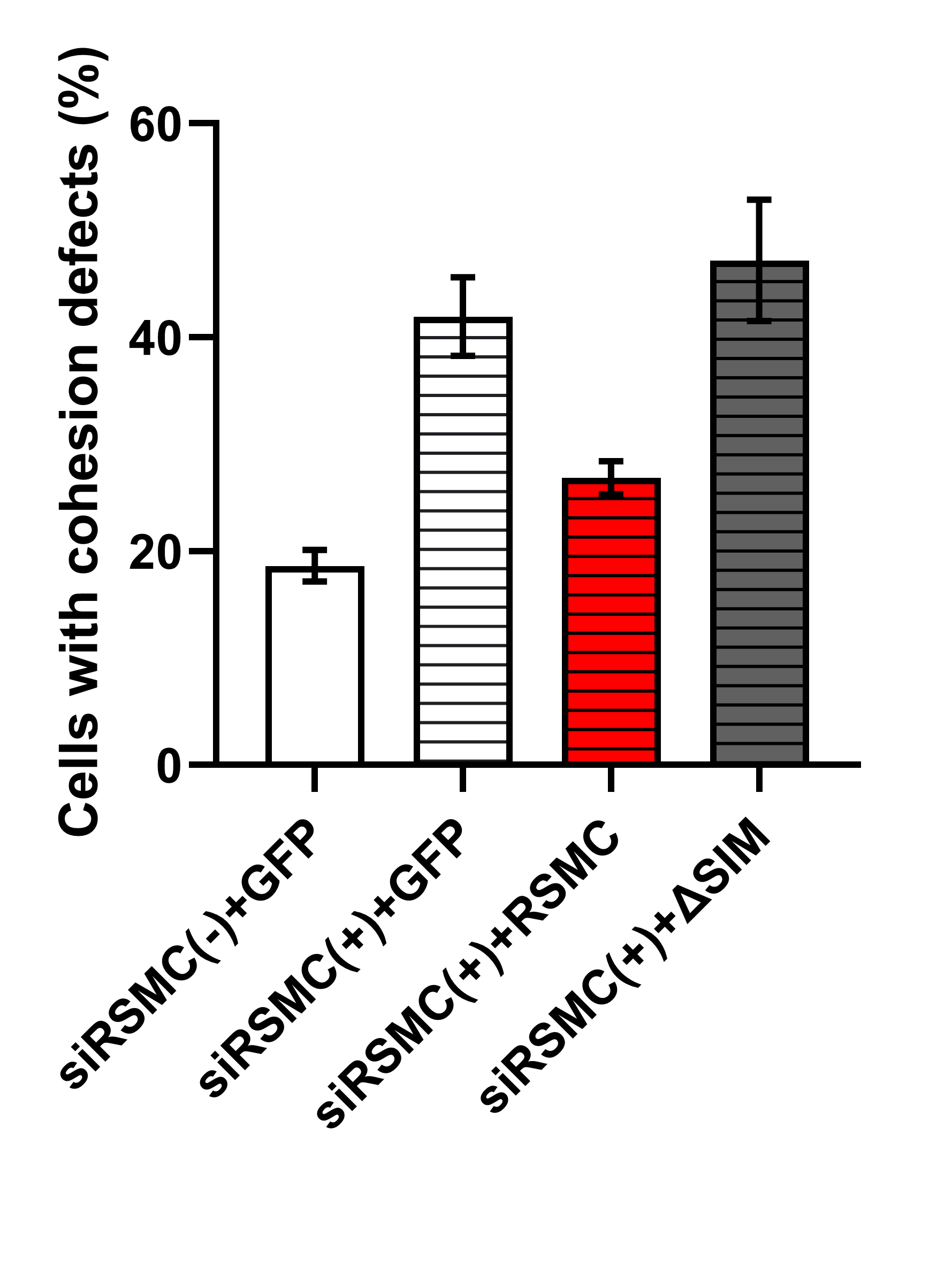

Supplement: Supplementary file 9 — Figure EV1-5 Source Data [file 44318_2025_641_MOESM9_ESM.zip › EMBOJ-2025-120713R_SourceDataForExpandedView/EMBOJ-2025-120713R_SourceDataForFigureEV2/FIG EV2F/FIG EV2F before PS.tif]

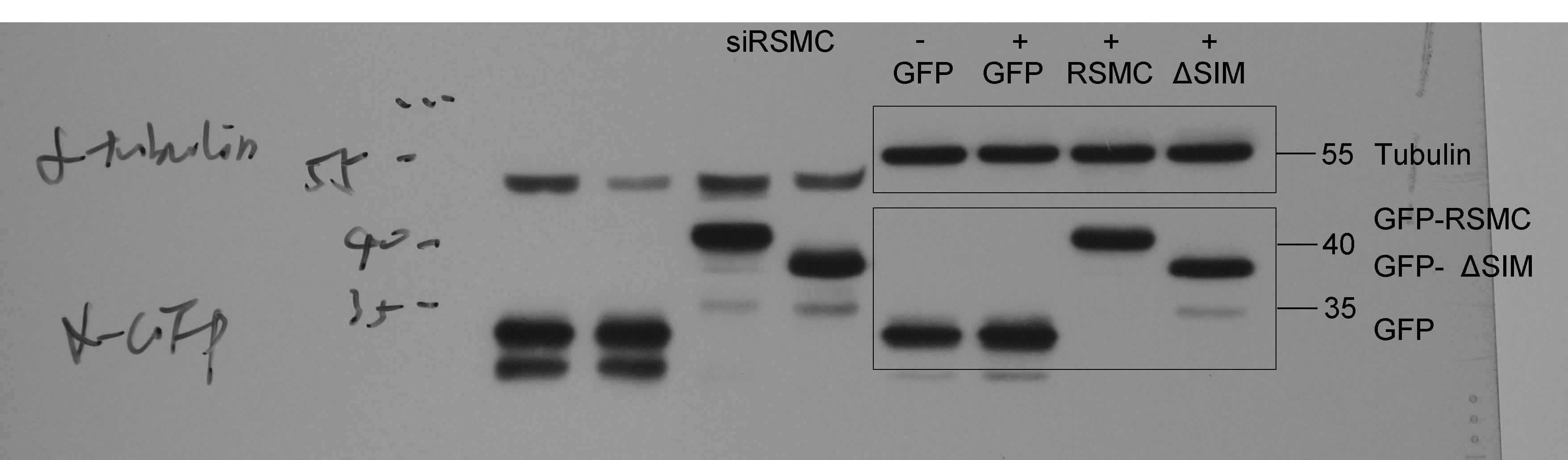

Supplement: Supplementary file 9 — Figure EV1-5 Source Data [file 44318_2025_641_MOESM9_ESM.zip › EMBOJ-2025-120713R_SourceDataForExpandedView/EMBOJ-2025-120713R_SourceDataForFigureEV2/FIG EV2F/FIG EV3F-SourceData.tif]

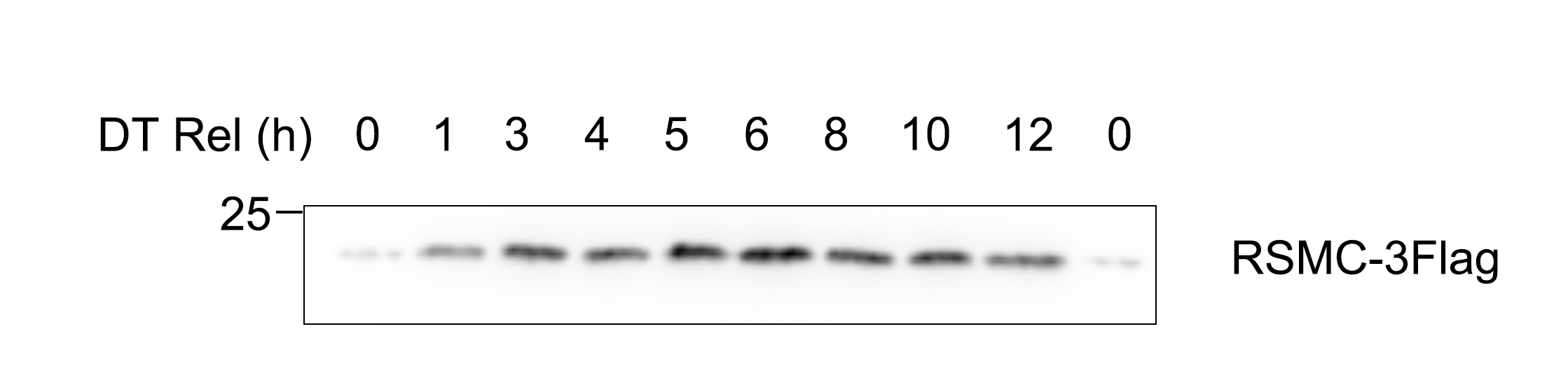

Supplement: Supplementary file 9 — Figure EV1-5 Source Data [file 44318_2025_641_MOESM9_ESM.zip › EMBOJ-2025-120713R_SourceDataForExpandedView/EMBOJ-2025-120713R_SourceDataForFigureEV3/FIG EV3A/FLAG-SourceData.tif]

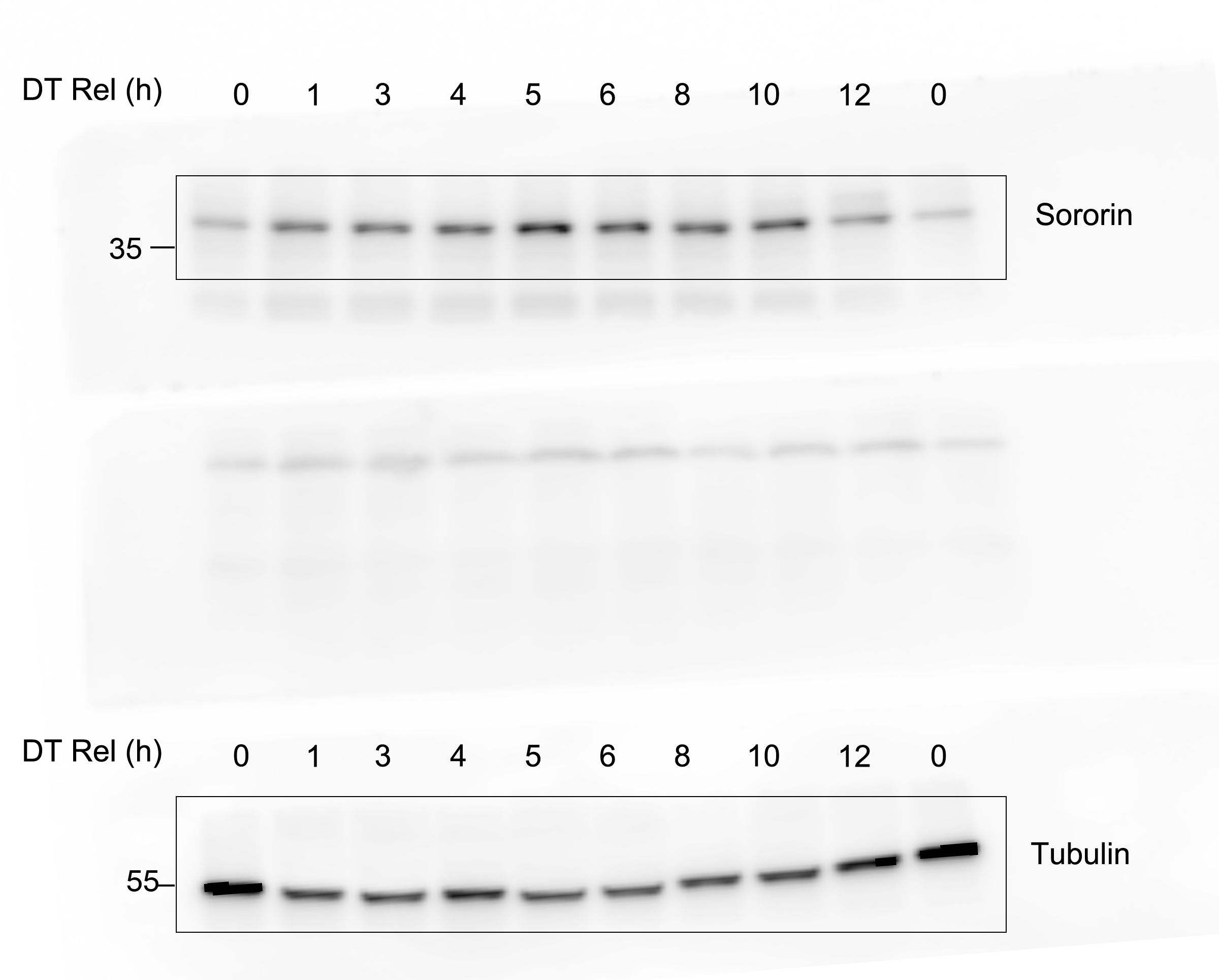

Supplement: Supplementary file 9 — Figure EV1-5 Source Data [file 44318_2025_641_MOESM9_ESM.zip › EMBOJ-2025-120713R_SourceDataForExpandedView/EMBOJ-2025-120713R_SourceDataForFigureEV3/FIG EV3A/Sororin-tubulin SourceData.tif]

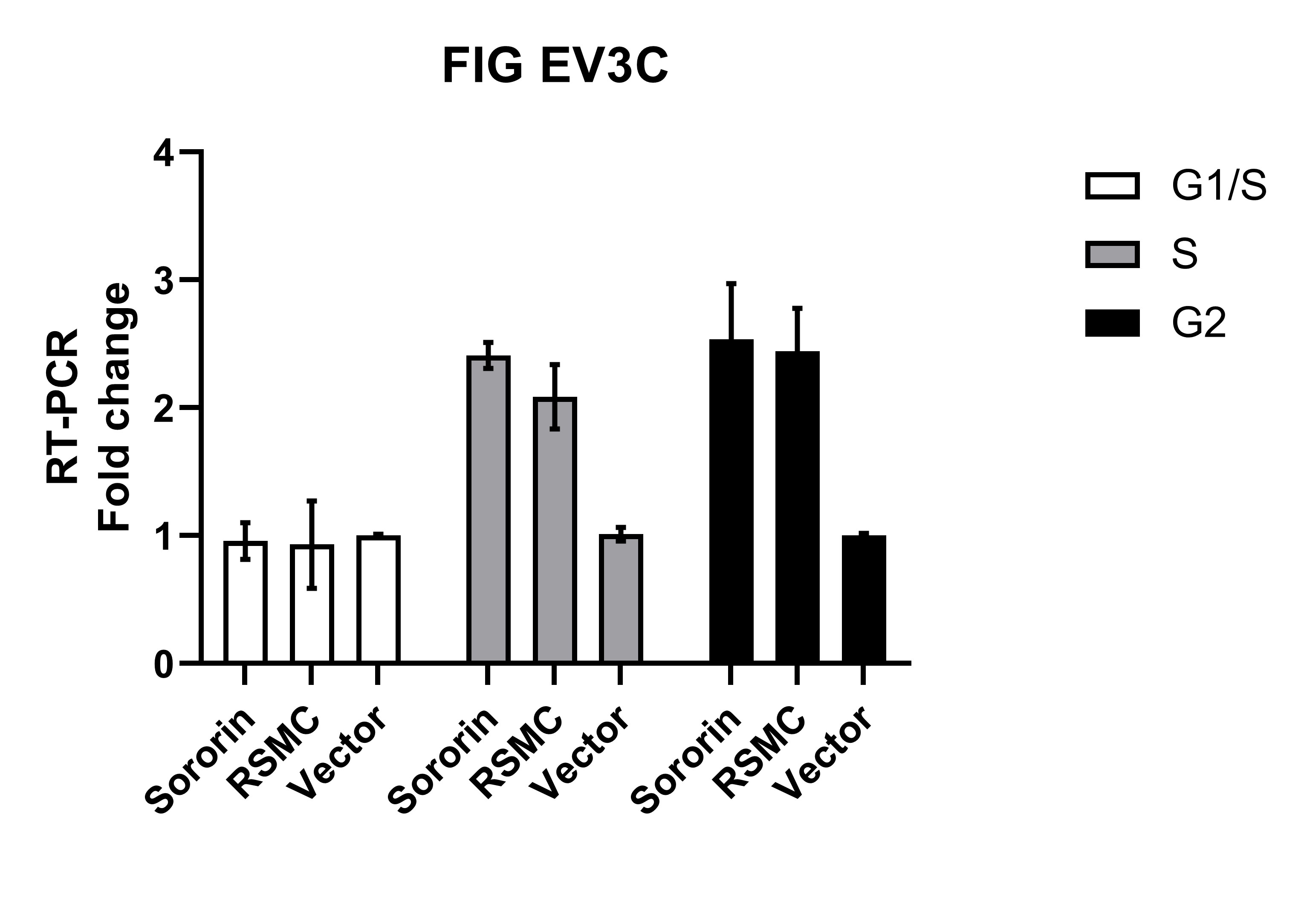

Supplement: Supplementary file 9 — Figure EV1-5 Source Data [file 44318_2025_641_MOESM9_ESM.zip › EMBOJ-2025-120713R_SourceDataForExpandedView/EMBOJ-2025-120713R_SourceDataForFigureEV3/FIG EV3C/FIG EV3C before PS.tif]

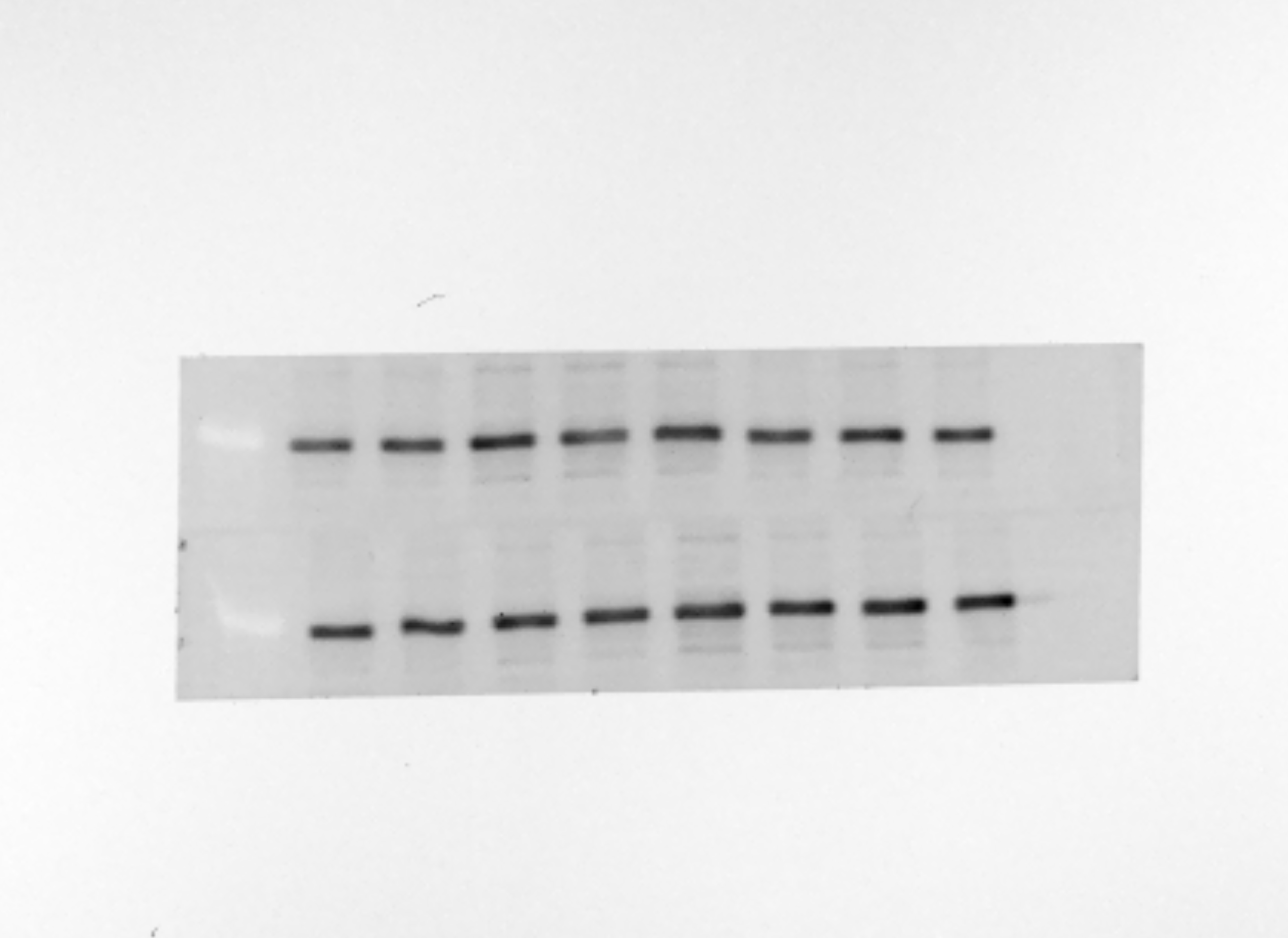

Supplement: Supplementary file 9 — Figure EV1-5 Source Data [file 44318_2025_641_MOESM9_ESM.zip › EMBOJ-2025-120713R_SourceDataForExpandedView/EMBOJ-2025-120713R_SourceDataForFigureEV3/FIG EV3E/EXP1/H3 export from Image J .png]

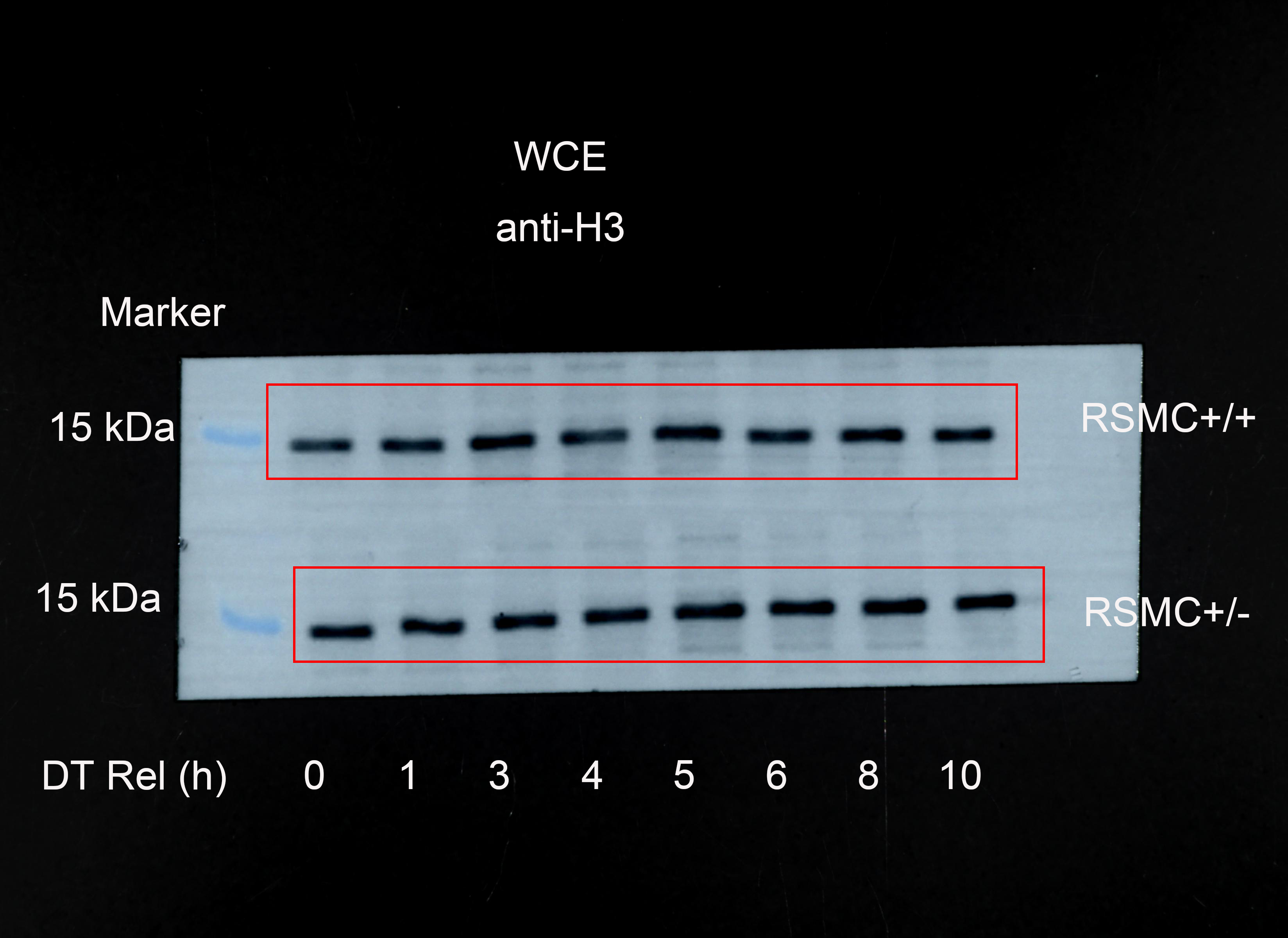

Supplement: Supplementary file 9 — Figure EV1-5 Source Data [file 44318_2025_641_MOESM9_ESM.zip › EMBOJ-2025-120713R_SourceDataForExpandedView/EMBOJ-2025-120713R_SourceDataForFigureEV3/FIG EV3E/EXP1/H3 Merge with protein Marker.tif]

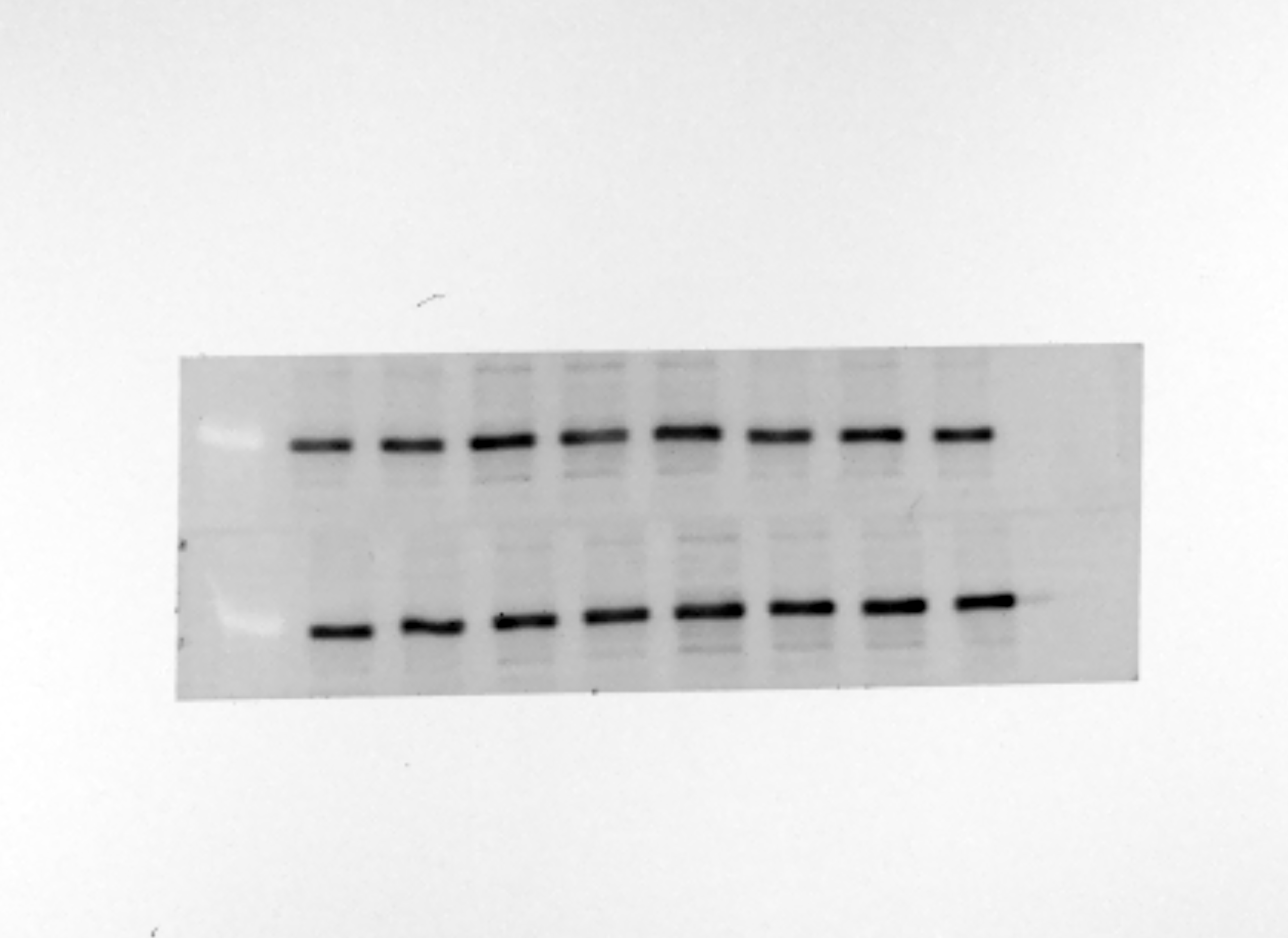

Supplement: Supplementary file 9 — Figure EV1-5 Source Data [file 44318_2025_641_MOESM9_ESM.zip › EMBOJ-2025-120713R_SourceDataForExpandedView/EMBOJ-2025-120713R_SourceDataForFigureEV3/FIG EV3E/EXP1/H3 RAW data.tif]

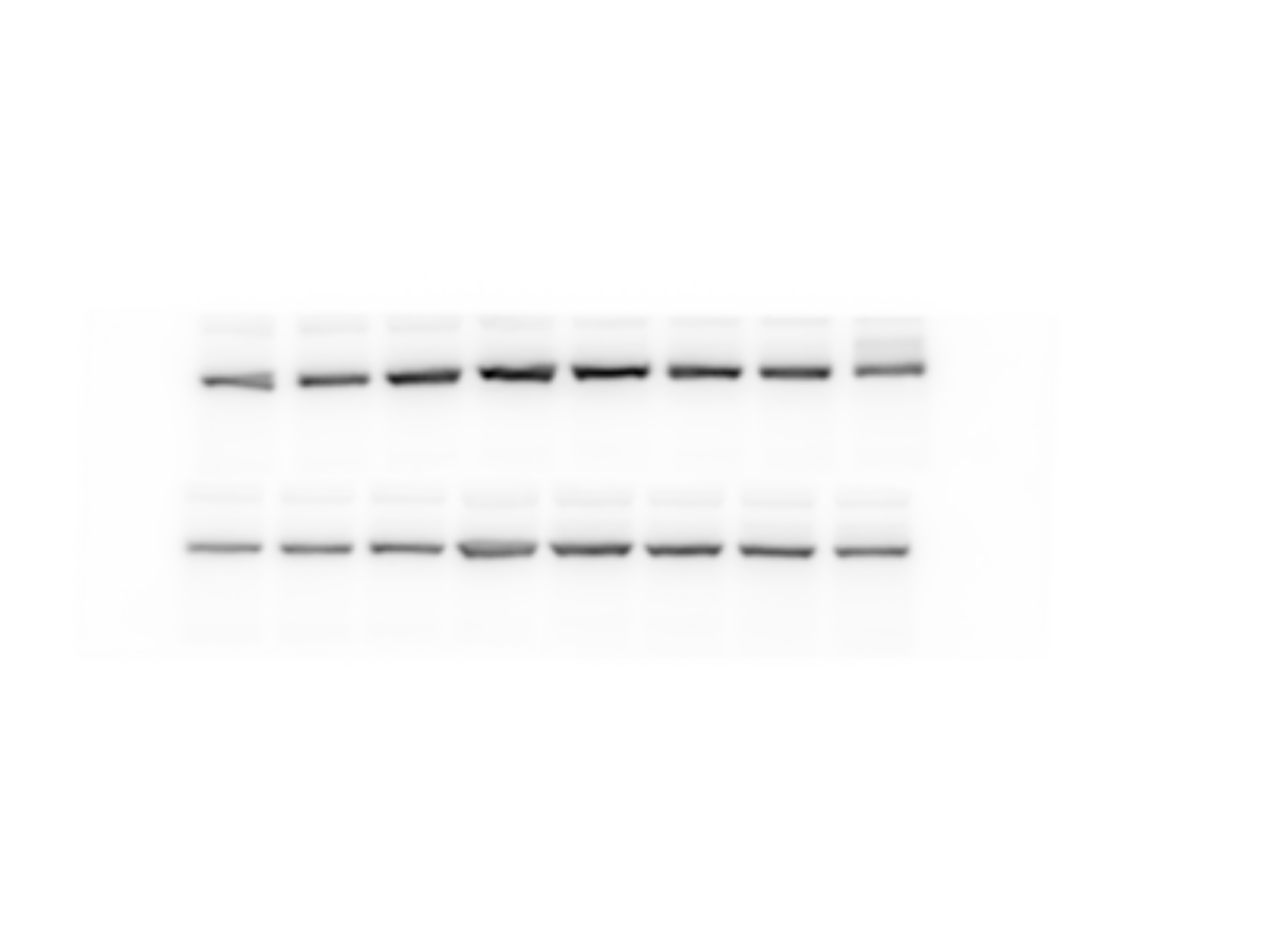

Supplement: Supplementary file 9 — Figure EV1-5 Source Data [file 44318_2025_641_MOESM9_ESM.zip › EMBOJ-2025-120713R_SourceDataForExpandedView/EMBOJ-2025-120713R_SourceDataForFigureEV3/FIG EV3E/EXP1/Sororin export from Image J.png]

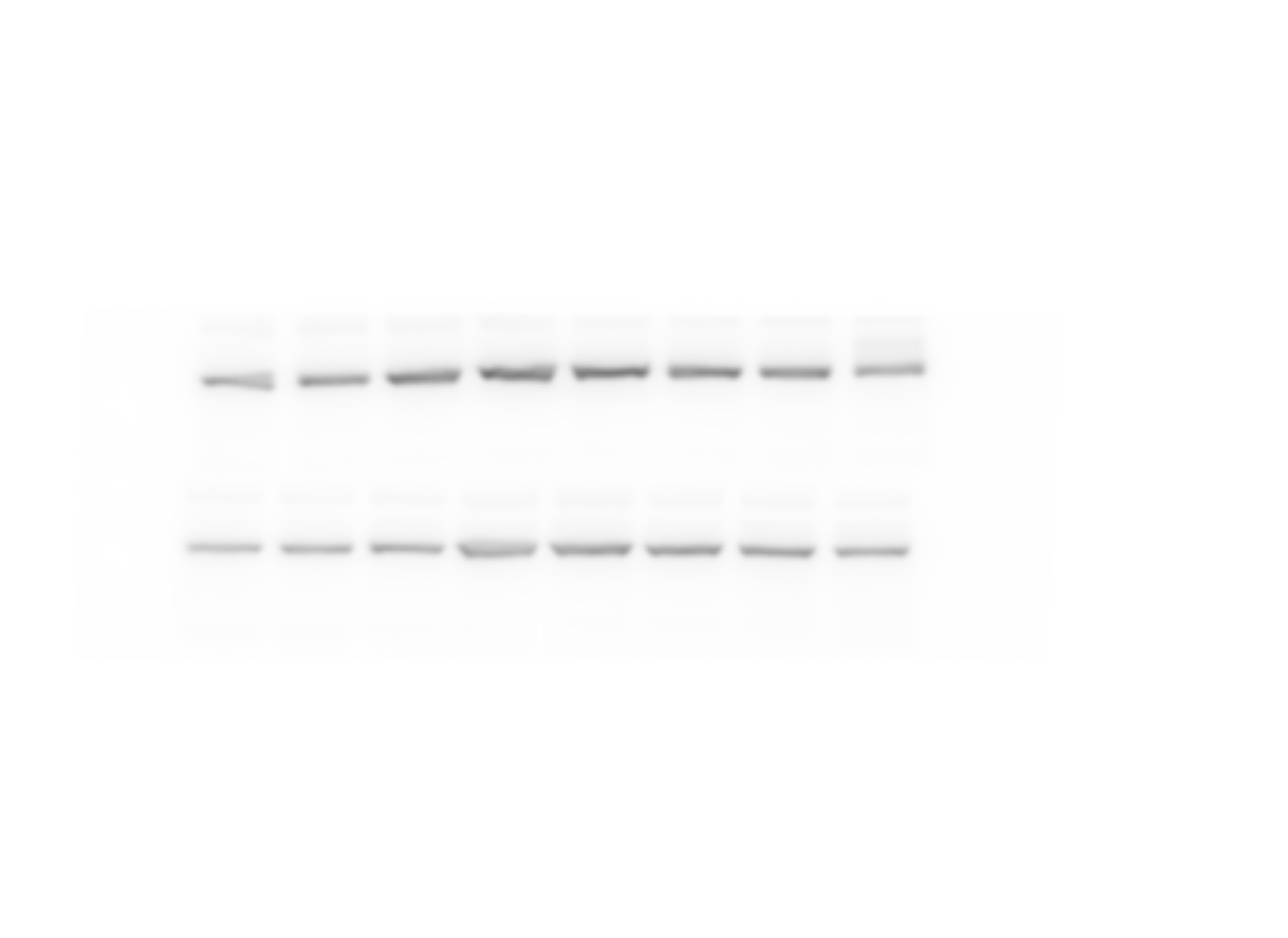

Supplement: Supplementary file 9 — Figure EV1-5 Source Data [file 44318_2025_641_MOESM9_ESM.zip › EMBOJ-2025-120713R_SourceDataForExpandedView/EMBOJ-2025-120713R_SourceDataForFigureEV3/FIG EV3E/EXP1/sororin RAW data.tif]

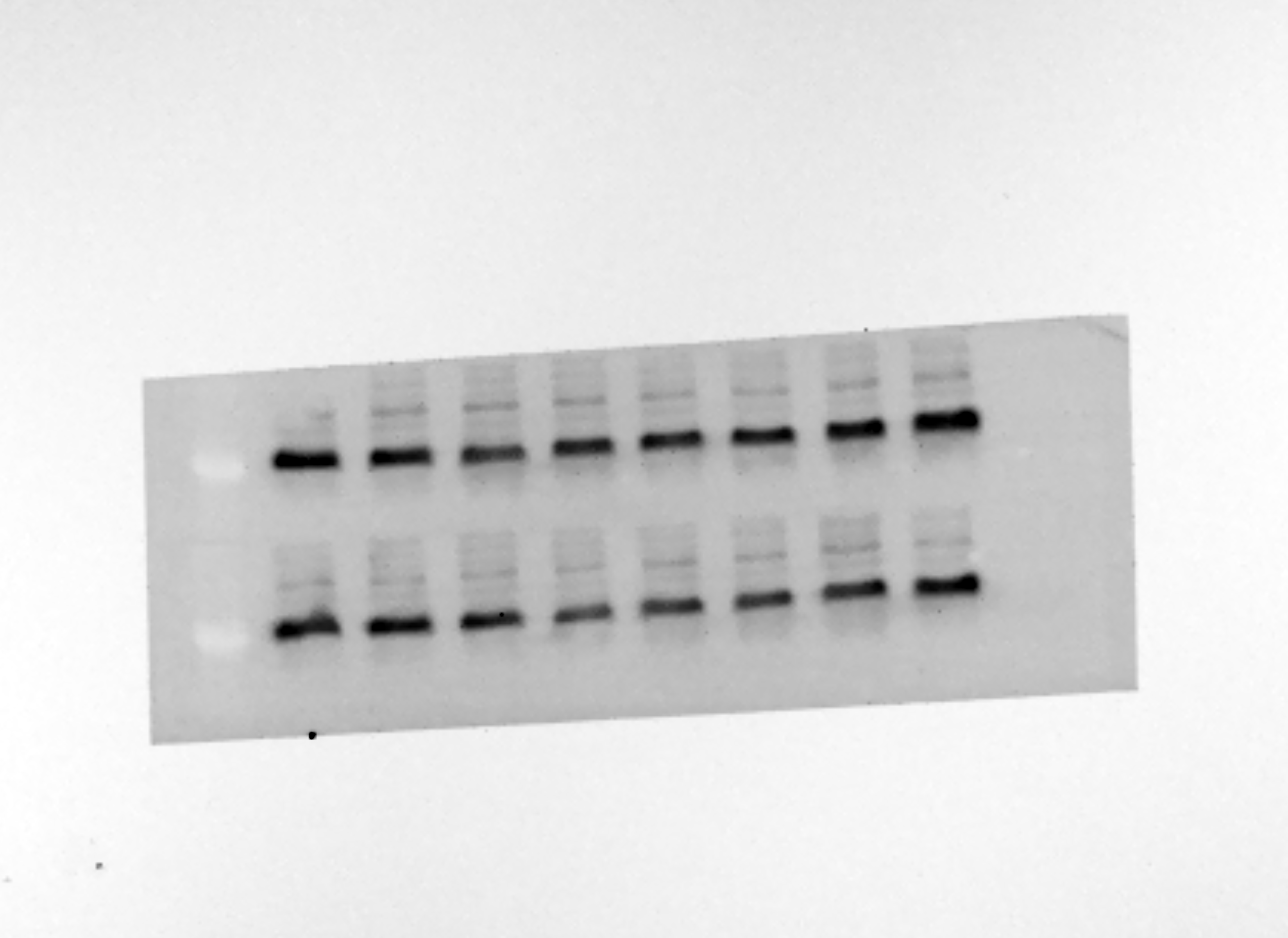

Supplement: Supplementary file 9 — Figure EV1-5 Source Data [file 44318_2025_641_MOESM9_ESM.zip › EMBOJ-2025-120713R_SourceDataForExpandedView/EMBOJ-2025-120713R_SourceDataForFigureEV3/FIG EV3E/EXP2/H3 RAW data.tif]

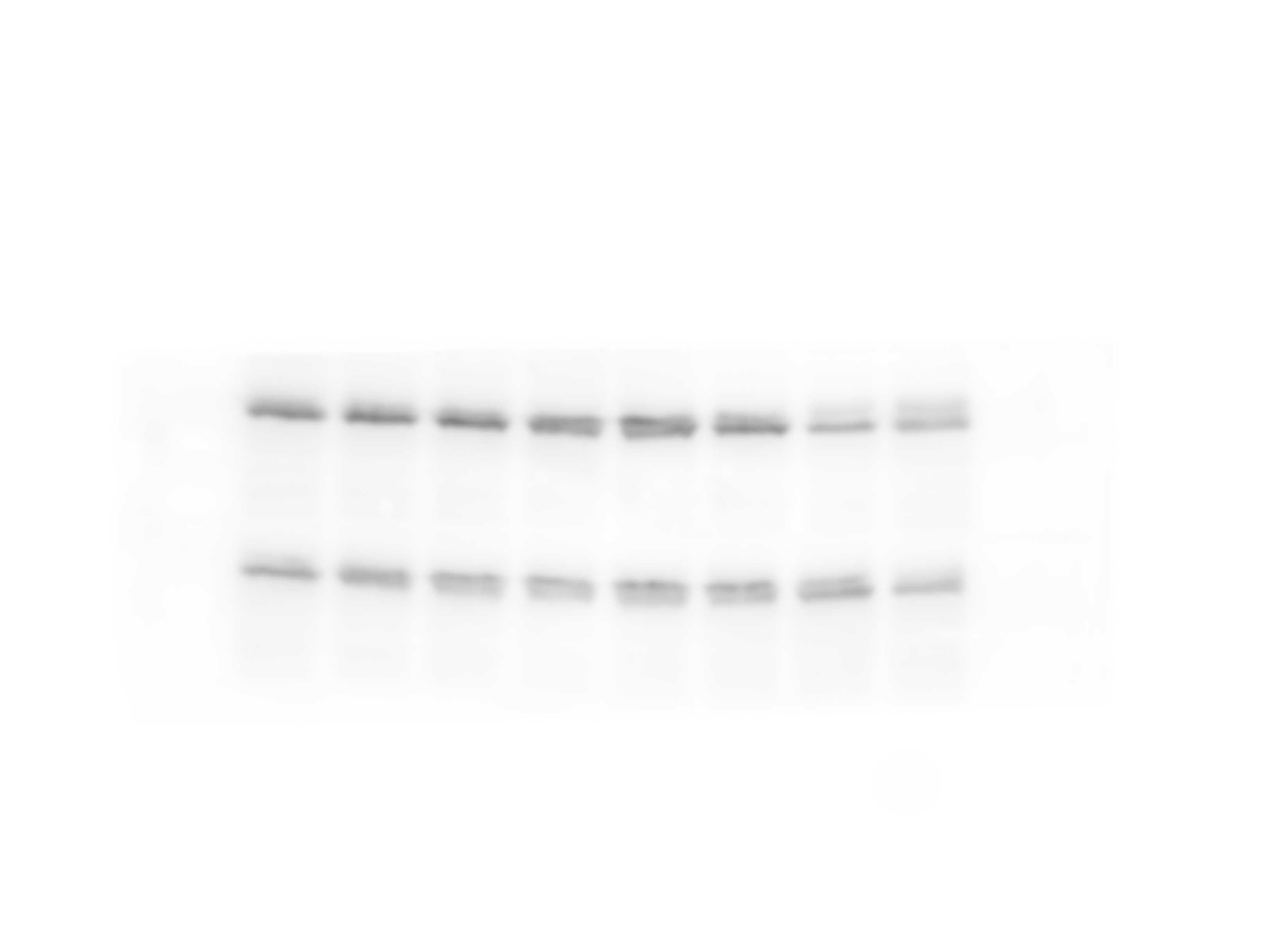

Supplement: Supplementary file 9 — Figure EV1-5 Source Data [file 44318_2025_641_MOESM9_ESM.zip › EMBOJ-2025-120713R_SourceDataForExpandedView/EMBOJ-2025-120713R_SourceDataForFigureEV3/FIG EV3E/EXP2/sororin RAW data.tif]

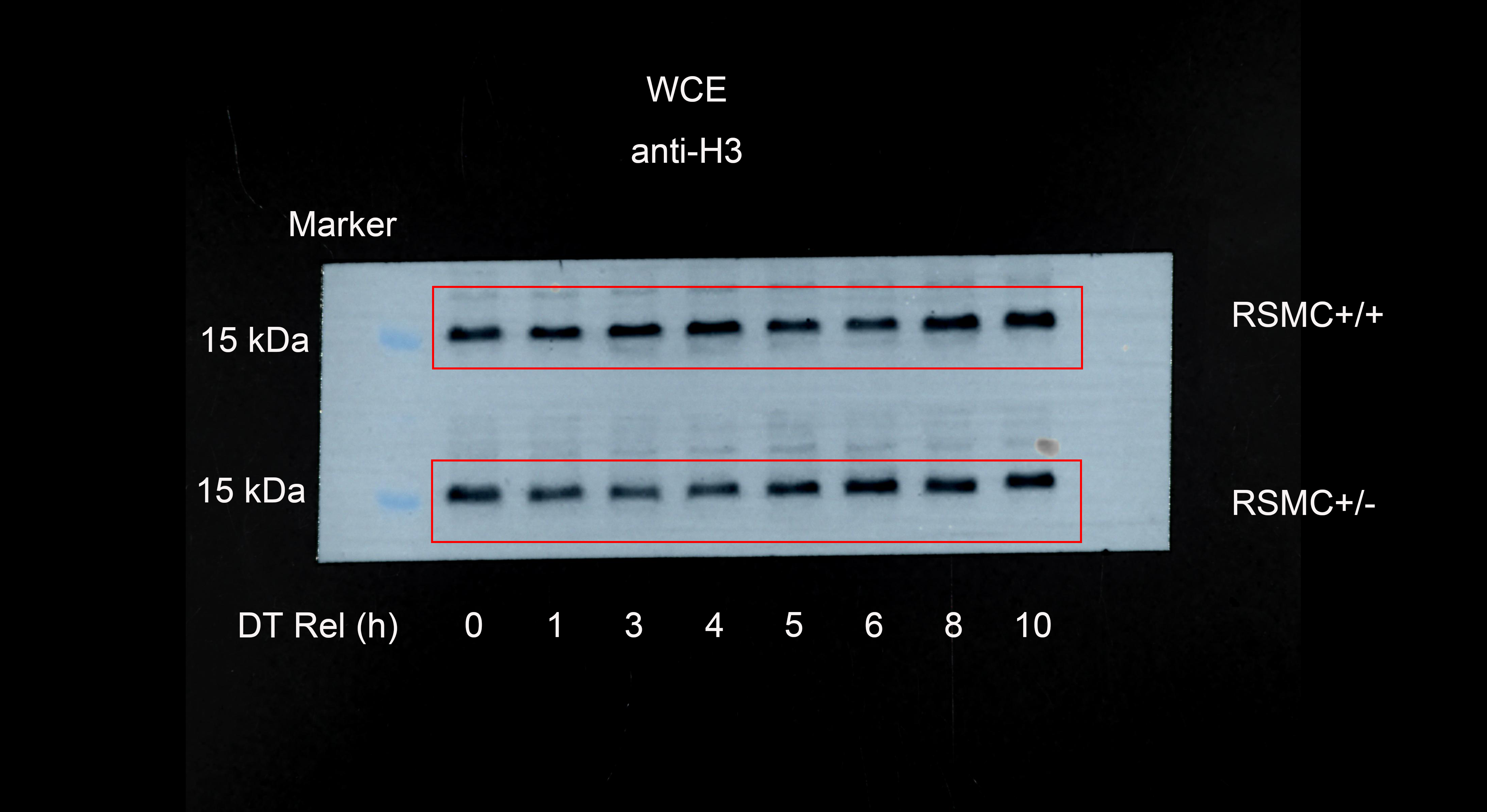

Supplement: Supplementary file 9 — Figure EV1-5 Source Data [file 44318_2025_641_MOESM9_ESM.zip › EMBOJ-2025-120713R_SourceDataForExpandedView/EMBOJ-2025-120713R_SourceDataForFigureEV3/FIG EV3E/EXP3/H3 Merge with protein Marker.tif]

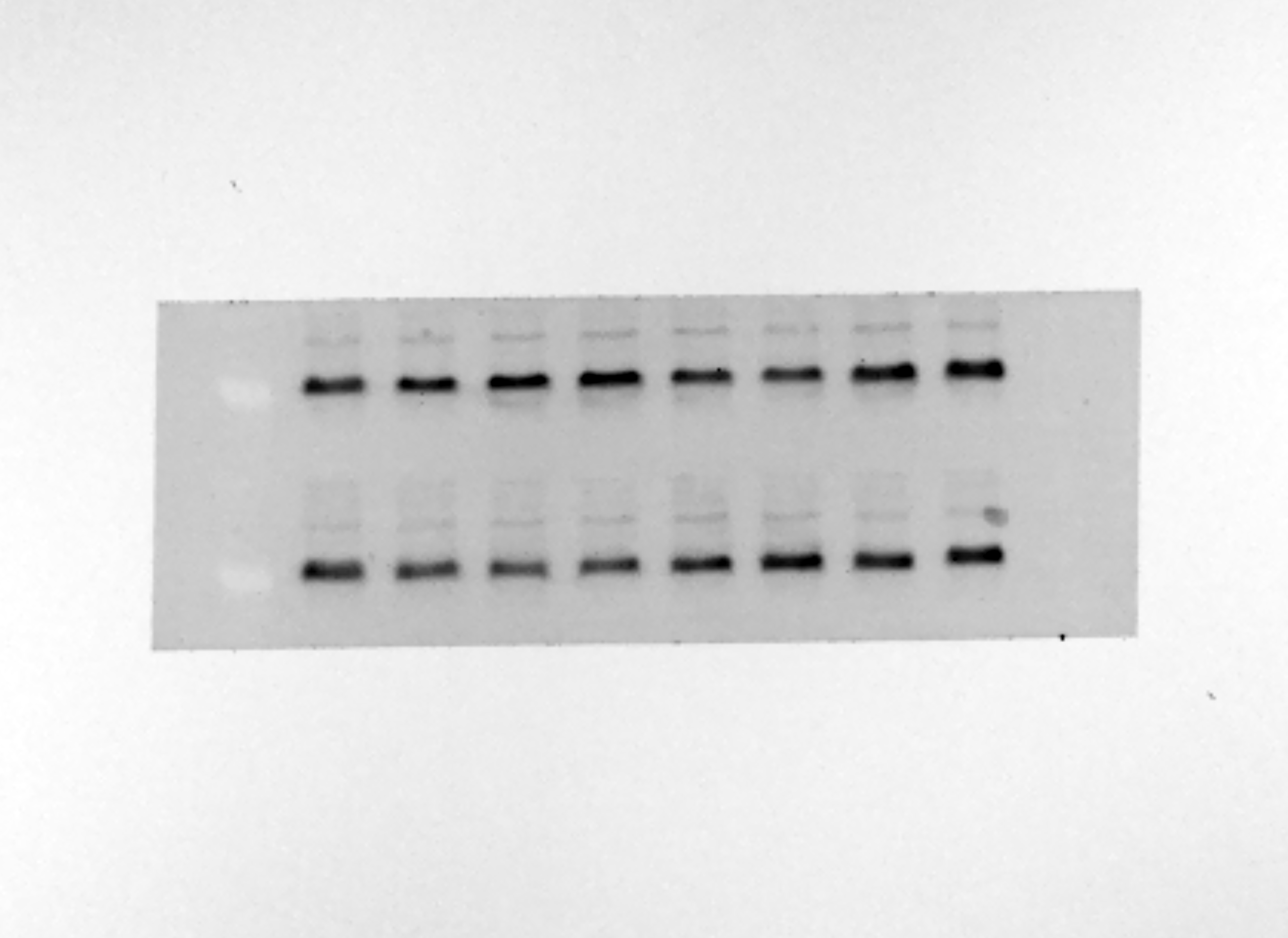

Supplement: Supplementary file 9 — Figure EV1-5 Source Data [file 44318_2025_641_MOESM9_ESM.zip › EMBOJ-2025-120713R_SourceDataForExpandedView/EMBOJ-2025-120713R_SourceDataForFigureEV3/FIG EV3E/EXP3/H3 RAW data.tif]

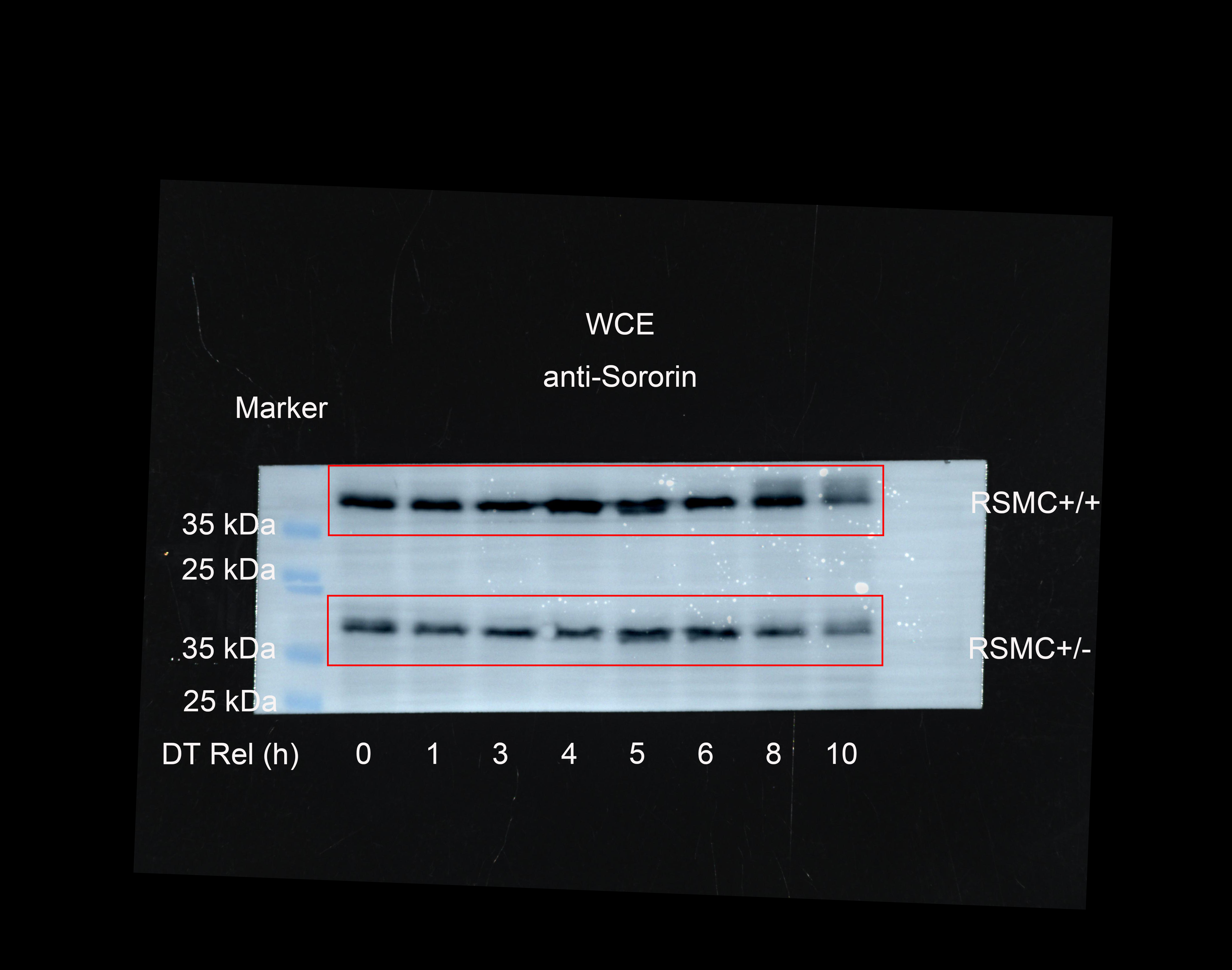

Supplement: Supplementary file 9 — Figure EV1-5 Source Data [file 44318_2025_641_MOESM9_ESM.zip › EMBOJ-2025-120713R_SourceDataForExpandedView/EMBOJ-2025-120713R_SourceDataForFigureEV3/FIG EV3E/EXP3/sororin Merge with protein Marker.tif]

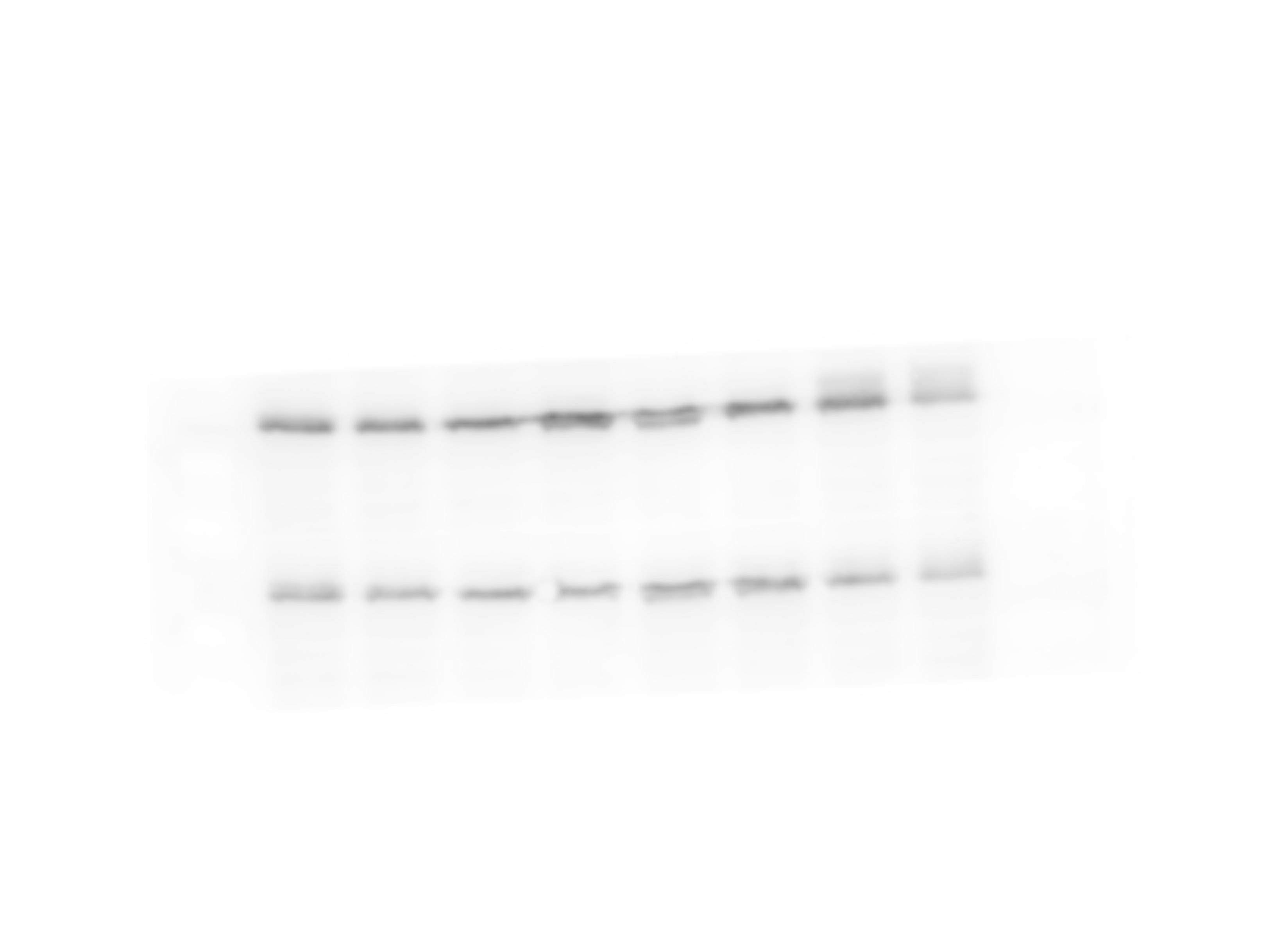

Supplement: Supplementary file 9 — Figure EV1-5 Source Data [file 44318_2025_641_MOESM9_ESM.zip › EMBOJ-2025-120713R_SourceDataForExpandedView/EMBOJ-2025-120713R_SourceDataForFigureEV3/FIG EV3E/EXP3/sororin RAW data.tif]

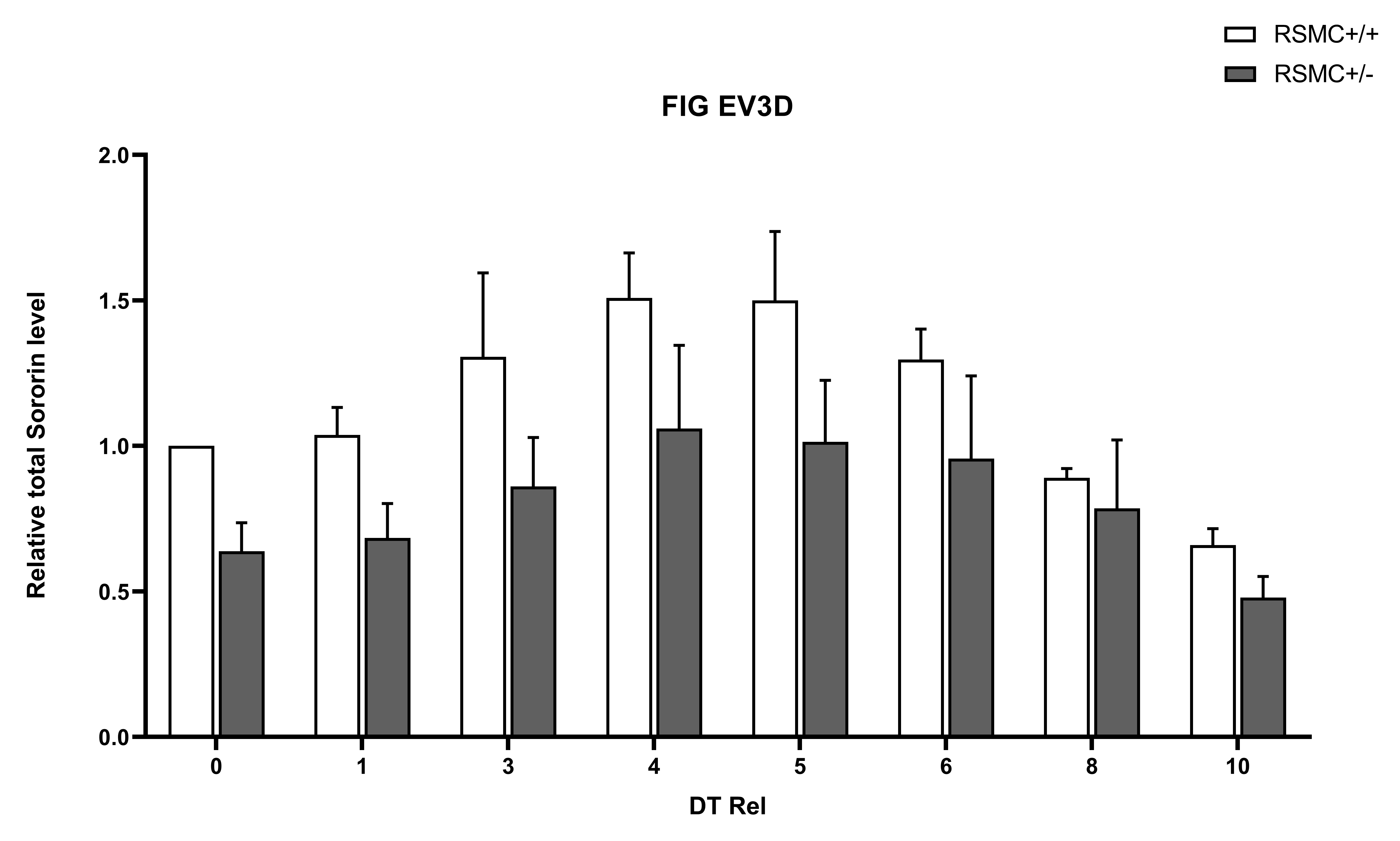

Supplement: Supplementary file 9 — Figure EV1-5 Source Data [file 44318_2025_641_MOESM9_ESM.zip › EMBOJ-2025-120713R_SourceDataForExpandedView/EMBOJ-2025-120713R_SourceDataForFigureEV3/FIG EV3E/FIG EV3E before PS.tif]

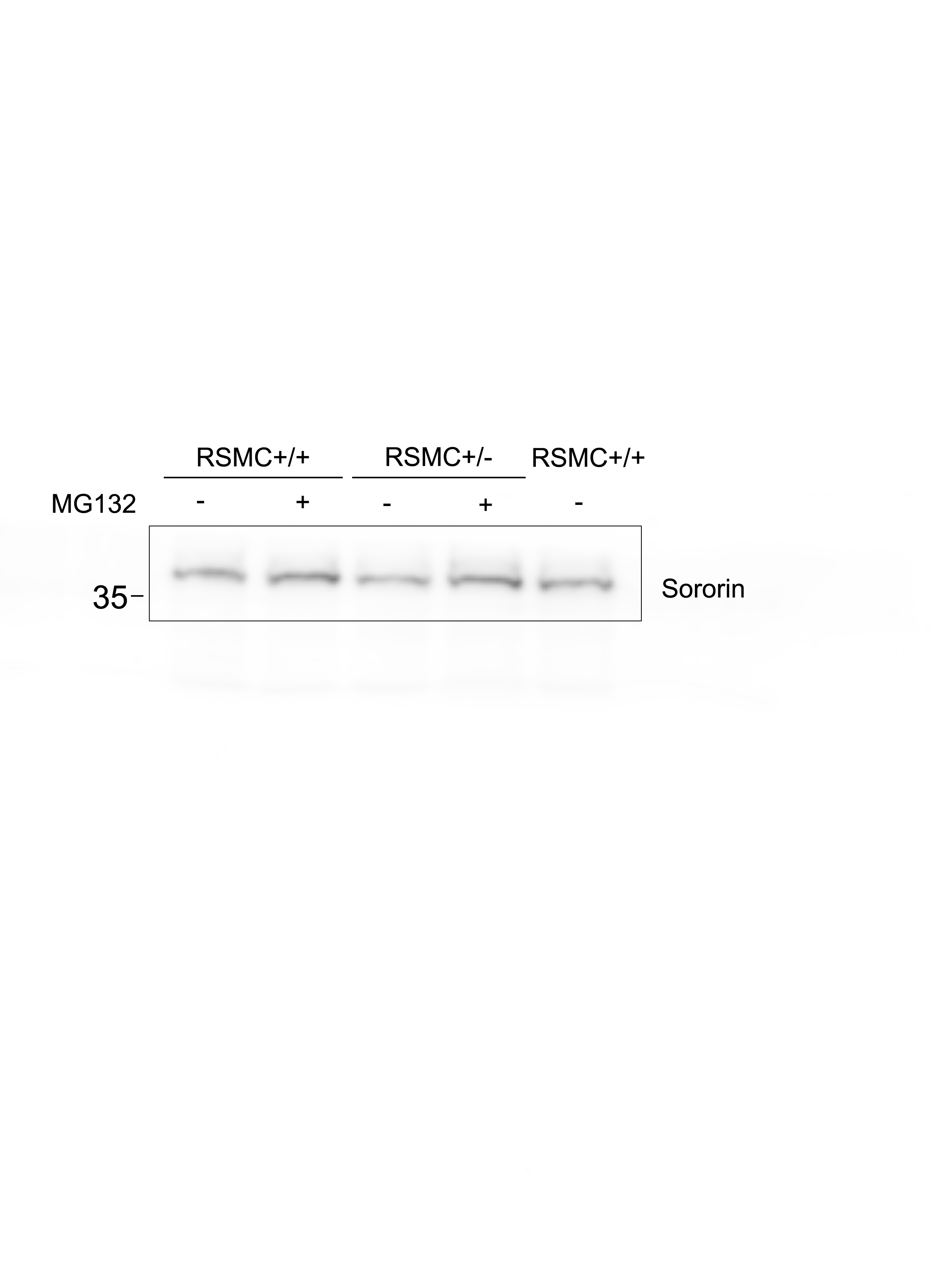

Supplement: Supplementary file 9 — Figure EV1-5 Source Data [file 44318_2025_641_MOESM9_ESM.zip › EMBOJ-2025-120713R_SourceDataForExpandedView/EMBOJ-2025-120713R_SourceDataForFigureEV3/FIG EV3F/sororin SourceData.tif]

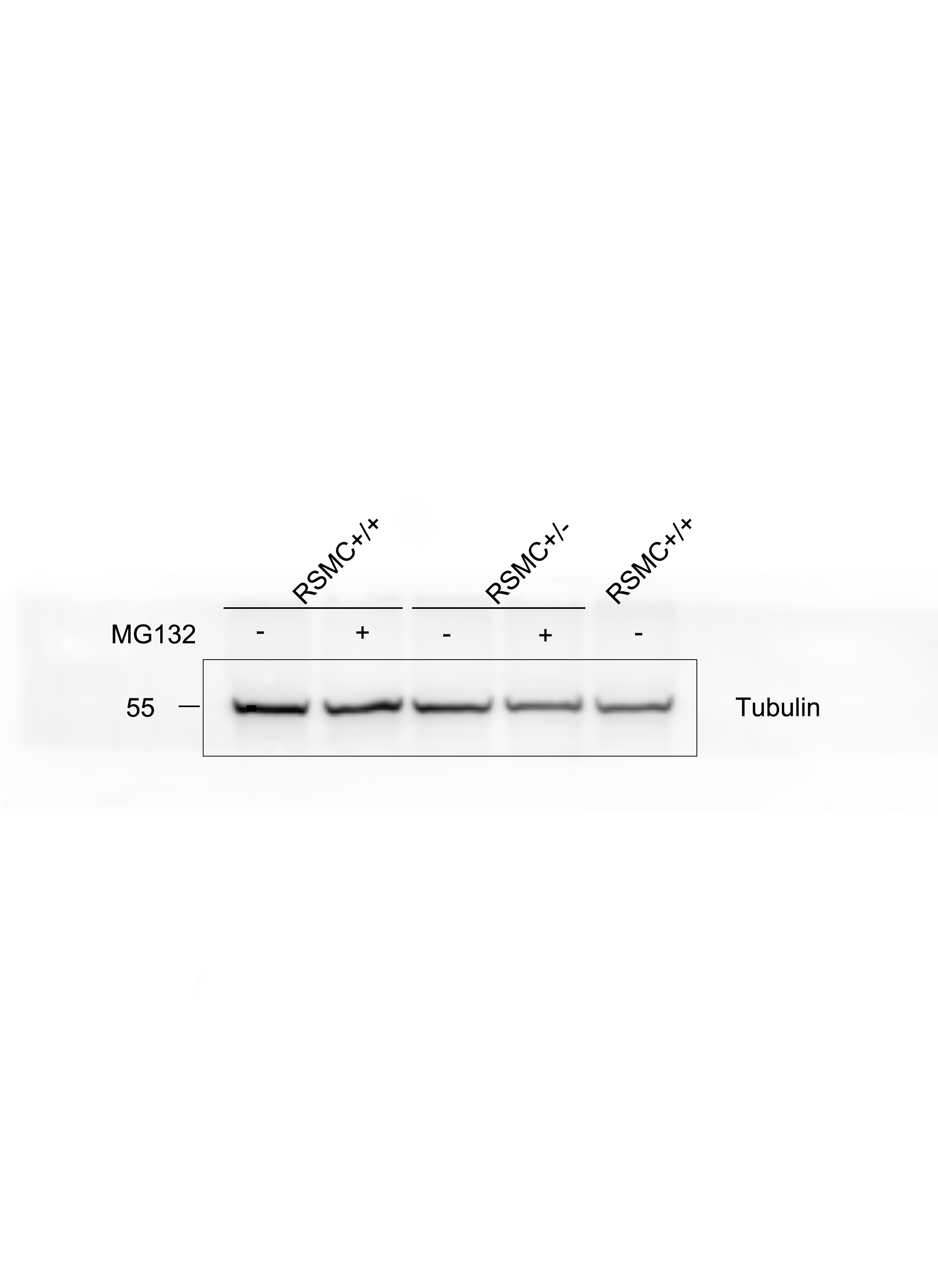

Supplement: Supplementary file 9 — Figure EV1-5 Source Data [file 44318_2025_641_MOESM9_ESM.zip › EMBOJ-2025-120713R_SourceDataForExpandedView/EMBOJ-2025-120713R_SourceDataForFigureEV3/FIG EV3F/tubulin SourceData.tif]

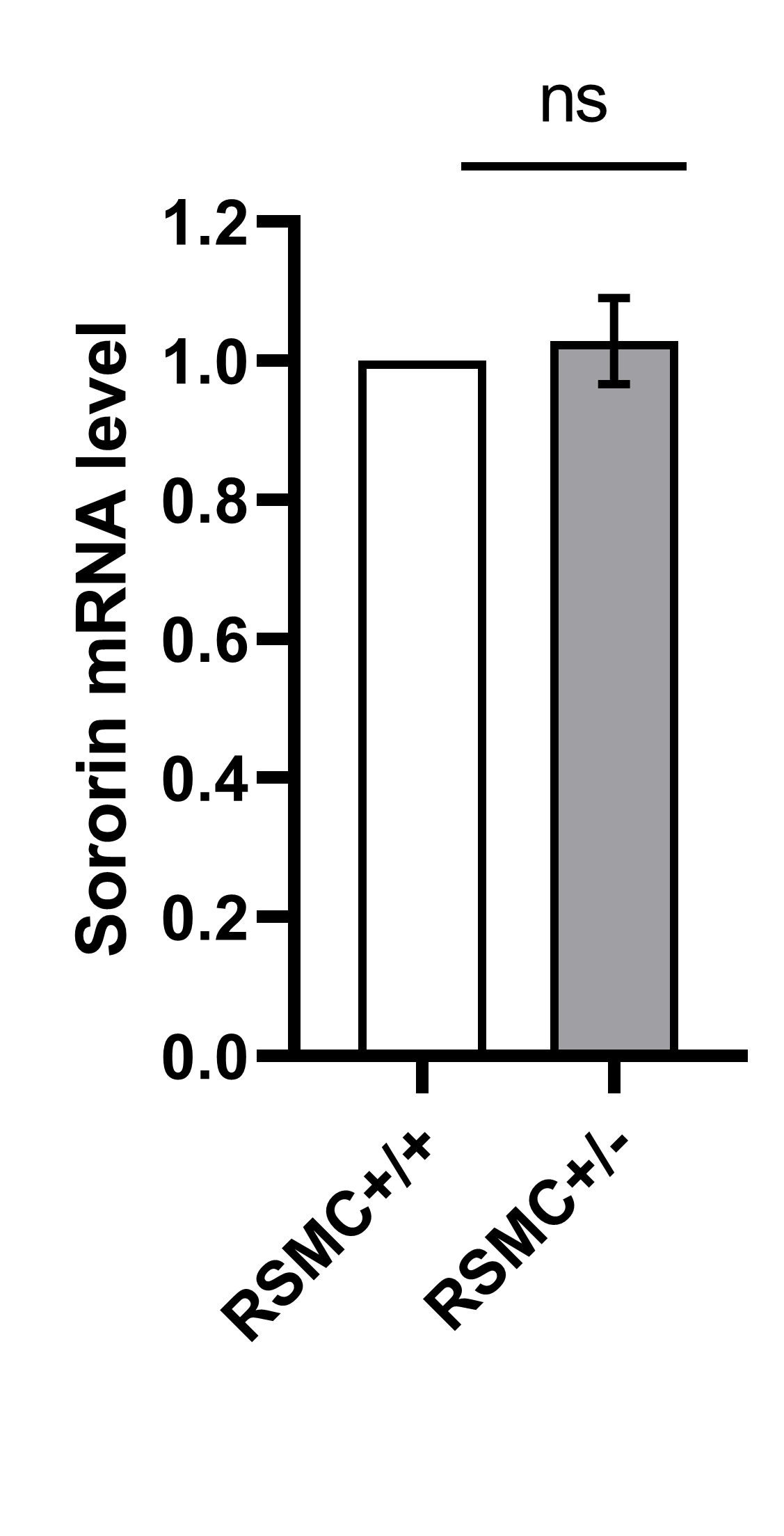

Supplement: Supplementary file 9 — Figure EV1-5 Source Data [file 44318_2025_641_MOESM9_ESM.zip › EMBOJ-2025-120713R_SourceDataForExpandedView/EMBOJ-2025-120713R_SourceDataForFigureEV3/FIG EV3G/FIG EV3G before PS.tif]

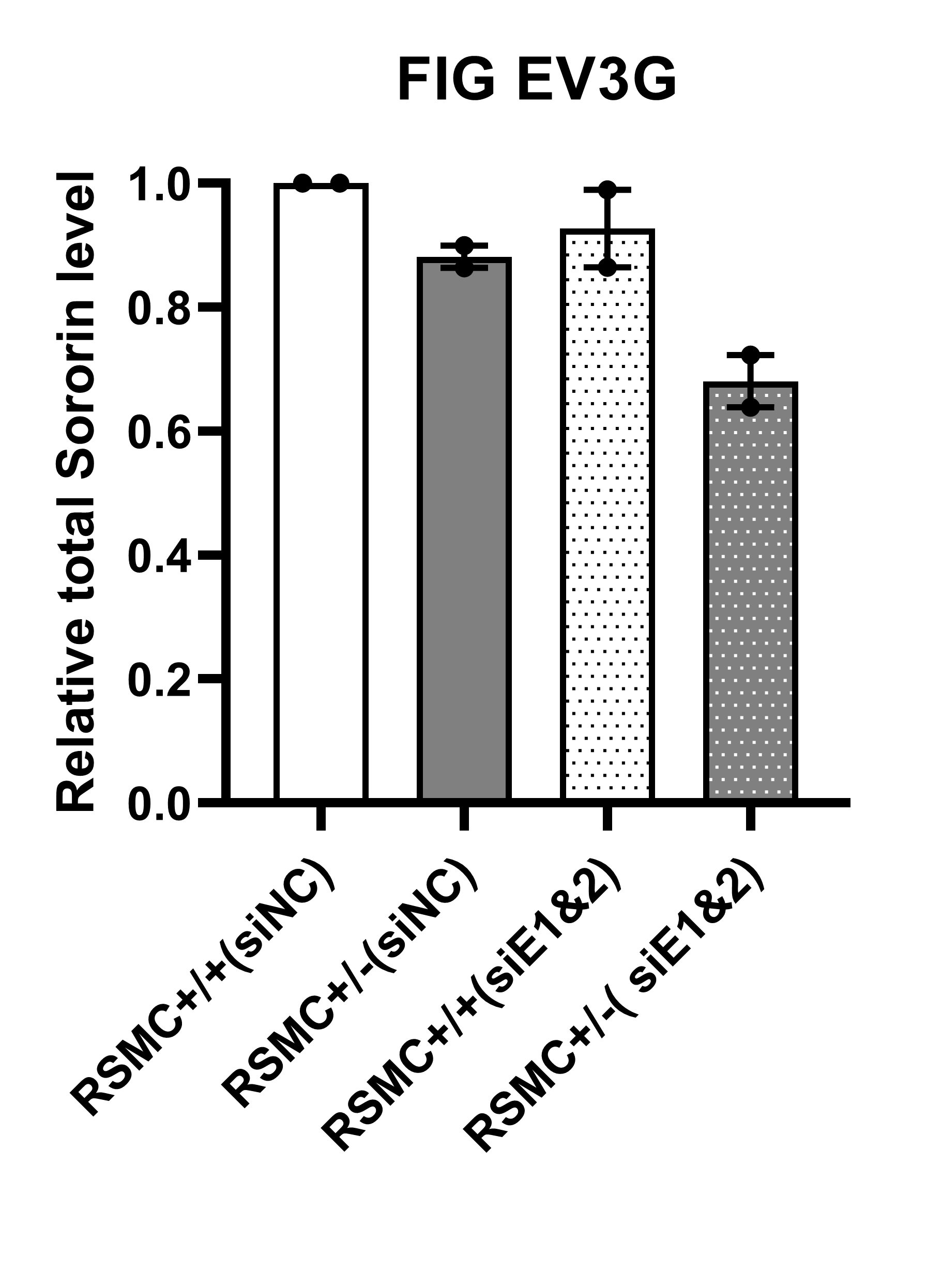

Supplement: Supplementary file 9 — Figure EV1-5 Source Data [file 44318_2025_641_MOESM9_ESM.zip › EMBOJ-2025-120713R_SourceDataForExpandedView/EMBOJ-2025-120713R_SourceDataForFigureEV3/FIG EV3H/FIG EV3H before PS.tif]

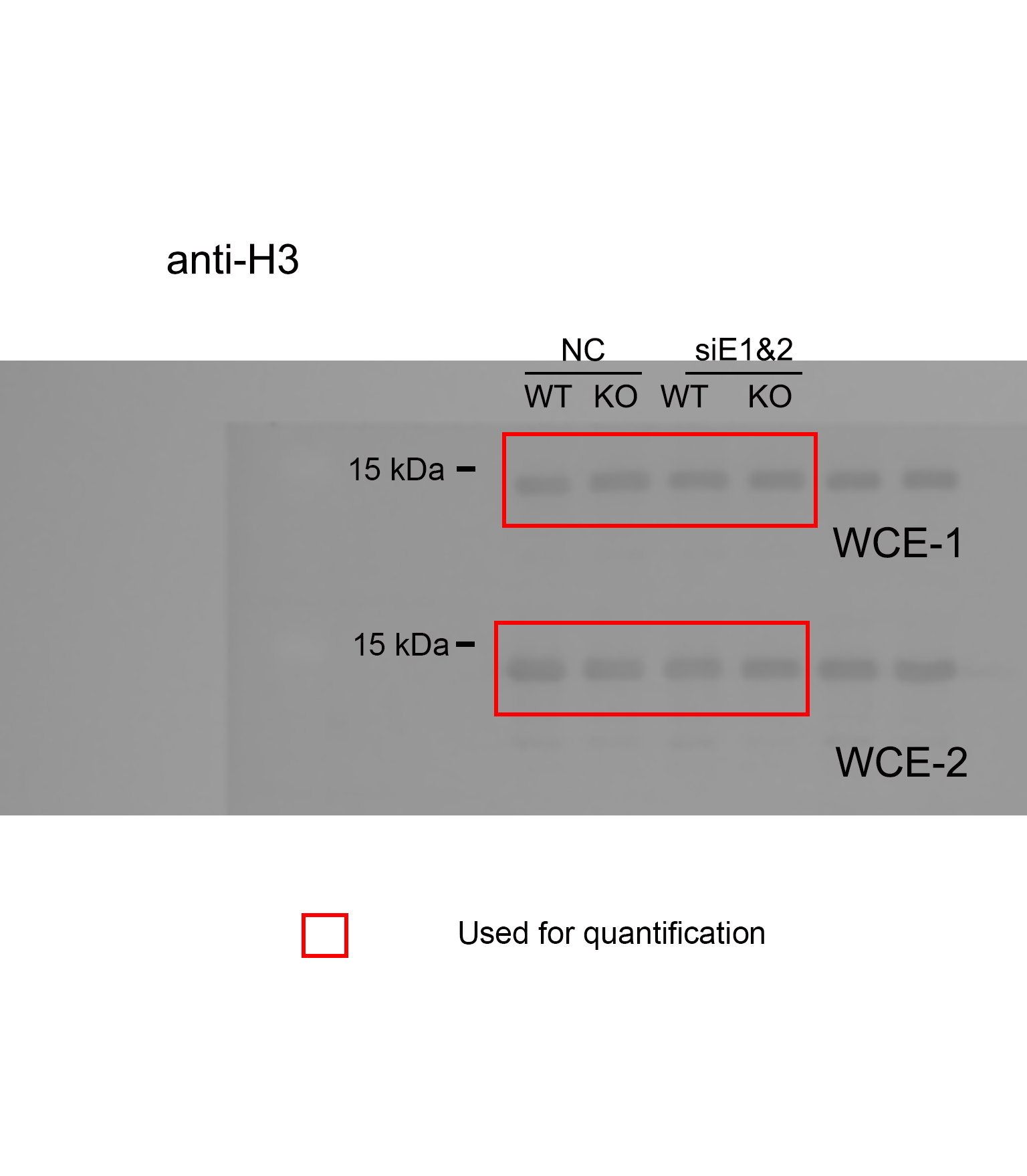

Supplement: Supplementary file 9 — Figure EV1-5 Source Data [file 44318_2025_641_MOESM9_ESM.zip › EMBOJ-2025-120713R_SourceDataForExpandedView/EMBOJ-2025-120713R_SourceDataForFigureEV3/FIG EV3H/H3 RAW data.tif]

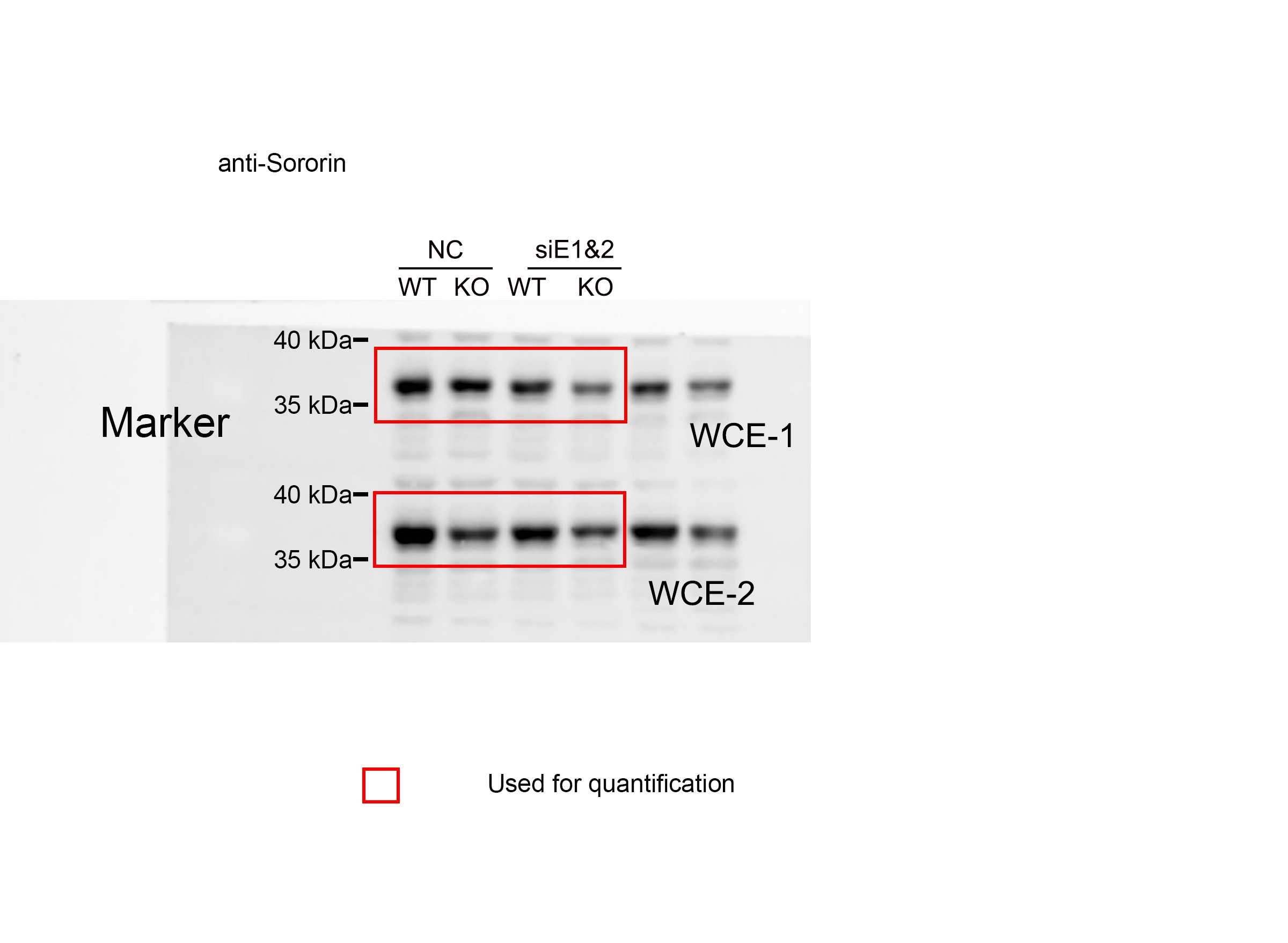

Supplement: Supplementary file 9 — Figure EV1-5 Source Data [file 44318_2025_641_MOESM9_ESM.zip › EMBOJ-2025-120713R_SourceDataForExpandedView/EMBOJ-2025-120713R_SourceDataForFigureEV3/FIG EV3H/Sororin RAW data (with Marker).tif]

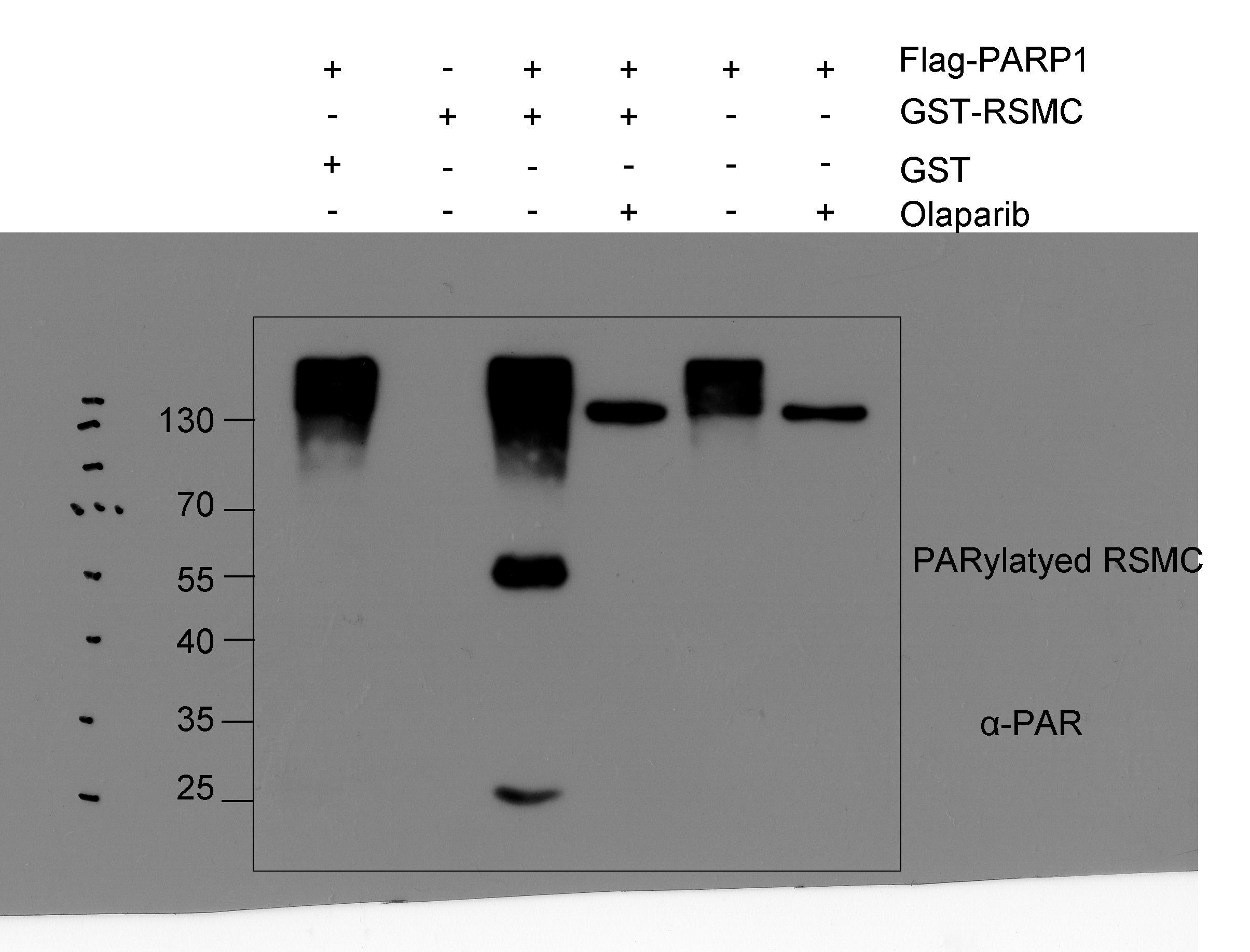

Supplement: Supplementary file 9 — Figure EV1-5 Source Data [file 44318_2025_641_MOESM9_ESM.zip › EMBOJ-2025-120713R_SourceDataForExpandedView/EMBOJ-2025-120713R_SourceDataForFigureEV4/FIG EV4A/PAR-SourceData.tif]

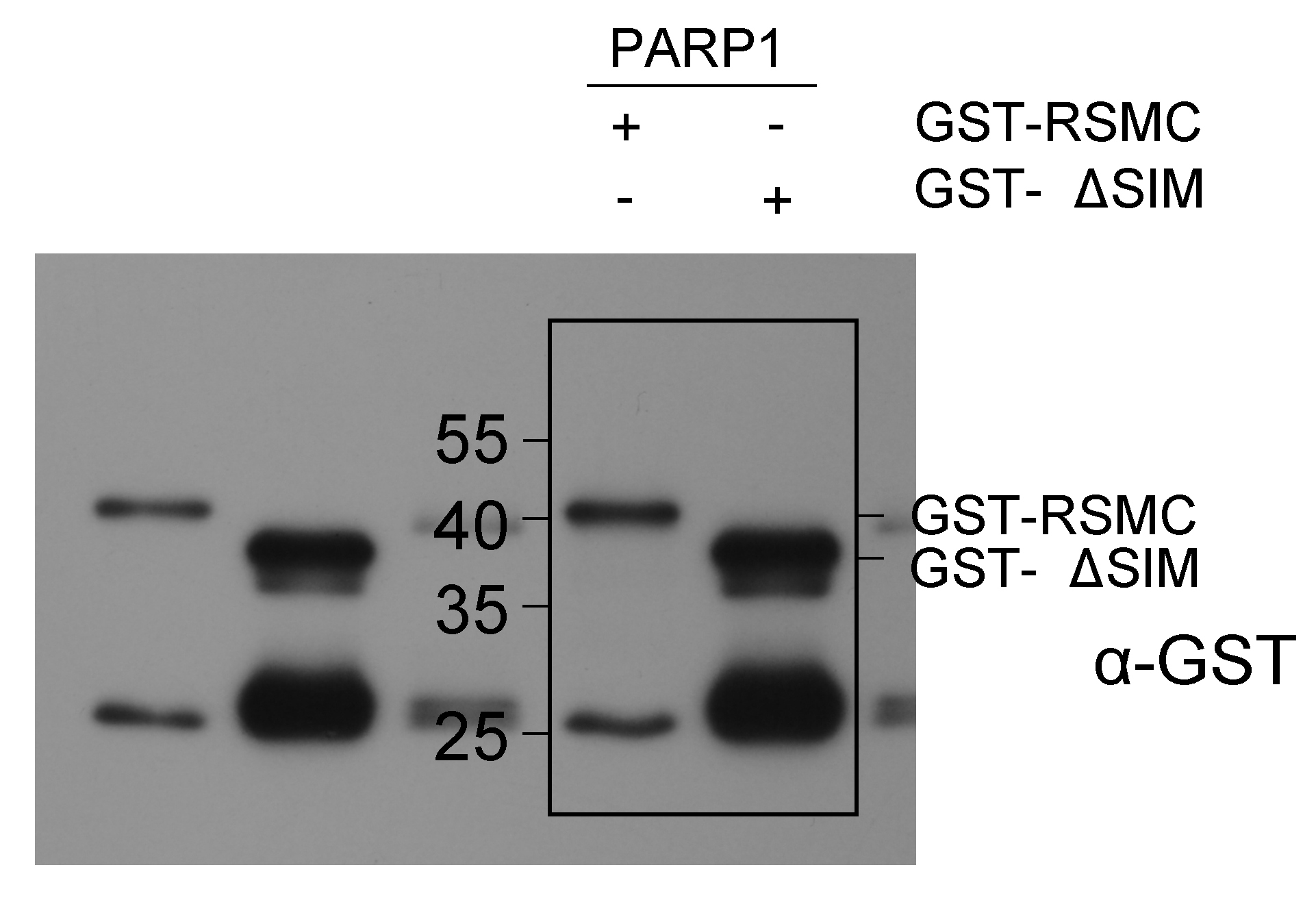

Supplement: Supplementary file 9 — Figure EV1-5 Source Data [file 44318_2025_641_MOESM9_ESM.zip › EMBOJ-2025-120713R_SourceDataForExpandedView/EMBOJ-2025-120713R_SourceDataForFigureEV4/FIG EV4B/α-GST-SourceData.tif]

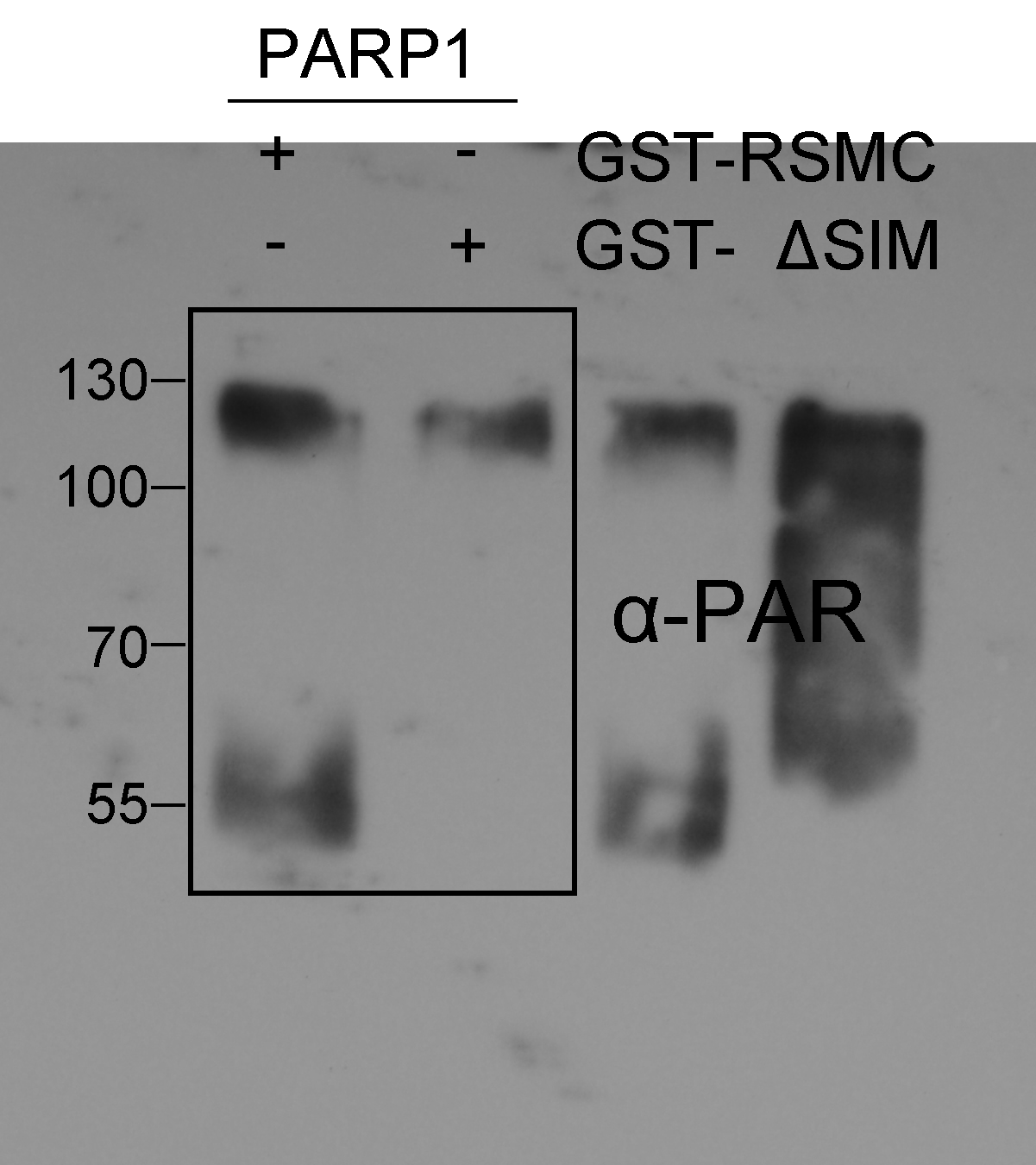

Supplement: Supplementary file 9 — Figure EV1-5 Source Data [file 44318_2025_641_MOESM9_ESM.zip › EMBOJ-2025-120713R_SourceDataForExpandedView/EMBOJ-2025-120713R_SourceDataForFigureEV4/FIG EV4B/α-PAR-SourceData.tif]

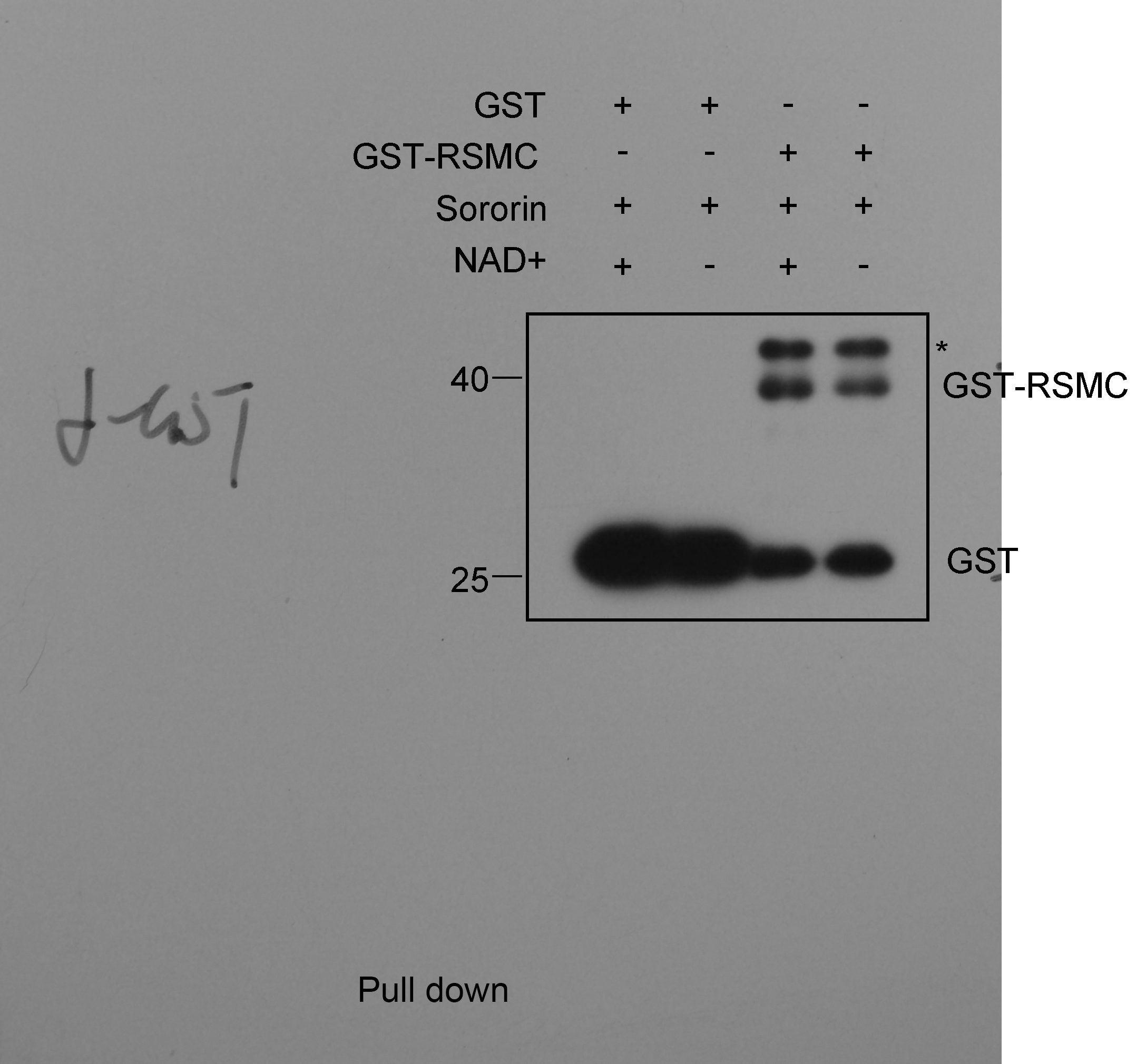

Supplement: Supplementary file 9 — Figure EV1-5 Source Data [file 44318_2025_641_MOESM9_ESM.zip › EMBOJ-2025-120713R_SourceDataForExpandedView/EMBOJ-2025-120713R_SourceDataForFigureEV4/FIG EV4C/anti-GST SourceData.tif]

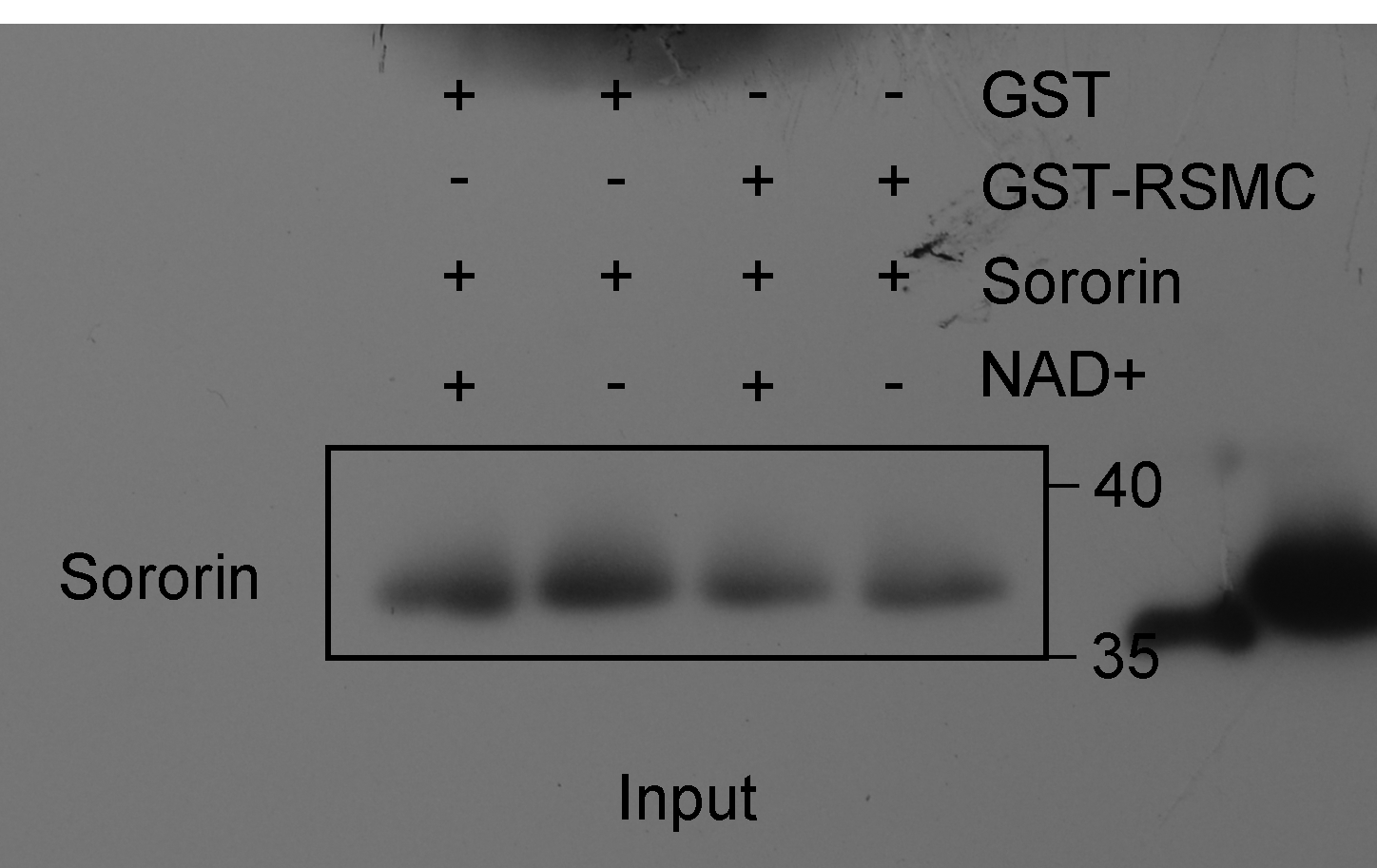

Supplement: Supplementary file 9 — Figure EV1-5 Source Data [file 44318_2025_641_MOESM9_ESM.zip › EMBOJ-2025-120713R_SourceDataForExpandedView/EMBOJ-2025-120713R_SourceDataForFigureEV4/FIG EV4C/sororin input SourceData.tif]

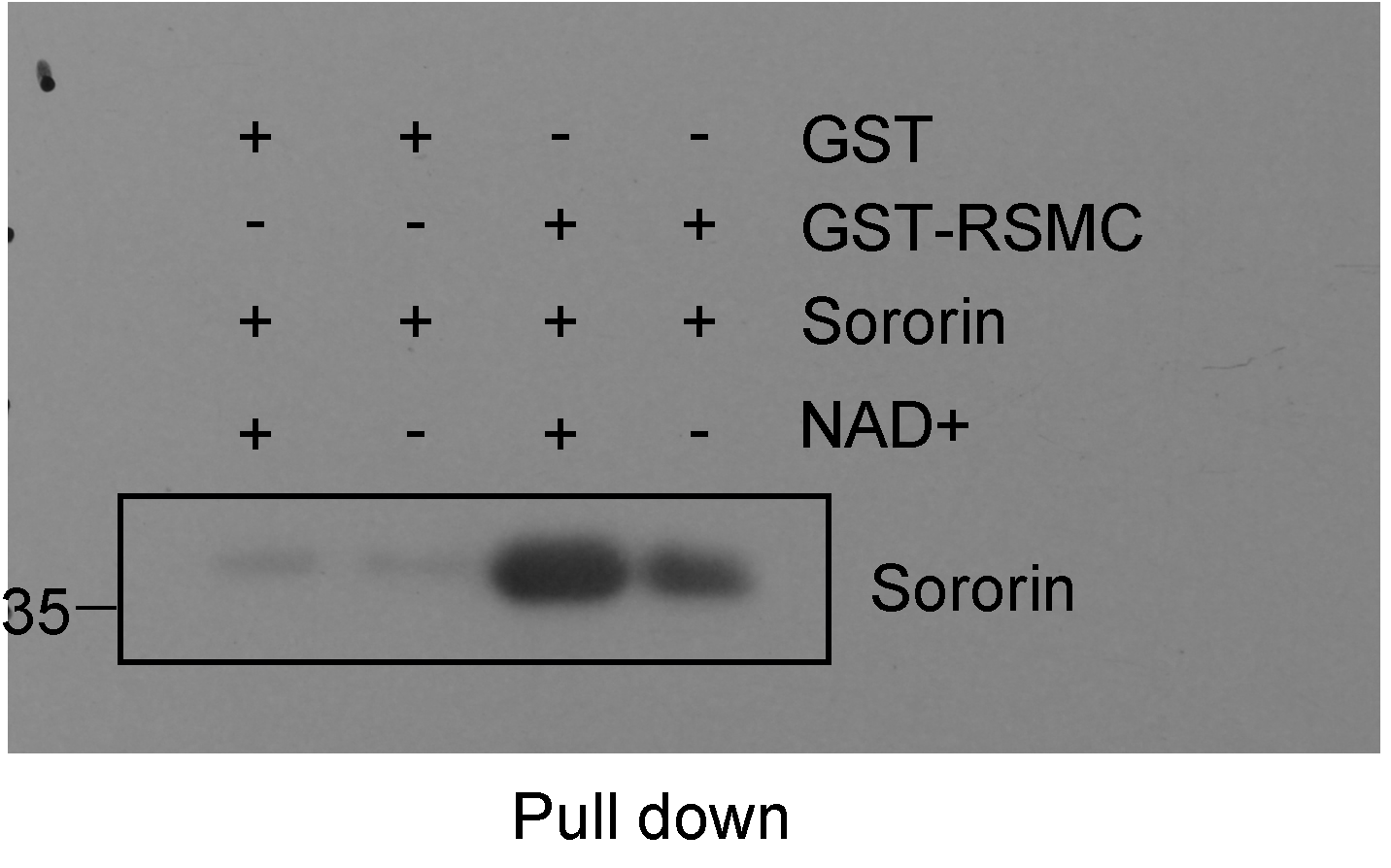

Supplement: Supplementary file 9 — Figure EV1-5 Source Data [file 44318_2025_641_MOESM9_ESM.zip › EMBOJ-2025-120713R_SourceDataForExpandedView/EMBOJ-2025-120713R_SourceDataForFigureEV4/FIG EV4C/Sororin pulldown SourceData.tif]

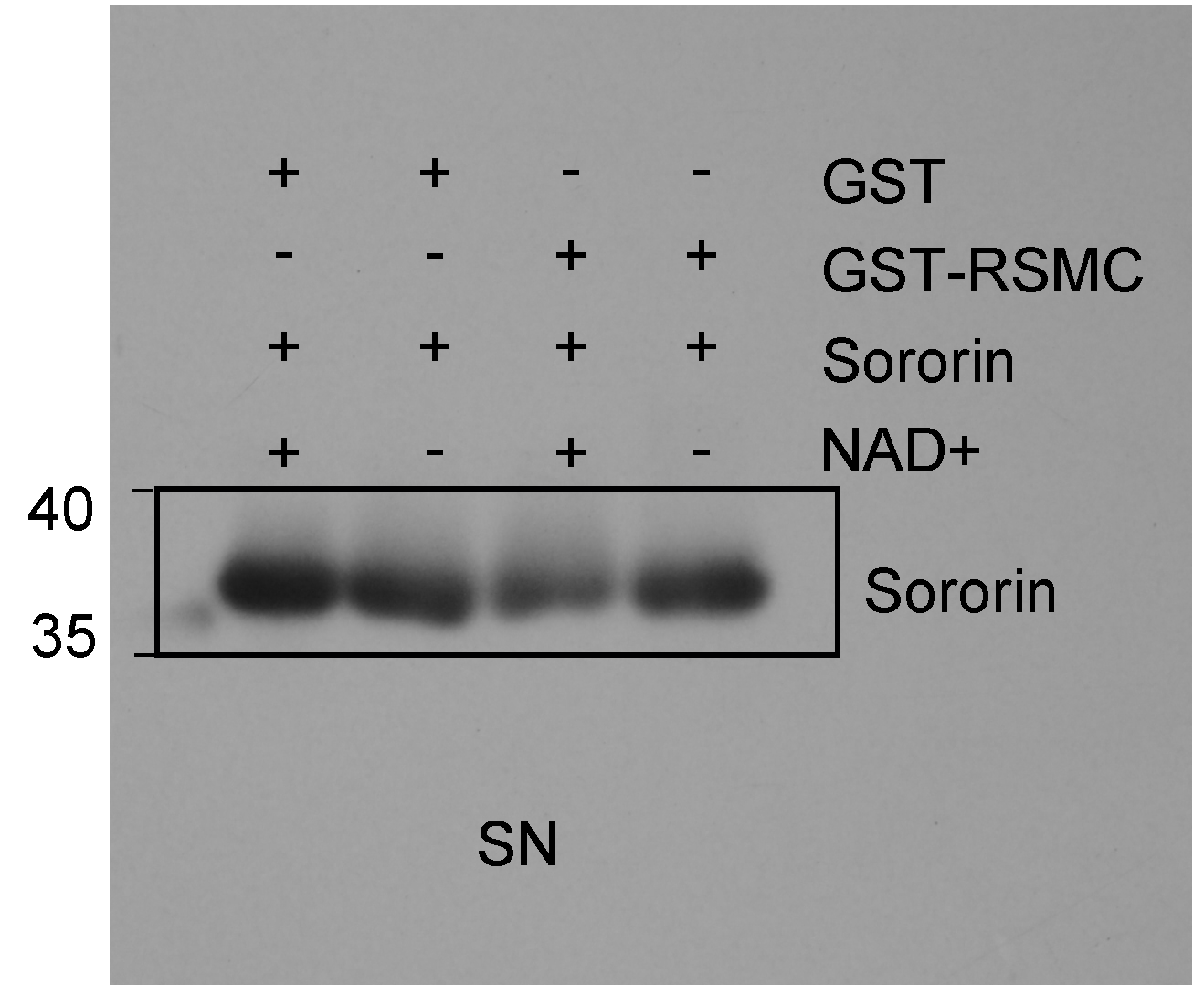

Supplement: Supplementary file 9 — Figure EV1-5 Source Data [file 44318_2025_641_MOESM9_ESM.zip › EMBOJ-2025-120713R_SourceDataForExpandedView/EMBOJ-2025-120713R_SourceDataForFigureEV4/FIG EV4C/sororin SN SourceData.tif]

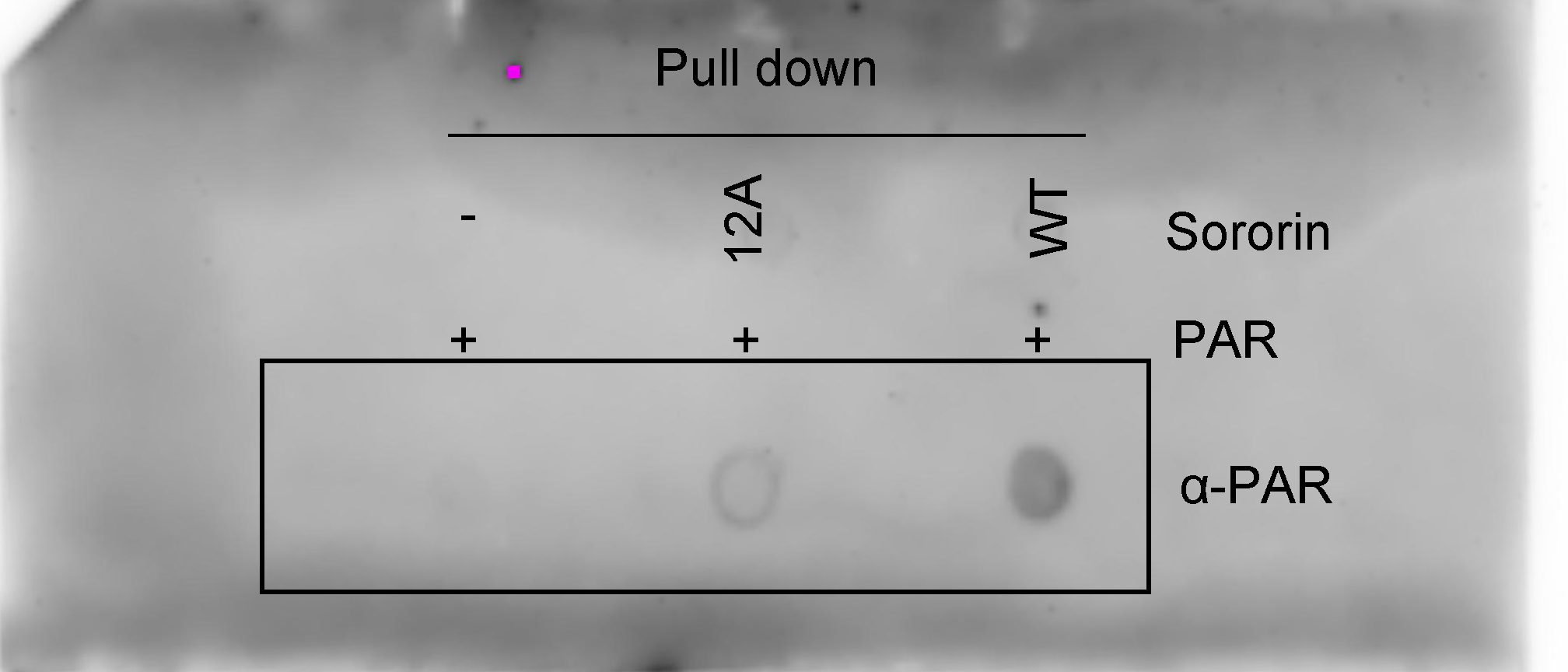

Supplement: Supplementary file 9 — Figure EV1-5 Source Data [file 44318_2025_641_MOESM9_ESM.zip › EMBOJ-2025-120713R_SourceDataForExpandedView/EMBOJ-2025-120713R_SourceDataForFigureEV4/FIG EV4D/PAR SourceData.tif]

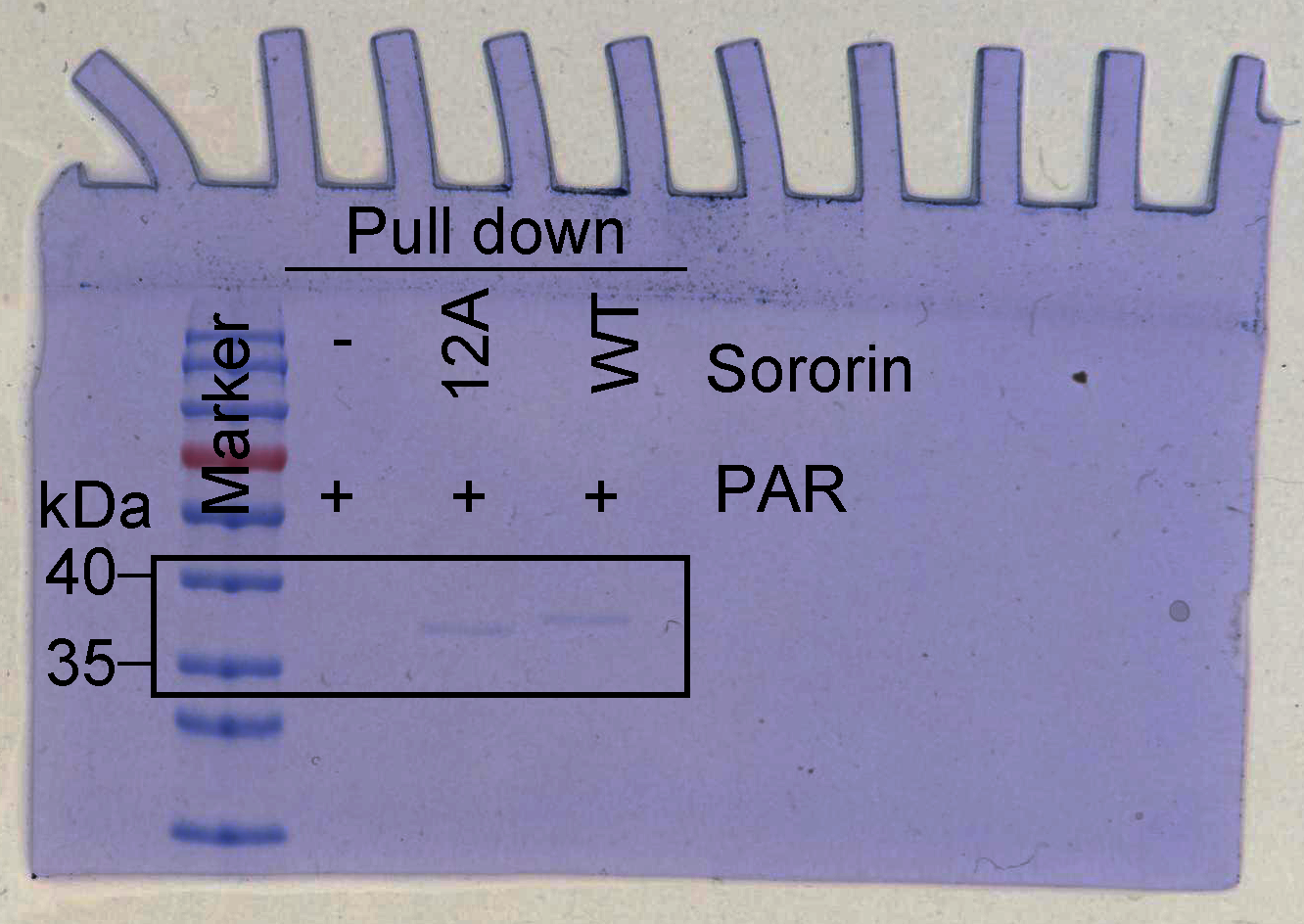

Supplement: Supplementary file 9 — Figure EV1-5 Source Data [file 44318_2025_641_MOESM9_ESM.zip › EMBOJ-2025-120713R_SourceDataForExpandedView/EMBOJ-2025-120713R_SourceDataForFigureEV4/FIG EV4D/Sororin SourceData.tif]

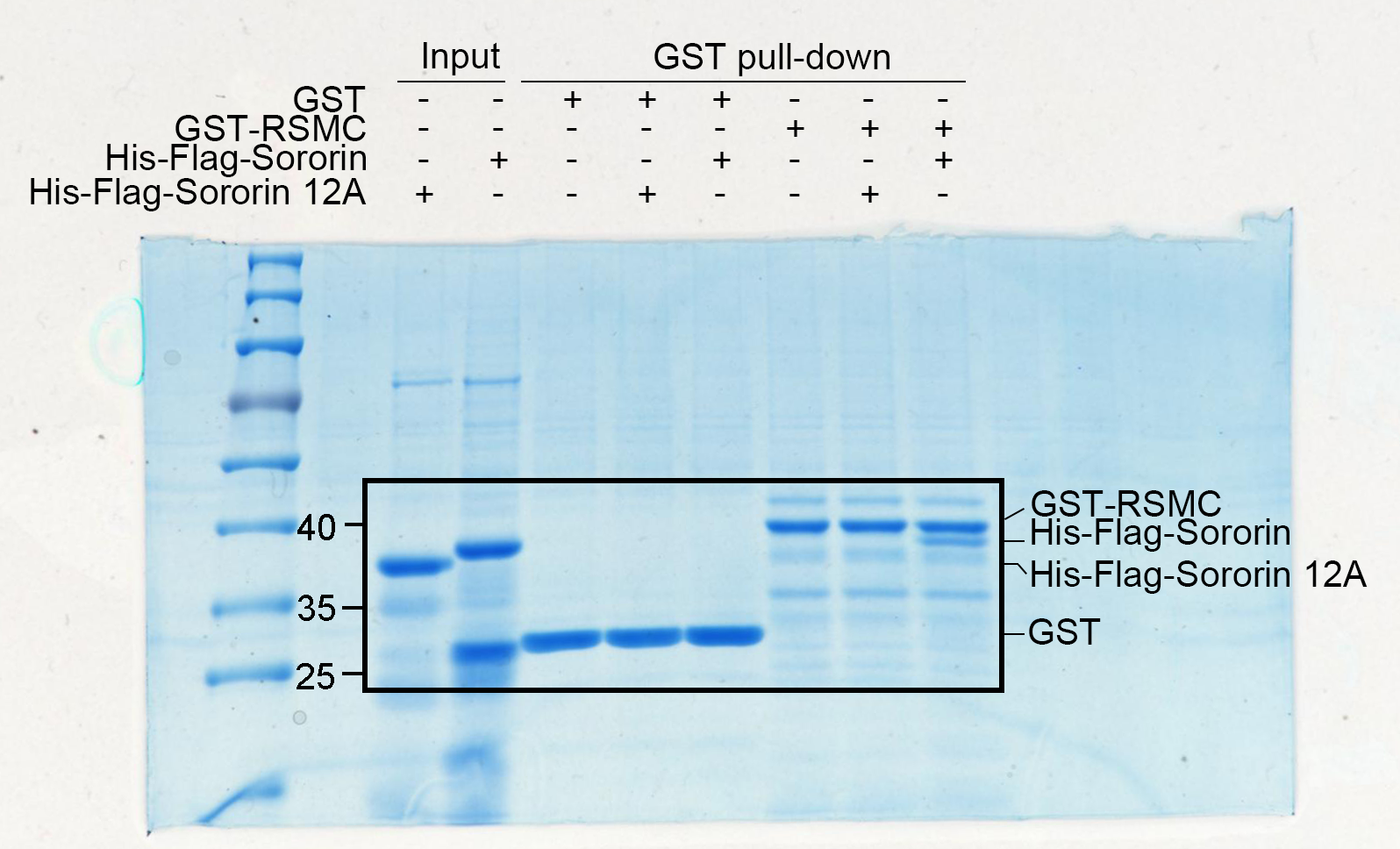

Supplement: Supplementary file 9 — Figure EV1-5 Source Data [file 44318_2025_641_MOESM9_ESM.zip › EMBOJ-2025-120713R_SourceDataForExpandedView/EMBOJ-2025-120713R_SourceDataForFigureEV4/FIG EV4E/SpurceData pull down 12A.tif]

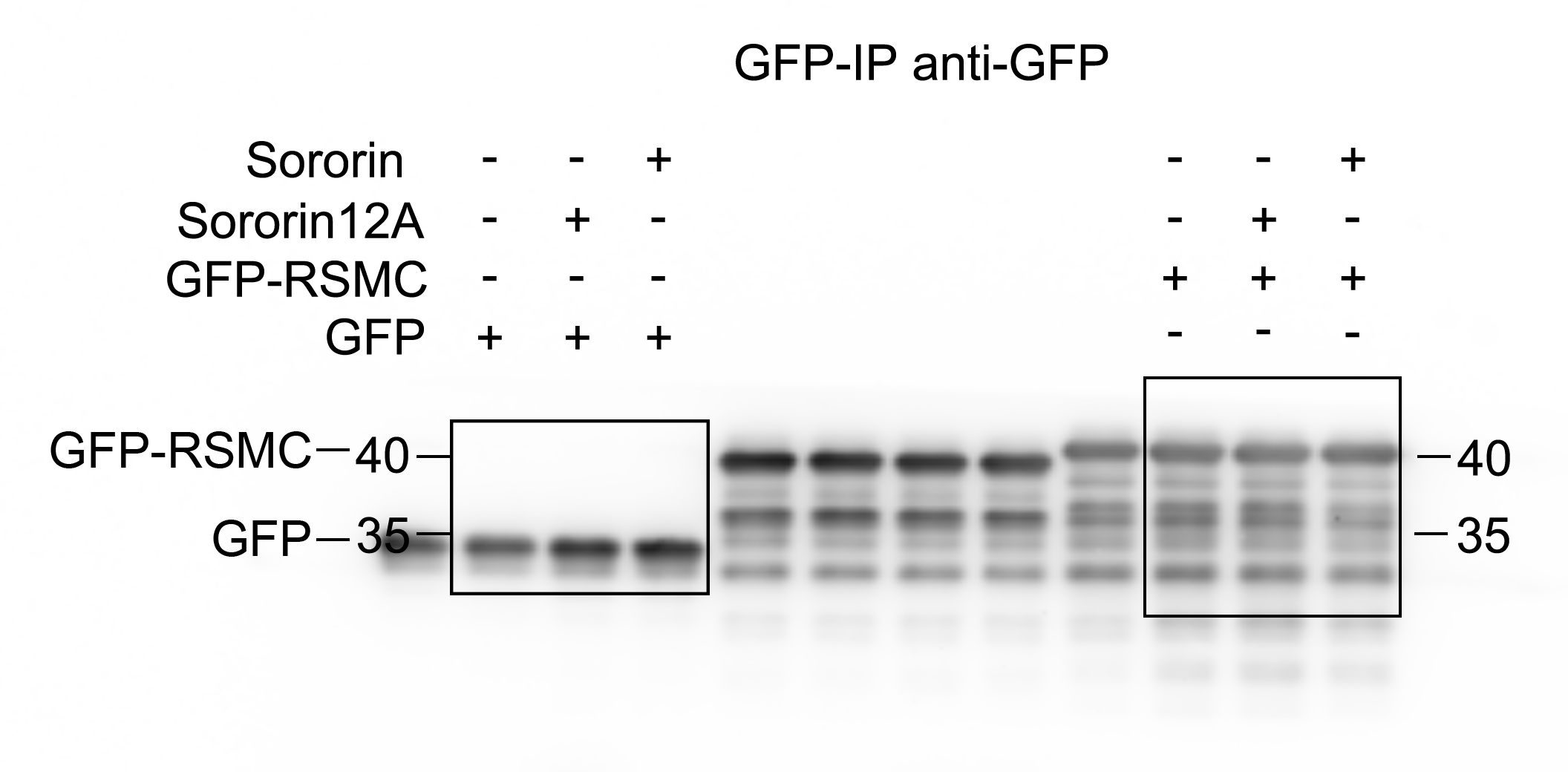

Supplement: Supplementary file 9 — Figure EV1-5 Source Data [file 44318_2025_641_MOESM9_ESM.zip › EMBOJ-2025-120713R_SourceDataForExpandedView/EMBOJ-2025-120713R_SourceDataForFigureEV4/FIG EV4F/GFP-IP anti-GFP.tif]

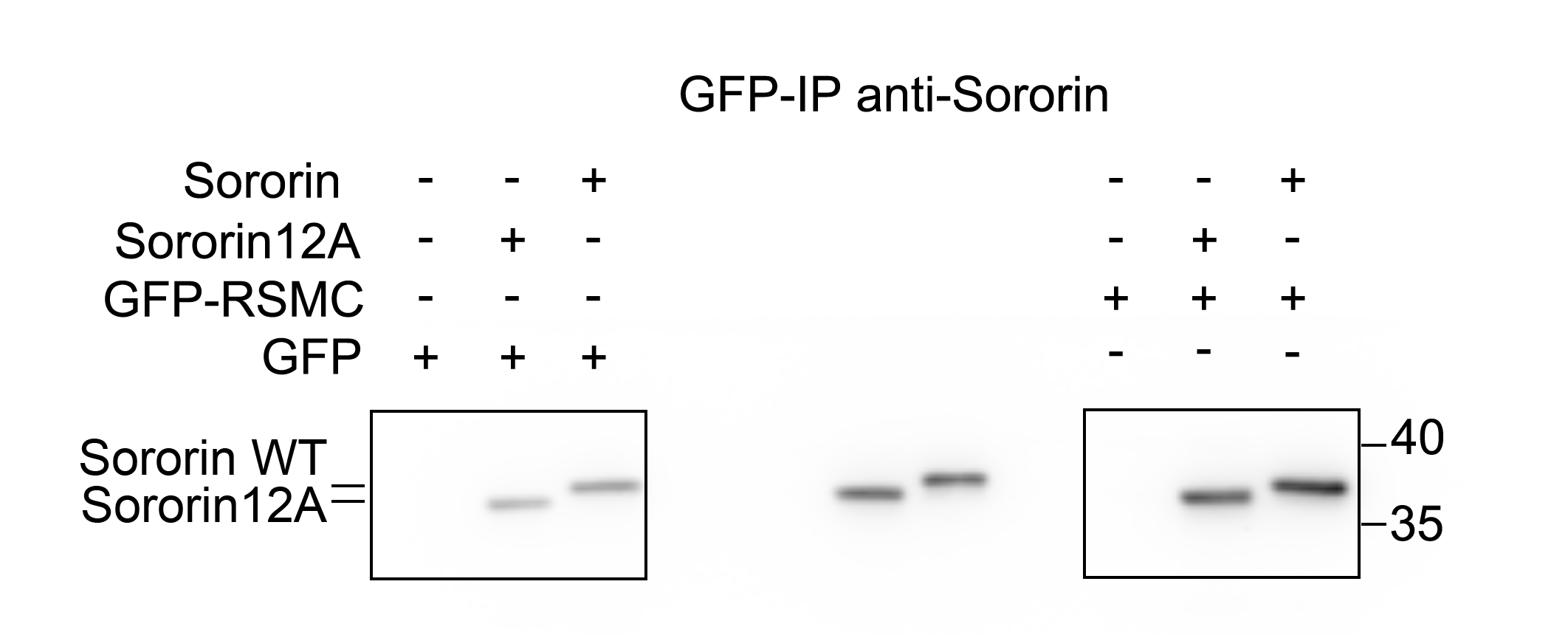

Supplement: Supplementary file 9 — Figure EV1-5 Source Data [file 44318_2025_641_MOESM9_ESM.zip › EMBOJ-2025-120713R_SourceDataForExpandedView/EMBOJ-2025-120713R_SourceDataForFigureEV4/FIG EV4F/GFP-IP anti-Sororin.tif]

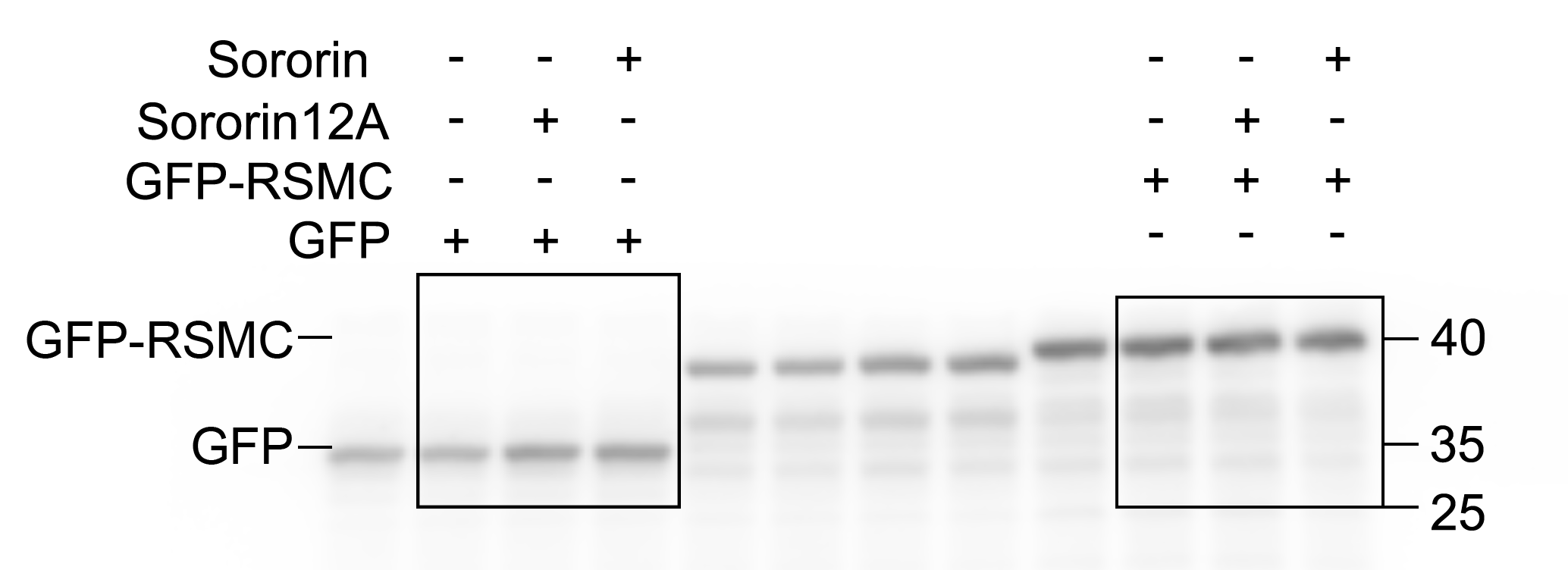

Supplement: Supplementary file 9 — Figure EV1-5 Source Data [file 44318_2025_641_MOESM9_ESM.zip › EMBOJ-2025-120713R_SourceDataForExpandedView/EMBOJ-2025-120713R_SourceDataForFigureEV4/FIG EV4F/input anti-GFP.tif]

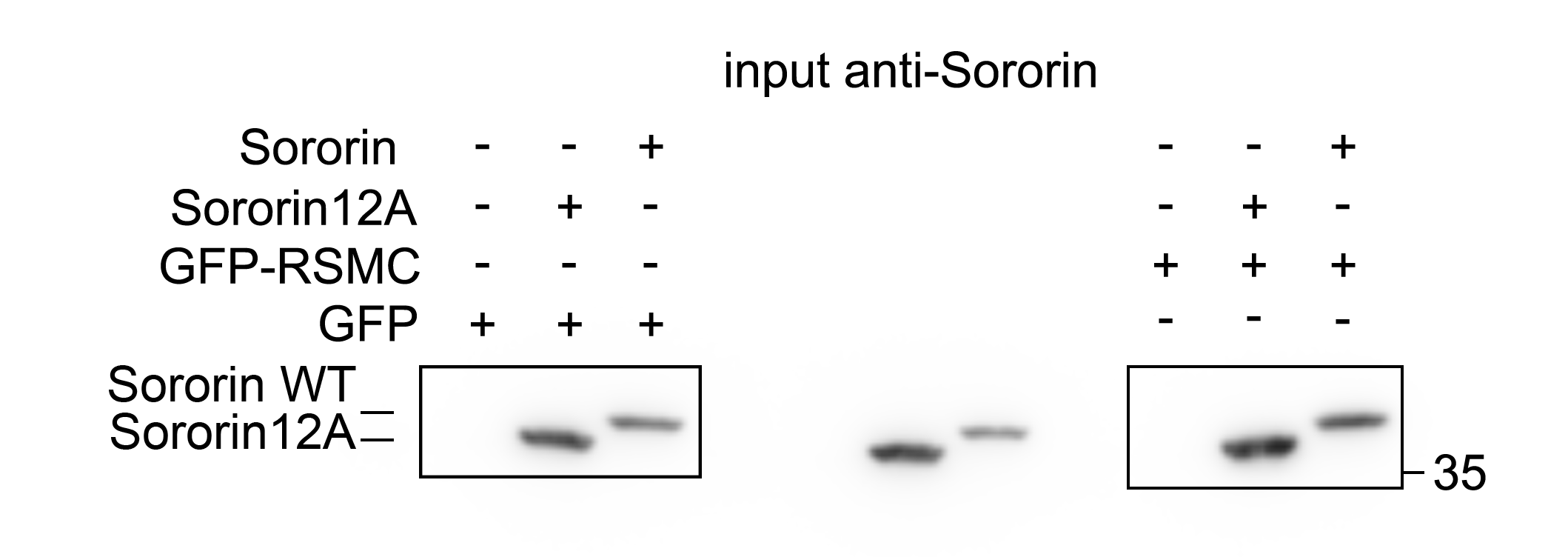

Supplement: Supplementary file 9 — Figure EV1-5 Source Data [file 44318_2025_641_MOESM9_ESM.zip › EMBOJ-2025-120713R_SourceDataForExpandedView/EMBOJ-2025-120713R_SourceDataForFigureEV4/FIG EV4F/input anti-Sororin.tif]

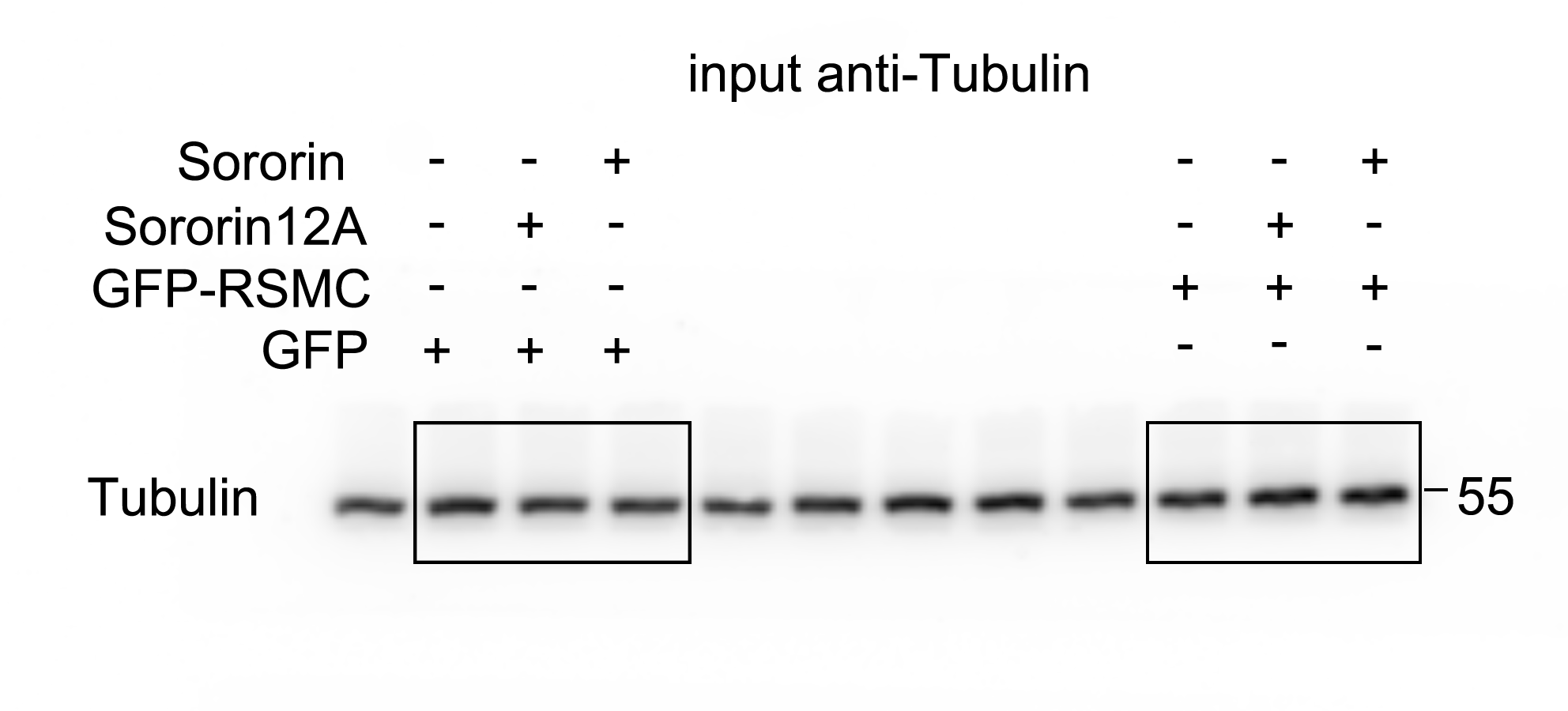

Supplement: Supplementary file 9 — Figure EV1-5 Source Data [file 44318_2025_641_MOESM9_ESM.zip › EMBOJ-2025-120713R_SourceDataForExpandedView/EMBOJ-2025-120713R_SourceDataForFigureEV4/FIG EV4F/input anti-Tubulin.tif]

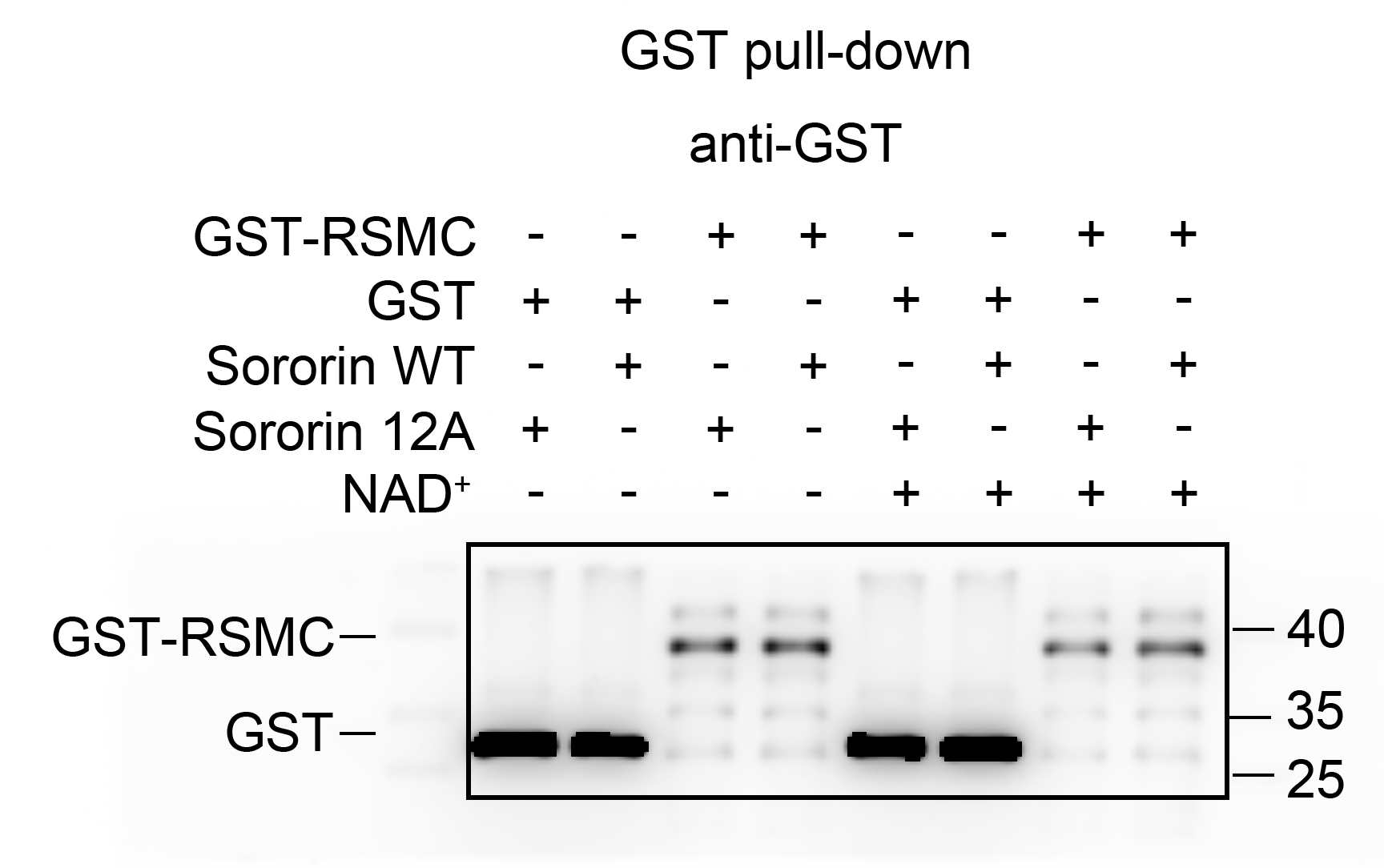

Supplement: Supplementary file 9 — Figure EV1-5 Source Data [file 44318_2025_641_MOESM9_ESM.zip › EMBOJ-2025-120713R_SourceDataForExpandedView/EMBOJ-2025-120713R_SourceDataForFigureEV4/FIG EV4G/raw data- GST pull-down anti-GST.tif]

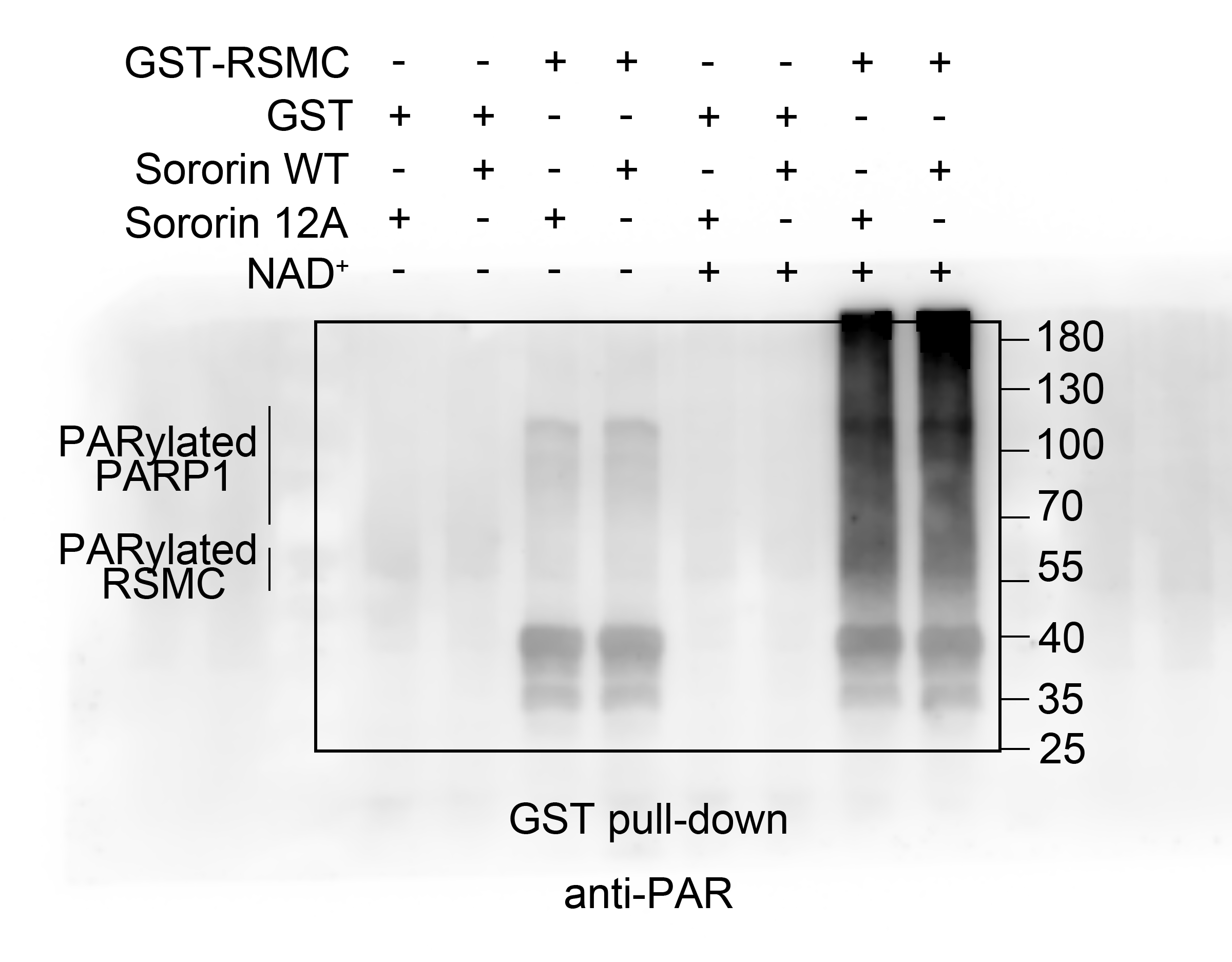

Supplement: Supplementary file 9 — Figure EV1-5 Source Data [file 44318_2025_641_MOESM9_ESM.zip › EMBOJ-2025-120713R_SourceDataForExpandedView/EMBOJ-2025-120713R_SourceDataForFigureEV4/FIG EV4G/raw data- GST pull-down anti-PAR.tif]

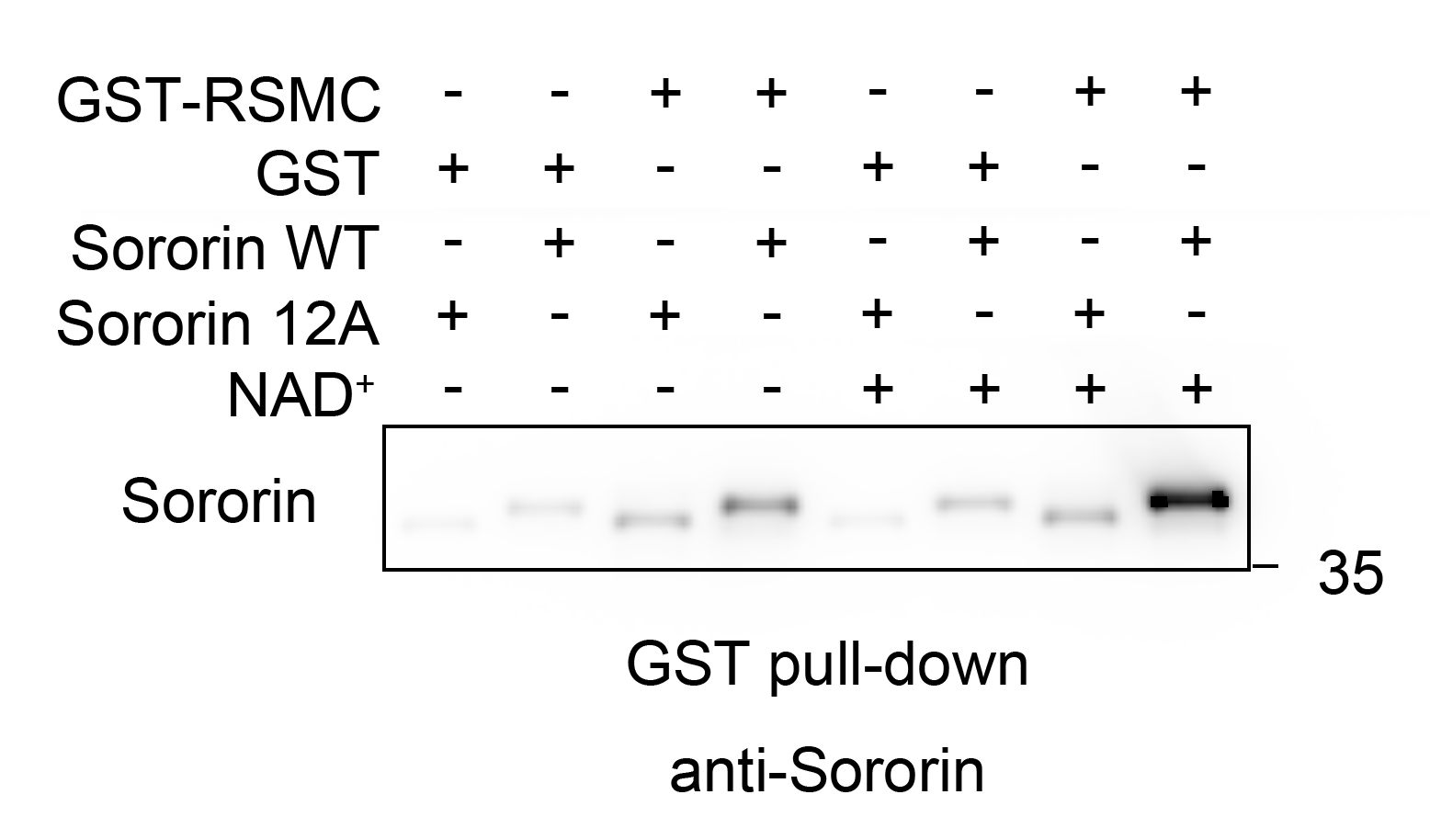

Supplement: Supplementary file 9 — Figure EV1-5 Source Data [file 44318_2025_641_MOESM9_ESM.zip › EMBOJ-2025-120713R_SourceDataForExpandedView/EMBOJ-2025-120713R_SourceDataForFigureEV4/FIG EV4G/raw data- GST pull-down anti-Soroin.tif]

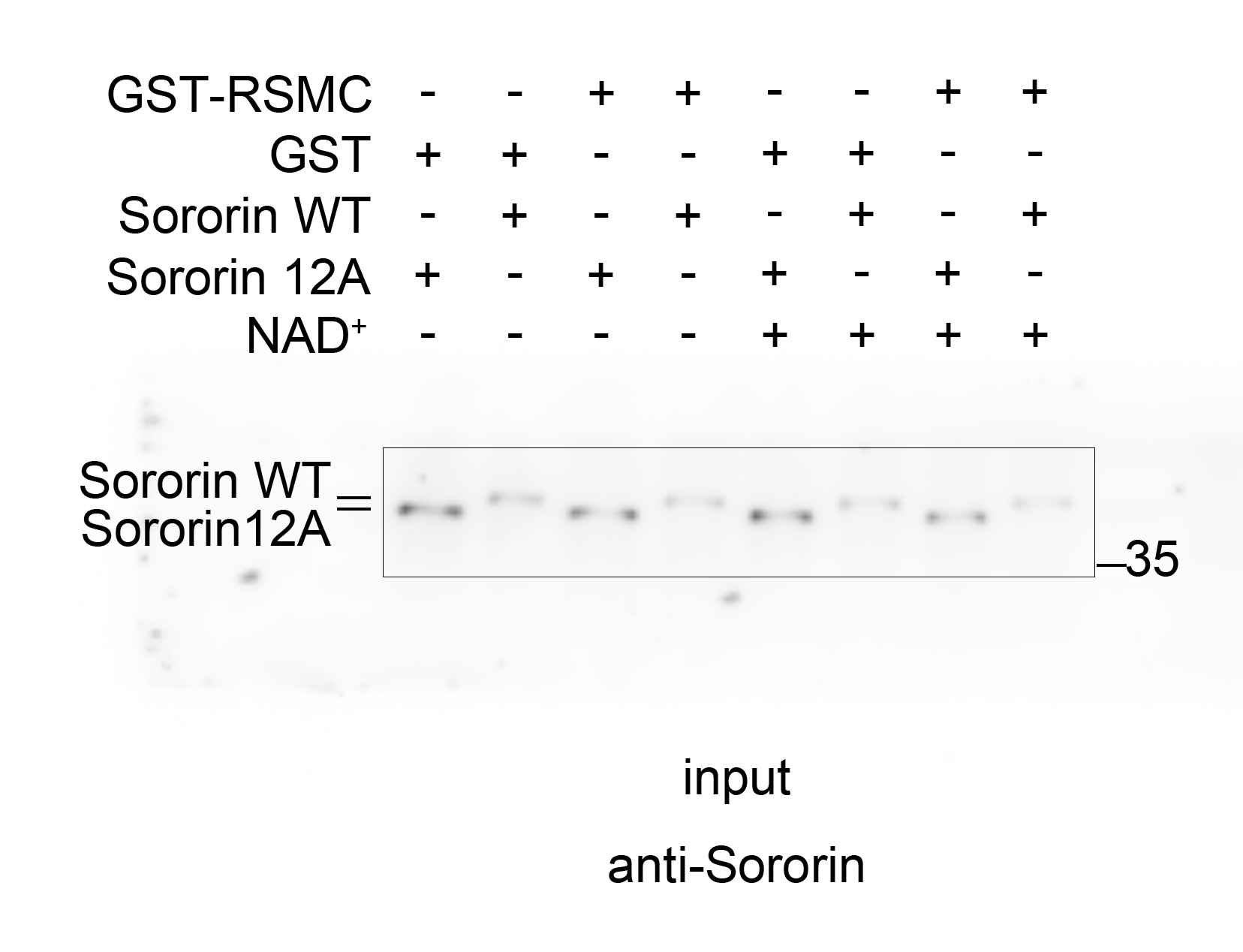

Supplement: Supplementary file 9 — Figure EV1-5 Source Data [file 44318_2025_641_MOESM9_ESM.zip › EMBOJ-2025-120713R_SourceDataForExpandedView/EMBOJ-2025-120713R_SourceDataForFigureEV4/FIG EV4G/raw data- input anti-Sororin.tif]

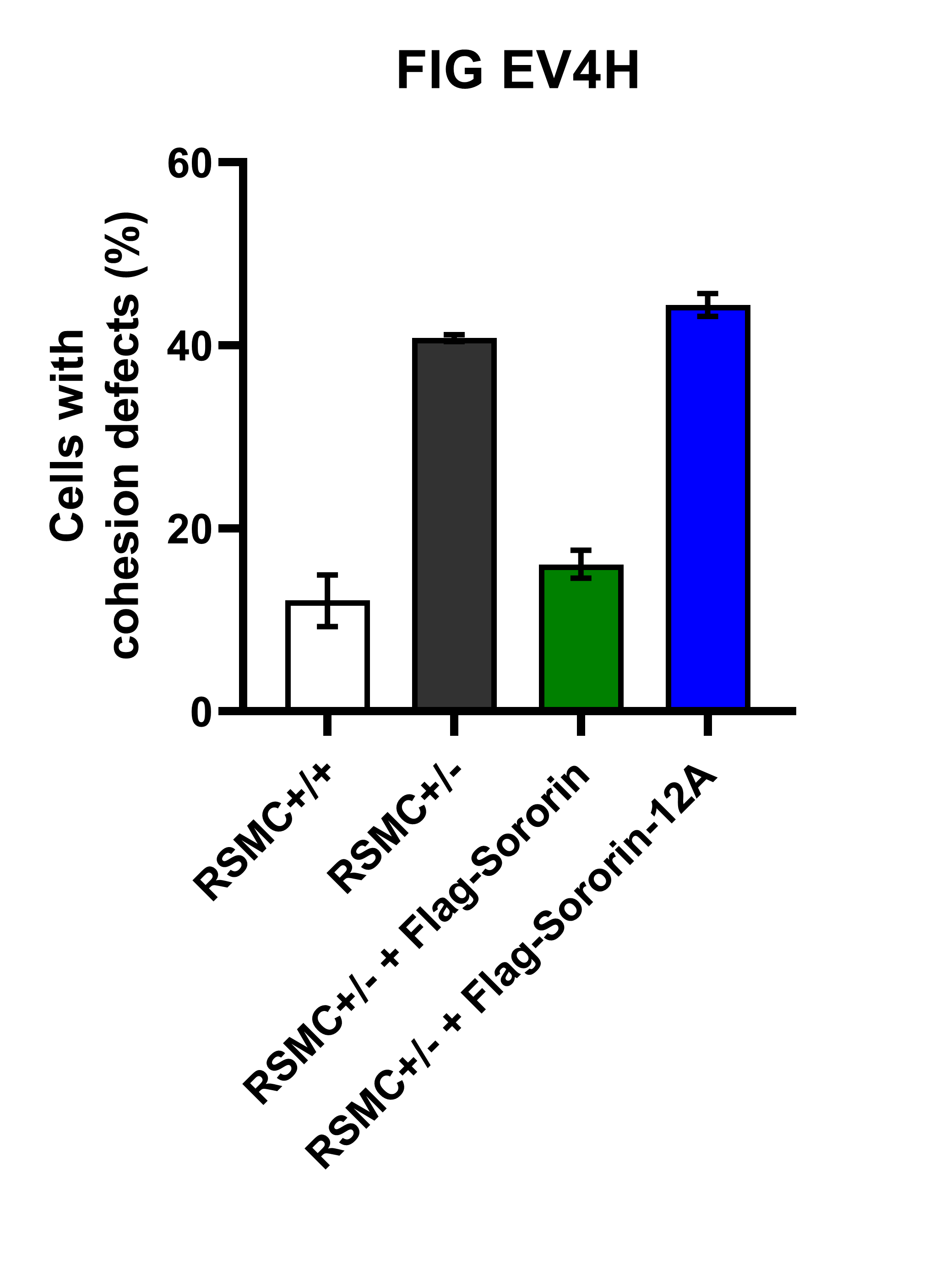

Supplement: Supplementary file 9 — Figure EV1-5 Source Data [file 44318_2025_641_MOESM9_ESM.zip › EMBOJ-2025-120713R_SourceDataForExpandedView/EMBOJ-2025-120713R_SourceDataForFigureEV4/FIG EV4H/FIG EV4H beforePS.tif]

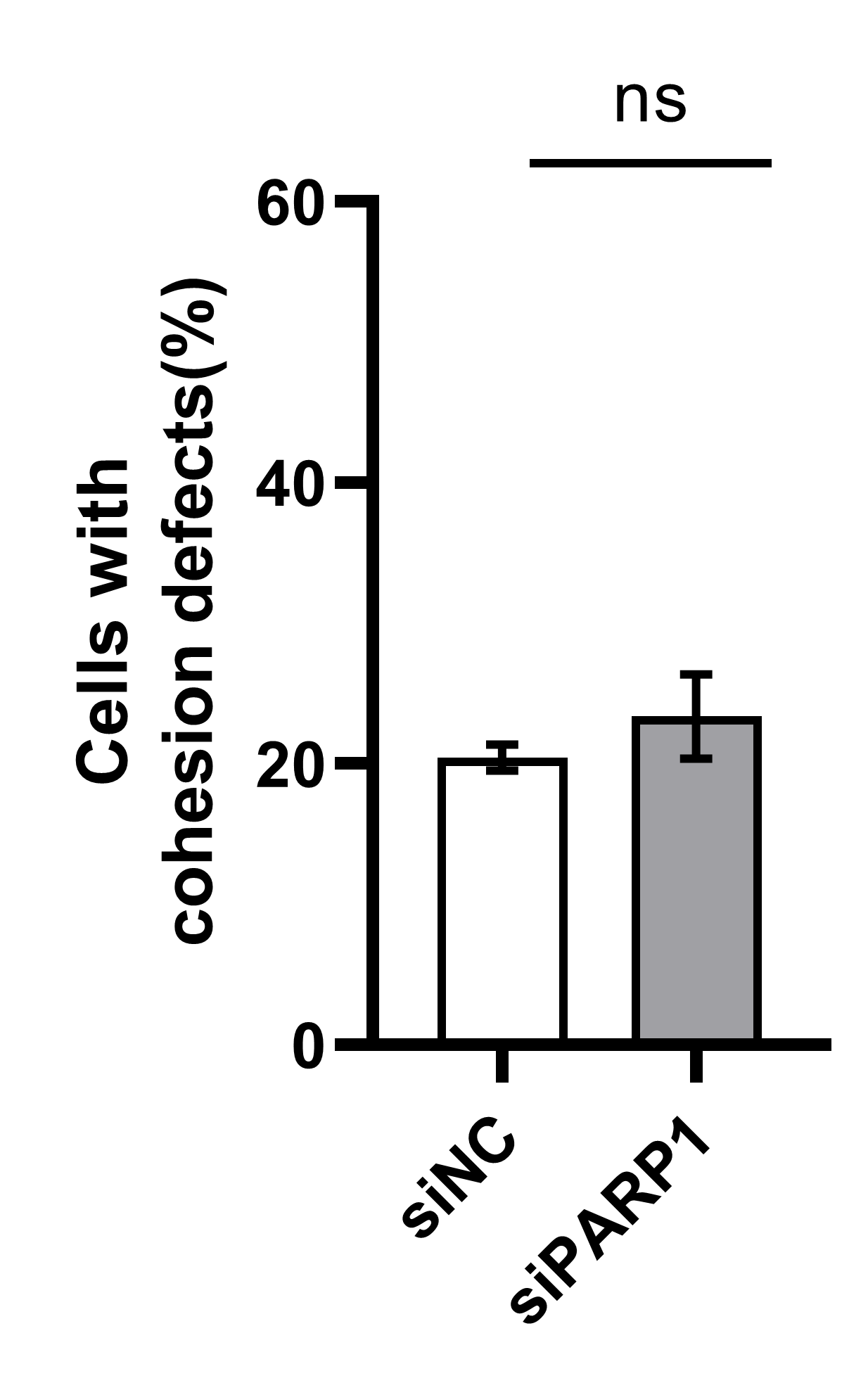

Supplement: Supplementary file 9 — Figure EV1-5 Source Data [file 44318_2025_641_MOESM9_ESM.zip › EMBOJ-2025-120713R_SourceDataForExpandedView/EMBOJ-2025-120713R_SourceDataForFigureEV5/FIG EV5A/FIG EV5A before PS.tif]

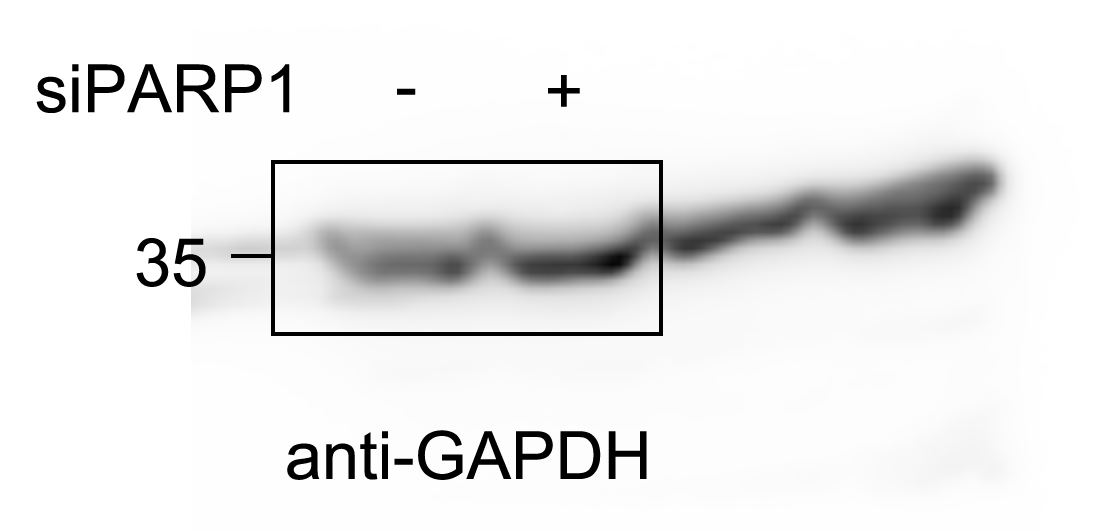

Supplement: Supplementary file 9 — Figure EV1-5 Source Data [file 44318_2025_641_MOESM9_ESM.zip › EMBOJ-2025-120713R_SourceDataForExpandedView/EMBOJ-2025-120713R_SourceDataForFigureEV5/FIG EV5A/siPARP1 WB/anti-GAPDH RawData.tif]

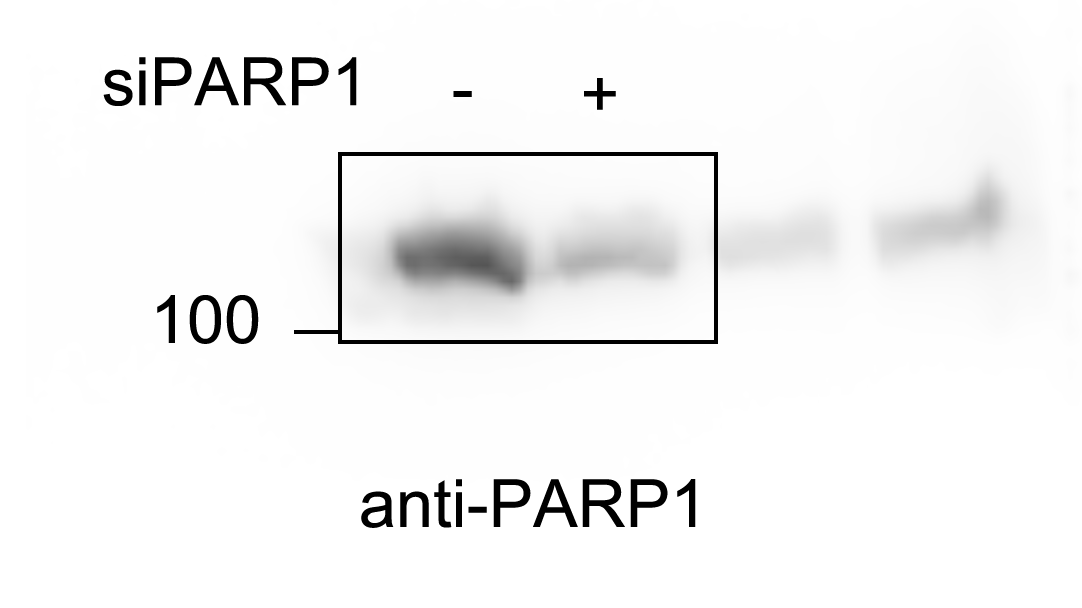

Supplement: Supplementary file 9 — Figure EV1-5 Source Data [file 44318_2025_641_MOESM9_ESM.zip › EMBOJ-2025-120713R_SourceDataForExpandedView/EMBOJ-2025-120713R_SourceDataForFigureEV5/FIG EV5A/siPARP1 WB/anti-PARP1 RawData.tif]

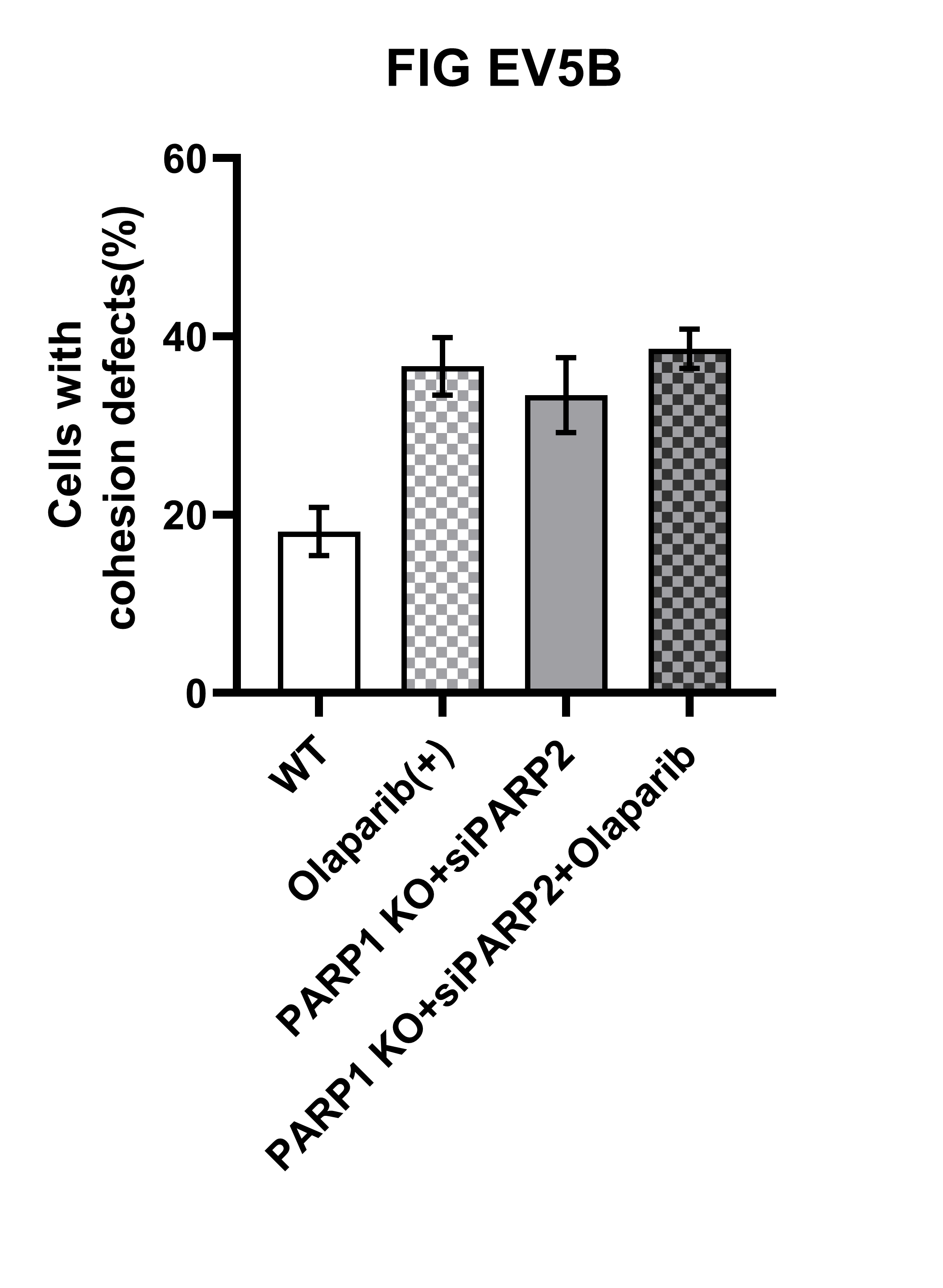

Supplement: Supplementary file 9 — Figure EV1-5 Source Data [file 44318_2025_641_MOESM9_ESM.zip › EMBOJ-2025-120713R_SourceDataForExpandedView/EMBOJ-2025-120713R_SourceDataForFigureEV5/FIG EV5B/FIG EV5B before PS.tif]

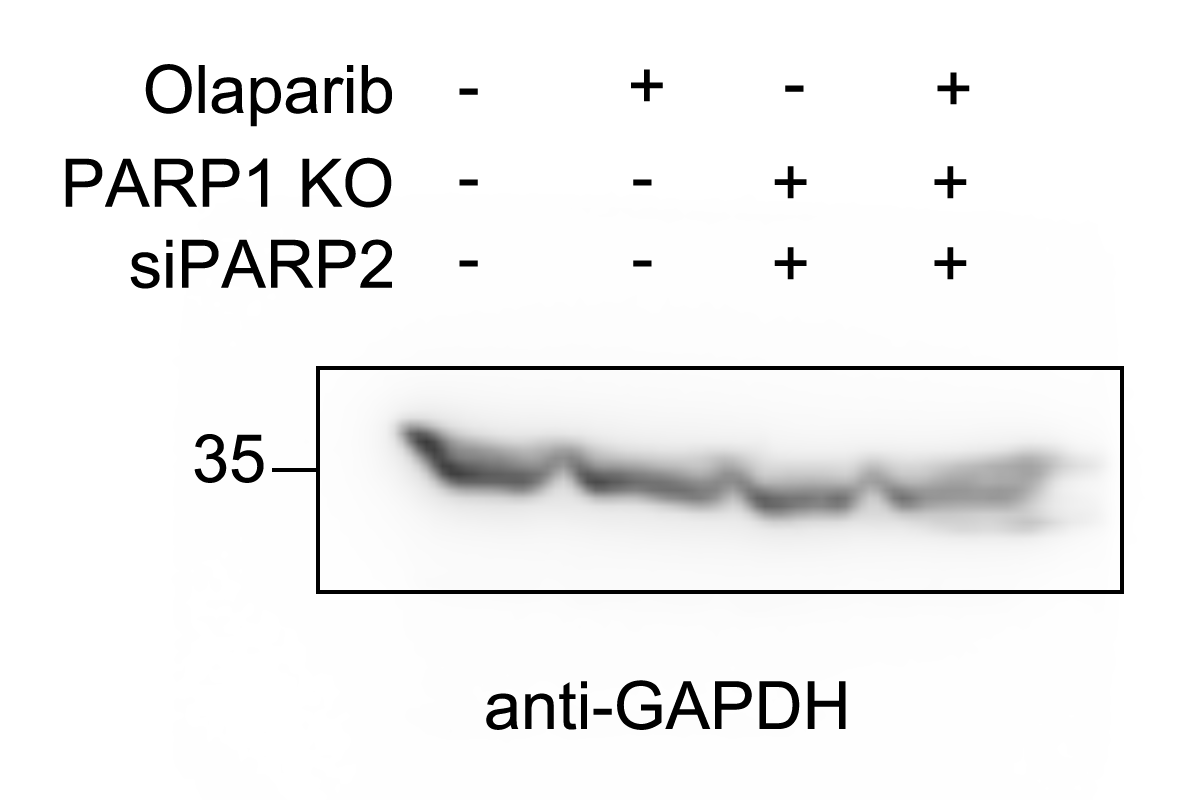

Supplement: Supplementary file 9 — Figure EV1-5 Source Data [file 44318_2025_641_MOESM9_ESM.zip › EMBOJ-2025-120713R_SourceDataForExpandedView/EMBOJ-2025-120713R_SourceDataForFigureEV5/FIG EV5B/siPARP2 WB/anti-GAPDH RawData.tif]

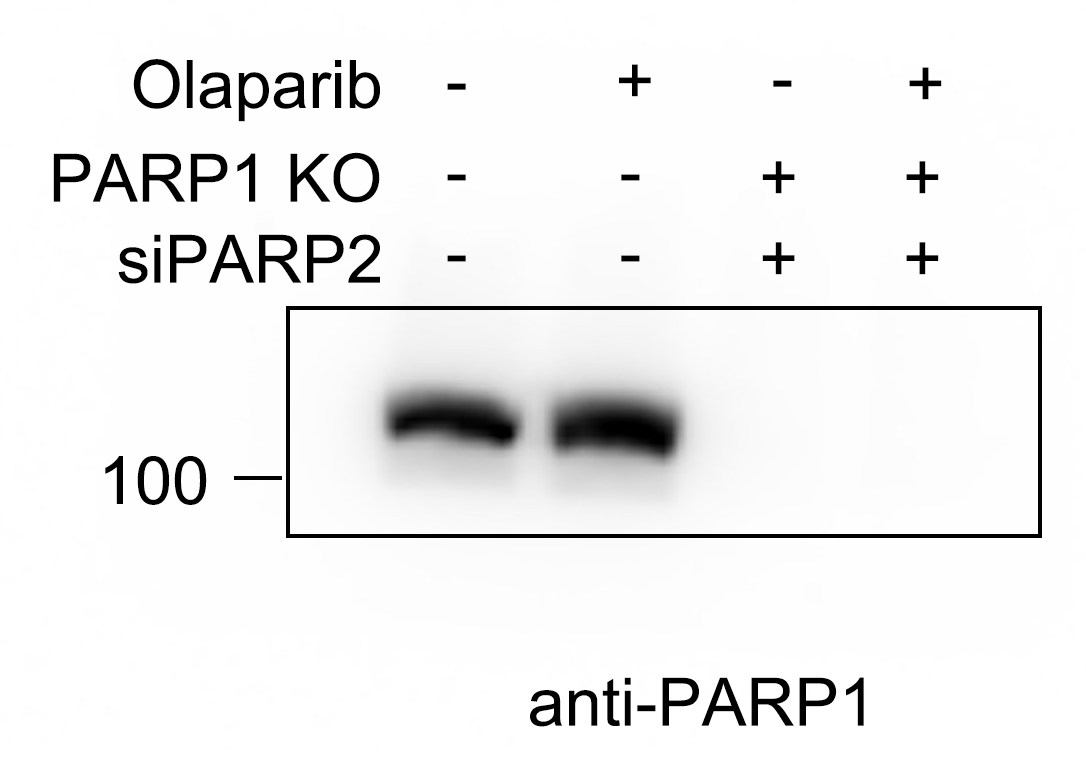

Supplement: Supplementary file 9 — Figure EV1-5 Source Data [file 44318_2025_641_MOESM9_ESM.zip › EMBOJ-2025-120713R_SourceDataForExpandedView/EMBOJ-2025-120713R_SourceDataForFigureEV5/FIG EV5B/siPARP2 WB/anti-PARP1 RawData.tif]

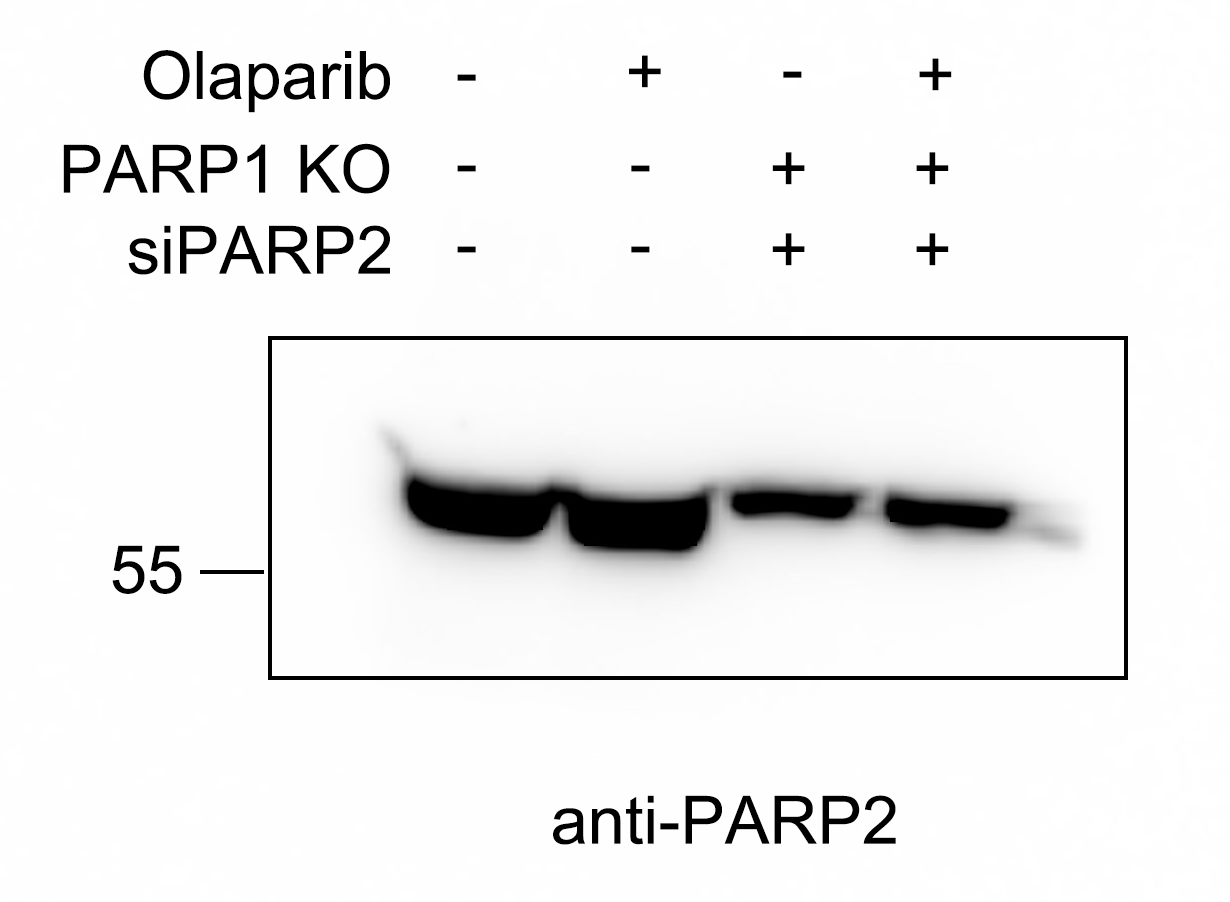

Supplement: Supplementary file 9 — Figure EV1-5 Source Data [file 44318_2025_641_MOESM9_ESM.zip › EMBOJ-2025-120713R_SourceDataForExpandedView/EMBOJ-2025-120713R_SourceDataForFigureEV5/FIG EV5B/siPARP2 WB/anti-PARP2 RawData.tif]

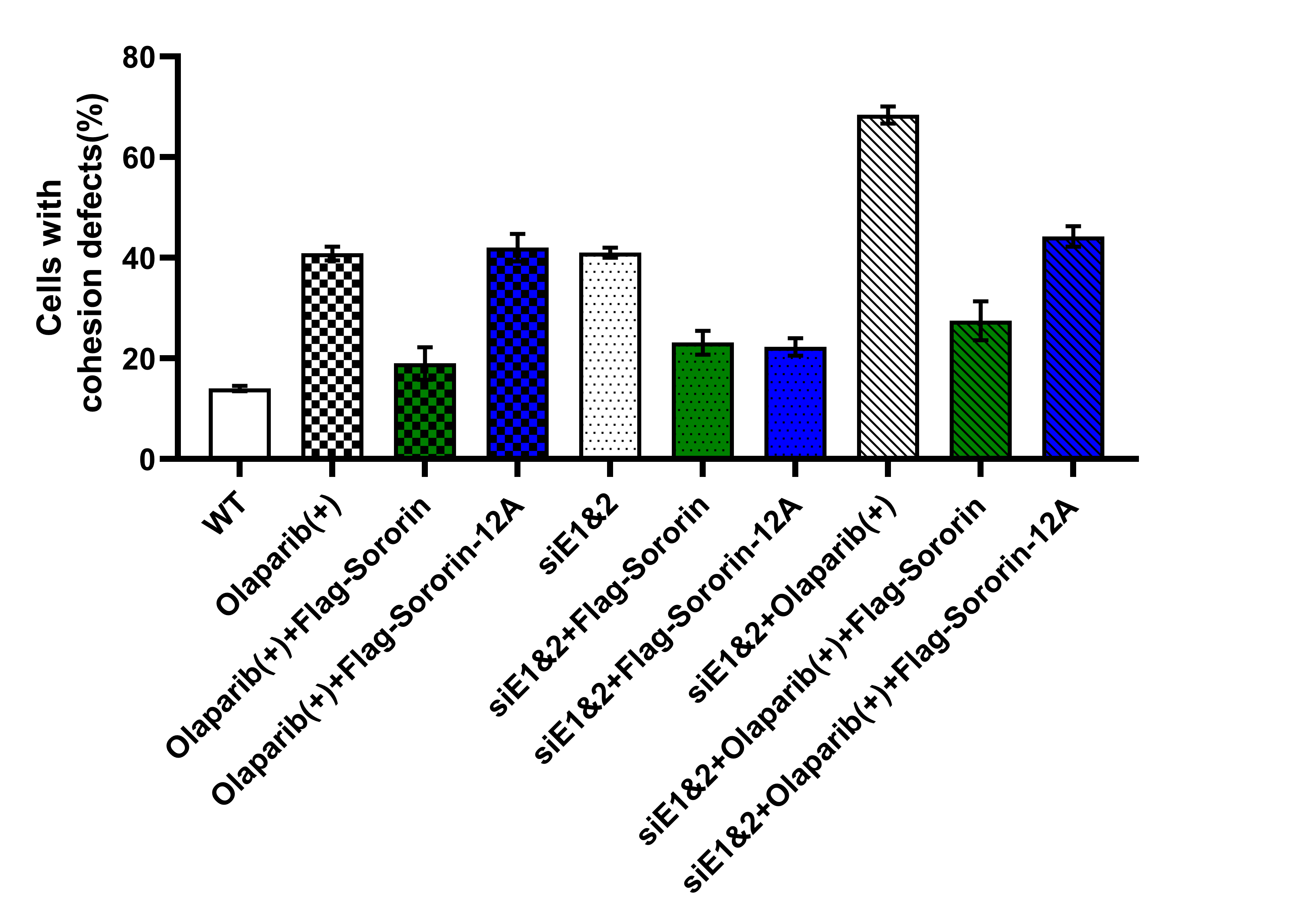

Supplement: Supplementary file 9 — Figure EV1-5 Source Data [file 44318_2025_641_MOESM9_ESM.zip › EMBOJ-2025-120713R_SourceDataForExpandedView/EMBOJ-2025-120713R_SourceDataForFigureEV5/FIG EV5C/FIG EV5C before PS.tif]

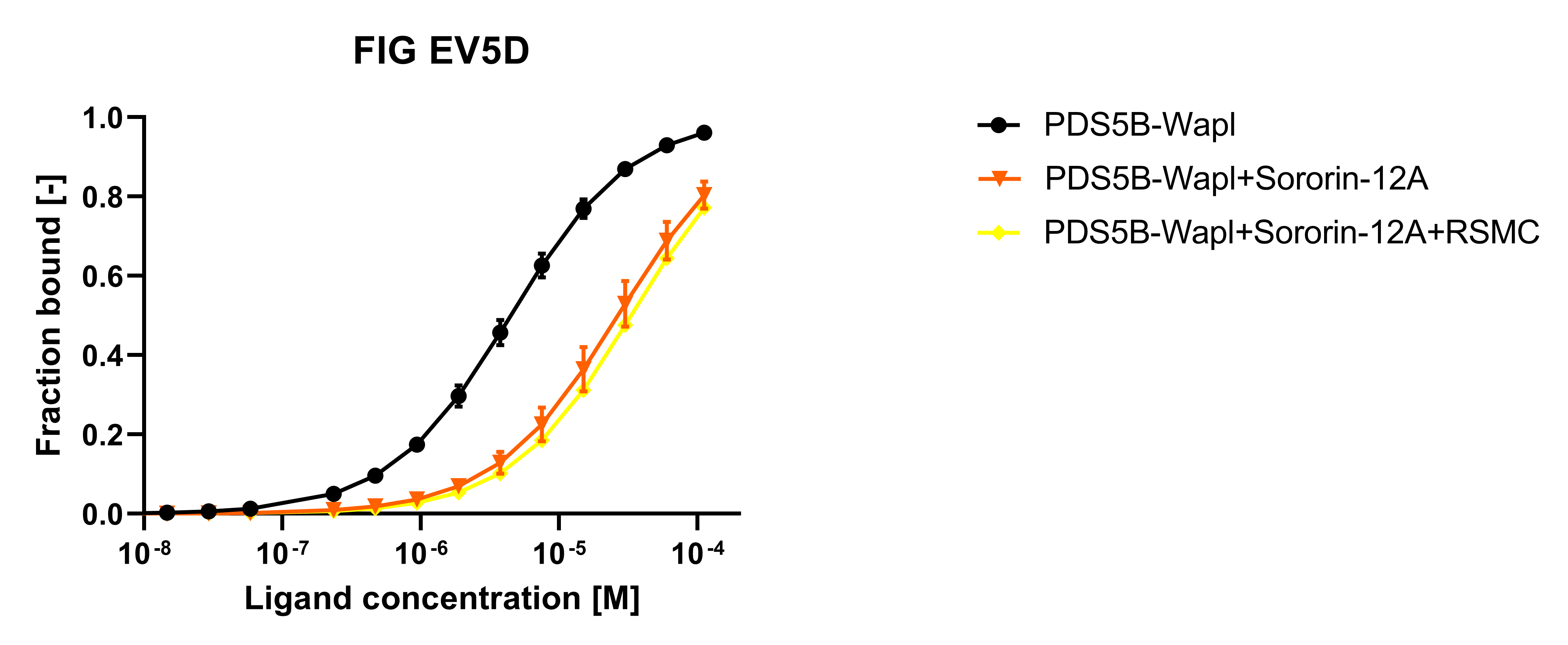

Supplement: Supplementary file 9 — Figure EV1-5 Source Data [file 44318_2025_641_MOESM9_ESM.zip › EMBOJ-2025-120713R_SourceDataForExpandedView/EMBOJ-2025-120713R_SourceDataForFigureEV5/FIG EV5D/FIG EV5D before PS.tif]

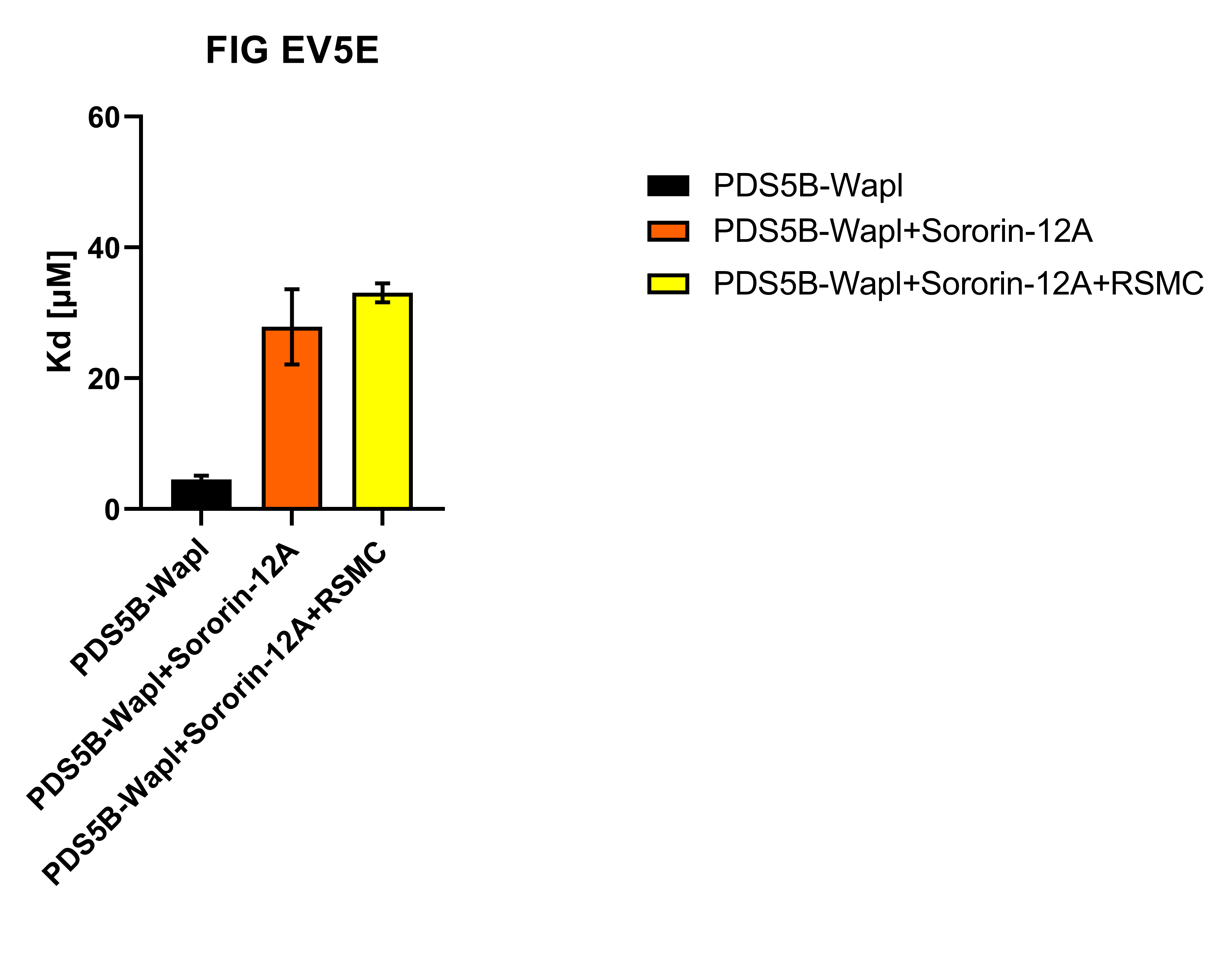

Supplement: Supplementary file 9 — Figure EV1-5 Source Data [file 44318_2025_641_MOESM9_ESM.zip › EMBOJ-2025-120713R_SourceDataForExpandedView/EMBOJ-2025-120713R_SourceDataForFigureEV5/FIG EV5E/FIG EV5E before PS.tif]
