# Supplementary material for: S-phase PARylation of microprotein RSMC enhances the function of Sororin in sister chromatid cohesion
Source: EMBO J. 2025 Nov 19;45(1):278–309. doi: 10.1038/s44318-025-00641-8 (PMC12759081; doi:10.1038/s44318-025-00641-8)
Supplement: Supplementary file 10 — Expanded View Figures [file 44318_2025_641_MOESM10_ESM.pdf]

Expanded View Figures

Raw mass spectrometry data

MS	MS-1		MS-2	
Protein	Score	emPAI	Score	emPAI
Sororin	3743	80.07	22605	81.51
SMC1A	264	0.56	305	0.24
SMC3	297	0.48	26	0.03
RAD21	135	0.42	61	0.13
STAG2	113	0.16	-	-
RSMC	73	0.79	30	0.34

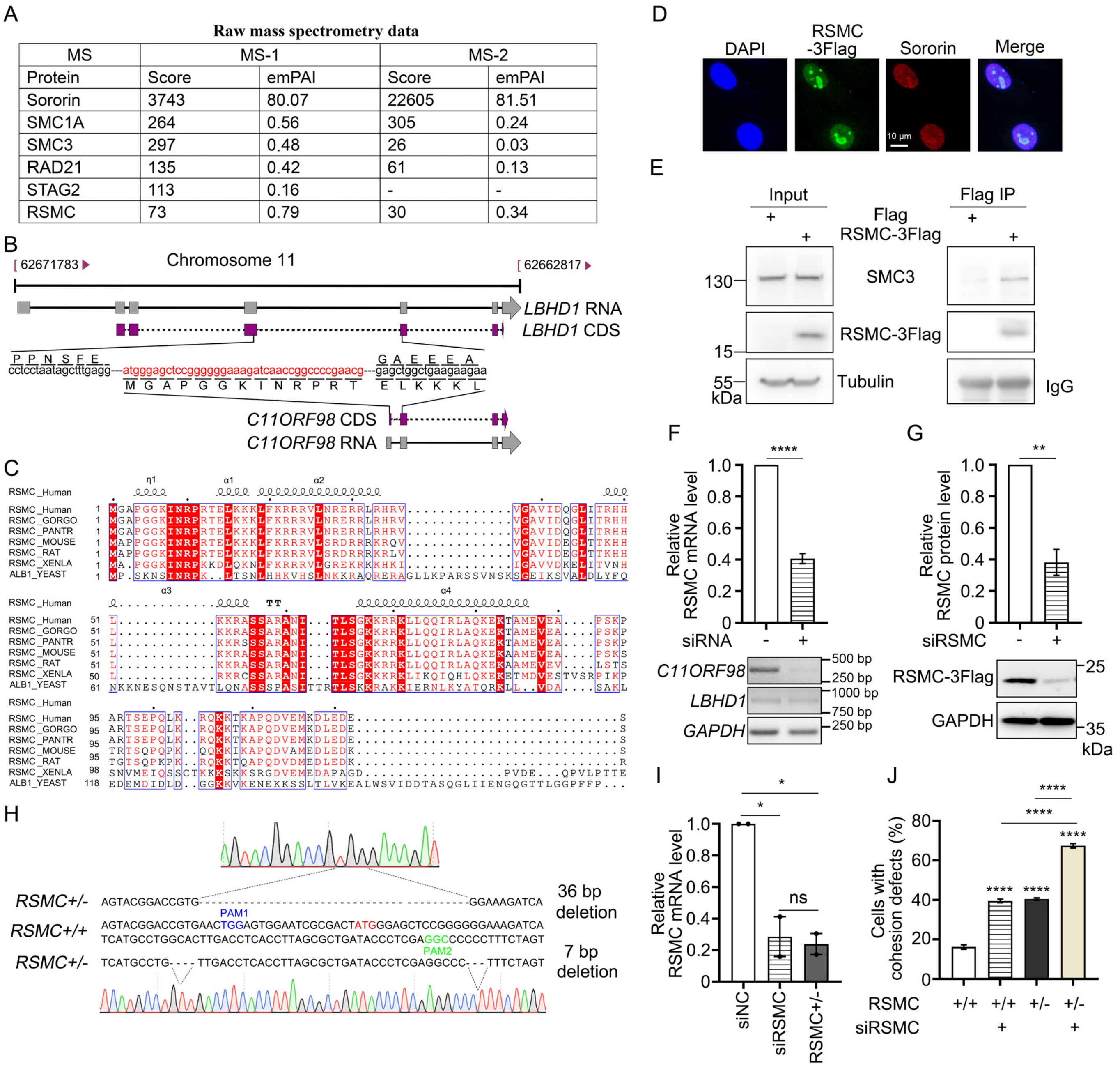

 © The Author(s)

Figure EV1. (related to Fig. 1). Identification of RSMC as a Sororin cofactor.

(**A**) Raw mass spectrometry data of Flag-Sororin immunoprecipitates (related to Fig. 1A). (**B**) The *C11ORF98* gene is an alternative open reading frame of the *LBHD1* gene. (**C**) RSMC is a conserved microprotein in eukaryotes. Sequence alignment of RSMC orthologs from the indicated species from T-coffee with the default setting and modified by ESPript 3.0. (**D**) Subcellular localization of RSMC. HeLa cells harboring endogenous RSMC-3Flag were synchronized at early S phase via double-thymidine block. After release into S phase for 6 h, cells were fixed and immunostained with the indicated antibodies. Nuclei were counterstained using DAPI. Scale Bar = 10 μm. (**E**) RSMC also interacts with the cohesin subunit SMC3. Co-immunoprecipitation (CoIP) assays were performed using RSMC-3Flag cells. Lysates were immunoprecipitated with anti-Flag M2 agarose, followed by immunoblotting with anti-SMC3 antibody to detect co-precipitated SMC3. 3% of samples were loaded as input. (**F**, **G**) Verification of the efficiency of RSMC (*C11ORF98*) KD by RNAi. (**F**) The mRNA level of RSMC was checked by qRT-PCR and compared to the *GAPDH* control. Note that *RSMC* mRNA was efficiently knocked down whereas the *LBHD1* mRNA level was not affected, Quantitative data are presented as mean ± SEM from four independent assays. The statistical significance was calculated via Student's *t* test. $^{****}P = 1.622e\text{-}6$. (**G**) The protein level of RSMC was measured by immunoblotting. RSMC-3Flag HeLa cells were transfected with RSMC siRNA for 48 h, then cells were collected and subjected to immunoblotting analysis. Quantitative data are presented as mean ± SEM from three independent assays. The statistical significance was calculated via Student's *t* test. $^{**}P = 0.0017$ (**G**). (**H**) Verification of CRISPR-Cas9-edited RSMC$^{+/-}$ clone by genomic sequencing. On-target deletion mutations were around the start codon of RSMC. (**I**) Real-time PCRs revealed that the RSMC$^{+/-}$ and RSMC siRNAs exhibited similar reduction of RSMC's mRNA levels. Quantitative data are presented as mean ± SEM from two independent assays. The statistical analyses were performed by one-way ANOVA with Tukey's post hoc test. siNC *vs* siRSMC $^{*}P = 0.0179$, siNC *vs* RSMC$^{+/-}$ $^{*}P = 0.0149$, siRSMC *vs* RSMC$^{+/-}$ $P = 0.9172$. (**J**) Combination of RSMC KD with RSMC$^{+/-}$ caused an additive cohesion defect. HEK293T WT and RSMC$^{+/-}$ cells were transfected with RSMC siRNA. After 48 h, mitotic cells were collected and subjected to chromosome spreads analysis. More than 300 mitotic cells from three independent assays were scored. Mean ± SEM are shown. The statistical analyses were performed by One-way ANOVA with Tukey's post hoc test. RSMC$^{+/+}$ *vs* RSMC$^{+/+}$+siRSMC $^{****}P = 6.0835e\text{-}7$, RSMC$^{+/+}$ *vs* RSMC$^{+/-}$ $^{****}P = 4.2887e\text{-}7$, RSMC$^{+/+}$ *vs* RSMC$^{+/-}$+siRSMC $^{****}P = 2.4757e\text{-}9$, RSMC$^{+/+}$+siRSMC *vs* RSMC$^{+/-}$+siRSMC $^{****}P = 1.1824e\text{-}7$, RSMC$^{+/-}$ *vs* RSMC$^{+/-}$+siRSMC $^{****}P = 1.6412e\text{-}7$. Source data are available online for this figure.

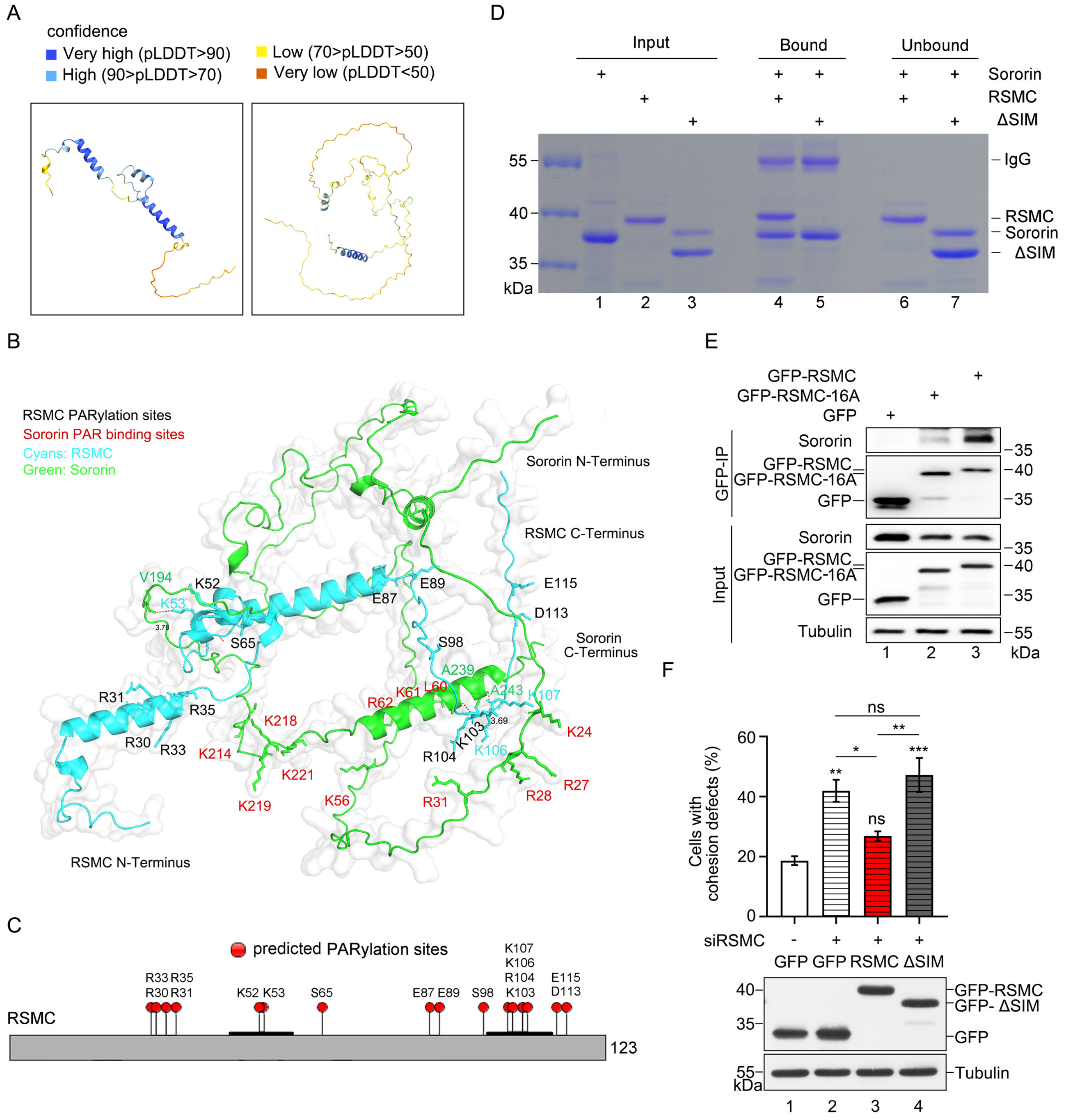
A
confidence
Very high (pLDDT>90)
High (90>pLDDT>70)
Low (70>pLDDT>50)
Very low (pLDDT<50)
D
Input
Bound
Unbound
Sororin
RSMC
ΔSIM
55
40
35
kDa
IgG
RSMC
Sororin
ΔSIM
1 2 3 4 5 6 7
B
RSMC PARylation sites
Sororin PAR binding sites
Cyans: RSMC
Green: Sororin
Sororin N-Terminus
RSMC C-Terminus
Sororin C-Terminus
RSMC N-Terminus
V194 K52 K53 S65 E87 E89 E115 D113 S98 A239 A243 K107 K24 K103 K106 R104 R27 R28 R31 K56 K219 K221 K214 K218 R62 K61 L60 R31 R35 R30 R33
E
GFP-RSMC
GFP-RSMC-16A
GFP
GFP-IP
Input
Sororin
GFP-RSMC
GFP-RSMC-16A
GFP
Tubulin
35
40
35
55
1 2 3 kDa
F
Cells with cohesion defects (%)
60
40
20
0
ns
*
**
***
siRSMC - + + +
GFP GFP RSMC ΔSIM
GFP-RSMC
GFP- ΔSIM
GFP
Tubulin
40
35
55
kDa
1 2 3 4
C
predicted PARylation sites
RSMC
R33 R35
R30 R31
K52 K53
S65
E87 E89
S98
K107
K106
R104
K103
E115
D113
123

Figure EV2. (related to Fig. 2). Mapping RSMC–Sororin interfaces.

(**A**) The RSMC and Sororin's structure were predicted by the α-Fold2 database. (**B**) The putative interacting interfaces between RSMC (cyan) and Sororin (green) predicted by GRAMM-X (https://gramm.compbio.ku.edu/). The predicted basal RSMC/Sororin interaction via hydrogen bonds was marked in the corresponding protein's color (RSMC K53, K106, K107, and Sororin V194, A239, A243). The predicted 16 PARylation sites of RSMC were labeled (K53, K106, K107, R30, R31, R33, R35, K52, S65, E87, E89, S98, K103, R104, D113, E115, in black). And the PAR-binding motifs of Sororin (related to Fig. EV5D) contain 12 amino acids (K24, R27, R28, R31, K56, L60, K61, R62, K214, K218, K219, K221, in red). (**C**) A schematic diagram of RSMC illustrating the RSMC–Sororin interfaces including black lines labeled a.a.46-60 and a.a.99-112, as well as their adjacent polar side chain residues (red dots) likely undergoing PARylation predicted by ADPredict. (https://www.adpredict.net/). (**D**) Mapping the interaction domains of RSMC with Sororin by pull-down assays. 6×His-Flag-Sororin was immobilized on anti-Flag M2 agarose. Then the GST-RSMC or its mutant proteins RSMCΔ46-60Δ99-112-10A (RSMCΔSIM) were incubated with immobilized Sororin. Sororin-bound proteins were separated by SDS-PAGE and detected by CBB. 30% of samples were loaded as input. (**E**) Compared to RSMC WT, RSMC-16A shows an attenuated interaction with Sororin. HEK293T cells were transfected with GFP-RSMC WT or GFP-RSMC-16A for 48 h. Lysates were immunoprecipitated with GBP beads. 3% of samples were loaded as input. (**F**) SIMs of RSMC are indispensable for its cohesion function. HEK293T cells transfected with RSMC siRNA and then with GFP, GFP-RSMC and GFP-RSMCΔSIM, respectively. Chromosome spreads analysis was conducted with four independent biological repeats as described in Fig. 1E. Data are presented as means ± SEM. The statistical significance was calculated via one-way ANOVA with Tukey's post hoc test. The expression of the indicated proteins was shown by immunoblotting. siRSMC(−)+GFP *vs* siRSMC(+)+GFP $^{**}P = 0.0028$, siRSMC(−)+GFP *vs* siRSMC(+)+RSMC $P = 0.3929$, siRSMC(−)+GFP *vs* siRSMC(+)+ΔSIM $^{***}P = 0.0005$, siRSMC(+)+GFP *vs* siRSMC(+)+RSMC $^{*}P = 0.0478$, siRSMC(+)+RSMC *vs* siRSMC(+)+ΔSIM $^{**}P = 0.0077$, siRSMC(+)+GFP *vs* siRSMC(+)+ΔSIM $P = 0.7272$. Source data are available online for this figure.

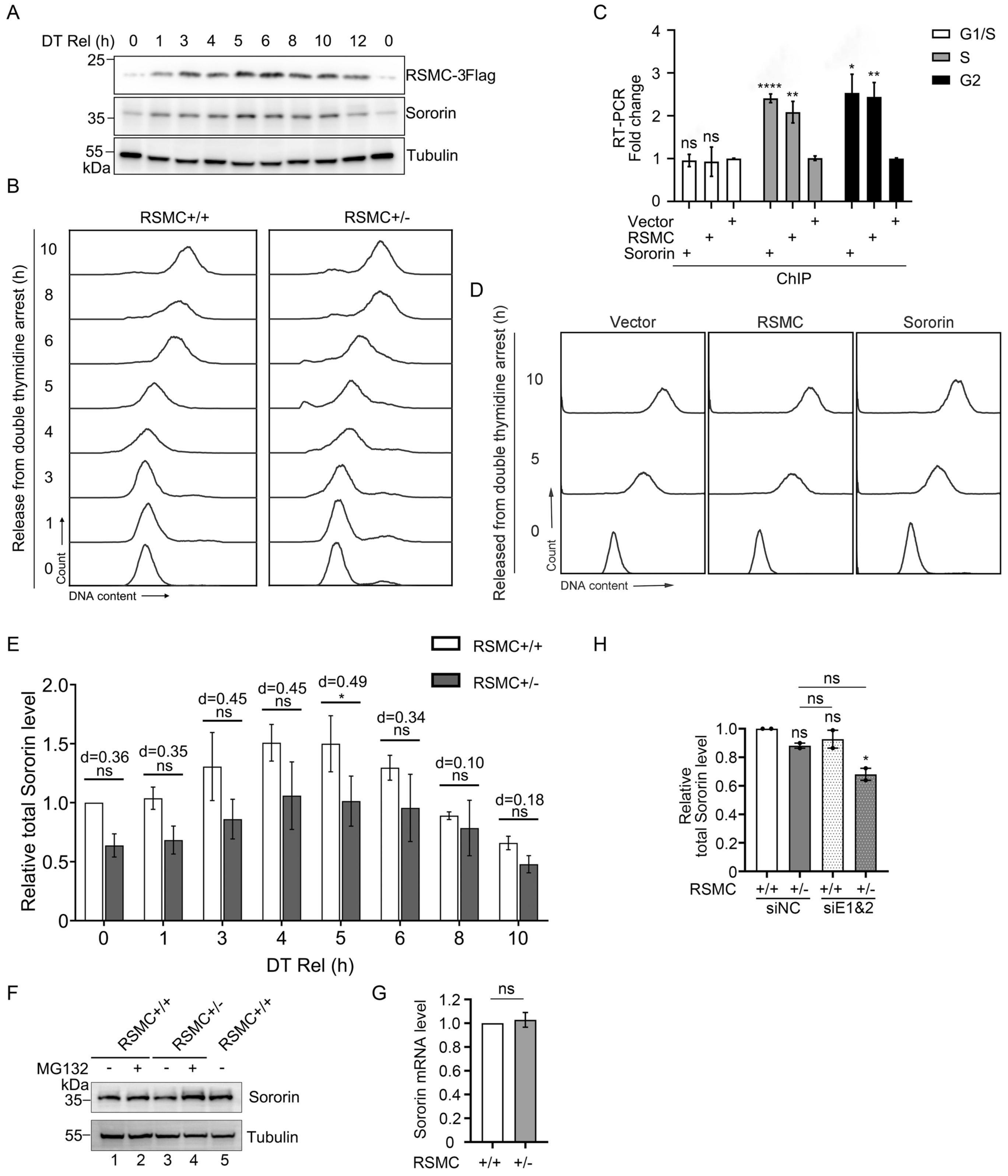
A
DT Rel (h) 0 1 3 4 5 6 8 10 12 0
RSMC-3Flag
Sororin
Tubulin
25
35
55
kDa
B
RSMC+/+
RSMC+/-
Release from double thymidine arrest (h)
Count
DNA content
C
RT-PCR Fold change
G1/S
S
G2
ns
****
**
Vector
RSMC
Sororin
ChIP
D
Vector
RSMC
Sororin
Released from double thymidine arrest (h)
Count
DNA content
E
RSMC+/+
RSMC+/-
Relative total Sororin level
d=0.36
d=0.35
d=0.45
d=0.45
d=0.49
d=0.34
d=0.10
d=0.18
DT Rel (h)
F
RSMC+/+
RSMC+/-
RSMC+/+
MG132
kDa
Sororin
Tubulin
G
Sororin mRNA level
RSMC +/+ +/-
H
Relative total Sororin level
RSMC +/+ +/- +/+ +/-
siNC
siE1&2

 © The Author(s)

Figure EV3. (related to Figs. 3 and 4). The cell-cycle-regulated RSMC protein levels and its contribution to Sororin stability.

(**A**) RSMC and Sororin exhibit similar fluctuations in protein levels throughout the cell cycle. RSMC-3Flag cells were harvested at the indicated time points following release from a double-thymidine block (early S phase). Immunoblotting analyses were performed using antibodies against the indicated proteins. (**B**) RSMC$^{+/-}$ shows a subtle effect on the cell cycle progression. HEK293T RSMC$^{+/+}$ and RSMC$^{+/-}$ cells were synchronized in early S phase by a double-thymidine block and harvested at the indicated time points. Cell samples were fixed and stained with PI and analyzed by flow cytometry (related to Fig. 3D). (**C**) Like Sororin, RSMC binds to specific cohesin-associated regions (CARs). HeLa cells transfected with 9Flag, 9Flag-RSMC, or Flag-Sororin were subjected to ChIP with anti-Flag M2 agarose. qPCR analysis was conducted using primers targeting known Sororin/cohesin-associated CARs (CARs, Human Chr8:134214868-134215746, related to Fig. 3C). Data are presented as means ± SEM from three independent assays. The statistical significance was calculated via two-way ANOVA with Tukey's post hoc test. *P* value from left to right, $p = 0.9550$, $P = 0.9731$, ****$P = 5.2387e\text{-}8$, **$P = 0.0084$, *$P = 0.0202$, **$P = 0.0068$. (**D**) Verification of the cell cycle progression. HeLa cells transfected with 9Flag, 9Flag-RSMC, or Flag-Sororin were synchronized in early S phase by a double-thymidine block and harvested at the indicated time points. Cell samples were fixed and stained with PI and analyzed by flow cytometry (related to Fig. 3C). (**E**) RSMC$^{+/-}$ reduces the total Sororin protein levels during S phase. The relative Sororin/H3 ratio of the whole-cell lysates was calculated from three independent assays. Data are presented as means ± SEM. The statistical significance was calculated via two-way ANOVA with Sidak's multiple comparisons test. The "d" represents the difference value of chromatin-bound Sororin in RSMC$^{+/+}$ and RSMC$^{+/-}$ (related to Fig. 3D). *P* value from left to right, $P = 0.1825$, $P = 0.2004$, $p = 0.0620$, $P = 0.0597$, *$P = 0.0359$, $P = 0.2369$, $P = 0.9948$, $P = 0.8797$. (**F**) The decreased Sororin in RSMC$^{+/-}$ was recovered by the proteasome inhibitor MG132 treatment for 5 h. (**G**) Sororin mRNA levels were not changed in RSMC$^{+/-}$. Data are presented as means ± SEM from three independent assays. The statistical significance was calculated via Student's *t* test. $P = 0.6681$. (**H**) Quantification of Sororin in whole-cell extracts. The relative Sororin/H3 ratio of WCE was calculated from two independent assays. Data are presented as means ± SEM and were normalized to siNC RSMC$^{+/+}$. The statistical significance was calculated via one-way ANOVA followed by Tukey's post hoc test. *P* value of RSMC$^{+/+}$(siNC) *vs* others (from left to right), $P = 0.2731$, $P = 0.5911$, *$P = 0.0146$; *P* value of RSMC$^{+/-}$(siNC) *vs* others (from left to right), $P = 0.8363$, $P = 0.0693$; RSMC$^{+/+}$(siE1&2) *vs* RSMC$^{+/-}$(siE1&2) *$P = 0.0359$. (related to Fig. 4C,D). Source data are available online for this figure.

© The Author(s)

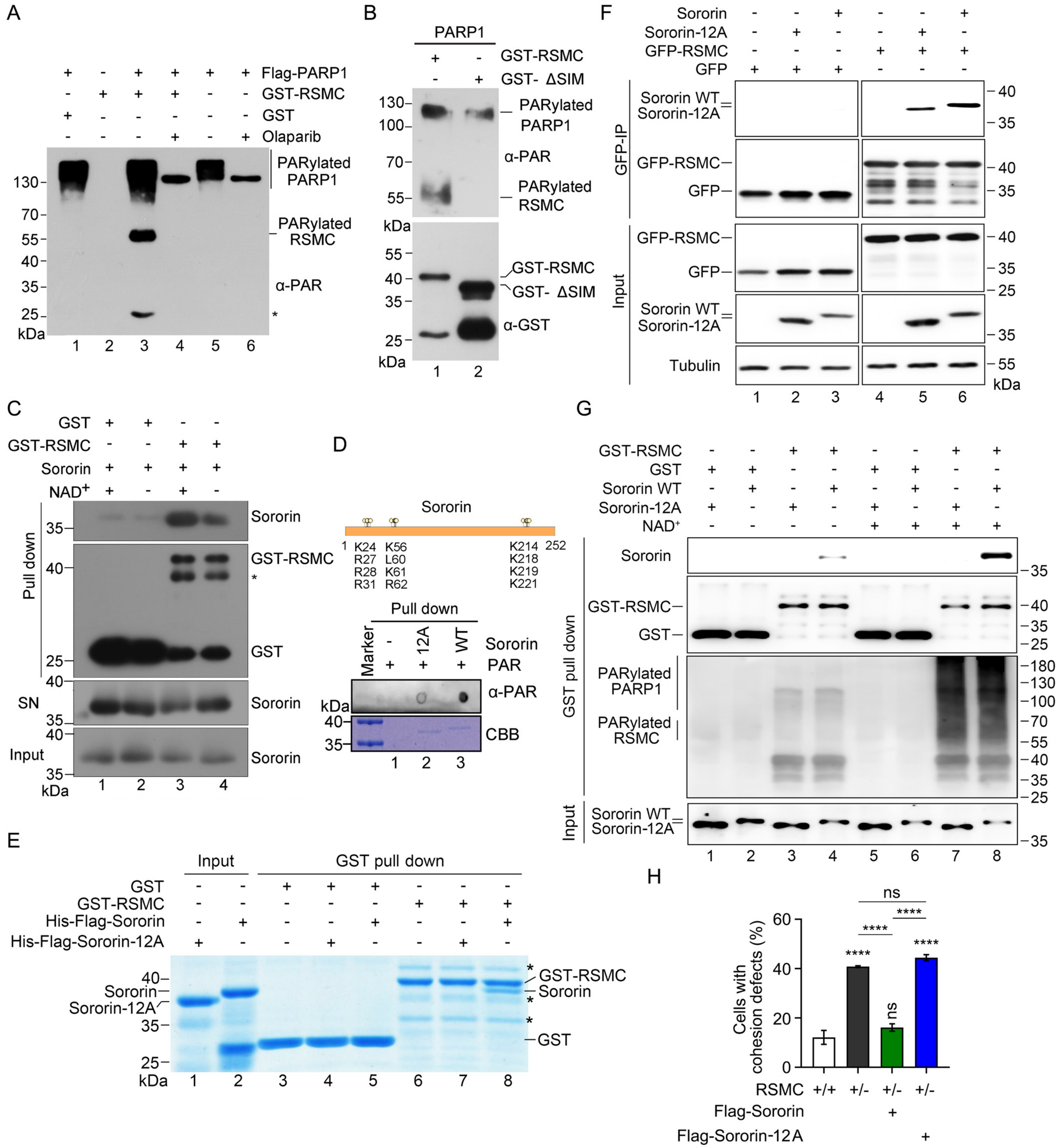
A
Flag-PARP1
GST-RSMC
GST
Olaparib
PARylated PARP1
PARylated RSMC
α-PAR
kDa
B
PARP1
GST-RSMC
GST- ΔSIM
PARylated PARP1
α-PAR
PARylated RSMC
GST-RSMC
GST- ΔSIM
α-GST
F
Sororin
Sororin-12A
GFP-RSMC
GFP
GFP-IP
Sororin WT
Sororin-12A
GFP-RSMC
GFP
Input
Tubulin
C
GST
GST-RSMC
Sororin
NAD+
Pull down
Sororin
GST-RSMC
GST
SN
Input
D
Sororin
K24 K56 K214 252
R27 L60 K218
R28 K61 K219
R31 R62 K221
Pull down
Marker
12A
WT
Sororin
PAR
α-PAR
CBB
G
GST-RSMC
GST
Sororin WT
Sororin-12A
NAD+
GST pull down
Sororin
GST-RSMC
GST
PARylated PARP1
PARylated RSMC
Input
Sororin WT
Sororin-12A
E
Input
GST pull down
GST
GST-RSMC
His-Flag-Sororin
His-Flag-Sororin-12A
Sororin
Sororin-12A
GST-RSMC
Sororin
GST
H
Cells with cohesion defects (%)
ns
****
RSMC +/+ +/- +/- +/-
Flag-Sororin
Flag-Sororin-12A

 © The Author(s)

Figure EV4. (related to Fig. 5). PARP1-mediated PARylation of RSMC enhances RSMC–Sororin interaction.

(**A**) The in vitro PARylation assay was performed using purified GST or GST-RSMC and Flag-PARP1 in the absence or presence of olaparib (20 μM) in the reaction buffer at 37 °C for 1 h. Immunoblotting was carried out with an anti-PAR monoclonal antibody. (**B**) RSMC is PARylated by PARP1 at SIMs. The in vitro PARylation assay was performed using purified GST-RSMC or GST-RSMCΔSIM and PARP1 in the reaction buffer at 37 °C for 1 h. Immunoblotting was carried out with an anti-PAR monoclonal antibody. (**C**) PARylation enhances RSMC's binding to Sororin. In vitro PARylation of RSMC was conducted as above in the absence or presence of NAD^+. After the removal of PARP1 and NAD^+, Sororin was incubated with immobilized RSMC. The resins were then washed three times, resuspended with 1×loading buffer and subjected to immunoblotting analyses with the indicated antibodies. (**D**) Diagram of Sororin with three PAR-binding motifs containing 12 residues and Sororin binds to PAR chains in vitro via 3 PAR-binding motifs. PAR (20 nM) was incubated with Sororin or Sororin-12A immobilized on Ni^{2+} and subjected to dot blot assays. (**E**) Sororin-12A is defective in binding RSMC in vitro. Immobilized GST-RSMC or GST (control) on glutathione beads was incubated with purified 6×His-Flag-Sororin WT or 12 A mutant. After washing, bound proteins were eluted and analyzed by SDS-PAGE followed by CBB staining. (**F**) Sororin-12A weakens its association with RSMC in cells. Cells were transfected with GFP, GFP-RSMC, Flag-Sororin WT, or Flag-Sororin-12A. Lysates were subjected to GFP-IP before immunoblotting with anti-Flag and anti-GFP antibodies. (**G**) Sororin-12A hardly binds PARylated RSMC in vitro. Immobilized RSMC was PARylated in the presence of NAD^+, then PARP1 and NAD^+ were washed away. Afterwards, PARylated RSMC was incubated with either Sororin WT or 12 A mutant. Bound proteins were analyzed by immunoblotting using anti-Sororin, anti-GST and anti-PAR antibodies. (**H**) Sororin-12A is defective in cohesion. Flag-tagged vector, Sororin WT, or Sororin-12A was transfected into HEK293T WT or $RSMC^{+/-}$ cells. Cohesion defects were quantified. Data are presented as means ± SEM from three independent assays. The statistical significance was calculated via one-way ANOVA followed by Tukey's post hoc test. P value of $RSMC^{+/+}$ *vs* others (from left to right), ****$P = 1.2454e\text{-}5$, $P = 0.4180$, ****$P = 5.0813e\text{-}6$; P value of $RSMC^{+/-}$ *vs* others (from left to right), ****$P = 3.8307e\text{-}5$, $P = 0.4946$; $RSMC^{+/-}$+Flag-Sororin *vs* $RSMC^{+/-}$+Flag-Sororin-12A ****$P = 1.3754e\text{-}5$. Source data are available online for this figure.

© The Author(s)

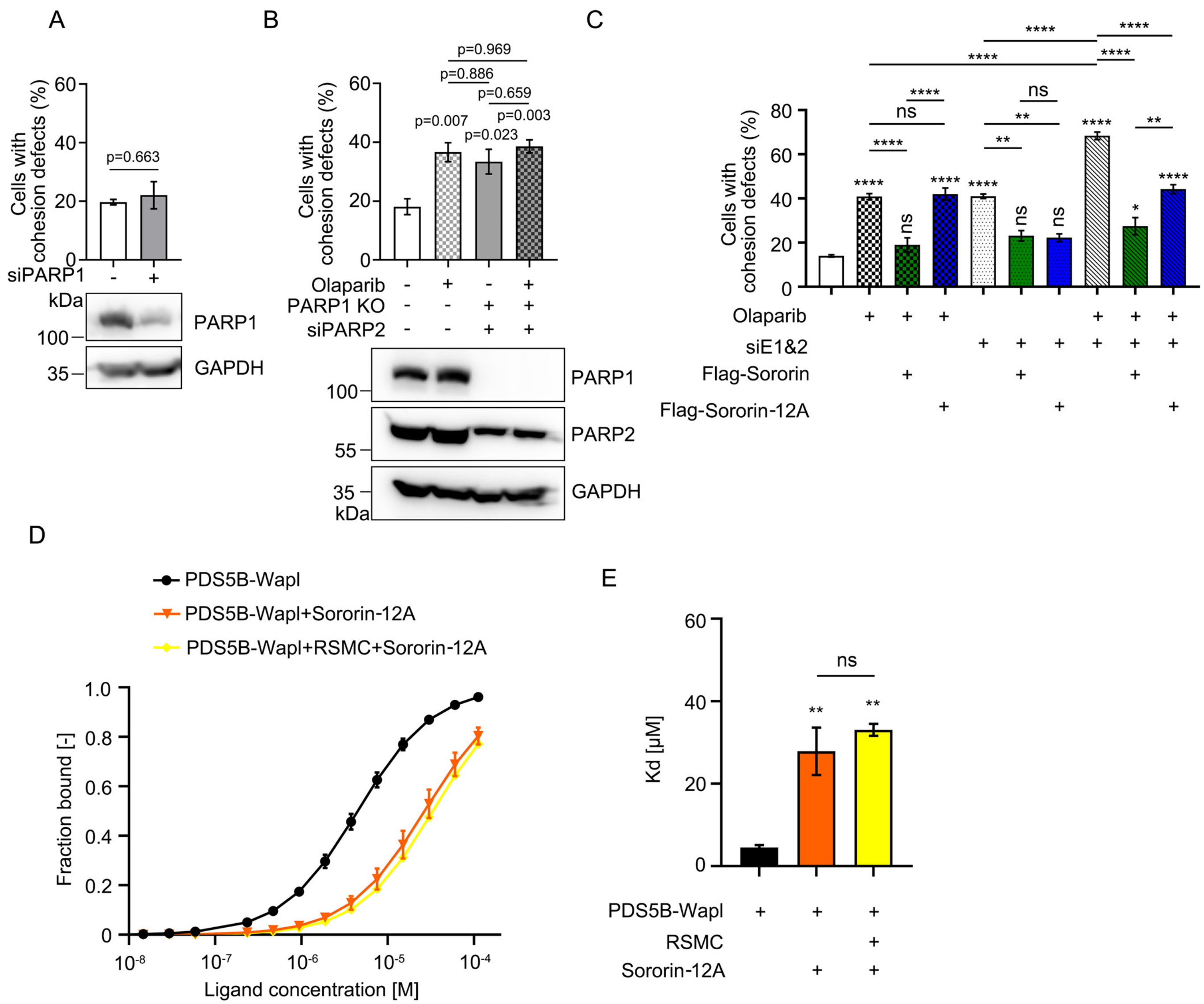

Figure EV5. (related to Figs. 6 and 7). PARPs cooperate with ESCO1/2 for Sororin functioning in cohesion.

(**A**) siPARP1 does not affect sister chromatid cohesion. Mitotic cells were collected after 48 h PARP1 RNAi and subjected to chromosome spread analyses. Sister chromatid cohesion defects were quantified from three independent assays. Immunoblots of PARP1 and GAPDH were shown below each column to confirm knockdown efficiency. Data represent mean ± SEM. The statistical significance was calculated via Student's *t* test. $P = 0.3984$. (**B**) PARP activity is required for cohesion. PARP1 KO HeLa cells were transfected with PARP2 or control siRNA with or without 10 μM olaparib treatment. Mitotic cells were analyzed by chromosome spreads after 48 h. More than 300 mitotic cells were scored from four independent assays. Immunoblots of PARP1, PARP2 and GAPDH were shown below each column to confirm knockout and knockdown efficiency. Mean ± SEM are shown. The statistical significance was calculated via one-way ANOVA followed by Tukey's post hoc test. *P* value of WT *vs* others (from left to right), $^{**}P = 0.0065$, $^{*}P = 0.0231$, $^{**}P = 0.0031$; *P* value of Olaparib(+) *vs* others (from left to right), $P = 0.8862$, $P = 0.9699$; PARP1 KO+siPARP2 *vs* PARP1 KO +siPARP2+Olaparib $P = 0.6589$. (**C**) PARPs function redundantly with ESCO1/2 in cohesion. HeLa cells transfected with ESCO1/2 or control siRNA treated ± 10 μM olaparib. Mitotic cells were analyzed by chromosome spreads after 48 h. Over 300 mitotic cells were scored from three independent assays. Mean ± SEM are shown. The statistical analyses were performed by One-way ANOVA followed by Tukey's post hoc test. *P* value of WT *vs* others (from left to right), $^{****}P = 4.3539\text{e-}6$, $P = 0.8503$, $^{****}P = 2.3505\text{e-}6$, $^{****}P = 4.0305\text{e-}6$, $P = 0.3061$, $P = 0.4265$, $^{****}P = 5.6290\text{e-}11$, $^{*}P = 0.0142$, $^{****}P = 7.6187\text{e-}7$; Olaparib(+) *vs* Olaparib(+)+Flag-Sororin $^{****}P = 7.2240\text{e-}5$, Olaparib(+) *vs* Olaparib(+)+Flag-Sororin-12A $P > 0.9999$, Olaparib(+) *vs* Olaparib(+)+siE1&2 $^{****}P = 3.0100\text{e-}6$; Olaparib(+)+Flag-Sororin *vs* Olaparib(+)+Flag-Sororin-12A$^{****}P = 3.6410\text{e-}5$; siE1&2 *vs* siE1&2+Flag-Sororin $^{**}P = 0.0028$, siE1&2 *vs* siE1&2+Flag-Sororin-12A$^{**}P = 0.0017$, siE1&2 *vs* siE1&2+Olaparib(+) $^{****}P = 3.2485\text{e-}6$; siE1&2+Flag-Sororin *vs* siE1&2+Flag-Sororin-12A $P > 0.9999$; siE1&2+Olaparib(+) *vs* siE1&2+Olaparib(+)+Flag-Sororin$^{****}P = 6.6151\text{e-}9$, siE1&2+Olaparib(+) *vs* siE1&2+Olaparib(+)+Flag-Sororin-12A $^{****}P = 1.9114\text{e-}5$; siE1&2+Olaparib(+)+Flag-Sororin *vs* siE1&2+Olaparib(+)+Flag-Sororin $^{**}P = 0.0017$. (**D**, **E**) Quantification of Wapl-PDS5B interactions by competitive microscale thermophoresis (MST). Cy5-labeled PDS5B (34 nM constant concentration) was titrated with 2-fold serially diluted Wapl (starting at 116 nM). (**D**) Binding curves and (**E**) calculated dissociation constants (Kd) under: control (black), 17 nM Sororin-12A (orange) and 17 nM each Sororin-12A and RSMC (yellow). Data represent mean ± SEM from three independent experiments. Statistical significance was determined by one-way ANOVA followed by Tukey's post hoc test. PDS5B-Wapl vs. PDS5B-Wapl+Sororin-12A $^{**}P = 0.0071$, PDS5B-Wapl vs. PDS5B-Wapl+Sororin-12A + RSMC $^{**}P = 0.0026$, PDS5B-Wapl+Sororin-12A vs. PDS5B-Wapl+Sororin-12A + RSMC $P = 0.5637$. Source data are available online for this figure.

 © The Author(s)
